# Supplementary material for: Seven-chain adaptive immune receptor repertoire analysis in rheumatoid arthritis reveals novel features associated with disease and clinically relevant phenotypes
Source: Genome Biol. 2024 Mar 11;25:68. doi: 10.1186/s13059-024-03210-0 (PMC10926600; doi:10.1186/s13059-024-03210-0)
Supplement: Supplementary file 3 — Additional file 3: Figure S1. Impact of technical and epidemiological variables on AIRR features. Evaluation of collinearity among technical variables (i.e., RNA concentration, RNA integrity, sequencing depth and plate), association of technical variables with AIRR features as well as association of epidemiological variables with AIRR features (i.e., adjusted by the influencing technical factors). [file 13059_2024_3210_MOESM3_ESM.pdf]

Fig S1. Impact of technical and epidemiological variables on AIRR features. Evaluation of collinearity among technical variables (i.e., RNA concentration, RNA integrity, sequencing depth and plate), association of technical variables with AIRR features as well as association of epidemiological variables with AIRR features (i.e., adjusted by the influencing technical factors).

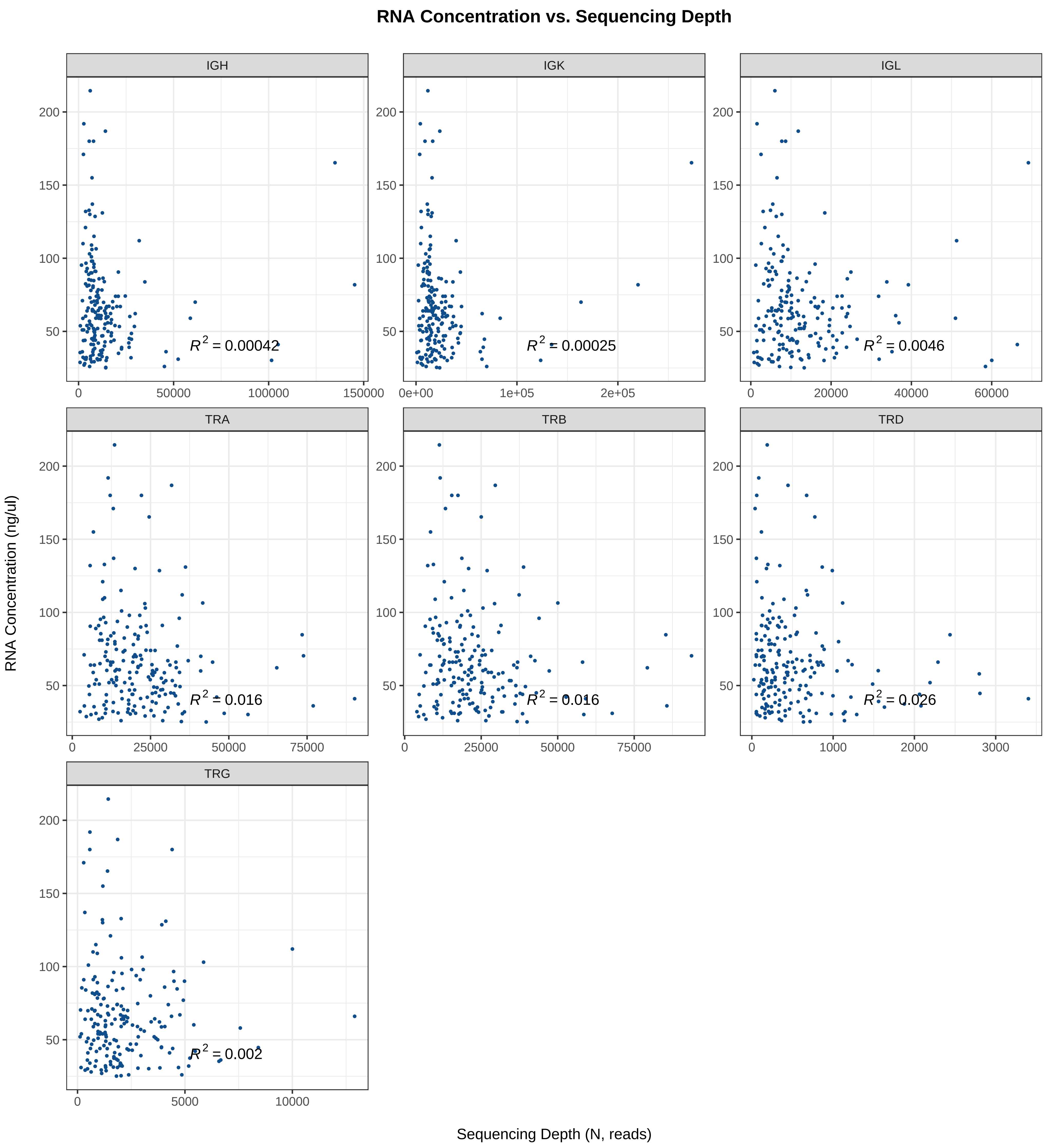

RNA Concentration vs. Sequencing Depth
IGH
IGK
IGL
TRA
TRB
TRD
TRG
R^2 = 0.00042
R^2 = 0.00025
R^2 = 0.0046
R^2 = 0.016
R^2 = 0.016
R^2 = 0.026
R^2 = 0.002
RNA Concentration (ng/ul)
Sequencing Depth (N, reads)

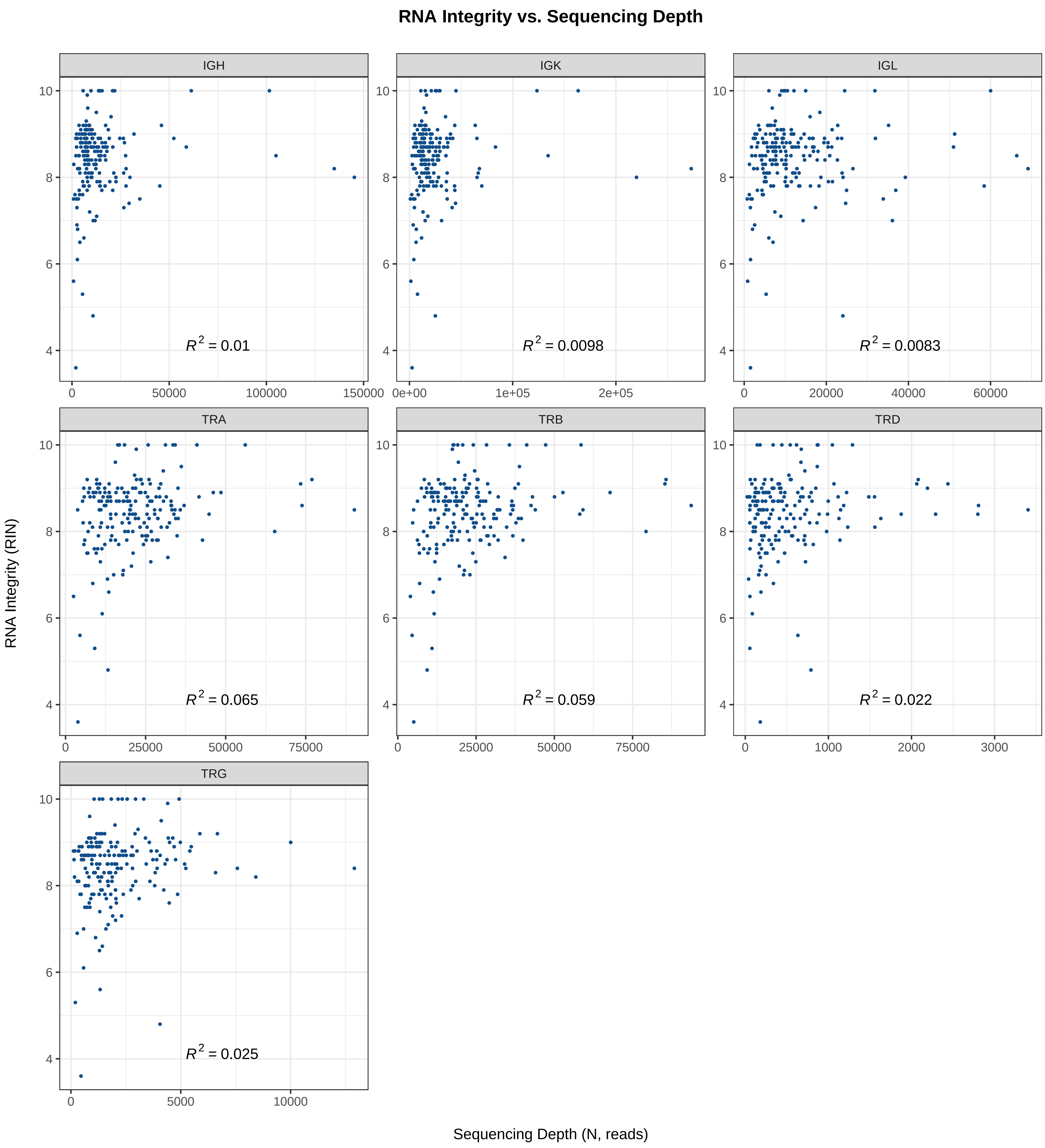

RNA Integrity vs. Sequencing Depth
IGH
IGK
IGL
TRA
TRB
TRD
TRG
$R^2 = 0.01$
$R^2 = 0.0098$
$R^2 = 0.0083$
$R^2 = 0.065$
$R^2 = 0.059$
$R^2 = 0.022$
$R^2 = 0.025$
RNA Integrity (RIN)
Sequencing Depth (N, reads)

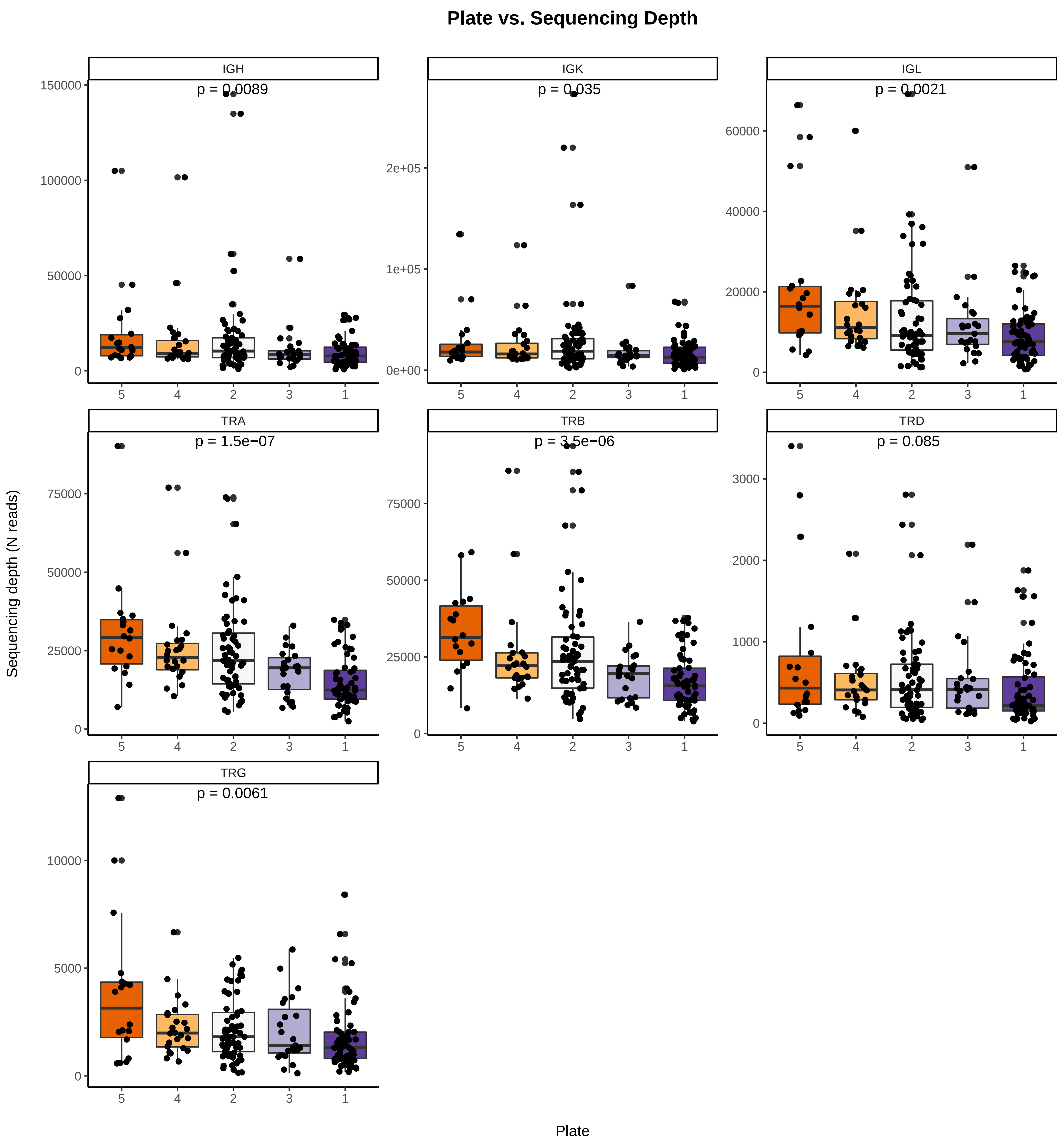
Plate vs. Sequencing Depth
IGH
p = 0.0089
IGK
p = 0.035
IGL
p = 0.0021
TRA
p = 1.5e−07
TRB
p = 3.5e−06
TRD
p = 0.085
TRG
p = 0.0061
Sequencing depth (N reads)
Plate

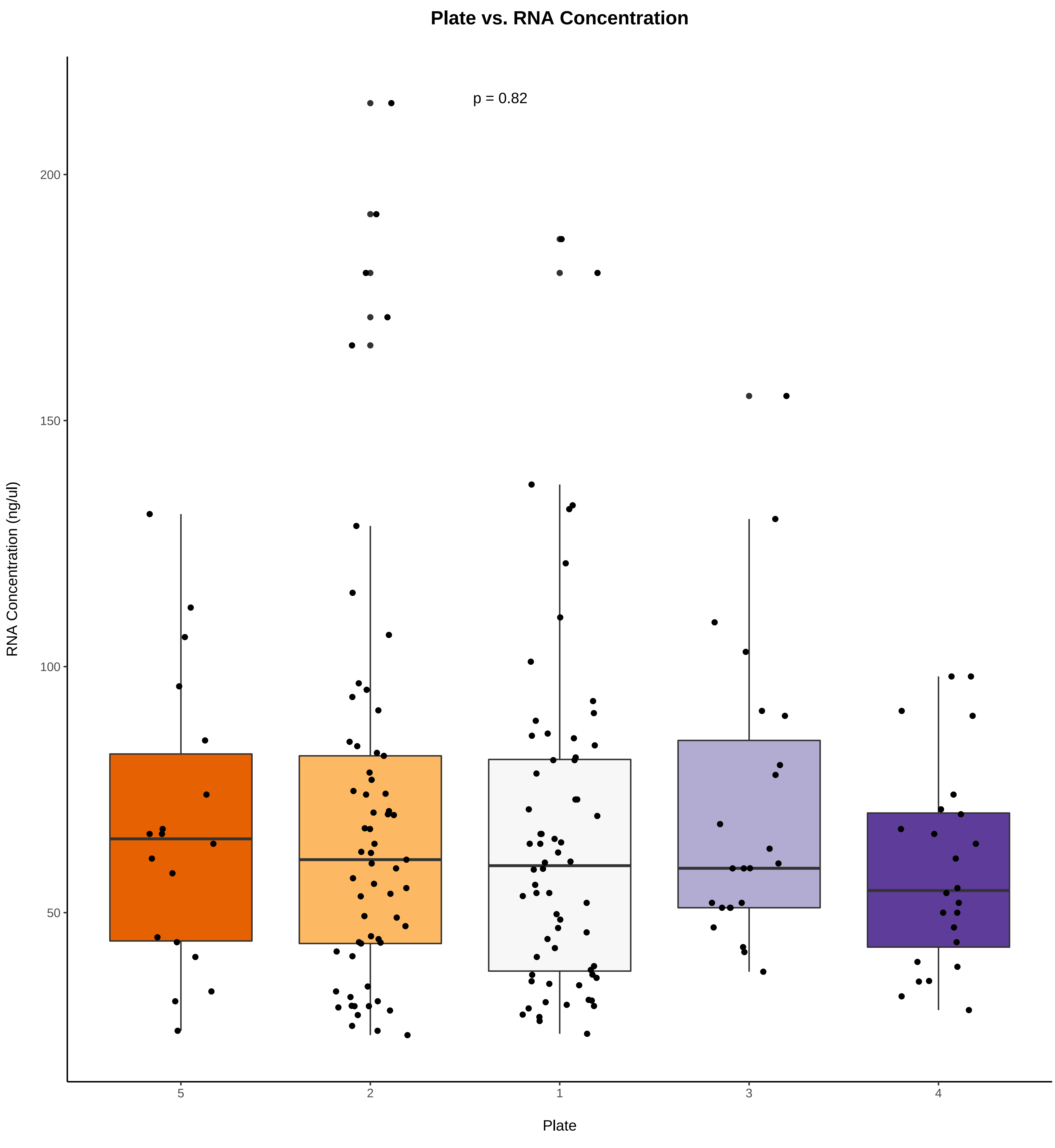

Plate vs. RNA Concentration
p = 0.82
RNA Concentration (ng/ul)
200
150
100
50
5
2
1
3
4
Plate

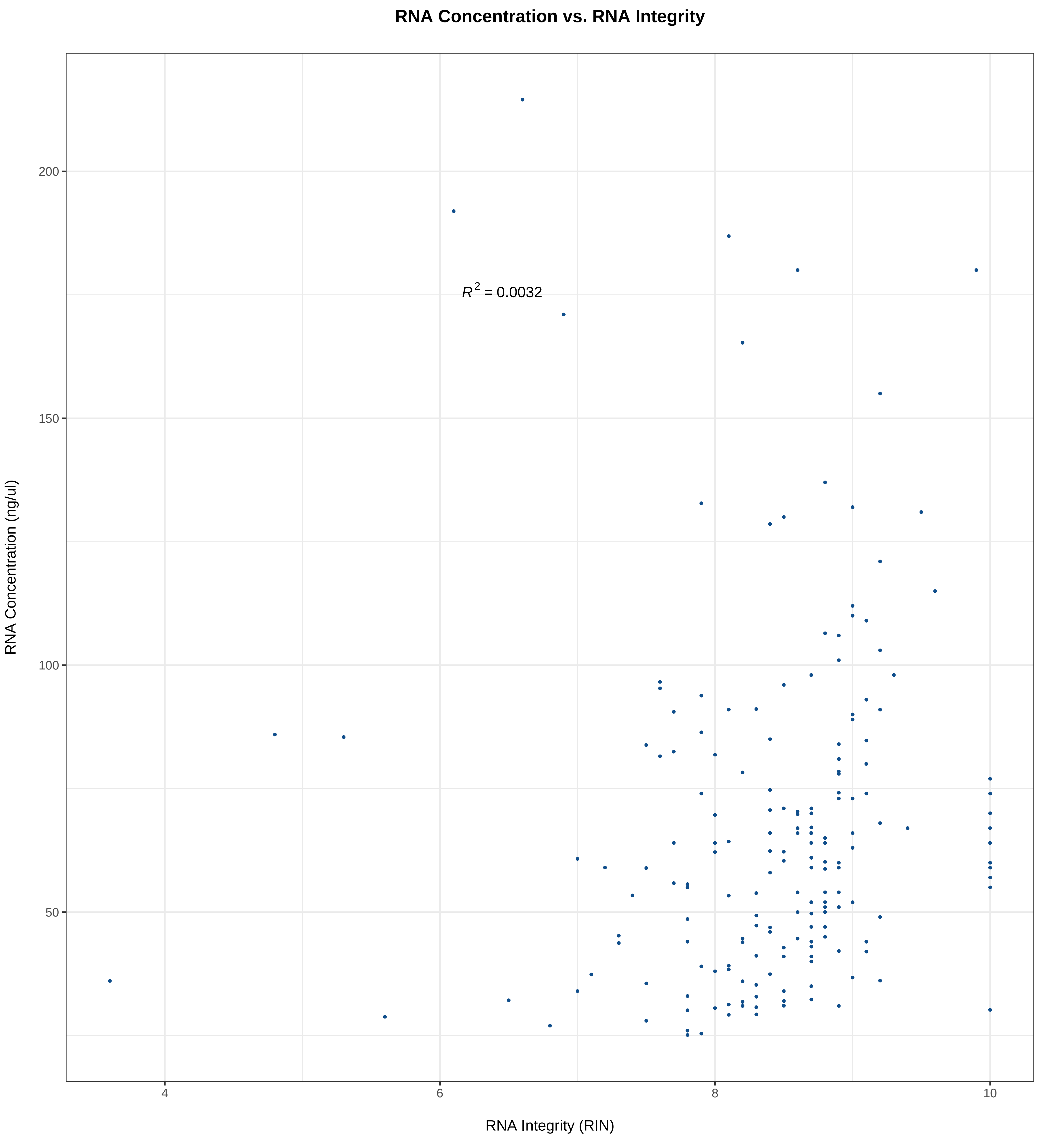

RNA Concentration vs. RNA Integrity
$R^2 = 0.0032$
RNA Concentration (ng/ul)
200
150
100
50
4
6
8
10
RNA Integrity (RIN)

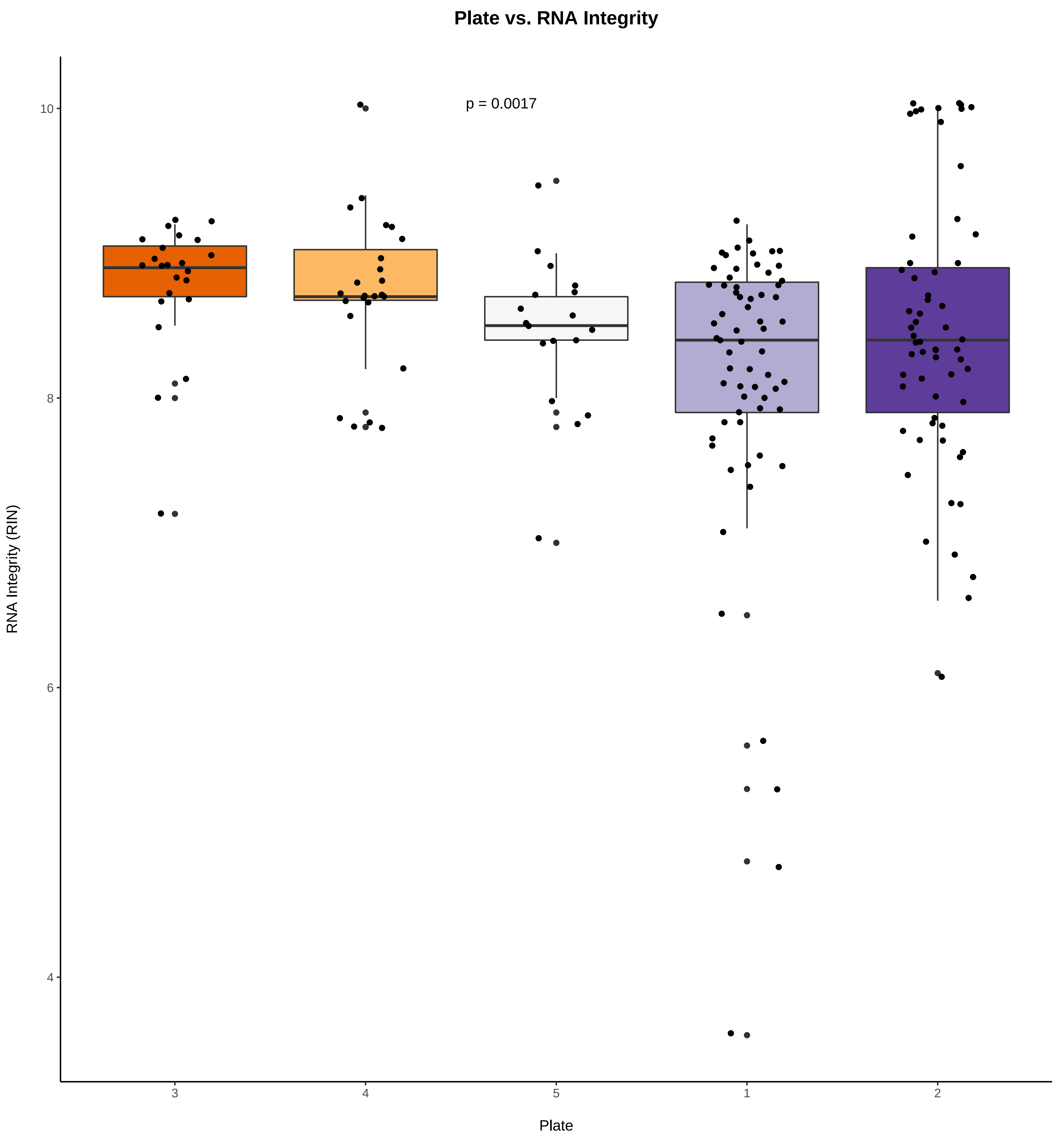

Plate vs. RNA Integrity
p = 0.0017
RNA Integrity (RIN)
10
8
6
4
3
4
5
1
2
Plate

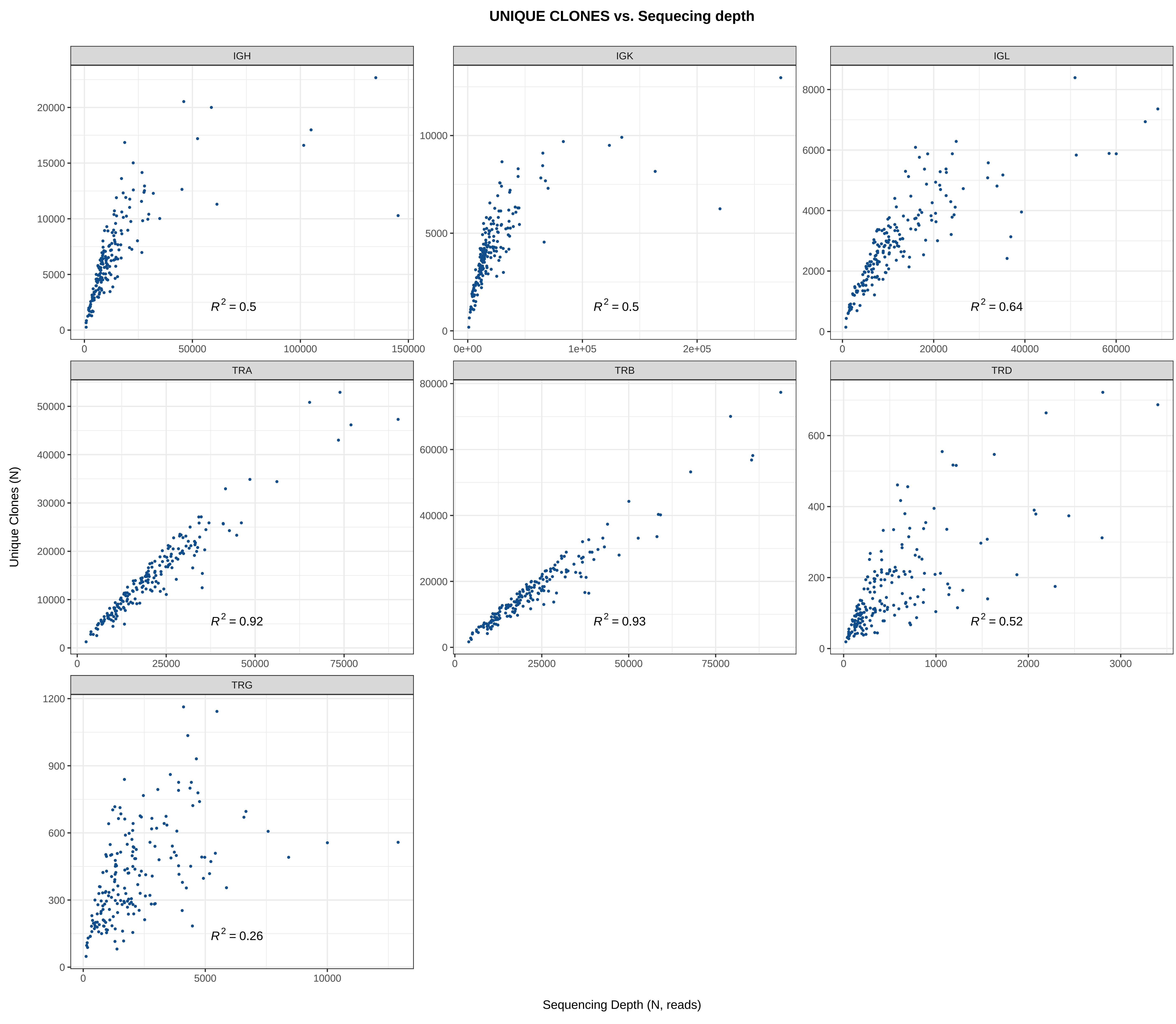
UNIQUE CLONES vs. Sequecing depth
IGH
IGK
IGL
TRA
TRB
TRD
TRG
$R^2 = 0.5$
$R^2 = 0.5$
$R^2 = 0.64$
$R^2 = 0.92$
$R^2 = 0.93$
$R^2 = 0.52$
$R^2 = 0.26$
Unique Clones (N)
Sequencing Depth (N, reads)

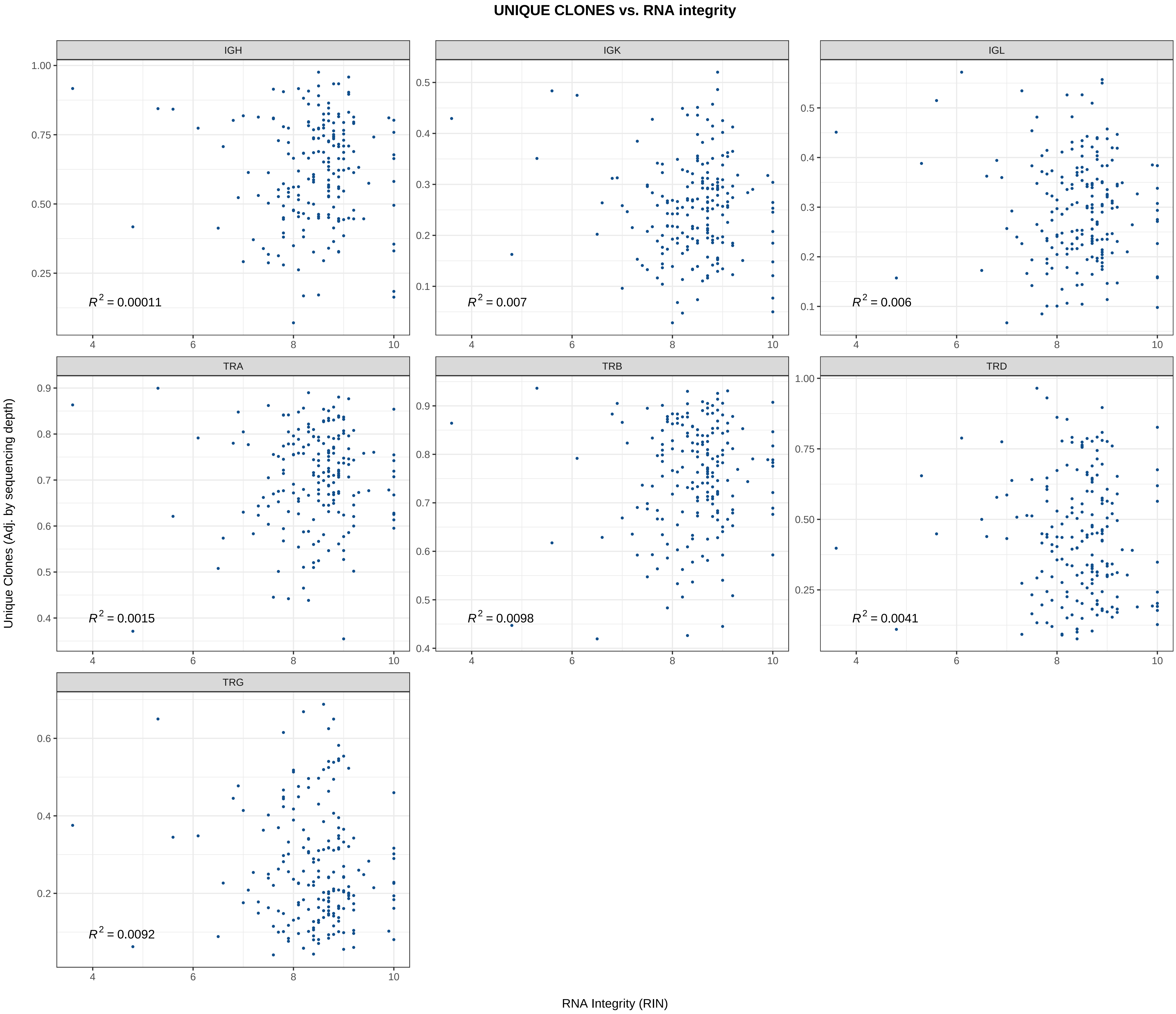
UNIQUE CLONES vs. RNA integrity
IGH
IGK
IGL
TRA
TRB
TRD
TRG
R^2 = 0.00011
R^2 = 0.007
R^2 = 0.006
R^2 = 0.0015
R^2 = 0.0098
R^2 = 0.0041
R^2 = 0.0092
Unique Clones (Adj. by sequencing depth)
RNA Integrity (RIN)

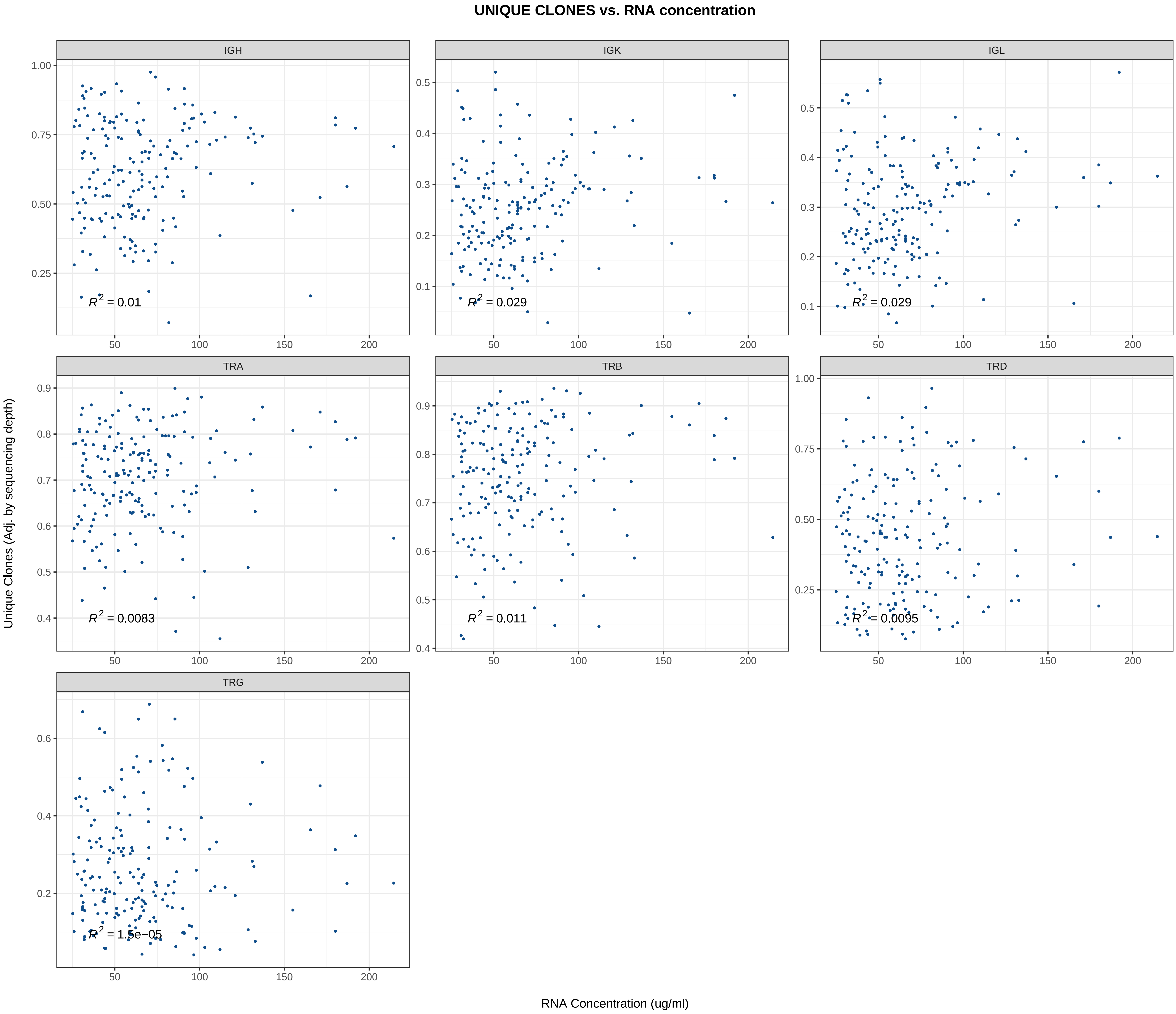
UNIQUE CLONES vs. RNA concentration
IGH
IGK
IGL
TRA
TRB
TRD
TRG
R^2 = 0.01
R^2 = 0.029
R^2 = 0.029
R^2 = 0.0083
R^2 = 0.011
R^2 = 0.0095
R^2 = 1.5e−05
Unique Clones (Adj. by sequencing depth)
RNA Concentration (ug/ml)

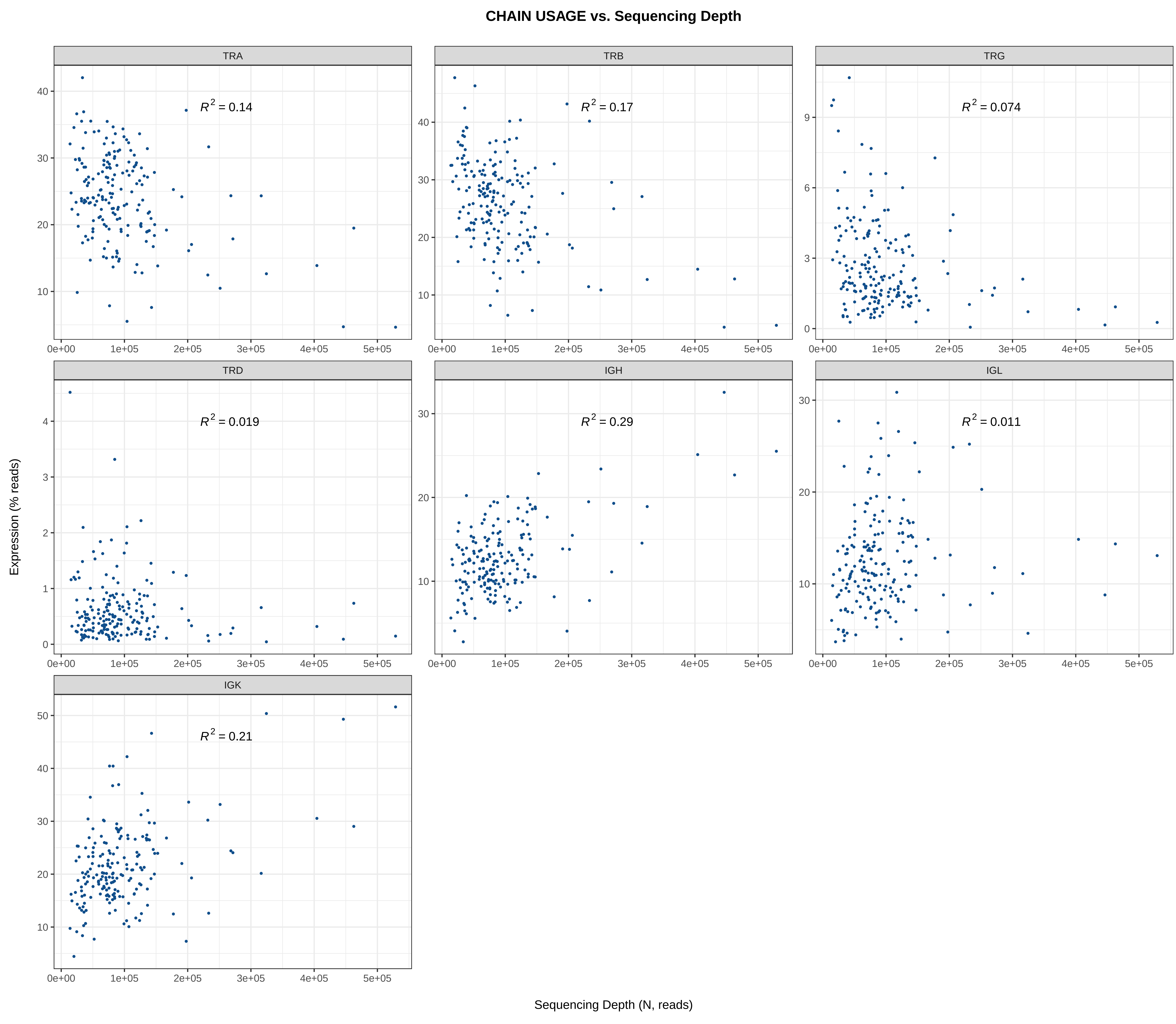

CHAIN USAGE vs. Sequencing Depth
TRA
$R^2 = 0.14$
TRB
$R^2 = 0.17$
TRG
$R^2 = 0.074$
TRD
$R^2 = 0.019$
IGH
$R^2 = 0.29$
IGL
$R^2 = 0.011$
IGK
$R^2 = 0.21$
Expression (% reads)
Sequencing Depth (N, reads)
0e+00
1e+05
2e+05
3e+05
4e+05
5e+05

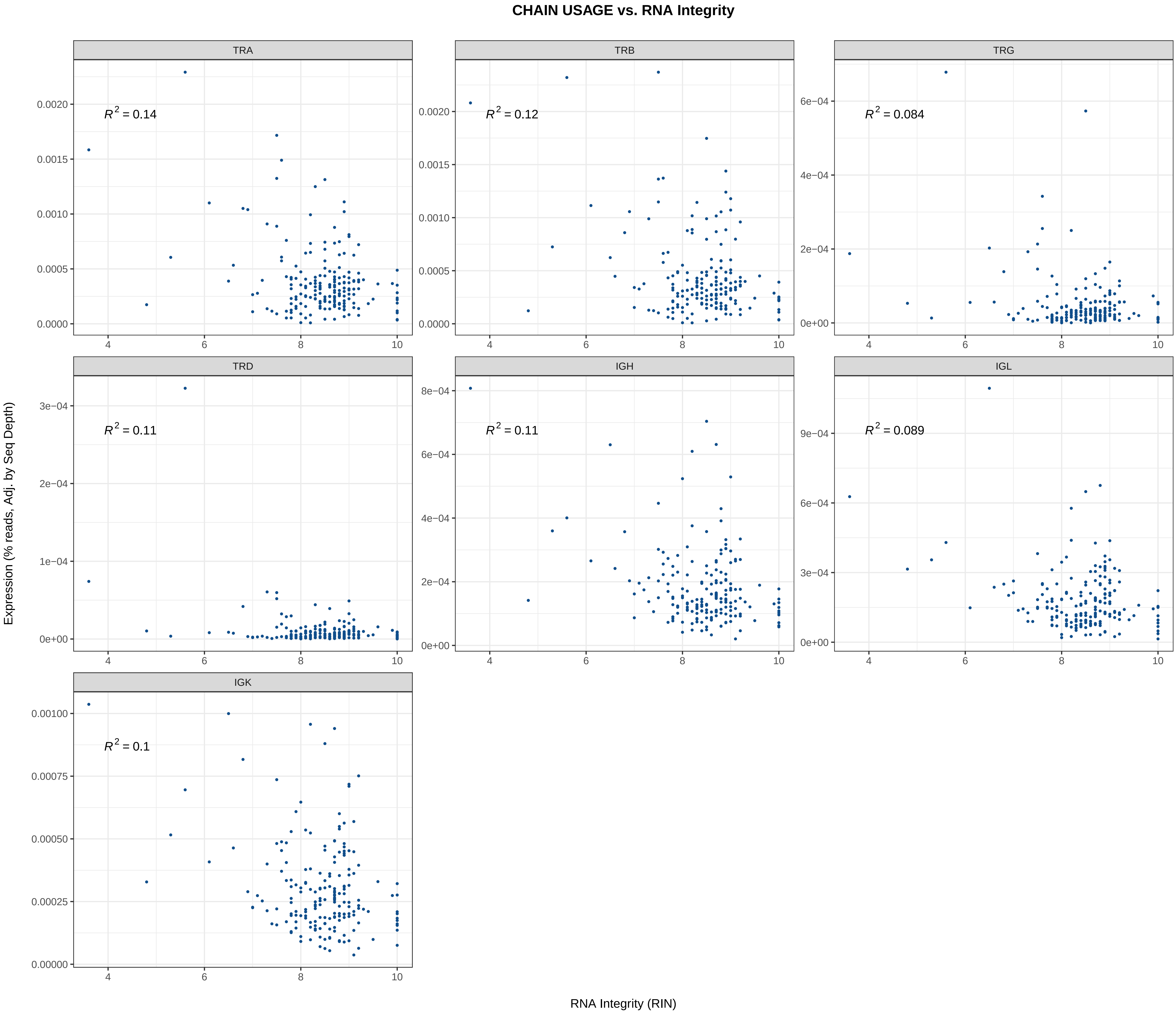
CHAIN USAGE vs. RNA Integrity
TRA
$R^2 = 0.14$
TRB
$R^2 = 0.12$
TRG
$R^2 = 0.084$
TRD
$R^2 = 0.11$
IGH
$R^2 = 0.11$
IGL
$R^2 = 0.089$
IGK
$R^2 = 0.1$
Expression (% reads, Adj. by Seq Depth)
RNA Integrity (RIN)

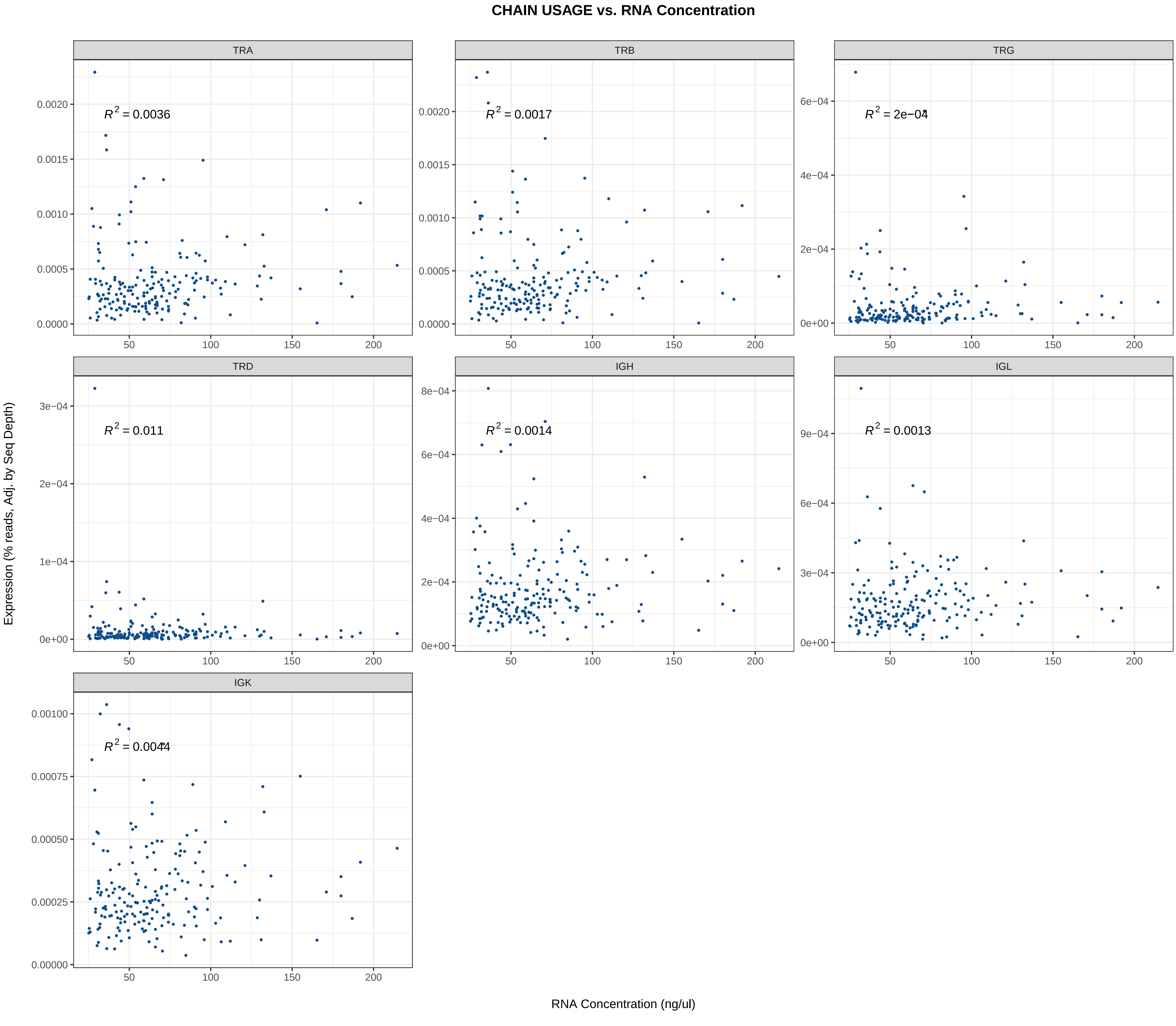

CHAIN USAGE vs. RNA Concentration
TRA
$R^2 = 0.0036$
TRB
$R^2 = 0.0017$
TRG
$R^2 = 2e-04$
TRD
$R^2 = 0.011$
IGH
$R^2 = 0.0014$
IGL
$R^2 = 0.0013$
IGK
$R^2 = 0.0044$
Expression (% reads, Adj. by Seq Depth)
RNA Concentration (ng/ul)

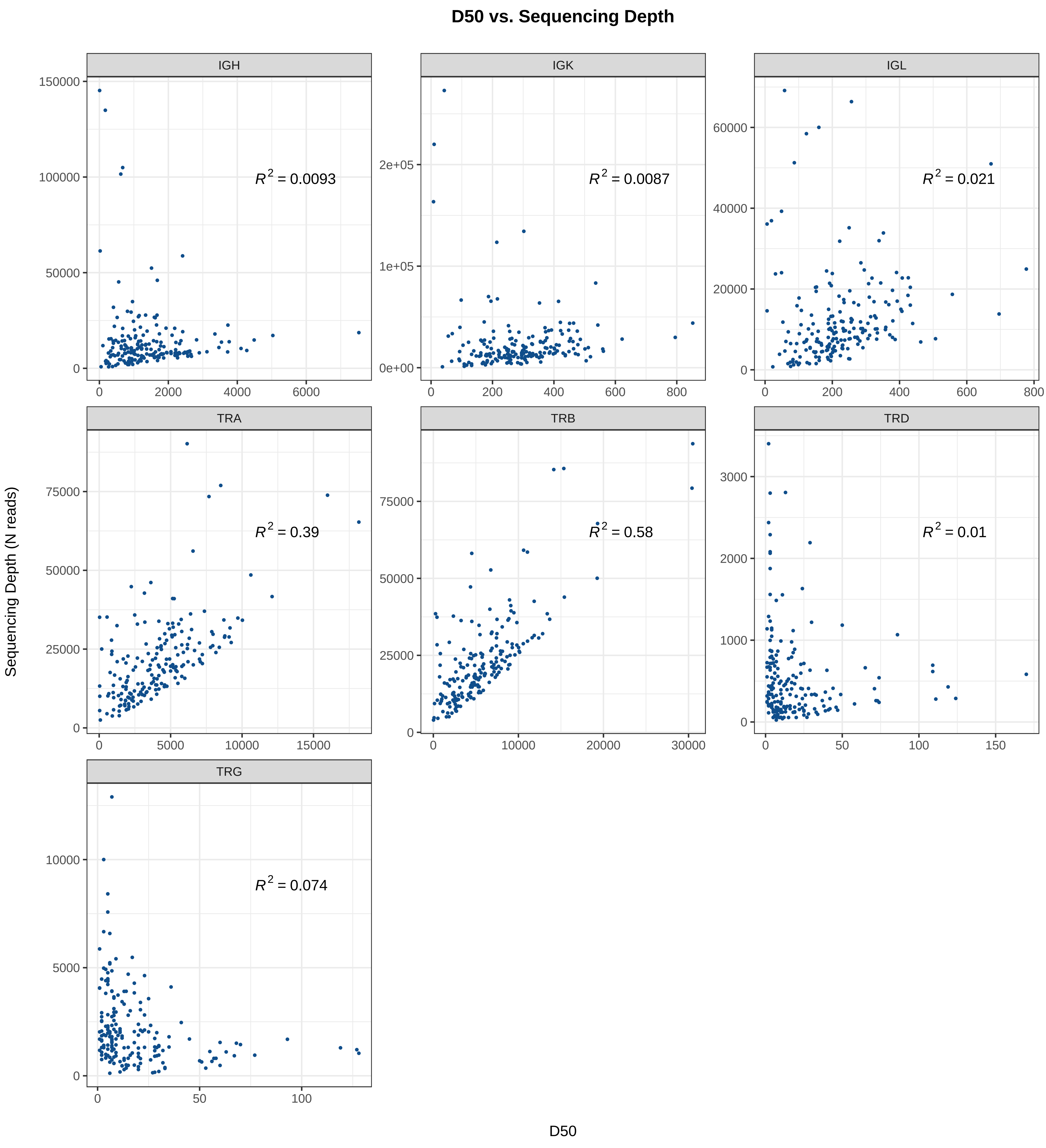
D50 vs. Sequencing Depth
IGH
R^2 = 0.0093
IGK
R^2 = 0.0087
IGL
R^2 = 0.021
TRA
R^2 = 0.39
TRB
R^2 = 0.58
TRD
R^2 = 0.01
TRG
R^2 = 0.074
Sequencing Depth (N reads)
D50

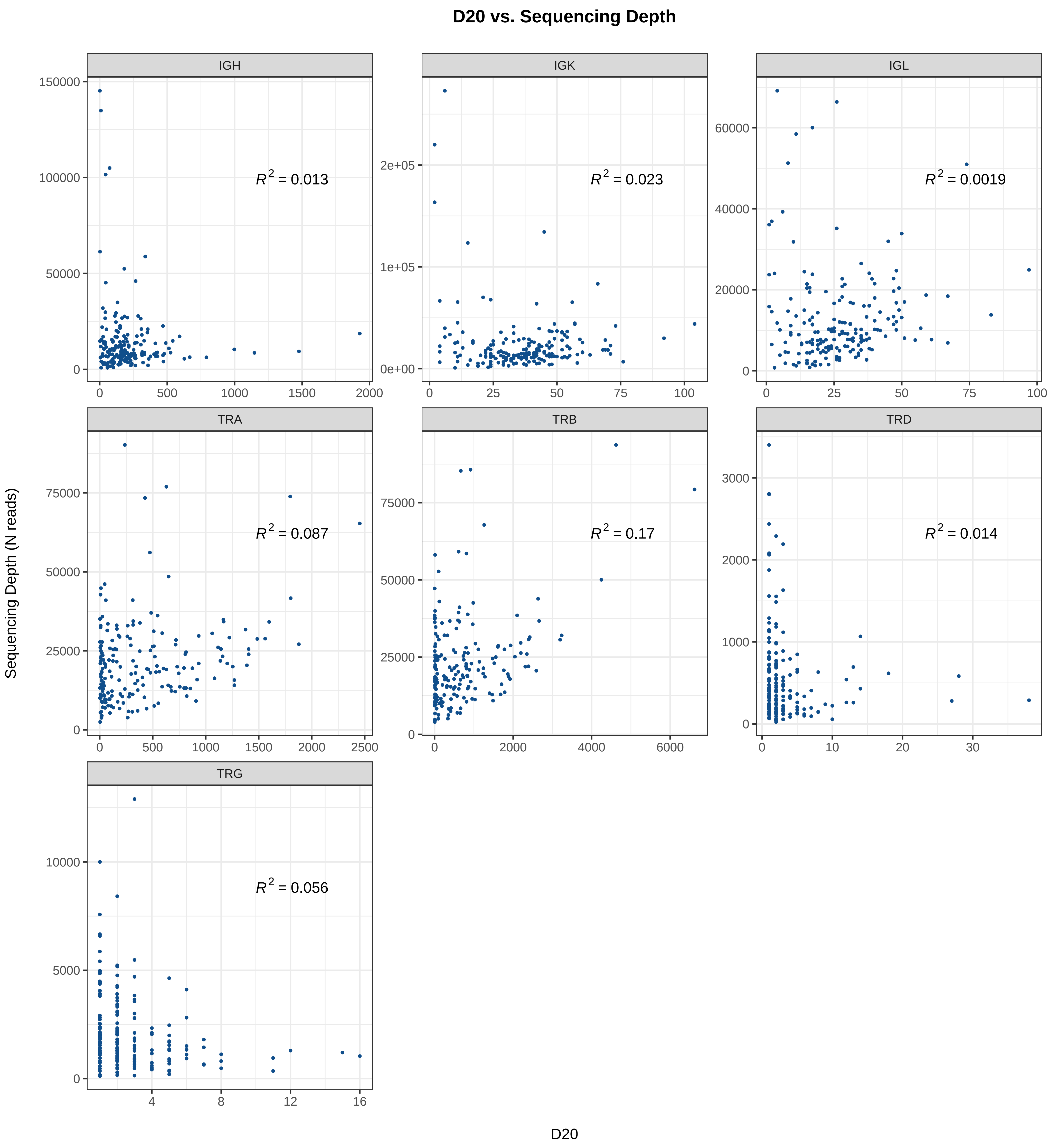
D20 vs. Sequencing Depth
IGH
IGK
IGL
TRA
TRB
TRD
TRG
$R^2 = 0.013$
$R^2 = 0.023$
$R^2 = 0.0019$
$R^2 = 0.087$
$R^2 = 0.17$
$R^2 = 0.014$
$R^2 = 0.056$
Sequencing Depth (N reads)
D20

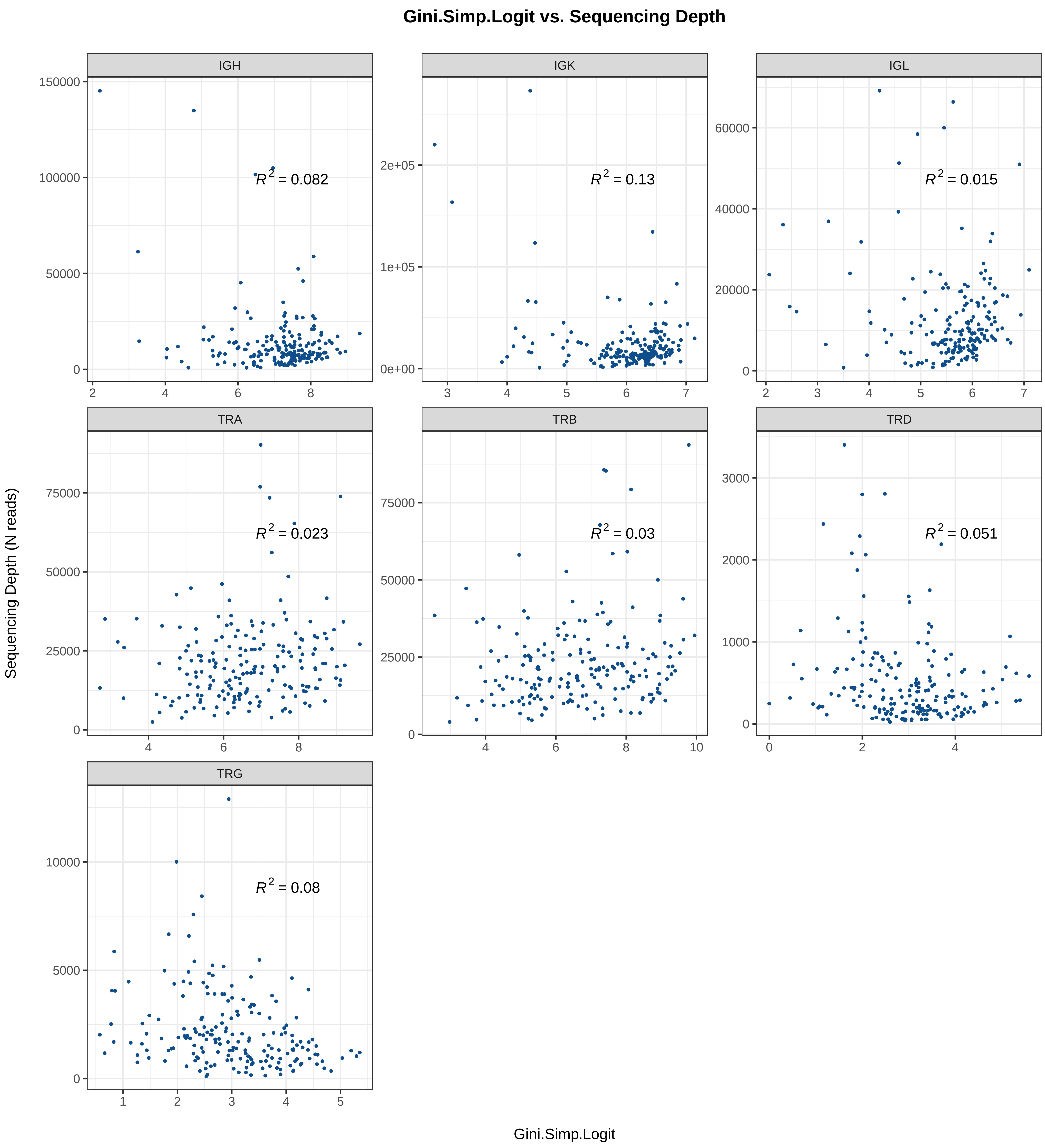

Gini.Simp.Logit vs. Sequencing Depth
IGH
IGK
IGL
TRA
TRB
TRD
TRG
$R^2 = 0.082$
$R^2 = 0.13$
$R^2 = 0.015$
$R^2 = 0.023$
$R^2 = 0.03$
$R^2 = 0.051$
$R^2 = 0.08$
Sequencing Depth (N reads)
Gini.Simp.Logit

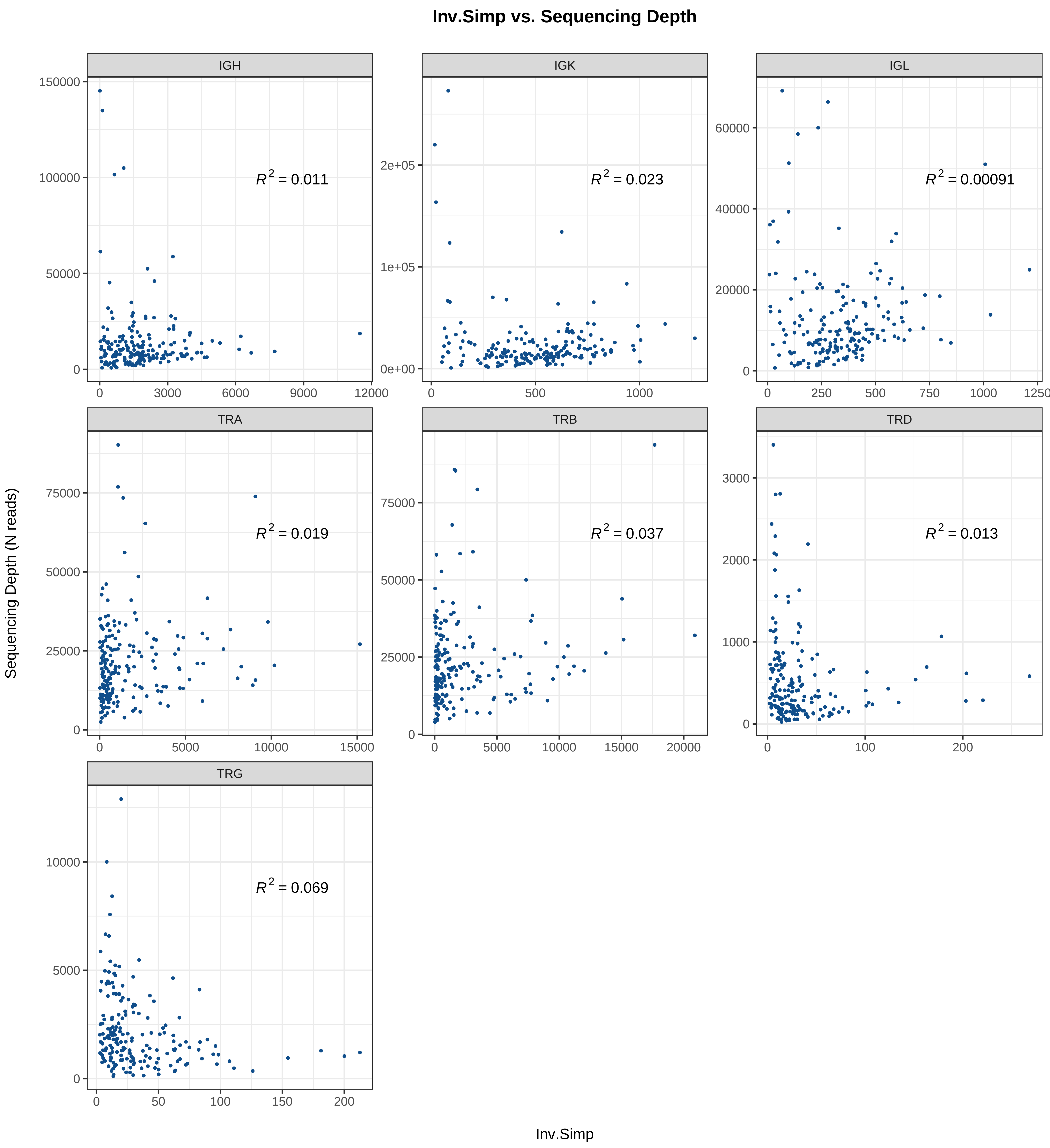
Inv.Simp vs. Sequencing Depth
IGH
$R^2 = 0.011$
IGK
$R^2 = 0.023$
IGL
$R^2 = 0.00091$
TRA
$R^2 = 0.019$
TRB
$R^2 = 0.037$
TRD
$R^2 = 0.013$
TRG
$R^2 = 0.069$
Sequencing Depth (N reads)
Inv.Simp

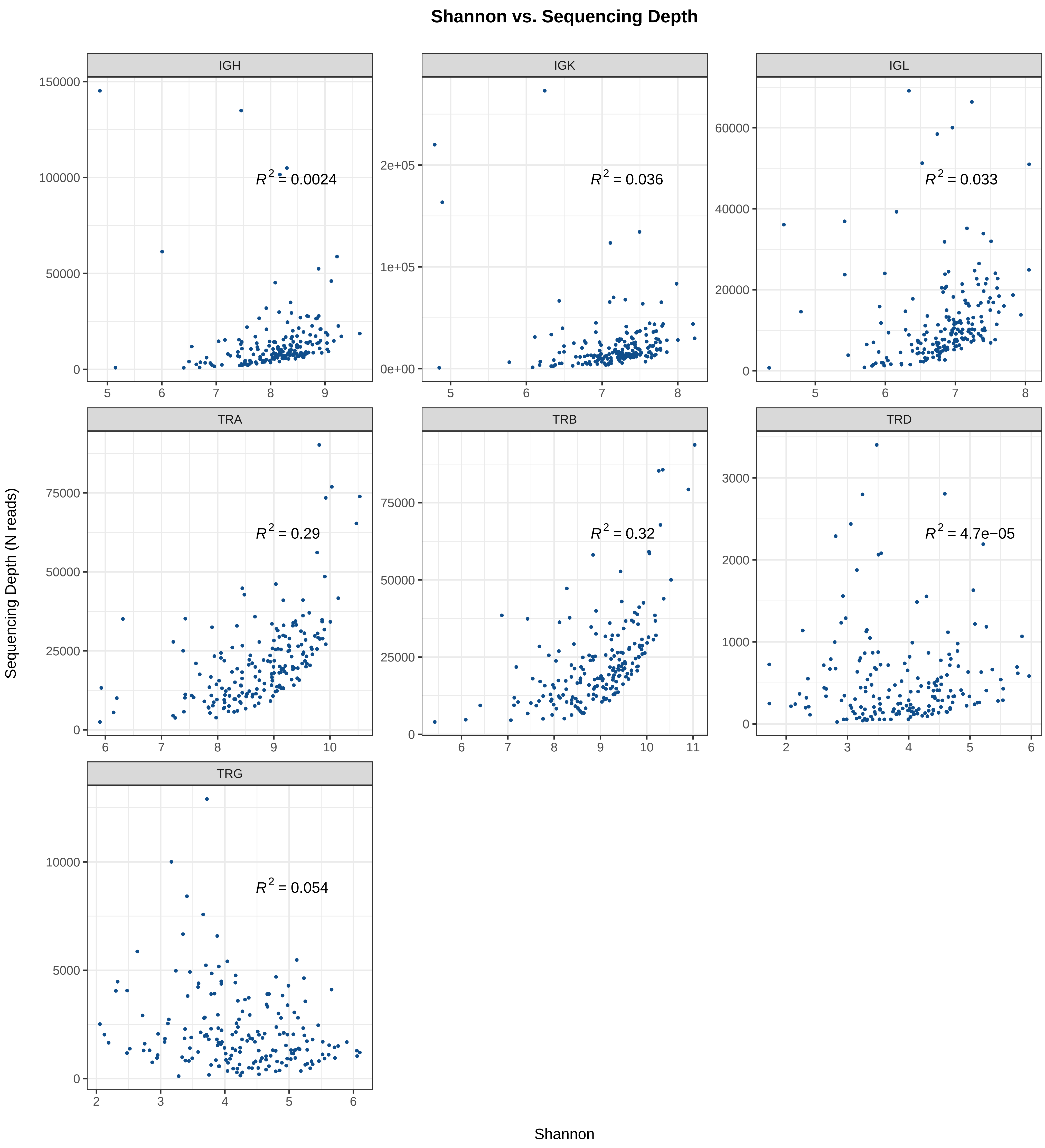
Shannon vs. Sequencing Depth
IGH
IGK
IGL
TRA
TRB
TRD
TRG
R² = 0.0024
R² = 0.036
R² = 0.033
R² = 0.29
R² = 0.32
R² = 4.7e−05
R² = 0.054
Sequencing Depth (N reads)
Shannon

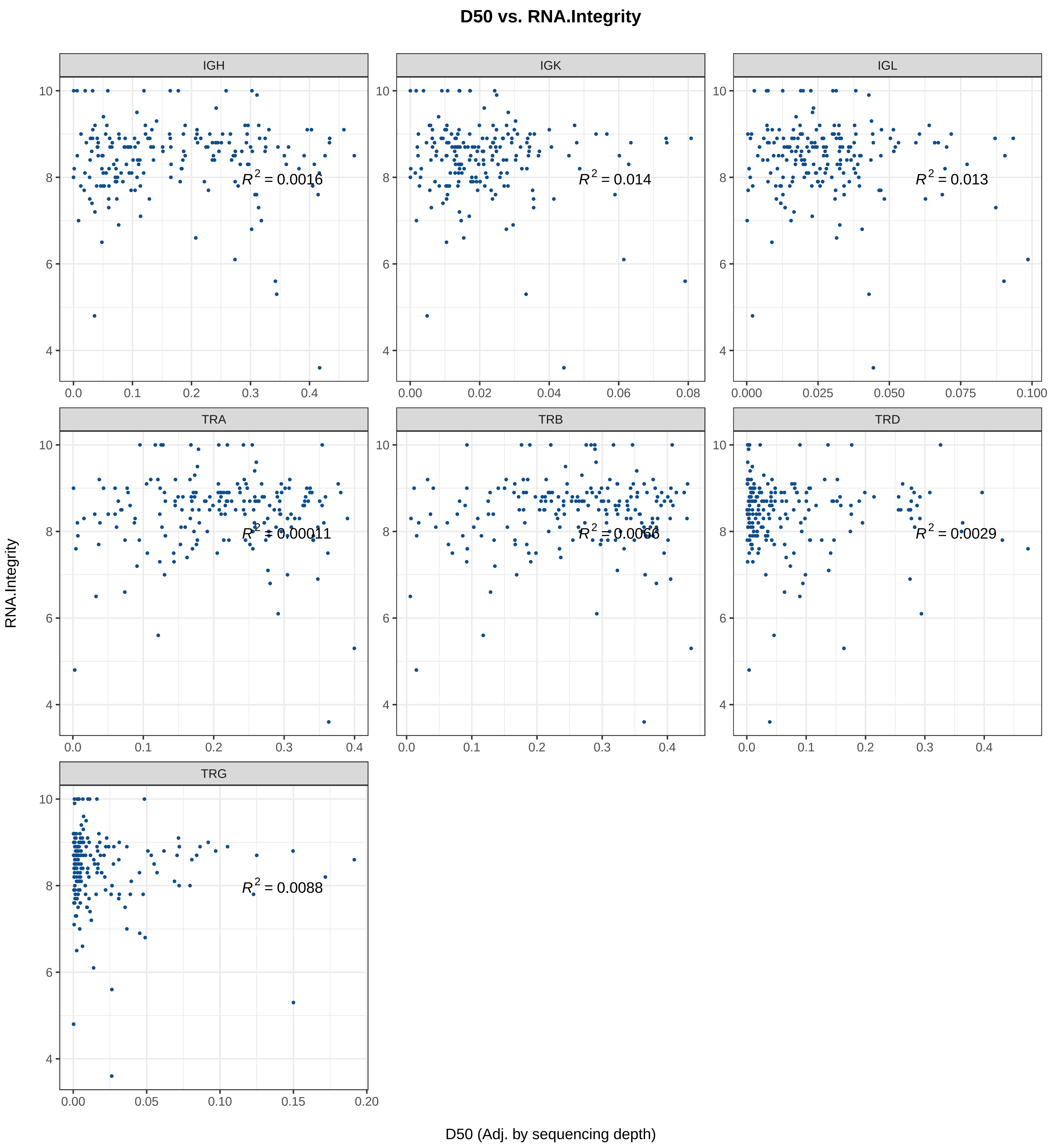
D50 vs. RNA.Integrity
IGH
IGK
IGL
TRA
TRB
TRD
TRG
R2 = 0.0016
R2 = 0.014
R2 = 0.013
R2 = 0.00011
R2 = 0.0066
R2 = 0.0029
R2 = 0.0088
RNA.Integrity
D50 (Adj. by sequencing depth)

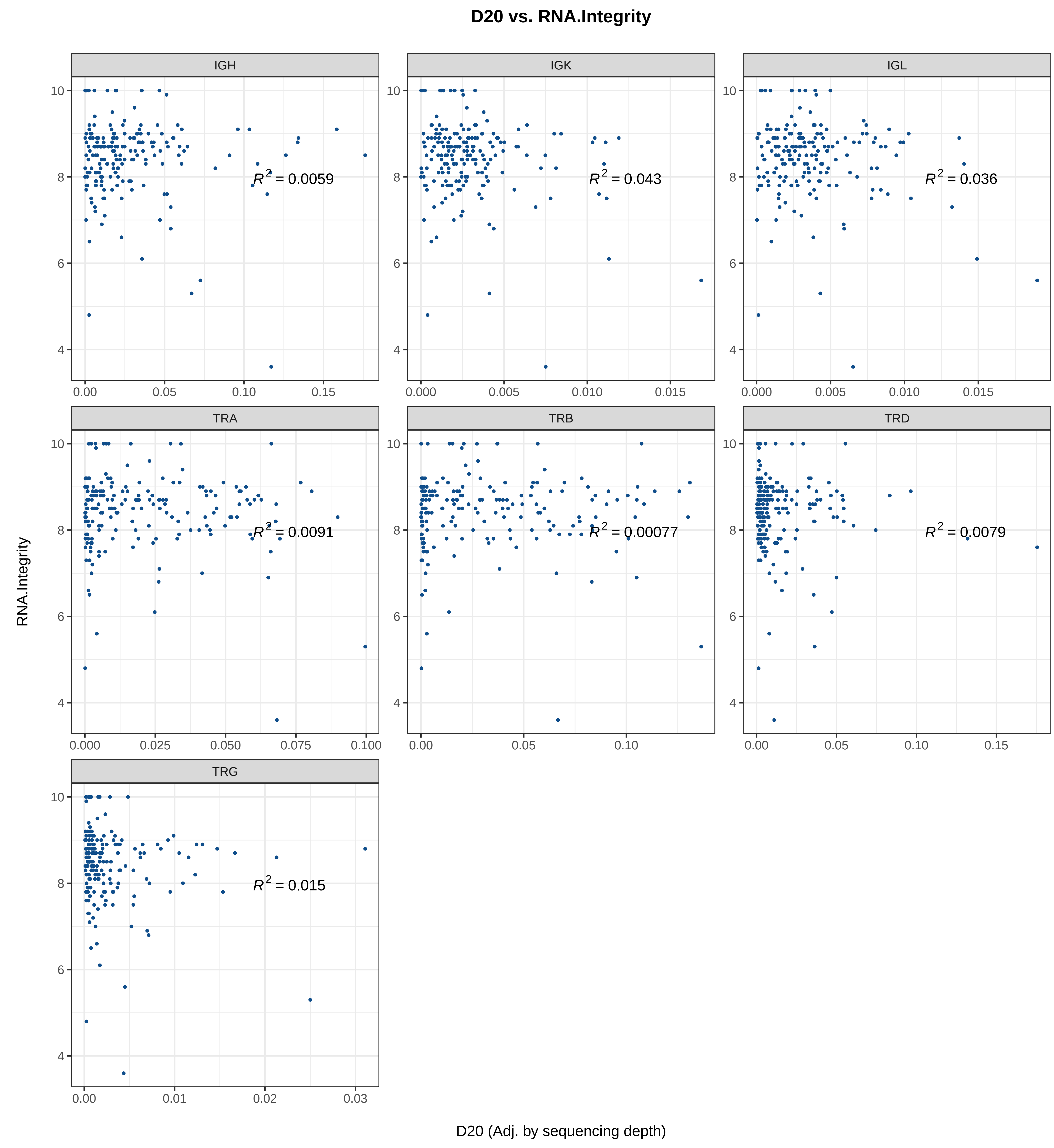

D20 vs. RNA.Integrity
IGH
IGK
IGL
TRA
TRB
TRD
TRG
R^2 = 0.0059
R^2 = 0.043
R^2 = 0.036
R^2 = 0.0091
R^2 = 0.00077
R^2 = 0.0079
R^2 = 0.015
RNA.Integrity
D20 (Adj. by sequencing depth)

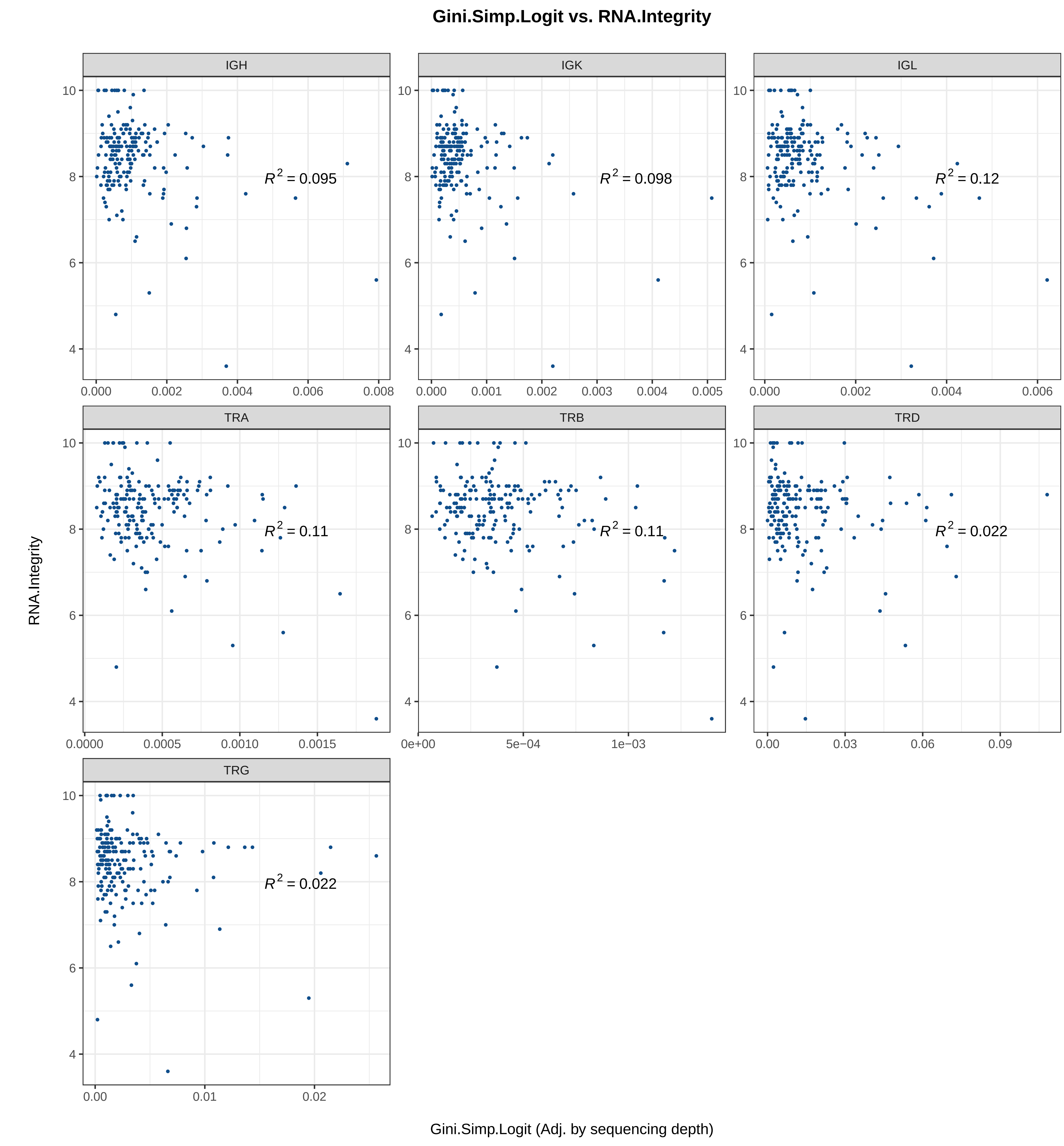
Gini.Simp.Logit vs. RNA.Integrity
IGH
IGK
IGL
TRA
TRB
TRD
TRG
$R^2 = 0.095$
$R^2 = 0.098$
$R^2 = 0.12$
$R^2 = 0.11$
$R^2 = 0.11$
$R^2 = 0.022$
$R^2 = 0.022$
RNA.Integrity
Gini.Simp.Logit (Adj. by sequencing depth)

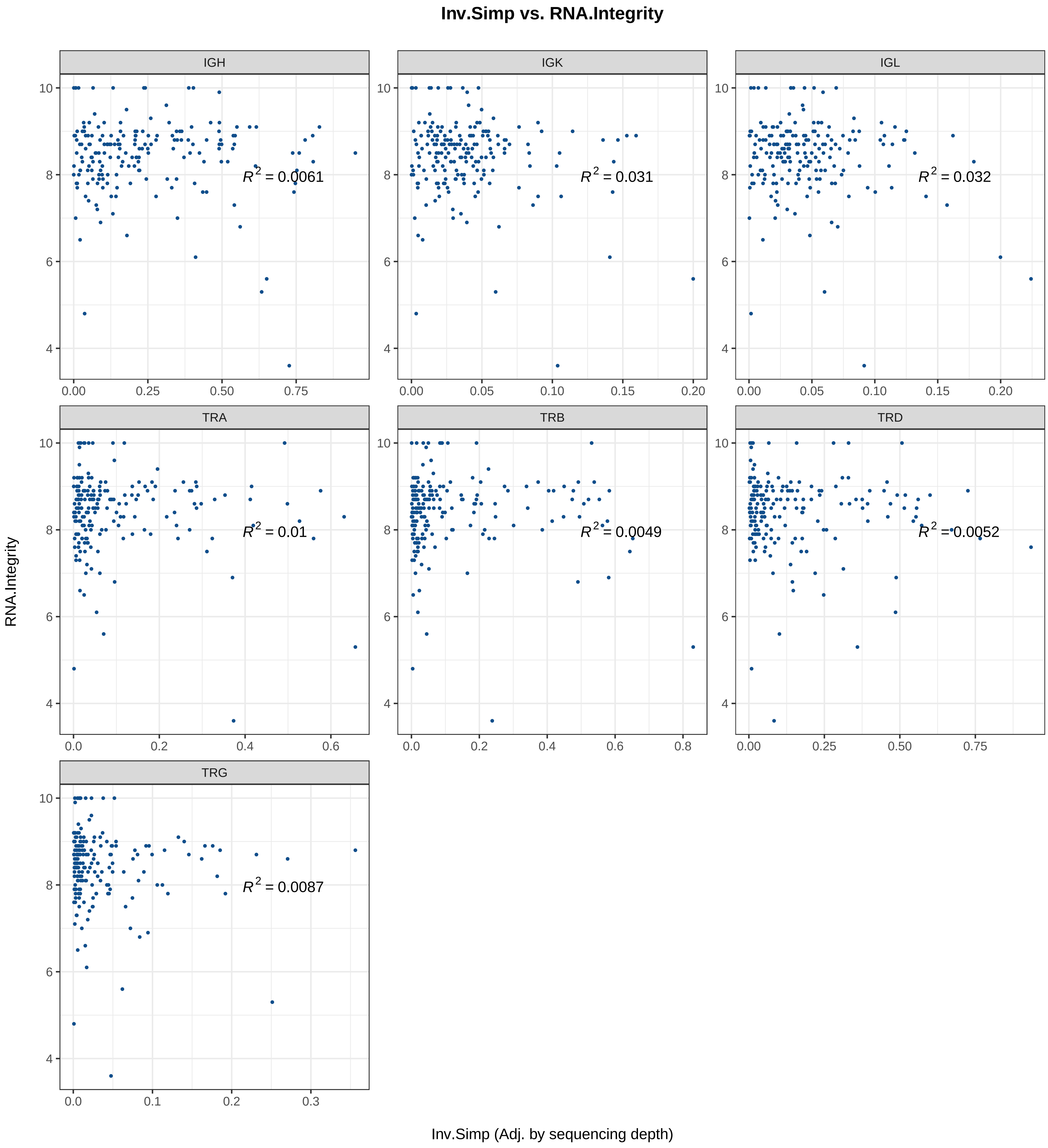

Inv.Simp vs. RNA.Integrity
IGH
IGK
IGL
TRA
TRB
TRD
TRG
$R^2 = 0.0061$
$R^2 = 0.031$
$R^2 = 0.032$
$R^2 = 0.01$
$R^2 = 0.0049$
$R^2 = 0.0052$
$R^2 = 0.0087$
RNA.Integrity
Inv.Simp (Adj. by sequencing depth)

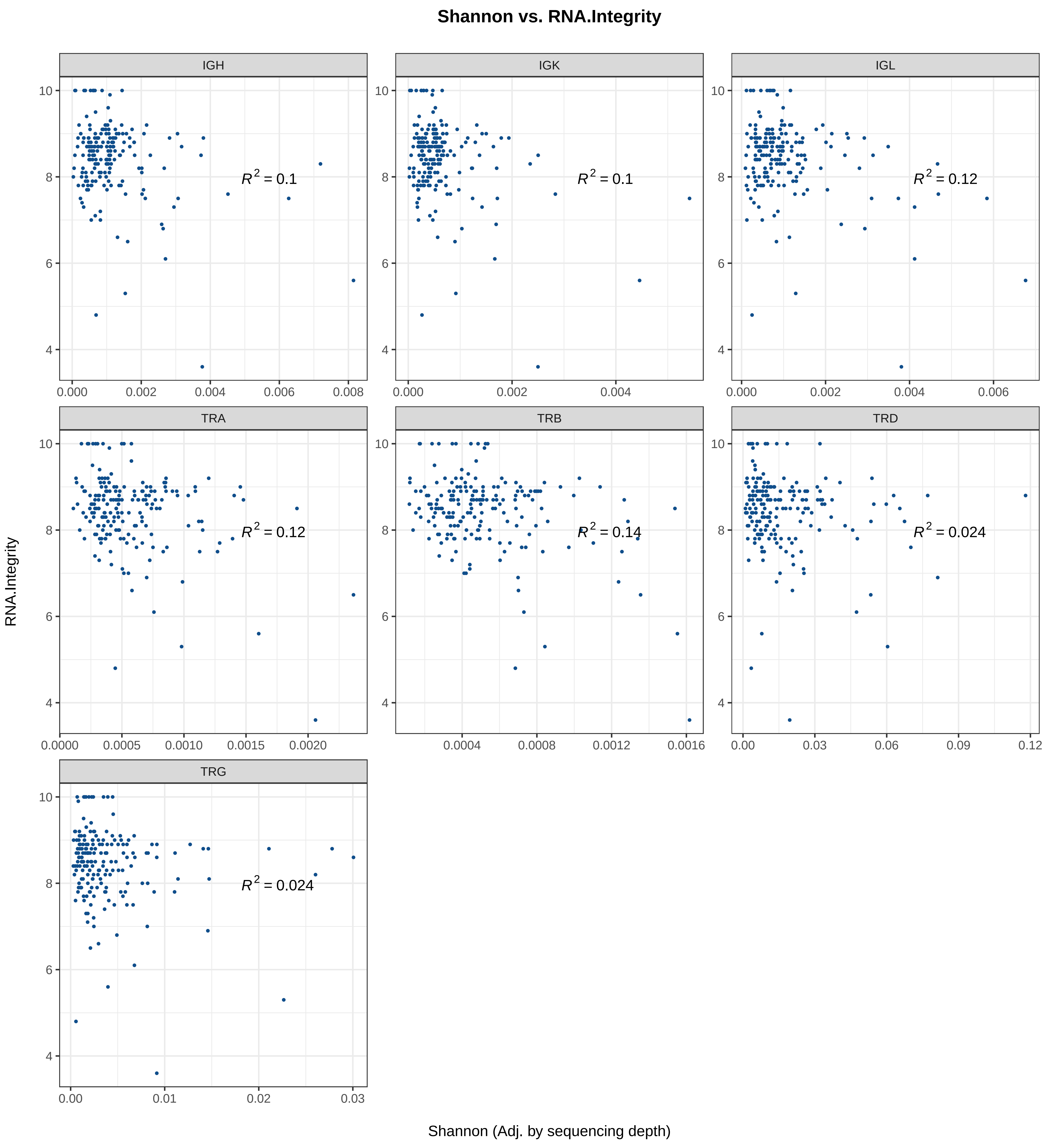

Shannon vs. RNA.Integrity
IGH
IGK
IGL
TRA
TRB
TRD
TRG
$R^2 = 0.1$
$R^2 = 0.1$
$R^2 = 0.12$
$R^2 = 0.12$
$R^2 = 0.14$
$R^2 = 0.024$
$R^2 = 0.024$
RNA.Integrity
Shannon (Adj. by sequencing depth)

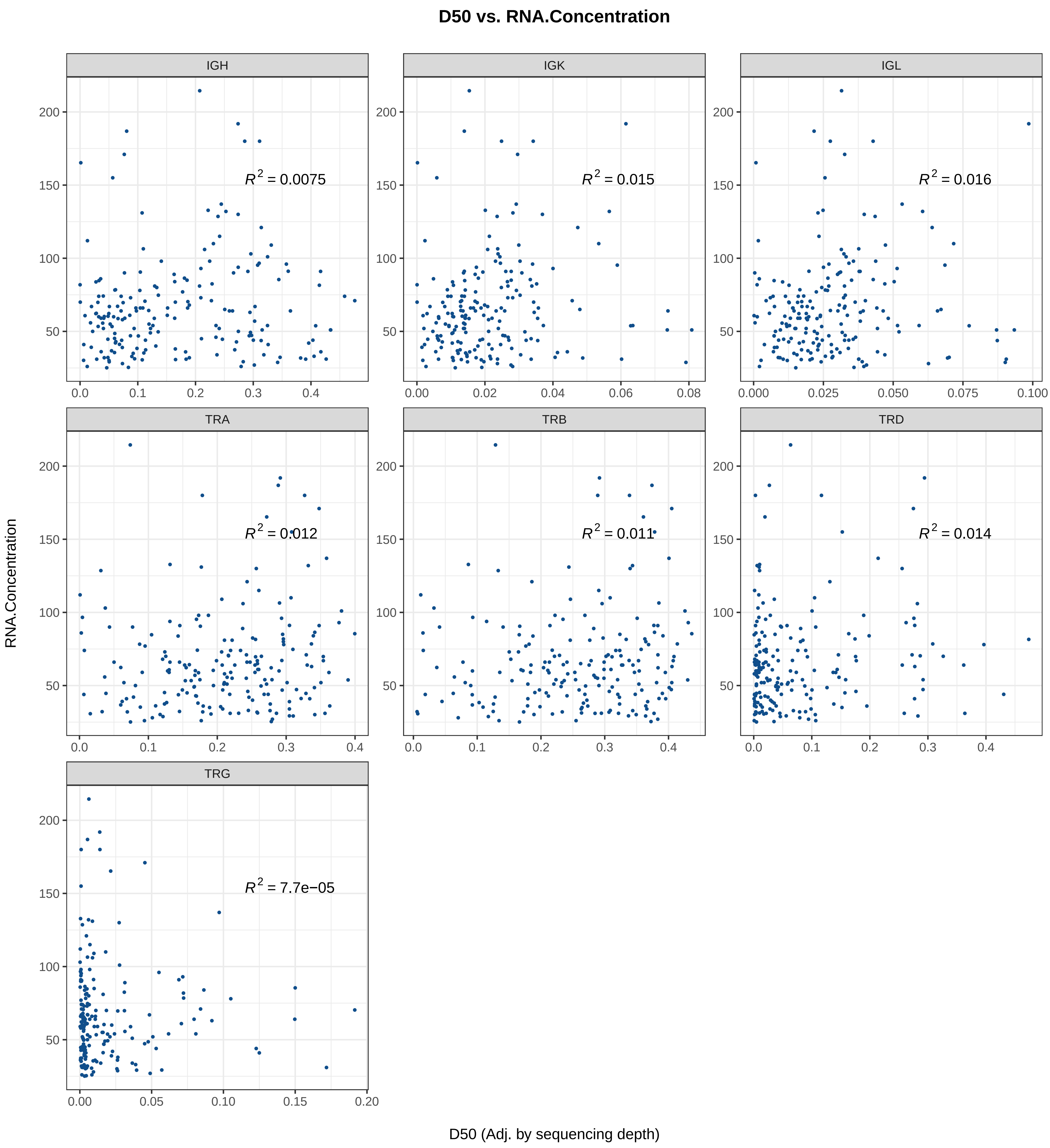
D50 vs. RNA.Concentration
IGH
IGK
IGL
TRA
TRB
TRD
TRG
R^2 = 0.0075
R^2 = 0.015
R^2 = 0.016
R^2 = 0.012
R^2 = 0.011
R^2 = 0.014
R^2 = 7.7e−05
RNA.Concentration
D50 (Adj. by sequencing depth)

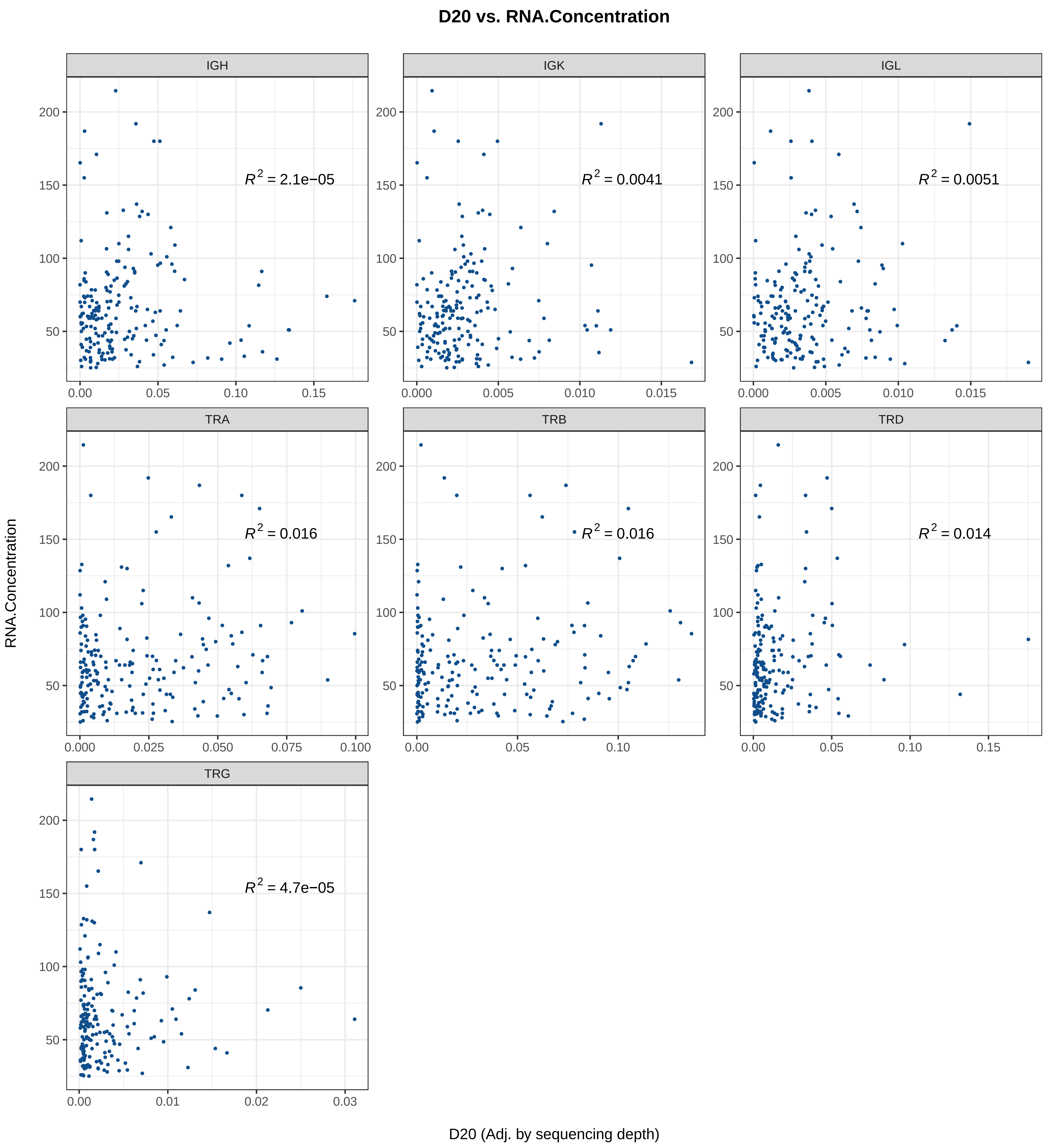
D20 vs. RNA.Concentration
IGH
IGK
IGL
TRA
TRB
TRD
TRG
$R^2 = 2.1e{-}05$
$R^2 = 0.0041$
$R^2 = 0.0051$
$R^2 = 0.016$
$R^2 = 0.016$
$R^2 = 0.014$
$R^2 = 4.7e{-}05$
RNA.Concentration
D20 (Adj. by sequencing depth)

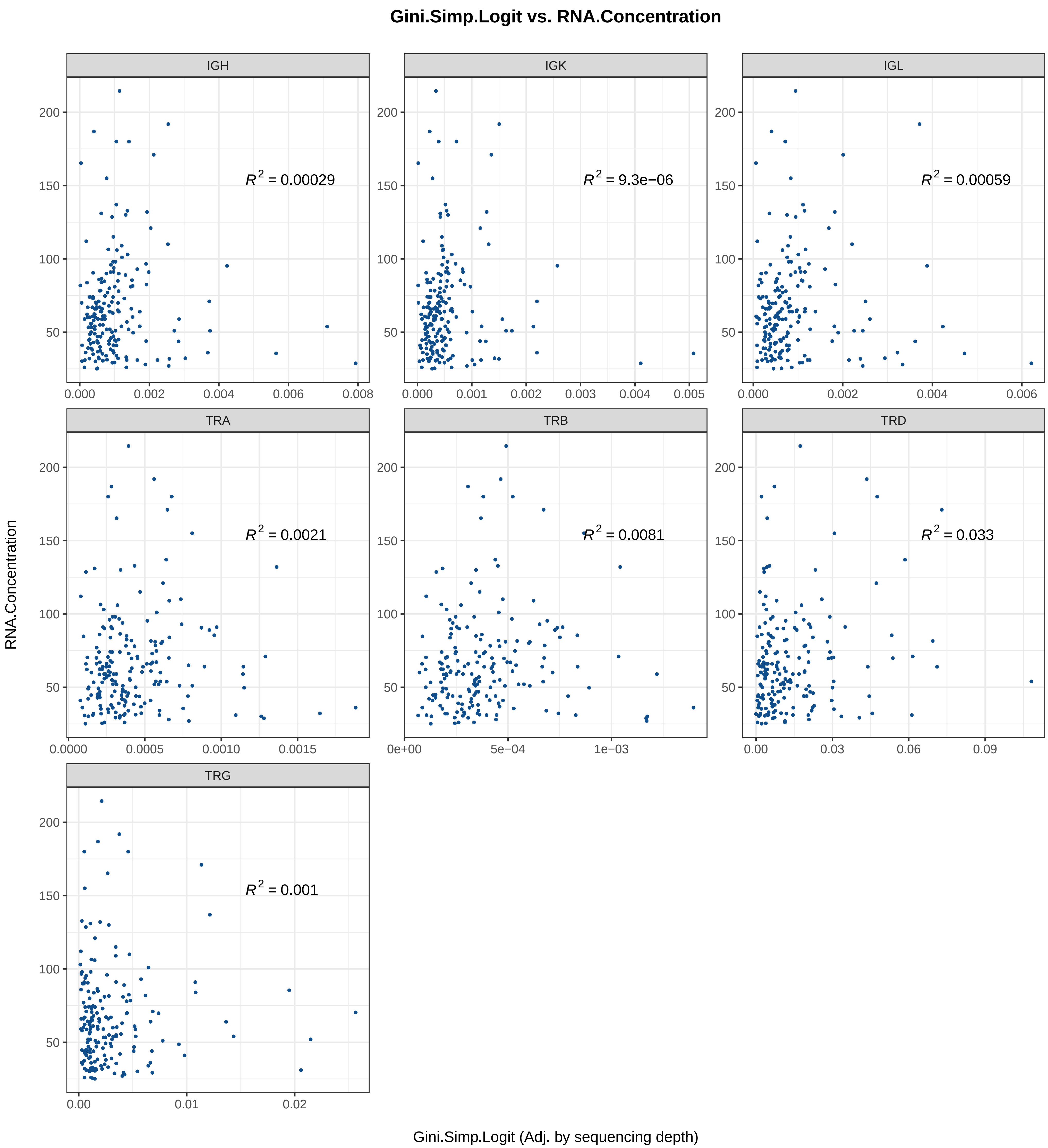
Gini.Simp.Logit vs. RNA.Concentration
IGH
IGK
IGL
TRA
TRB
TRD
TRG
R2 = 0.00029
R2 = 9.3e−06
R2 = 0.00059
R2 = 0.0021
R2 = 0.0081
R2 = 0.033
R2 = 0.001
RNA.Concentration
Gini.Simp.Logit (Adj. by sequencing depth)

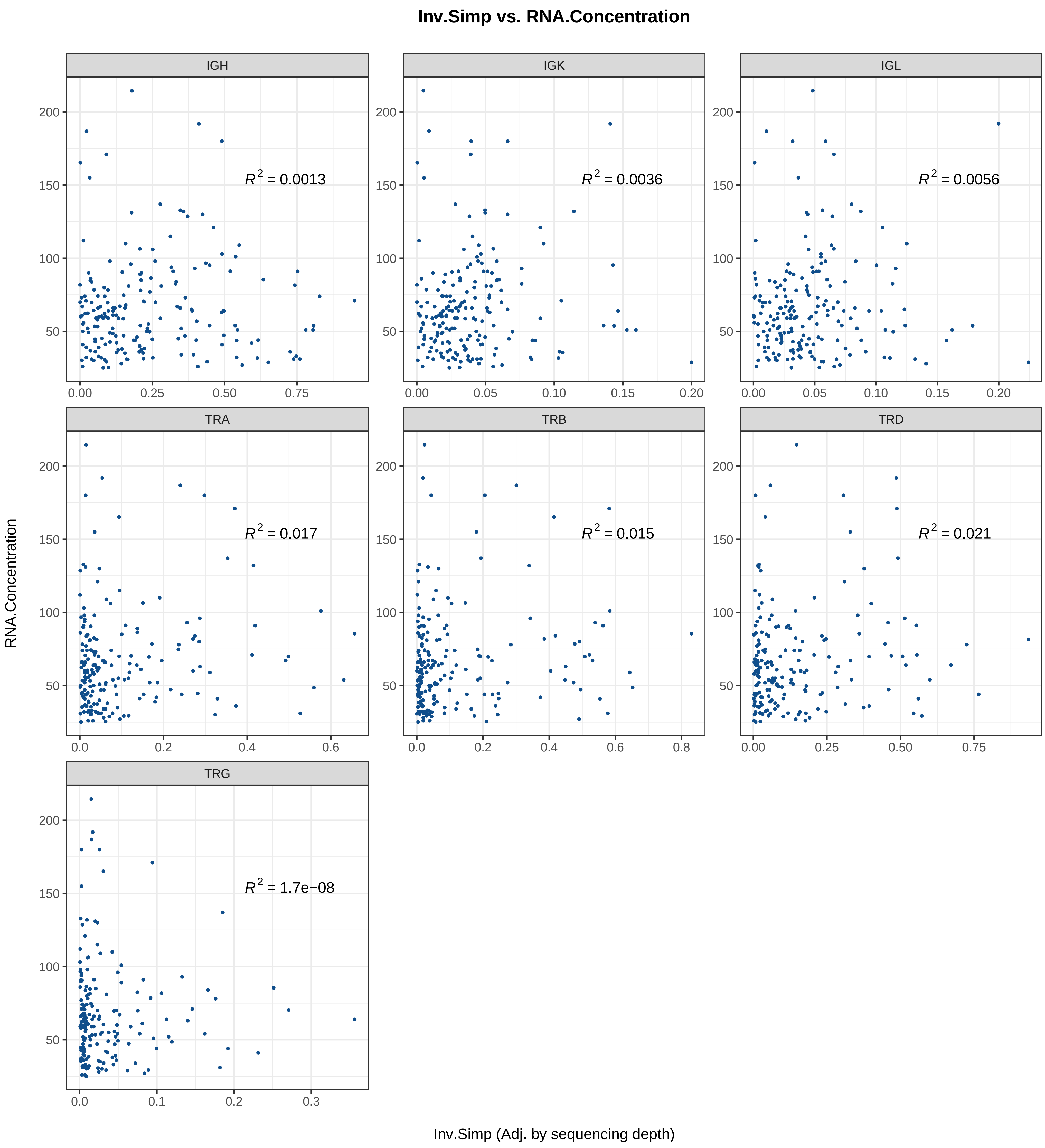
Inv.Simp vs. RNA.Concentration
IGH
IGK
IGL
TRA
TRB
TRD
TRG
$R^2 = 0.0013$
$R^2 = 0.0036$
$R^2 = 0.0056$
$R^2 = 0.017$
$R^2 = 0.015$
$R^2 = 0.021$
$R^2 = 1.7e{-}08$
RNA.Concentration
Inv.Simp (Adj. by sequencing depth)

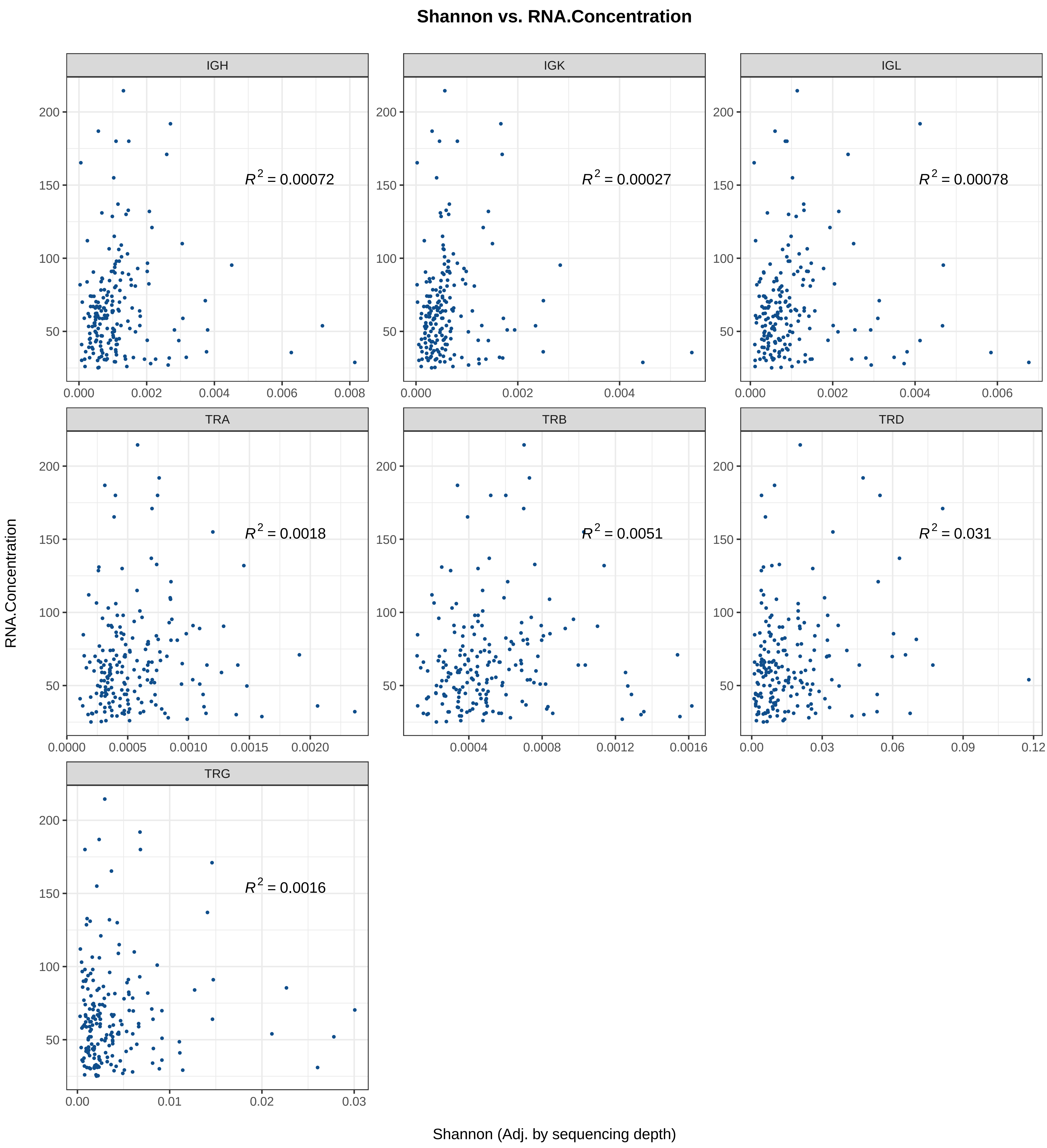

Shannon vs. RNA.Concentration
IGH
IGK
IGL
TRA
TRB
TRD
TRG
$R^2 = 0.00072$
$R^2 = 0.00027$
$R^2 = 0.00078$
$R^2 = 0.0018$
$R^2 = 0.0051$
$R^2 = 0.031$
$R^2 = 0.0016$
RNA.Concentration
Shannon (Adj. by sequencing depth)

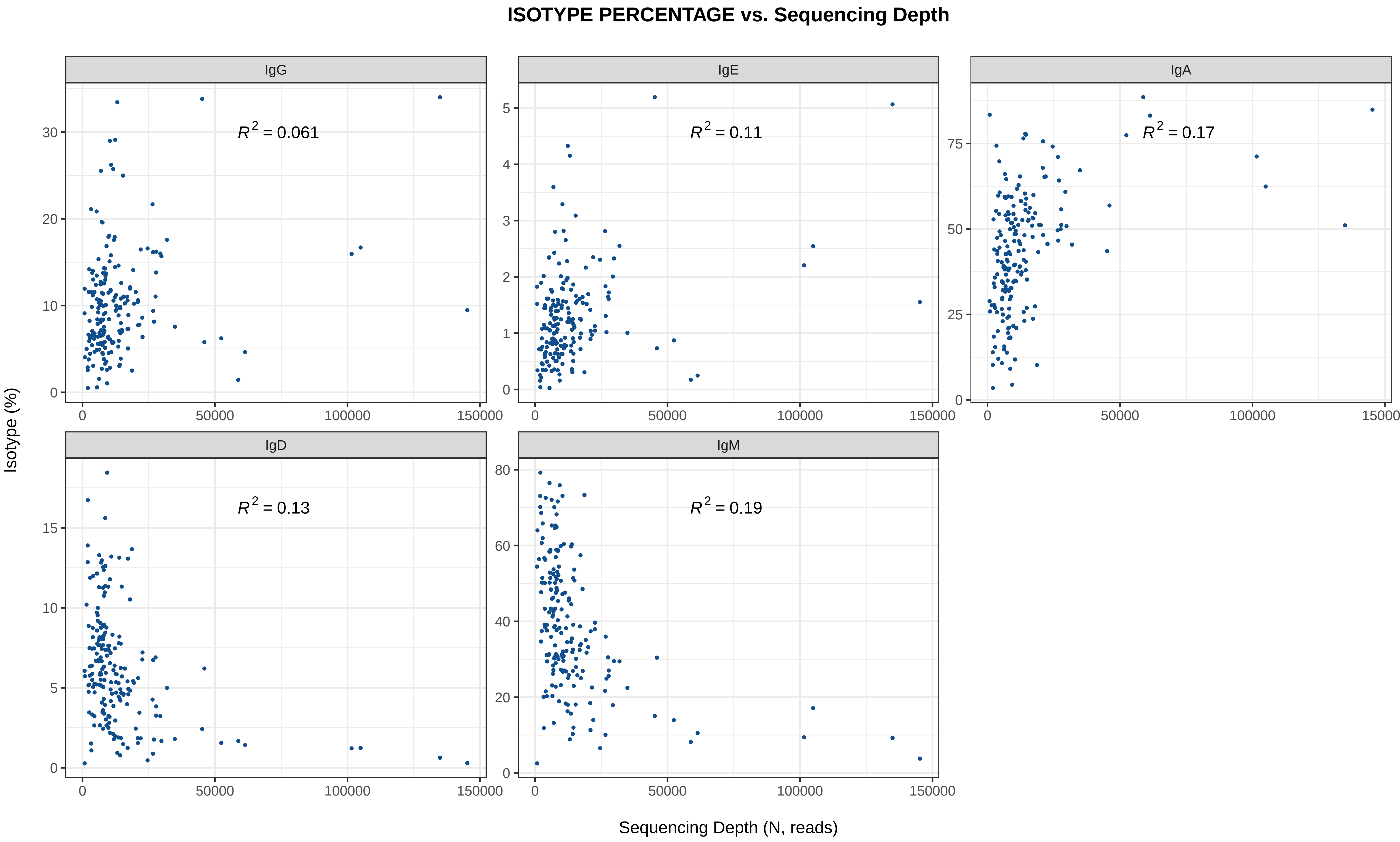
ISOTYPE PERCENTAGE vs. Sequencing Depth
IgG
IgE
IgA
IgD
IgM
$R^2 = 0.061$
$R^2 = 0.11$
$R^2 = 0.17$
$R^2 = 0.13$
$R^2 = 0.19$
Isotype (%)
Sequencing Depth (N, reads)
0
50000
100000
150000

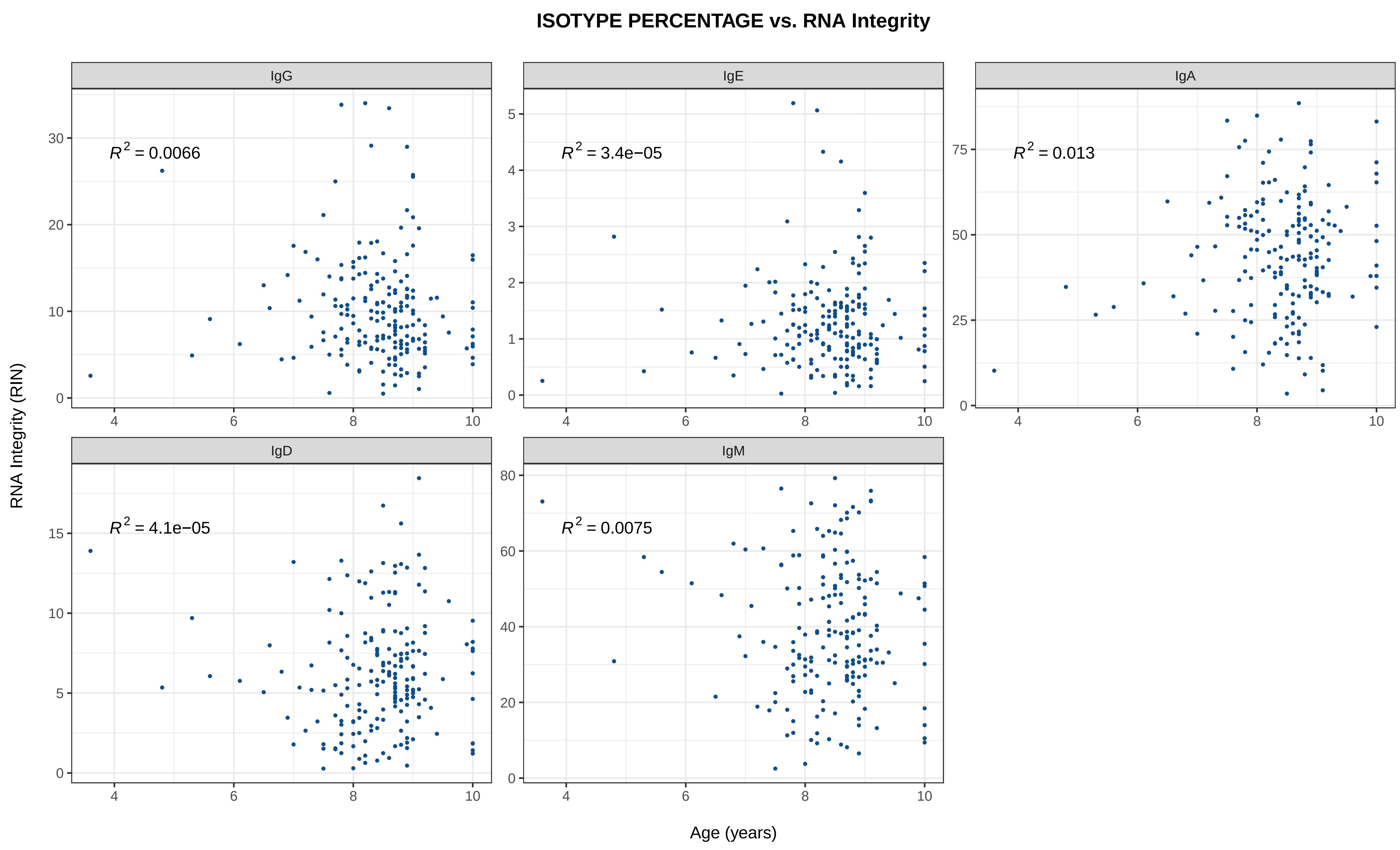
ISOTYPE PERCENTAGE vs. RNA Integrity
IgG
IgE
IgA
IgD
IgM
R2 = 0.0066
R2 = 3.4e−05
R2 = 0.013
R2 = 4.1e−05
R2 = 0.0075
RNA Integrity (RIN)
Age (years)

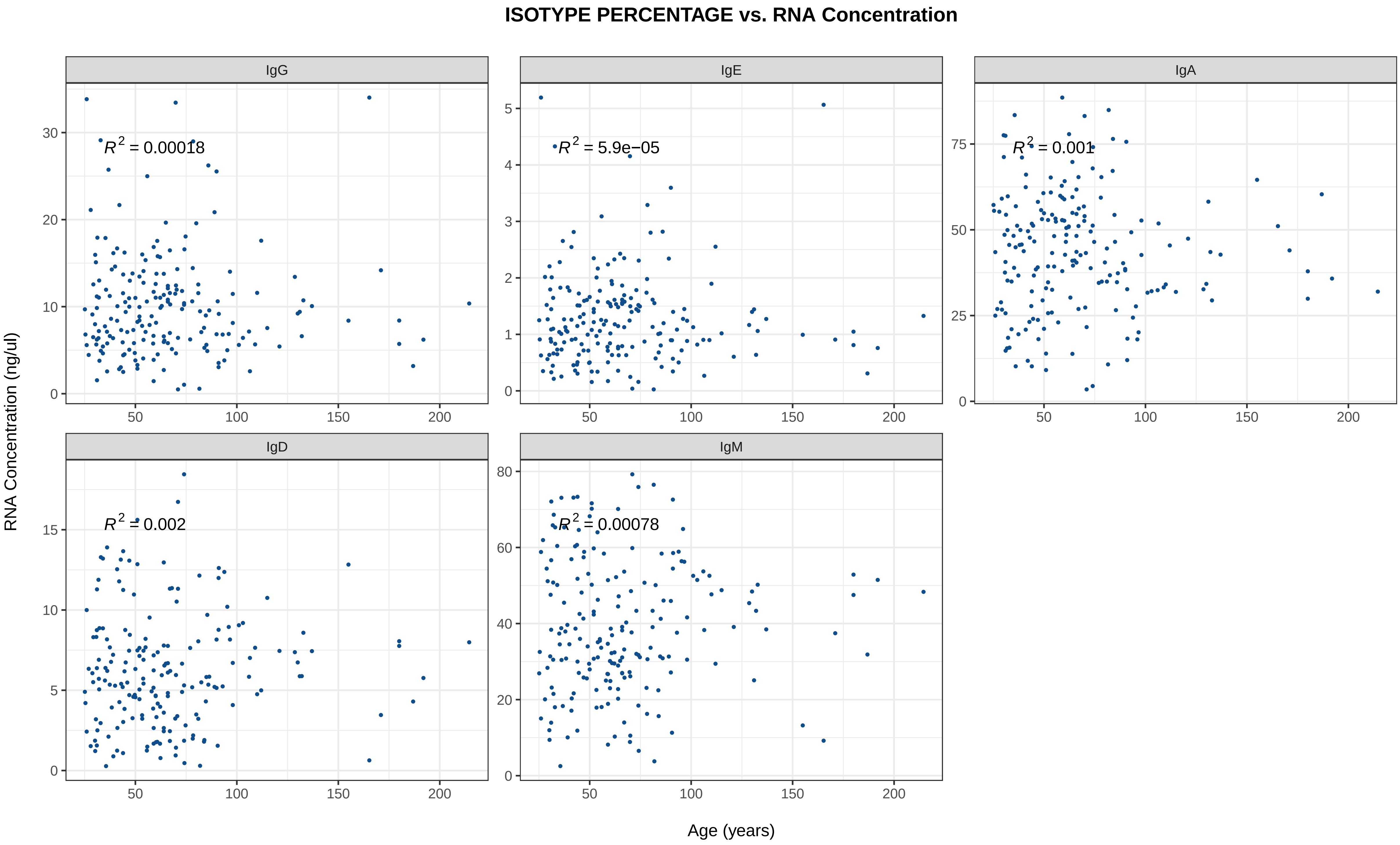

ISOTYPE PERCENTAGE vs. RNA Concentration
IgG
R^2 = 0.00018
IgE
R^2 = 5.9e−05
IgA
R^2 = 0.001
IgD
R^2 = 0.002
IgM
R^2 = 0.00078
RNA Concentration (ng/ul)
Age (years)
0
10
20
30
0
1
2
3
4
5
0
25
50
75
0
5
10
15
0
20
40
60
80
50
100
150
200

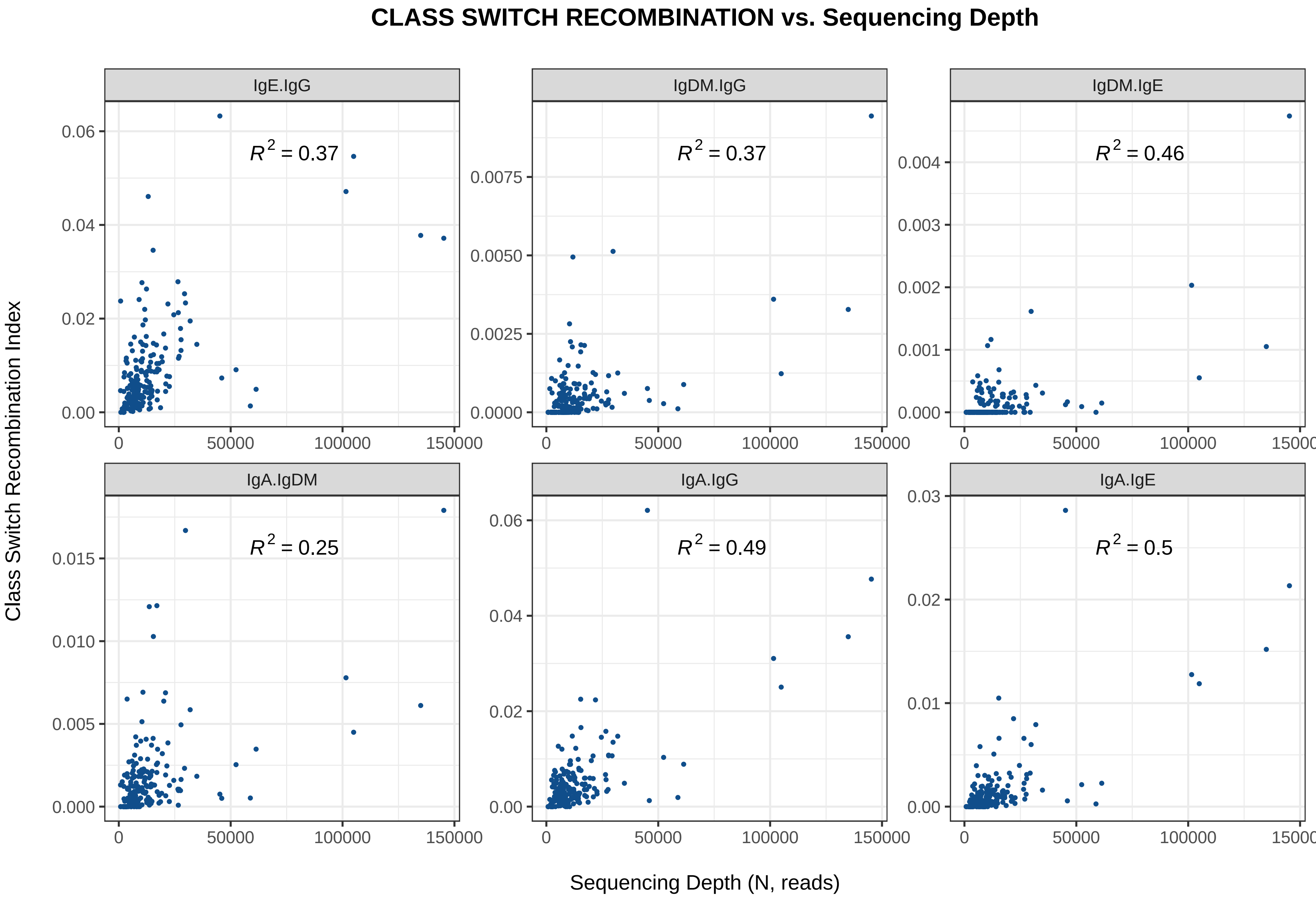

CLASS SWITCH RECOMBINATION vs. Sequencing Depth
IgE.IgG
$R^2 = 0.37$
IgDM.IgG
$R^2 = 0.37$
IgDM.IgE
$R^2 = 0.46$
IgA.IgDM
$R^2 = 0.25$
IgA.IgG
$R^2 = 0.49$
IgA.IgE
$R^2 = 0.5$
Class Switch Recombination Index
Sequencing Depth (N, reads)
0
50000
100000
150000
0.00
0.02
0.04
0.06
0.0000
0.0025
0.0050
0.0075
0.000
0.001
0.002
0.003
0.004
0.005
0.010
0.015
0.01
0.03

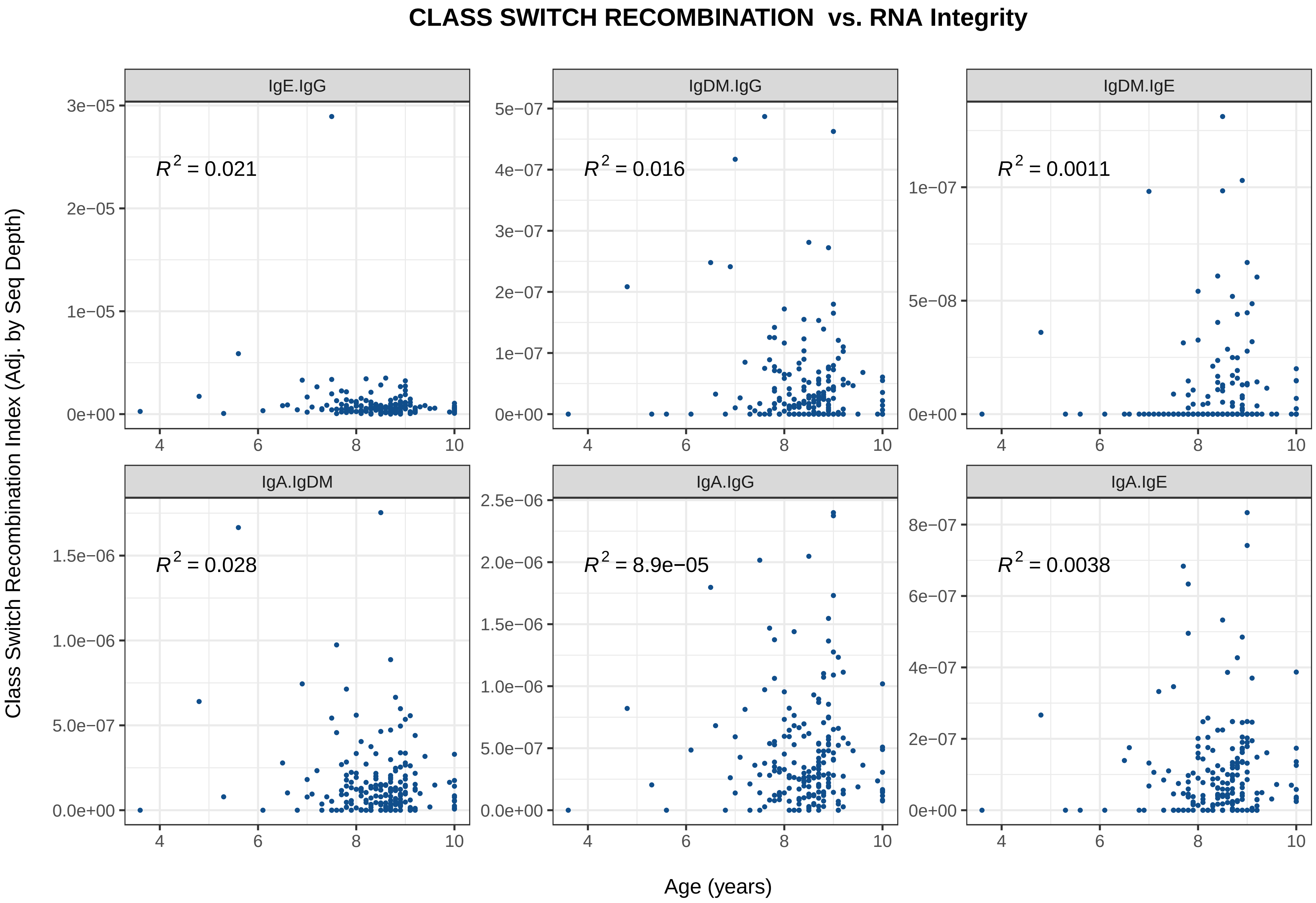

CLASS SWITCH RECOMBINATION vs. RNA Integrity
IgE.IgG
R² = 0.021
IgDM.IgG
R² = 0.016
IgDM.IgE
R² = 0.0011
IgA.IgDM
R² = 0.028
IgA.IgG
R² = 8.9e−05
IgA.IgE
R² = 0.0038
Class Switch Recombination Index (Adj. by Seq Depth)
Age (years)

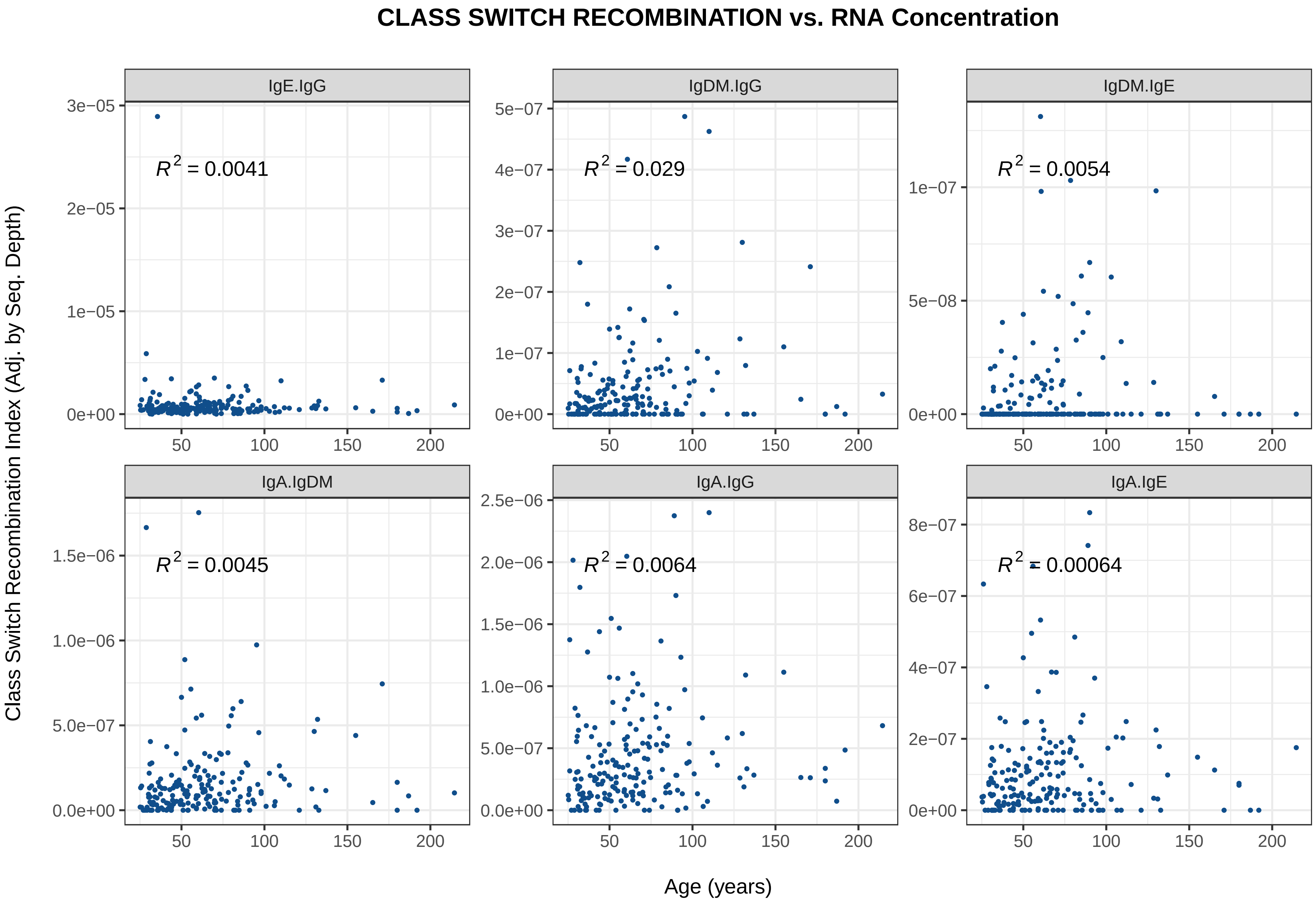

CLASS SWITCH RECOMBINATION vs. RNA Concentration
IgE.IgG
R^2 = 0.0041
IgDM.IgG
R^2 = 0.029
IgDM.IgE
R^2 = 0.0054
IgA.IgDM
R^2 = 0.0045
IgA.IgG
R^2 = 0.0064
IgA.IgE
R^2 = 0.00064
Class Switch Recombination Index (Adj. by Seq. Depth)
Age (years)

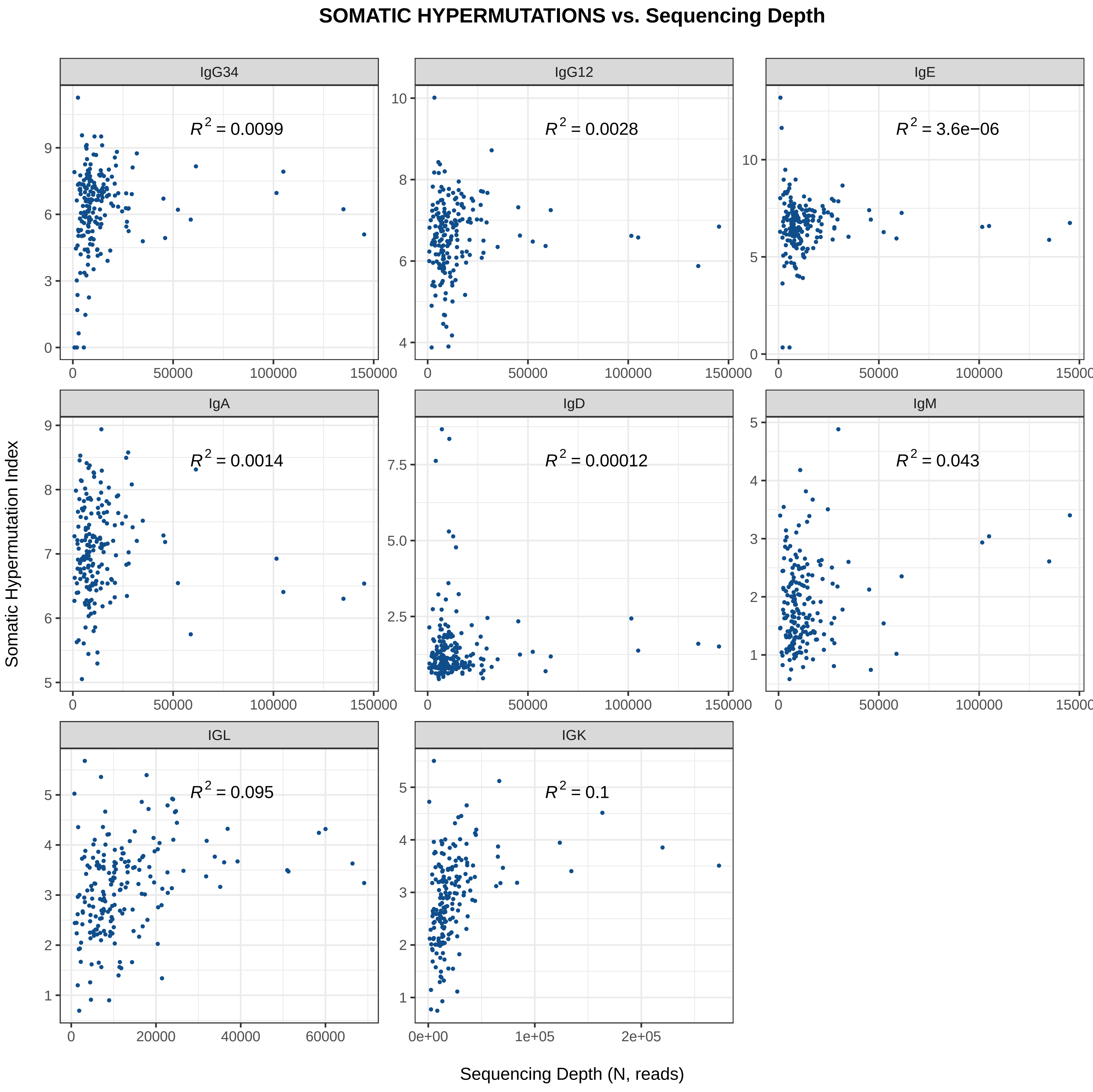
SOMATIC HYPERMUTATIONS vs. Sequencing Depth
IgG34
R^2 = 0.0099
IgG12
R^2 = 0.0028
IgE
R^2 = 3.6e−06
IgA
R^2 = 0.0014
IgD
R^2 = 0.00012
IgM
R^2 = 0.043
IGL
R^2 = 0.095
IGK
R^2 = 0.1
Somatic Hypermutation Index
Sequencing Depth (N, reads)

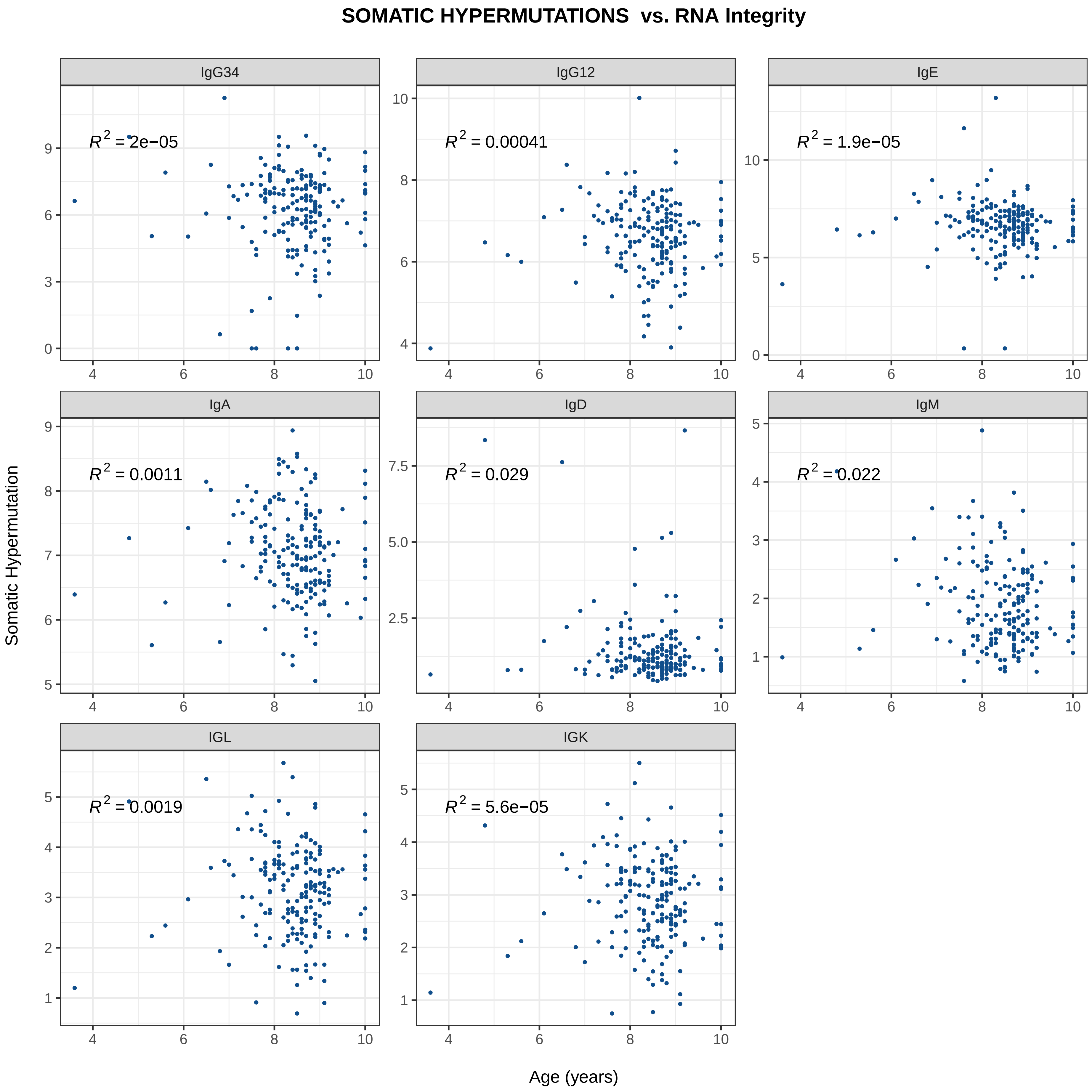

SOMATIC HYPERMUTATIONS vs. RNA Integrity
IgG34
R^2 = 2e−05
IgG12
R^2 = 0.00041
IgE
R^2 = 1.9e−05
IgA
R^2 = 0.0011
IgD
R^2 = 0.029
IgM
R^2 = 0.022
IGL
R^2 = 0.0019
IGK
R^2 = 5.6e−05
Somatic Hypermutation
Age (years)

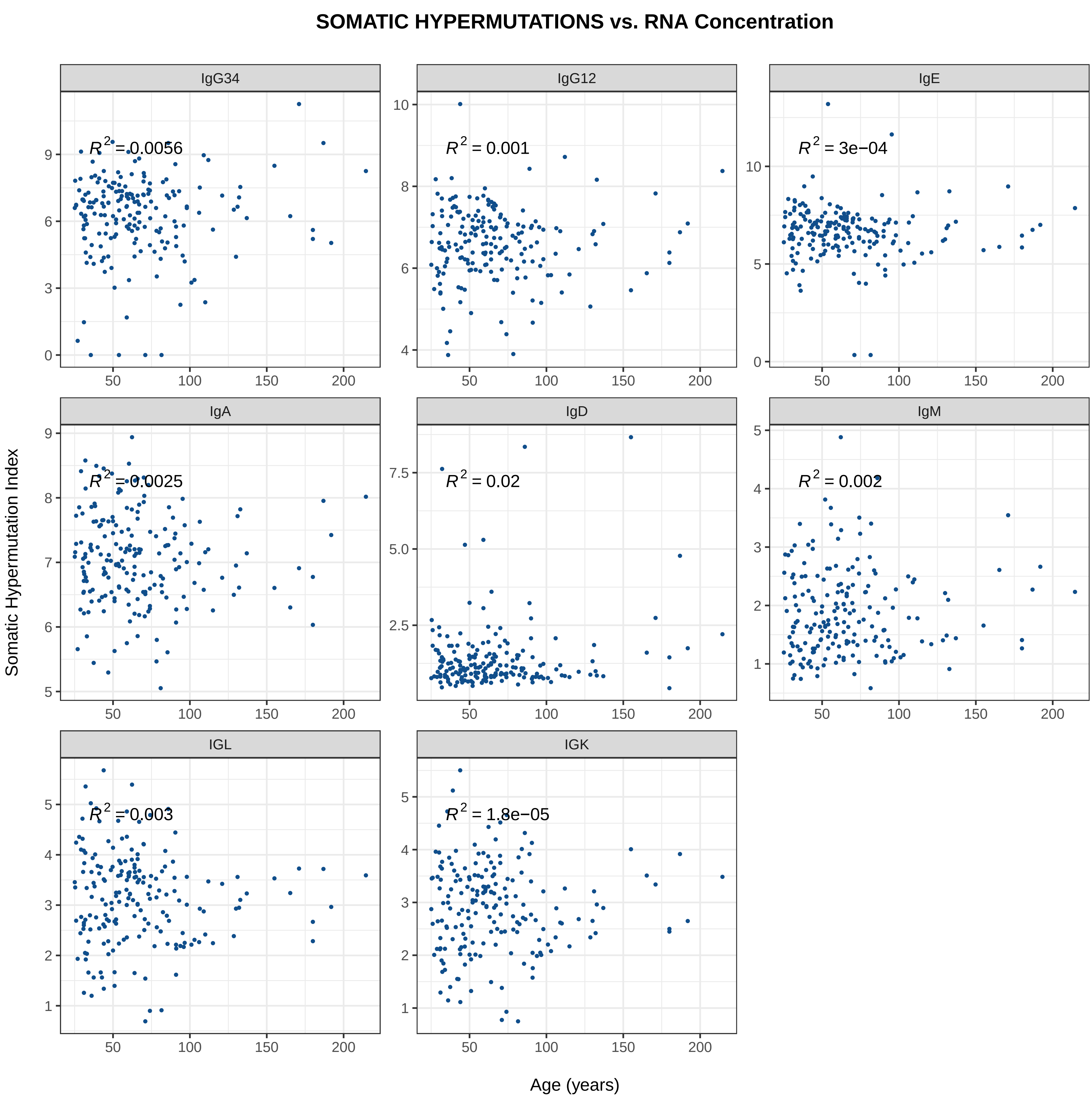

SOMATIC HYPERMUTATIONS vs. RNA Concentration
IgG34
$R^2 = 0.0056$
IgG12
$R^2 = 0.001$
IgE
$R^2 = 3e-04$
IgA
$R^2 = 0.0025$
IgD
$R^2 = 0.02$
IgM
$R^2 = 0.002$
IGL
$R^2 = 0.003$
IGK
$R^2 = 1.8e-05$
Somatic Hypermutation Index
Age (years)

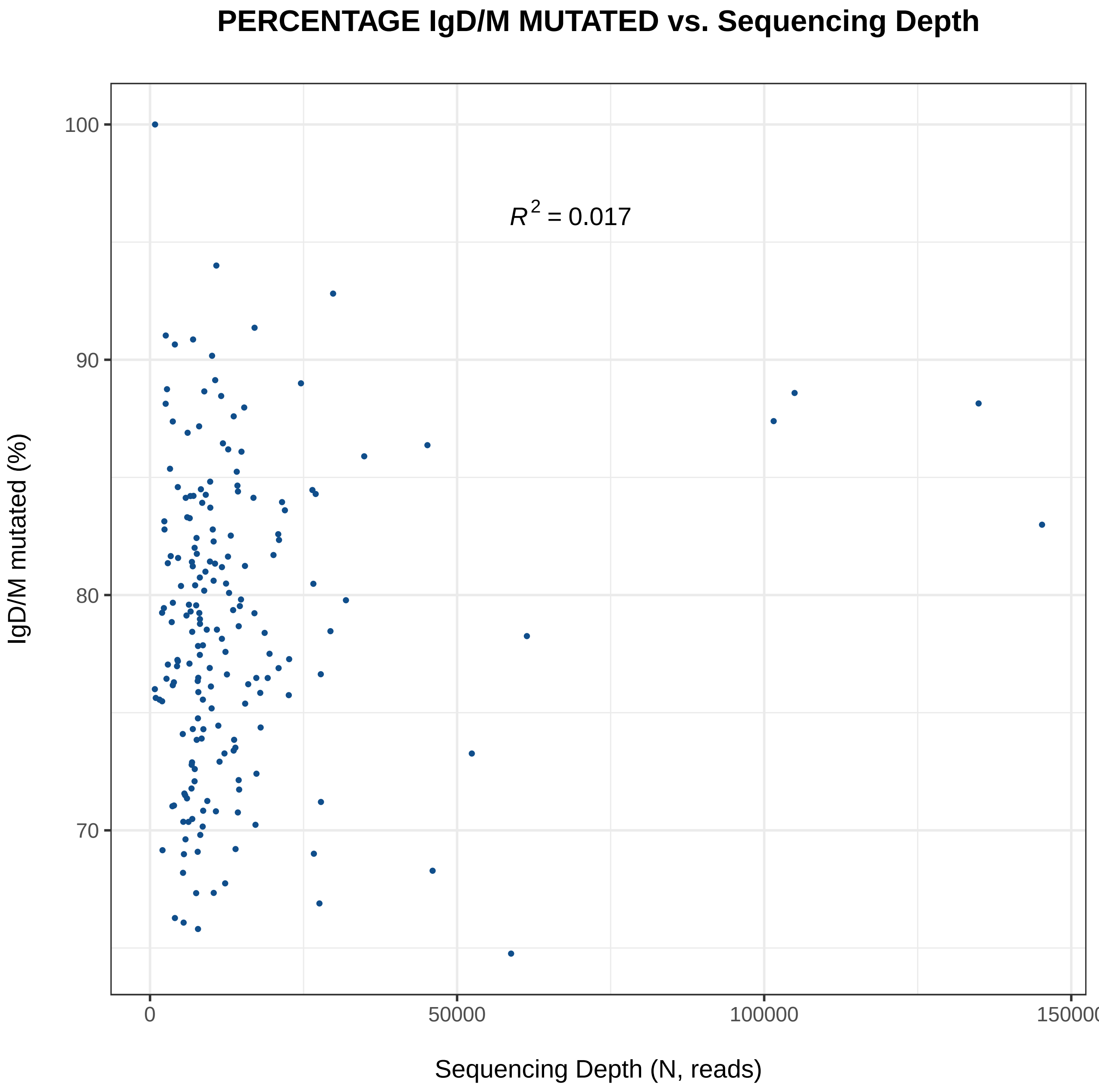
PERCENTAGE IgD/M MUTATED vs. Sequencing Depth
R^2 = 0.017
IgD/M mutated (%)
100
90
80
70
0
50000
100000
150000
Sequencing Depth (N, reads)

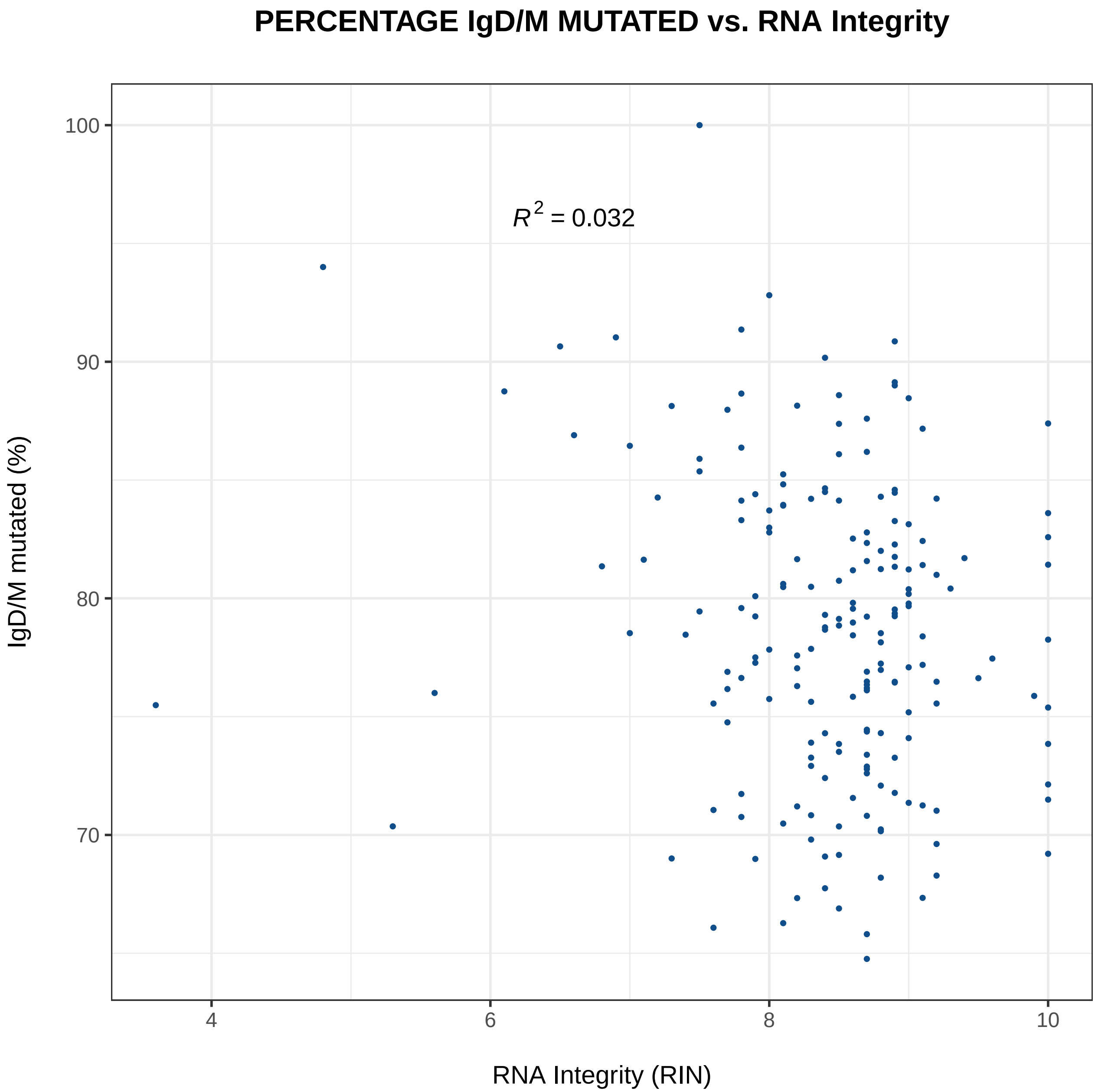

PERCENTAGE IgD/M MUTATED vs. RNA Integrity
$R^2 = 0.032$
IgD/M mutated (%)
100
90
80
70
4
6
8
10
RNA Integrity (RIN)

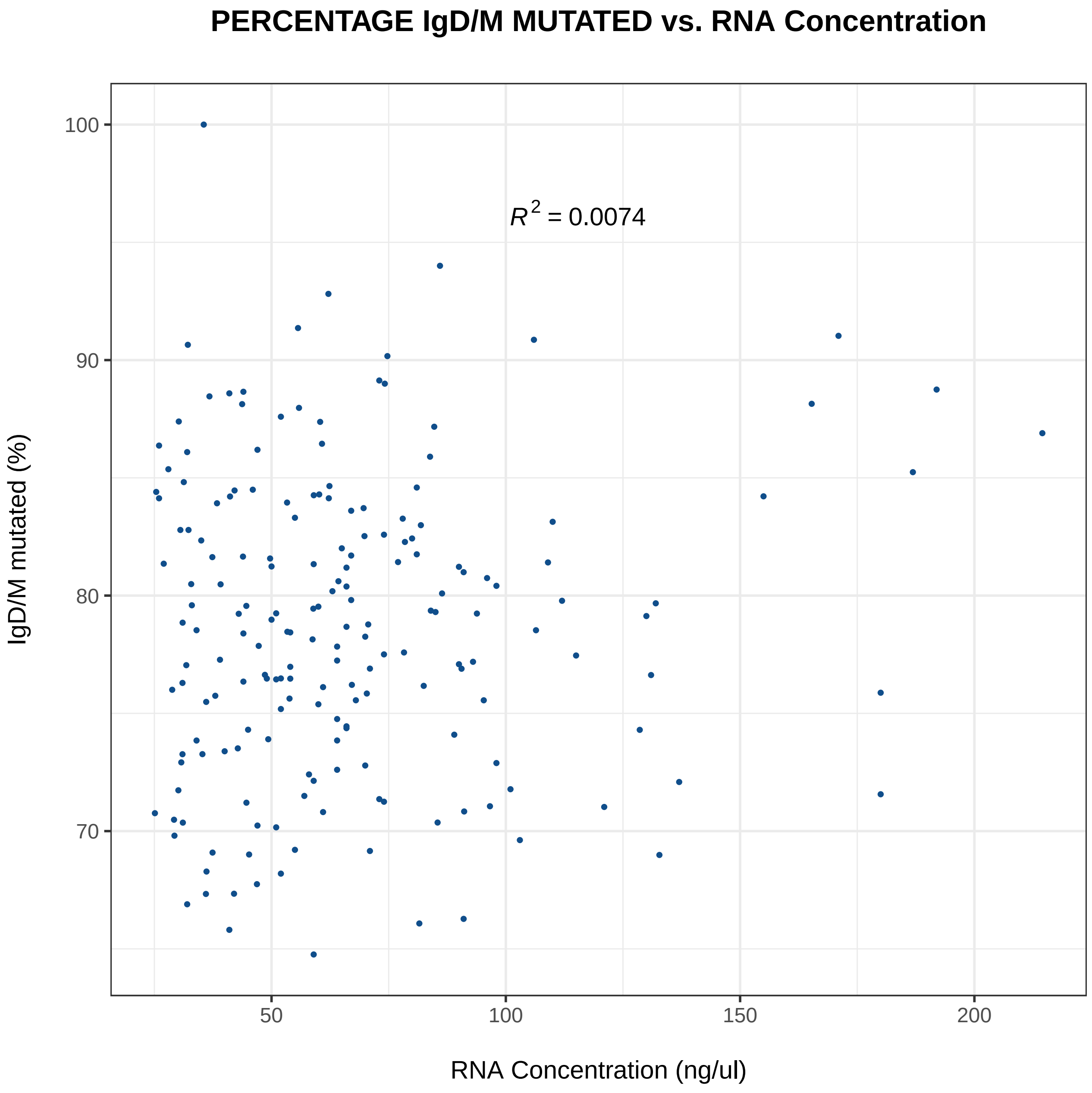

PERCENTAGE IgD/M MUTATED vs. RNA Concentration
$R^2 = 0.0074$
IgD/M mutated (%)
100
90
80
70
50
100
150
200
RNA Concentration (ng/ul)

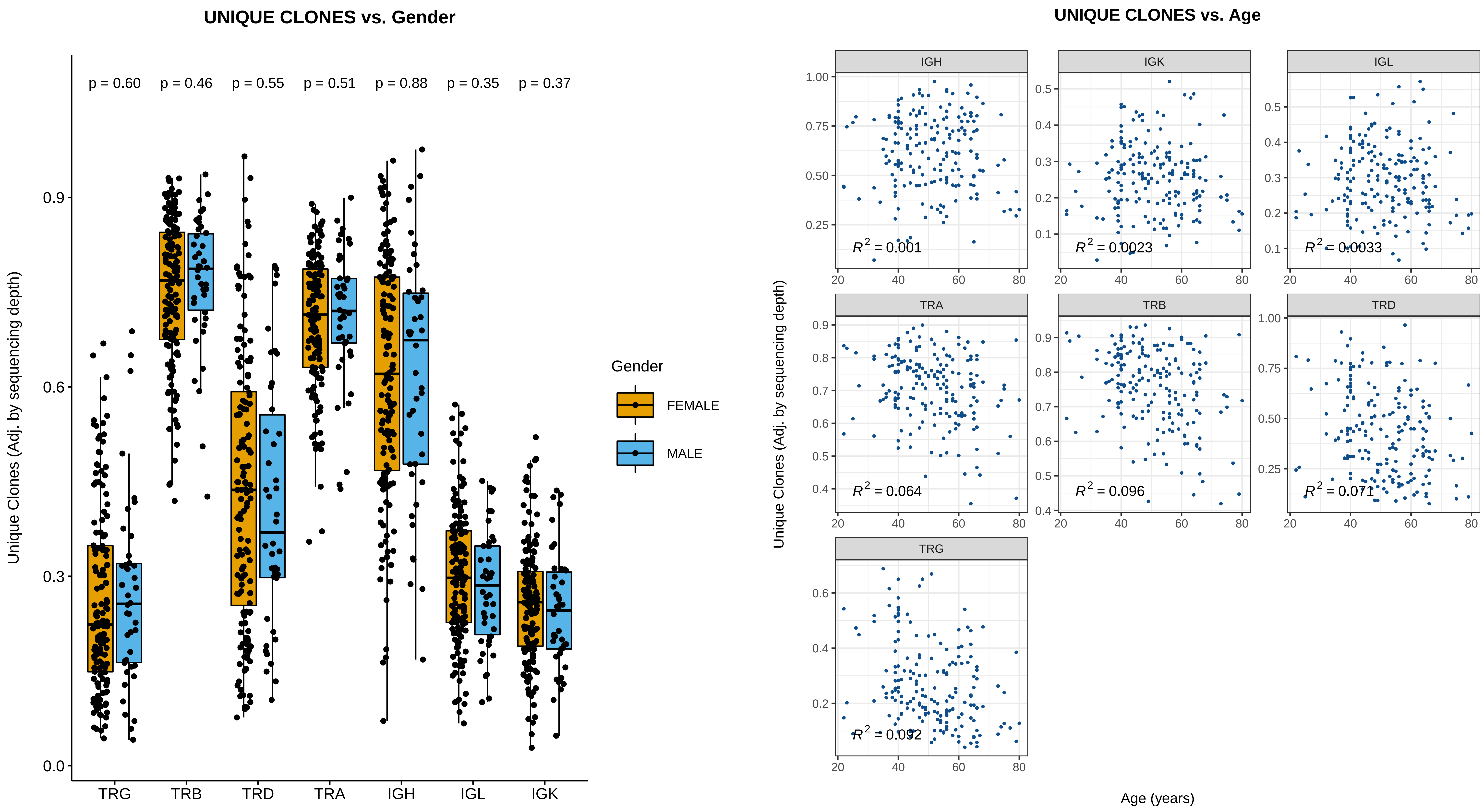
UNIQUE CLONES vs. Gender
p = 0.60
p = 0.46
p = 0.55
p = 0.51
p = 0.88
p = 0.35
p = 0.37
Unique Clones (Adj. by sequencing depth)
0.0
0.3
0.6
0.9
TRG
TRB
TRD
TRA
IGH
IGL
IGK
Gender
FEMALE
MALE
UNIQUE CLONES vs. Age
IGH
IGK
IGL
TRA
TRB
TRD
TRG
$R^2 = 0.001$
$R^2 = 0.0023$
$R^2 = 0.0033$
$R^2 = 0.064$
$R^2 = 0.096$
$R^2 = 0.071$
$R^2 = 0.092$
Unique Clones (Adj. by sequencing depth)
Age (years)

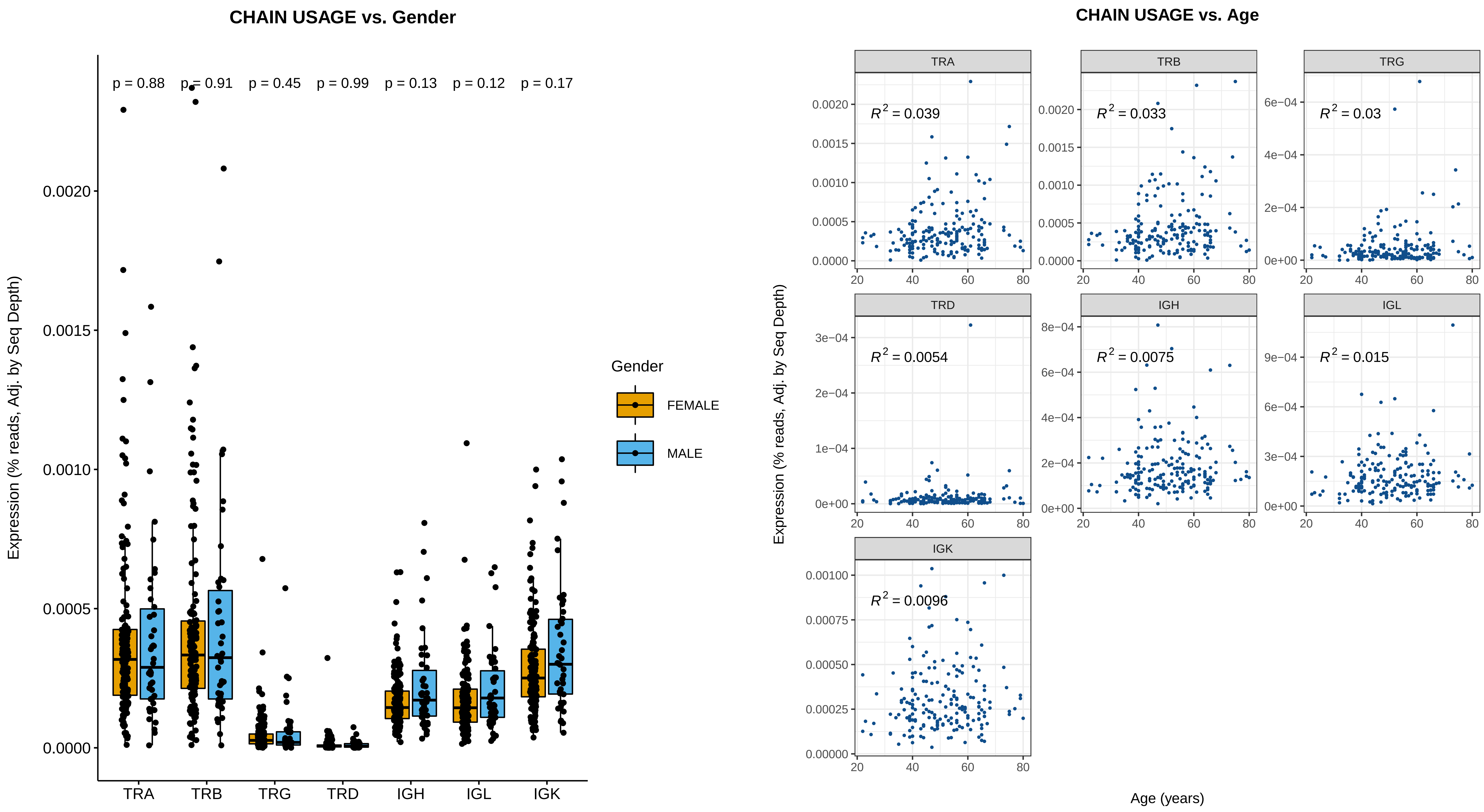

CHAIN USAGE vs. Gender
p = 0.88
p = 0.91
p = 0.45
p = 0.99
p = 0.13
p = 0.12
p = 0.17
Expression (% reads, Adj. by Seq Depth)
0.0020
0.0015
0.0010
0.0005
0.0000
TRA
TRB
TRG
TRD
IGH
IGL
IGK
Gender
FEMALE
MALE
CHAIN USAGE vs. Age
TRA
R^2 = 0.039
TRB
R^2 = 0.033
TRG
R^2 = 0.03
TRD
R^2 = 0.0054
IGH
R^2 = 0.0075
IGL
R^2 = 0.015
IGK
R^2 = 0.0096
Expression (% reads, Adj. by Seq Depth)
Age (years)

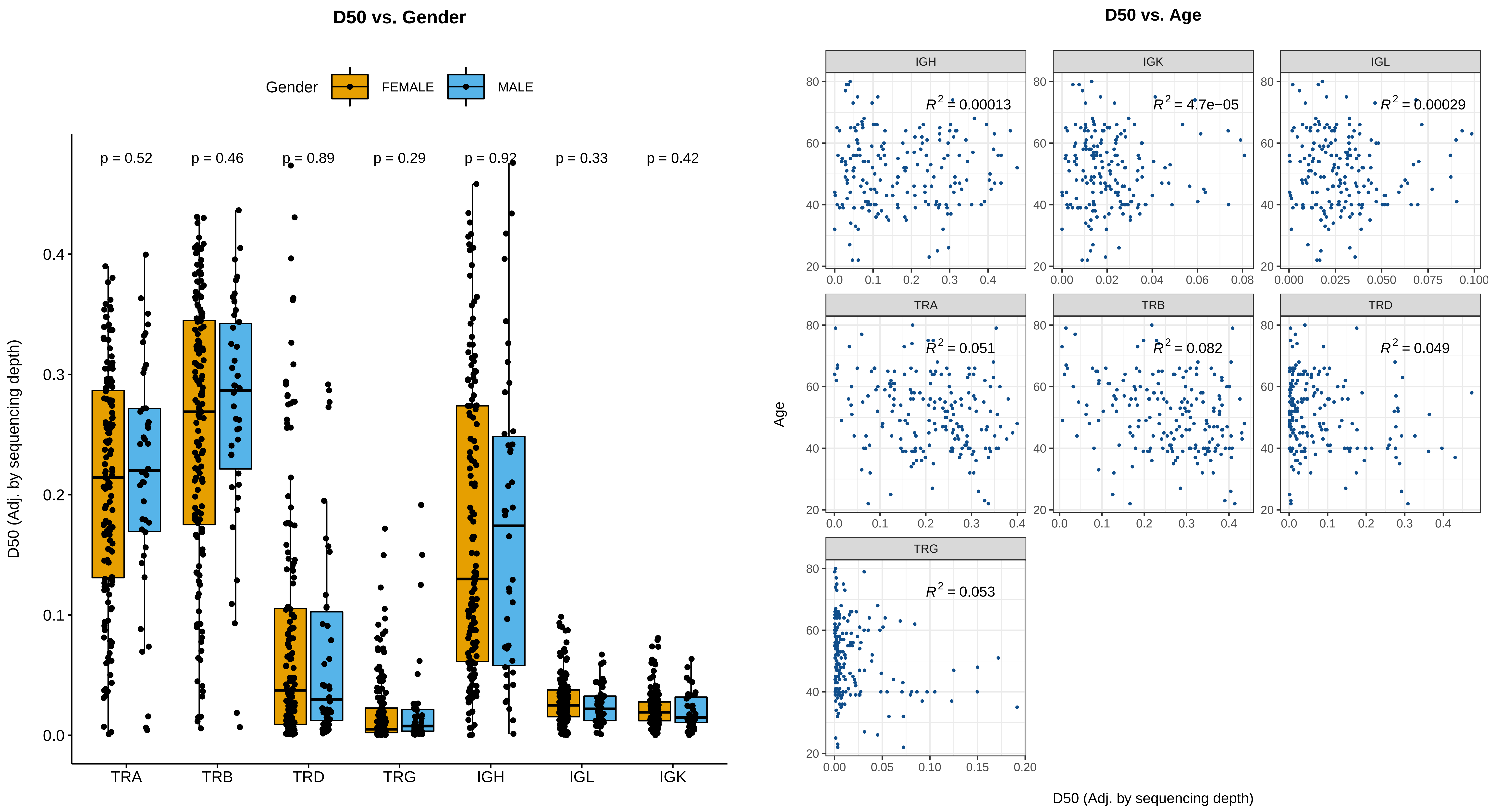

D50 vs. Gender
Gender
FEMALE
MALE
p = 0.52
p = 0.46
p = 0.89
p = 0.29
p = 0.92
p = 0.33
p = 0.42
D50 (Adj. by sequencing depth)
0.0
0.1
0.2
0.3
0.4
TRA
TRB
TRD
TRG
IGH
IGL
IGK
D50 vs. Age
IGH
IGK
IGL
TRA
TRB
TRD
TRG
$R^2 = 0.00013$
$R^2 = 4.7e-05$
$R^2 = 0.00029$
$R^2 = 0.051$
$R^2 = 0.082$
$R^2 = 0.049$
$R^2 = 0.053$
Age
D50 (Adj. by sequencing depth)

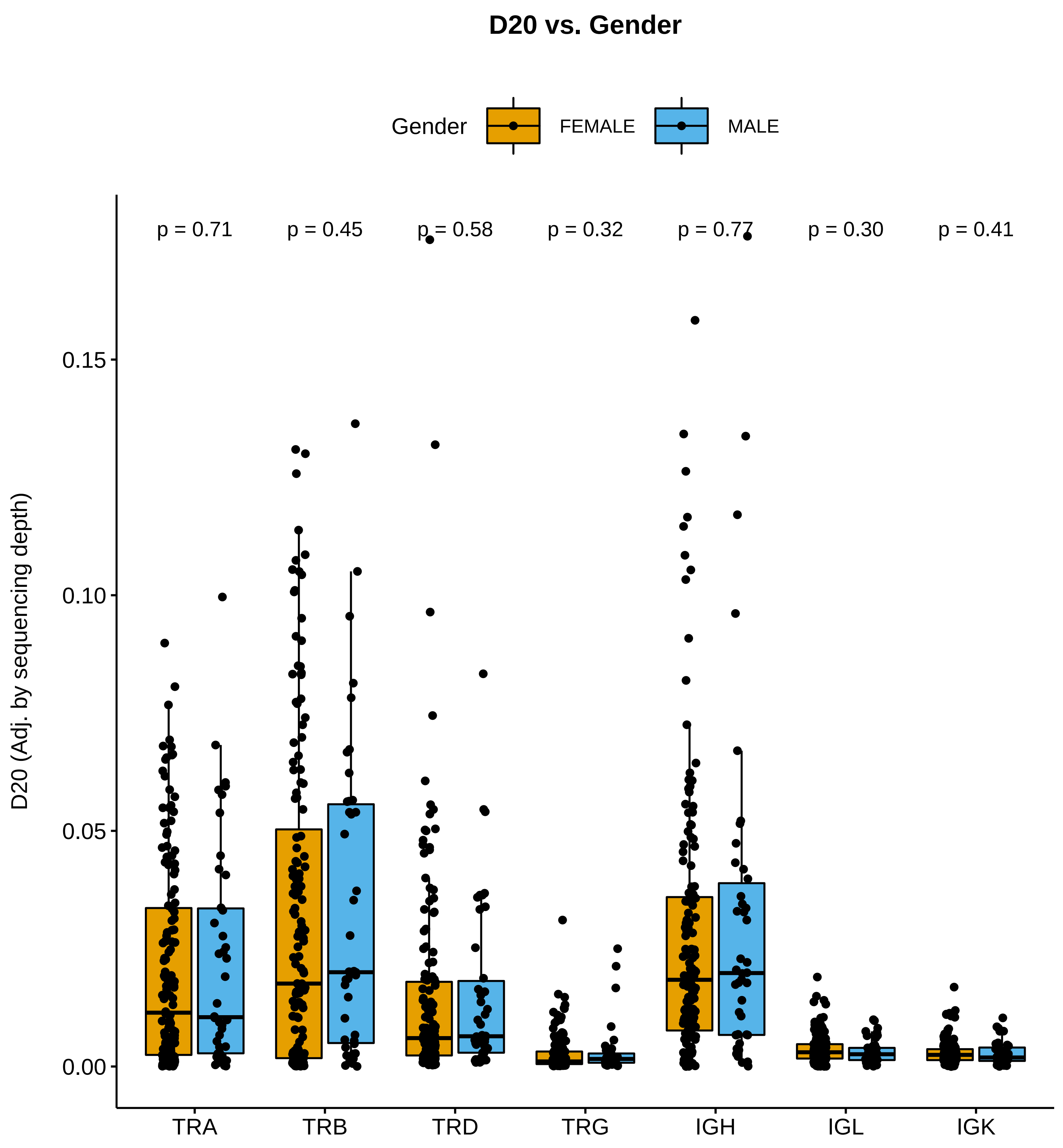

D20 vs. Gender
Gender FEMALE MALE
p = 0.71
p = 0.45
p = 0.58
p = 0.32
p = 0.77
p = 0.30
p = 0.41
D20 (Adj. by sequencing depth)
0.00
0.05
0.10
0.15
TRA
TRB
TRD
TRG
IGH
IGL
IGK

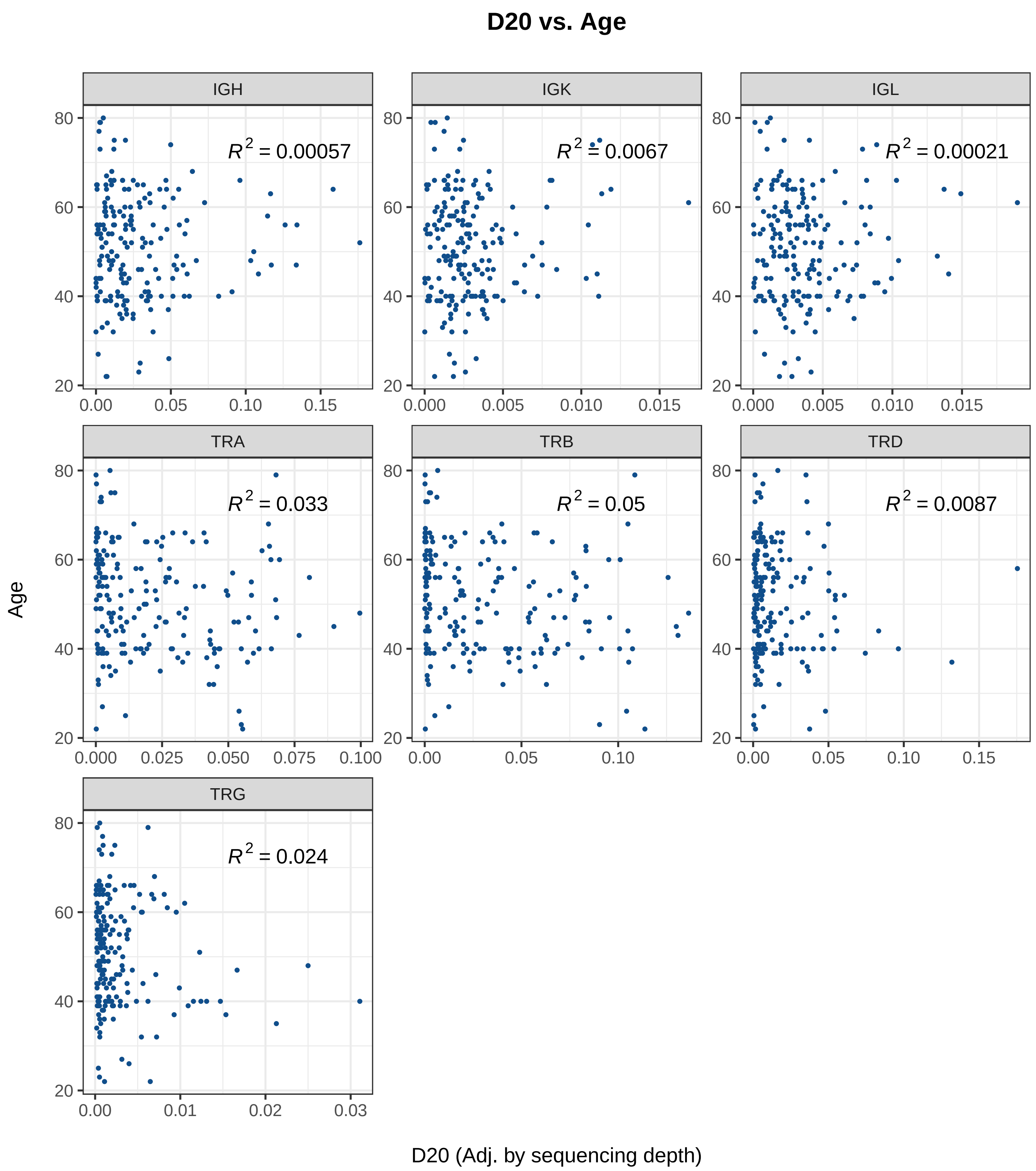

D20 vs. Age
IGH
R^2 = 0.00057
IGK
R^2 = 0.0067
IGL
R^2 = 0.00021
TRA
R^2 = 0.033
TRB
R^2 = 0.05
TRD
R^2 = 0.0087
TRG
R^2 = 0.024
Age
D20 (Adj. by sequencing depth)

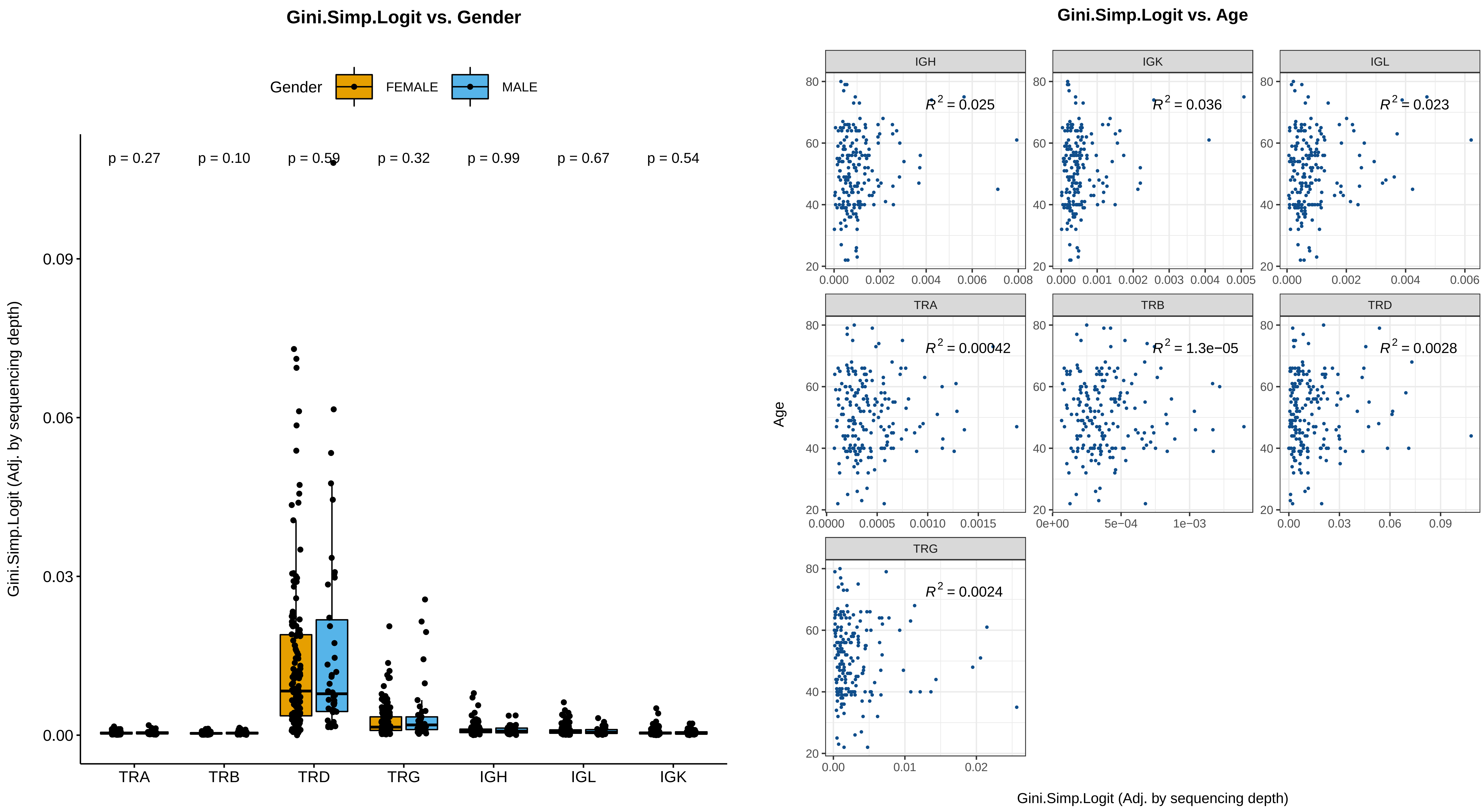

Gini.Simp.Logit vs. Gender
Gender
FEMALE
MALE
p = 0.27
p = 0.10
p = 0.59
p = 0.32
p = 0.99
p = 0.67
p = 0.54
Gini.Simp.Logit (Adj. by sequencing depth)
0.00
0.03
0.06
0.09
TRA
TRB
TRD
TRG
IGH
IGL
IGK
Gini.Simp.Logit vs. Age
IGH
IGK
IGL
TRA
TRB
TRD
TRG
R^2 = 0.025
R^2 = 0.036
R^2 = 0.023
R^2 = 0.00042
R^2 = 1.3e−05
R^2 = 0.0028
R^2 = 0.0024
Age
20
40
60
80
0.000
0.002
0.004
0.006
0.008
0.001
0.003
0.005
0.0000
0.0005
0.0010
0.0015
0e+00
5e−04
1e−03
0.00
0.03
0.06
0.09
0.01
0.02
Gini.Simp.Logit (Adj. by sequencing depth)

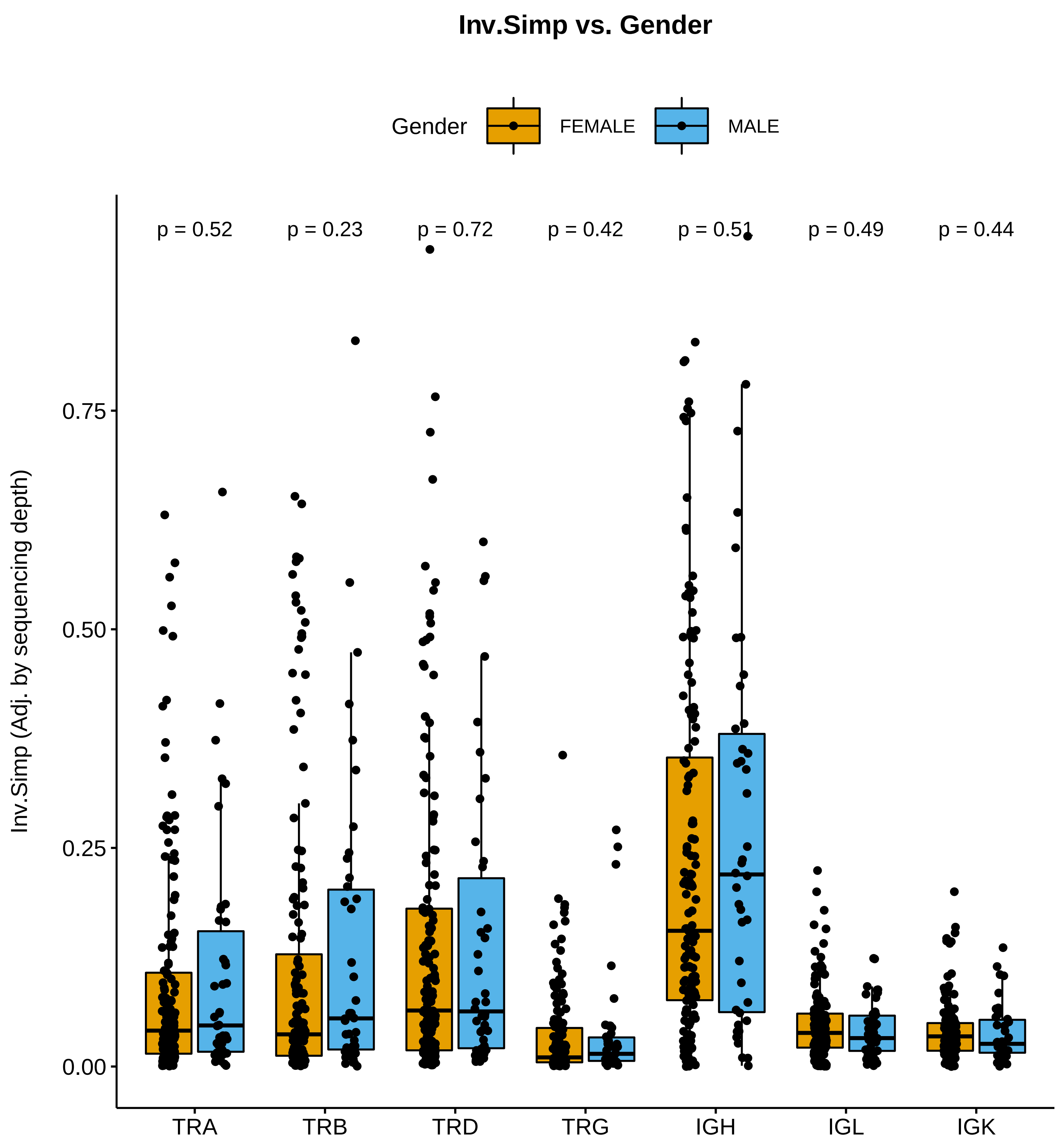
Inv.Simp vs. Gender
Gender FEMALE MALE
p = 0.52
p = 0.23
p = 0.72
p = 0.42
p = 0.51
p = 0.49
p = 0.44
Inv.Simp (Adj. by sequencing depth)
0.00
0.25
0.50
0.75
TRA
TRB
TRD
TRG
IGH
IGL
IGK

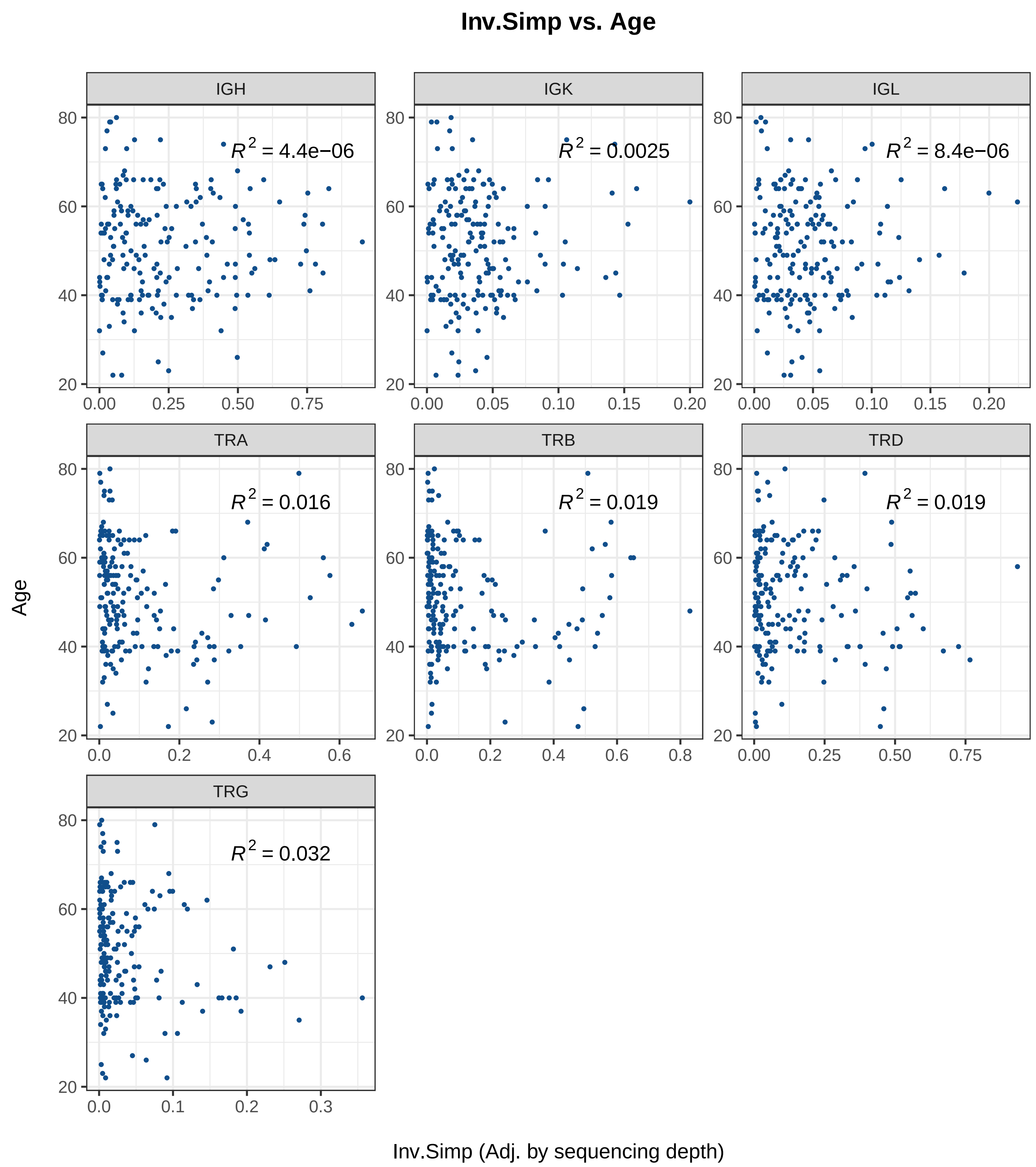
Inv.Simp vs. Age
IGH
R^2 = 4.4e−06
IGK
R^2 = 0.0025
IGL
R^2 = 8.4e−06
TRA
R^2 = 0.016
TRB
R^2 = 0.019
TRD
R^2 = 0.019
TRG
R^2 = 0.032
Age
Inv.Simp (Adj. by sequencing depth)

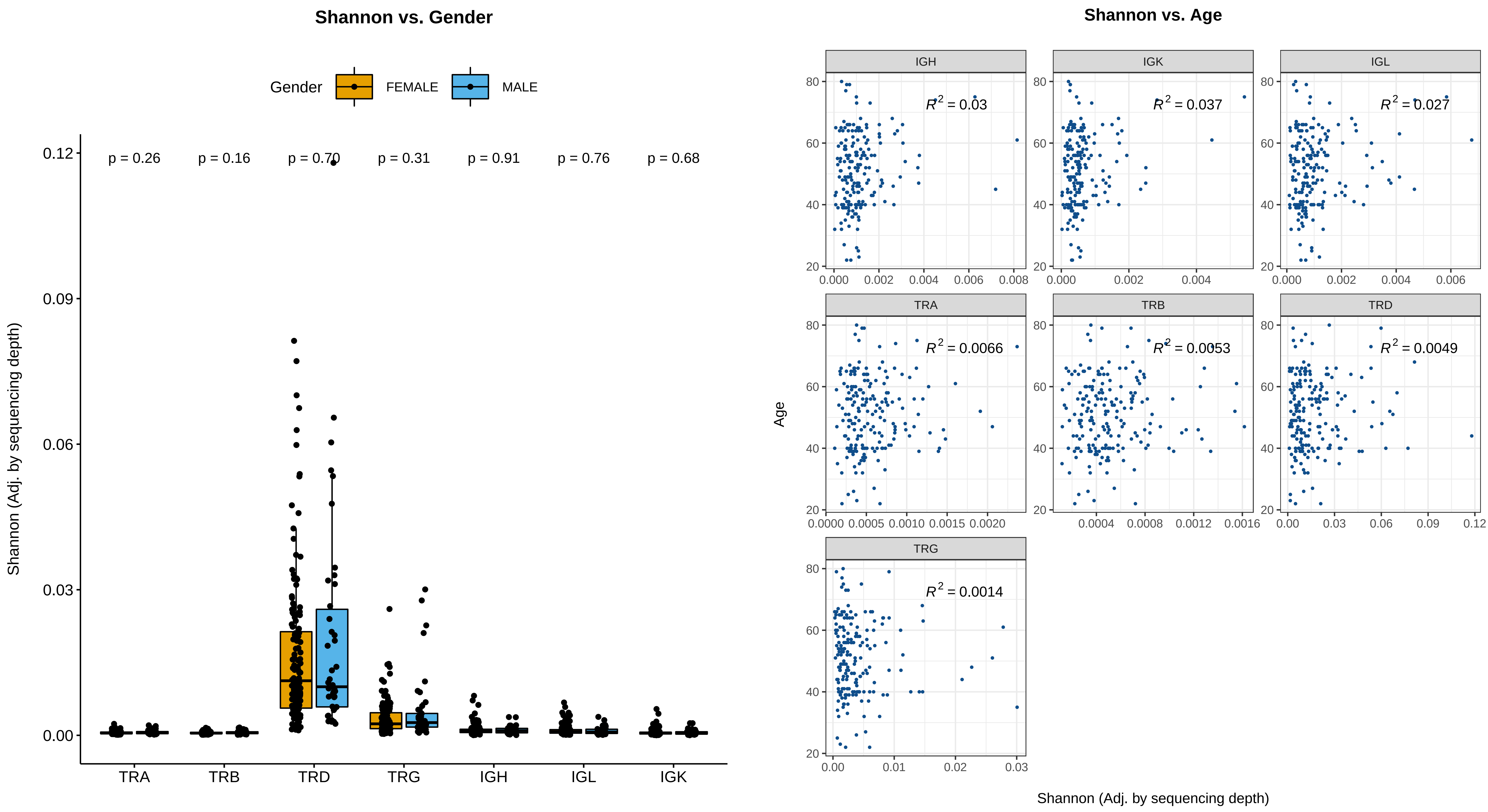

Shannon vs. Gender
Gender FEMALE MALE
Shannon (Adj. by sequencing depth)
p = 0.26
p = 0.16
p = 0.70
p = 0.31
p = 0.91
p = 0.76
p = 0.68
0.12
0.09
0.06
0.03
0.00
TRA
TRB
TRD
TRG
IGH
IGL
IGK
Shannon vs. Age
IGH
IGK
IGL
TRA
TRB
TRD
TRG
R^2 = 0.03
R^2 = 0.037
R^2 = 0.027
R^2 = 0.0066
R^2 = 0.0053
R^2 = 0.0049
R^2 = 0.0014
Age
Shannon (Adj. by sequencing depth)

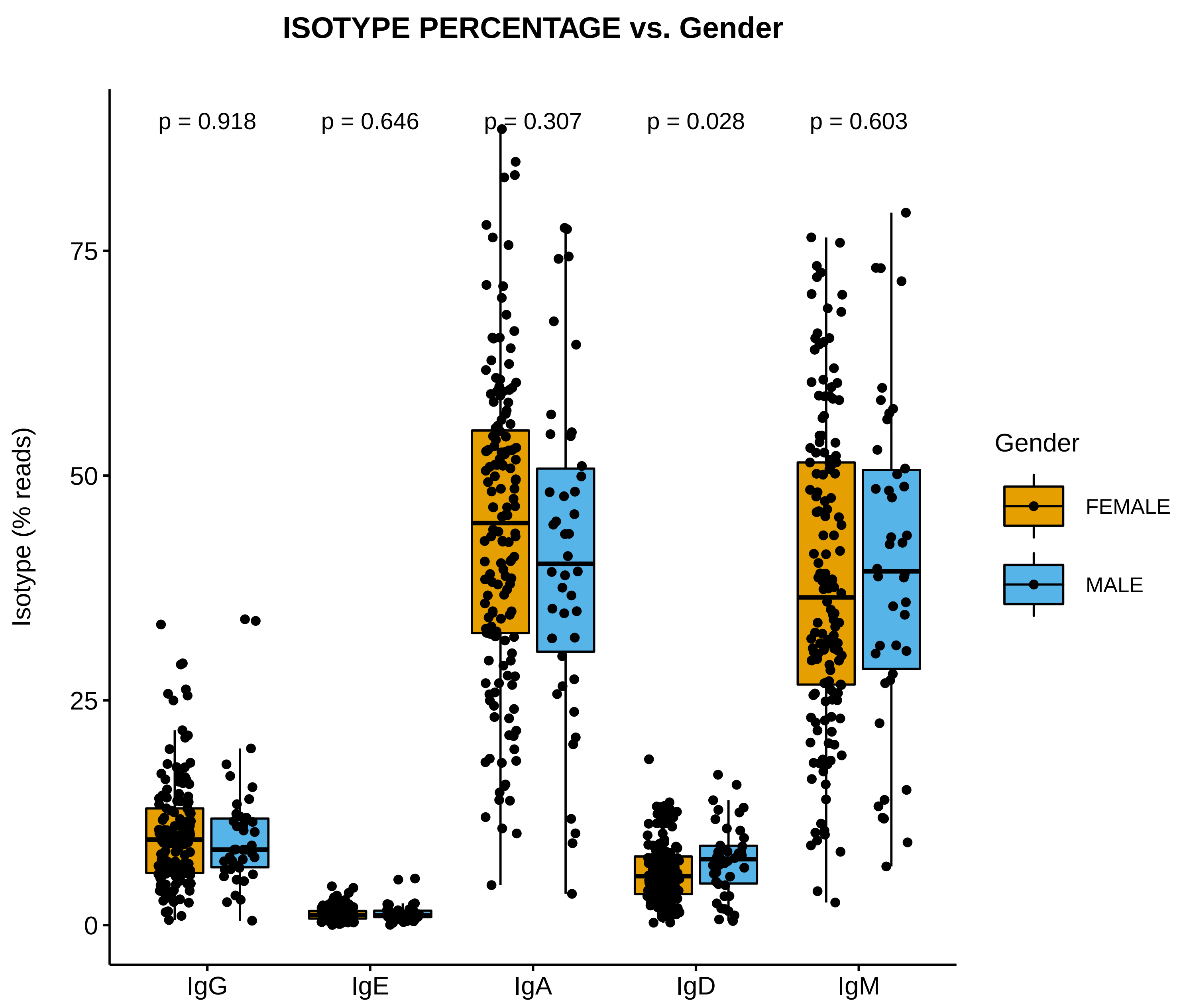
ISOTYPE PERCENTAGE vs. Gender
p = 0.918
p = 0.646
p = 0.307
p = 0.028
p = 0.603
Isotype (% reads)
0
25
50
75
IgG
IgE
IgA
IgD
IgM
Gender
FEMALE
MALE

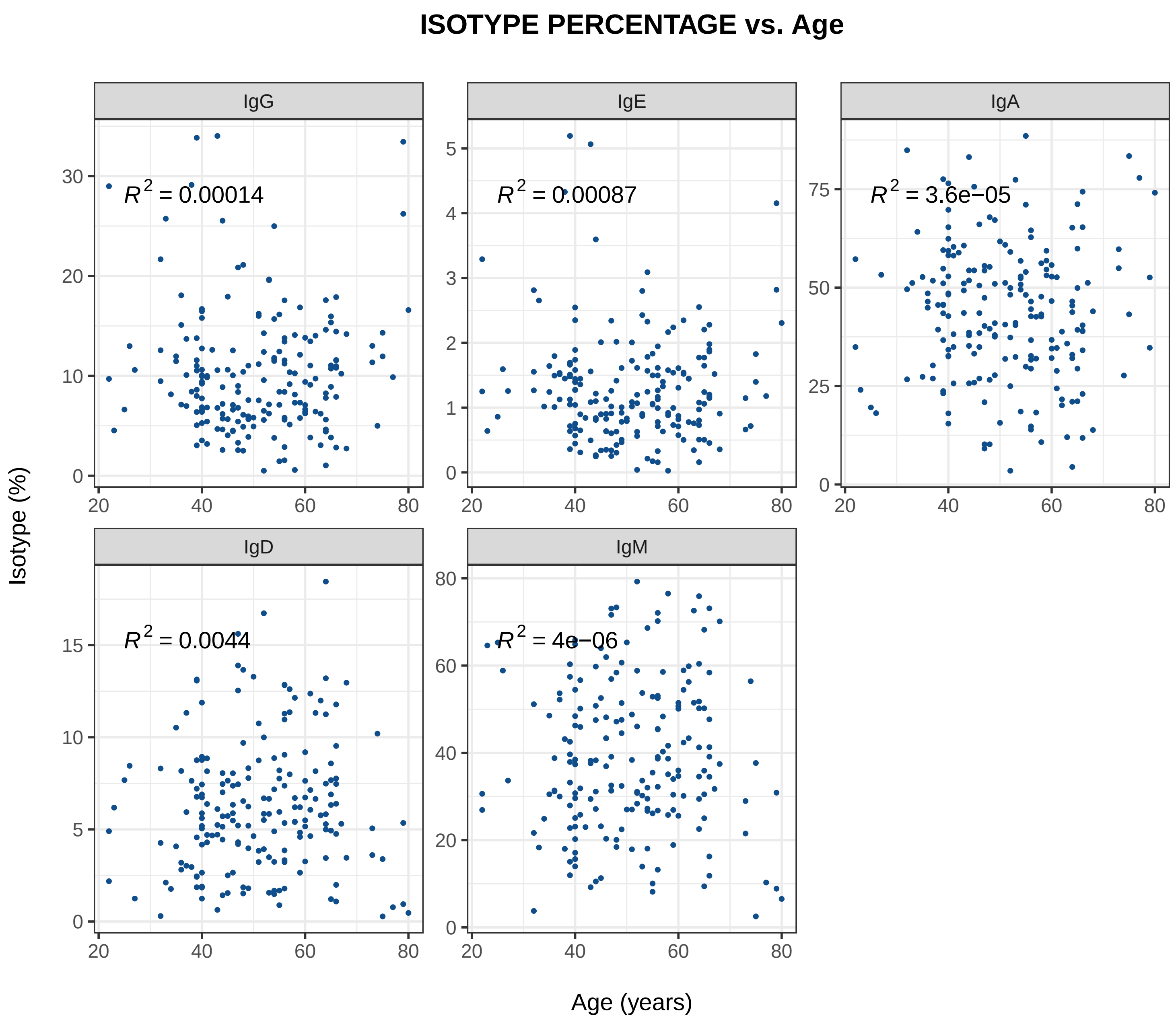
ISOTYPE PERCENTAGE vs. Age
IgG
IgE
IgA
IgD
IgM
R^2 = 0.00014
R^2 = 0.00087
R^2 = 3.6e−05
R^2 = 0.0044
R^2 = 4e−06
Isotype (%)
Age (years)

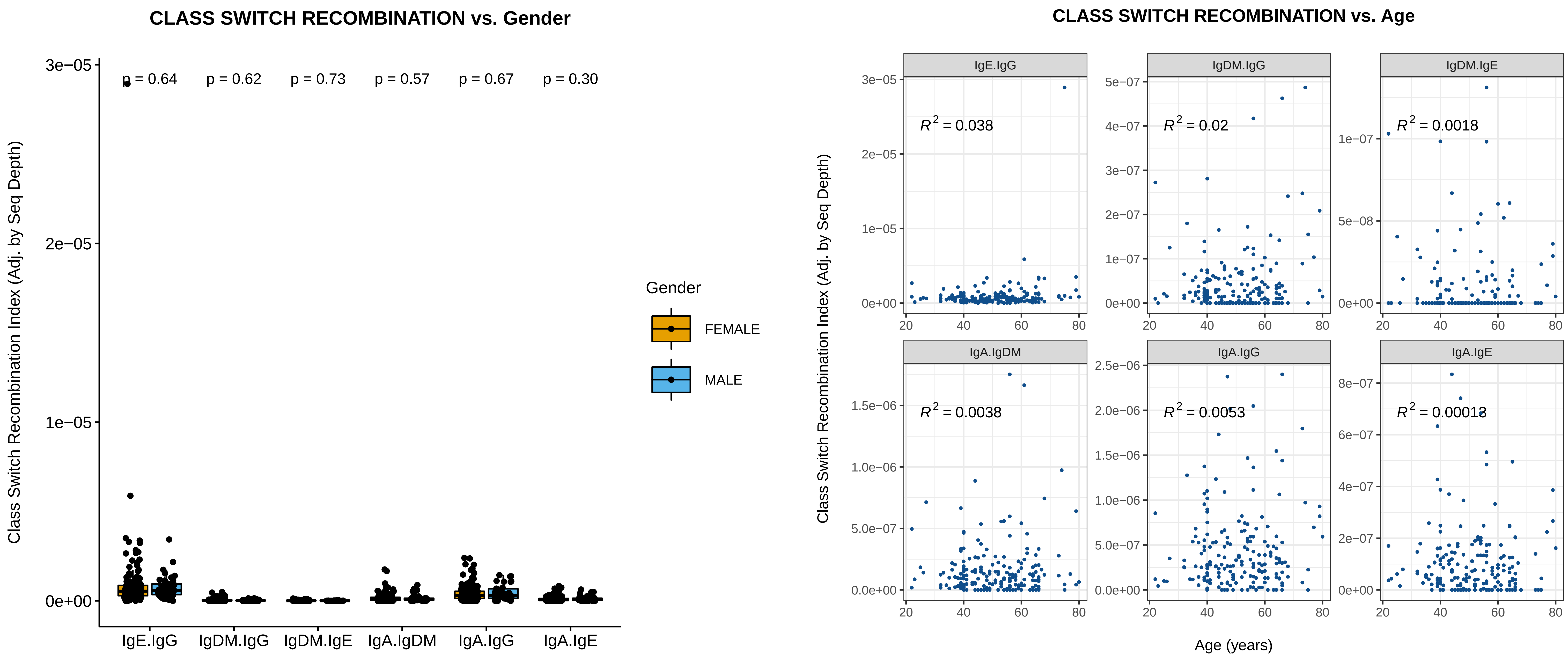

CLASS SWITCH RECOMBINATION vs. Gender
Class Switch Recombination Index (Adj. by Seq Depth)
3e−05
2e−05
1e−05
0e+00
p = 0.64
p = 0.62
p = 0.73
p = 0.57
p = 0.67
p = 0.30
IgE.IgG
IgDM.IgG
IgDM.IgE
IgA.IgDM
IgA.IgG
IgA.IgE
Gender
FEMALE
MALE
CLASS SWITCH RECOMBINATION vs. Age
Class Switch Recombination Index (Adj. by Seq Depth)
IgE.IgG
$R^2 = 0.038$
IgDM.IgG
$R^2 = 0.02$
IgDM.IgE
$R^2 = 0.0018$
IgA.IgDM
$R^2 = 0.0038$
IgA.IgG
$R^2 = 0.0053$
IgA.IgE
$R^2 = 0.00013$
20
40
60
80
Age (years)

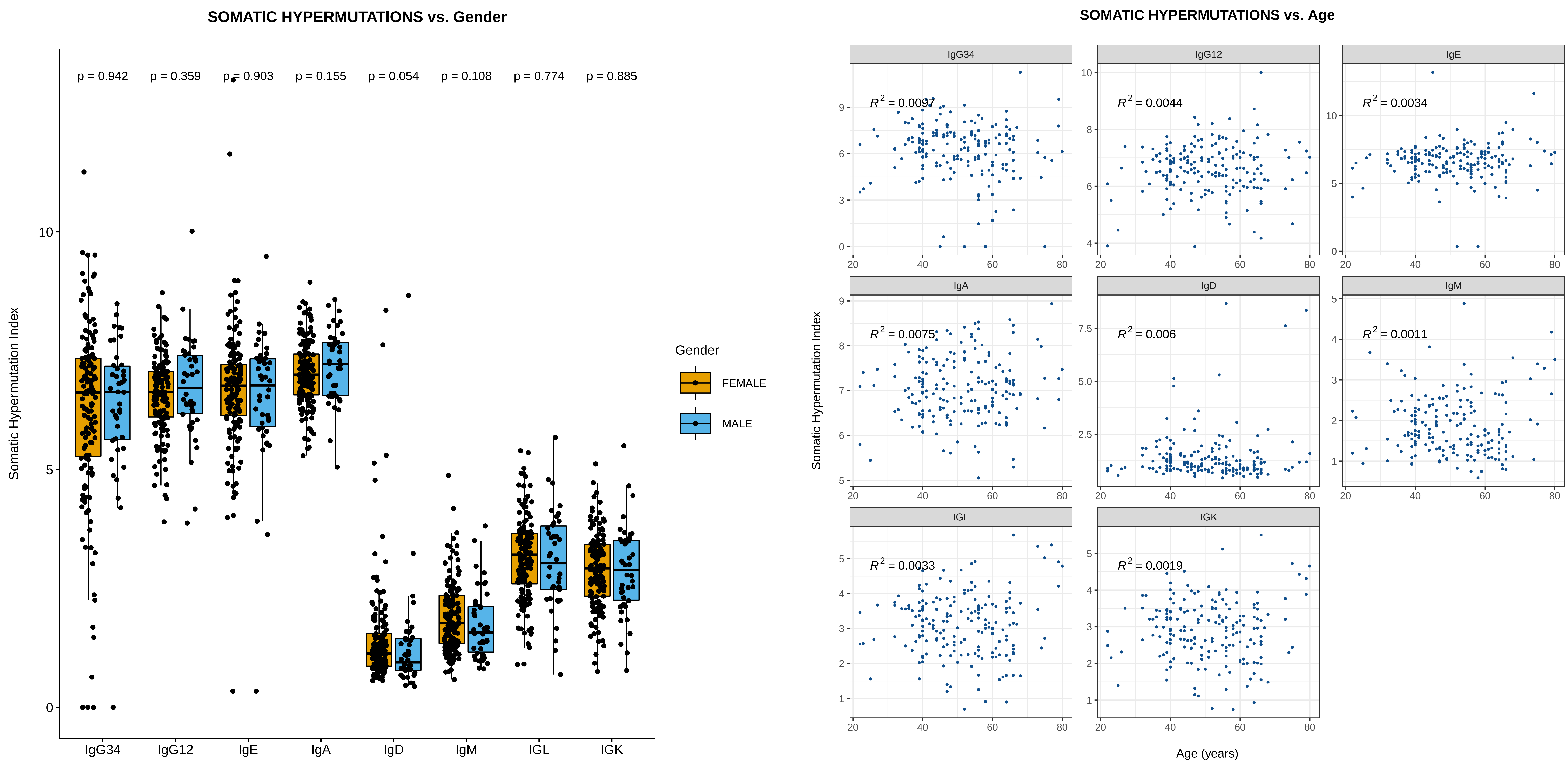
SOMATIC HYPERMUTATIONS vs. Gender
p = 0.942
p = 0.359
p = 0.903
p = 0.155
p = 0.054
p = 0.108
p = 0.774
p = 0.885
Somatic Hypermutation Index
IgG34
IgG12
IgE
IgA
IgD
IgM
IGL
IGK
Gender
FEMALE
MALE
SOMATIC HYPERMUTATIONS vs. Age
IgG34
R^2 = 0.0097
IgG12
R^2 = 0.0044
IgE
R^2 = 0.0034
IgA
R^2 = 0.0075
IgD
R^2 = 0.006
IgM
R^2 = 0.0011
IGL
R^2 = 0.0033
IGK
R^2 = 0.0019
Somatic Hypermutation Index
Age (years)

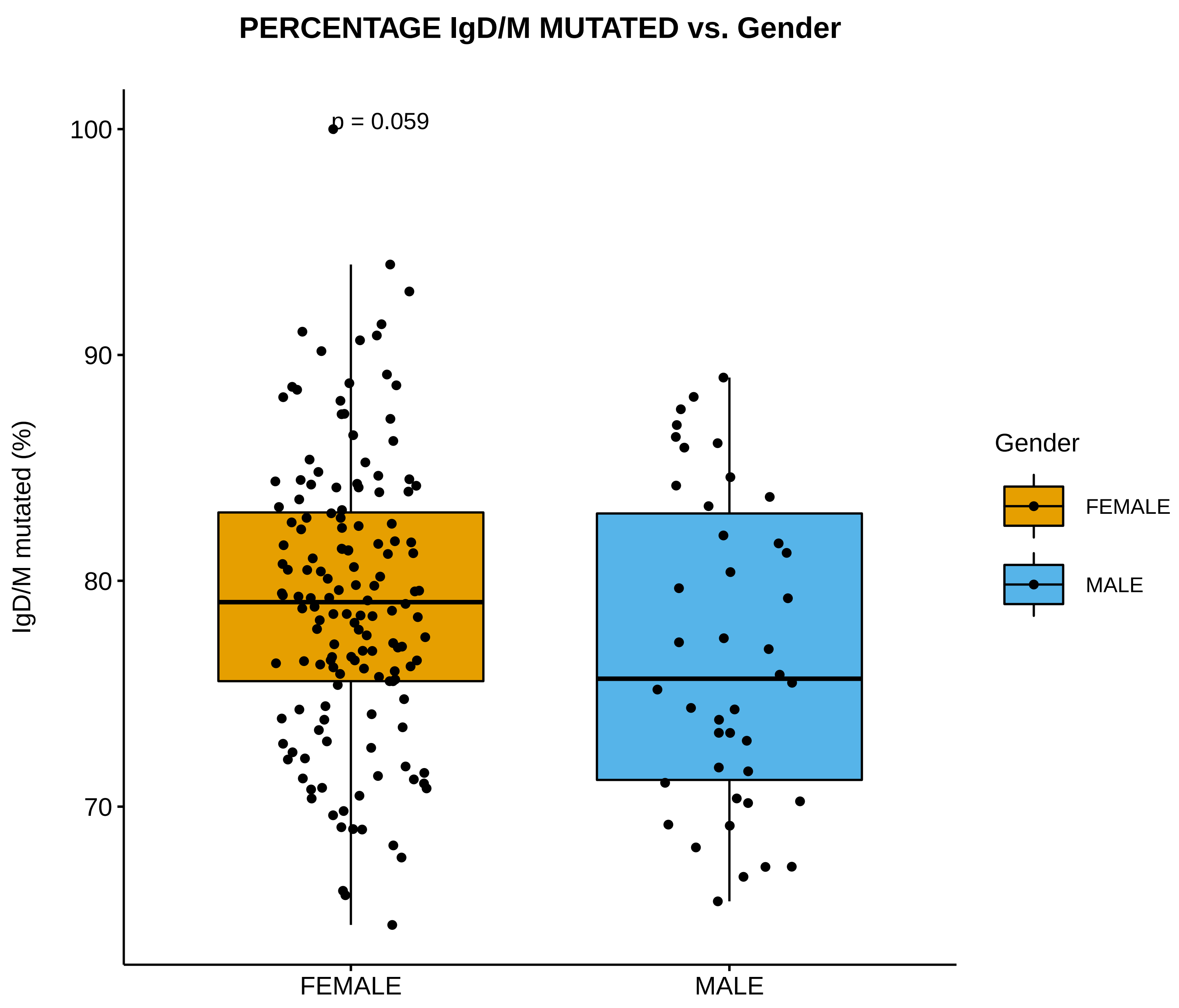
PERCENTAGE IgD/M MUTATED vs. Gender
p = 0.059
100
90
80
70
IgD/M mutated (%)
FEMALE
MALE
Gender
FEMALE
MALE

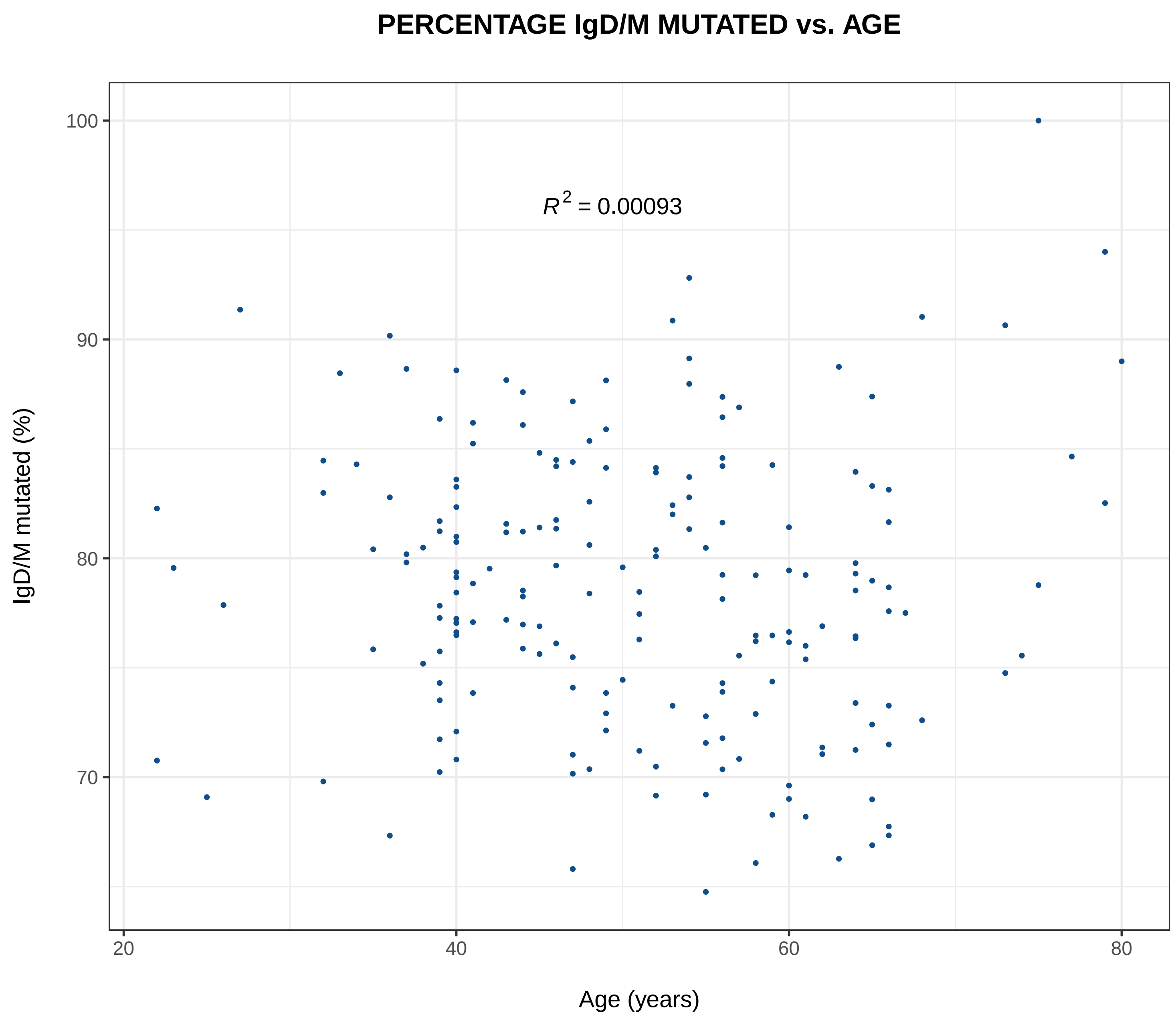
PERCENTAGE IgD/M MUTATED vs. AGE
R 2 = 0.00093
100
90
80
70
IgD/M mutated (%)
20
40
60
80
Age (years)
