# Supplementary material for: Seven-chain adaptive immune receptor repertoire analysis in rheumatoid arthritis reveals novel features associated with disease and clinically relevant phenotypes
Source: Genome Biol. 2024 Mar 11;25:68. doi: 10.1186/s13059-024-03210-0 (PMC10926600; doi:10.1186/s13059-024-03210-0)
Supplement: Supplementary file 5 — Additional file 5: Figure S2. Chain-wide characterization of the AIRR diversity in rheumatoid arthritis phenotypes. The statistical significance and effect size of the association between each diversity measure and clinical phenotype are shown. The heatmap is colored according to the effect size of the association at the chain level. Details on the direction of the effect size are provided in Table S4. Abbreviations: P<0.05; **, P<5.00e-03; ***,P<5.00e-04; ****, P<5.00e-05. [file 13059_2024_3210_MOESM5_ESM.pdf]

Fig S2. Chain-wide characterization of the AIRR diversity in rheumatoid arthritis phenotypes. The statistical significance and effect size of the association between each diversity measure and clinical phenotype are shown. The heatmap is colored according to the effect size of the association at the chain level. Details on the direction of the effect size are provided in **Table S4**. Abbreviations: P<0.05; **, P<5.00e-03; ***, P<5.00e-04; ****, P<5.00e-05.

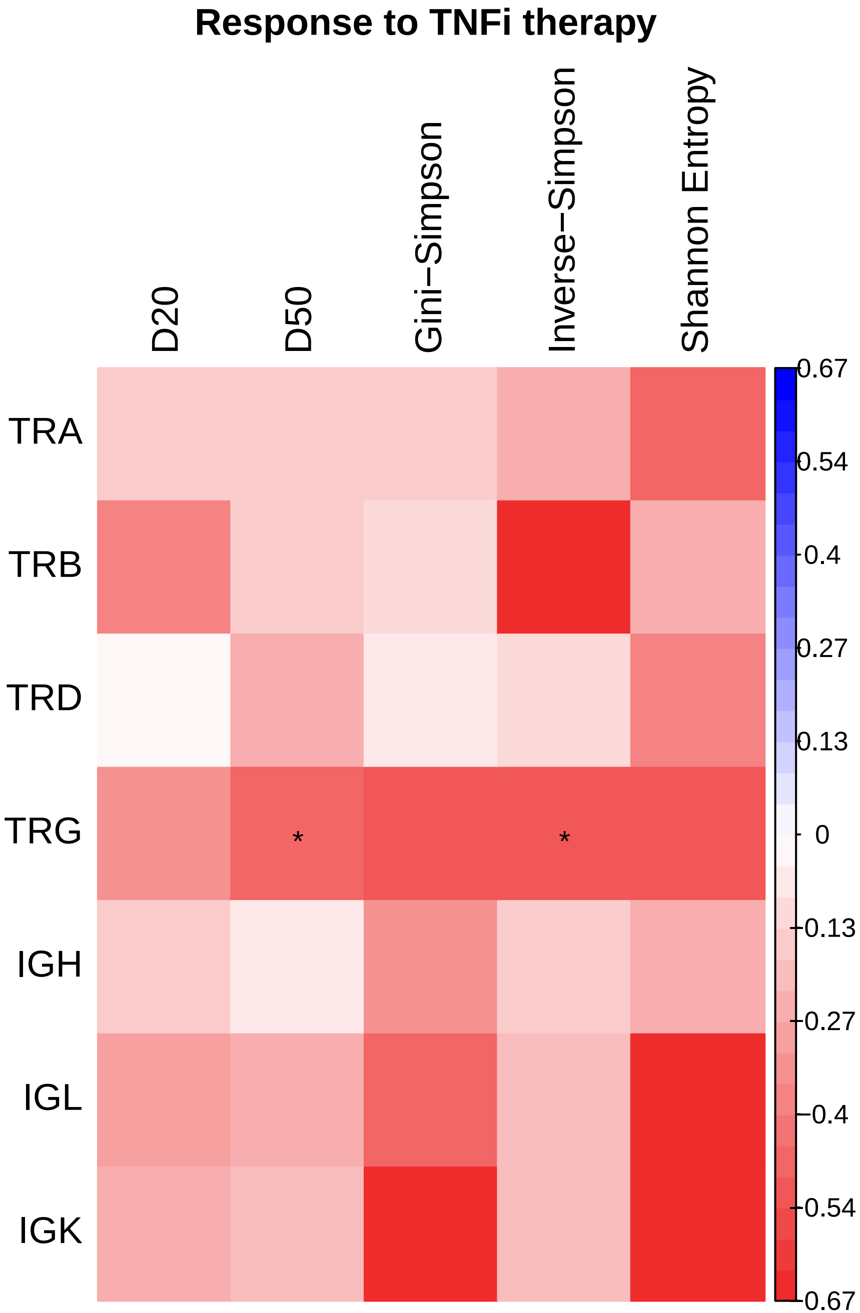

Response to TNFi therapy
D20
D50
Gini−Simpson
Inverse−Simpson
Shannon Entropy
TRA
TRB
TRD
TRG
IGH
IGL
IGK
*
*
0.67
0.54
0.4
0.27
0.13
0
−0.13
−0.27
−0.4
−0.54
−0.67

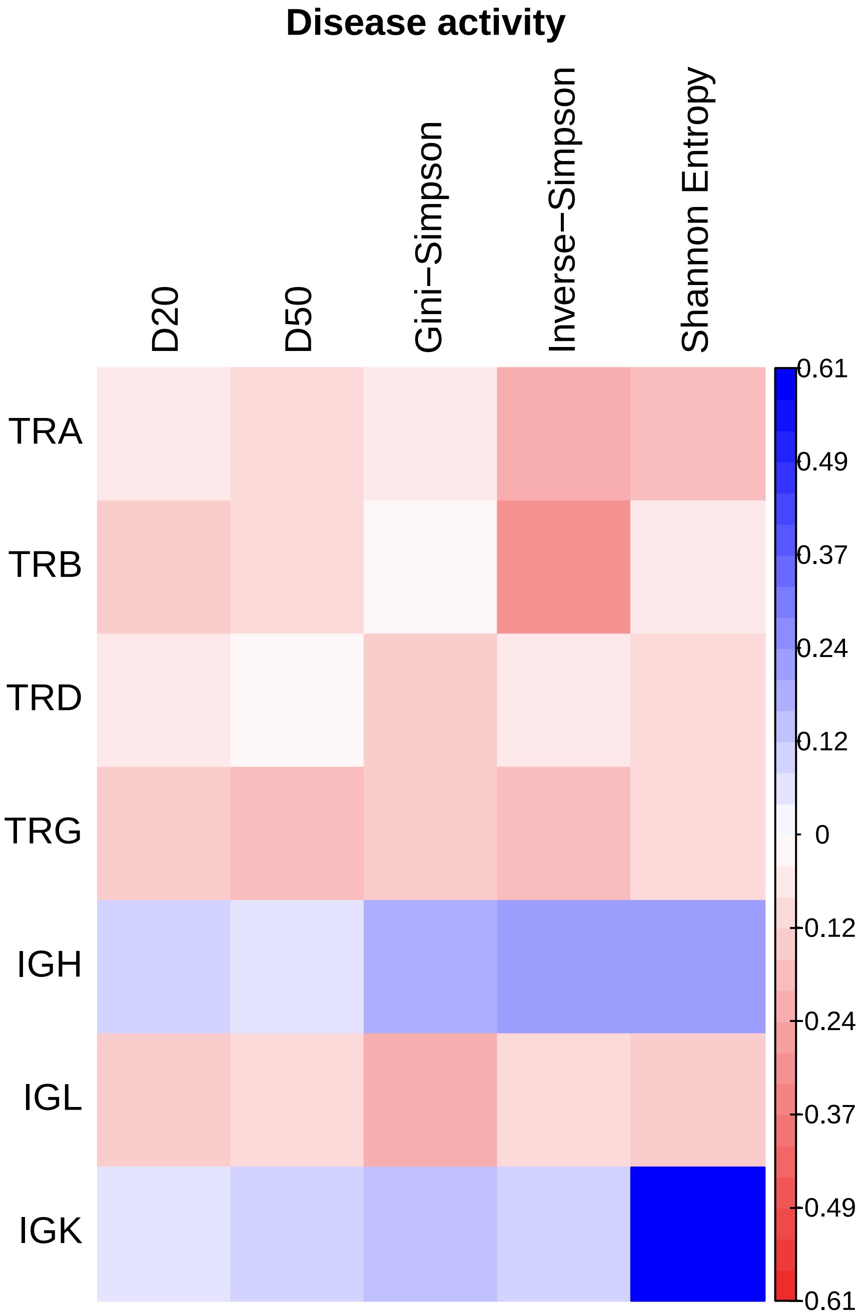

Disease activity
D20
D50
Gini−Simpson
Inverse−Simpson
Shannon Entropy
TRA
TRB
TRD
TRG
IGH
IGL
IGK
0.61
0.49
0.37
0.24
0.12
0
-0.12
-0.24
-0.37
-0.49
-0.61

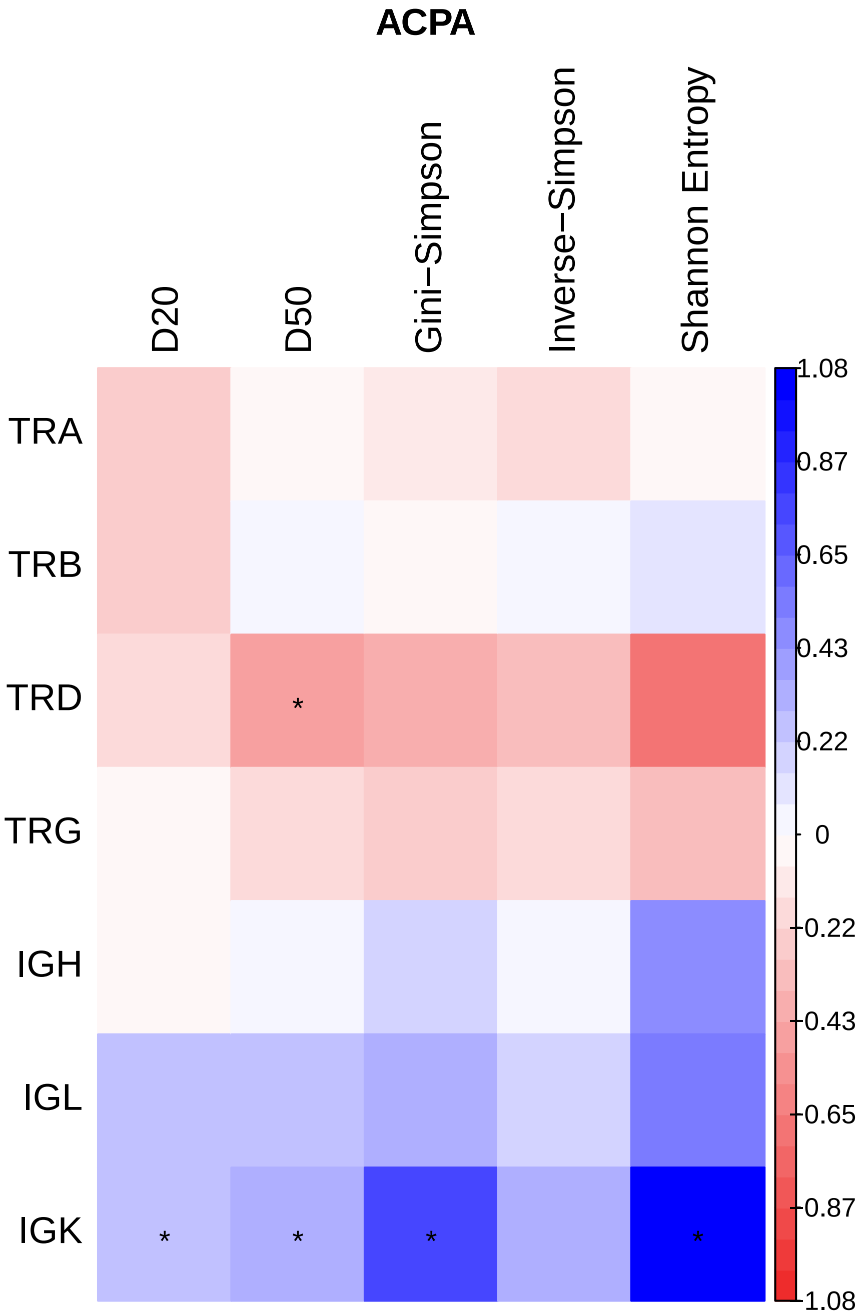

ACPA
D20
D50
Gini−Simpson
Inverse−Simpson
Shannon Entropy
TRA
TRB
TRD
TRG
IGH
IGL
IGK
1.08
0.87
0.65
0.43
0.22
0
-0.22
-0.43
-0.65
-0.87
-1.08

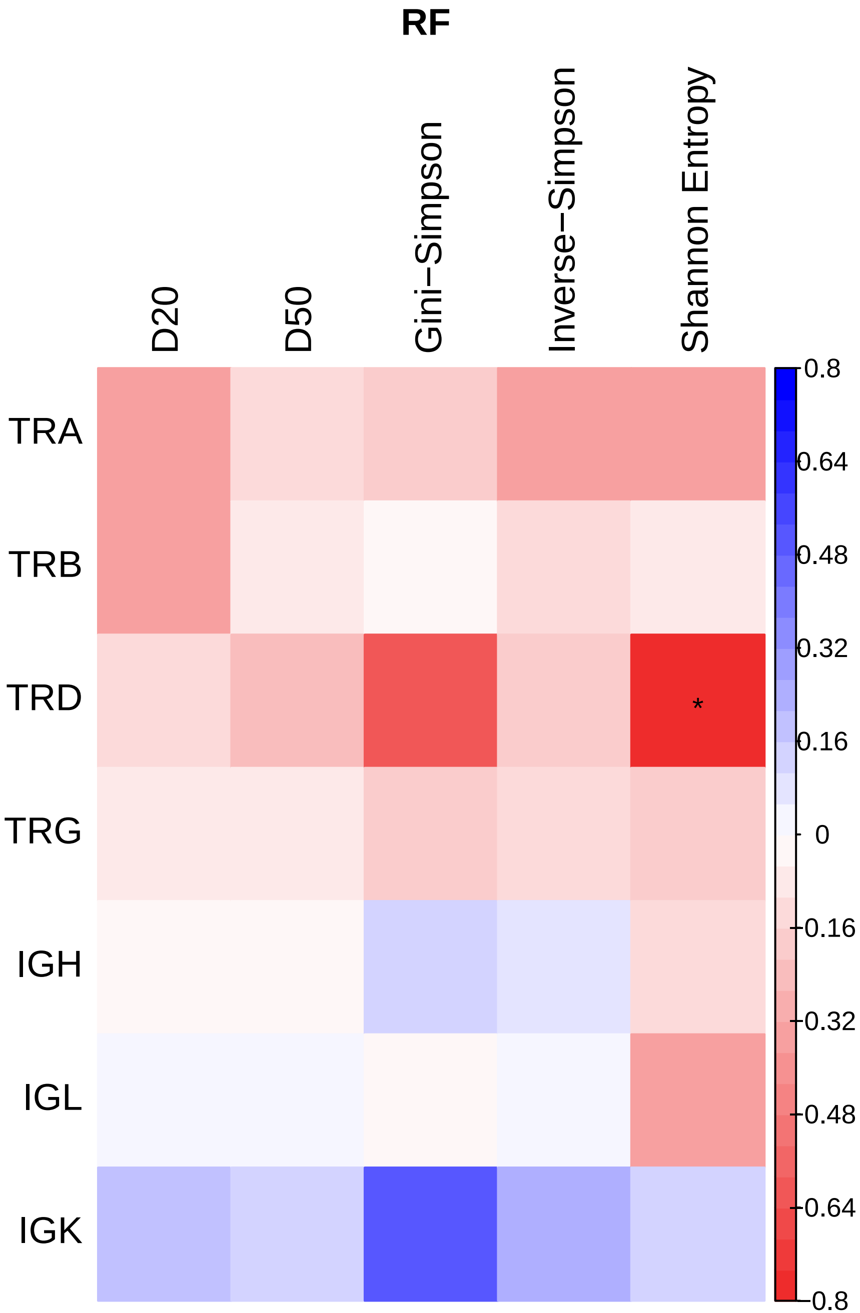
RF
D20
D50
Gini−Simpson
Inverse−Simpson
Shannon Entropy
TRA
TRB
TRD
TRG
IGH
IGL
IGK
*
0.8
0.64
0.48
0.32
0.16
0
−0.16
−0.32
−0.48
−0.64
−0.8
