# Supplementary material for: Seven-chain adaptive immune receptor repertoire analysis in rheumatoid arthritis reveals novel features associated with disease and clinically relevant phenotypes
Source: Genome Biol. 2024 Mar 11;25:68. doi: 10.1186/s13059-024-03210-0 (PMC10926600; doi:10.1186/s13059-024-03210-0)
Supplement: Supplementary file 12 — Additional file 12: Figure S3. Chain usage association with clinical phenotypes in rheumatoid arthritis. The percentage of patient UMIs mapping to each immune receptor chain is separately represented by clinical phenotype. [file 13059_2024_3210_MOESM12_ESM.pdf]

Fig S3. Chain usage association with clinical phenotypes in rheumatoid arthritis. The percentage of patient UMIs mapping to each immune receptor chain is separately represented by clinical phenotype.

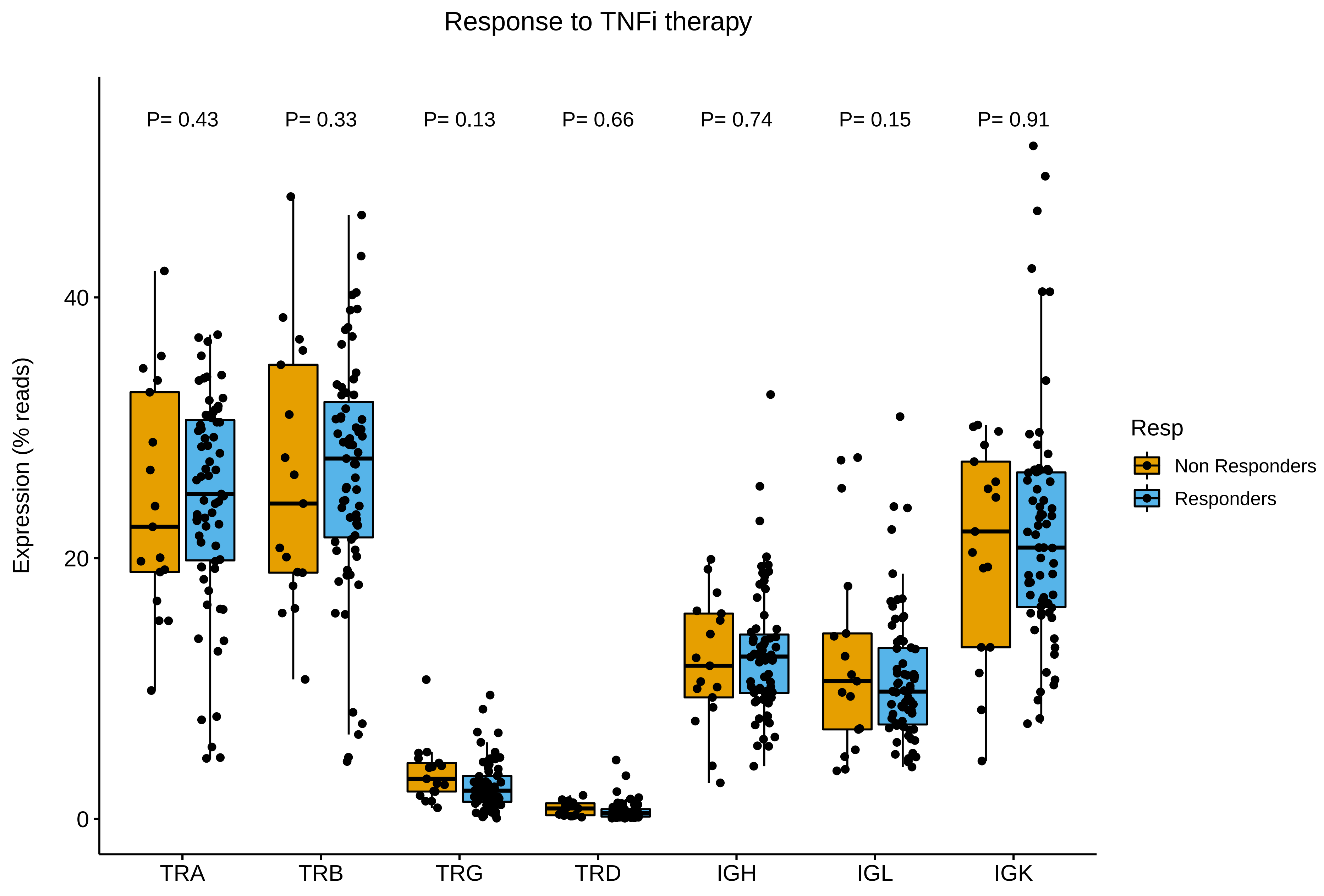

Response to TNFi therapy
P= 0.43
P= 0.33
P= 0.13
P= 0.66
P= 0.74
P= 0.15
P= 0.91
Expression (% reads)
0
20
40
TRA
TRB
TRG
TRD
IGH
IGL
IGK
Resp
Non Responders
Responders

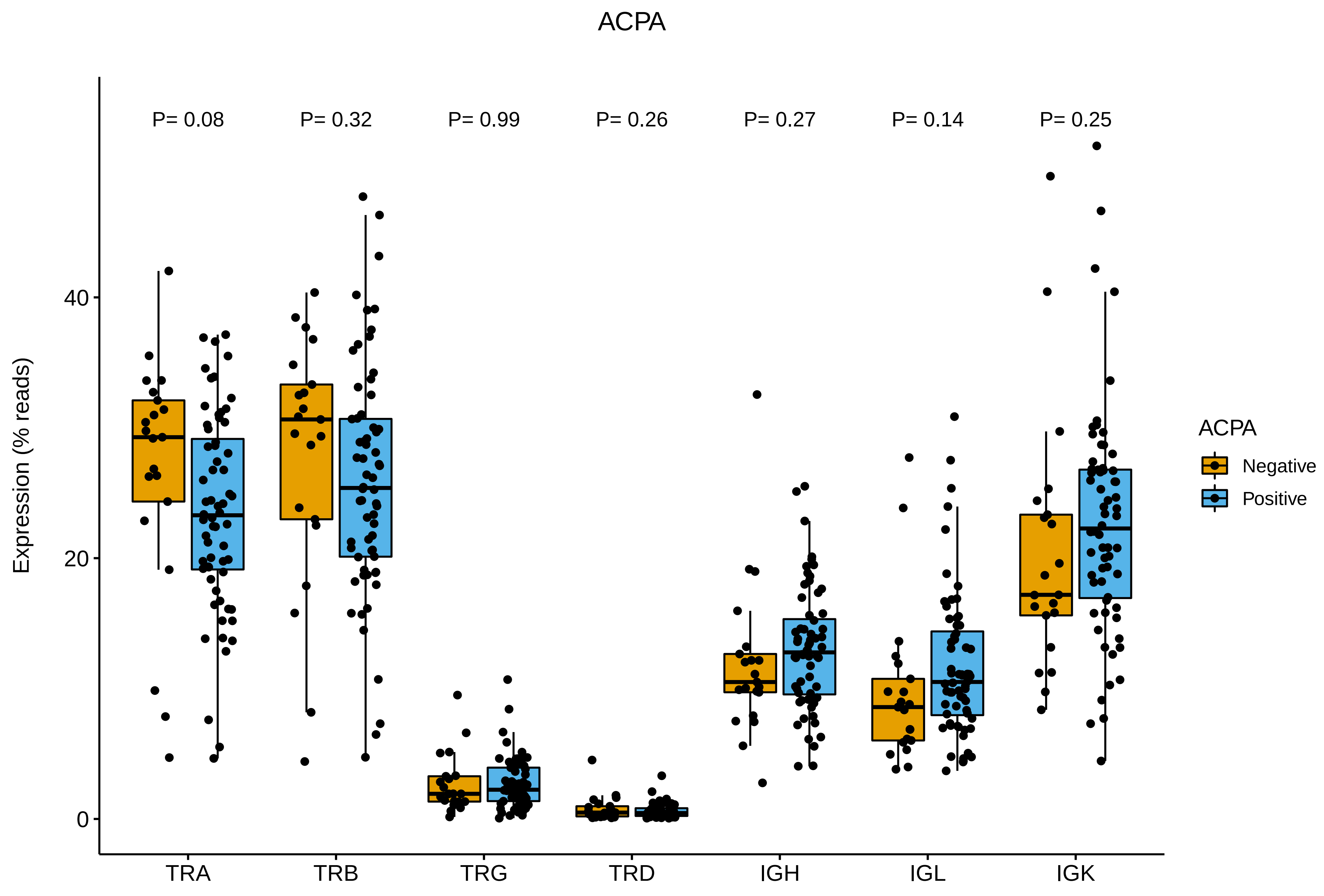

ACPA
P= 0.08
P= 0.32
P= 0.99
P= 0.26
P= 0.27
P= 0.14
P= 0.25
Expression (% reads)
0
20
40
TRA
TRB
TRG
TRD
IGH
IGL
IGK
ACPA
Negative
Positive

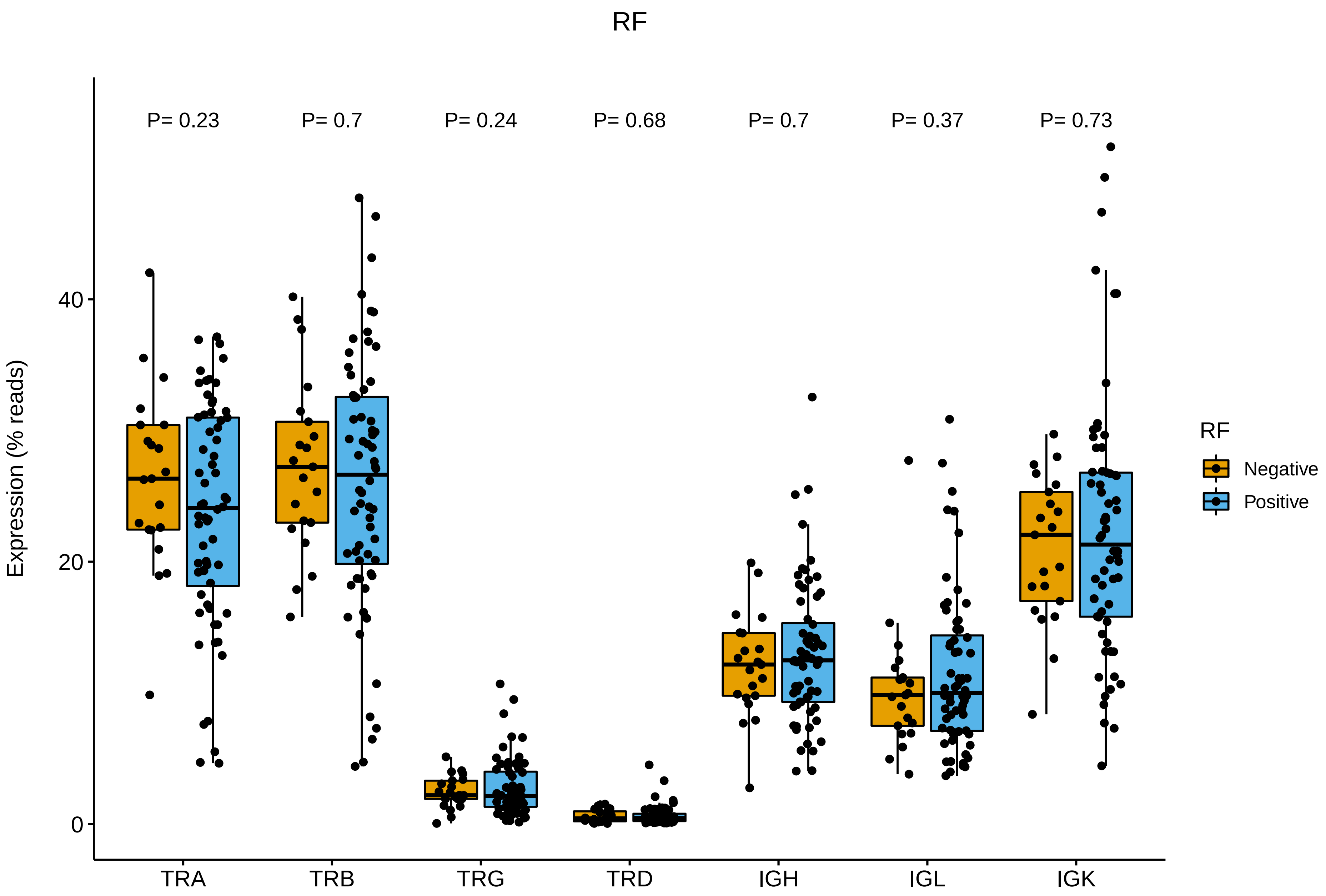

RF
P= 0.23
P= 0.7
P= 0.24
P= 0.68
P= 0.7
P= 0.37
P= 0.73
Expression (% reads)
0
20
40
TRA
TRB
TRG
TRD
IGH
IGL
IGK
RF
Negative
Positive

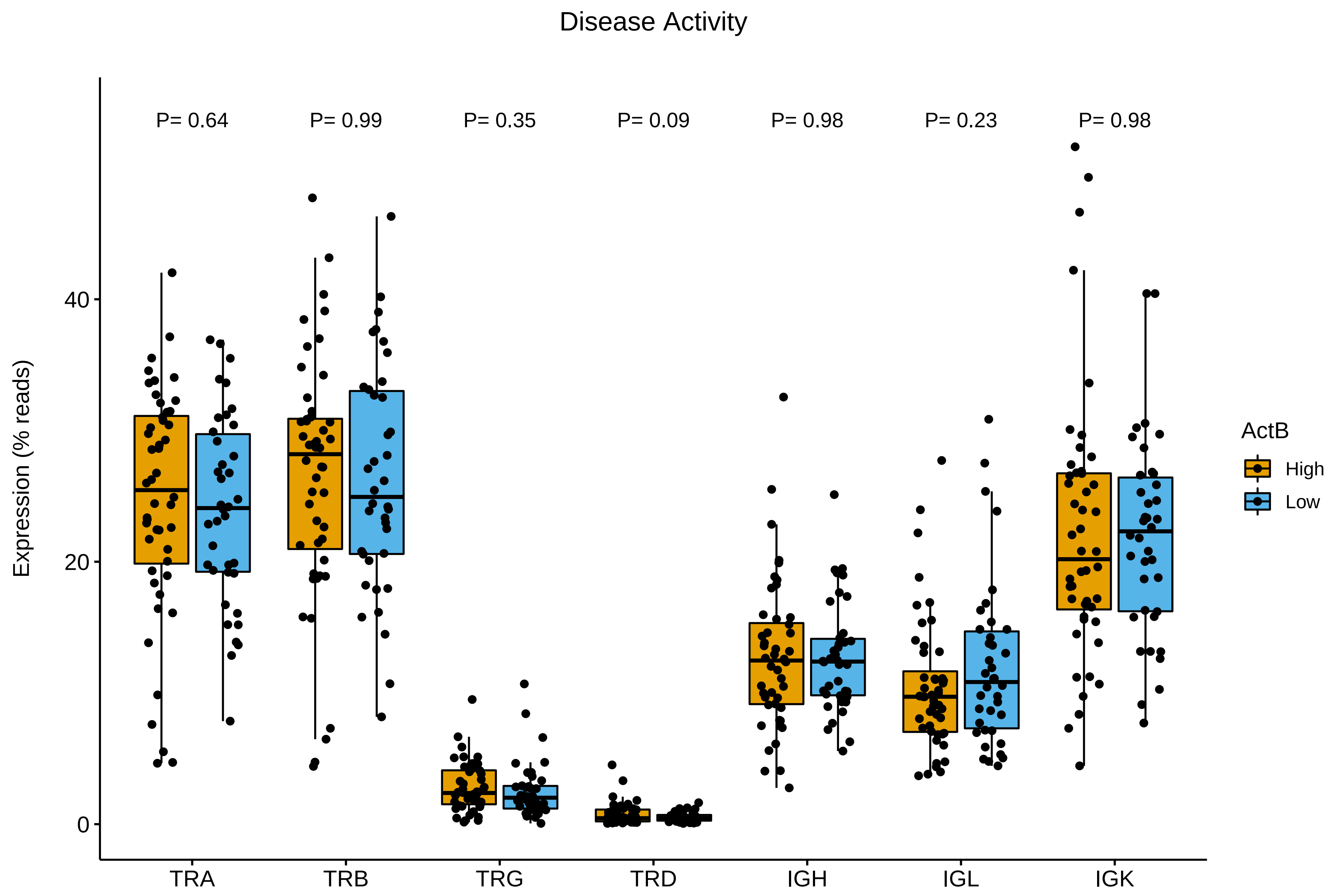

Disease Activity
P= 0.64
P= 0.99
P= 0.35
P= 0.09
P= 0.98
P= 0.23
P= 0.98
Expression (% reads)
0
20
40
TRA
TRB
TRG
TRD
IGH
IGL
IGK
ActB
High
Low
