# Supplementary material for: Seven-chain adaptive immune receptor repertoire analysis in rheumatoid arthritis reveals novel features associated with disease and clinically relevant phenotypes
Source: Genome Biol. 2024 Mar 11;25:68. doi: 10.1186/s13059-024-03210-0 (PMC10926600; doi:10.1186/s13059-024-03210-0)
Supplement: Supplementary file 18 — Additional file 18: Figure S4. Graphical representation of the significant associations between IGL/IGK clones and rheumatoid arthritis. Significant associations detected by the Hurdle or continuous models are represented using violin plots, where the clone expression is plotted separately for each phenotype. Significant associations detected by the discrete model are represented using bar plots, where the number of individuals having the clone are plotted separately for each phenotype. For all significant associations (FDR<0.05), the clone expression is also plotted against the standardized clone detection rate. Abbreviations: CDR, clone detection rate; CPM, count per million on the logarithmic scale; Cont, continuous model; CTRL, healthy individuals; Disc, discrete model; P, p-value; RA, rheumatoid arthritis. [file 13059_2024_3210_MOESM18_ESM.pdf]

Fig S4. Graphical representation of the significant associations between IGL/IGK clones and rheumatoid arthritis. Significant associations detected by the Hurdle or continuous models are represented using violin plots, where the clone expression is plotted separately for each phenotype. Significant associations detected by the discrete model are represented using bar plots, where the number of individuals having the clone are plotted separately for each phenotype. For all significant associations (FDR<0.05), the clone expression is also plotted against the standardized clone detection rate. Abbreviations: CDR, clone detection rate; CPM, count per million on the logarithmic scale; Cont, continuous model; CTRL, healthy individuals; Disc, discrete model; P, p-value; RA, rheumatoid arthritis.

CAAWDDSLSGPNWVF from IGL chain significant in Hurdle model

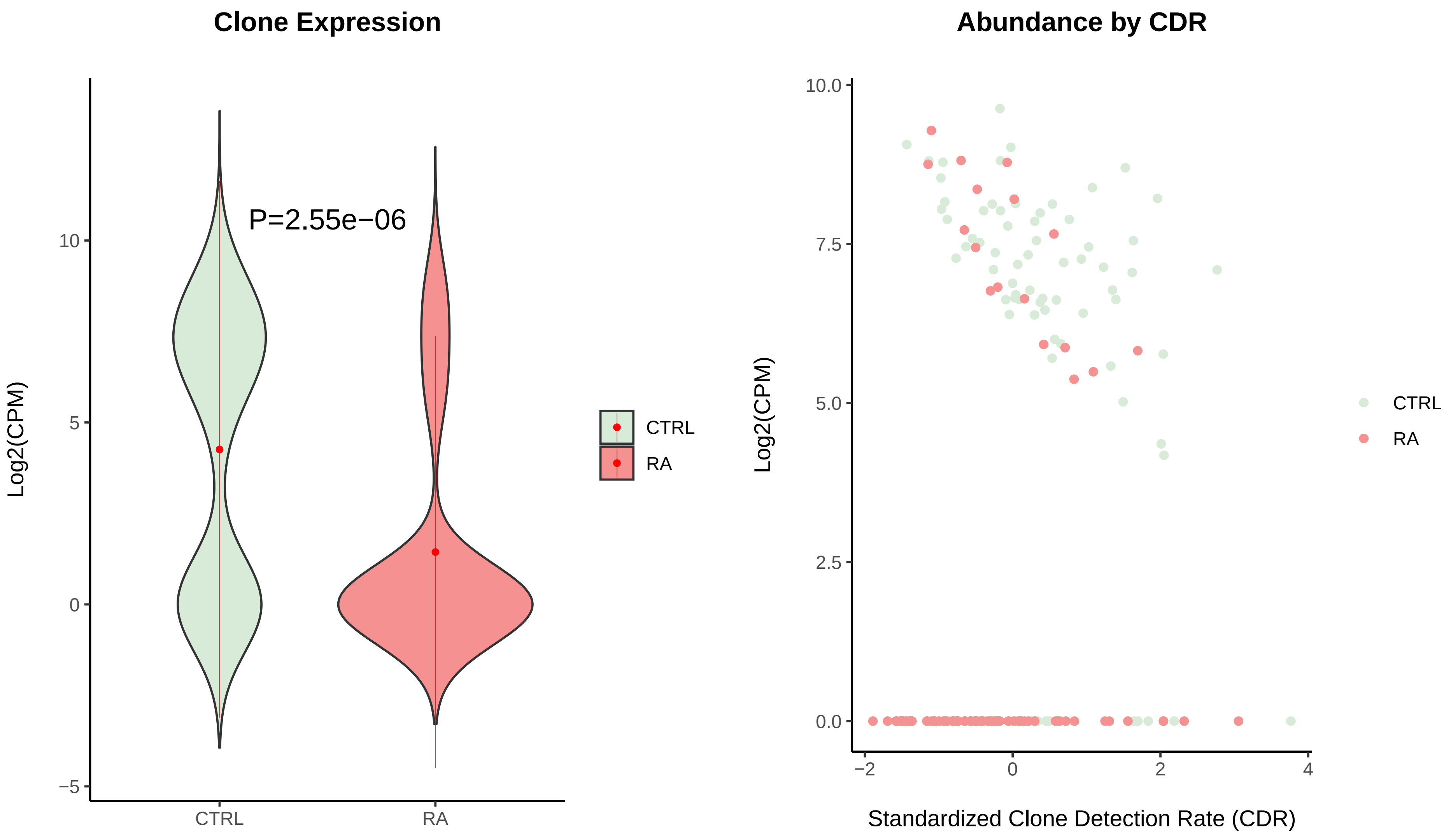

CAAWDDSLSGPVF from IGL chain significant in Hurdle model

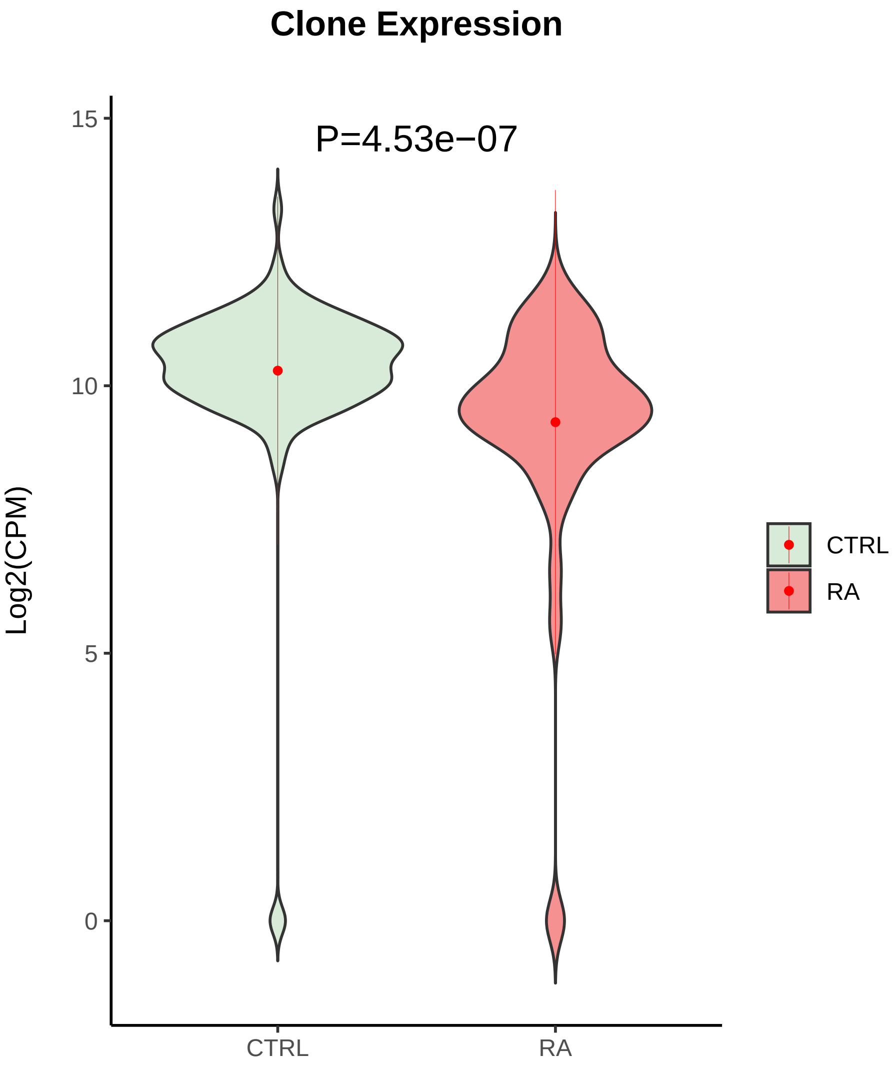

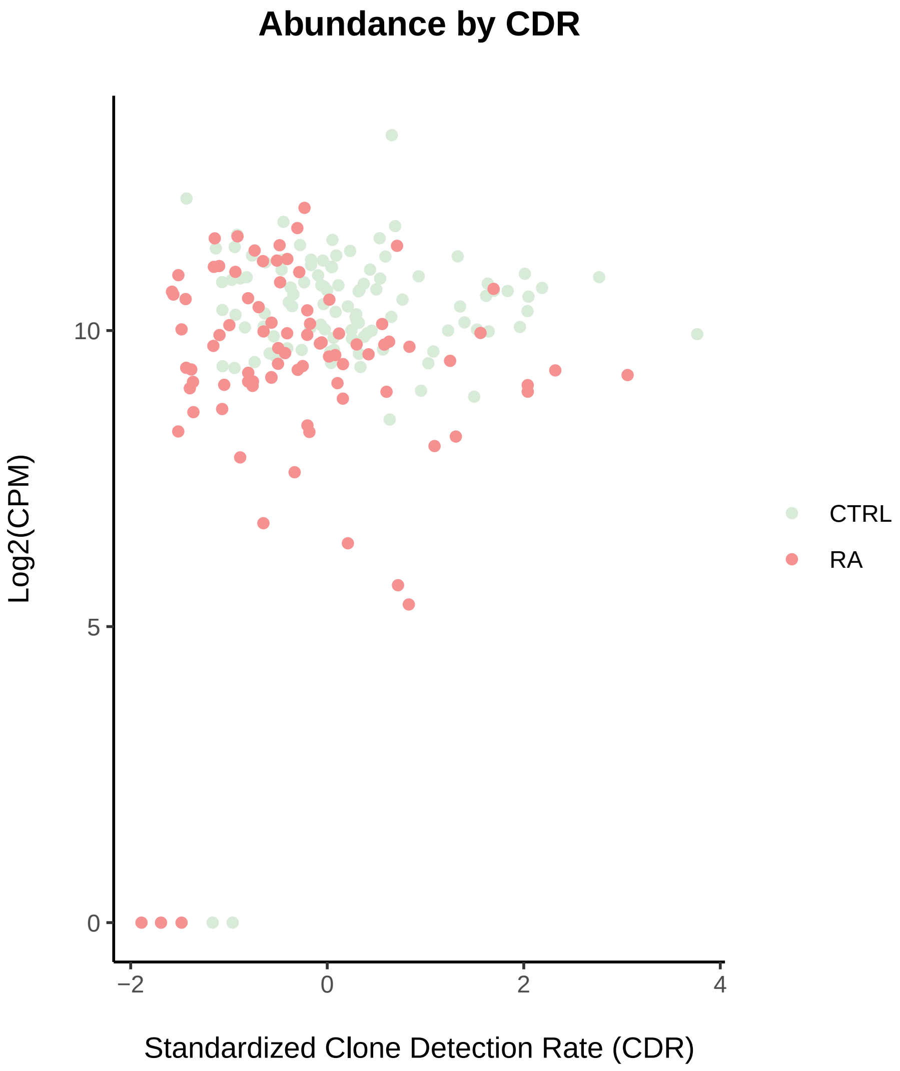

CAAWDDSLSGWVF from IGL chain significant in Hurdle model

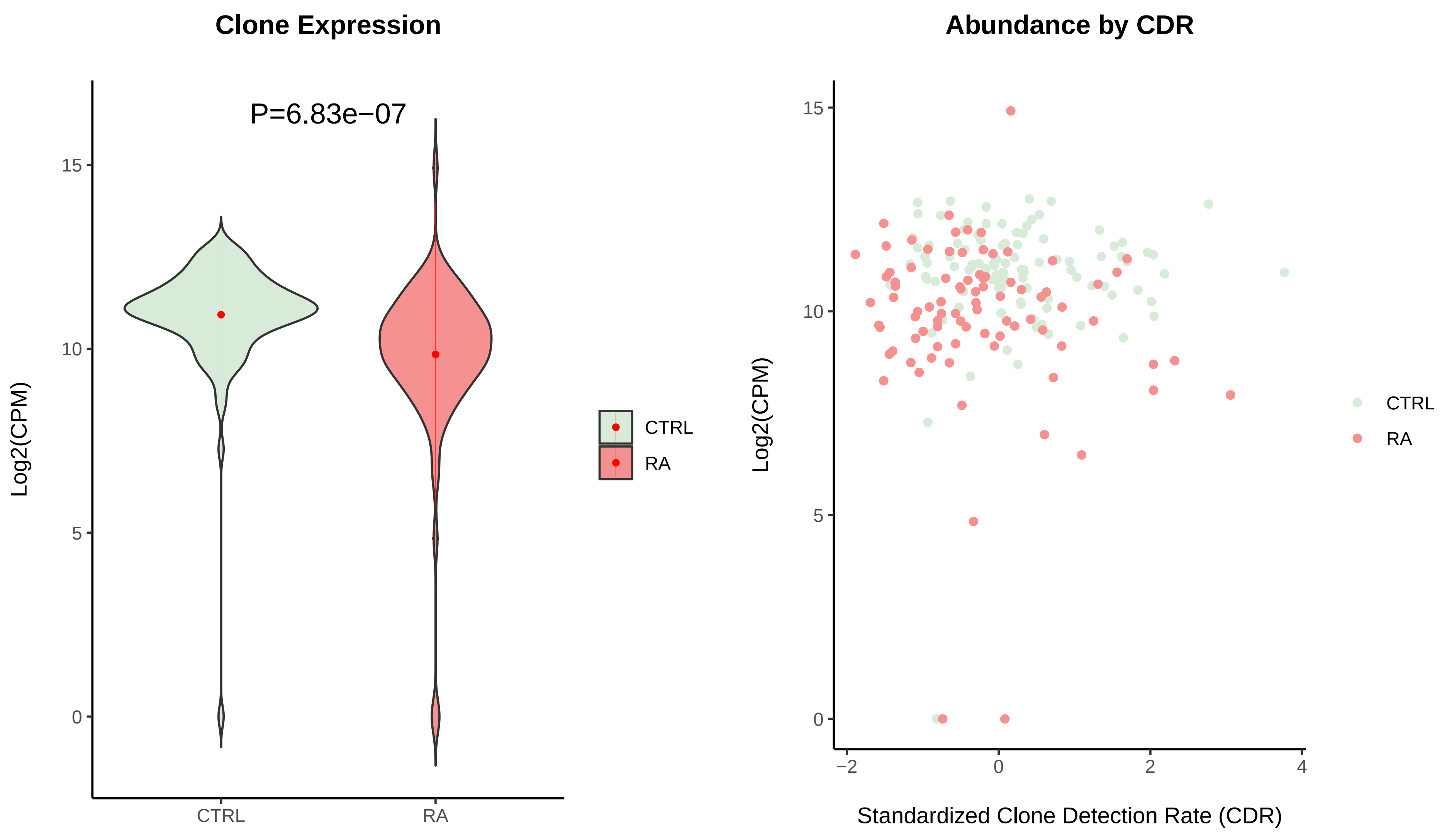

CGADHGSGSNFVKVF from IGL chain significant in Hurdle model

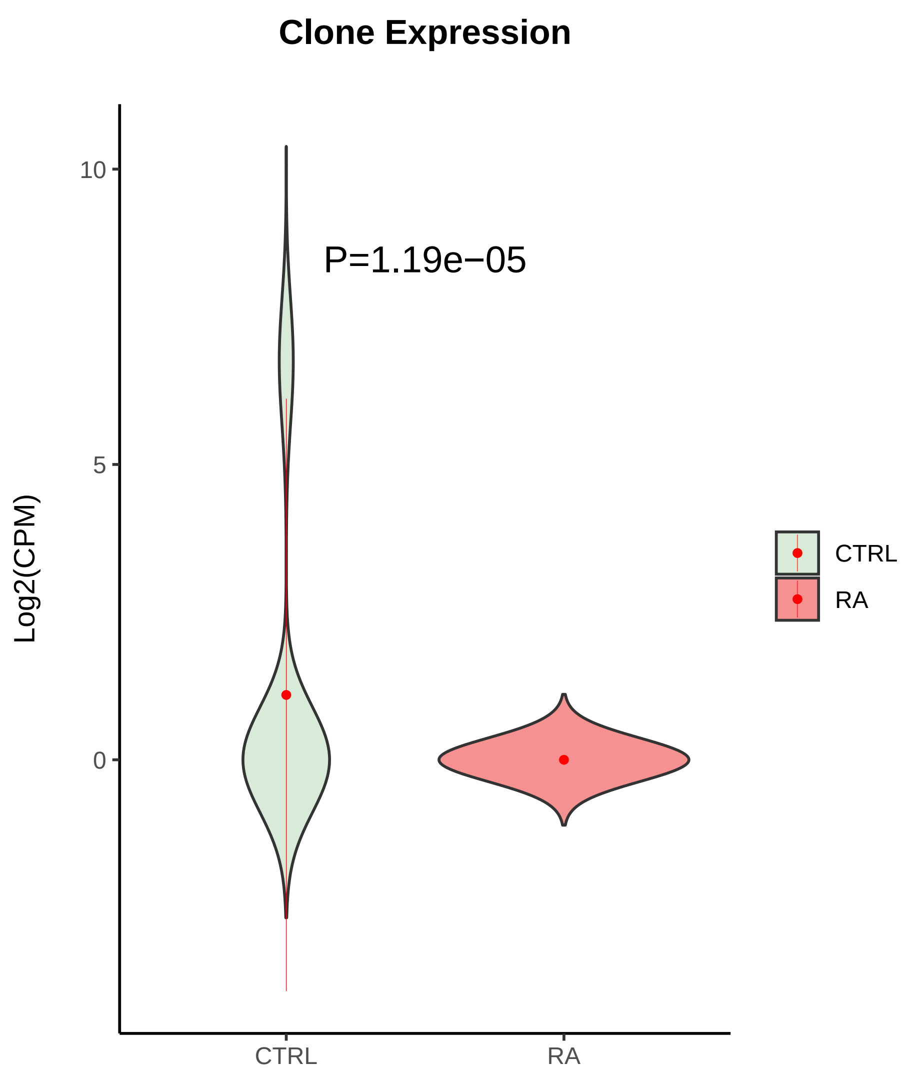

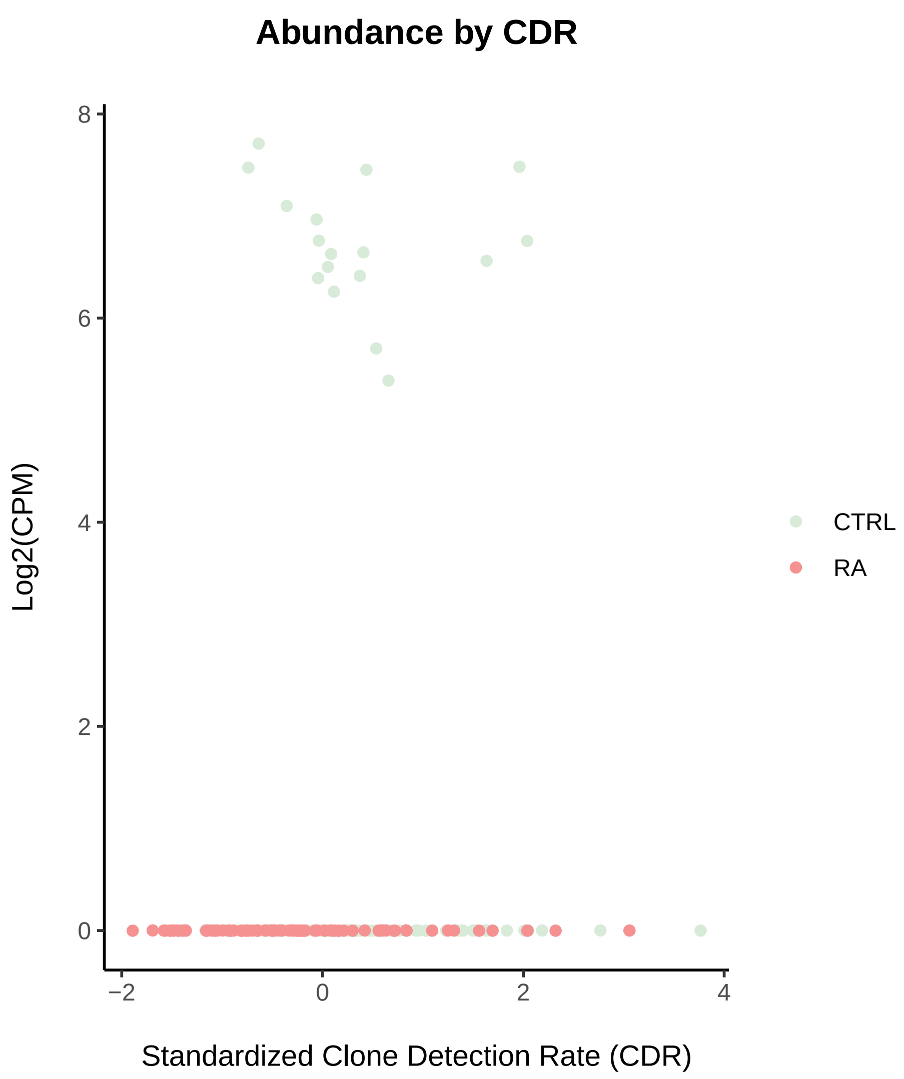

CGADHGSGSNFVWVF from IGL chain significant in Hurdle model

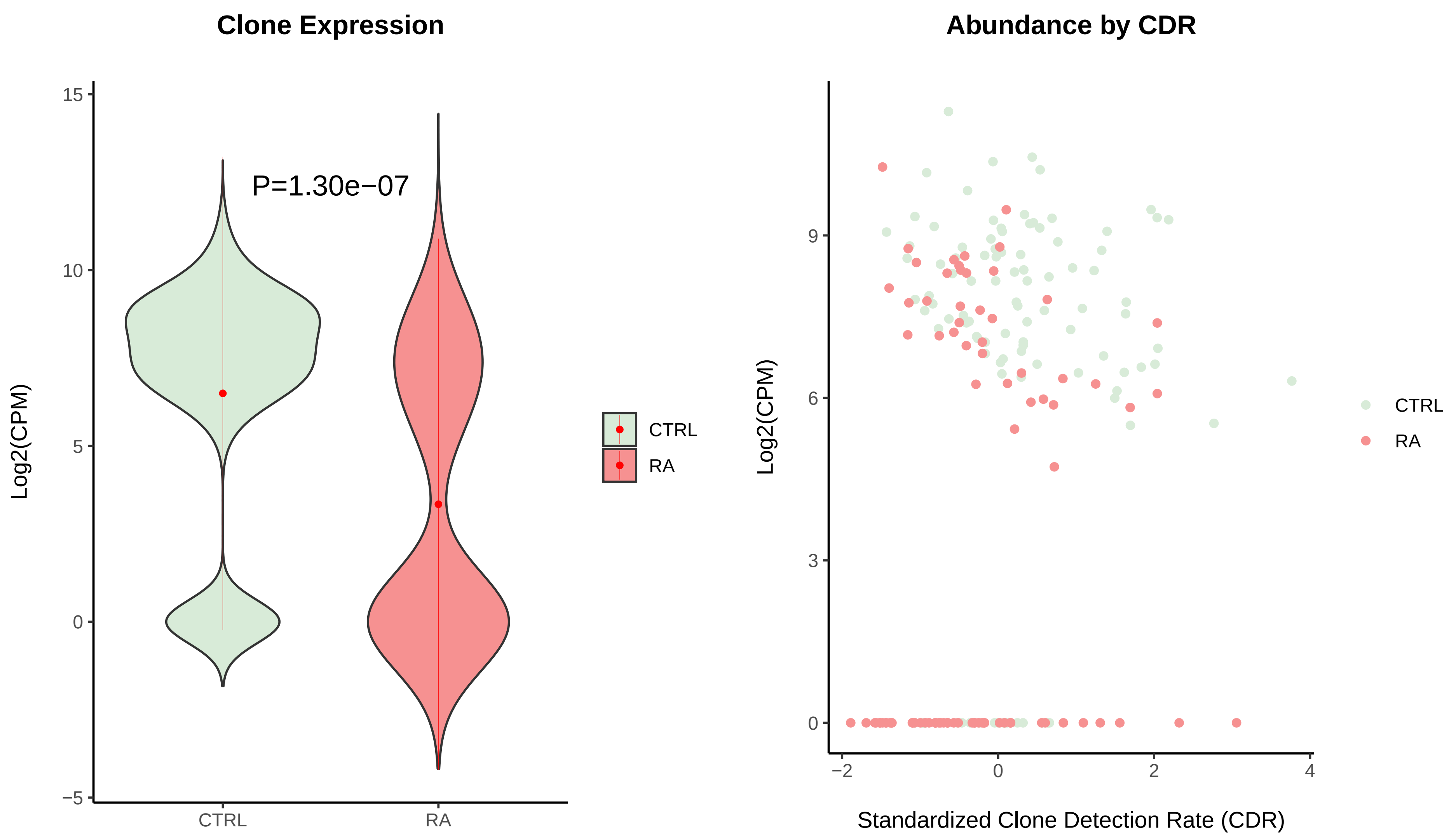

CGADHGSGSNFVYVF from IGL chain significant in Hurdle model

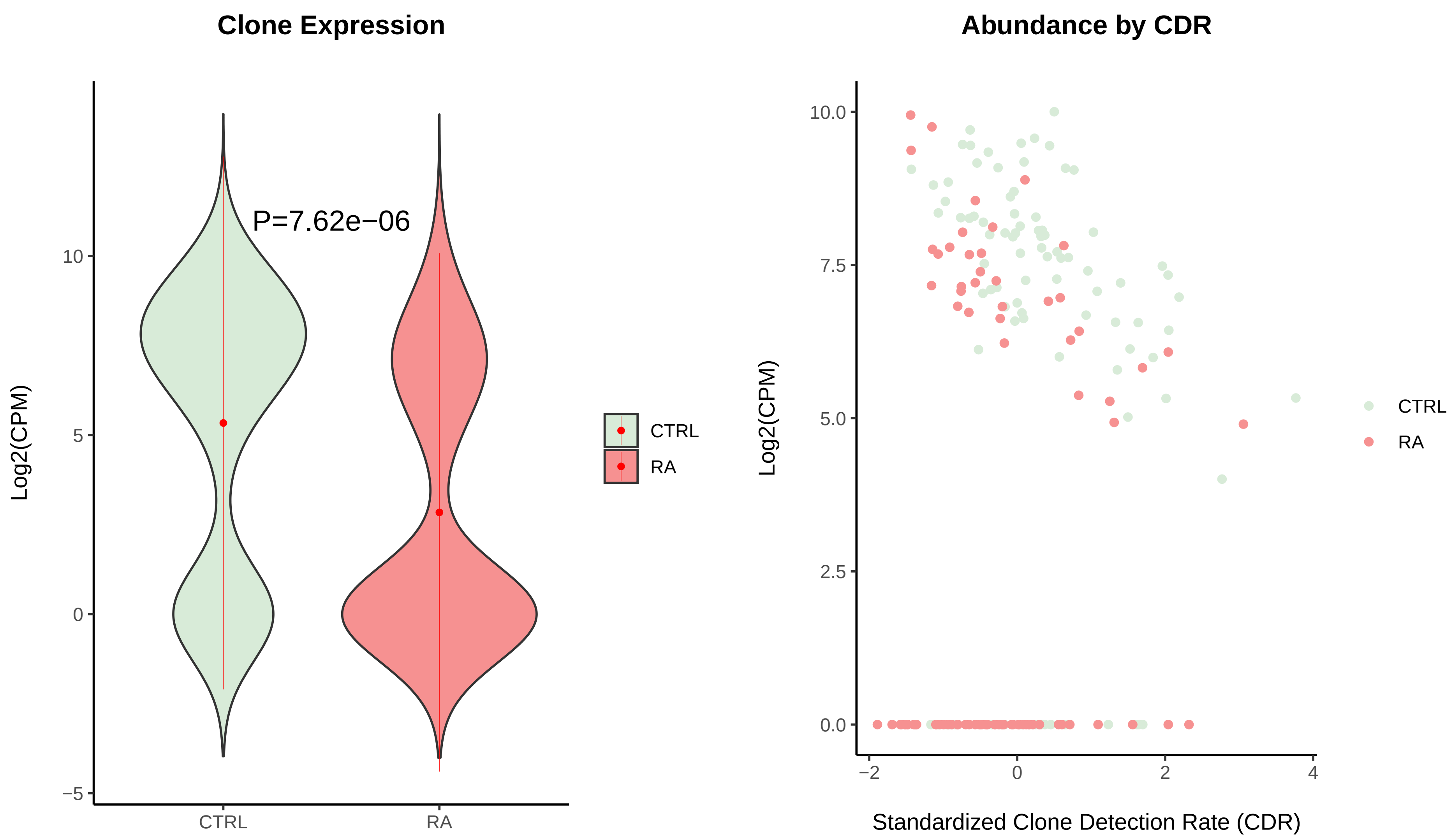

CQSYDSSLYVVF from IGL chain significant in Hurdle model

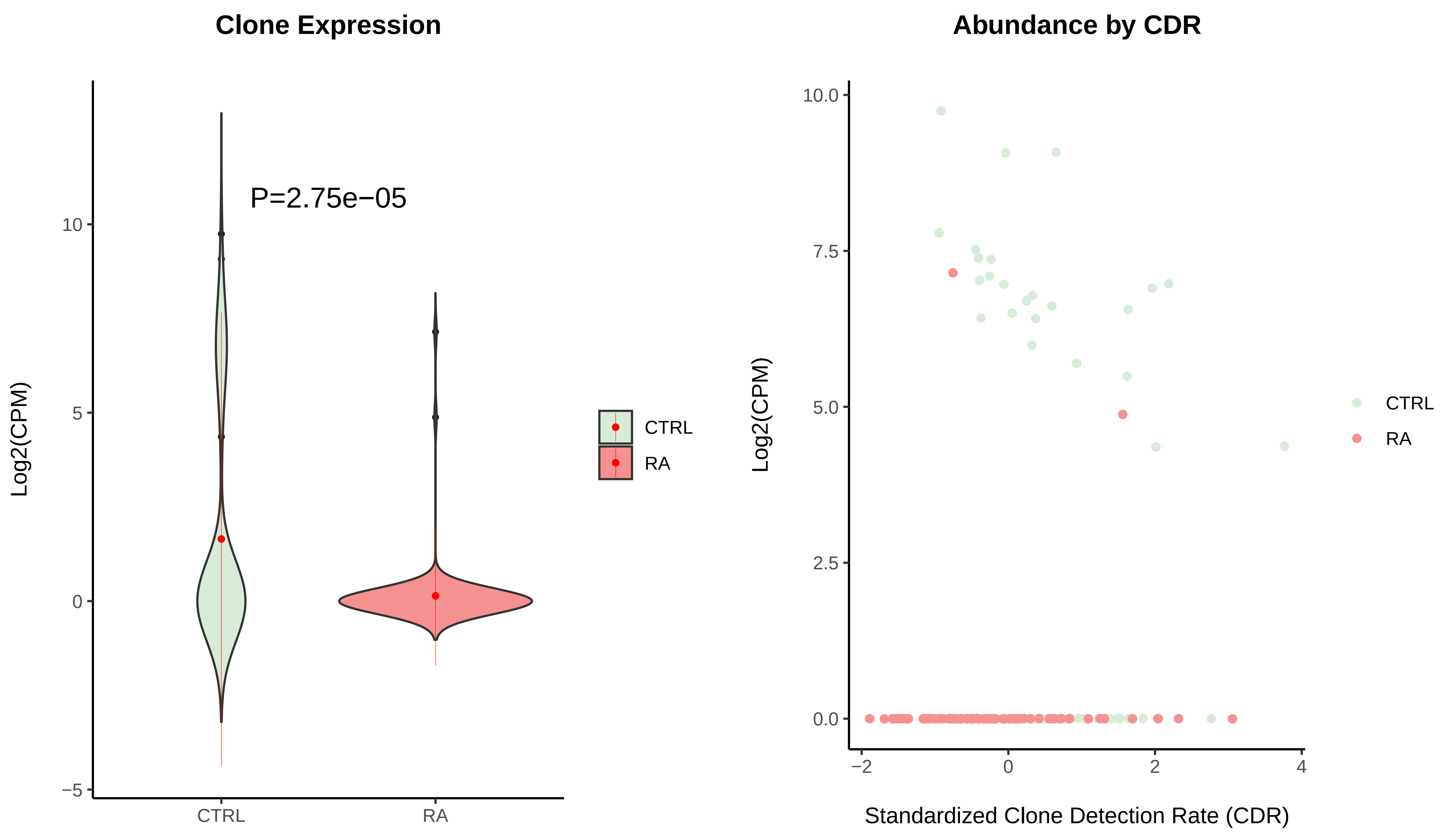

CQVWDSSSDHHVVF from IGL chain significant in Hurdle model

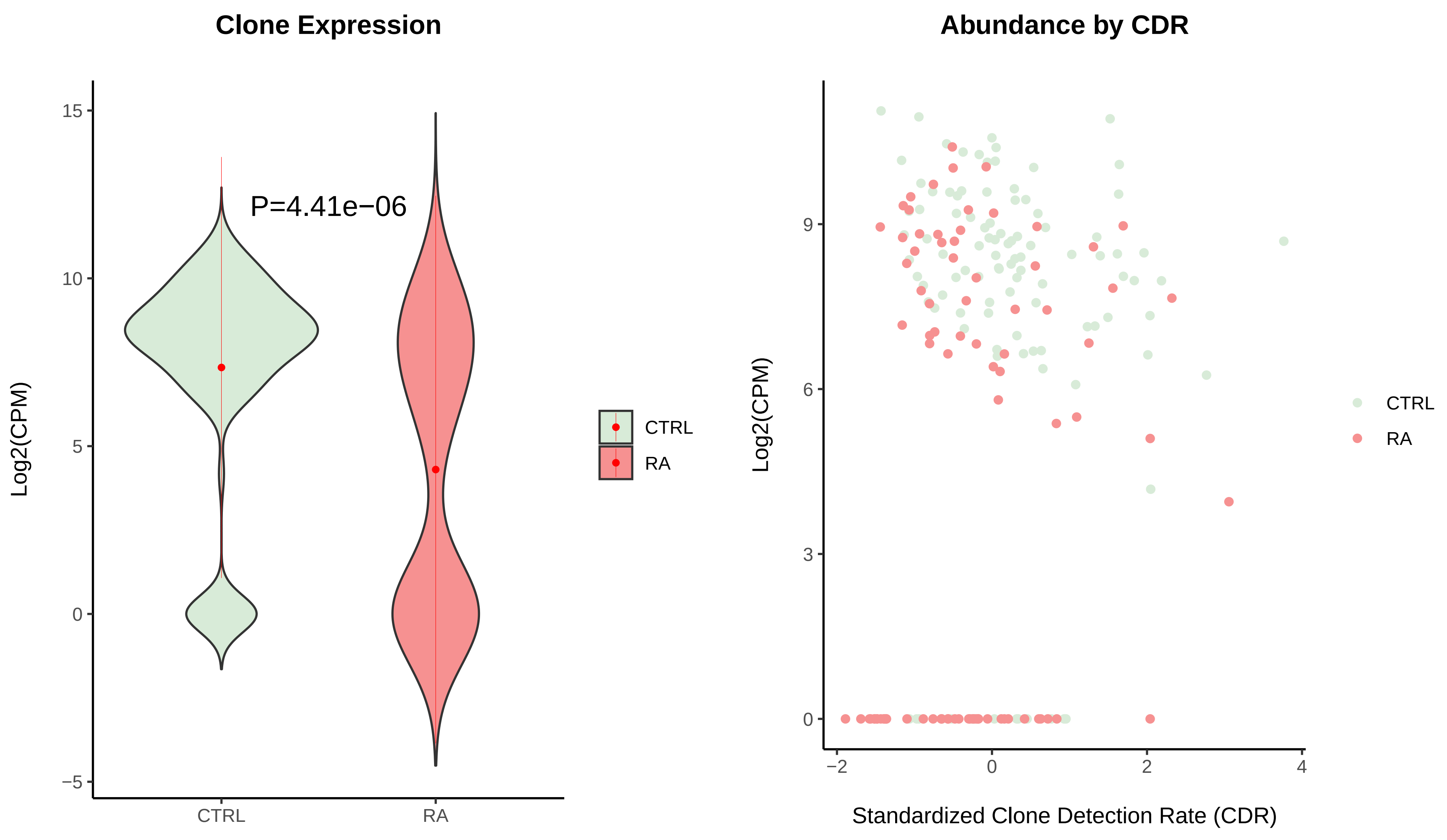

CQVWDSSSDHLVVF from IGL chain significant in Hurdle model

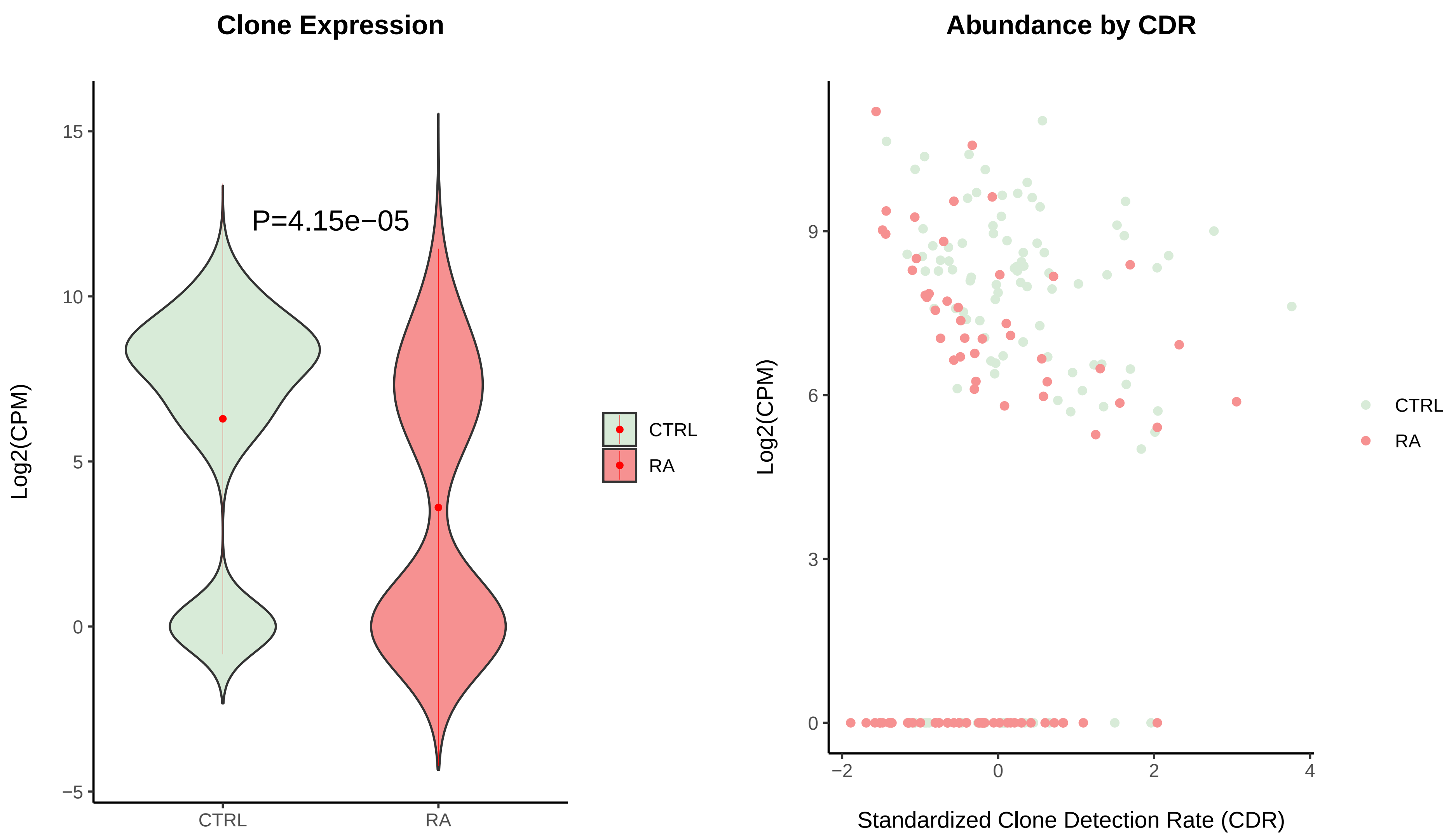

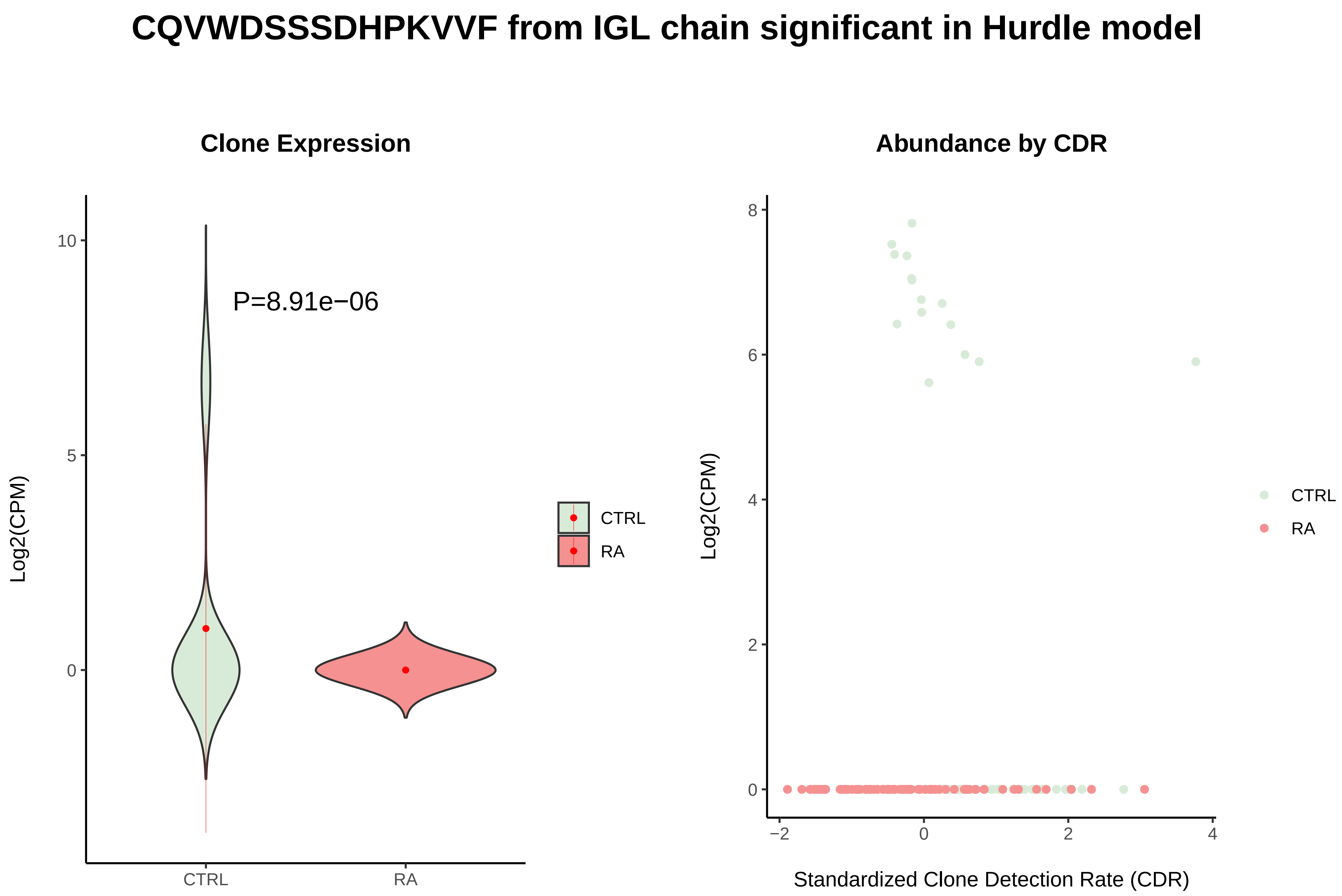

CQVWDSSSDHPKVVF from IGL chain significant in Hurdle model
Clone Expression
P=8.91e−06
Log2(CPM)
10
5
0
CTRL
RA
CTRL
RA
Abundance by CDR
Log2(CPM)
8
6
4
2
0
−2
0
2
4
Standardized Clone Detection Rate (CDR)
CTRL
RA

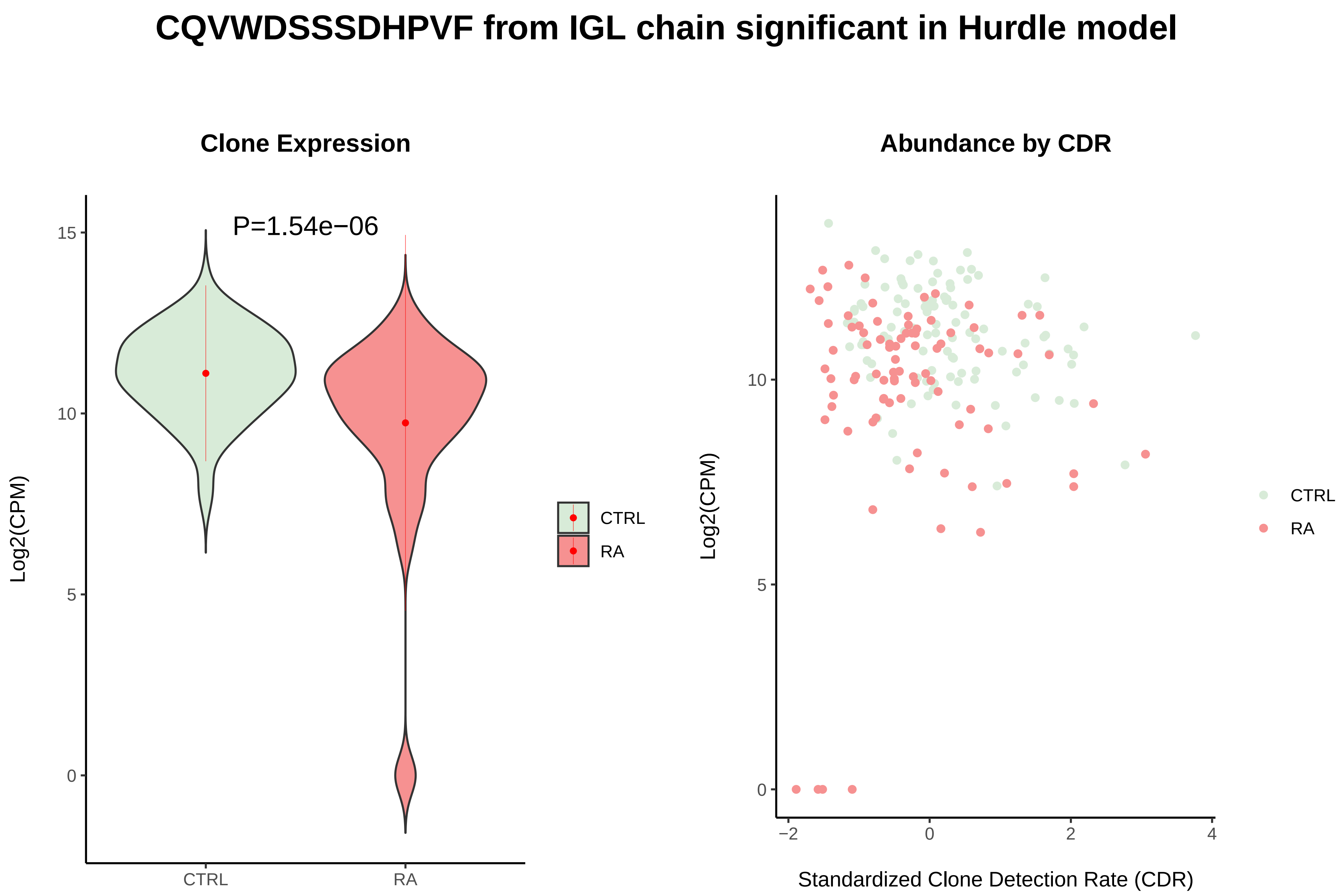

CQVWDSSSDHPVF from IGL chain significant in Hurdle model
Clone Expression
P=1.54e−06
15
10
5
0
Log2(CPM)
CTRL
RA
CTRL
RA
Abundance by CDR
10
5
0
Log2(CPM)
−2
0
2
4
Standardized Clone Detection Rate (CDR)
CTRL
RA

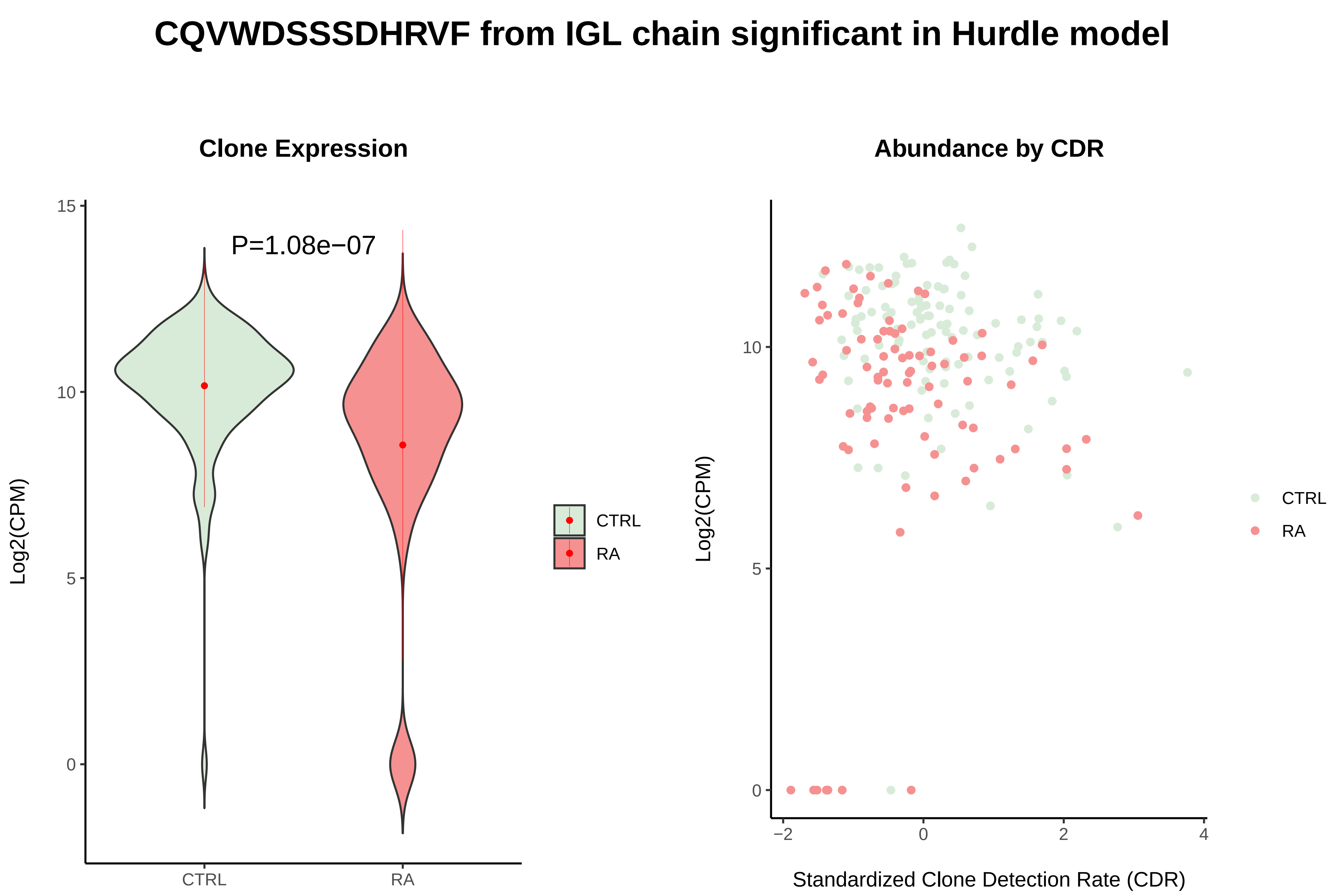
CQVWDSSSDHRVF from IGL chain significant in Hurdle model
Clone Expression
P=1.08e−07
Log2(CPM)
15
10
5
0
CTRL
RA
Abundance by CDR
Log2(CPM)
10
5
0
−2
0
2
4
Standardized Clone Detection Rate (CDR)
CTRL
RA

CQVWDSSSDHVVF from IGL chain significant in Hurdle model

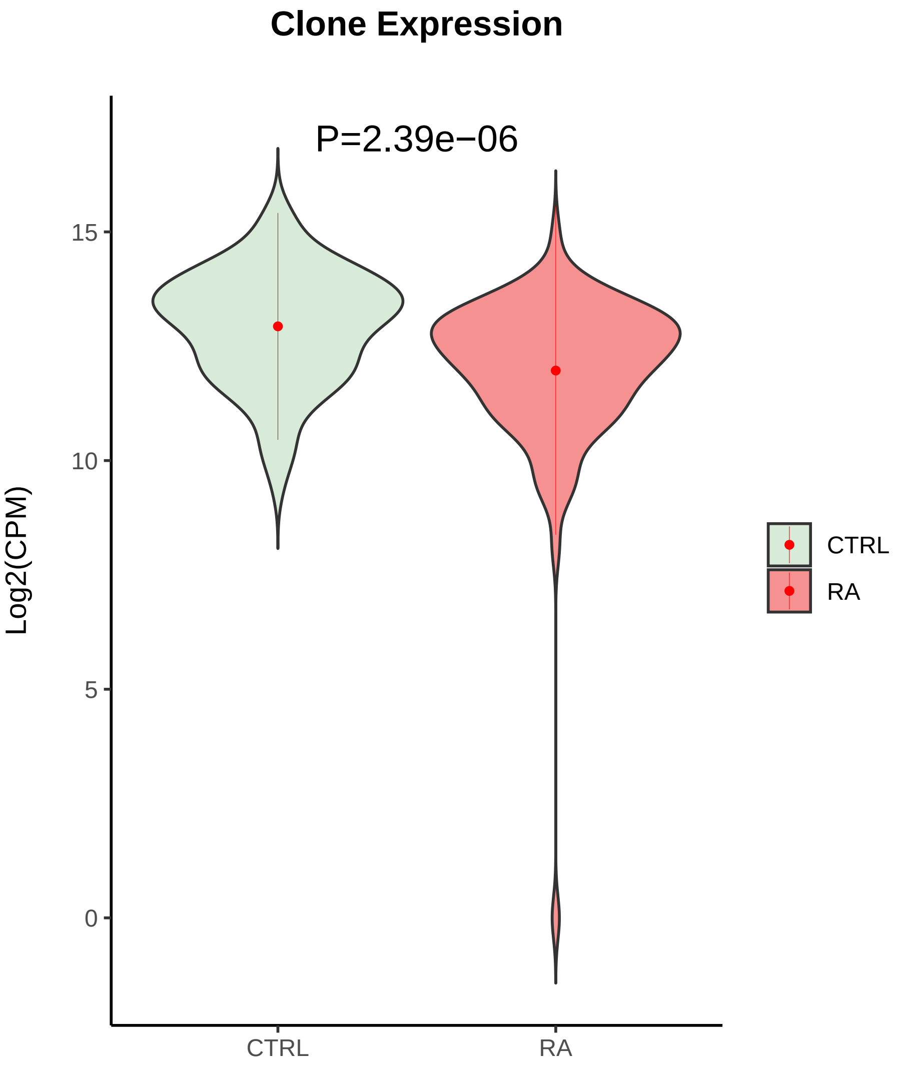

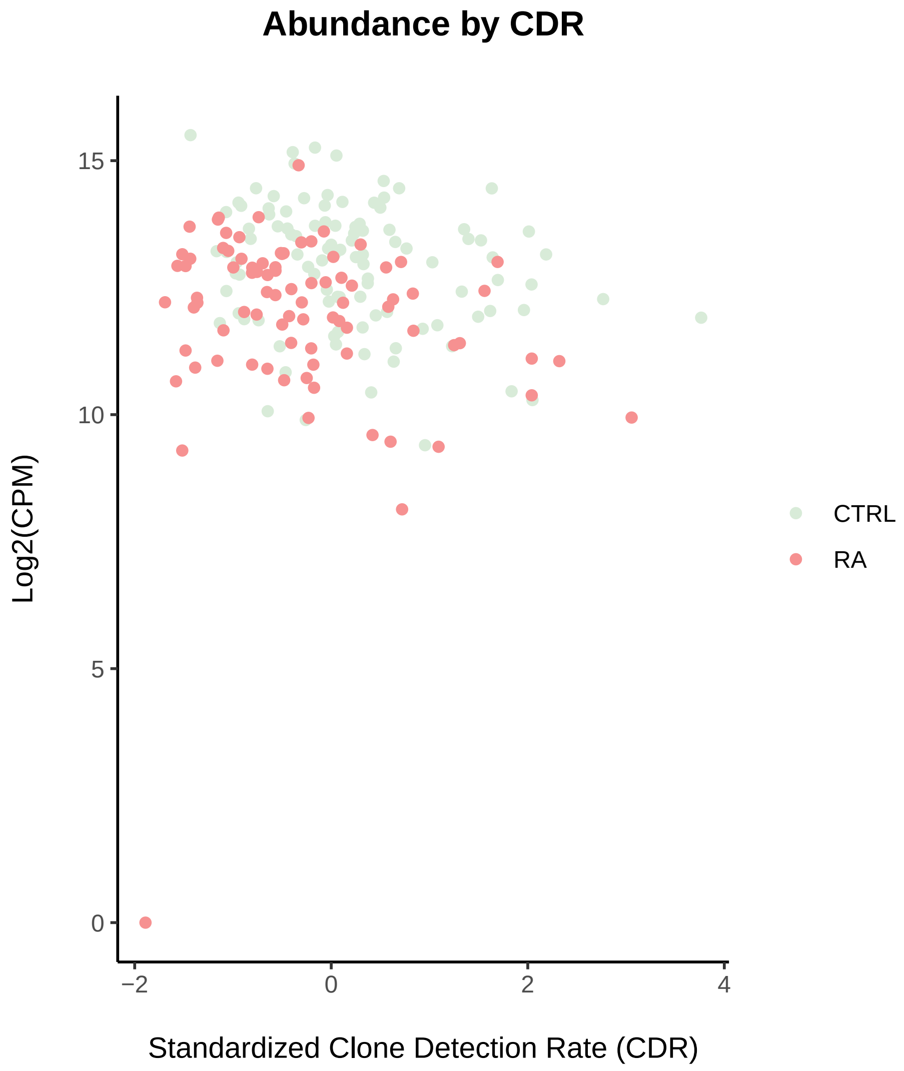

CQVWDSSSDHWVF from IGL chain significant in Hurdle model

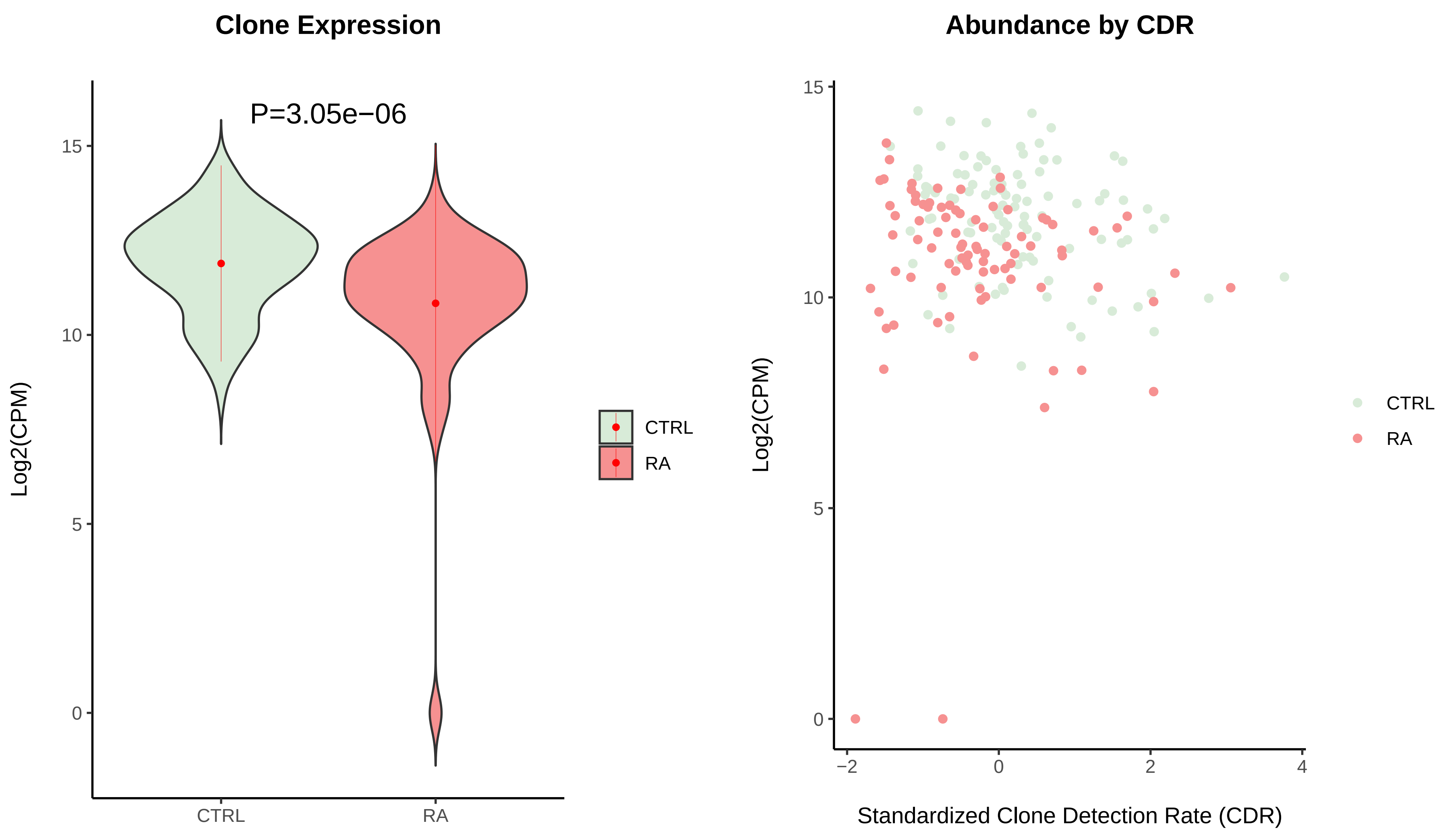

CQVWDSSSDHYVF from IGL chain significant in Hurdle model

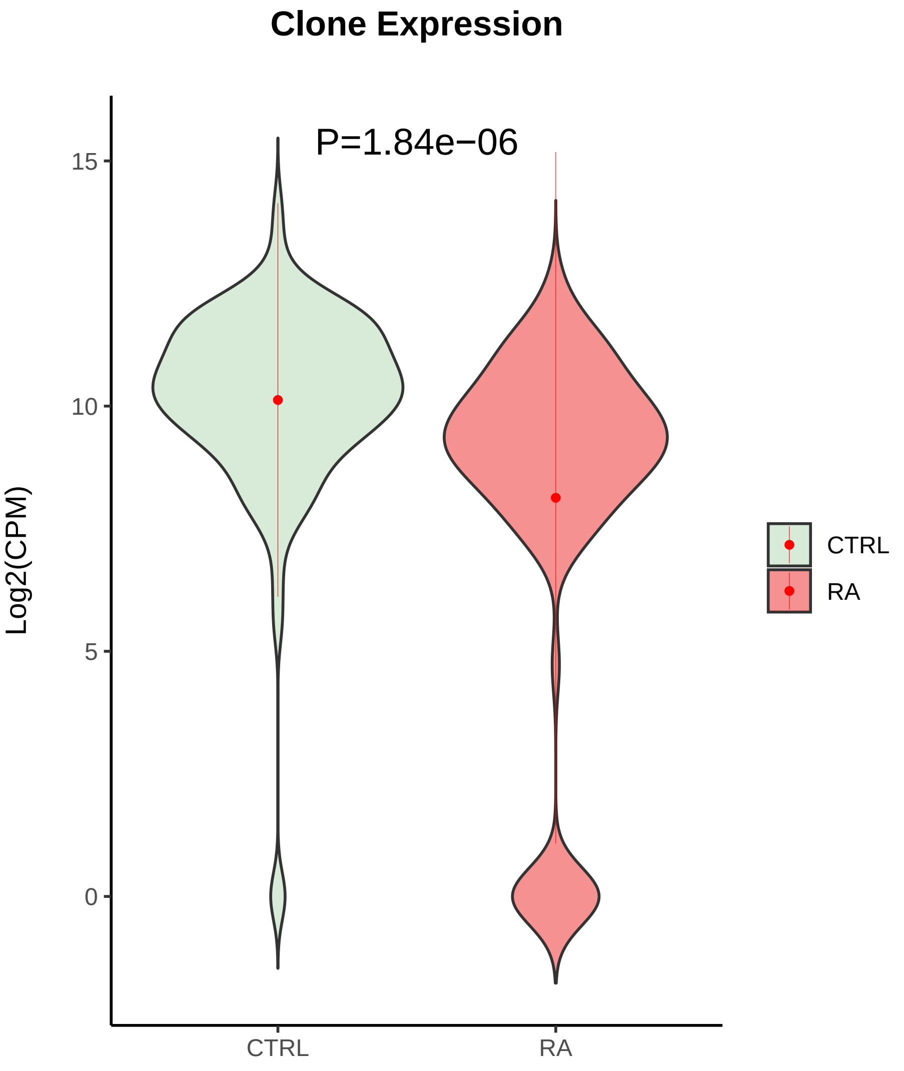

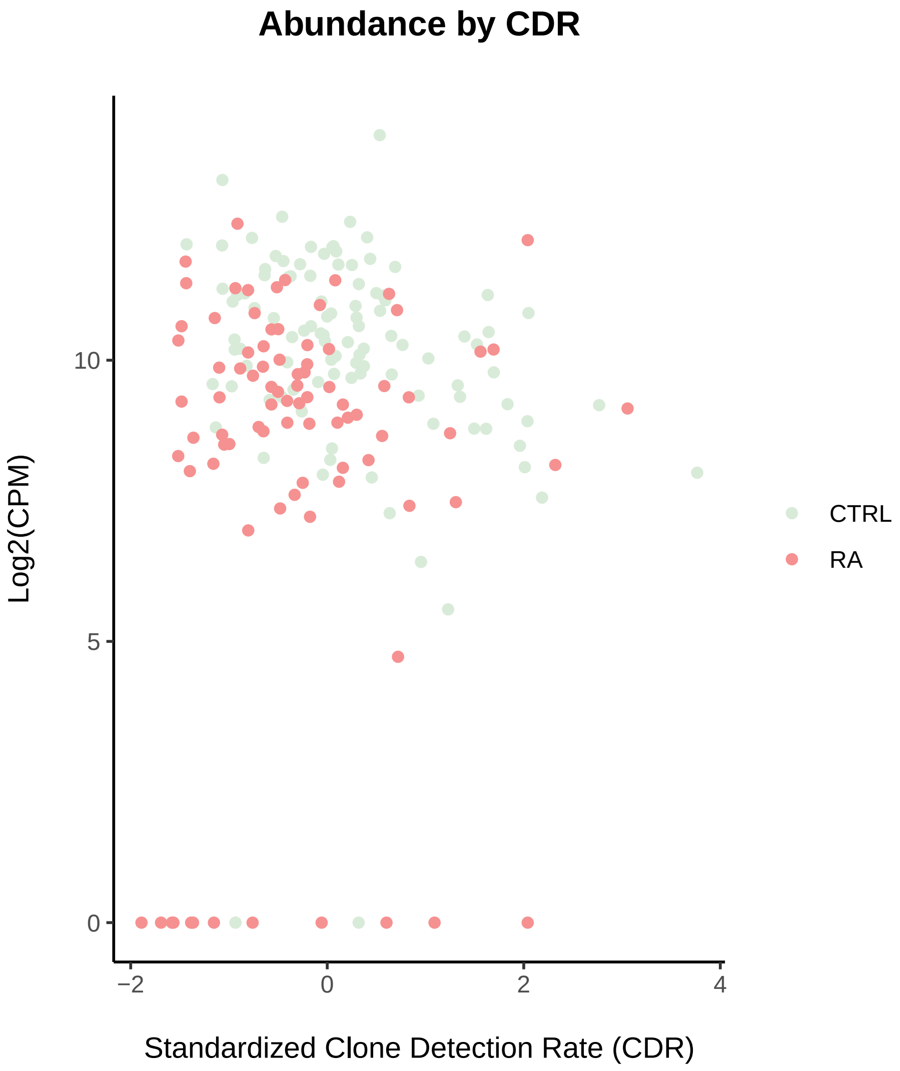

CQVWDSSSDQEVF from IGL chain significant in Hurdle model

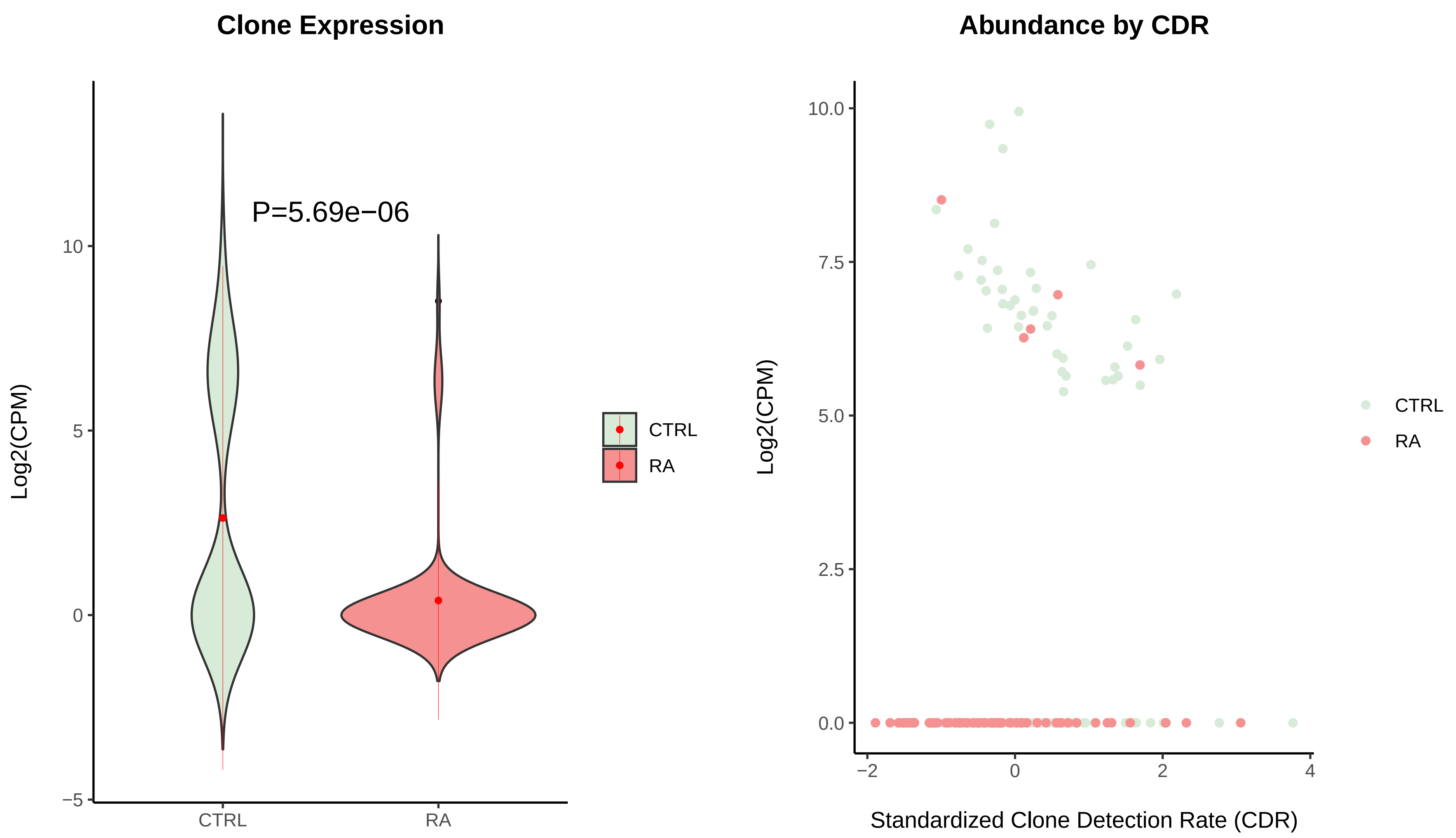

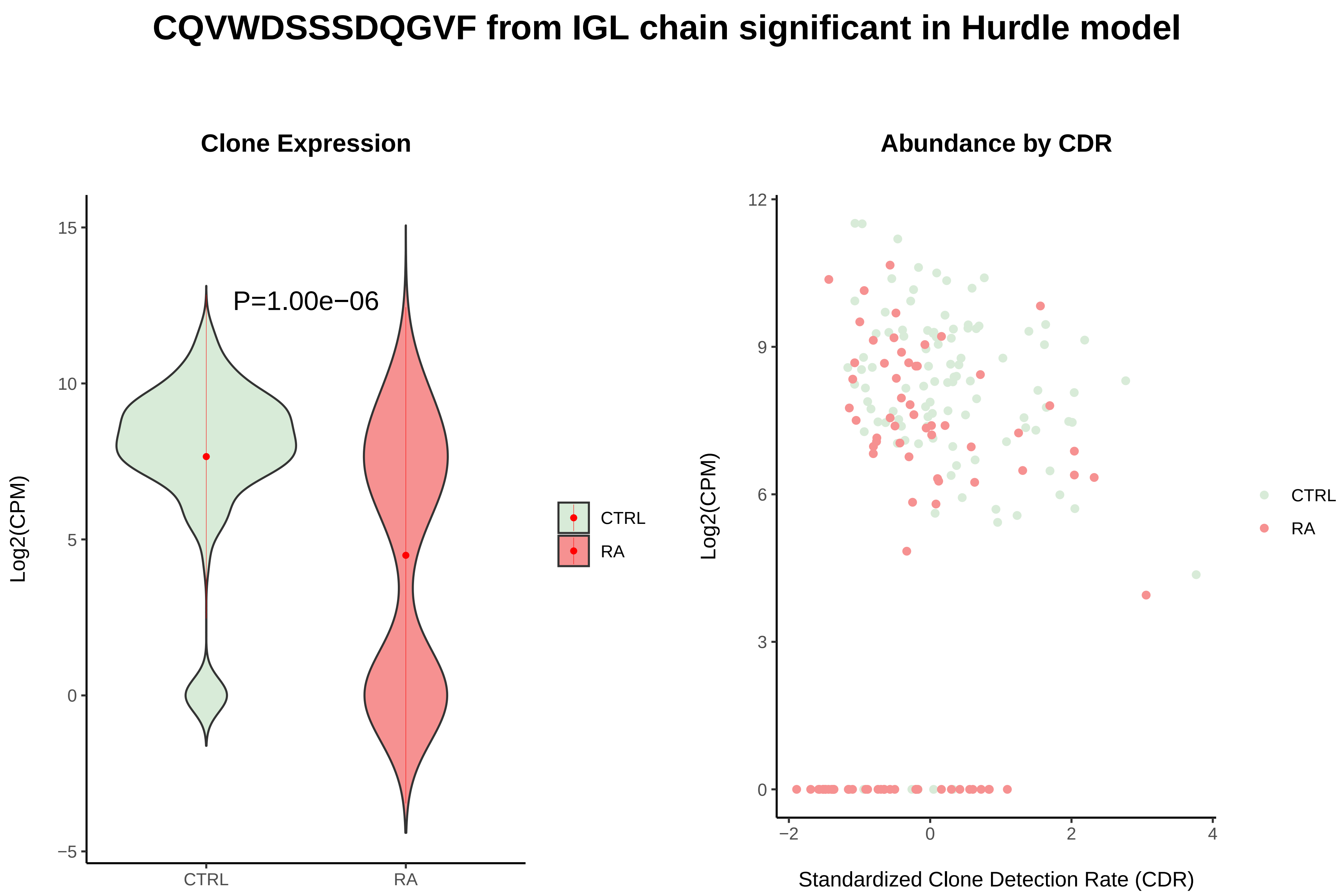

CQVWDSSSDQGVF from IGL chain significant in Hurdle model
Clone Expression
P=1.00e−06
Log2(CPM)
15
10
5
0
−5
CTRL
RA
CTRL
RA
Abundance by CDR
Log2(CPM)
12
9
6
3
0
−2
0
2
4
Standardized Clone Detection Rate (CDR)
CTRL
RA

CQVWDSSSDRVF from IGL chain significant in Hurdle model

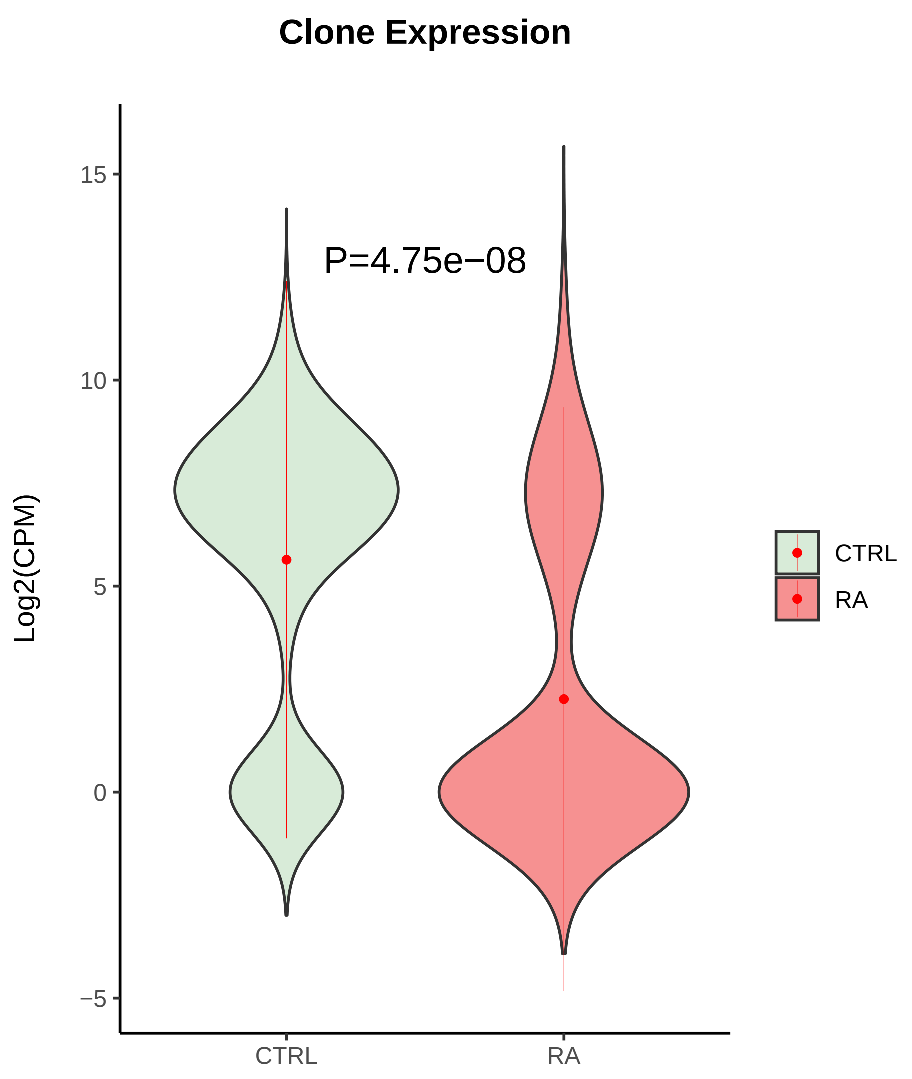

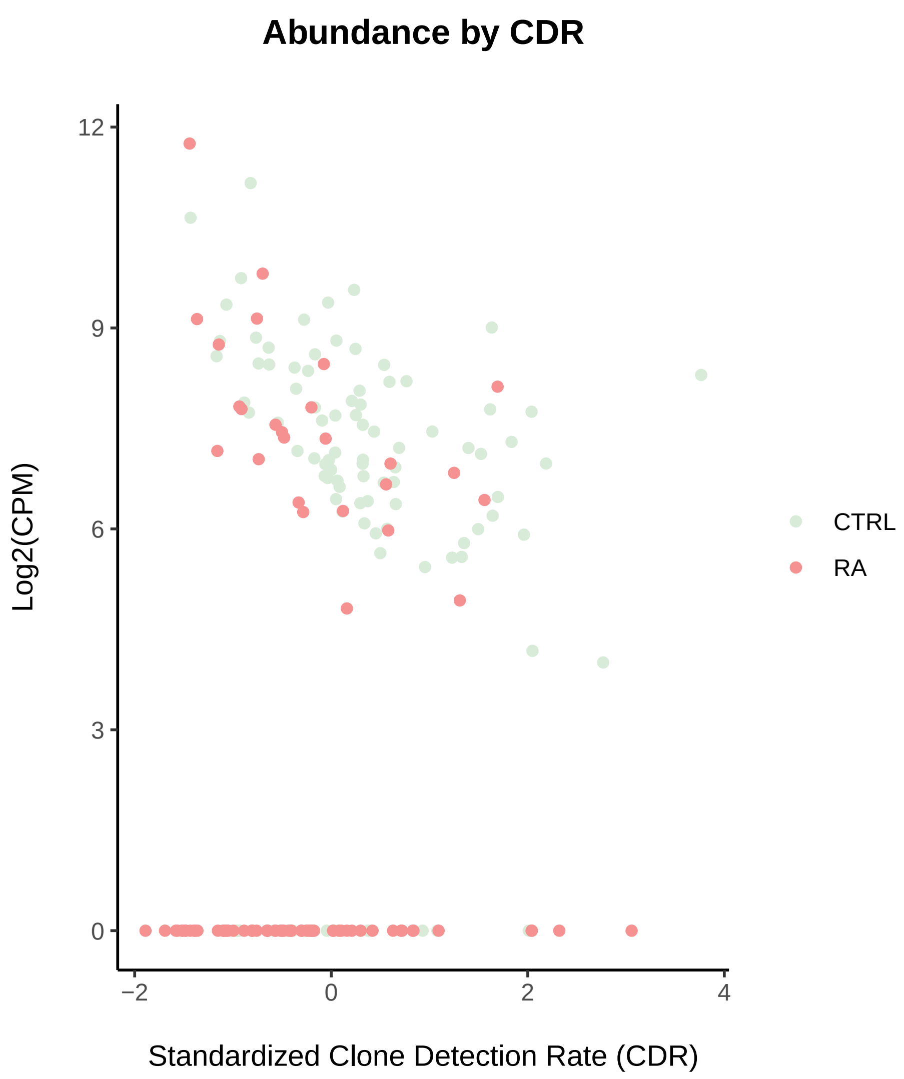

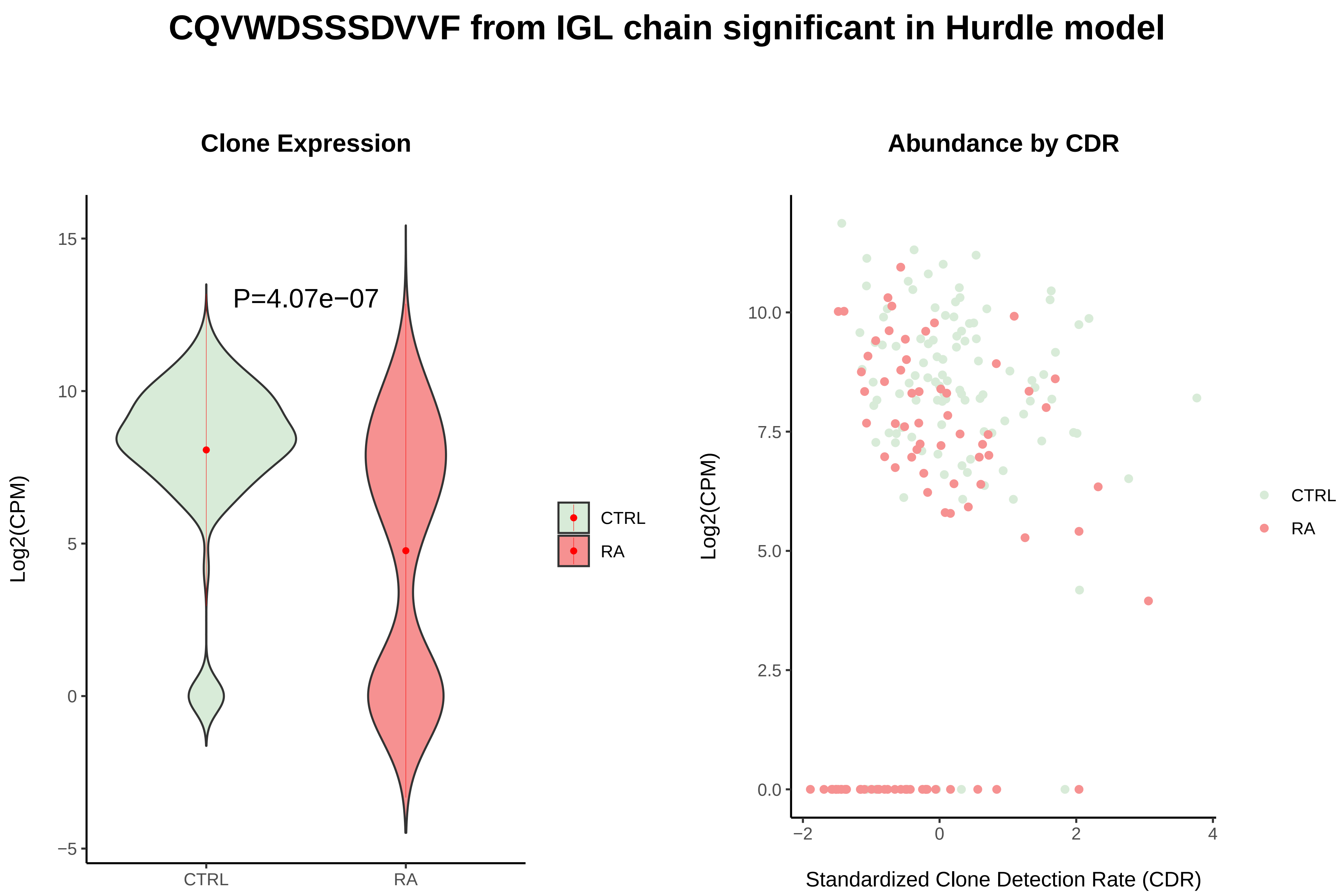
CQVWDSSSDVVF from IGL chain significant in Hurdle model
Clone Expression
Abundance by CDR
P=4.07e−07
Log2(CPM)
15
10
5
0
−5
CTRL
RA
10.0
7.5
5.0
2.5
0.0
−2
0
2
4
Standardized Clone Detection Rate (CDR)

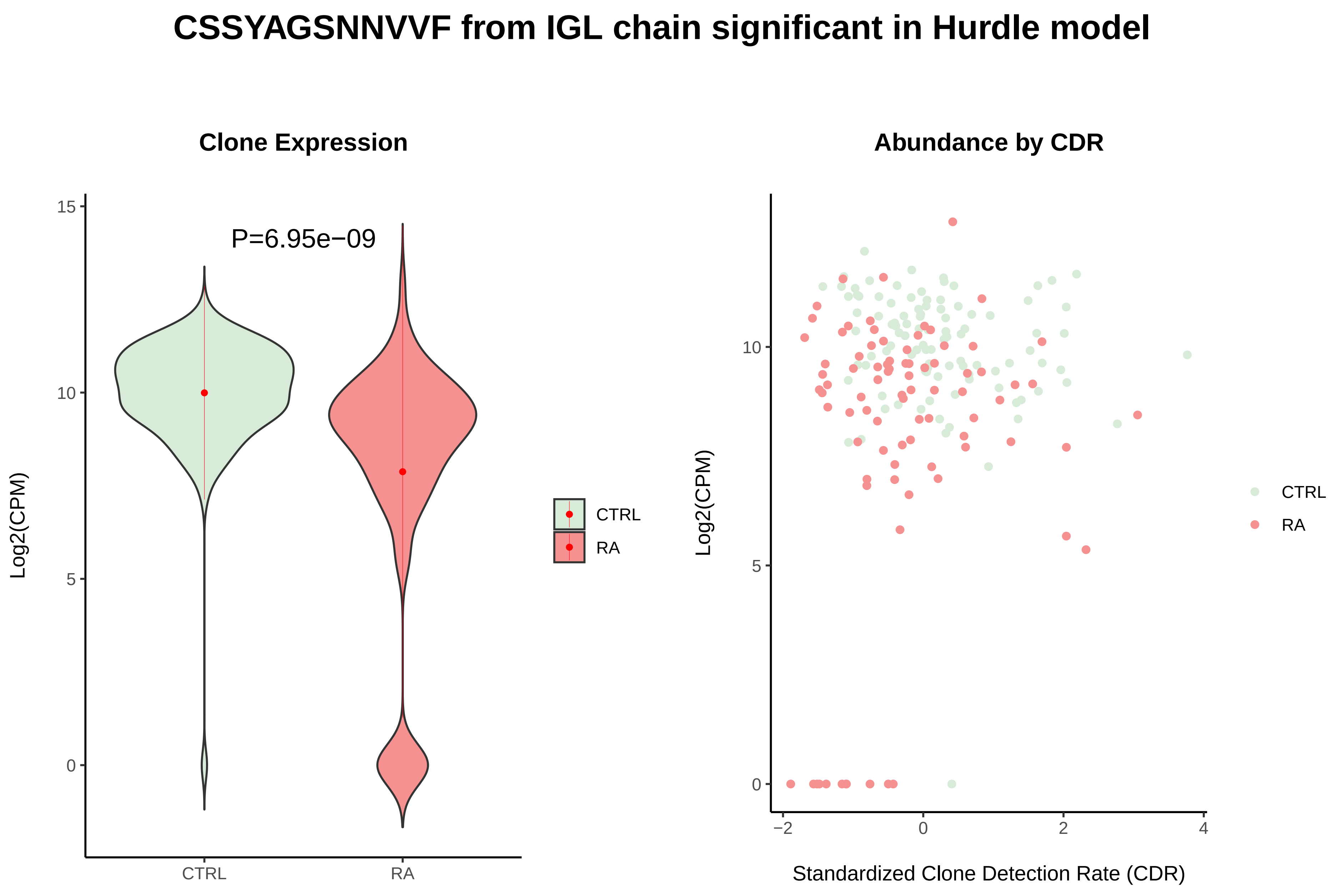

CSSYAGSNNVVF from IGL chain significant in Hurdle model
Clone Expression
P=6.95e−09
Log2(CPM)
15
10
5
0
CTRL
RA
CTRL
RA
Abundance by CDR
Log2(CPM)
10
5
0
−2
0
2
4
Standardized Clone Detection Rate (CDR)
CTRL
RA

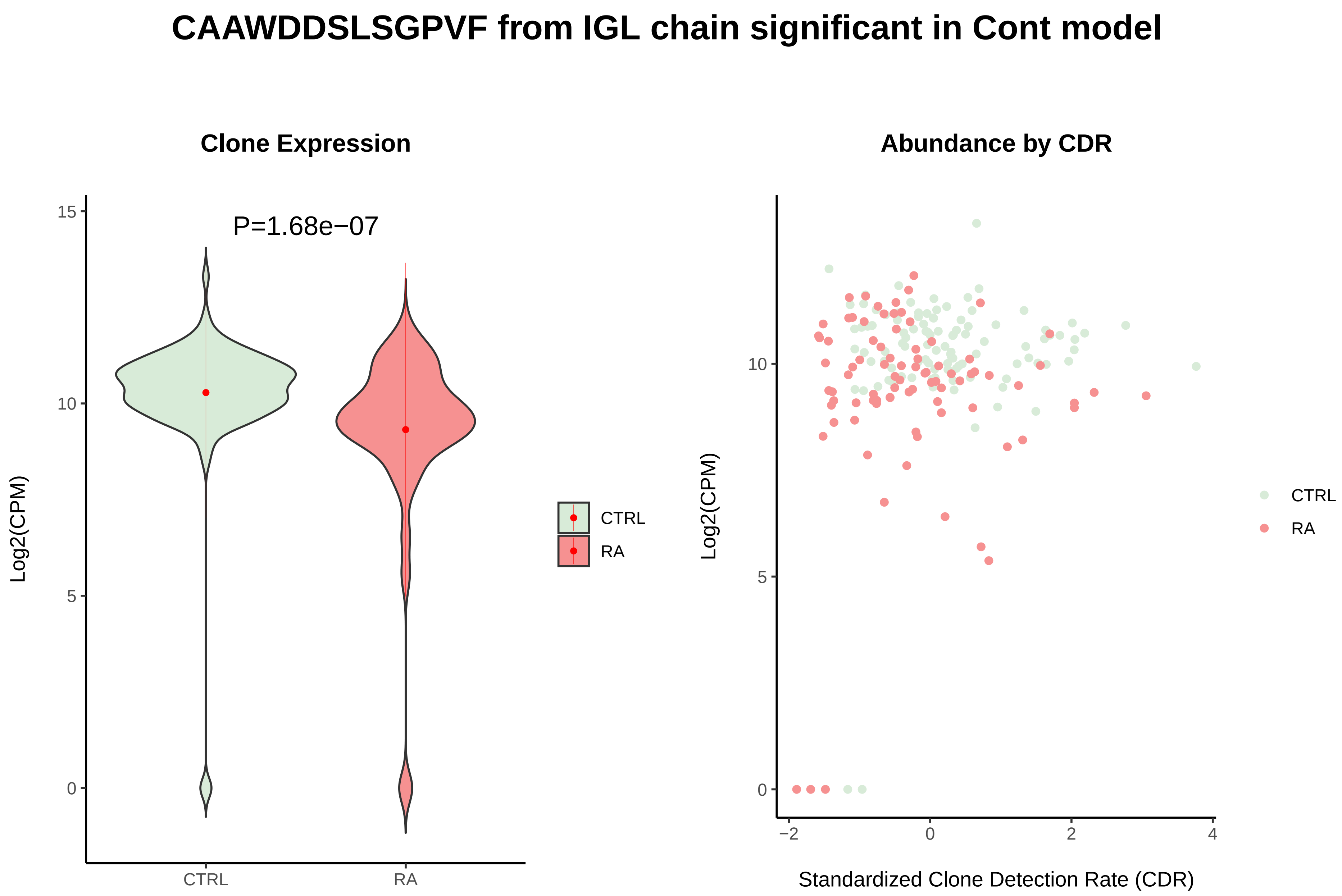

CAAWDDSLSGPVF from IGL chain significant in Cont model
Clone Expression
P=1.68e−07
Log2(CPM)
15
10
5
0
CTRL
RA
CTRL
RA
Abundance by CDR
Log2(CPM)
10
5
0
−2
0
2
4
Standardized Clone Detection Rate (CDR)
CTRL
RA

CAAWDDSLSGWVF from IGL chain significant in Cont model

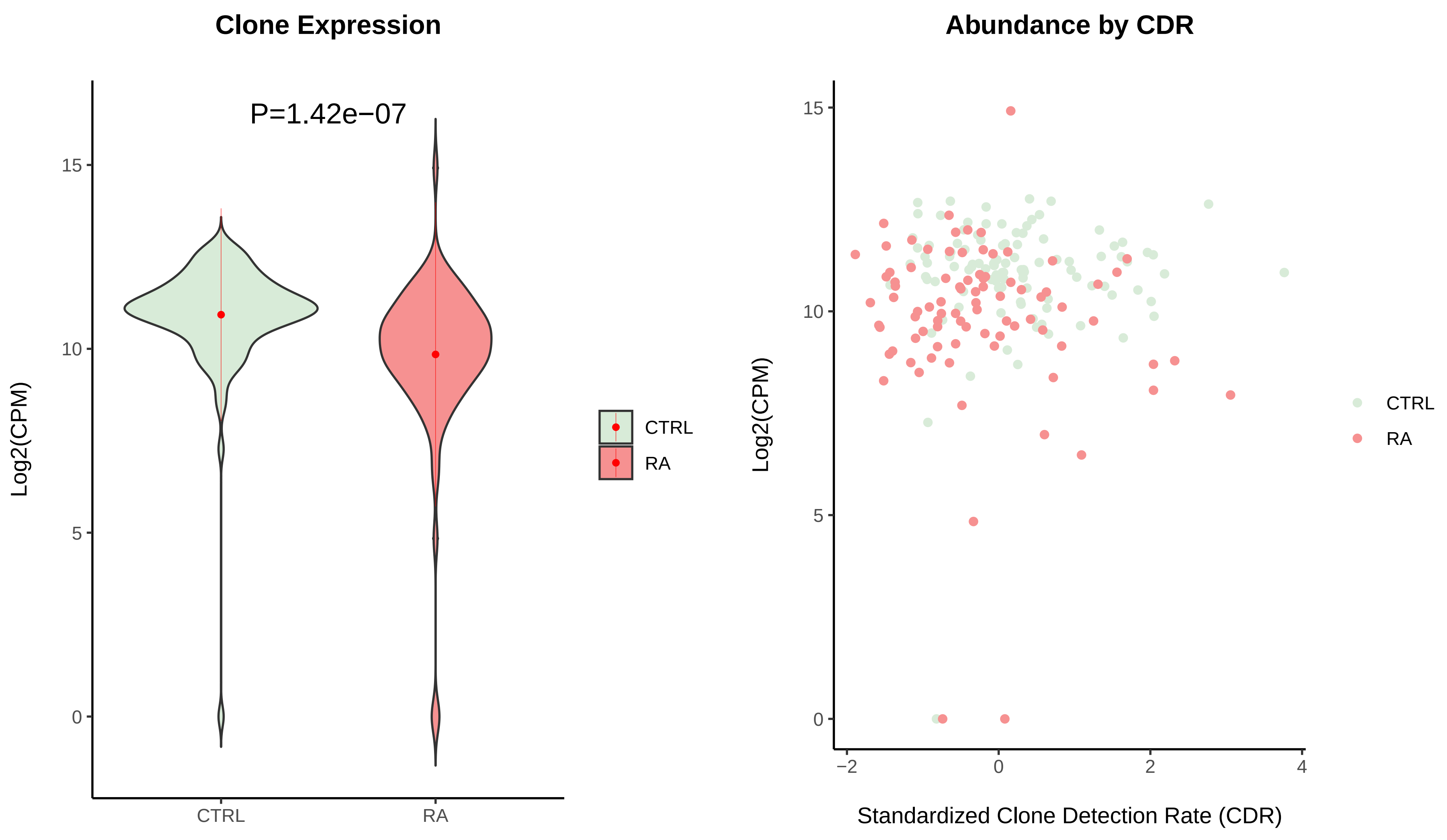

CQVWDSSSDHPVF from IGL chain significant in Cont model

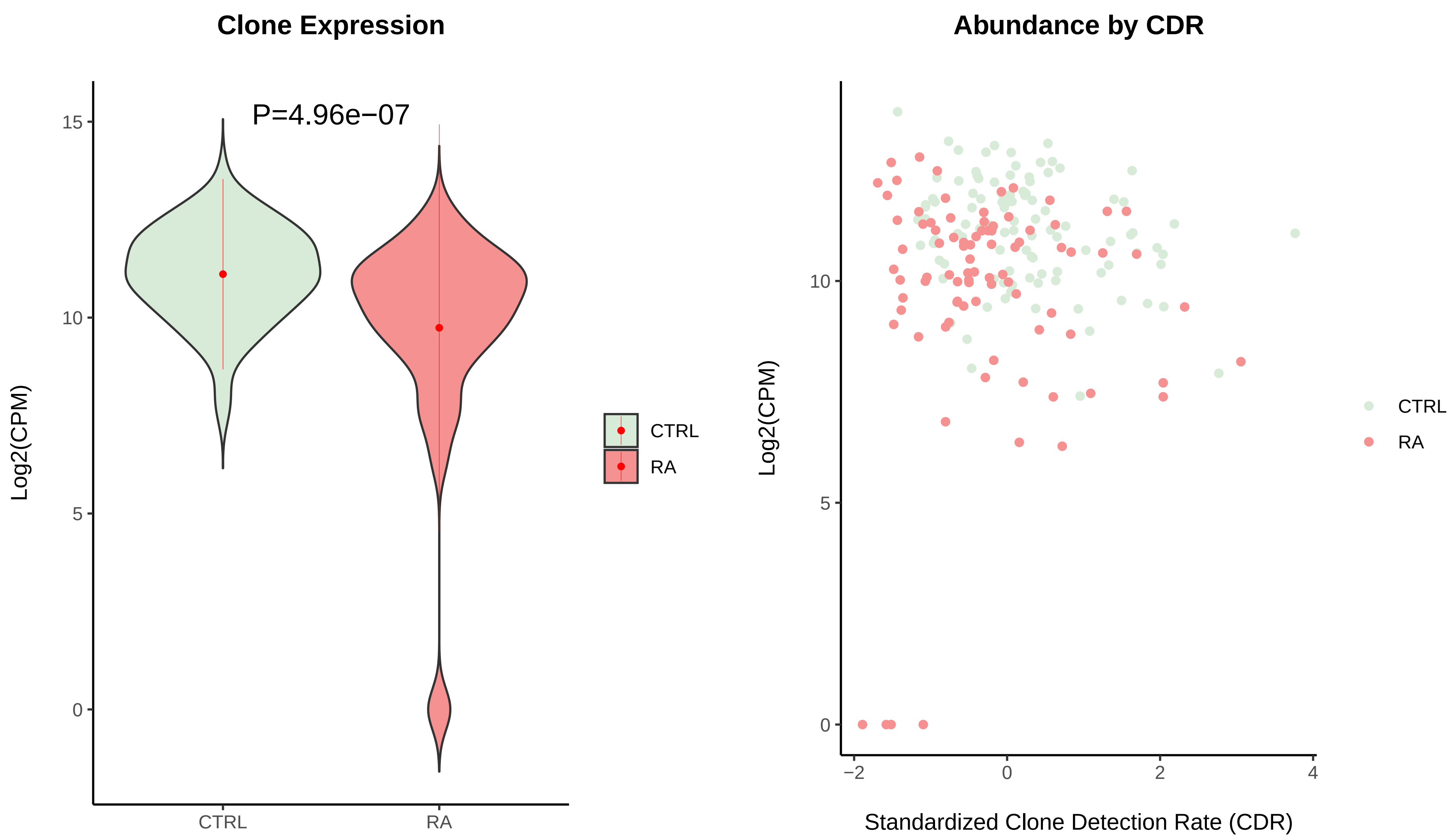

CQVWDSSSDHRVF from IGL chain significant in Cont model

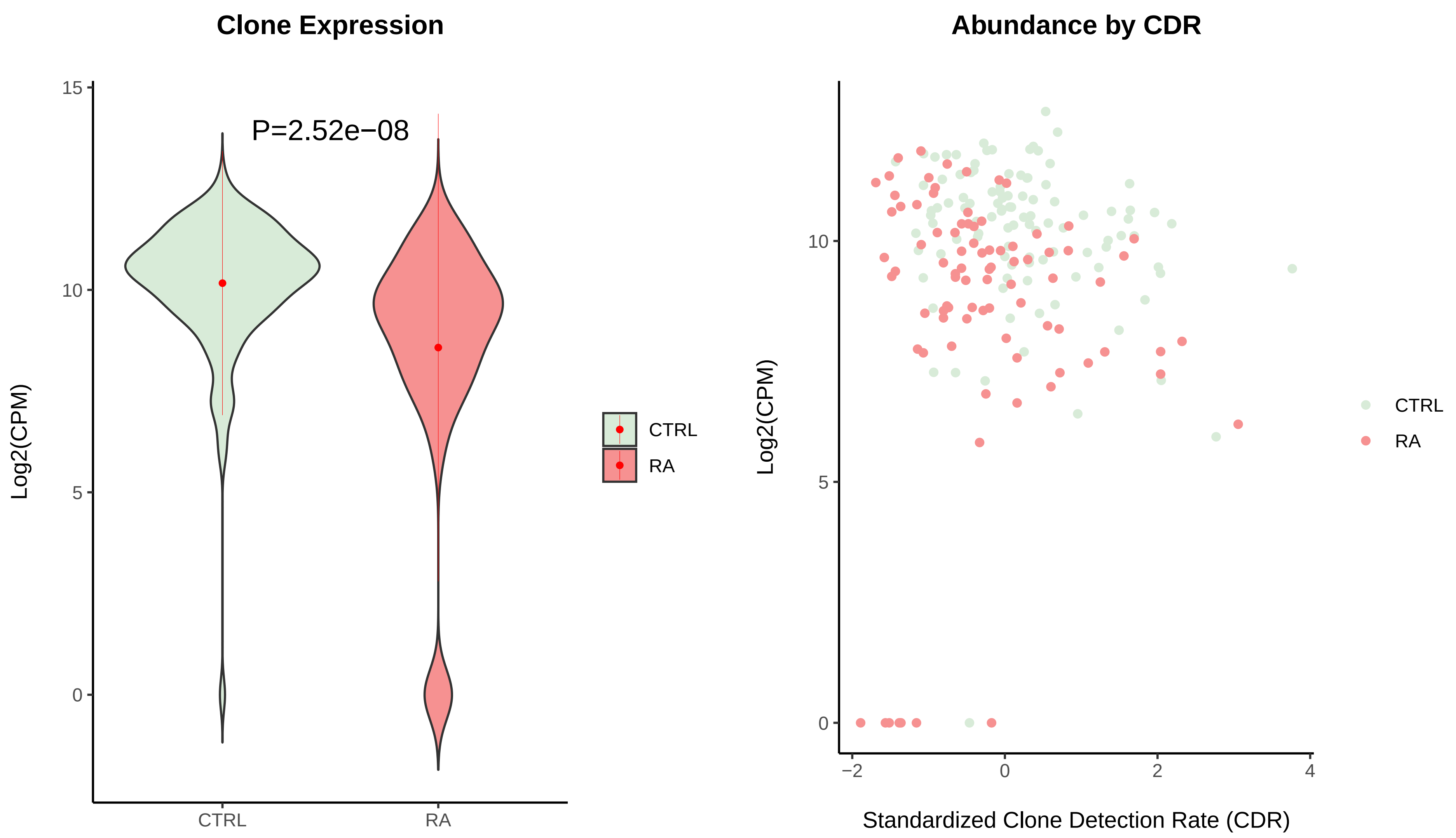

CQVWDSSSDHVVF from IGL chain significant in Cont model

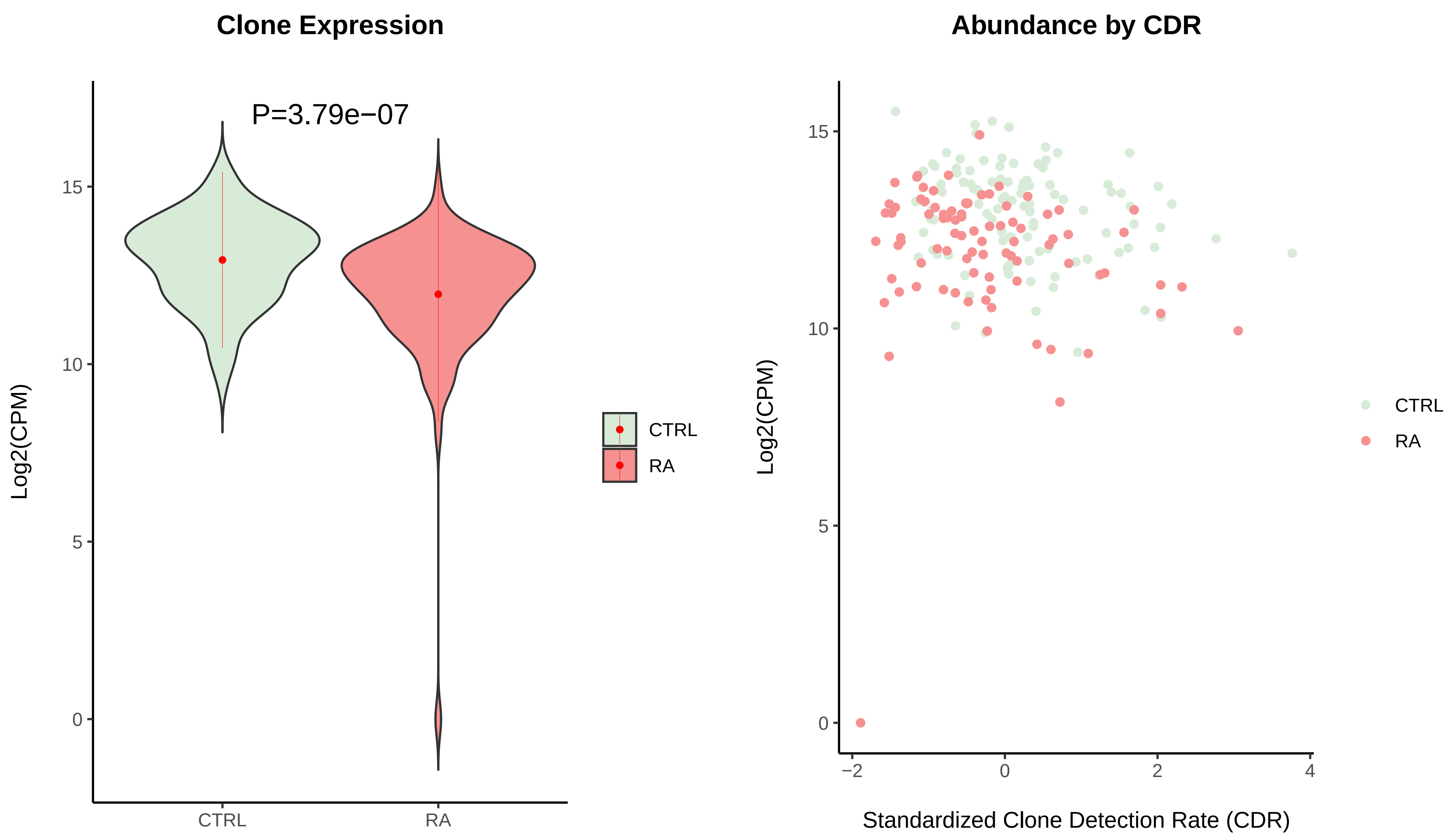

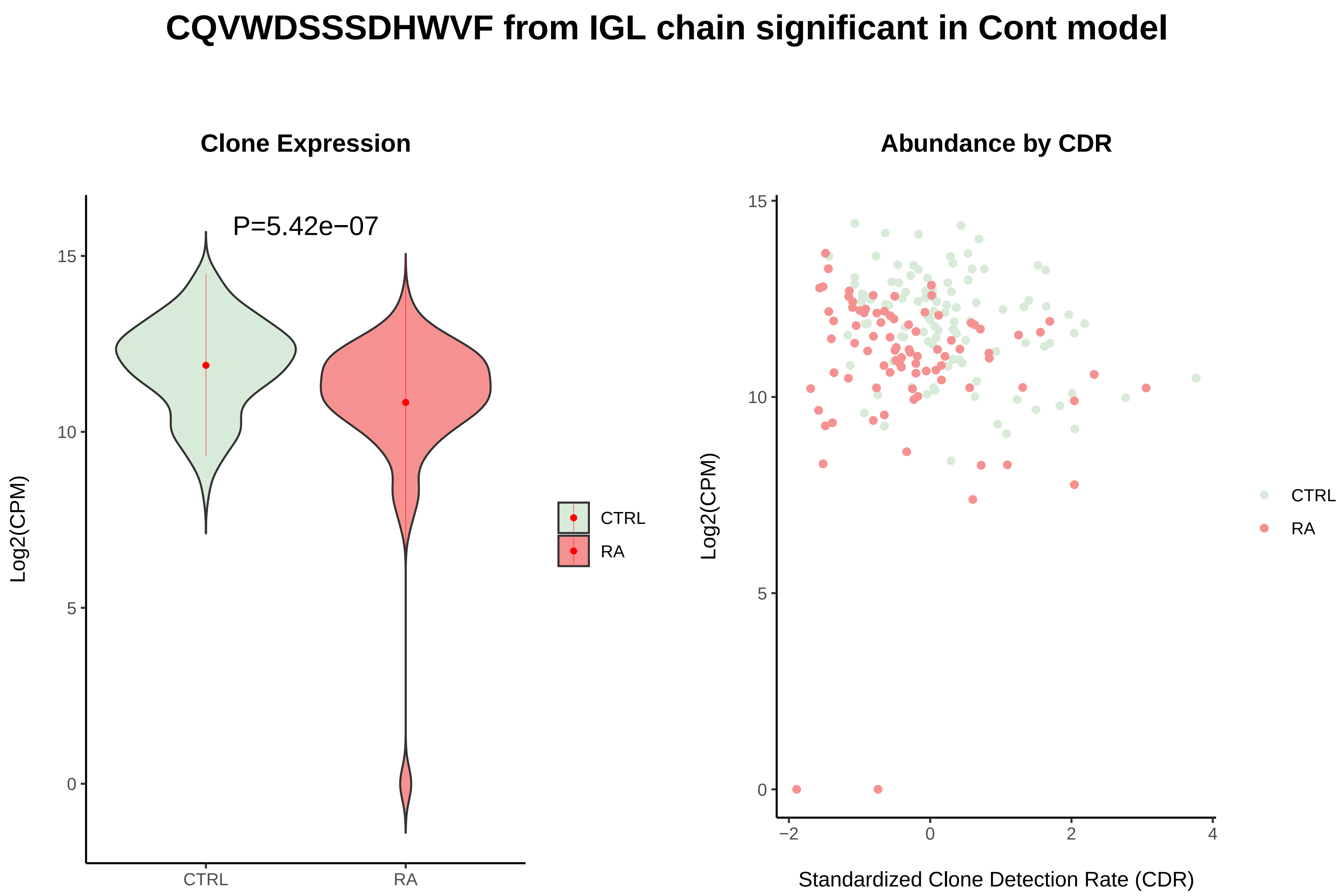

CQVWDSSSDHWVF from IGL chain significant in Cont model
Clone Expression
P=5.42e−07
Log2(CPM)
15
10
5
0
CTRL
RA
CTRL
RA
Abundance by CDR
Log2(CPM)
15
10
5
0
−2
0
2
4
Standardized Clone Detection Rate (CDR)
CTRL
RA

CQVWDSSSDHYVF from IGL chain significant in Cont model

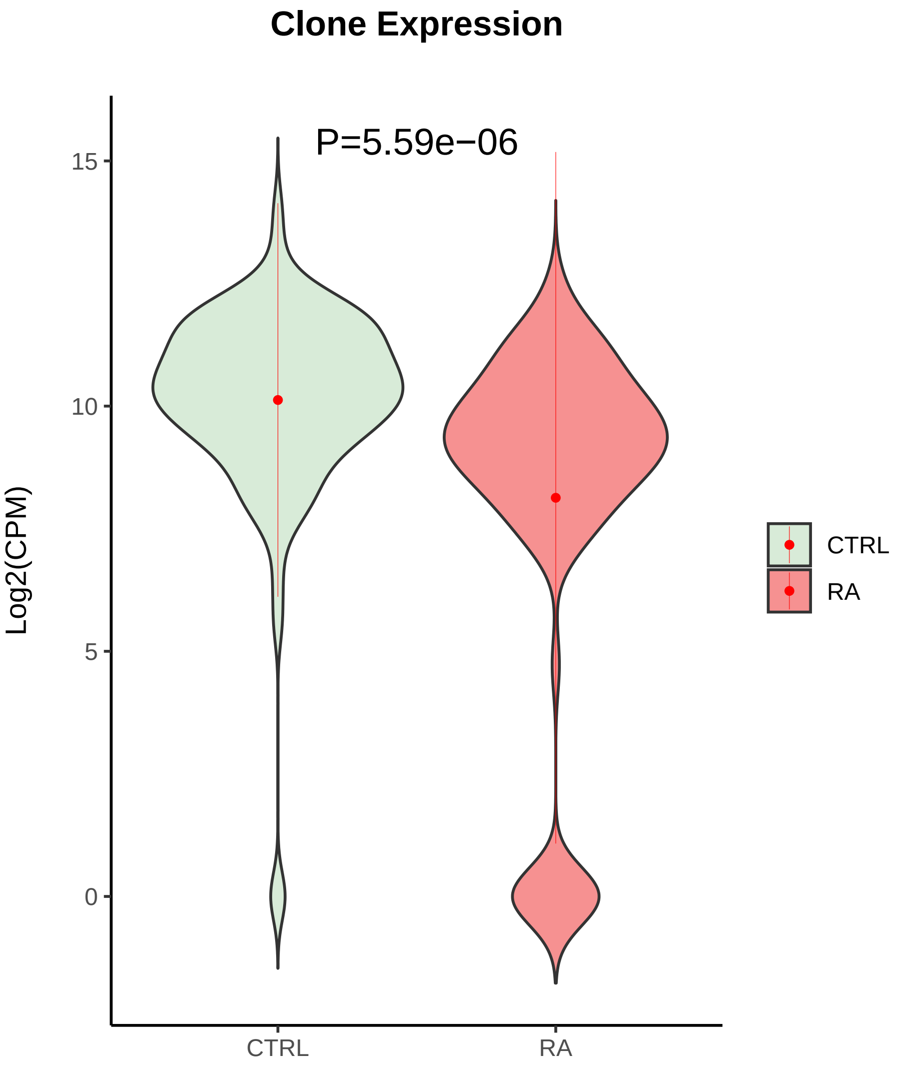

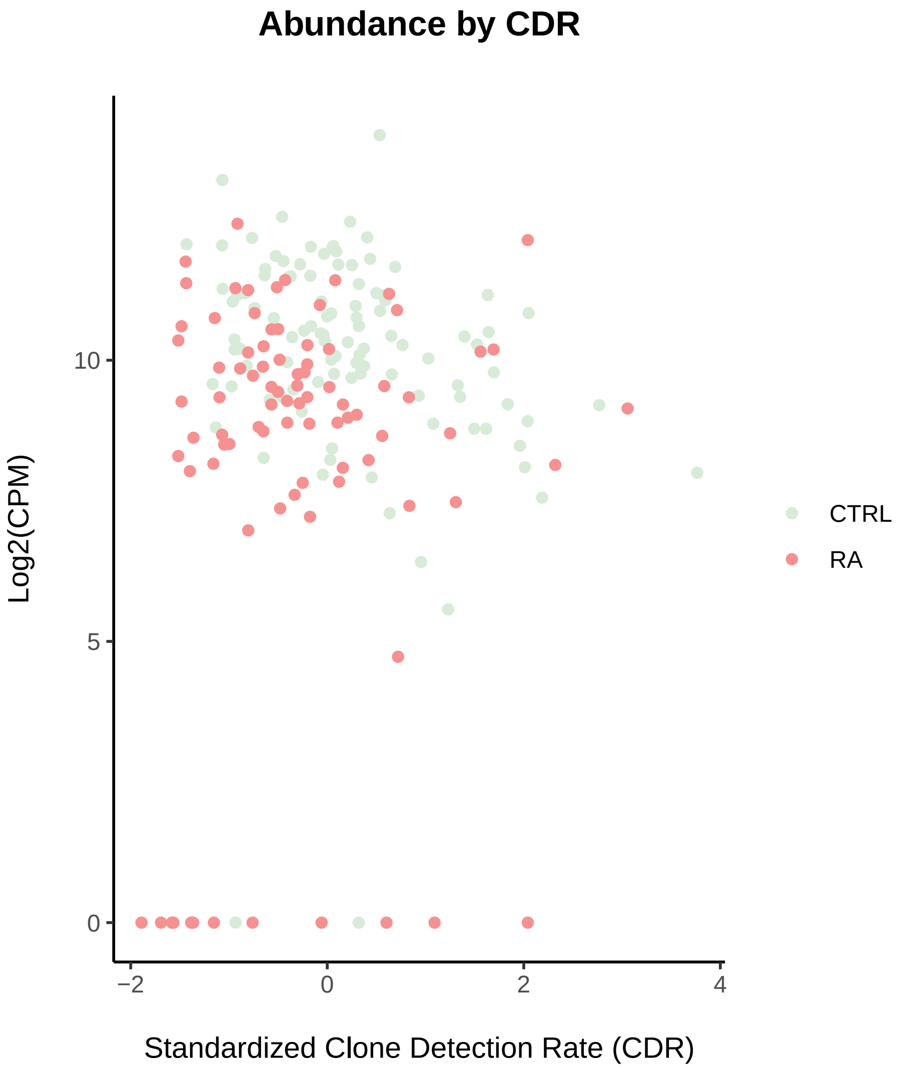

CSSYAGSNNVVF from IGL chain significant in Cont model

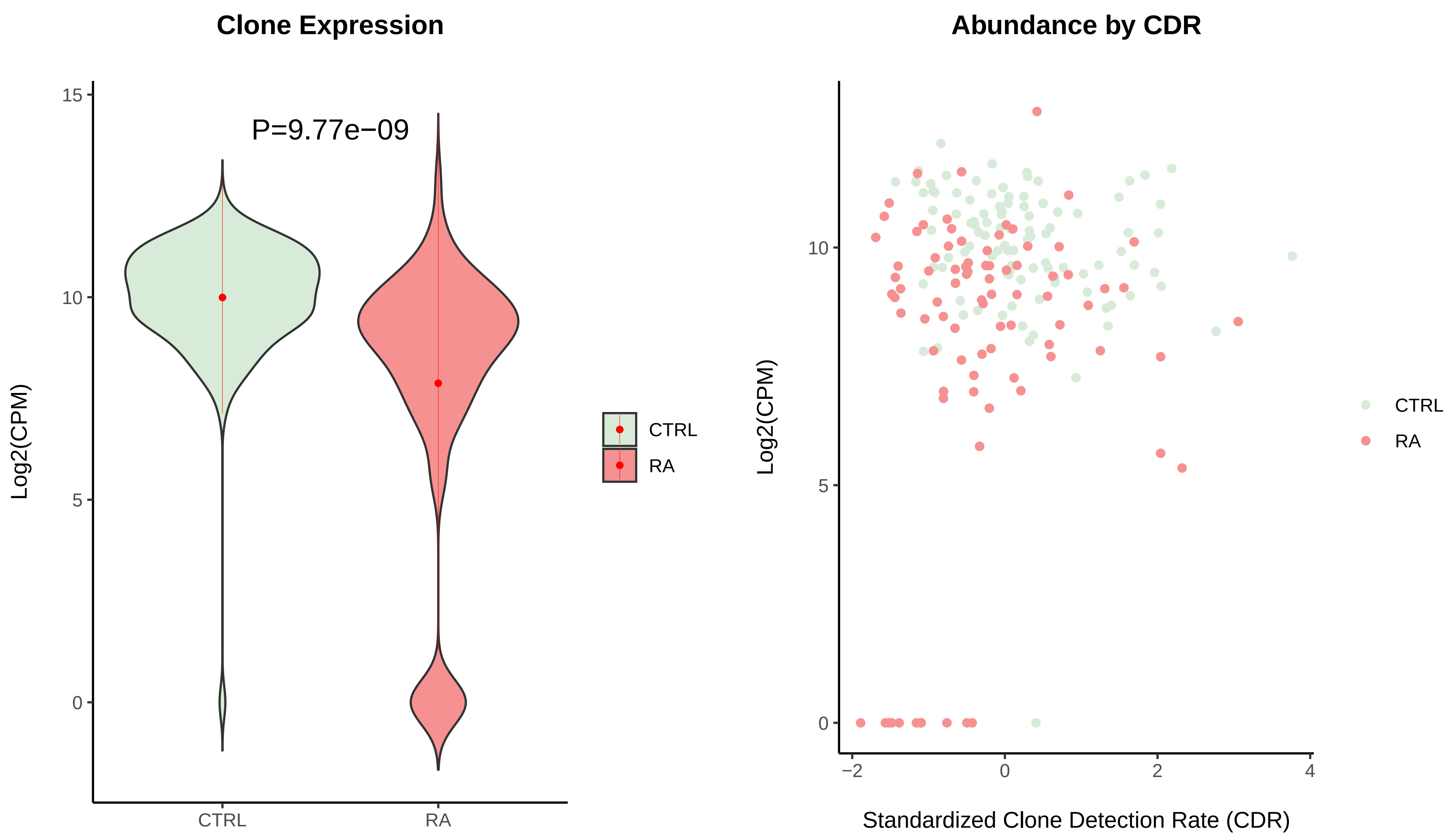

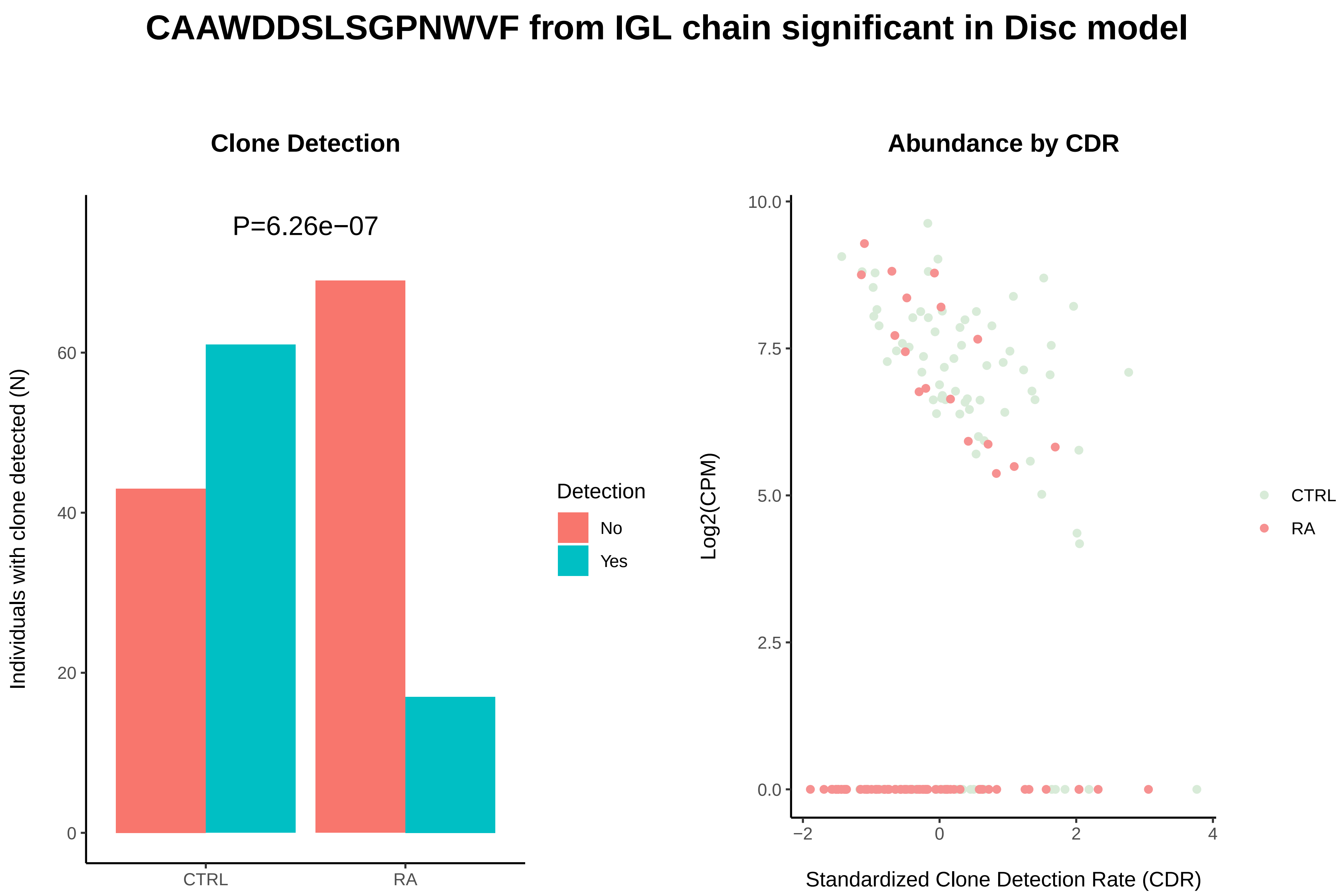

CAAWDDSLSGPNWVF from IGL chain significant in Disc model
Clone Detection
P=6.26e−07
Individuals with clone detected (N)
0
20
40
60
CTRL
RA
Detection
No
Yes
Abundance by CDR
Log2(CPM)
0.0
2.5
5.0
7.5
10.0
−2
0
2
4
Standardized Clone Detection Rate (CDR)
CTRL
RA

CGADHGSGSNFVKVF from IGL chain significant in Disc model

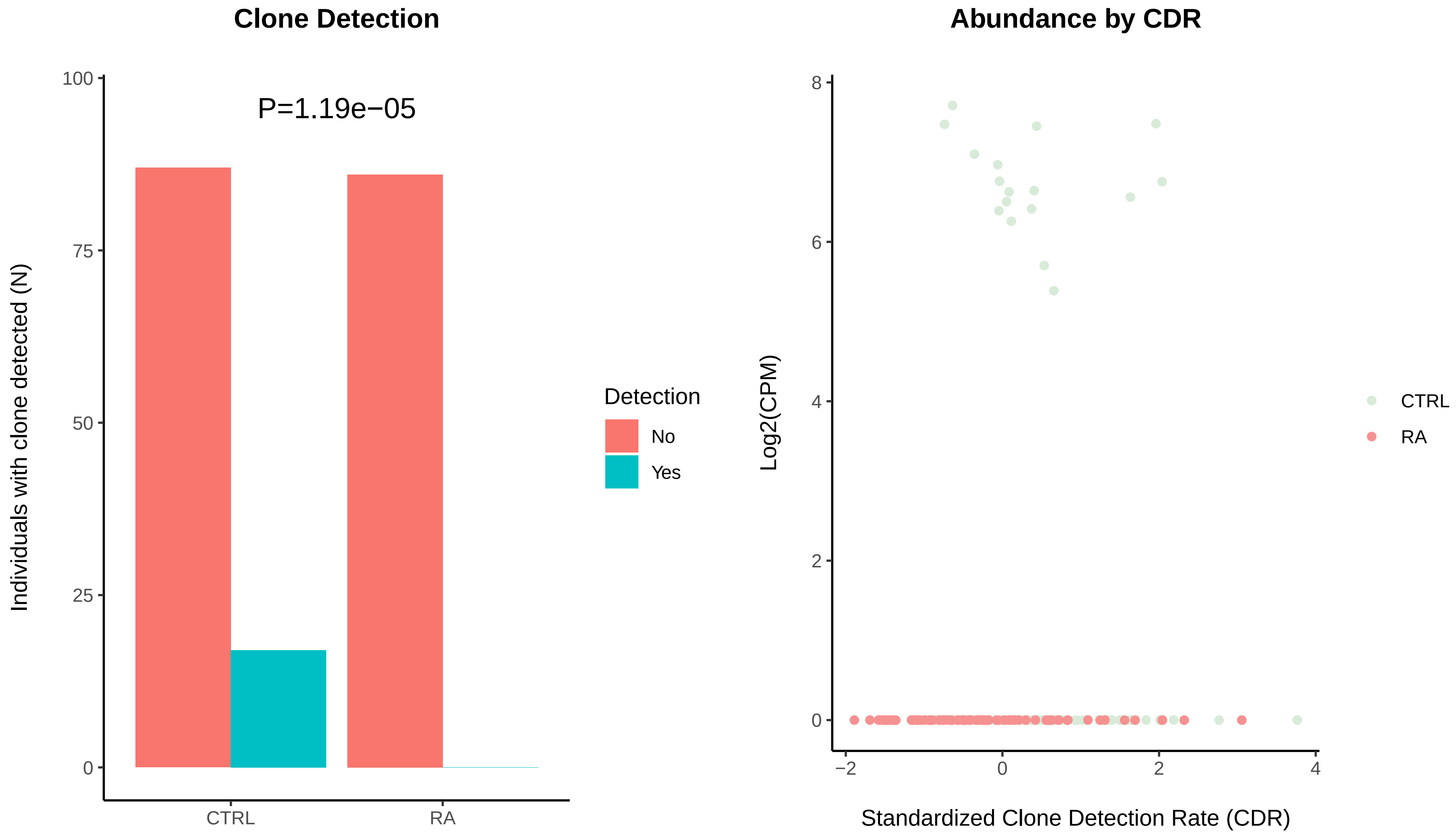

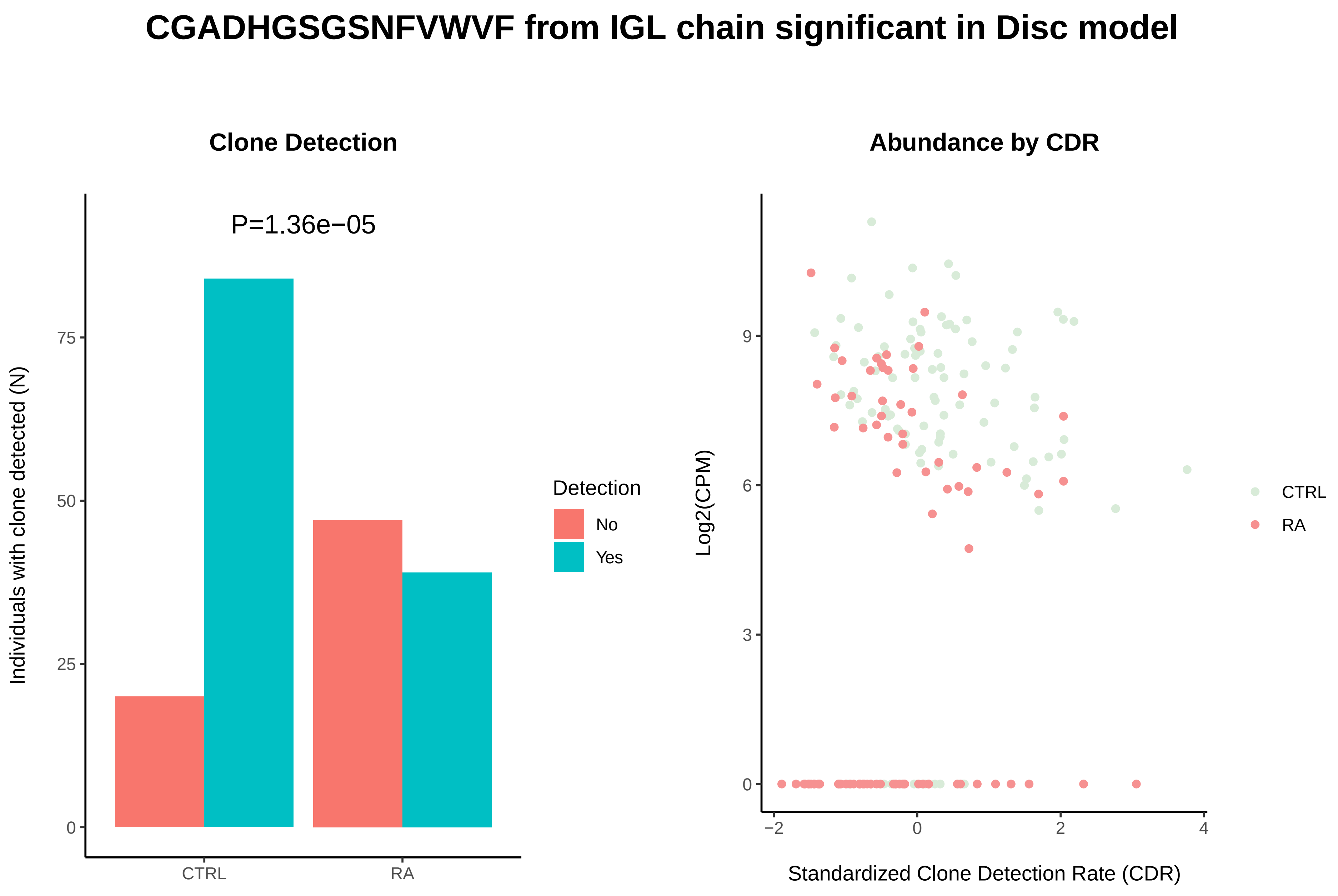
CGADHGSGSNFVWVF from IGL chain significant in Disc model
Clone Detection
P=1.36e−05
Individuals with clone detected (N)
0
25
50
75
CTRL
RA
Detection
No
Yes
Abundance by CDR
Log2(CPM)
0
3
6
9
−2
0
2
4
Standardized Clone Detection Rate (CDR)
CTRL
RA

CQSYDSSLYVVF from IGL chain significant in Disc model

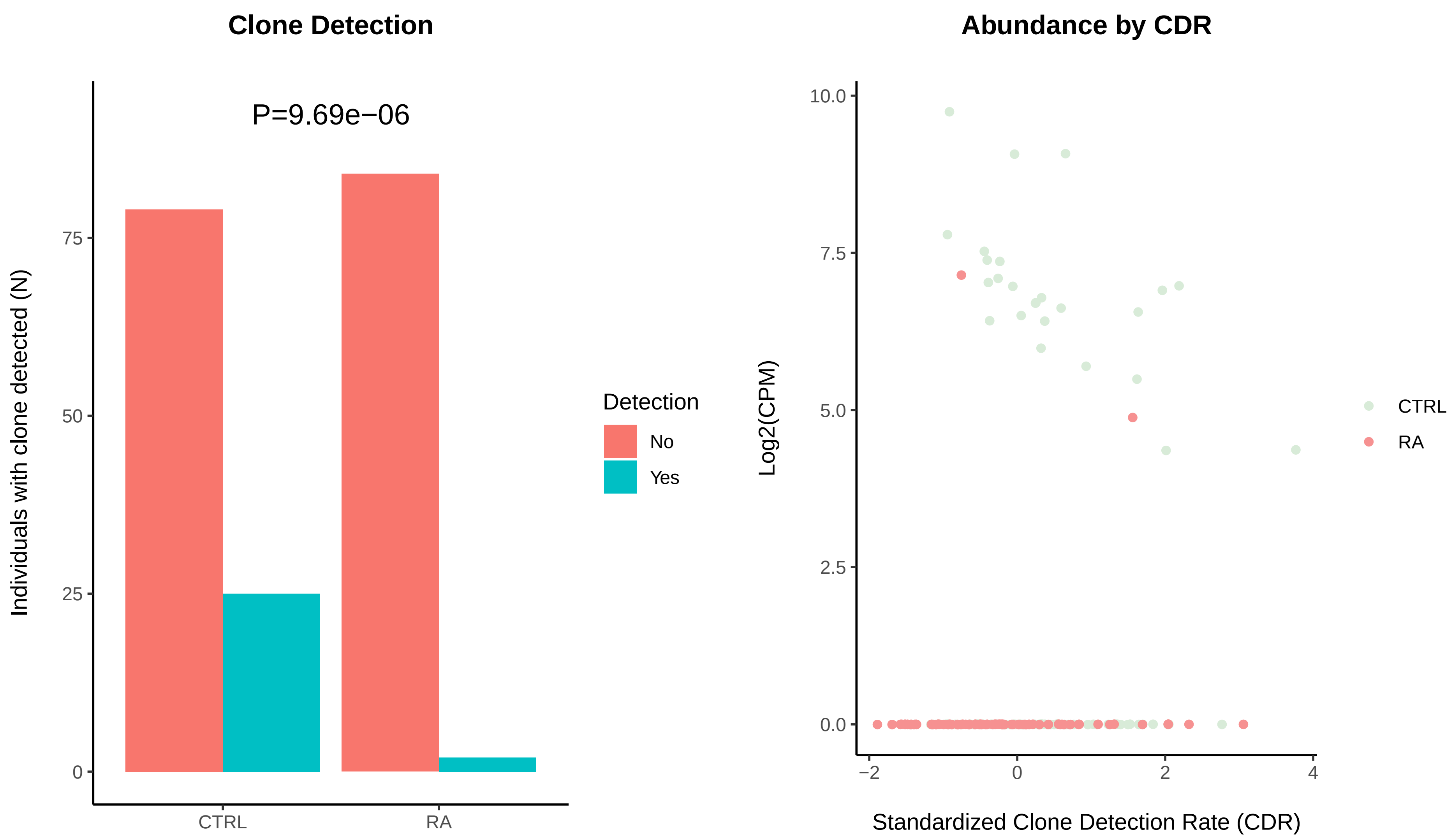

CQVWDSSSDHPKVVF from IGL chain significant in Disc model

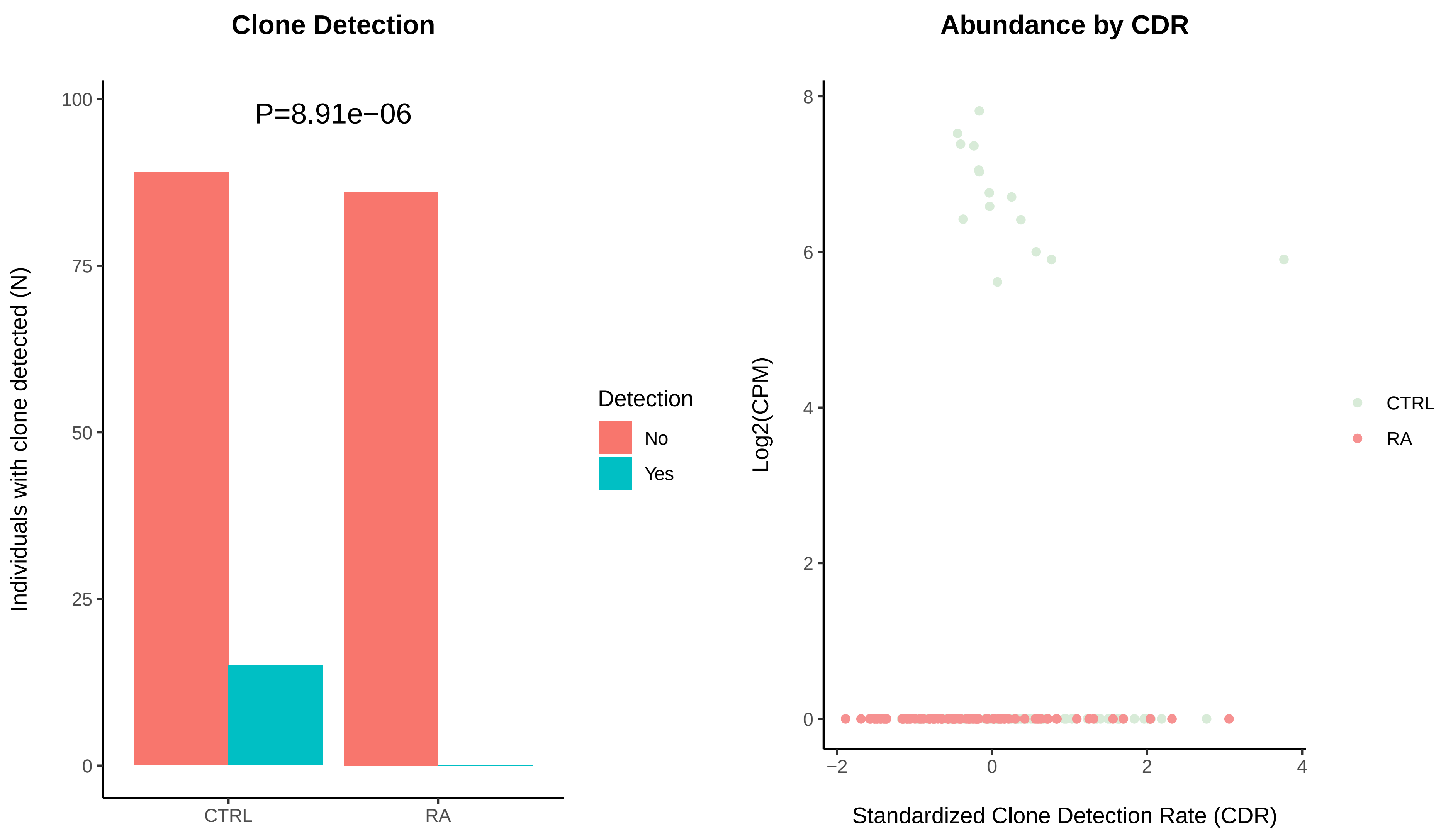

CQVWDSSSDQEVF from IGL chain significant in Disc model

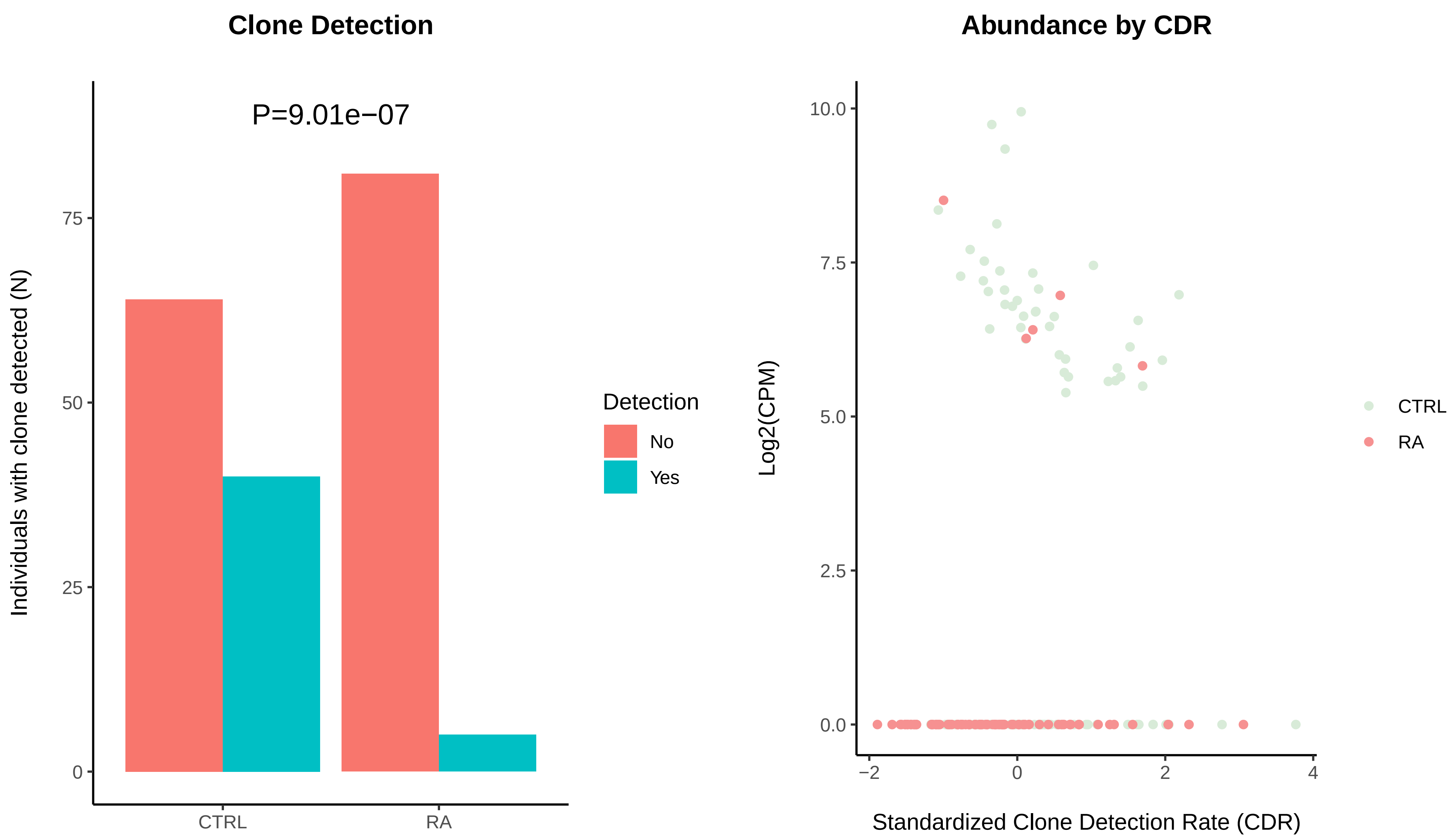

CQVWDSSSDRVF from IGL chain significant in Disc model

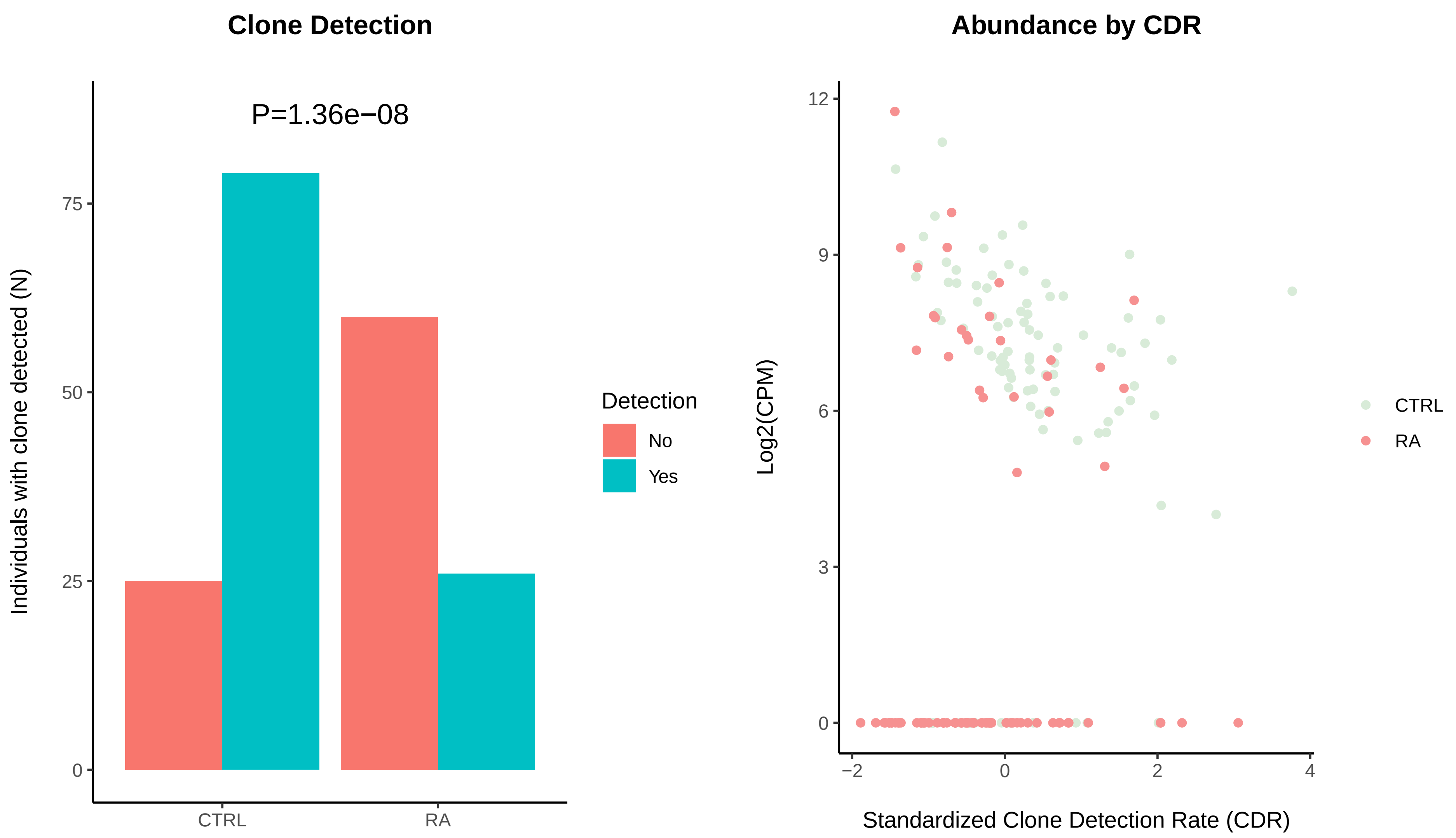

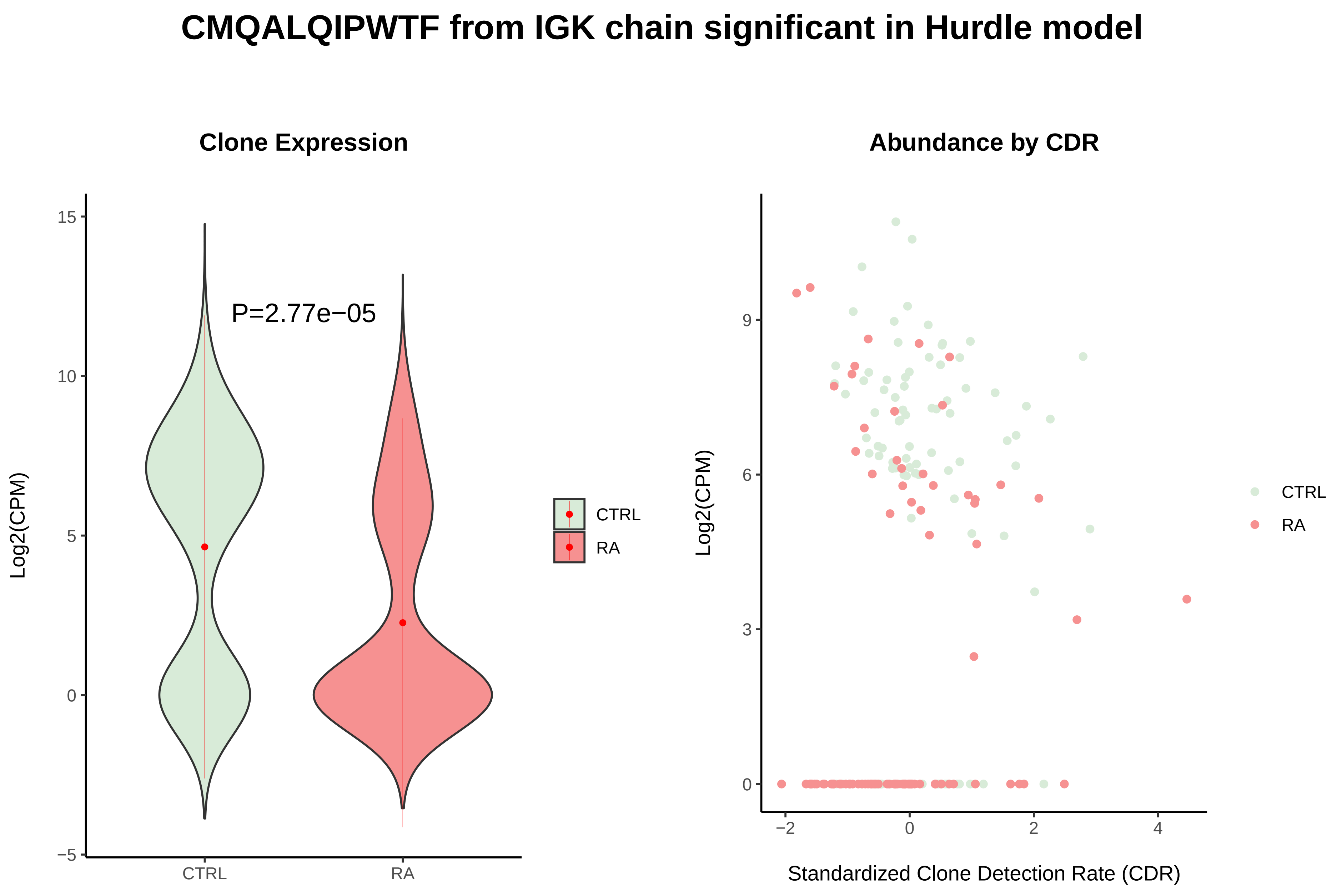

CMQALQIPWTF from IGK chain significant in Hurdle model
Clone Expression
Abundance by CDR
P=2.77e−05
Log2(CPM)
15
10
5
0
−5
CTRL
RA
9
6
3
0
−2
0
2
4
Standardized Clone Detection Rate (CDR)

CMQALQTLTF from IGK chain significant in Hurdle model

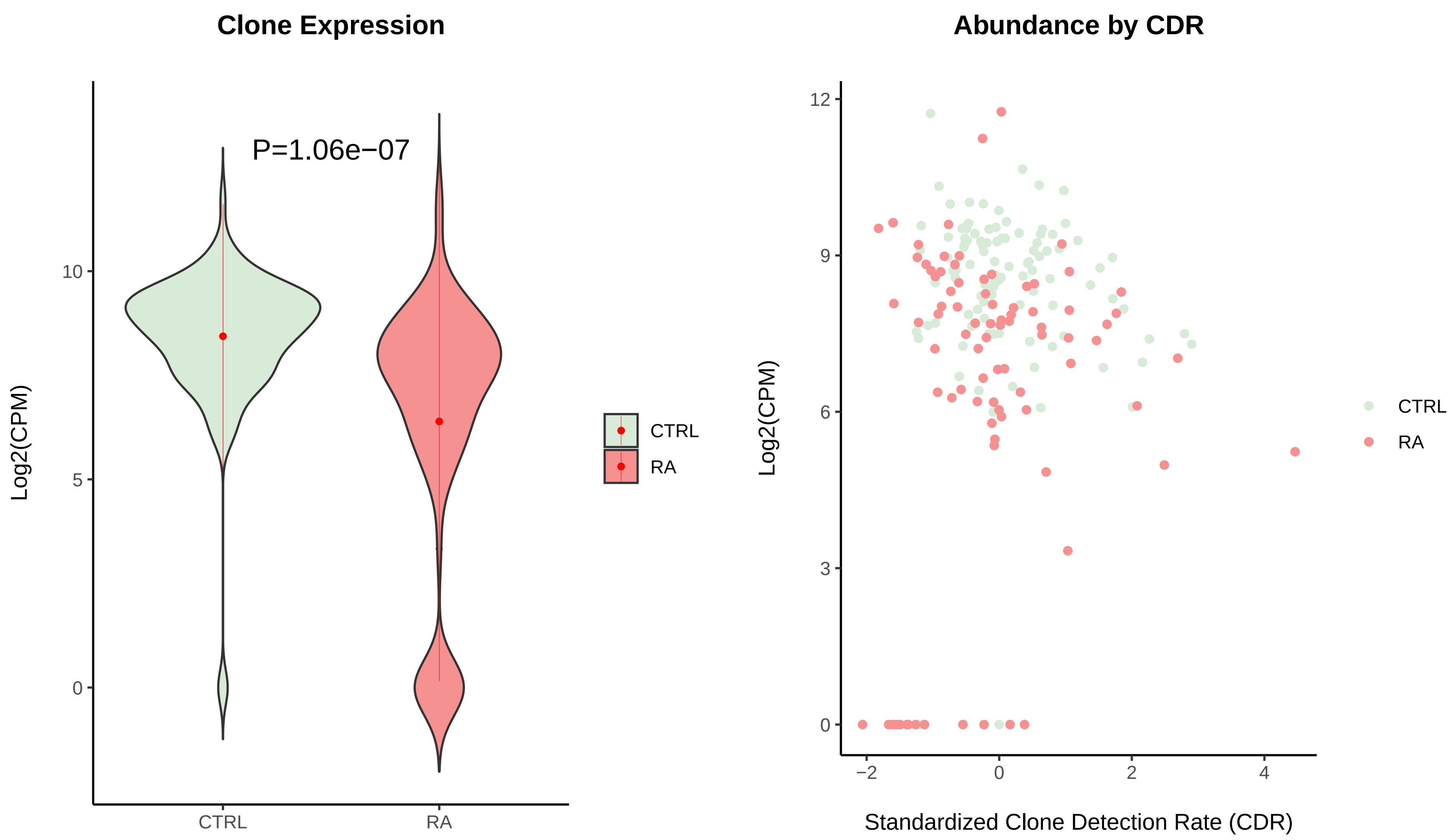

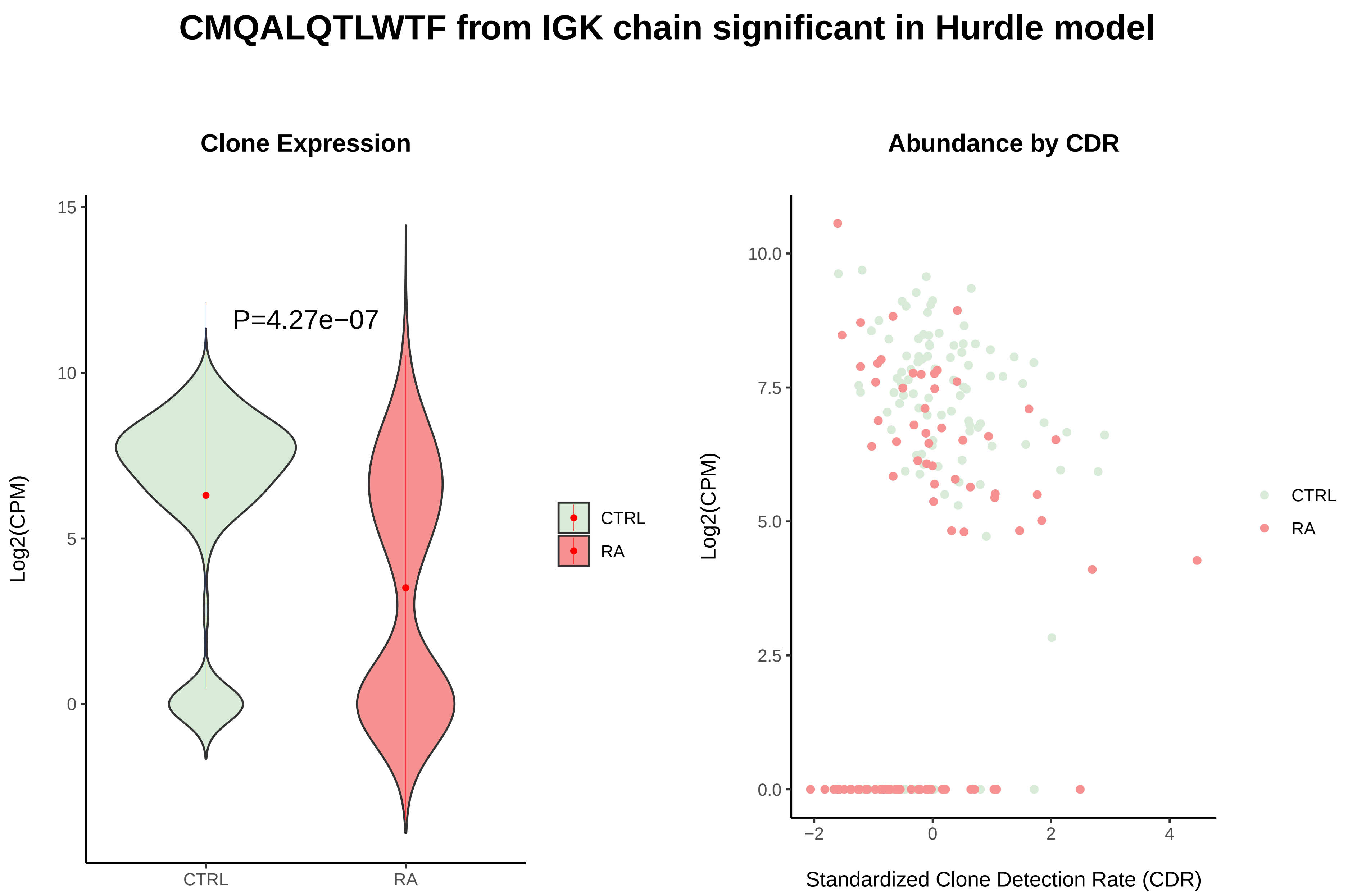

CMQALQTLWTF from IGK chain significant in Hurdle model
Clone Expression
P=4.27e−07
Log2(CPM)
15
10
5
0
CTRL
RA
CTRL
RA
Abundance by CDR
Log2(CPM)
10.0
7.5
5.0
2.5
0.0
−2
0
2
4
Standardized Clone Detection Rate (CDR)
CTRL
RA

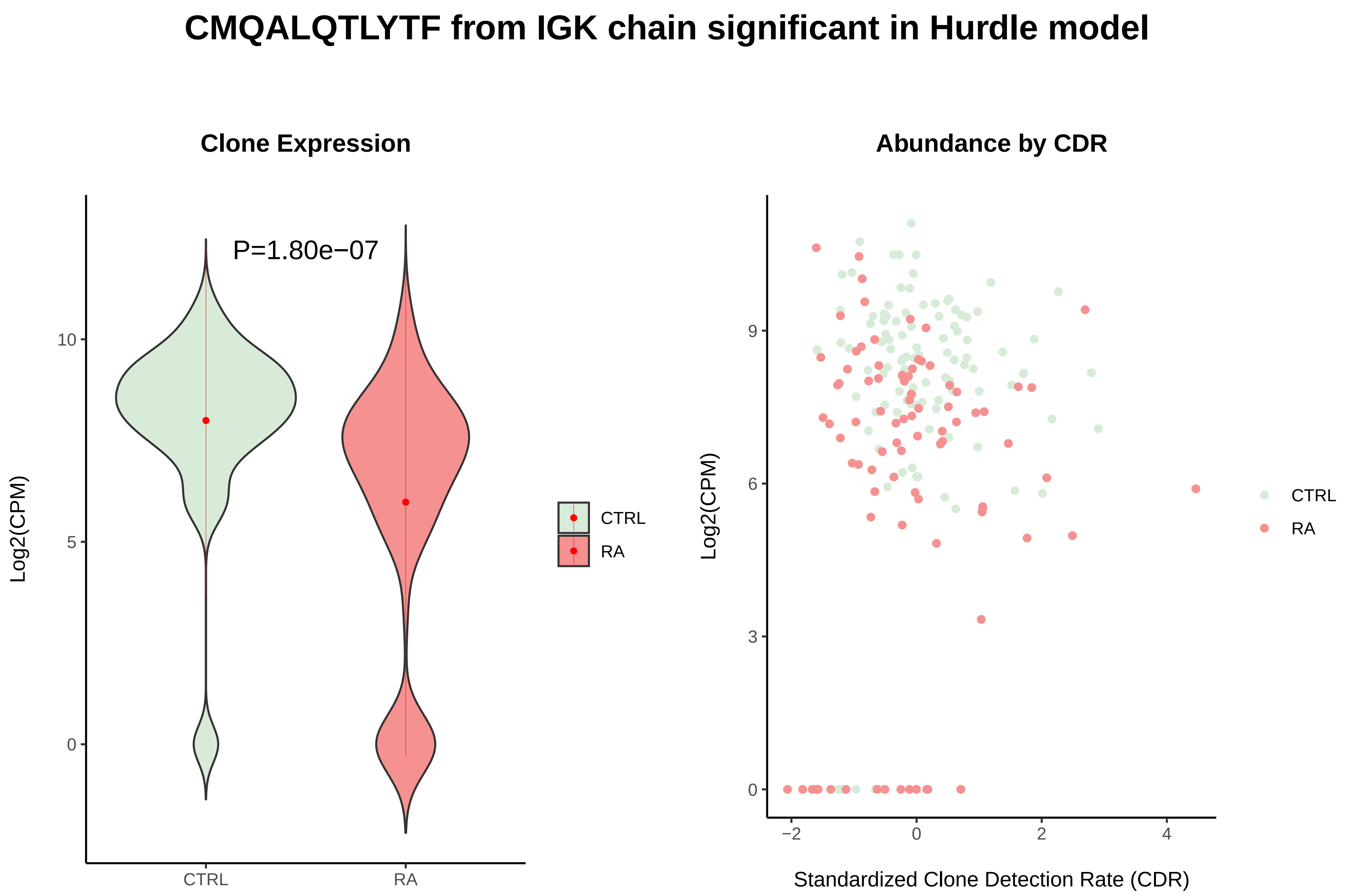
CMQALQTLYTF from IGK chain significant in Hurdle model
Clone Expression
P=1.80e−07
Log2(CPM)
10
5
0
CTRL
RA
CTRL
RA
Abundance by CDR
Log2(CPM)
9
6
3
0
−2
0
2
4
Standardized Clone Detection Rate (CDR)
CTRL
RA

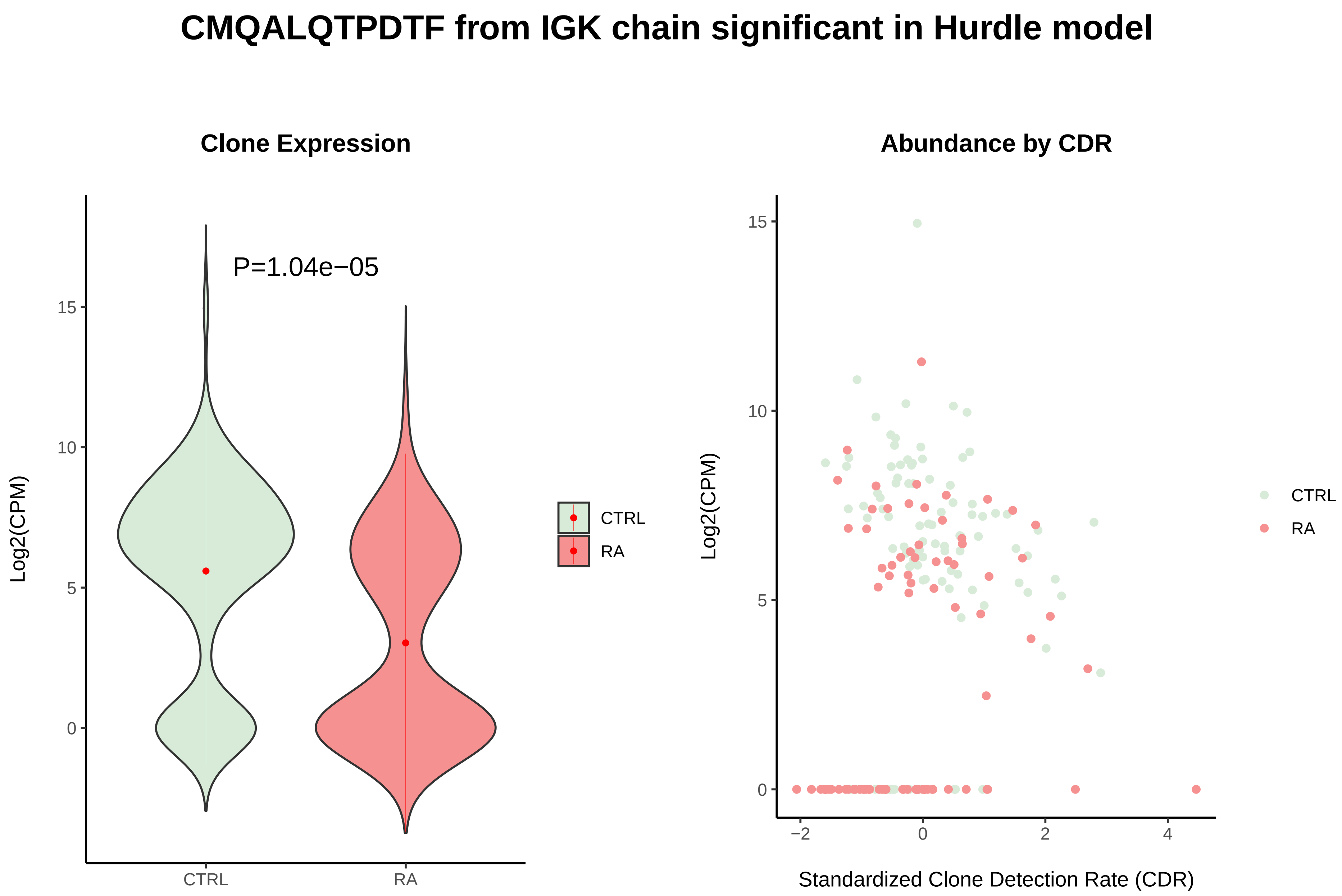
CMQALQTPDTF from IGK chain significant in Hurdle model
Clone Expression
P=1.04e−05
Log2(CPM)
0
5
10
15
CTRL
RA
Abundance by CDR
Log2(CPM)
Standardized Clone Detection Rate (CDR)
−2
0
2
4
CTRL
RA

CMQALQTPFTF from IGK chain significant in Hurdle model

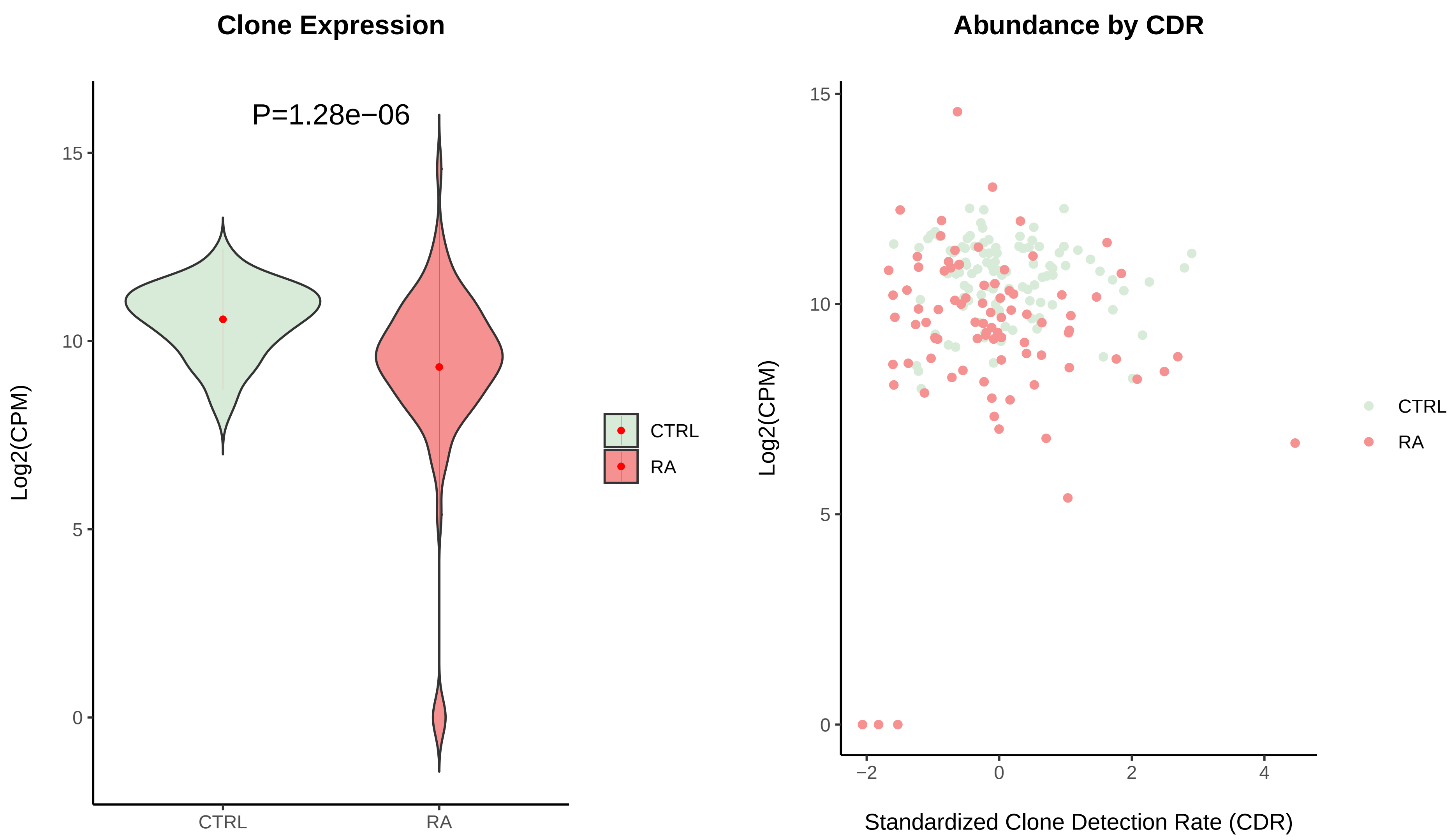

CMQALQTPGTF from IGK chain significant in Hurdle model

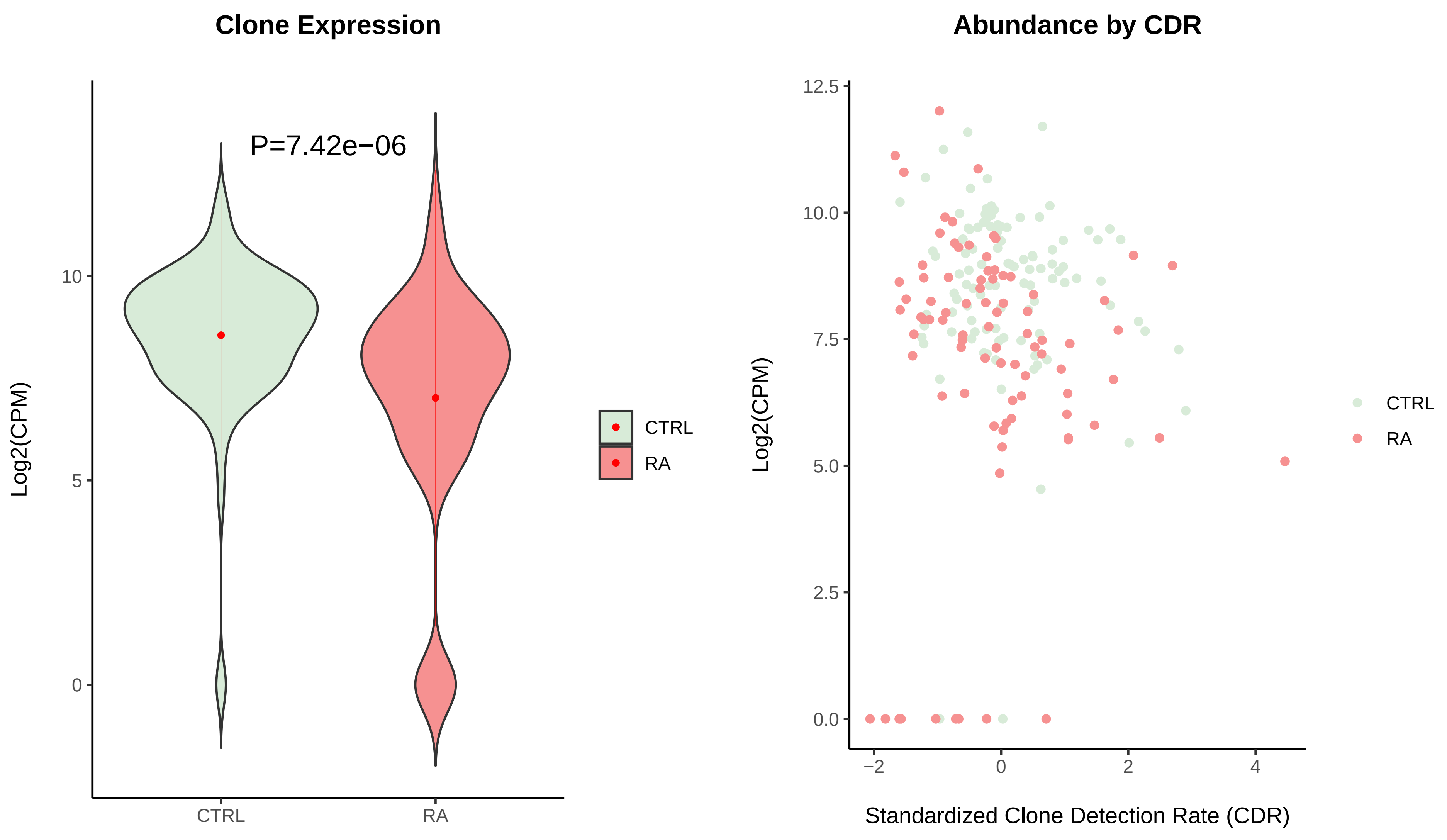

CMQALQTPHTF from IGK chain significant in Hurdle model

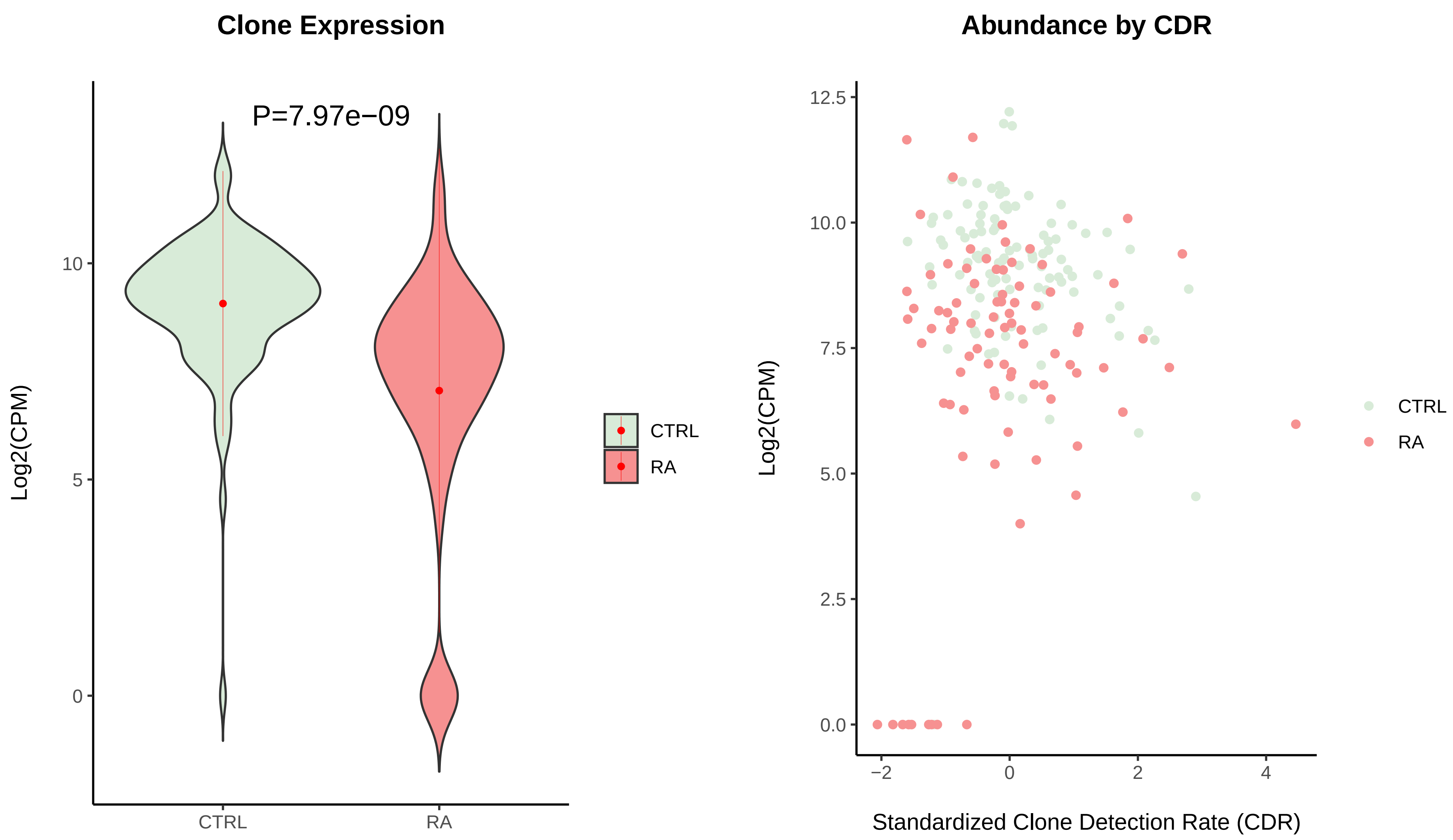

CMQALQTPITF from IGK chain significant in Hurdle model

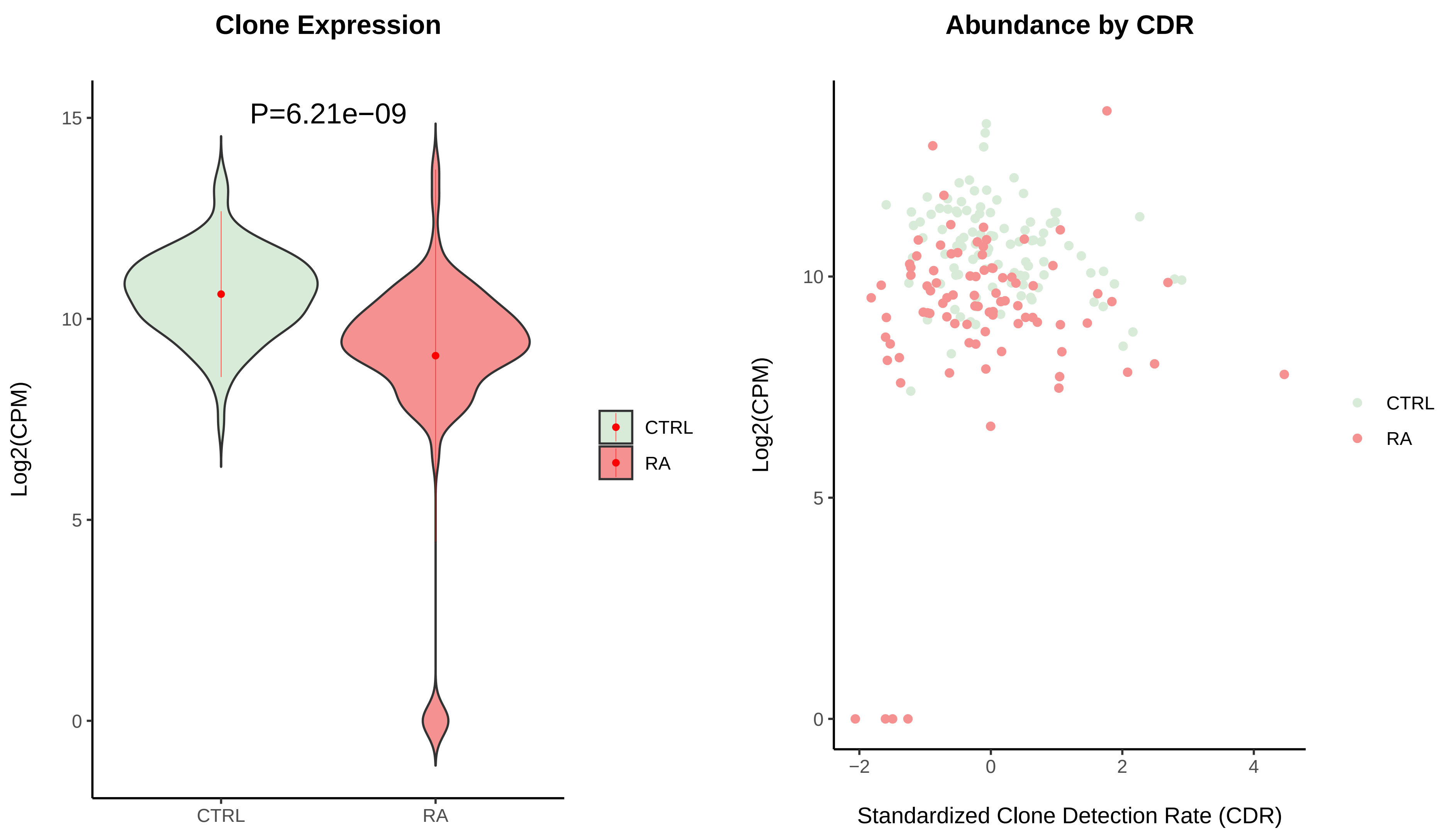

CMQALQTPLTF from IGK chain significant in Hurdle model

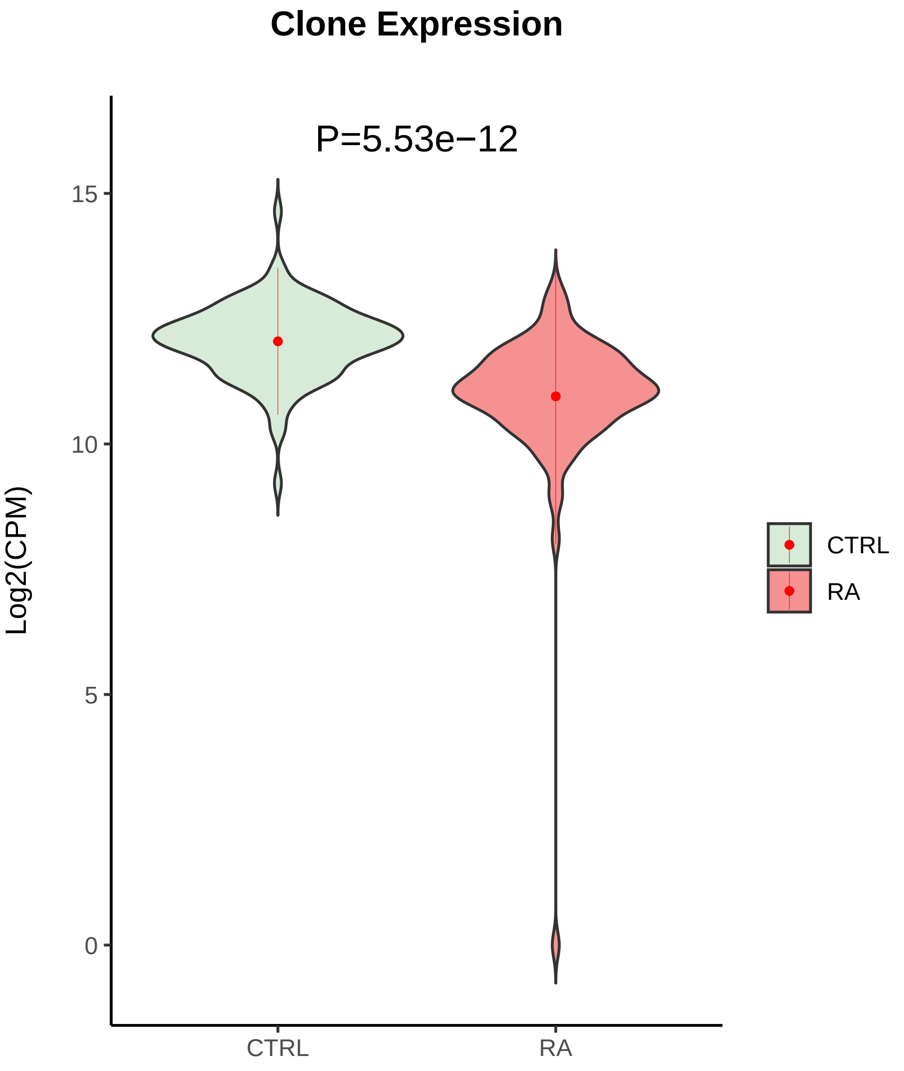

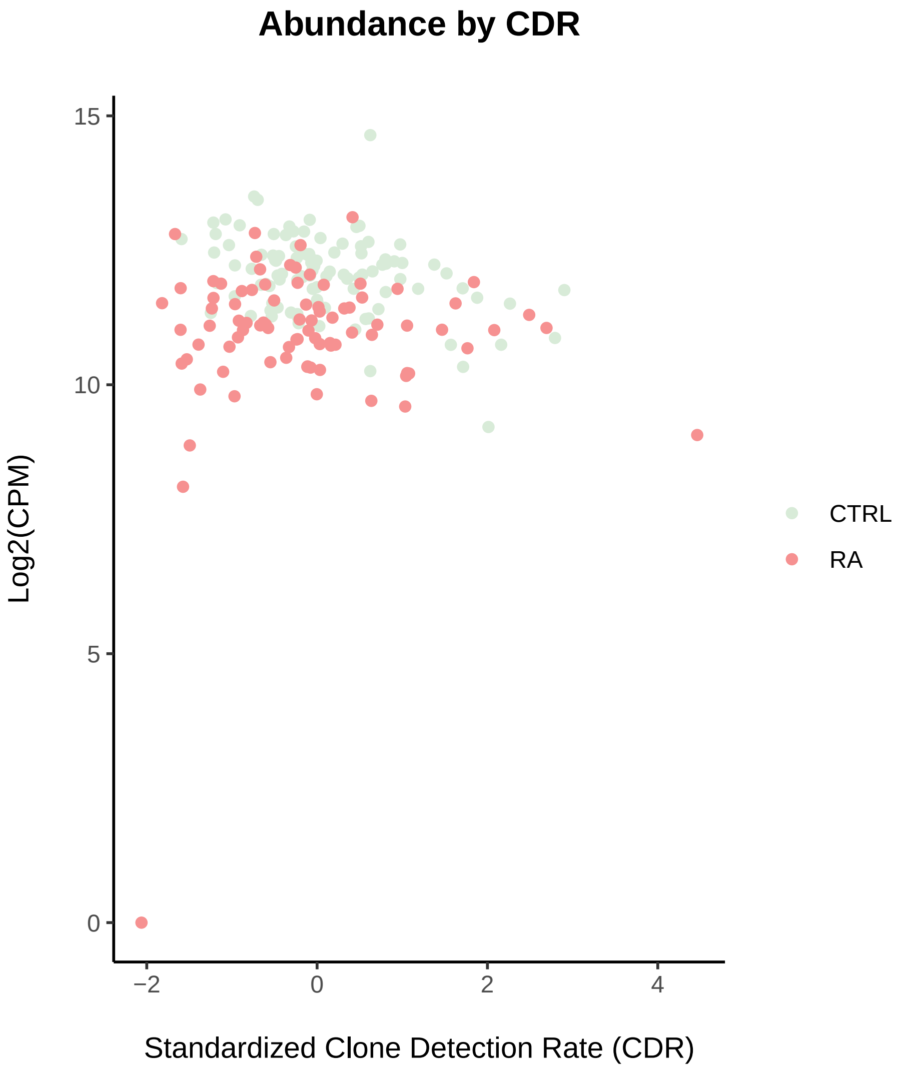

CMQALQTPMYTF from IGK chain significant in Hurdle model

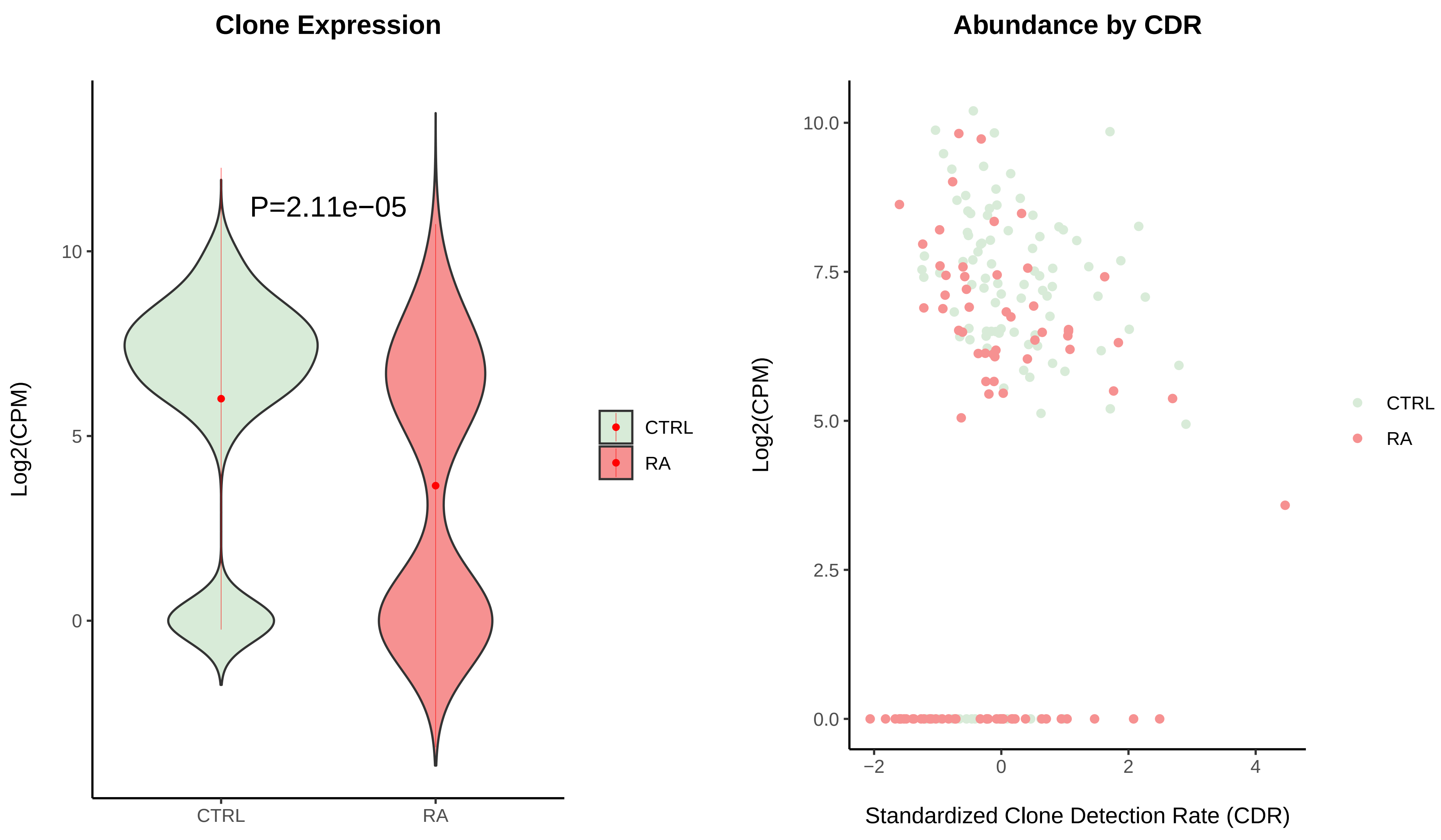

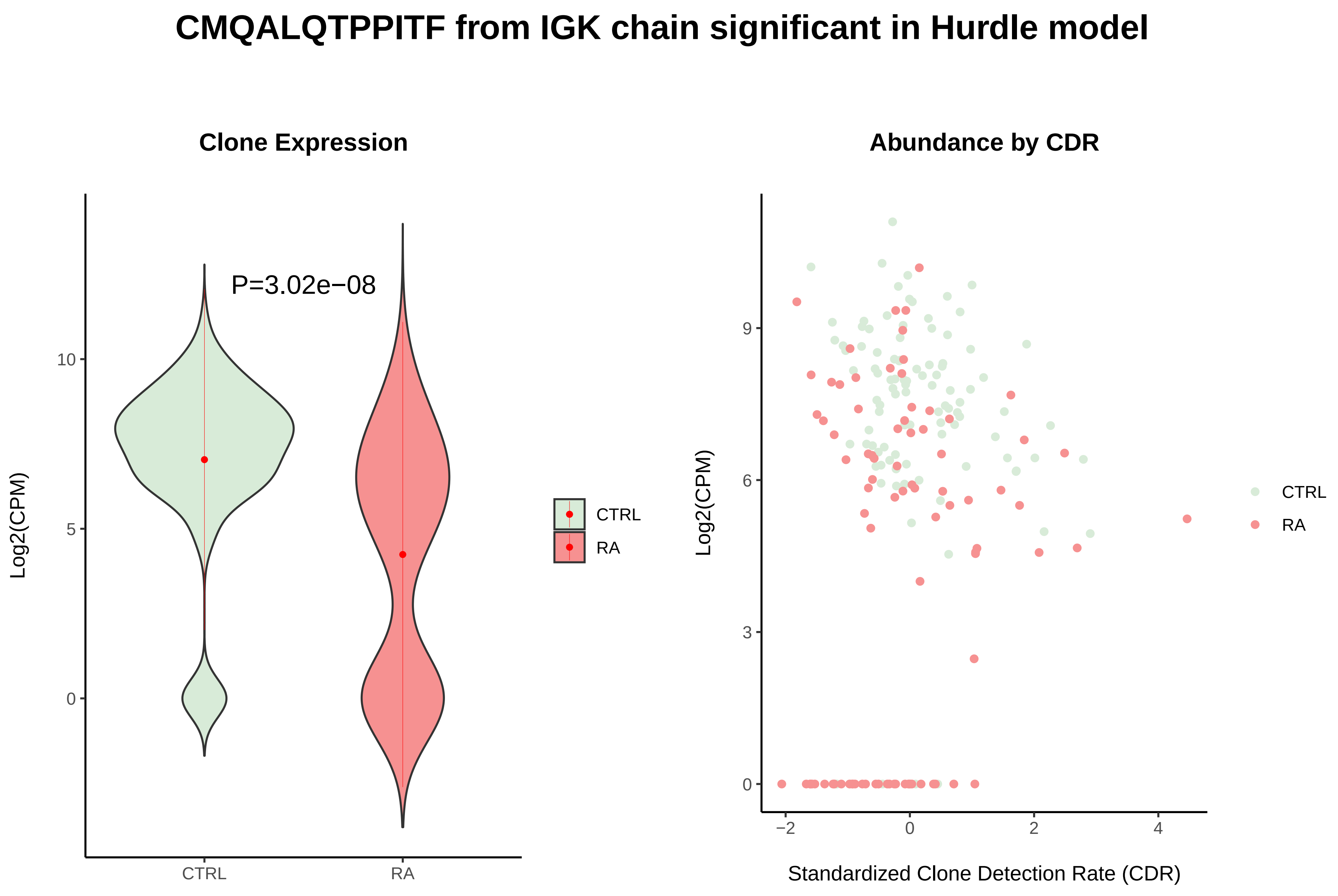
CMQALQTPPITF from IGK chain significant in Hurdle model
Clone Expression
P=3.02e−08
Log2(CPM)
10
5
0
CTRL
RA
CTRL
RA
Abundance by CDR
Log2(CPM)
9
6
3
0
−2
0
2
4
Standardized Clone Detection Rate (CDR)
CTRL
RA

CMQALQTPPLTF from IGK chain significant in Hurdle model

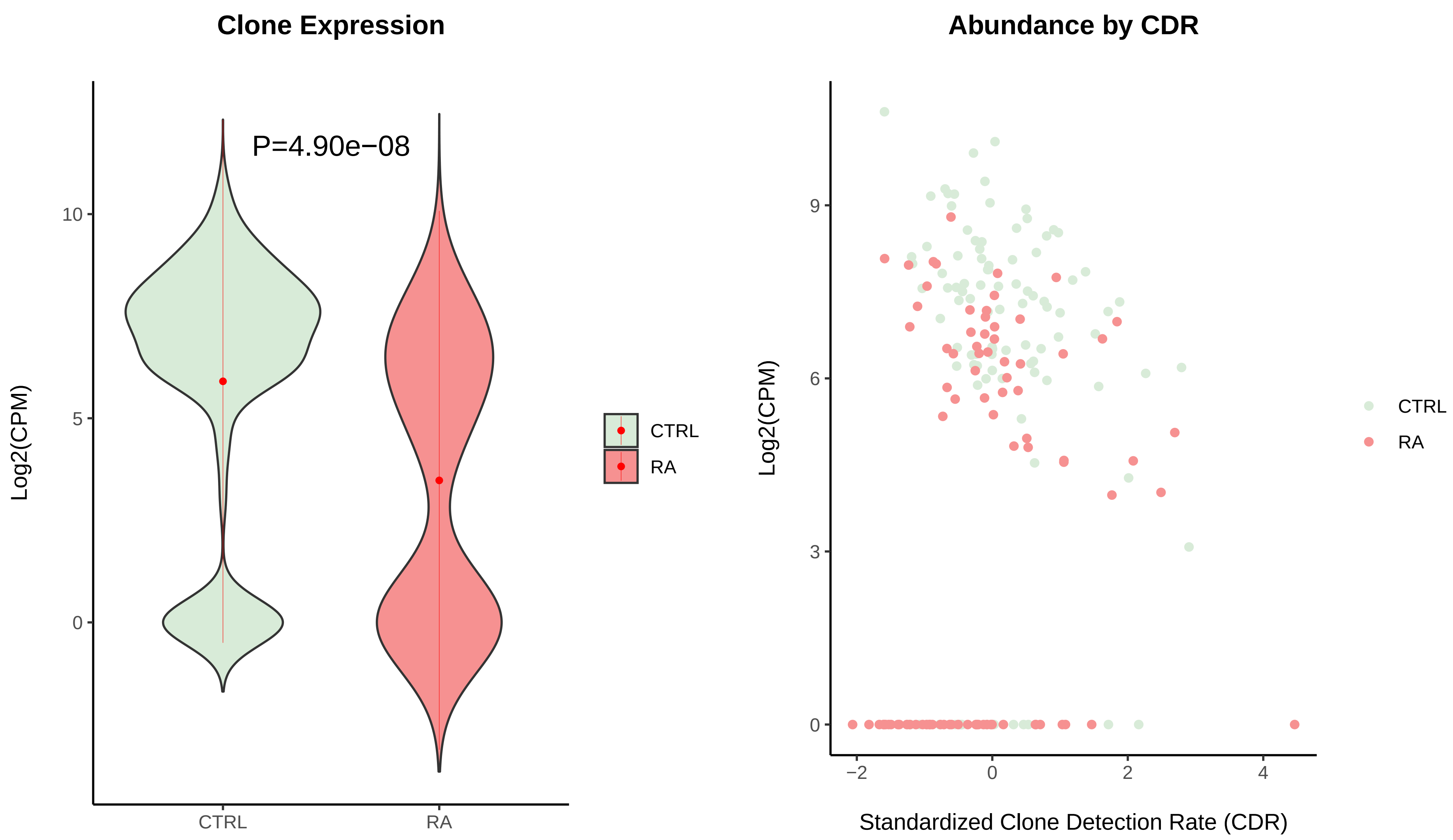

CMQALQTPPPTF from IGK chain significant in Hurdle model

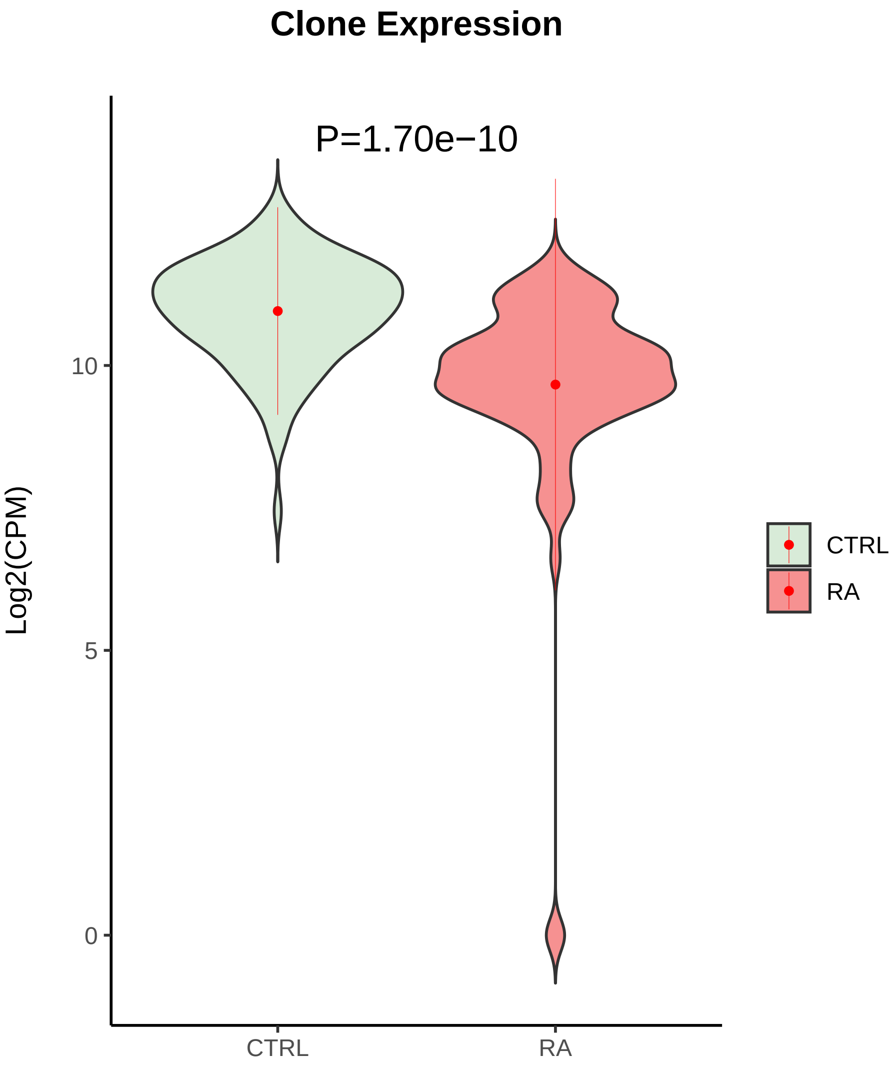

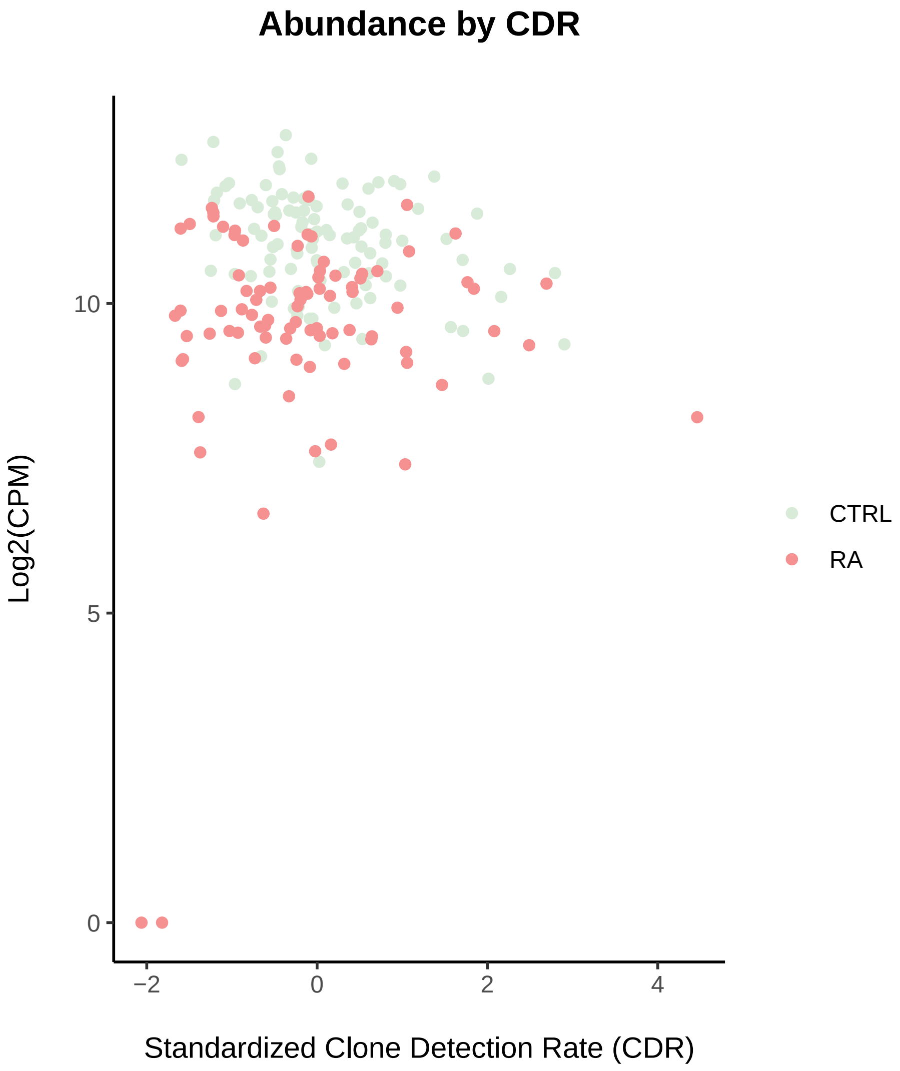

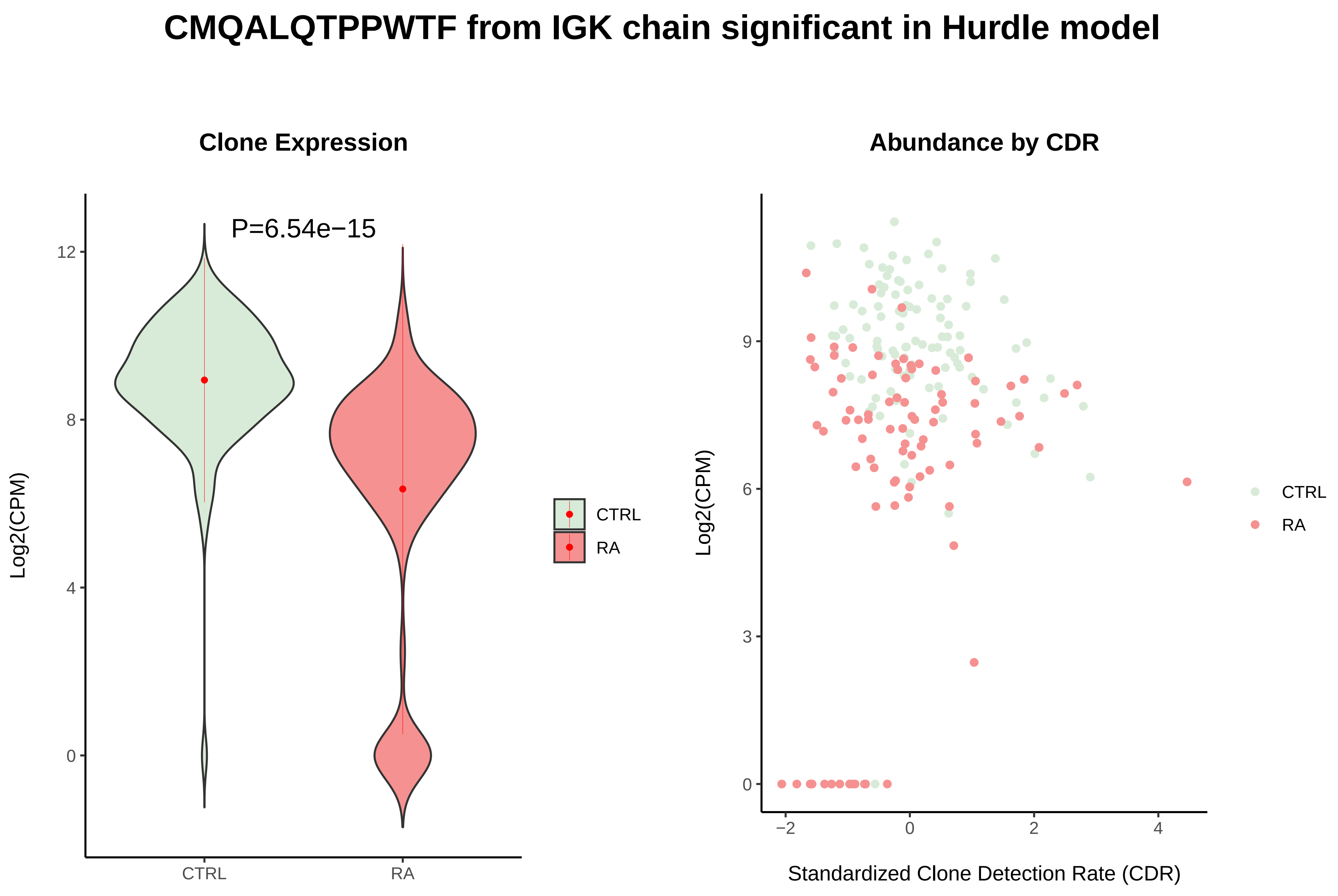
CMQALQTPPWTF from IGK chain significant in Hurdle model
Clone Expression
P=6.54e−15
Log2(CPM)
12
8
4
0
CTRL
RA
CTRL
RA
Abundance by CDR
Log2(CPM)
9
6
3
0
−2
0
2
4
Standardized Clone Detection Rate (CDR)
CTRL
RA

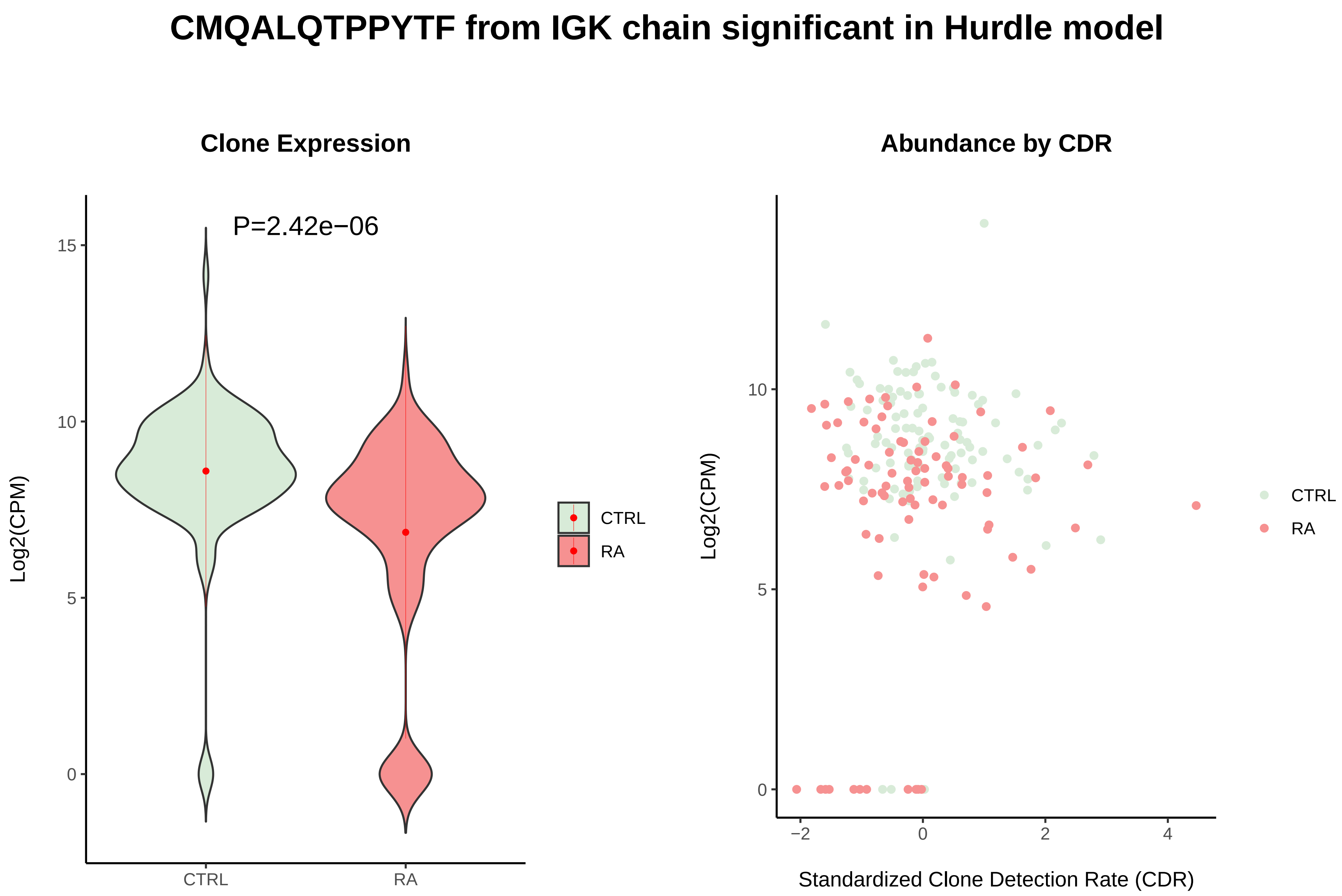

CMQALQTPPYTF from IGK chain significant in Hurdle model
Clone Expression
P=2.42e−06
Log2(CPM)
15
10
5
0
CTRL
RA
CTRL
RA
Abundance by CDR
Log2(CPM)
10
5
0
−2
0
2
4
Standardized Clone Detection Rate (CDR)
CTRL
RA

CMQALQTPQTF from IGK chain significant in Hurdle model

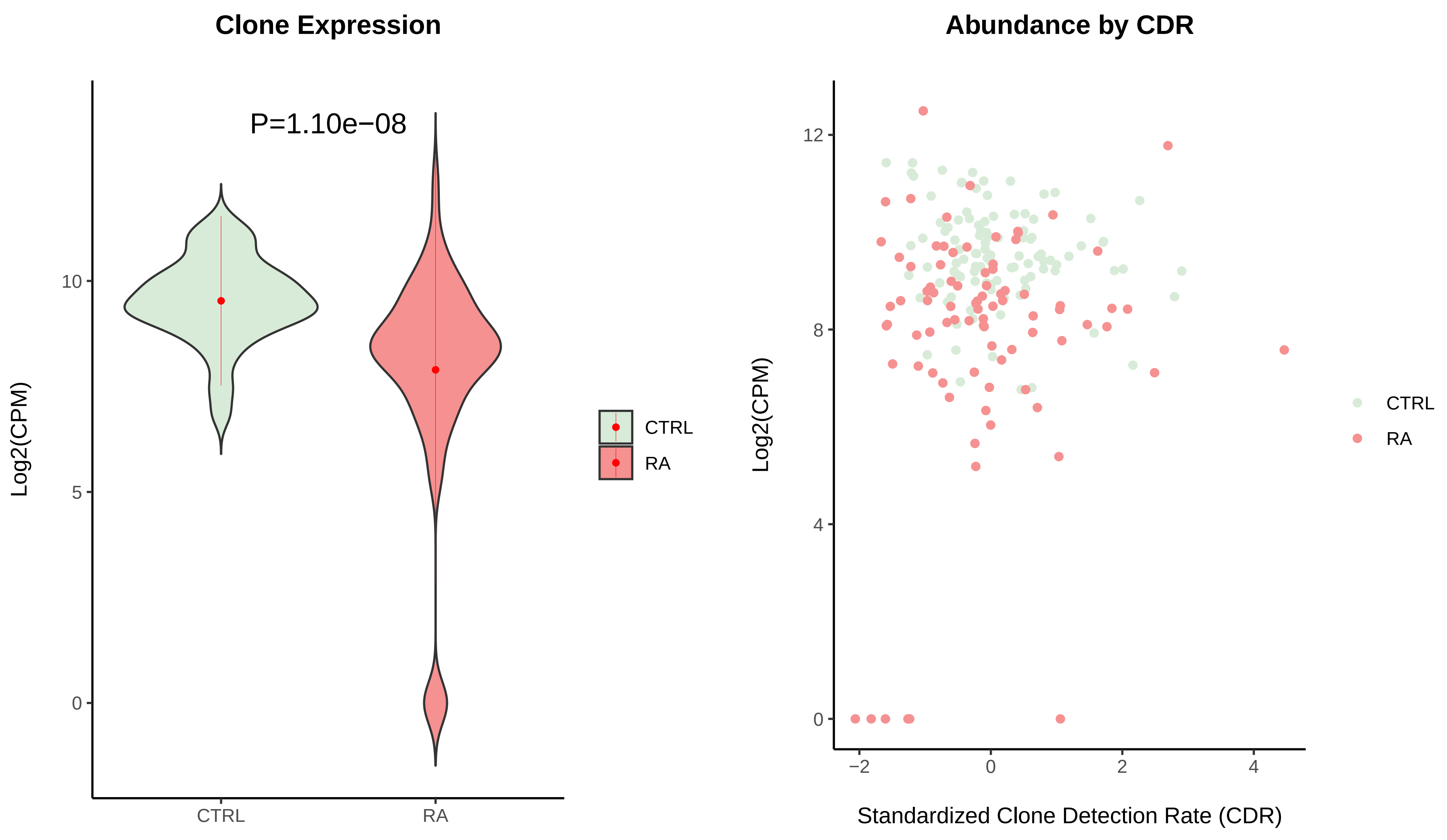

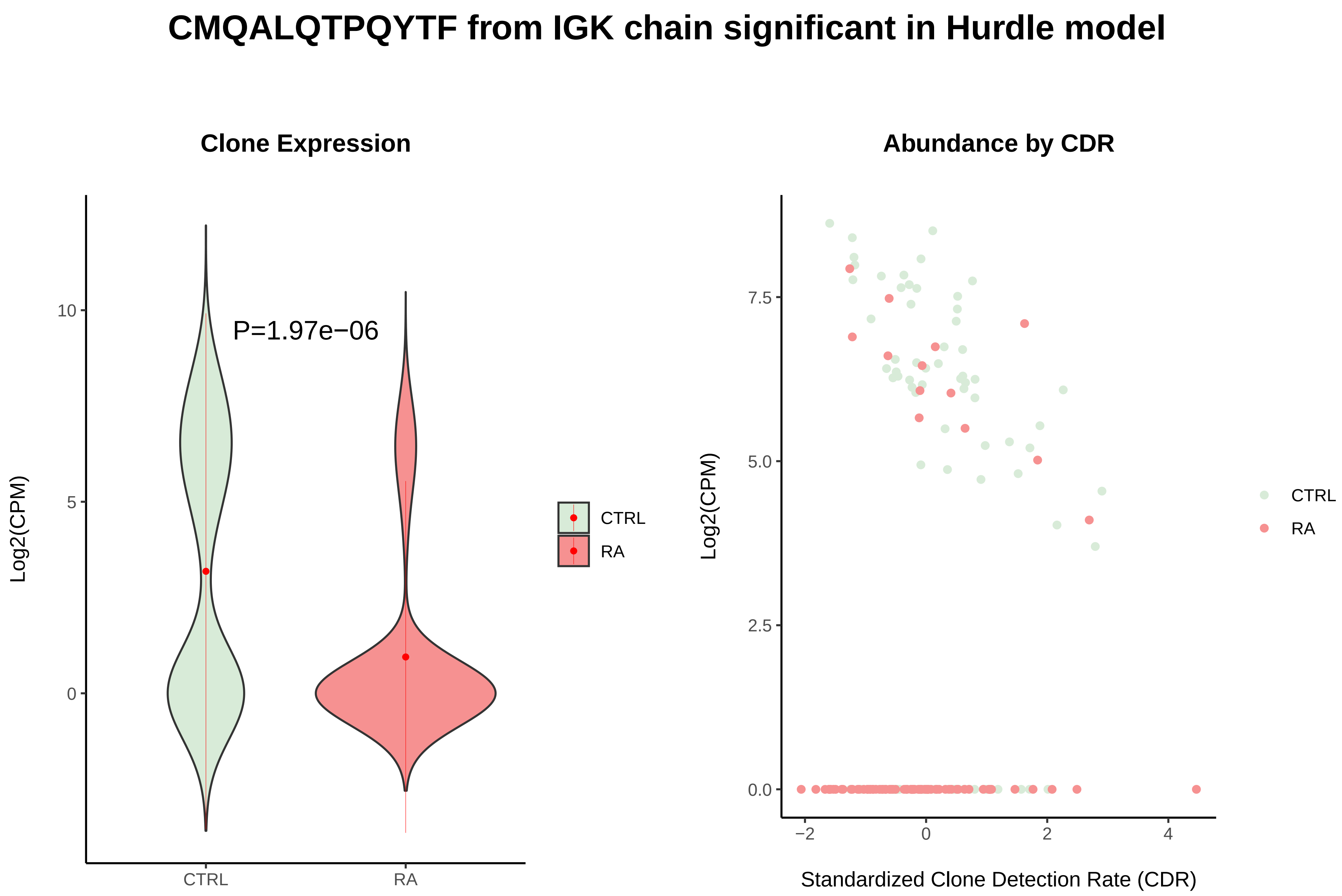
CMQALQTPQYTF from IGK chain significant in Hurdle model
Clone Expression
Log2(CPM)
10
5
0
P=1.97e−06
CTRL
RA
CTRL
RA
Abundance by CDR
Log2(CPM)
7.5
5.0
2.5
0.0
−2
0
2
4
Standardized Clone Detection Rate (CDR)
CTRL
RA

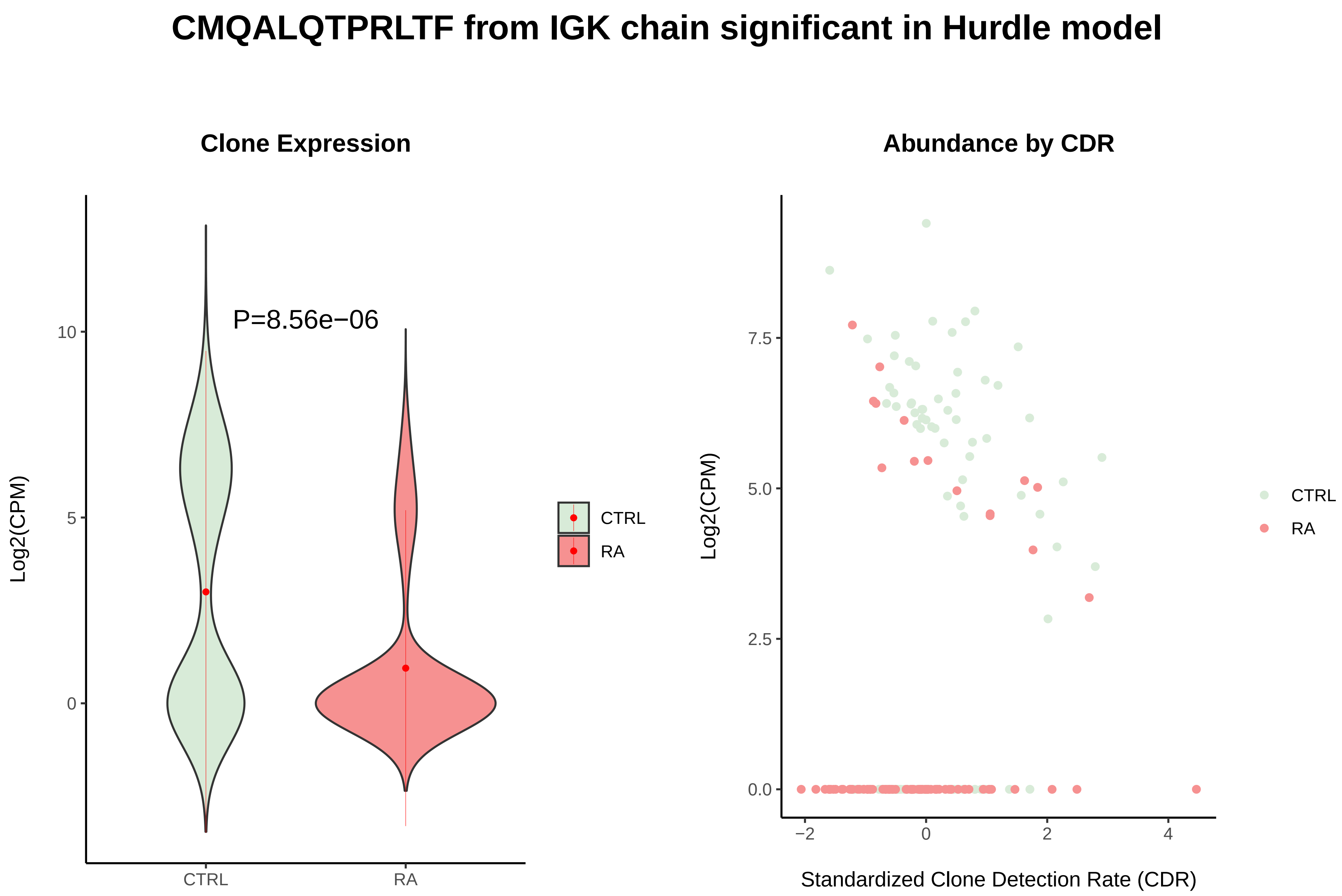

CMQALQTPRLTF from IGK chain significant in Hurdle model
Clone Expression
P=8.56e−06
Log2(CPM)
10
5
0
CTRL
RA
Abundance by CDR
Log2(CPM)
7.5
5.0
2.5
0.0
−2
0
2
4
Standardized Clone Detection Rate (CDR)
CTRL
RA

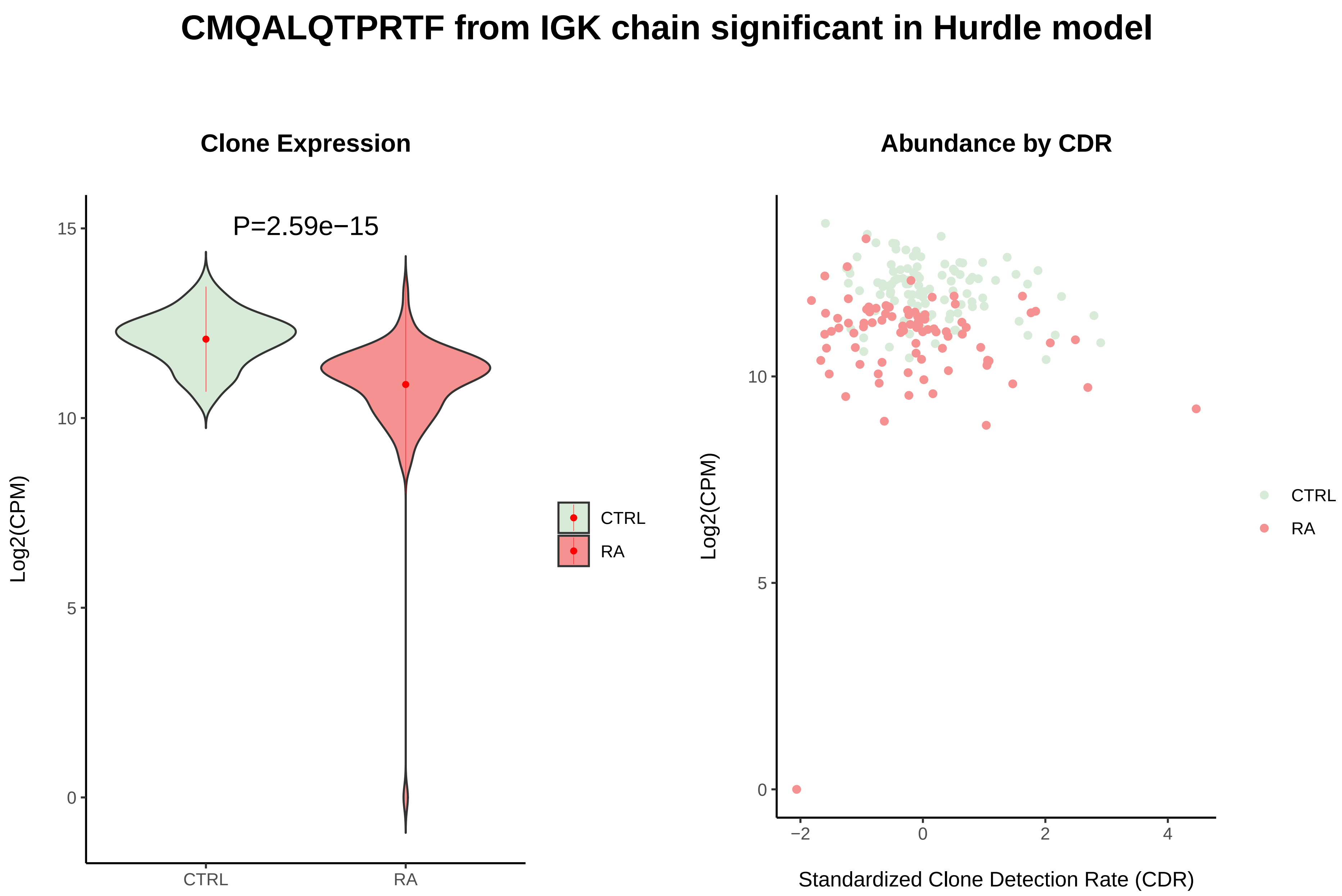

CMQALQTPRTF from IGK chain significant in Hurdle model
Clone Expression
P=2.59e−15
Log2(CPM)
15
10
5
0
CTRL
RA
Abundance by CDR
Log2(CPM)
10
5
0
−2
0
2
4
Standardized Clone Detection Rate (CDR)
CTRL
RA

CMQALQTPRYTF from IGK chain significant in Hurdle model

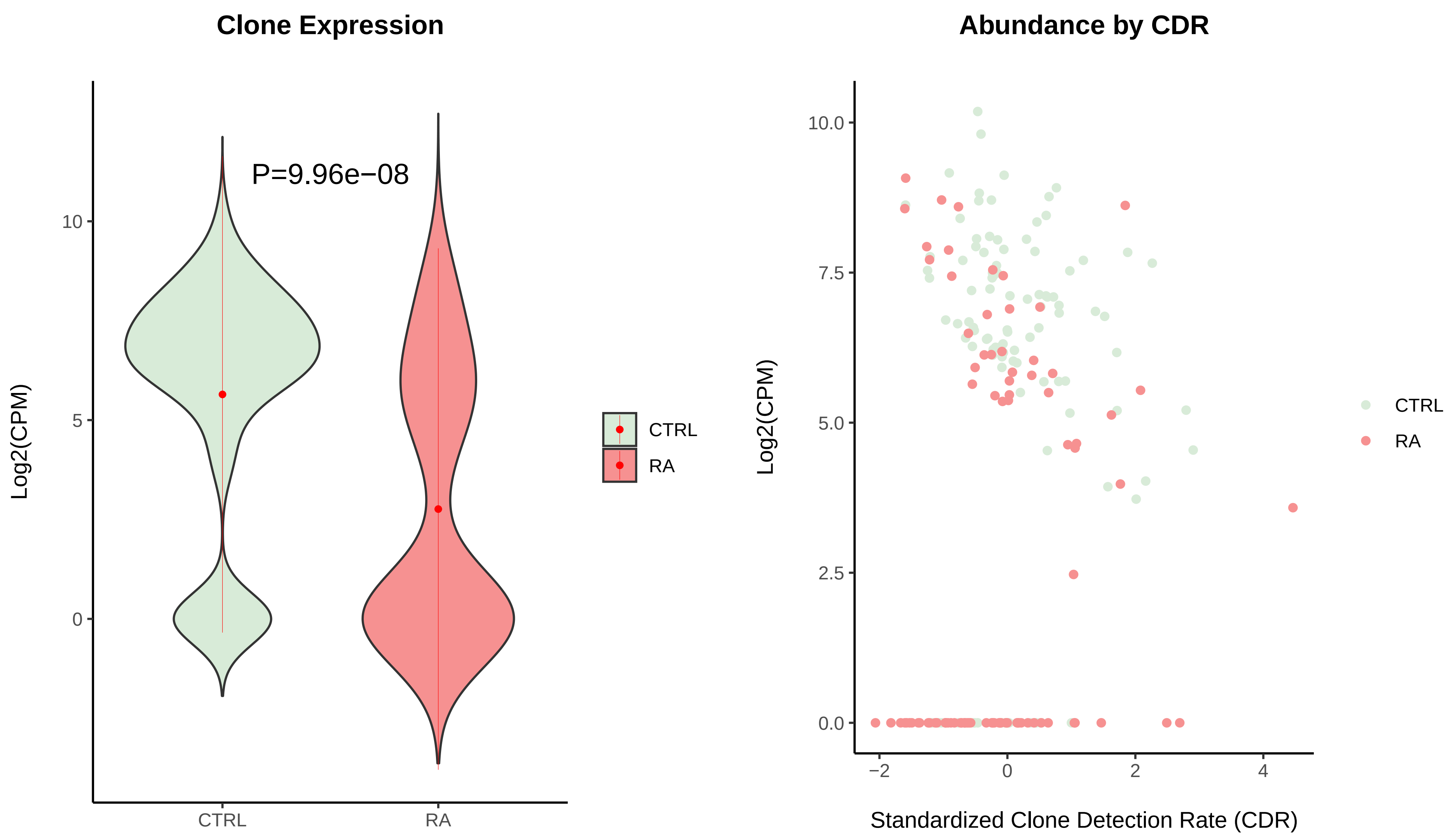

CMQALQTPSITF from IGK chain significant in Hurdle model

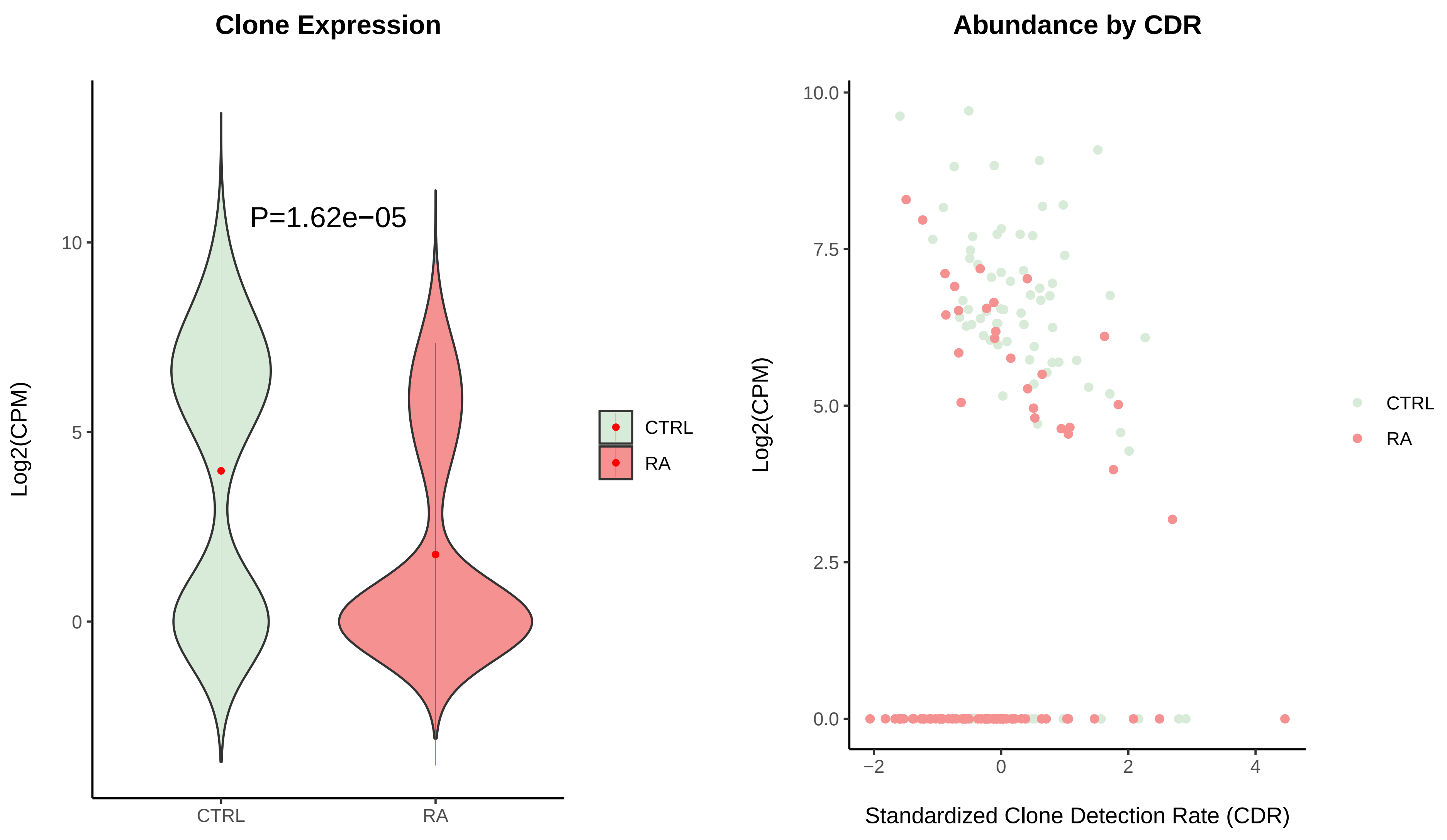

CMQALQTPTF from IGK chain significant in Hurdle model

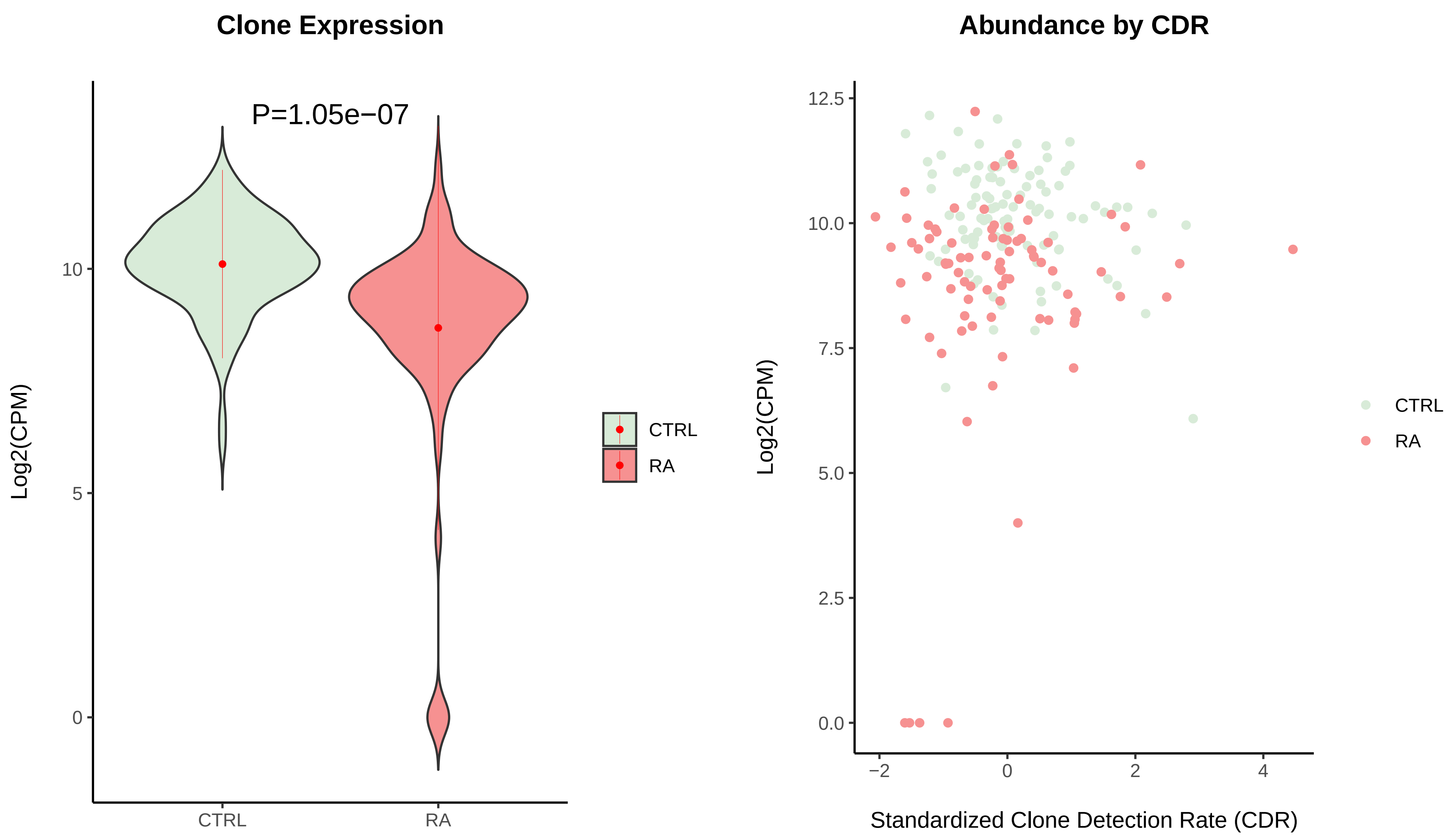

CMQALQTPVTF from IGK chain significant in Hurdle model

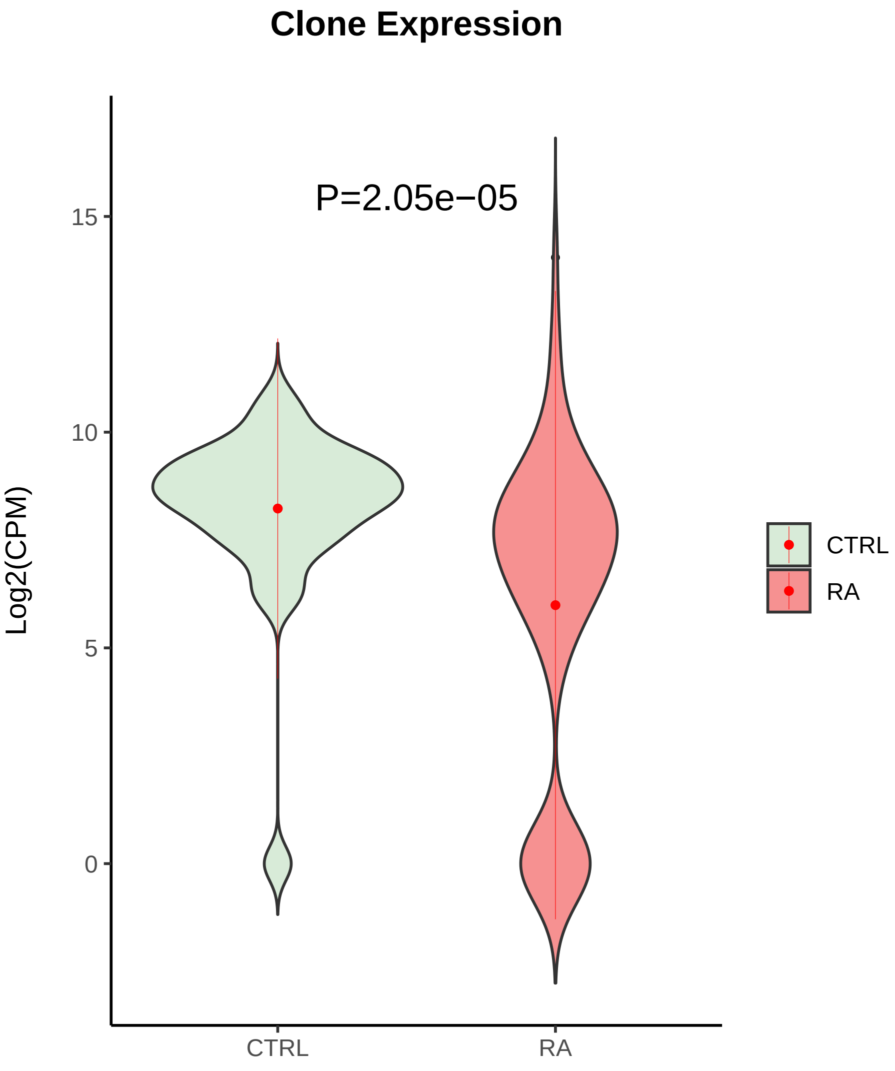

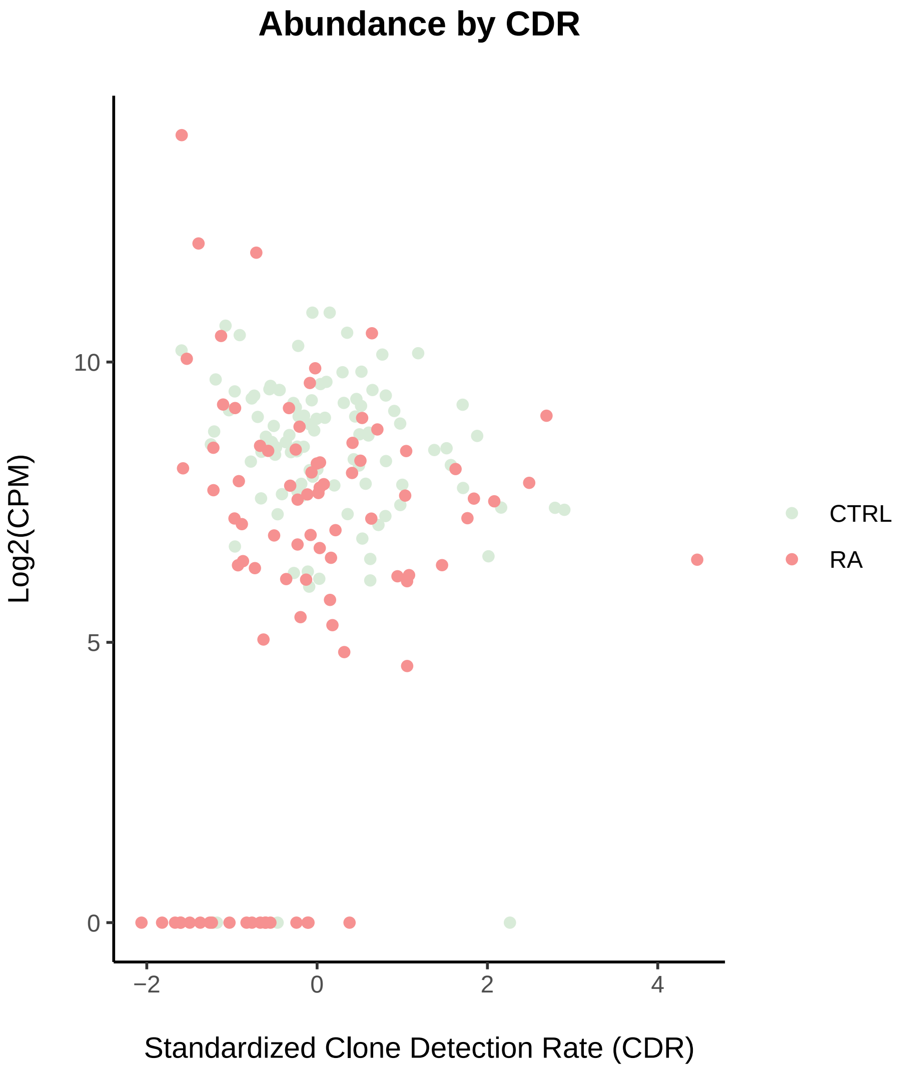

CMQALQTPWTF from IGK chain significant in Hurdle model

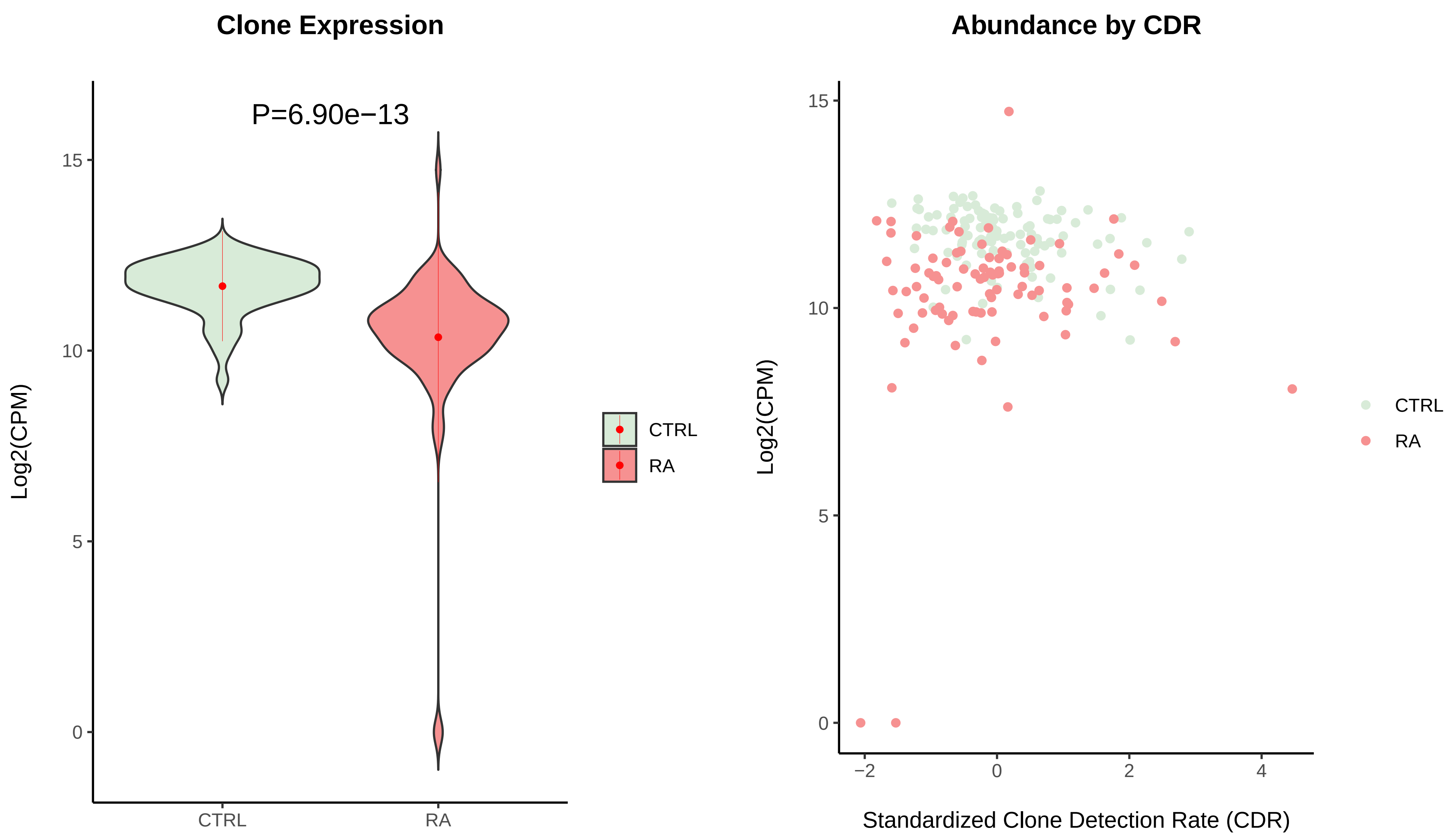

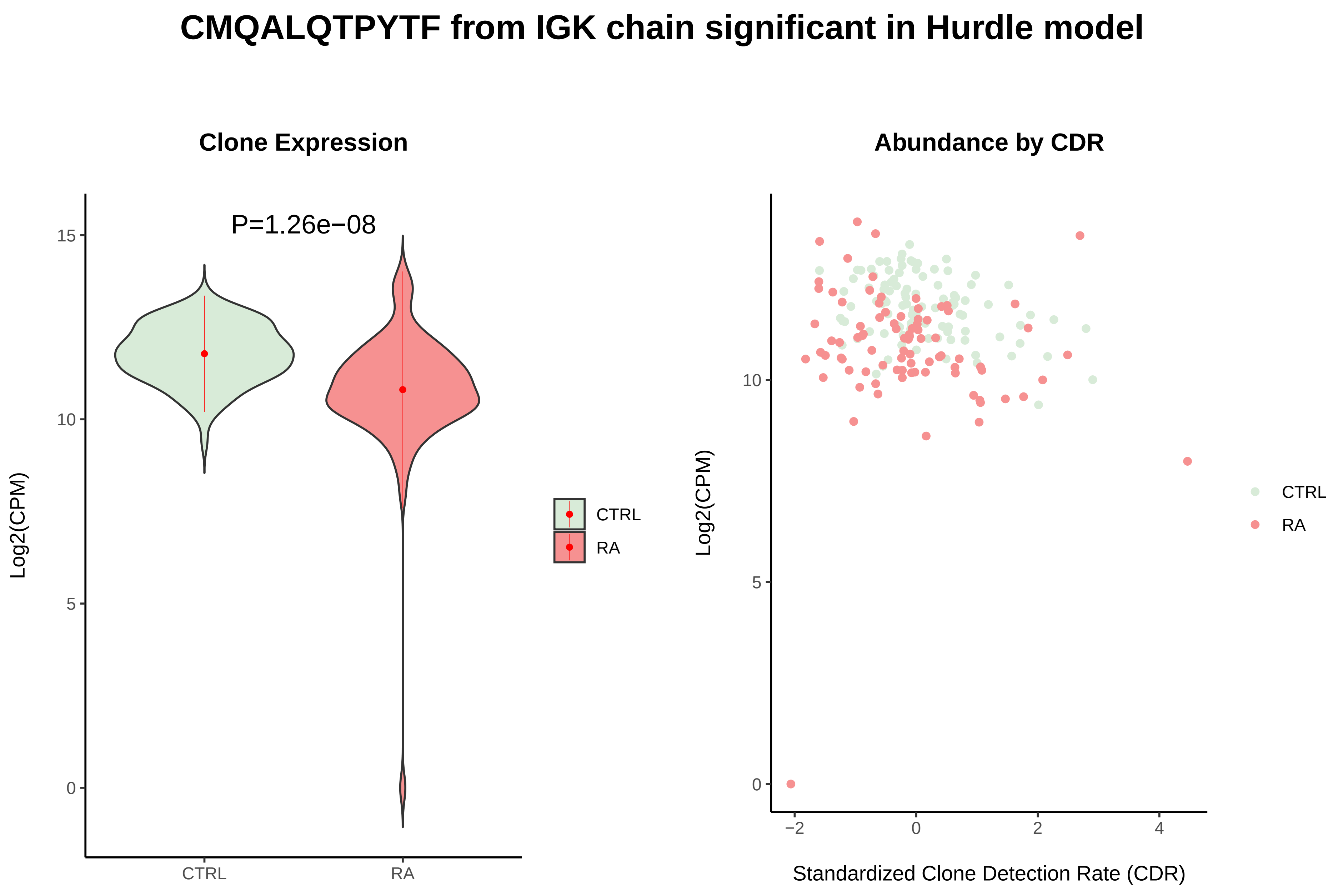

CMQALQTPYTF from IGK chain significant in Hurdle model
Clone Expression
P=1.26e−08
Log2(CPM)
15
10
5
0
CTRL
RA
CTRL
RA
Abundance by CDR
Log2(CPM)
10
5
0
−2
0
2
4
Standardized Clone Detection Rate (CDR)
CTRL
RA

CMQALQTRTF from IGK chain significant in Hurdle model

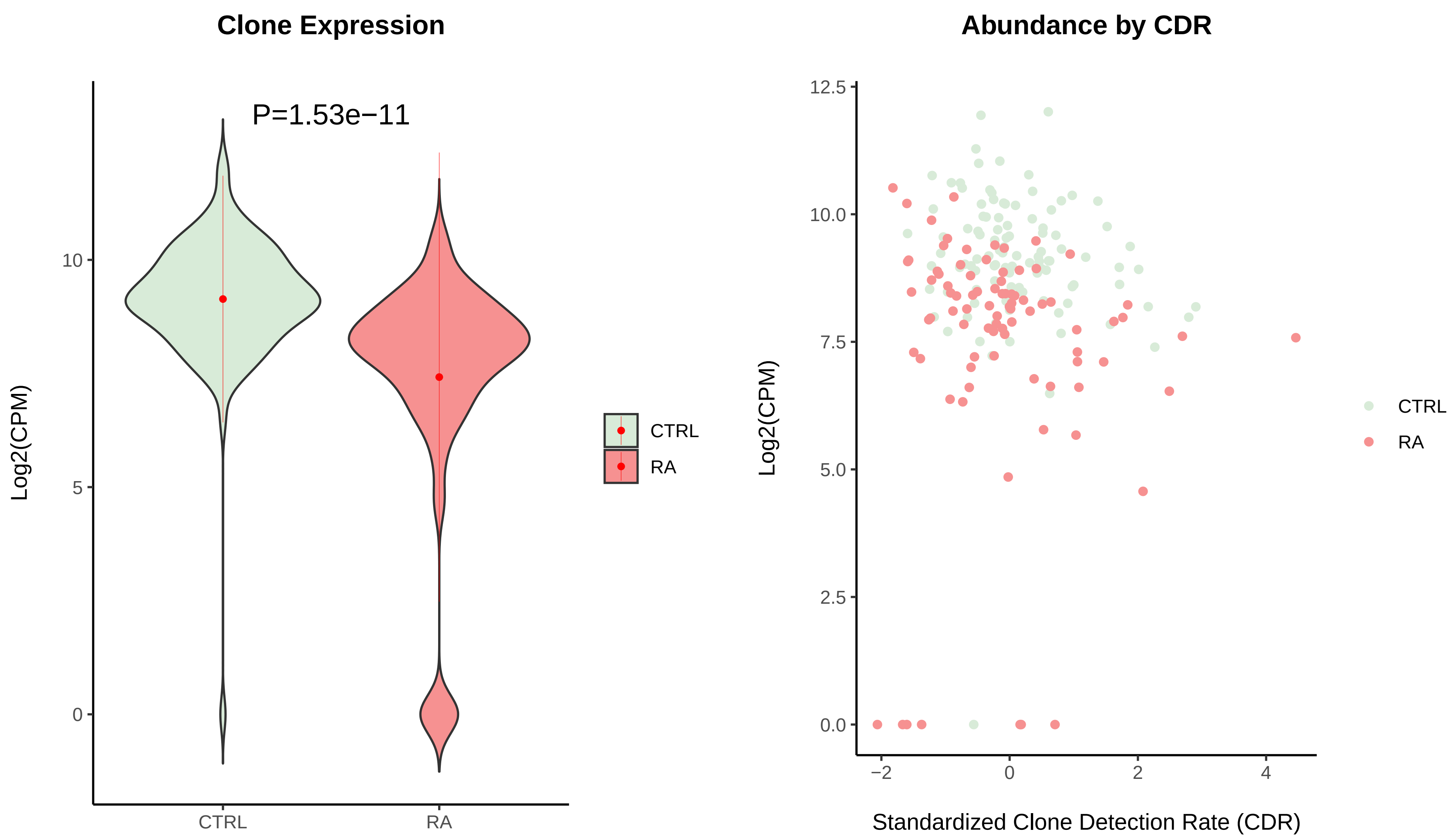

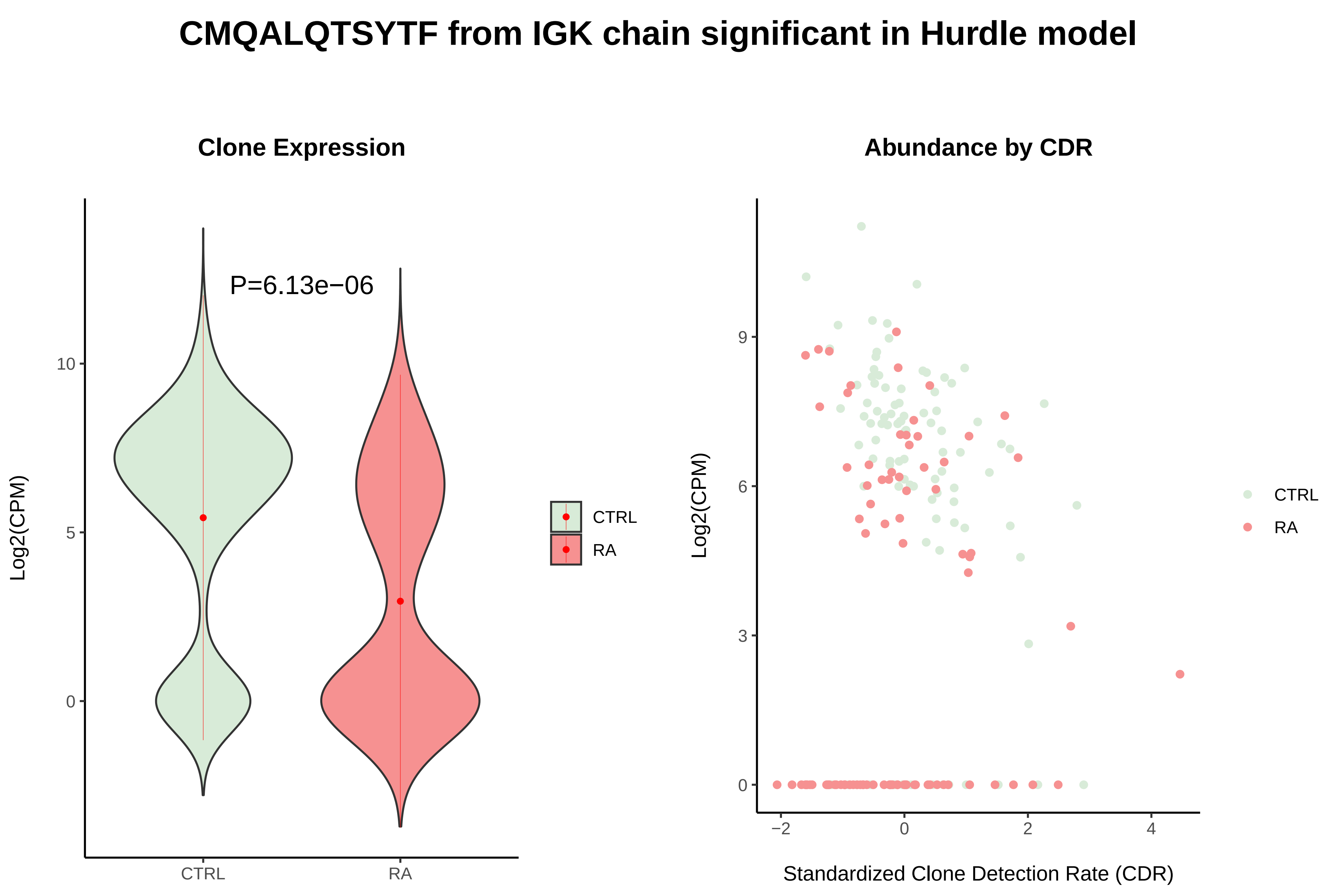
CMQALQTSYTF from IGK chain significant in Hurdle model
Clone Expression
P=6.13e−06
Log2(CPM)
10
5
0
CTRL
RA
Abundance by CDR
Log2(CPM)
9
6
3
0
−2
0
2
4
Standardized Clone Detection Rate (CDR)

CMQALQTWTF from IGK chain significant in Hurdle model

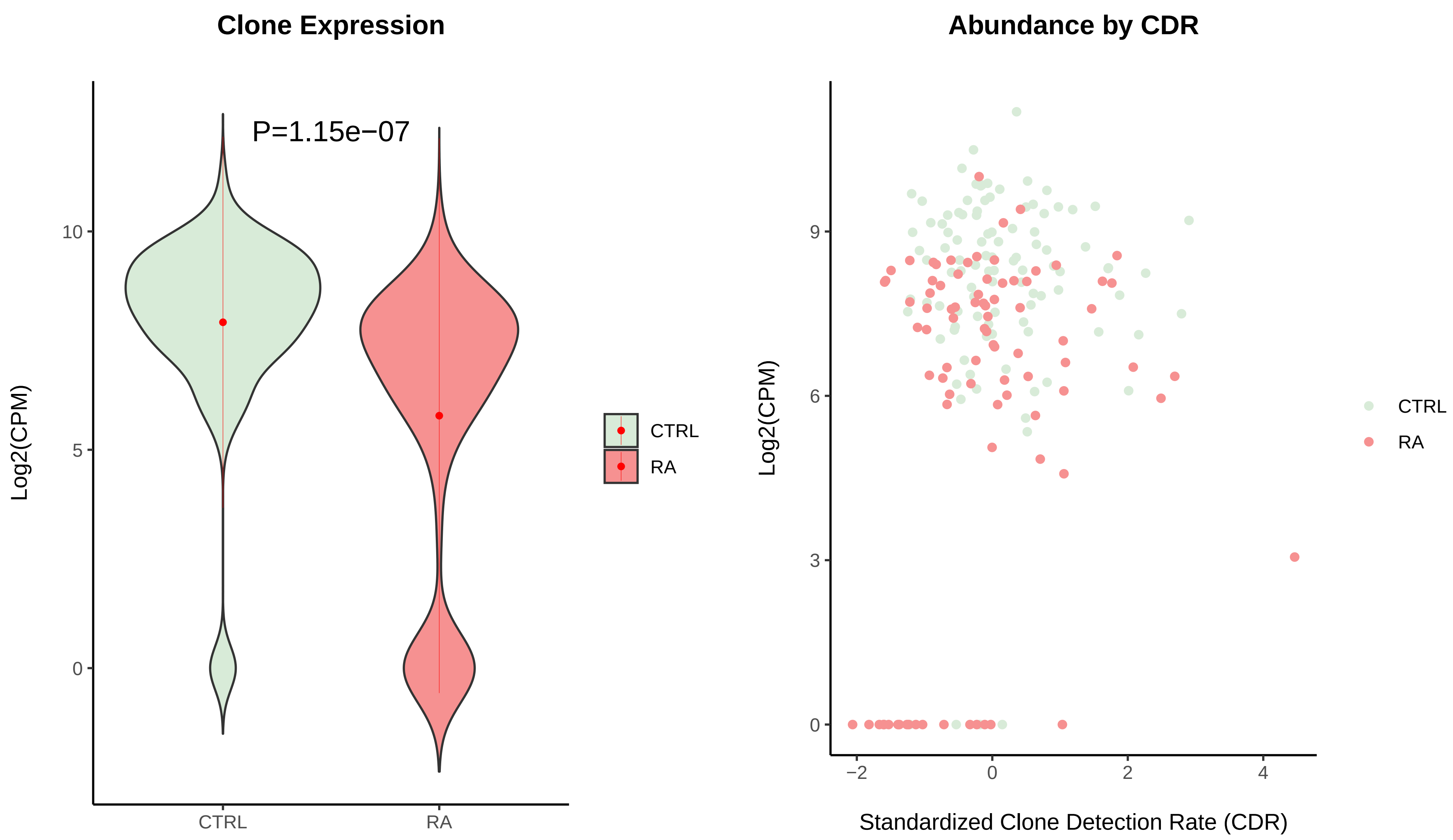

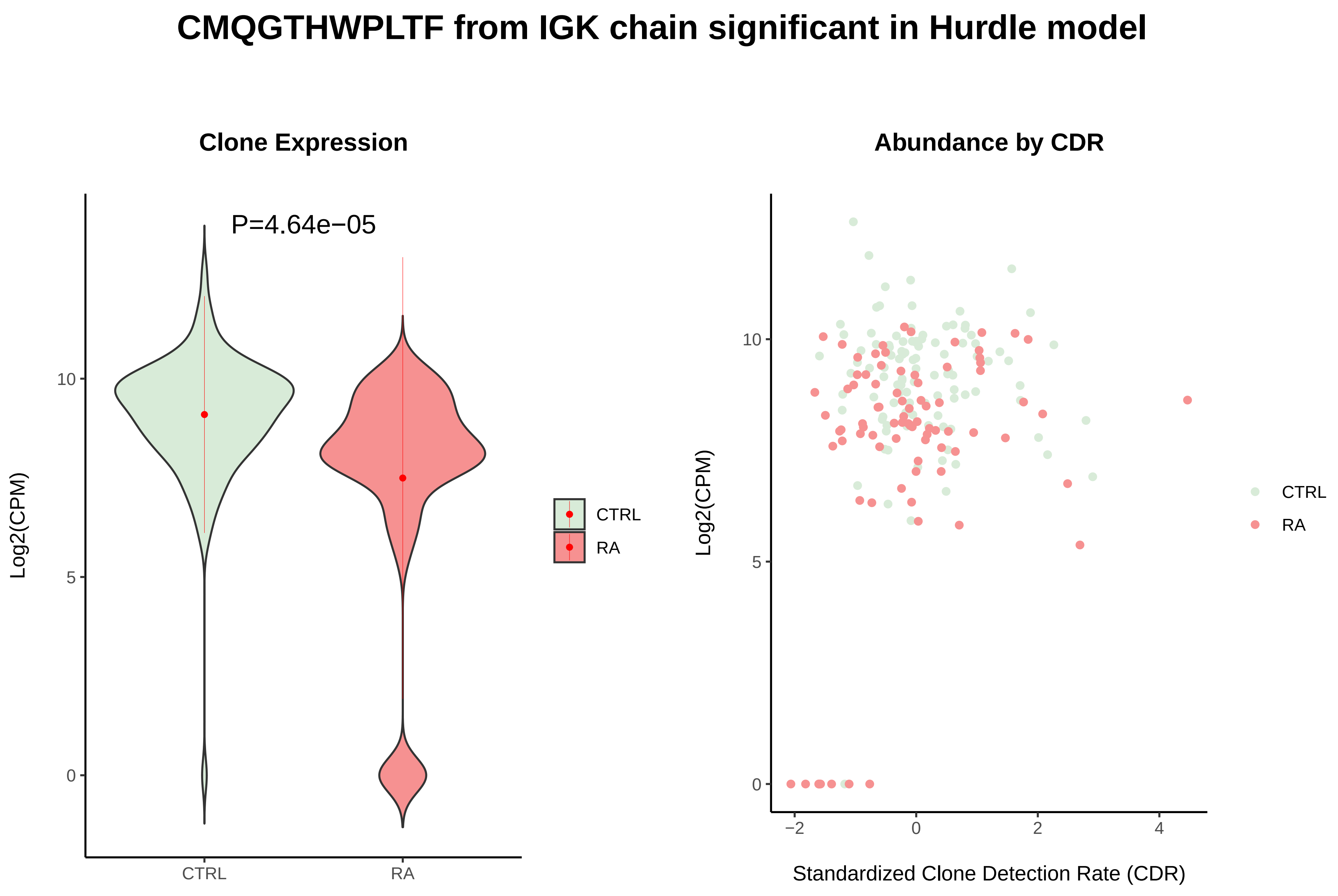

CMQGTHWPLTF from IGK chain significant in Hurdle model
Clone Expression
P=4.64e−05
Log2(CPM)
10
5
0
CTRL
RA
CTRL
RA
Abundance by CDR
Log2(CPM)
10
5
0
−2
0
2
4
Standardized Clone Detection Rate (CDR)
CTRL
RA

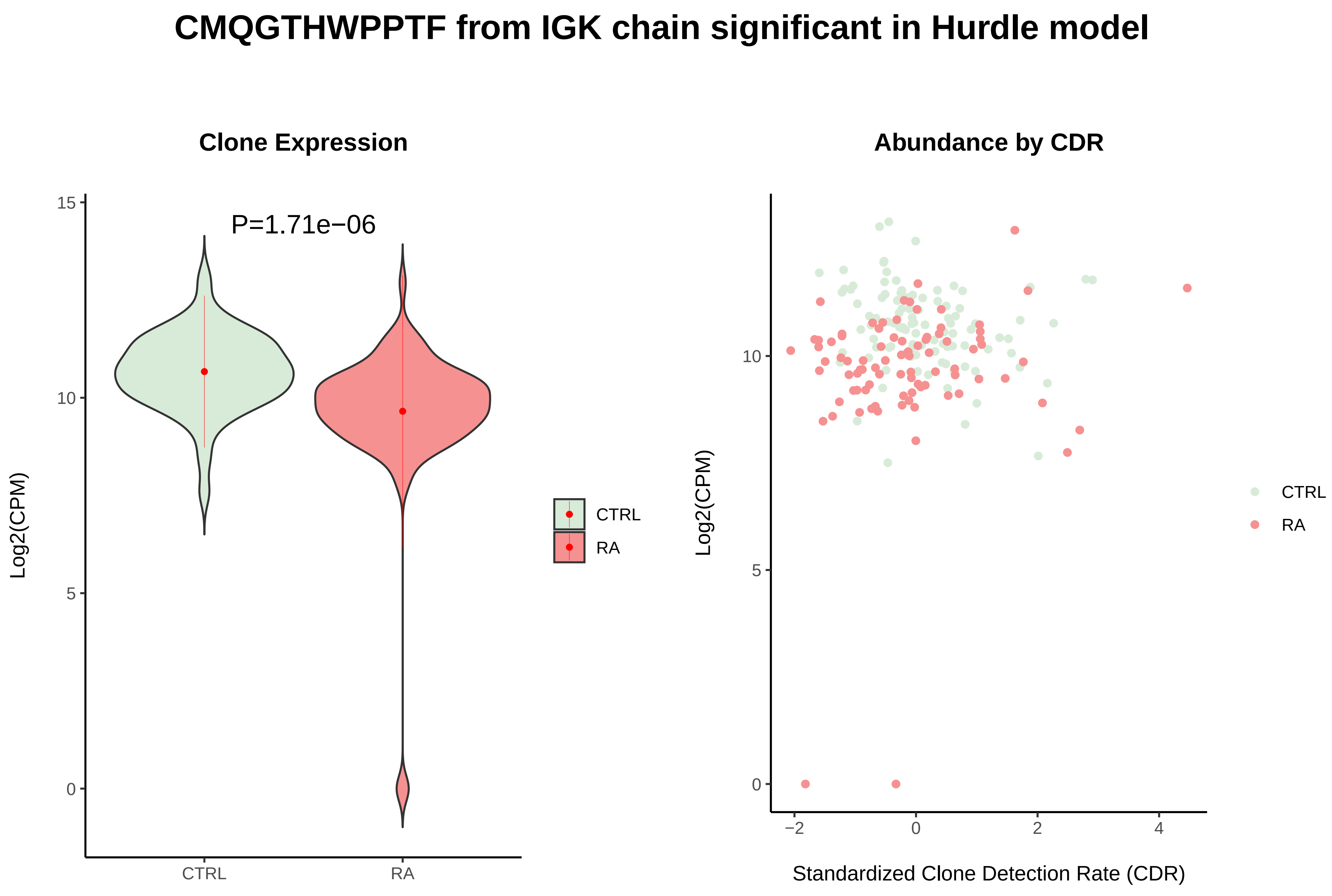
CMQGTHWPPTF from IGK chain significant in Hurdle model
Clone Expression
P=1.71e−06
Log2(CPM)
15
10
5
0
CTRL
RA
CTRL
RA
Abundance by CDR
Log2(CPM)
10
5
0
−2
0
2
4
Standardized Clone Detection Rate (CDR)
CTRL
RA

CMQGTHWPRTF from IGK chain significant in Hurdle model

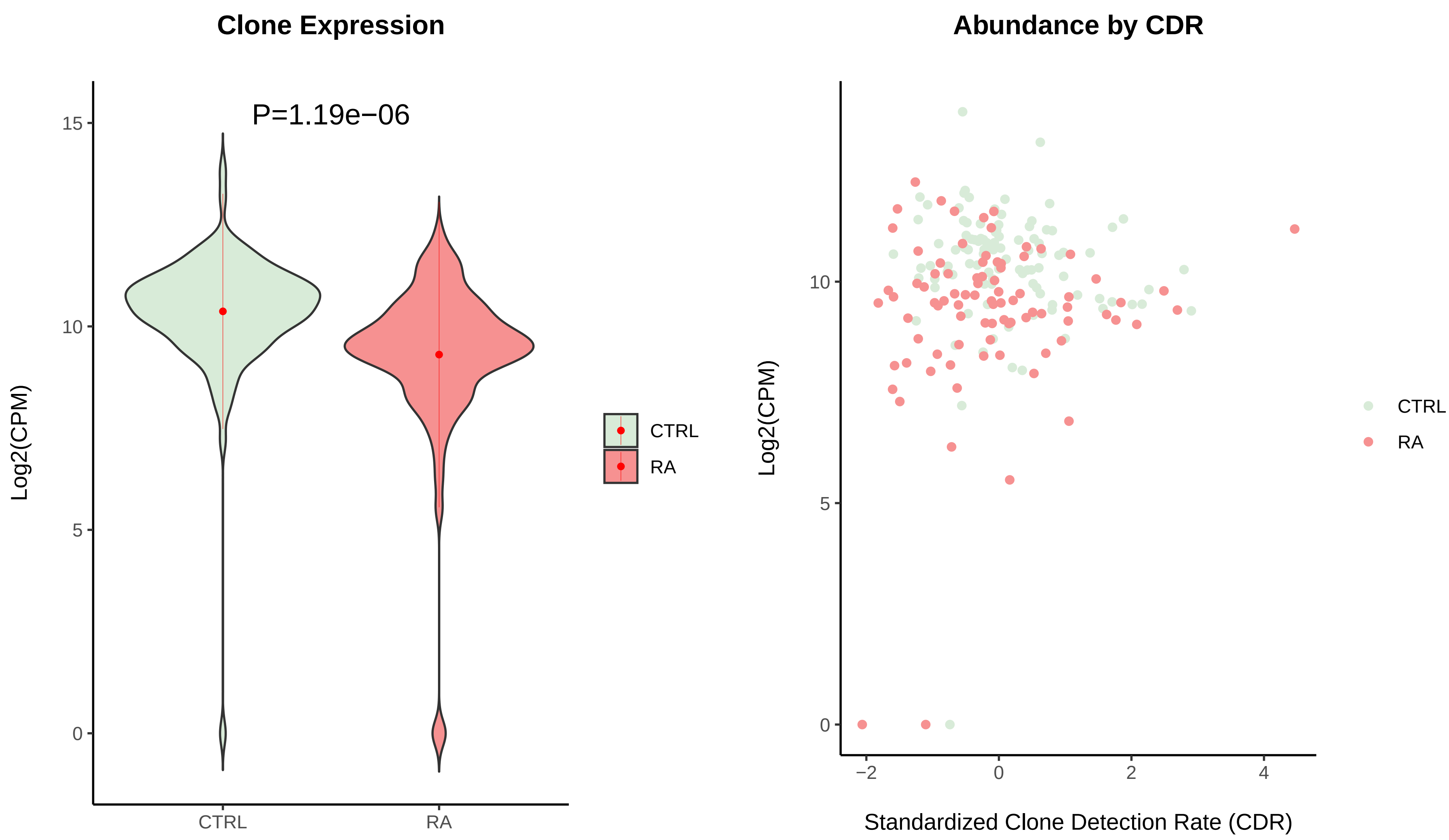

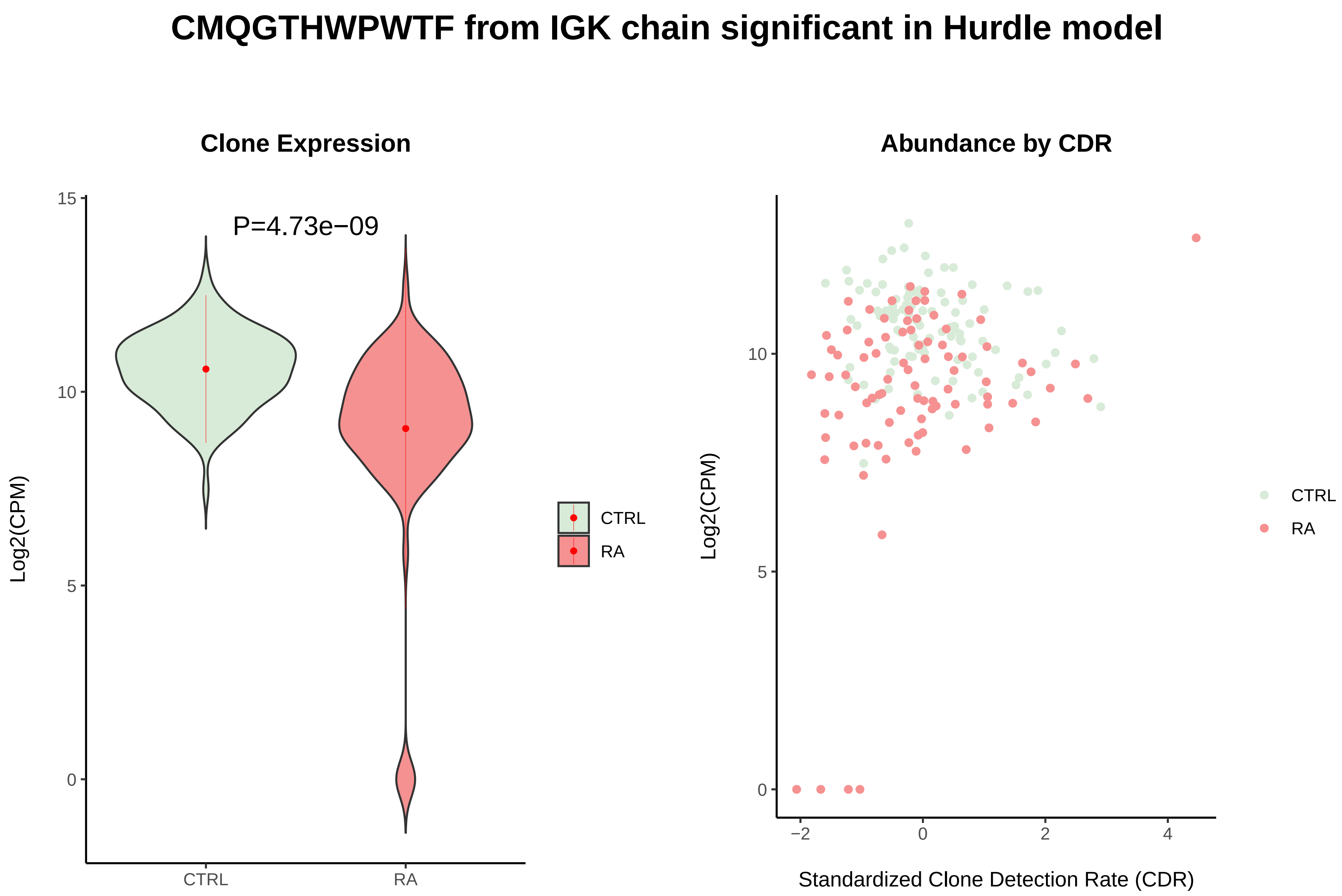
CMQGTHWPWTF from IGK chain significant in Hurdle model
Clone Expression
P=4.73e−09
Log2(CPM)
15
10
5
0
CTRL
RA
CTRL
RA
Abundance by CDR
Log2(CPM)
10
5
0
−2
0
2
4
Standardized Clone Detection Rate (CDR)
CTRL
RA

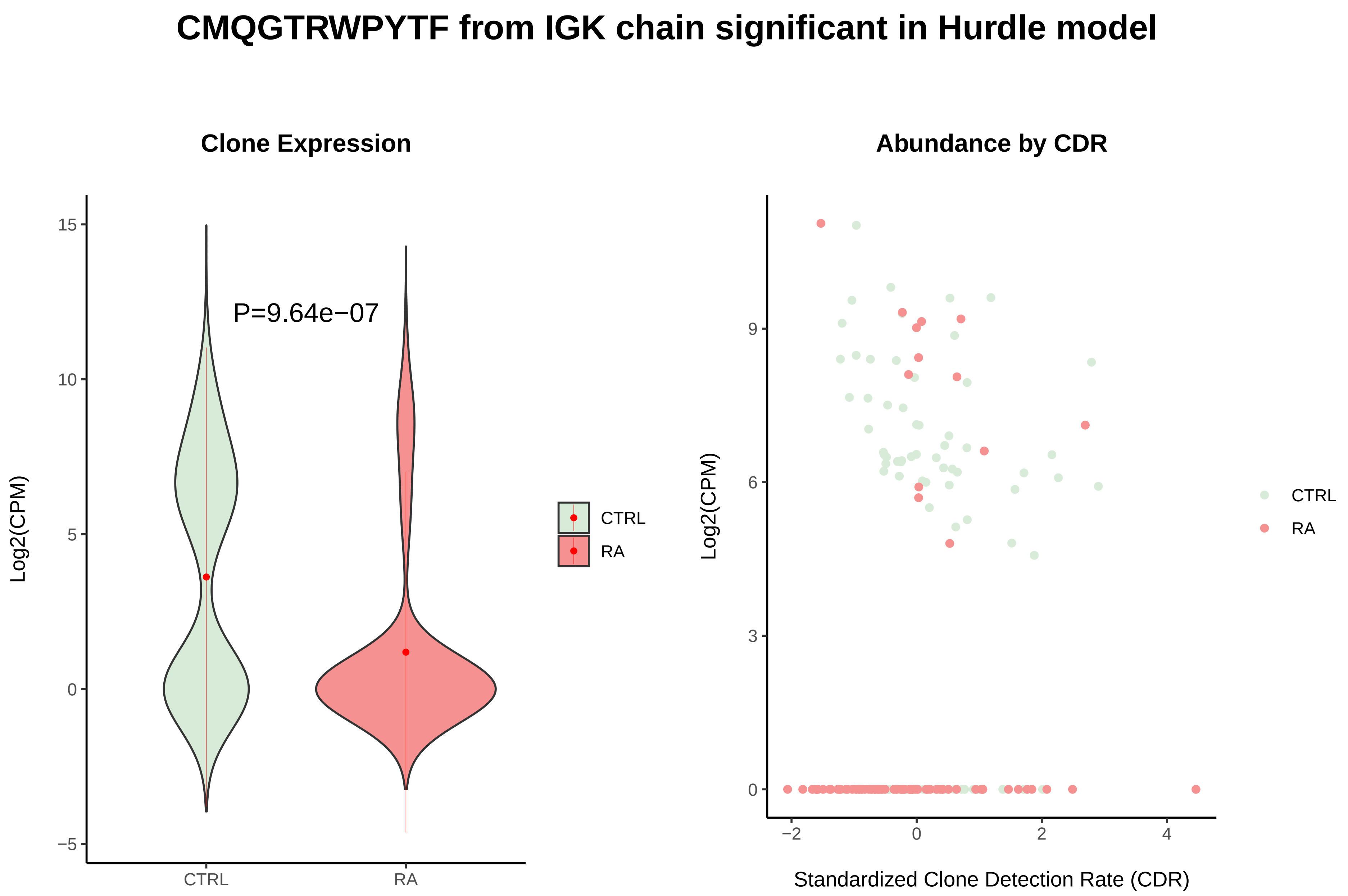

CMQGTRWPYTF from IGK chain significant in Hurdle model
Clone Expression
P=9.64e−07
Log2(CPM)
15
10
5
0
−5
CTRL
RA
CTRL
RA
Abundance by CDR
Log2(CPM)
9
6
3
0
−2
0
2
4
Standardized Clone Detection Rate (CDR)
CTRL
RA

CMQRIEFPLTF from IGK chain significant in Hurdle model

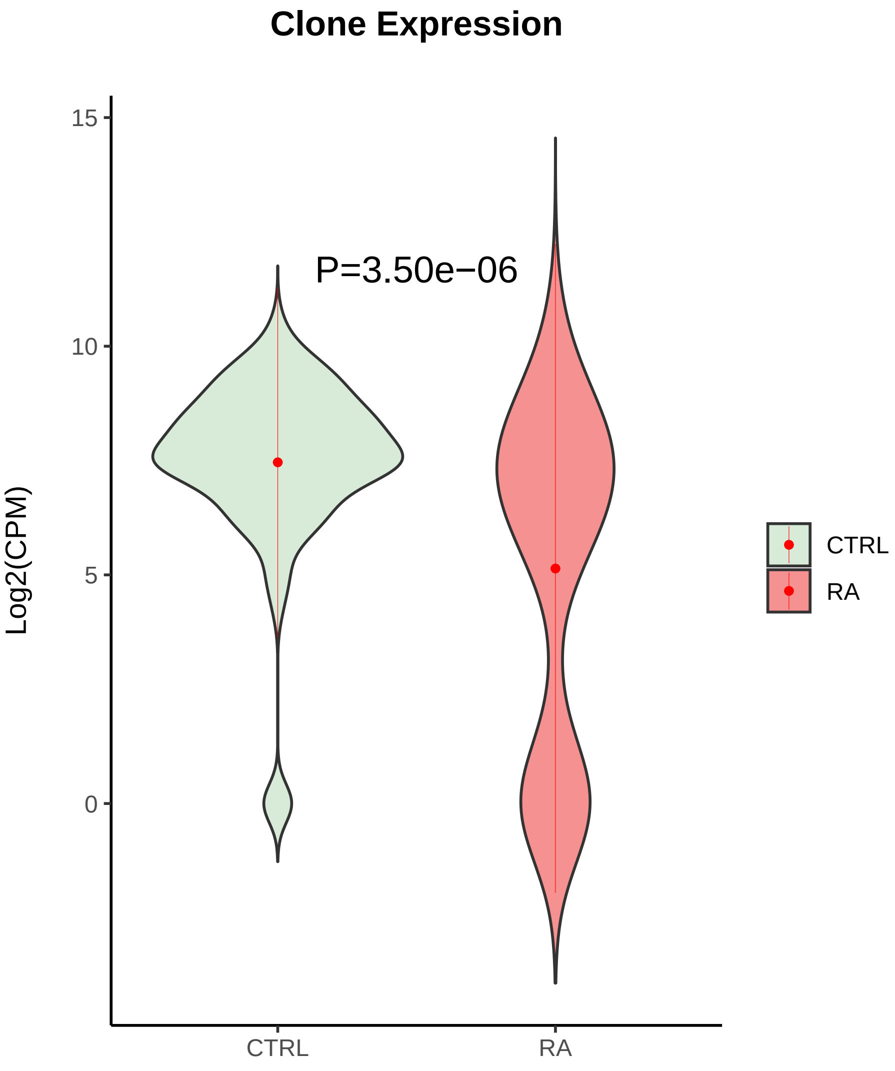

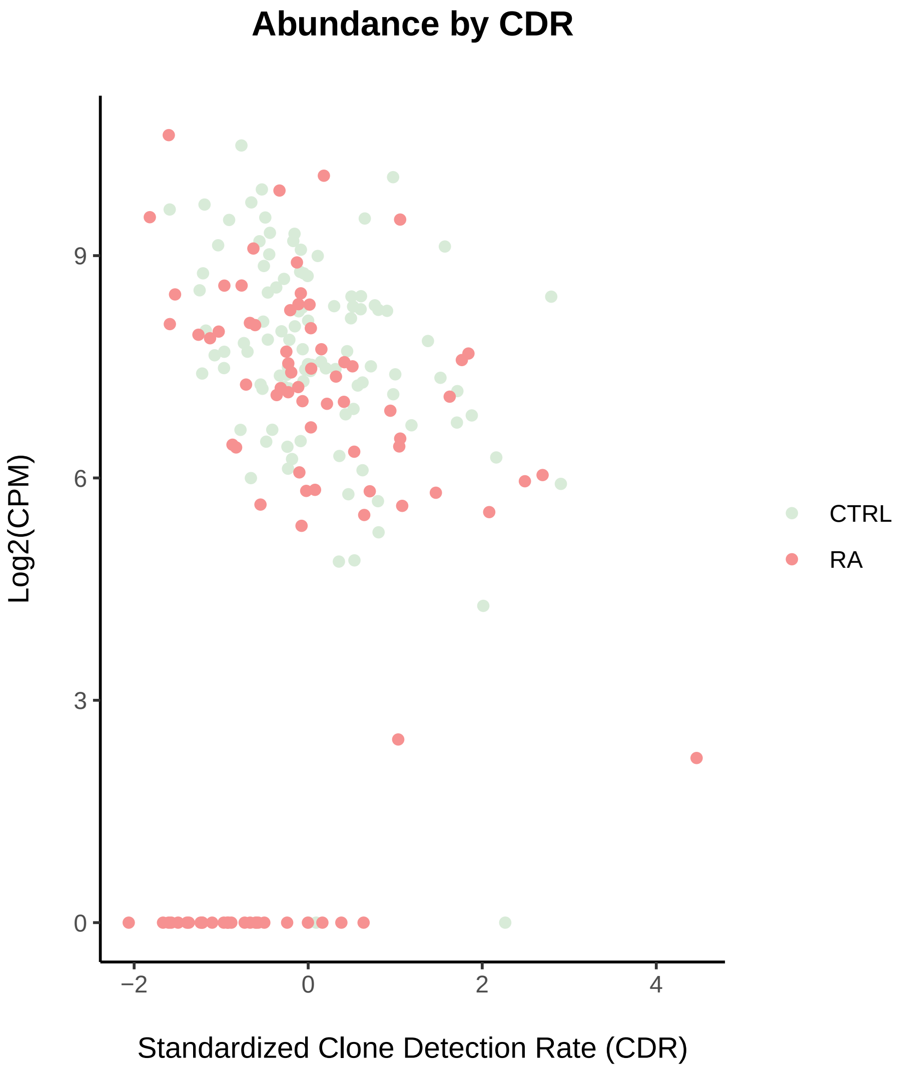

CMQRIEFPWTF from IGK chain significant in Hurdle model

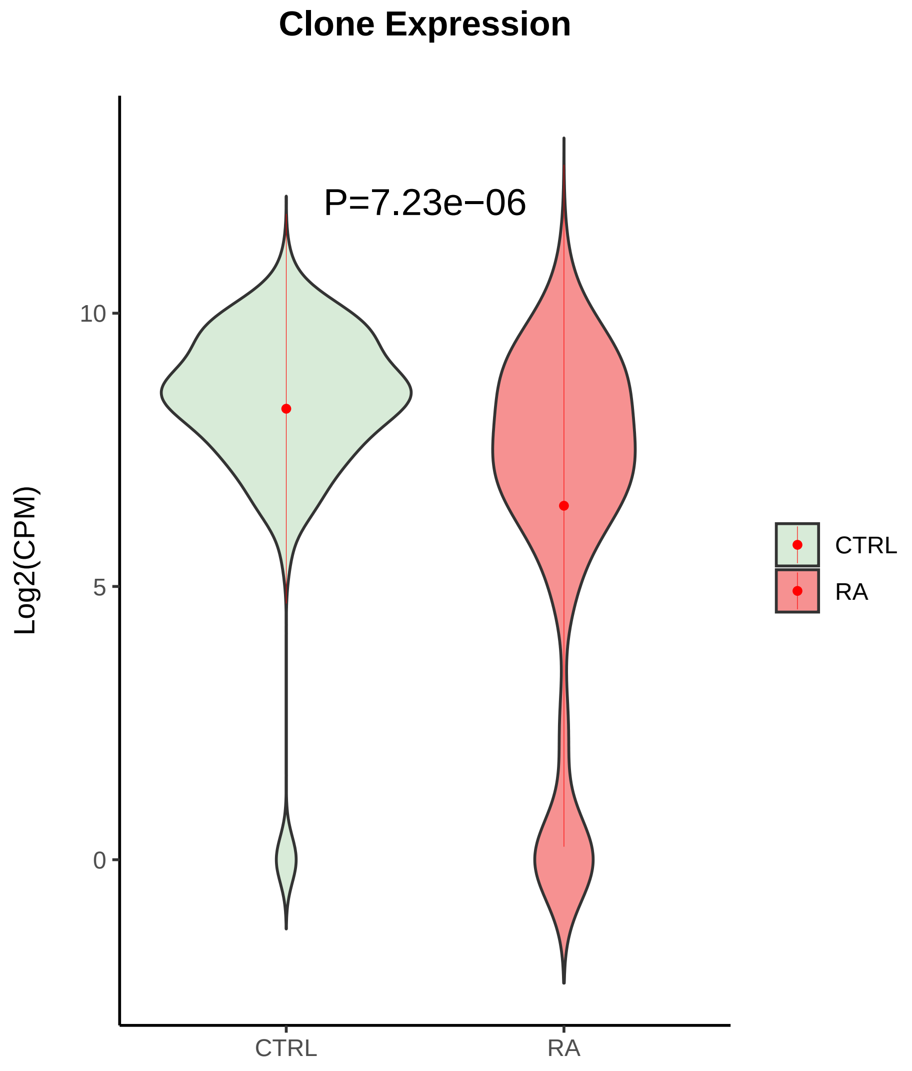

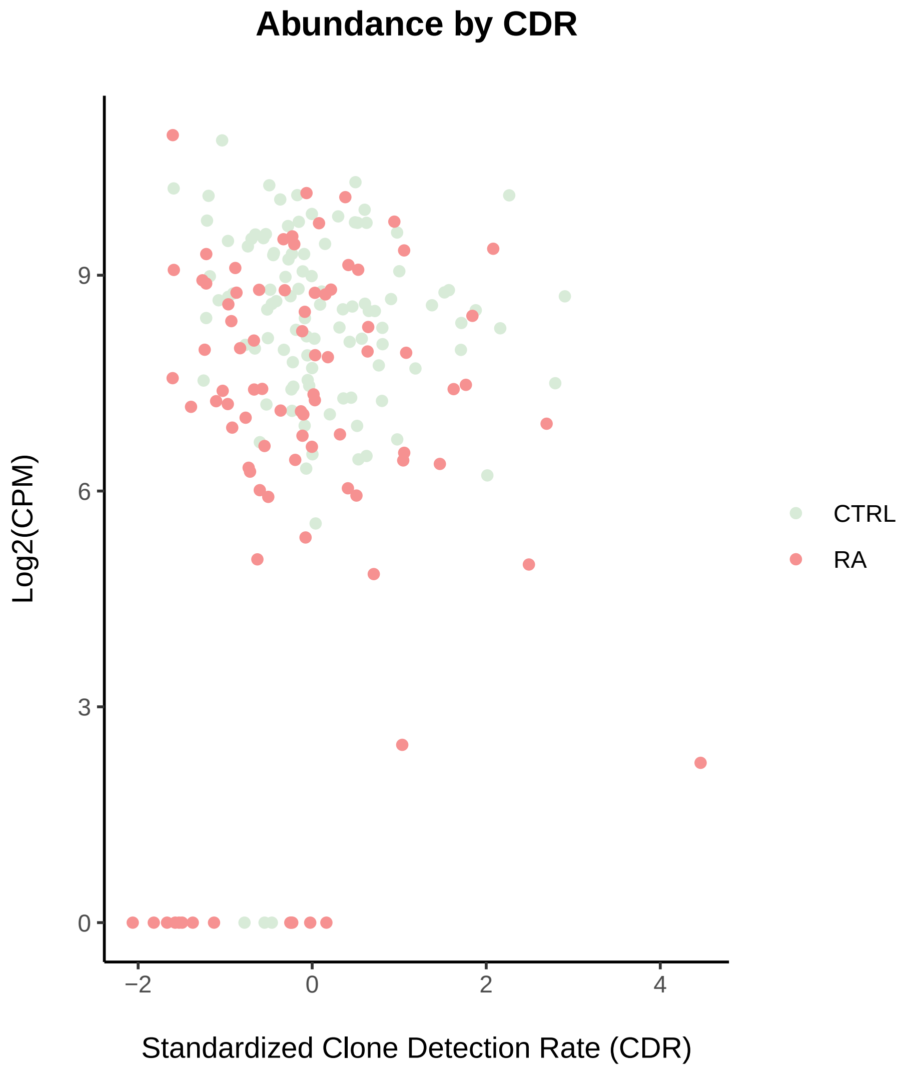

CMQRIEFPYTF from IGK chain significant in Hurdle model

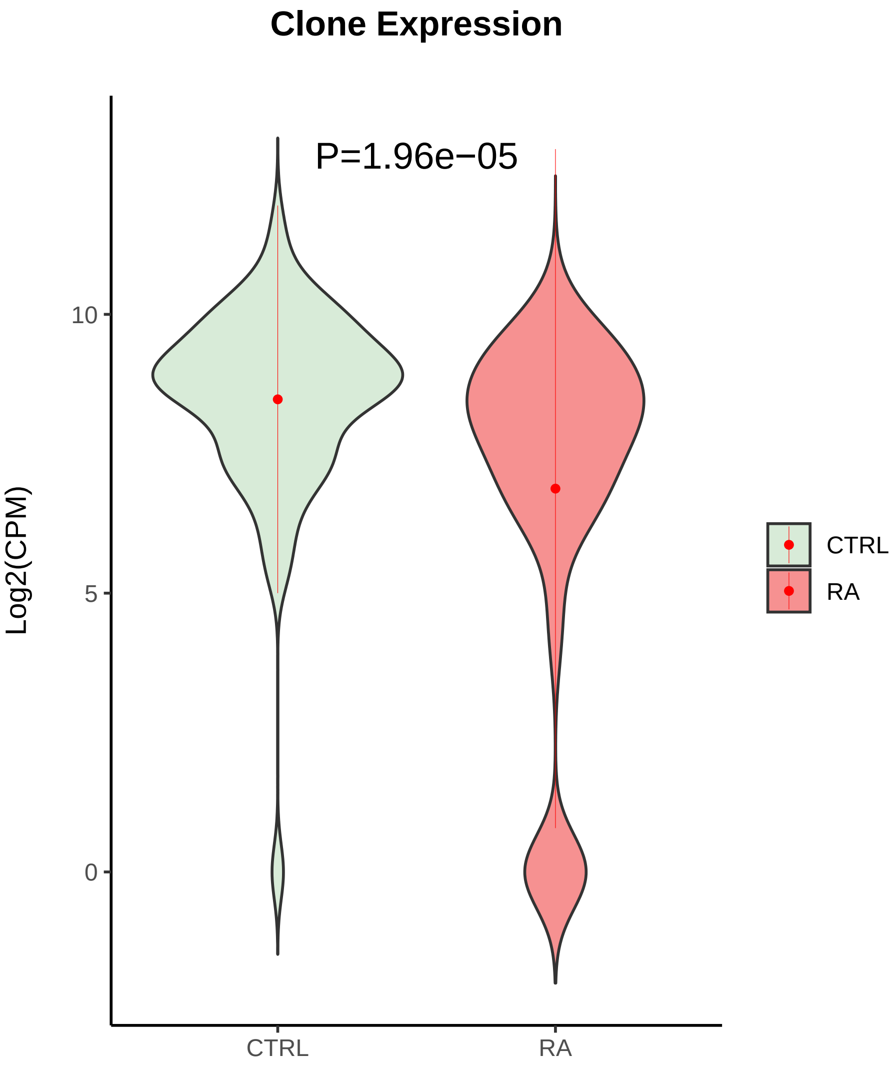

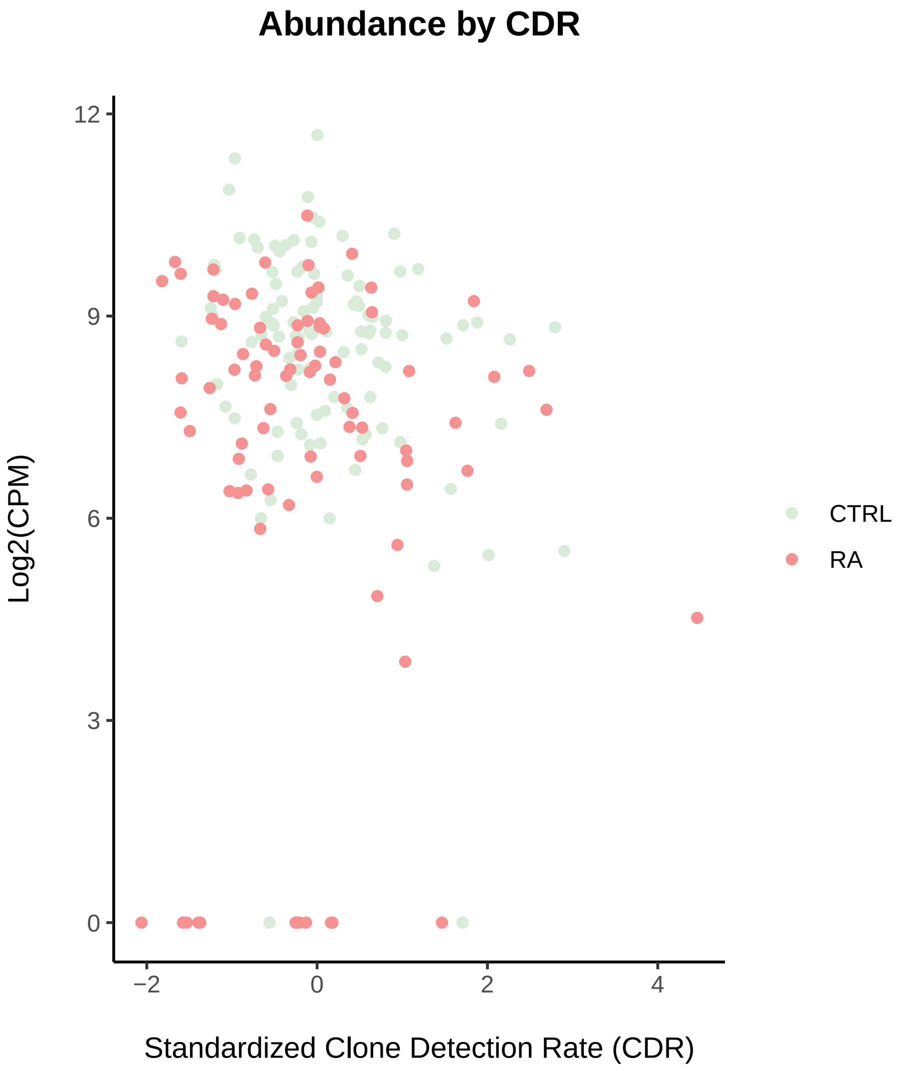

CMQSIQVPWTF from IGK chain significant in Hurdle model

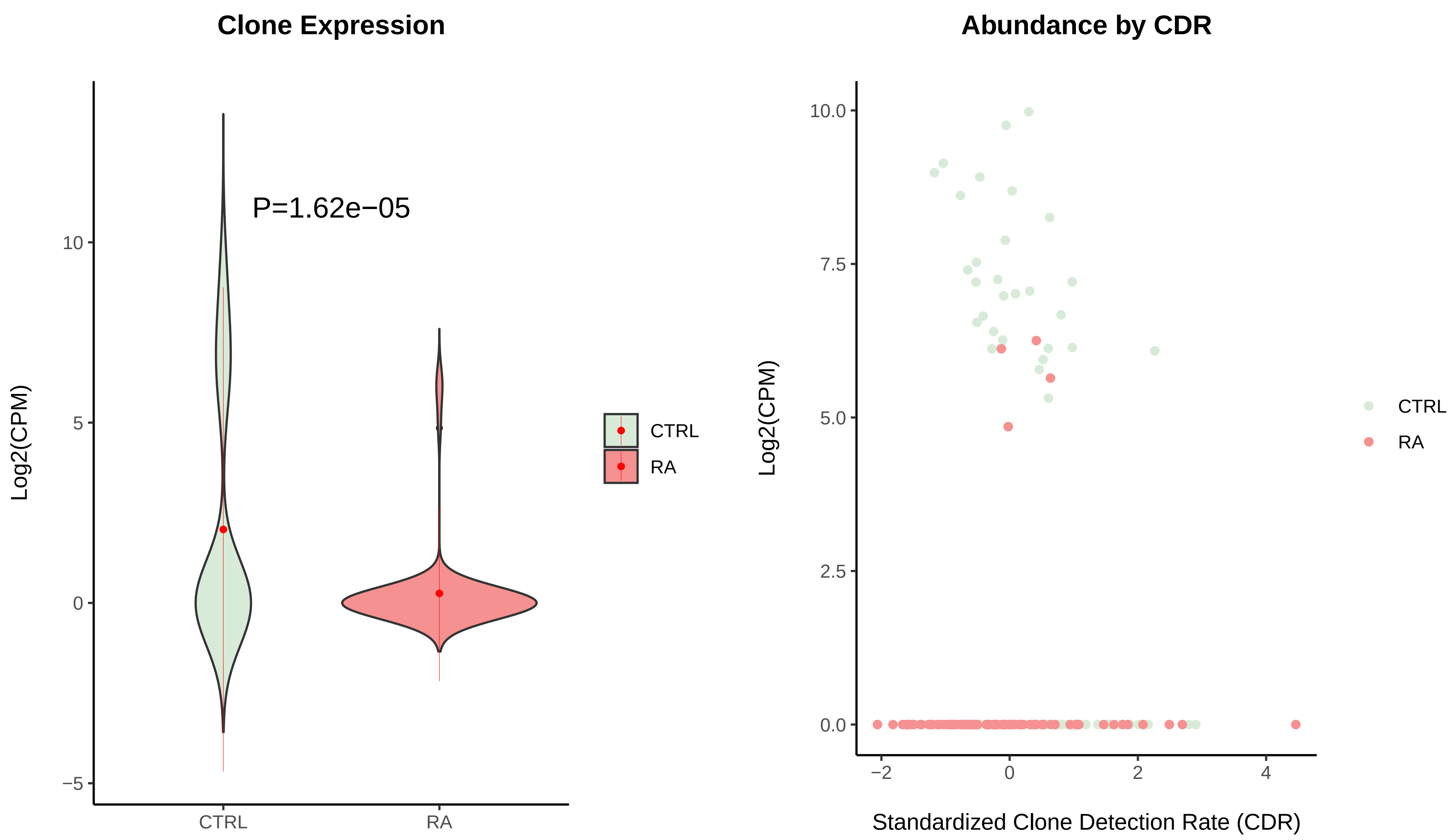

CMQSLQTPPTF from IGK chain significant in Hurdle model

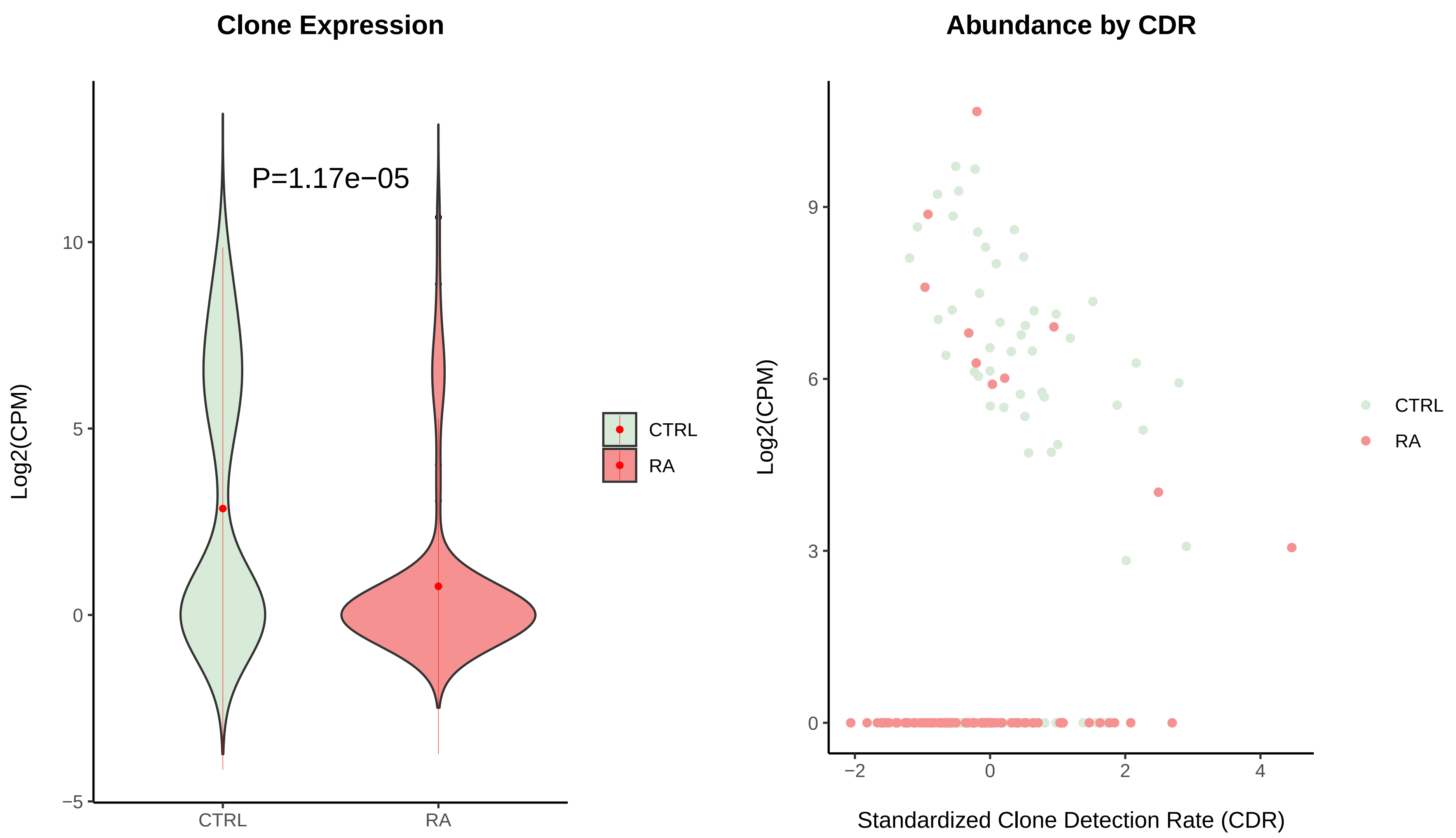

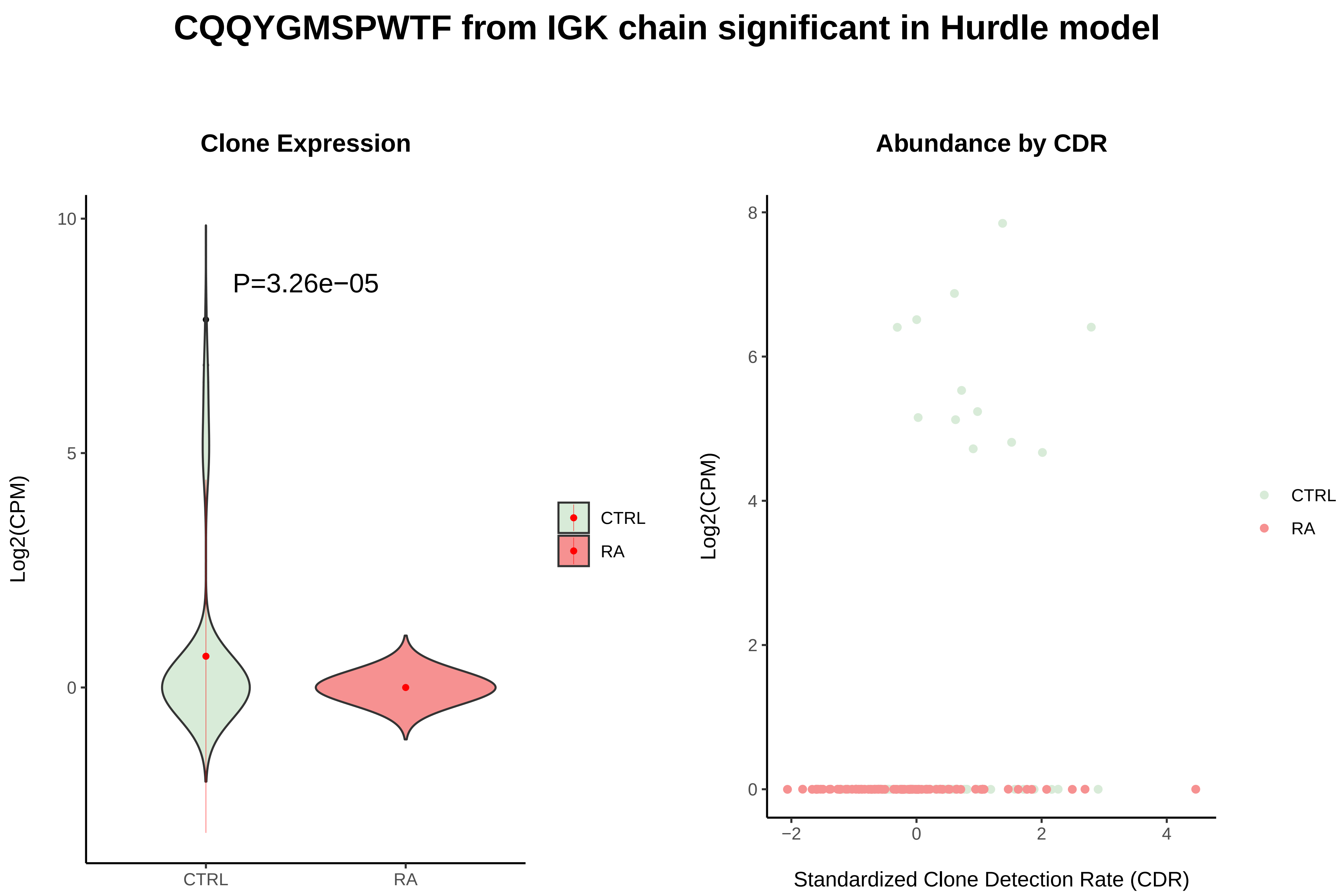

CQQYGMSPWTF from IGK chain significant in Hurdle model
Clone Expression
P=3.26e−05
Log2(CPM)
10
5
0
CTRL
RA
CTRL
RA
Abundance by CDR
Log2(CPM)
8
6
4
2
0
−2
0
2
4
Standardized Clone Detection Rate (CDR)
CTRL
RA

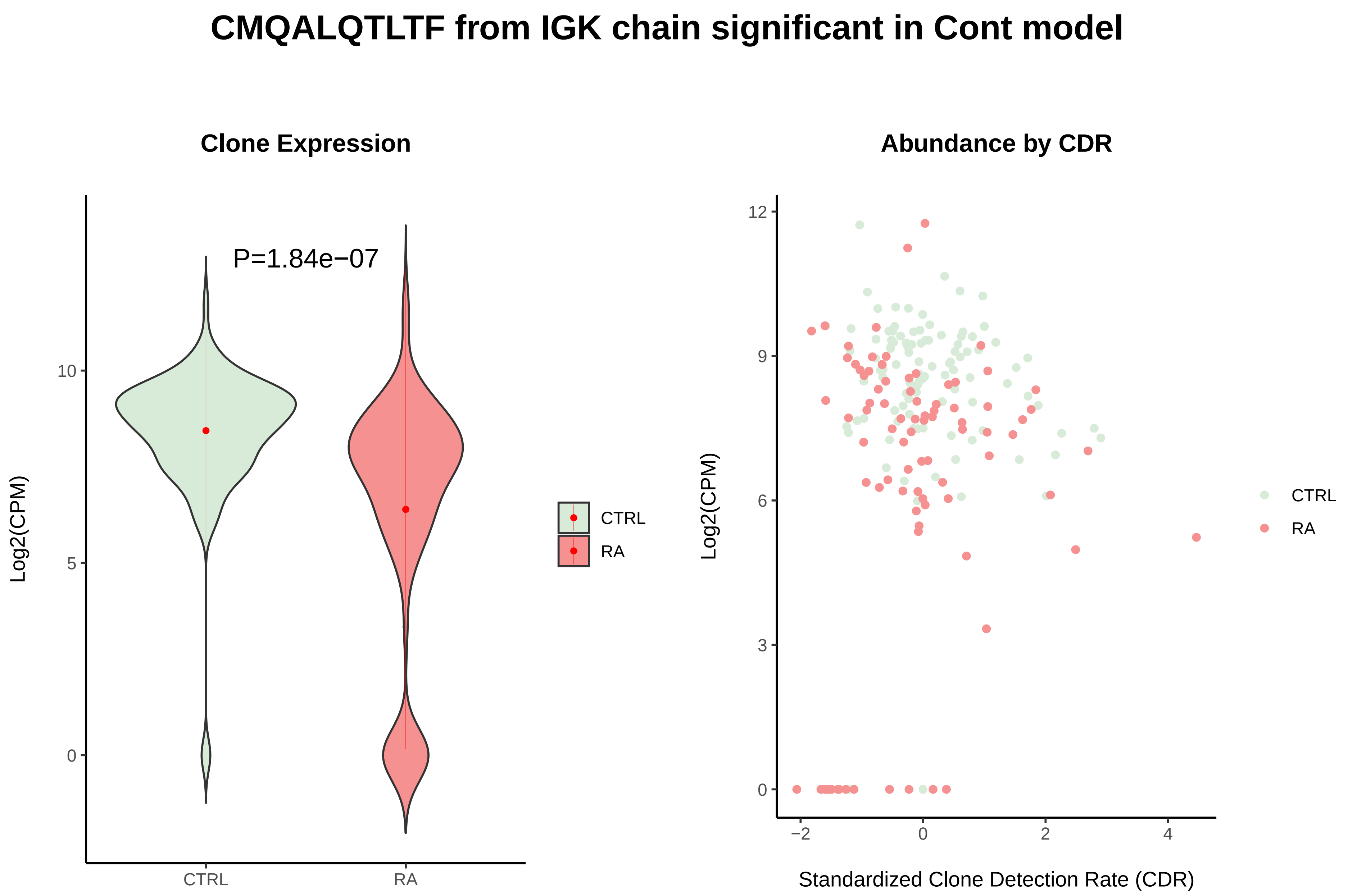
CMQALQTLTF from IGK chain significant in Cont model
Clone Expression
P=1.84e−07
Log2(CPM)
0
5
10
CTRL
RA
CTRL
RA
Abundance by CDR
Log2(CPM)
0
3
6
9
12
−2
0
2
4
Standardized Clone Detection Rate (CDR)
CTRL
RA

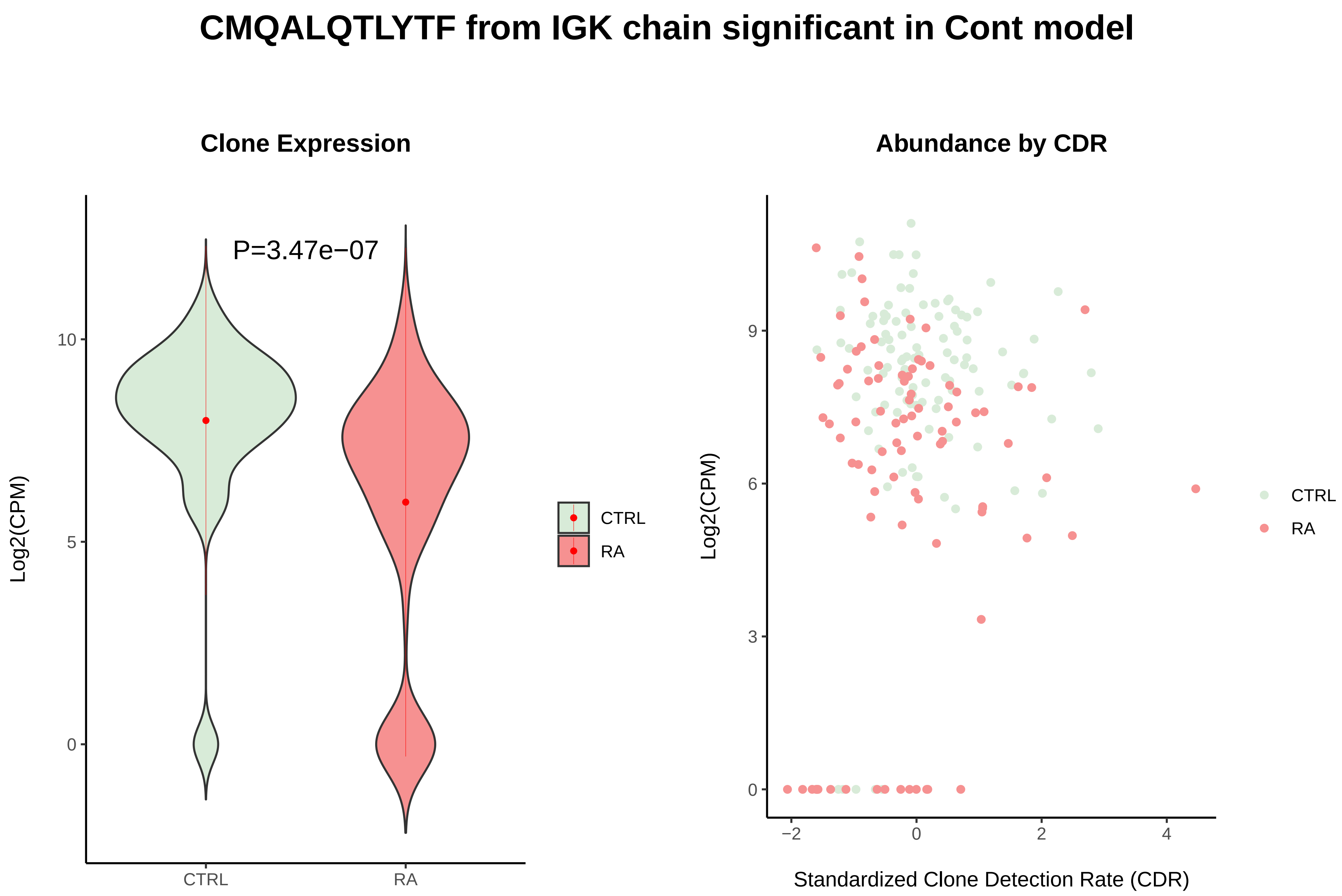

CMQALQTLYTF from IGK chain significant in Cont model
Clone Expression
P=3.47e−07
Log2(CPM)
10
5
0
CTRL
RA
Abundance by CDR
9
6
3
0
−2
0
2
4
Standardized Clone Detection Rate (CDR)

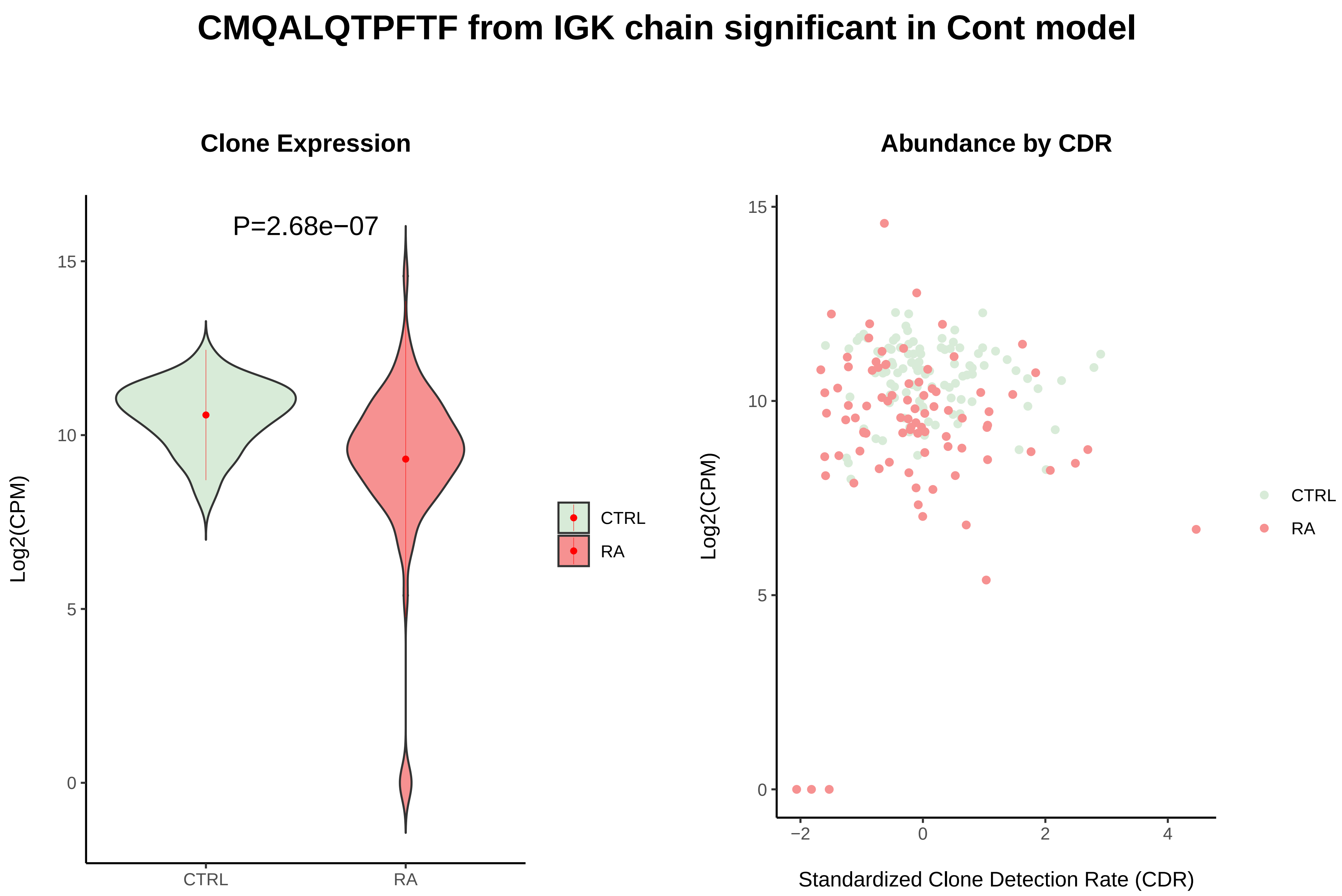
CMQALQTPFTF from IGK chain significant in Cont model
Clone Expression
P=2.68e−07
Log2(CPM)
0
5
10
15
CTRL
RA
CTRL
RA
Abundance by CDR
Log2(CPM)
0
5
10
15
−2
0
2
4
Standardized Clone Detection Rate (CDR)
CTRL
RA

CMQALQTPGTF from IGK chain significant in Cont model

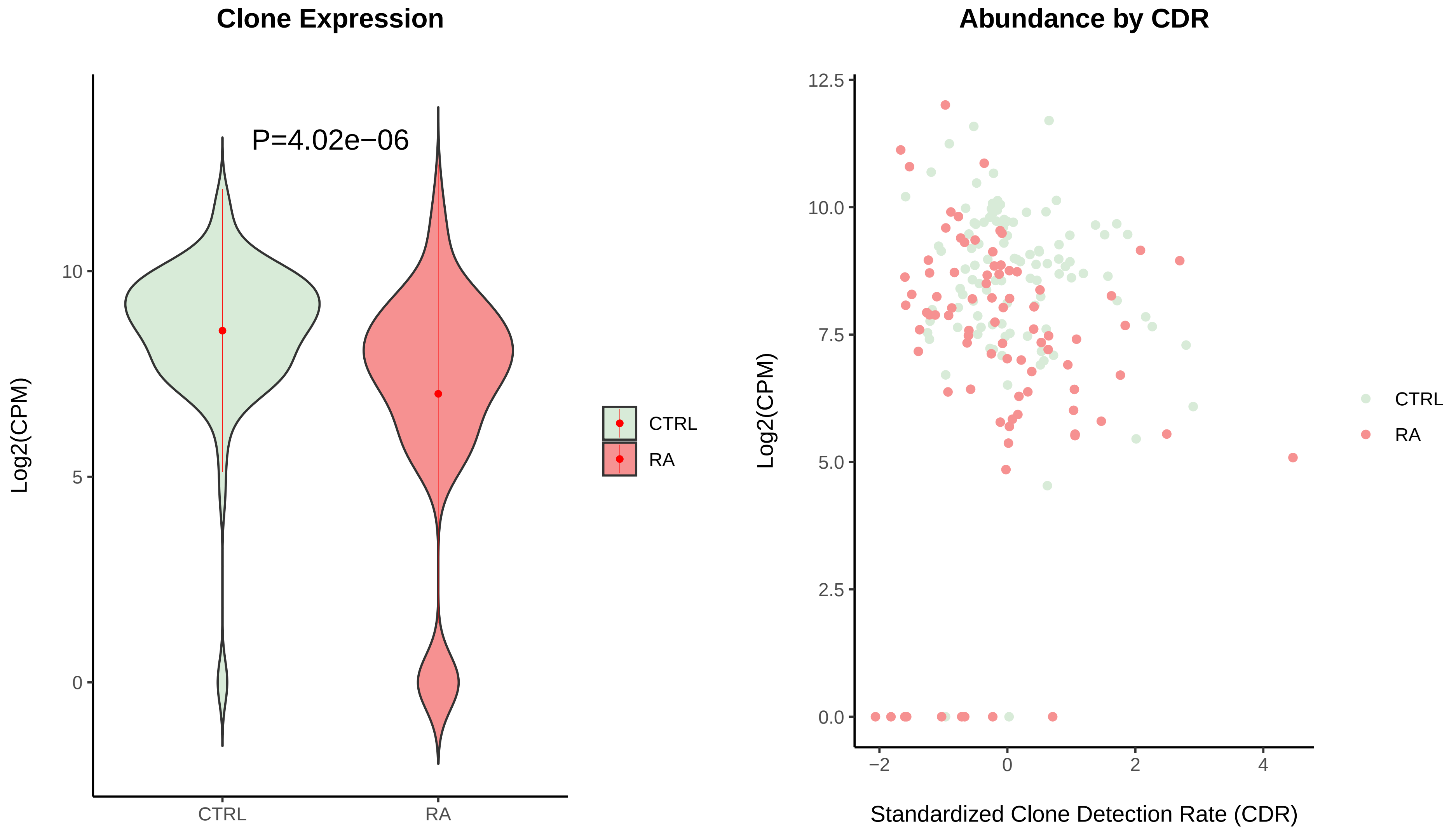

CMQALQTPHTF from IGK chain significant in Cont model

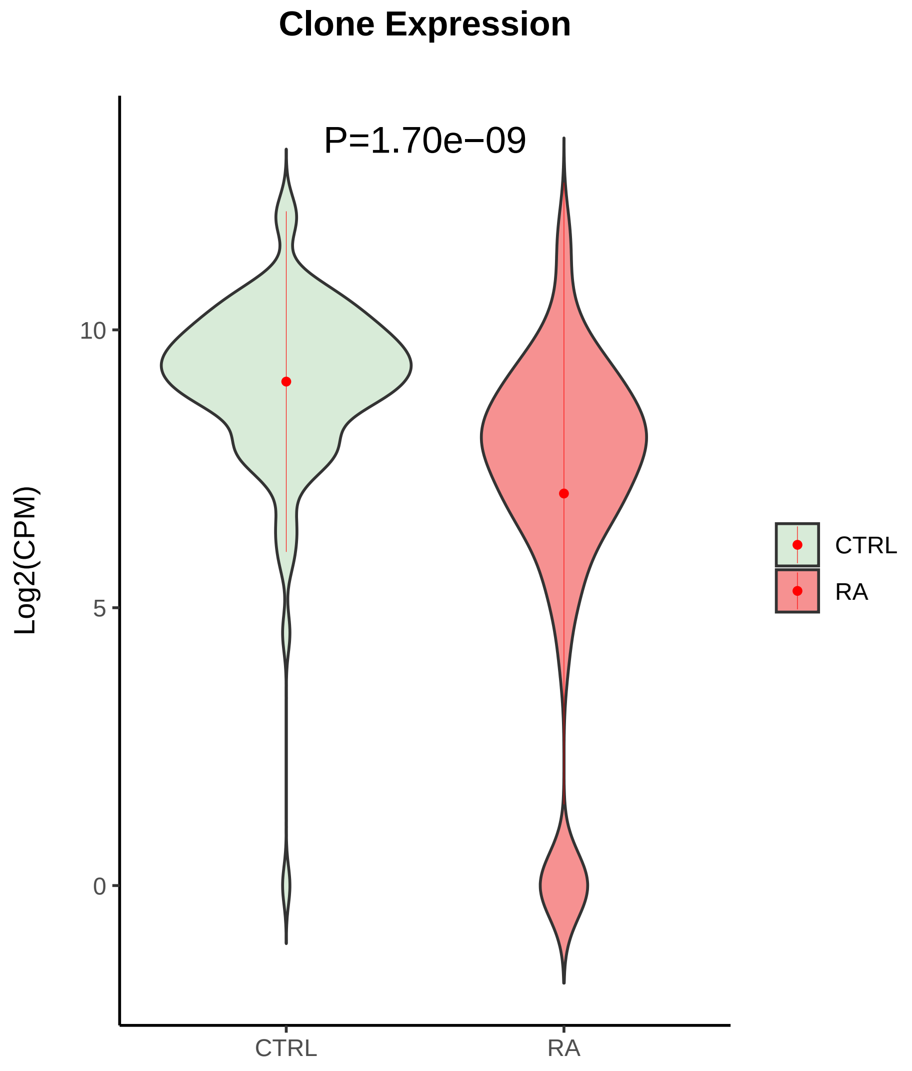

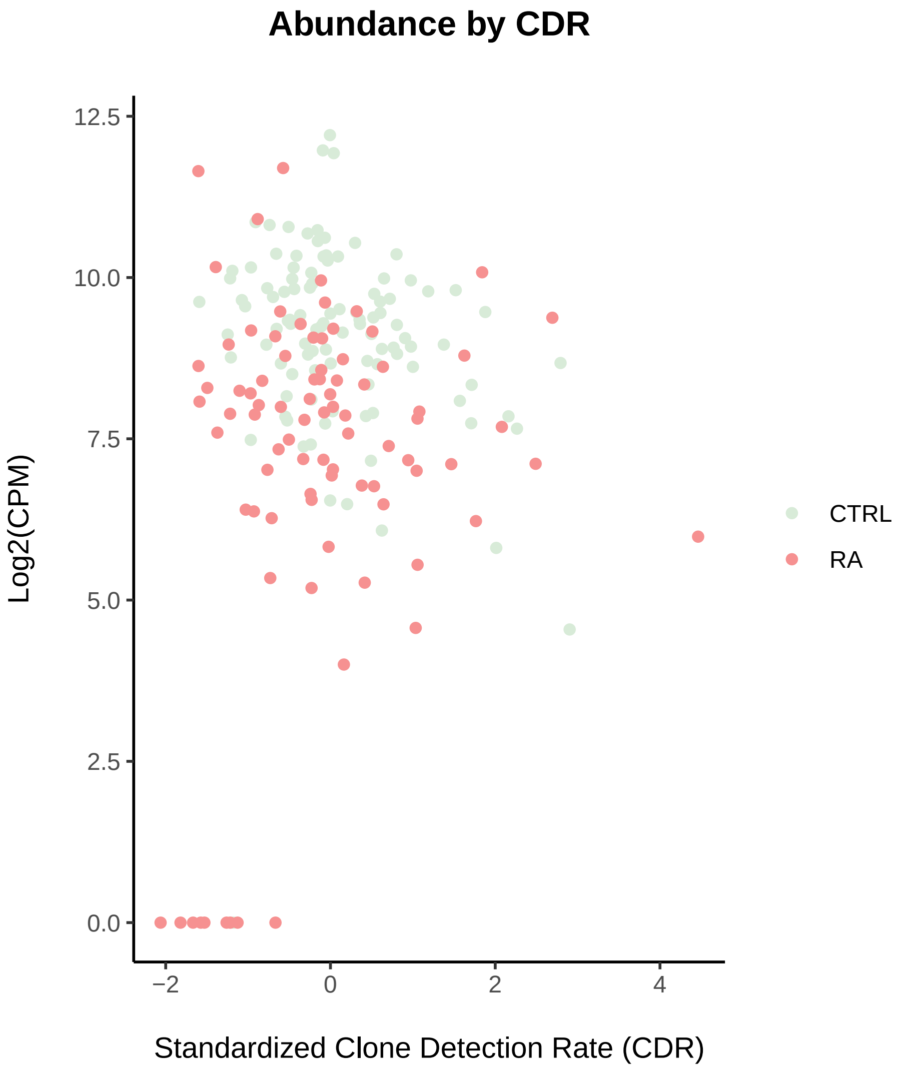

CMQALQTPITF from IGK chain significant in Cont model

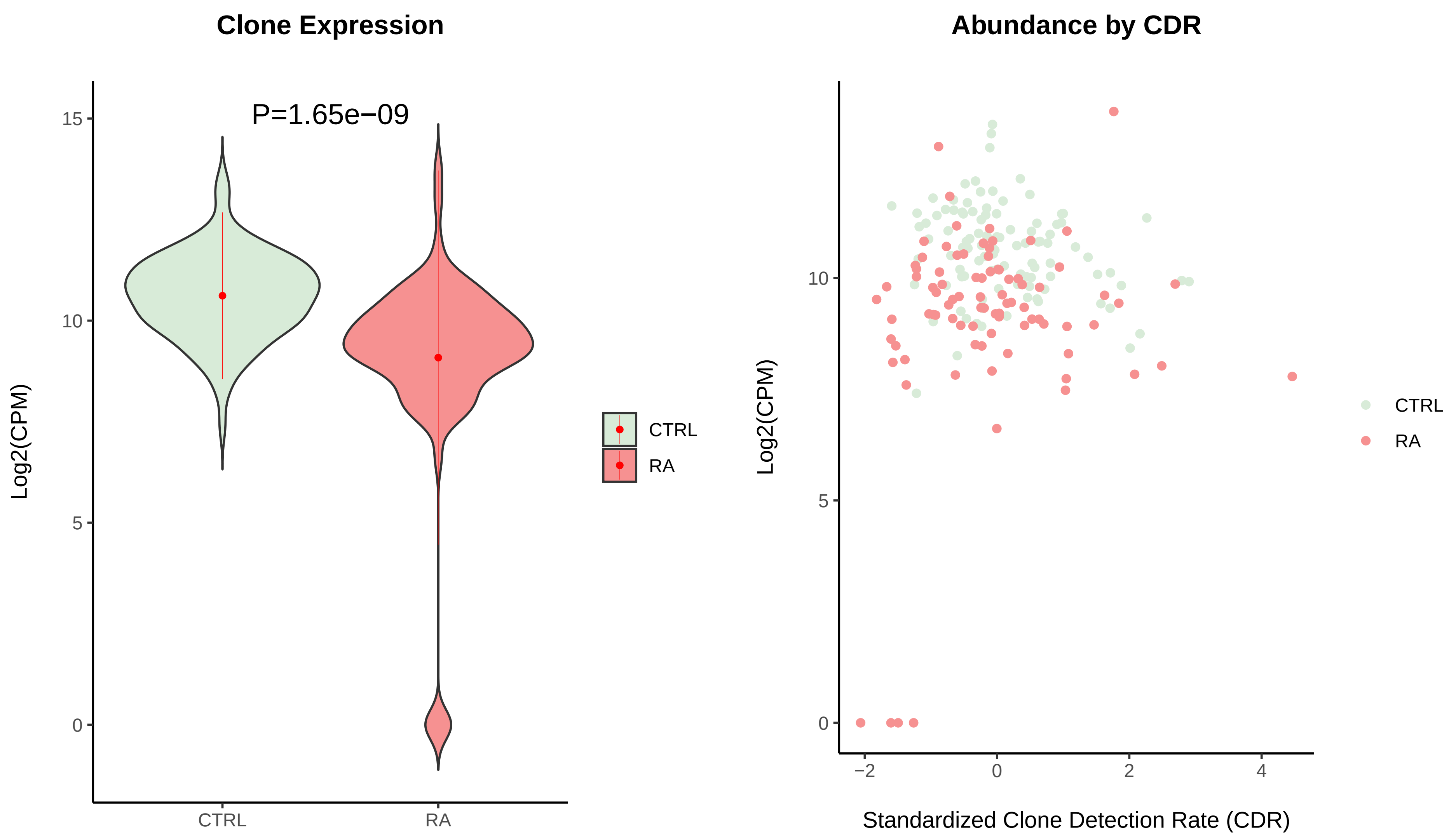

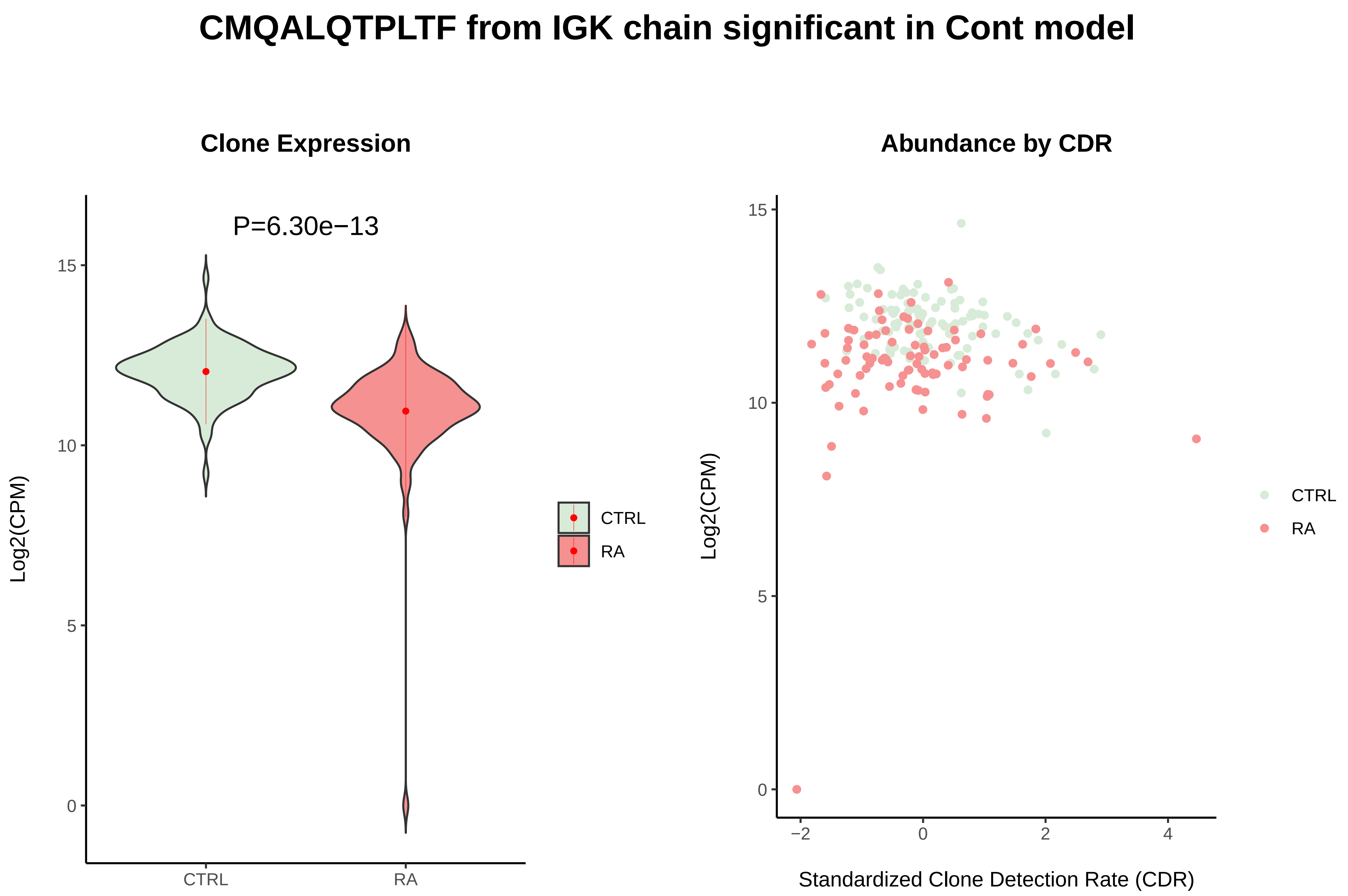
CMQALQTPLTF from IGK chain significant in Cont model
Clone Expression
P=6.30e−13
Log2(CPM)
0
5
10
15
CTRL
RA
CTRL
RA
Abundance by CDR
Log2(CPM)
0
5
10
15
−2
0
2
4
Standardized Clone Detection Rate (CDR)
CTRL
RA

CMQALQTPPLTF from IGK chain significant in Cont model

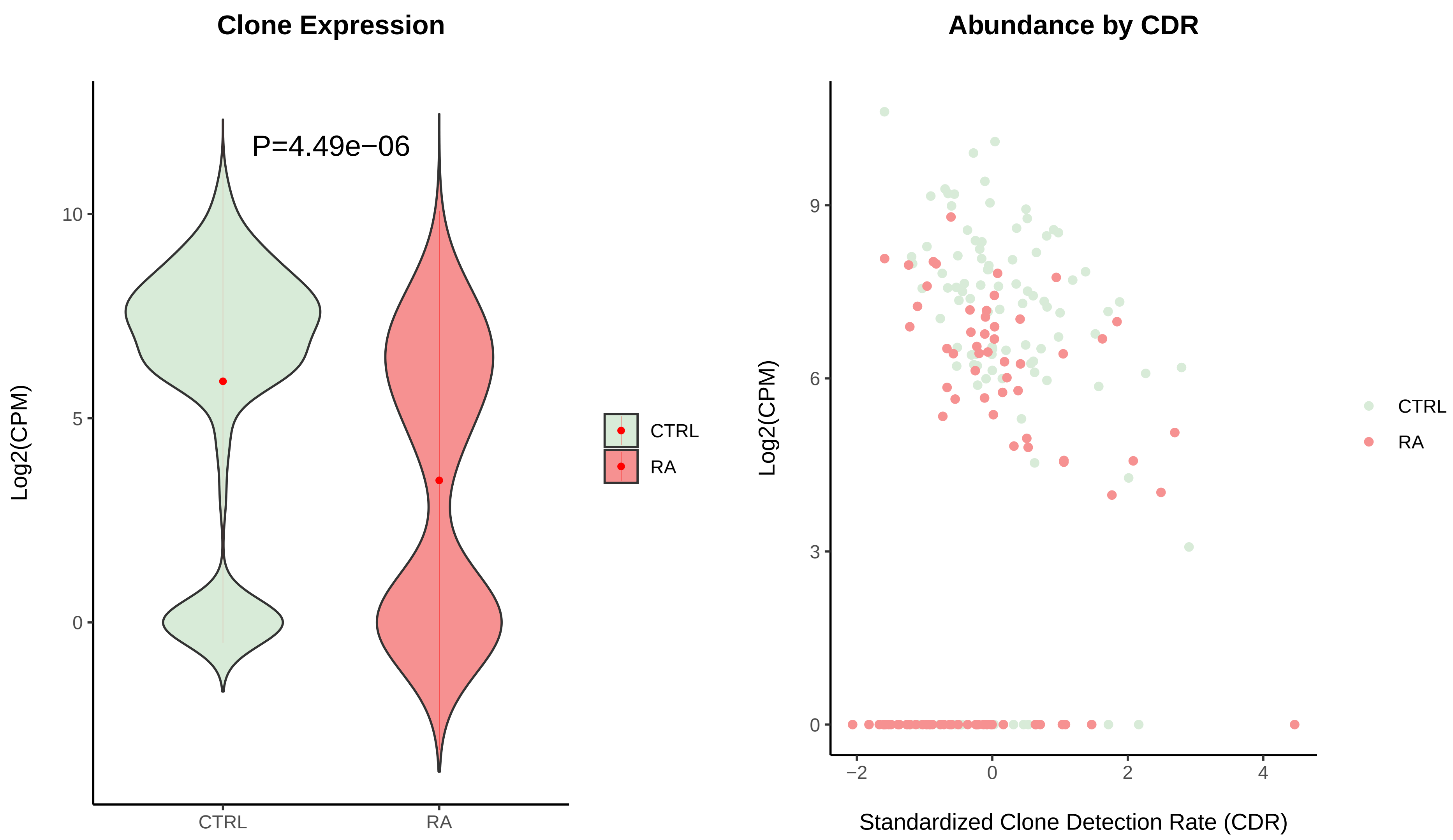

CMQALQTPPTF from IGK chain significant in Cont model

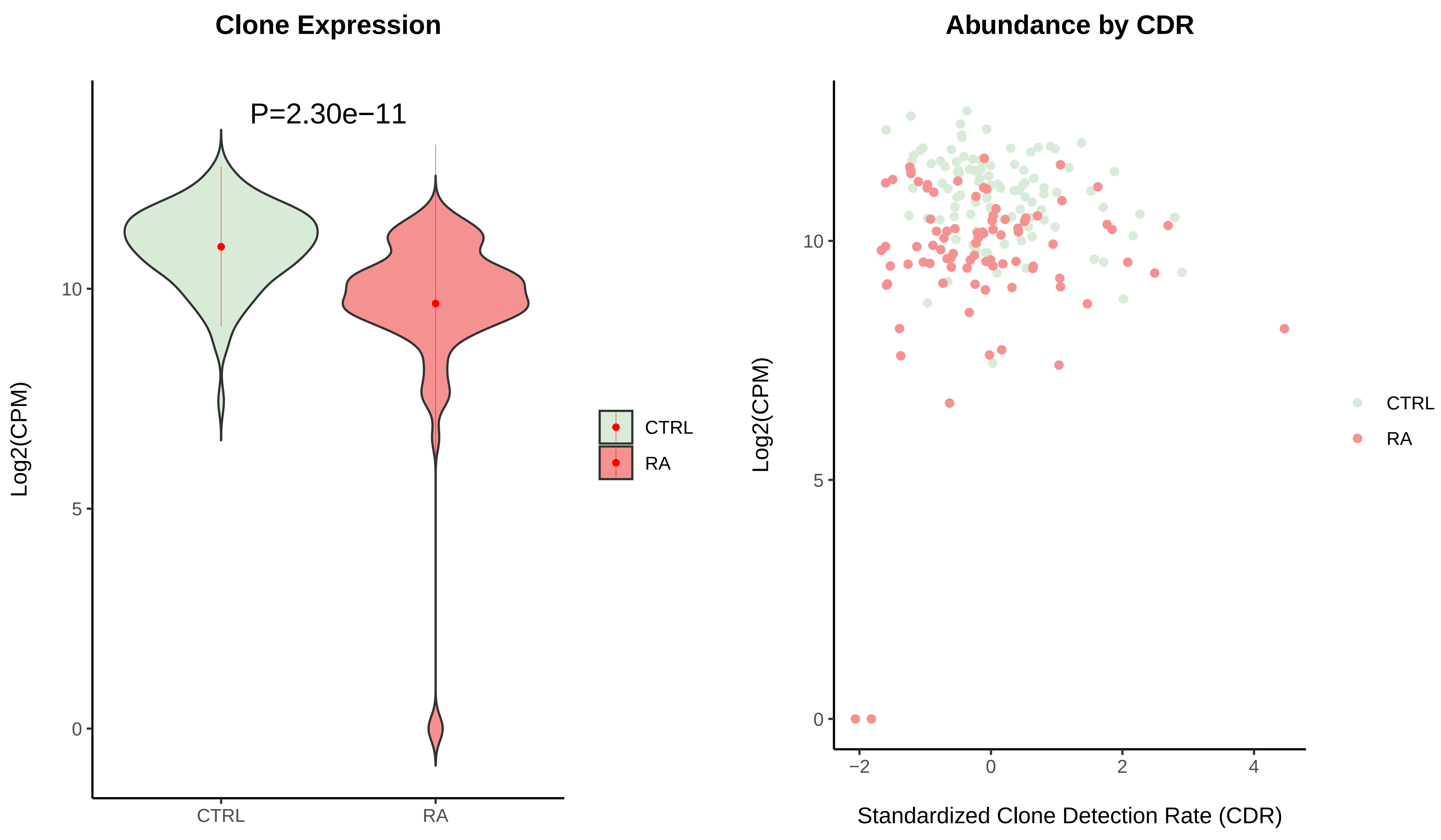

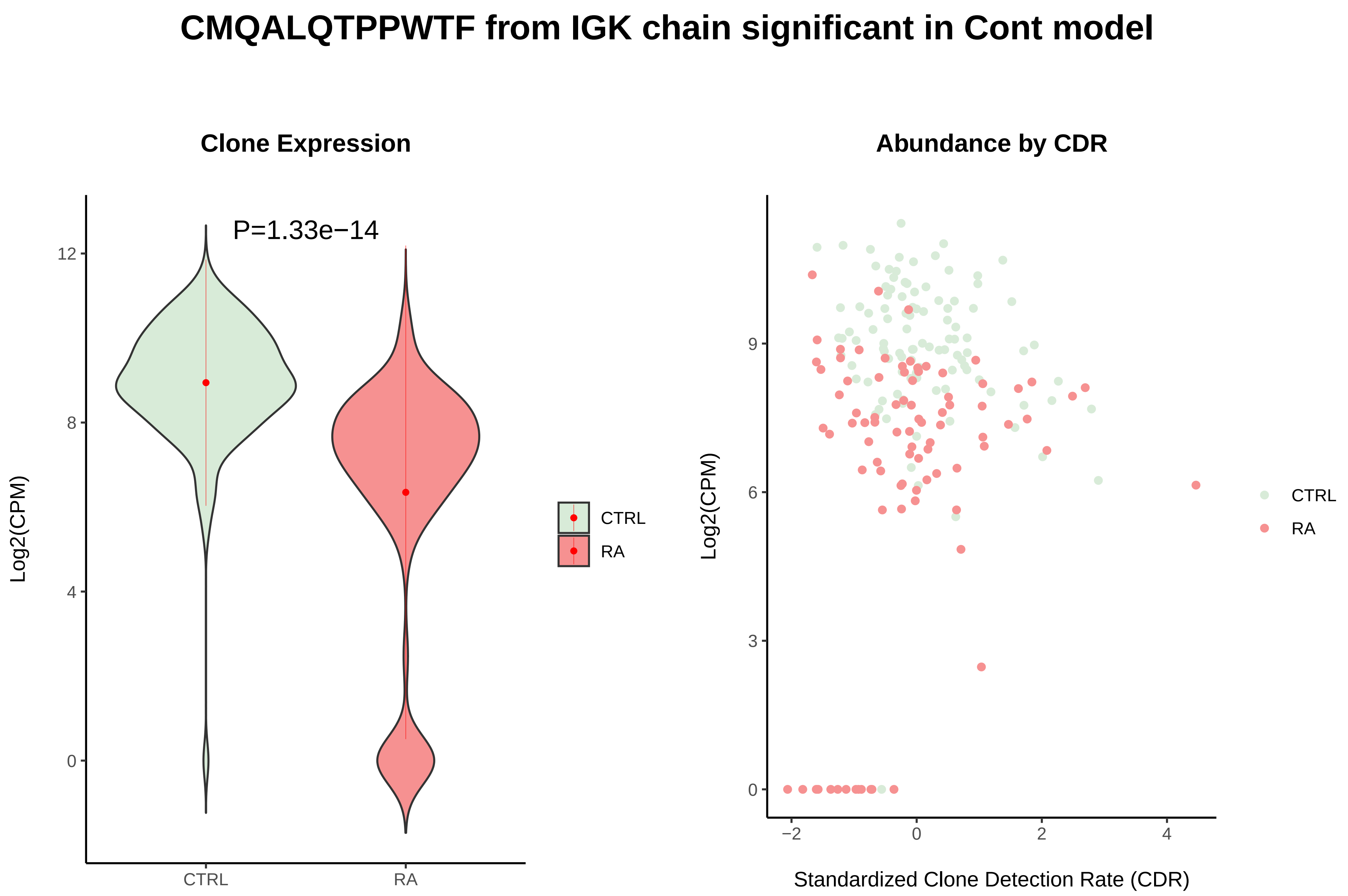
CMQALQTPPWTF from IGK chain significant in Cont model
Clone Expression
P=1.33e−14
Log2(CPM)
0
4
8
12
CTRL
RA
CTRL
RA
Abundance by CDR
Log2(CPM)
0
3
6
9
−2
0
2
4
Standardized Clone Detection Rate (CDR)
CTRL
RA

CMQALQTPPYTF from IGK chain significant in Cont model

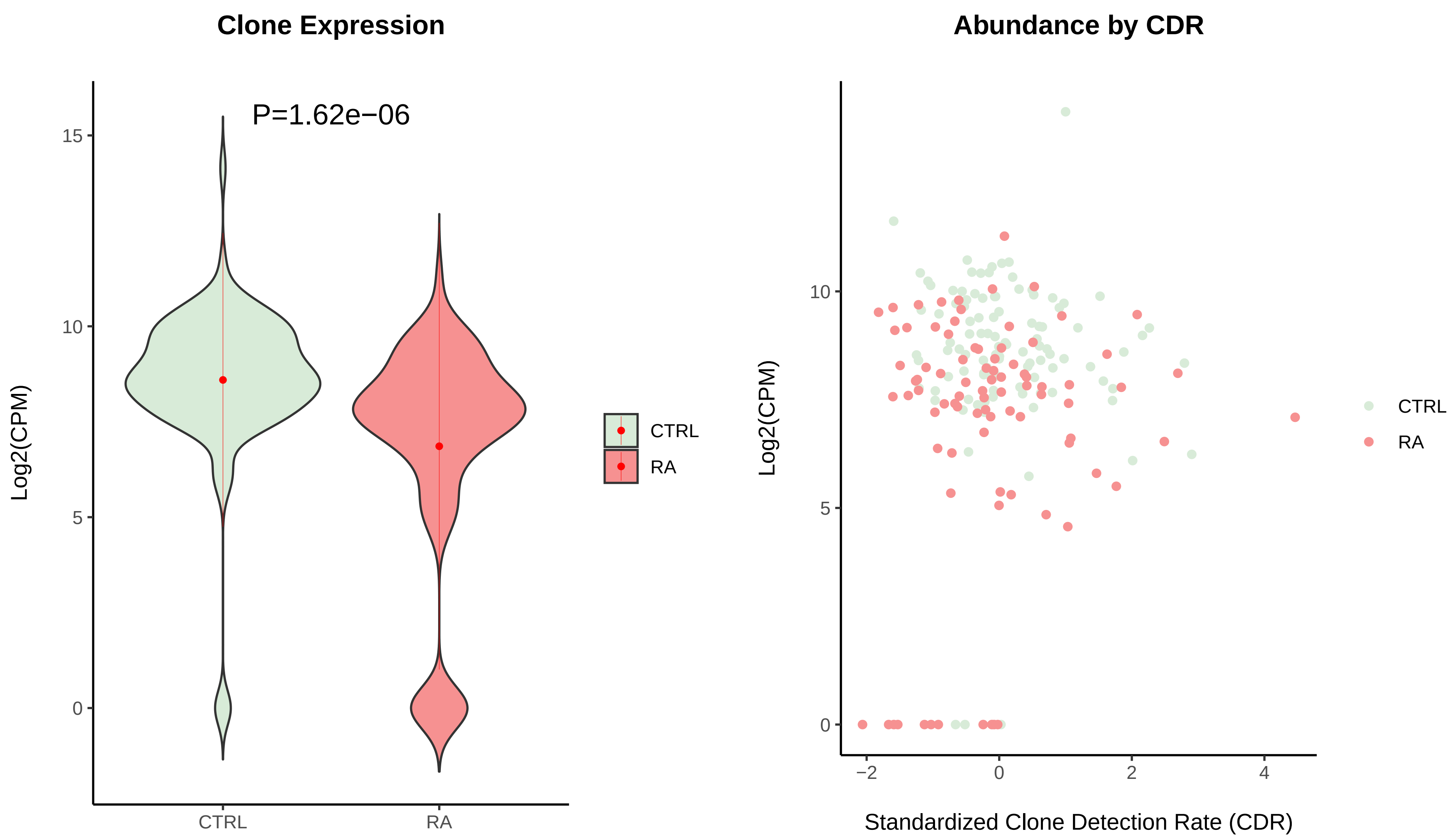

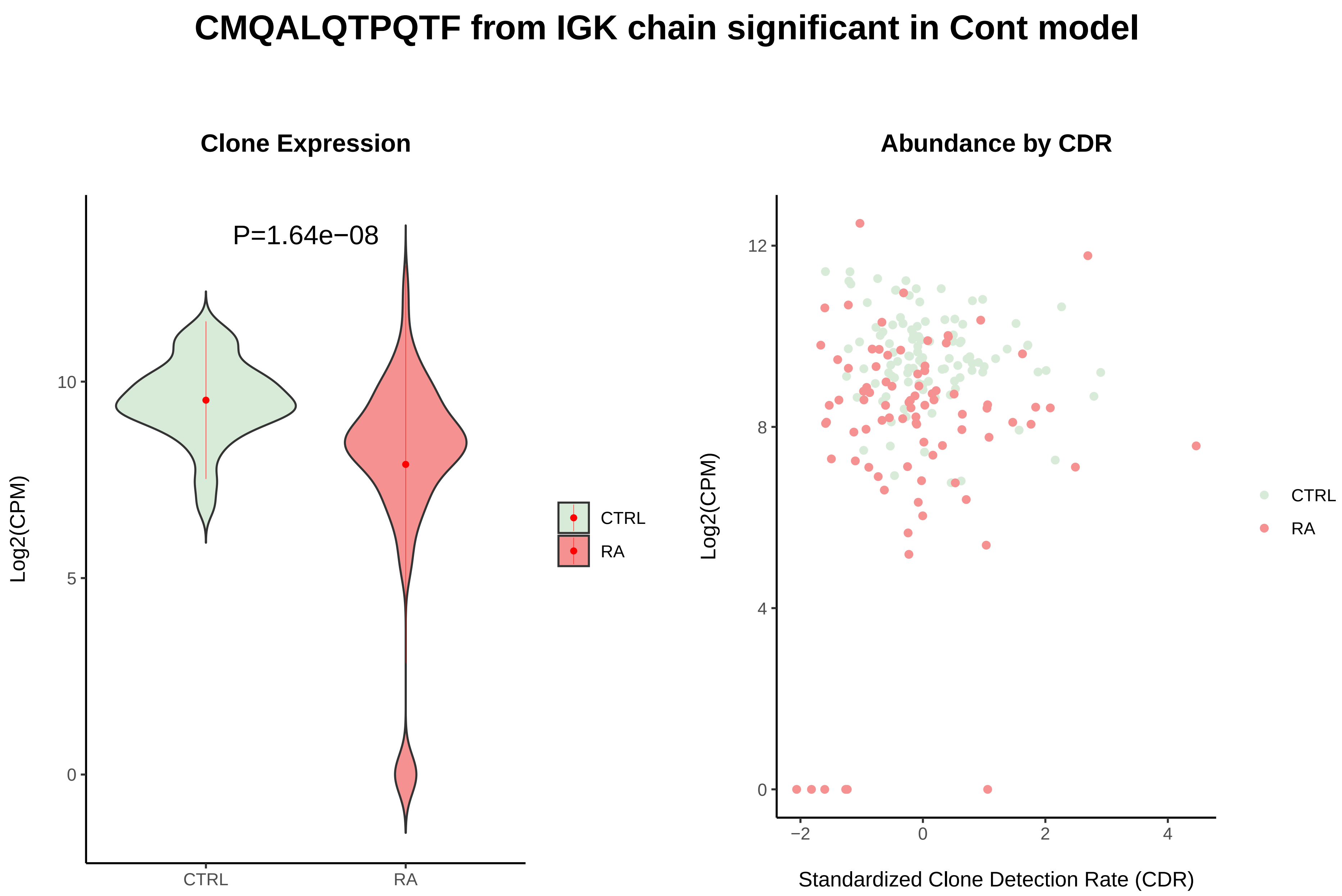
CMQALQTPQTF from IGK chain significant in Cont model
Clone Expression
P=1.64e−08
Log2(CPM)
10
5
0
CTRL
RA
CTRL
RA
Abundance by CDR
Log2(CPM)
12
8
4
0
−2
0
2
4
Standardized Clone Detection Rate (CDR)
CTRL
RA

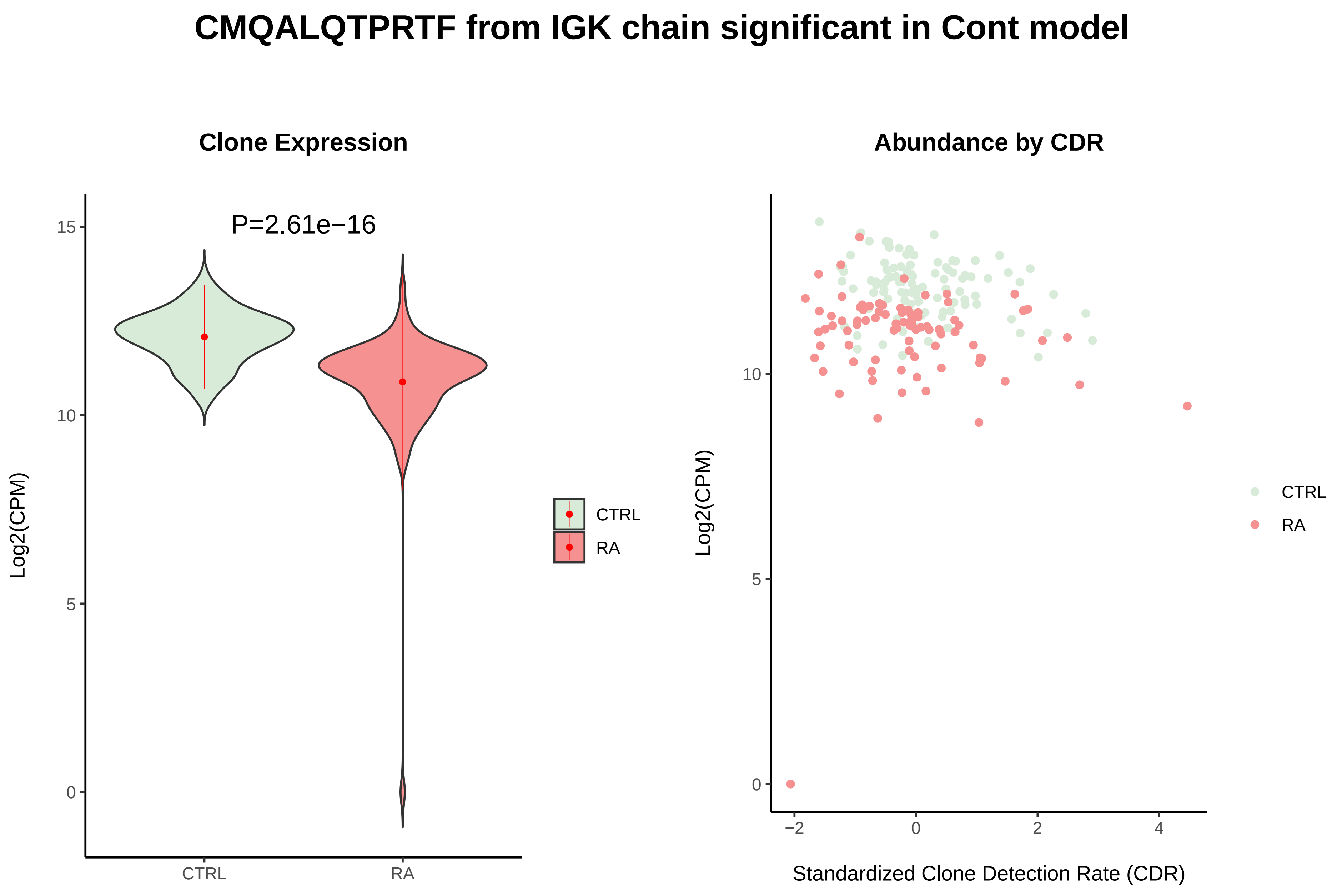

CMQALQTPRTF from IGK chain significant in Cont model
Clone Expression
P=2.61e−16
Log2(CPM)
15
10
5
0
CTRL
RA
CTRL
RA
Abundance by CDR
Log2(CPM)
10
5
0
−2
0
2
4
Standardized Clone Detection Rate (CDR)
CTRL
RA

CMQALQTPTF from IGK chain significant in Cont model

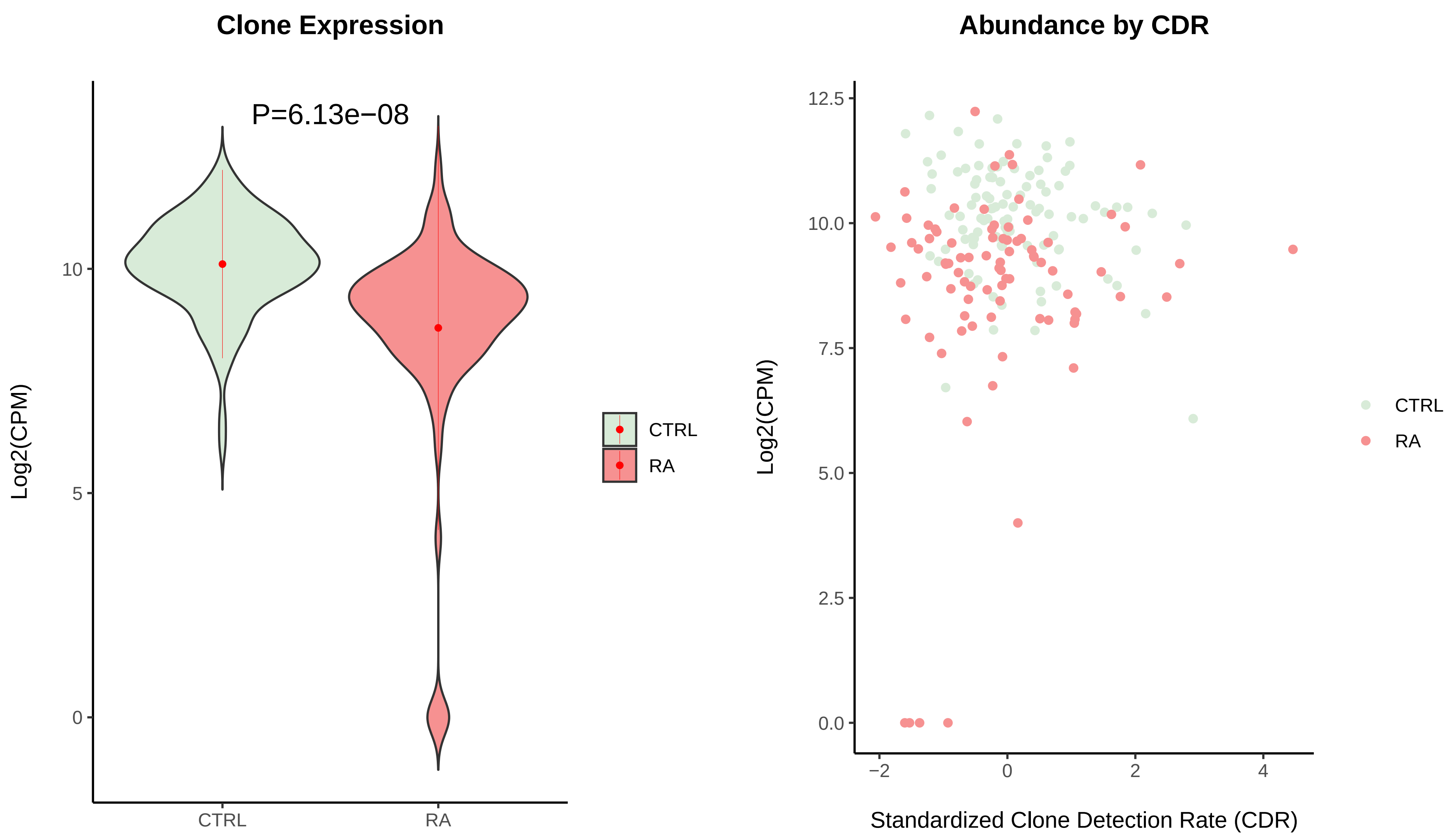

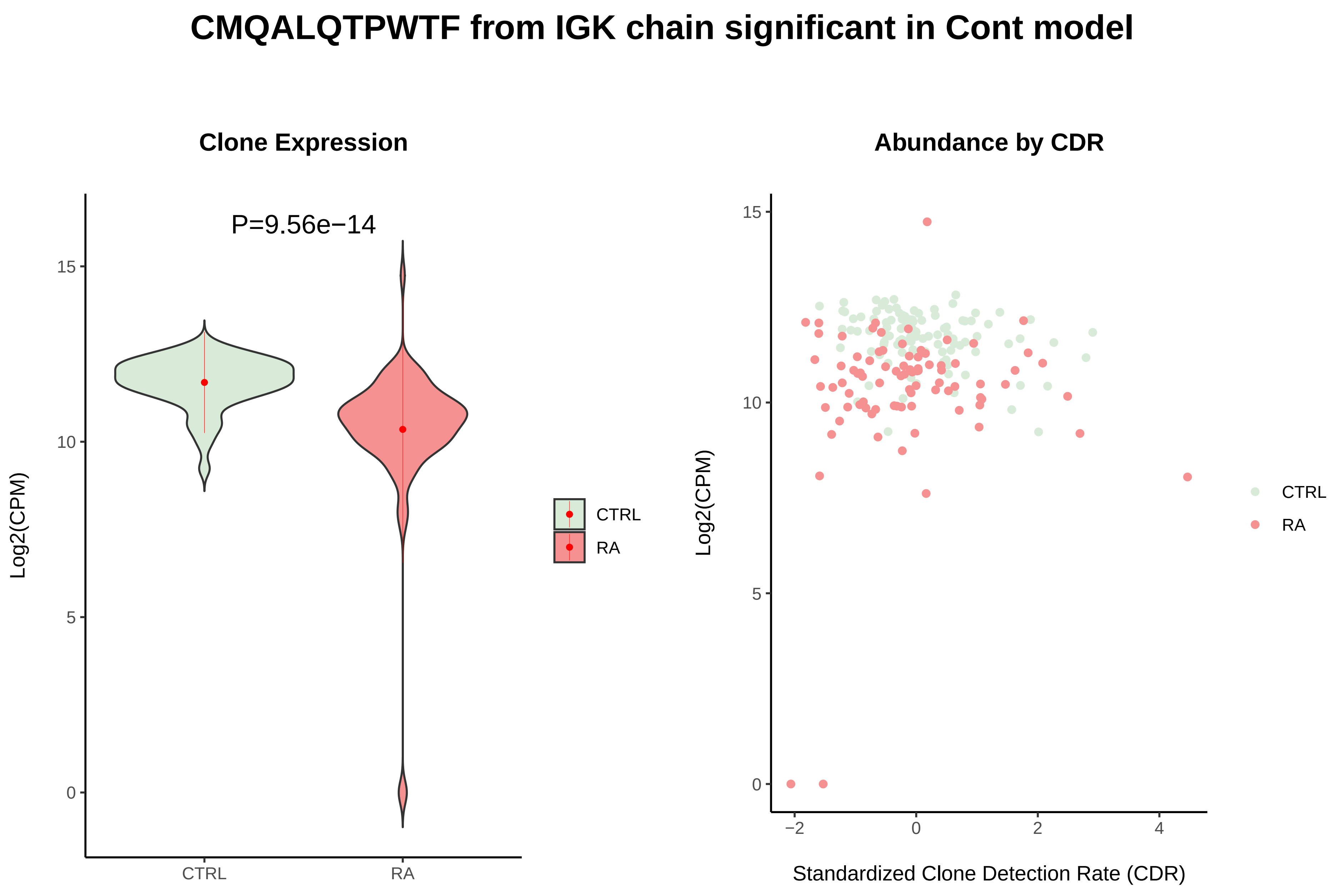
CMQALQTPWTF from IGK chain significant in Cont model
Clone Expression
P=9.56e−14
Log2(CPM)
15
10
5
0
CTRL
RA
CTRL
RA
Abundance by CDR
Log2(CPM)
15
10
5
0
−2
0
2
4
Standardized Clone Detection Rate (CDR)
CTRL
RA

CMQALQTPYTF from IGK chain significant in Cont model

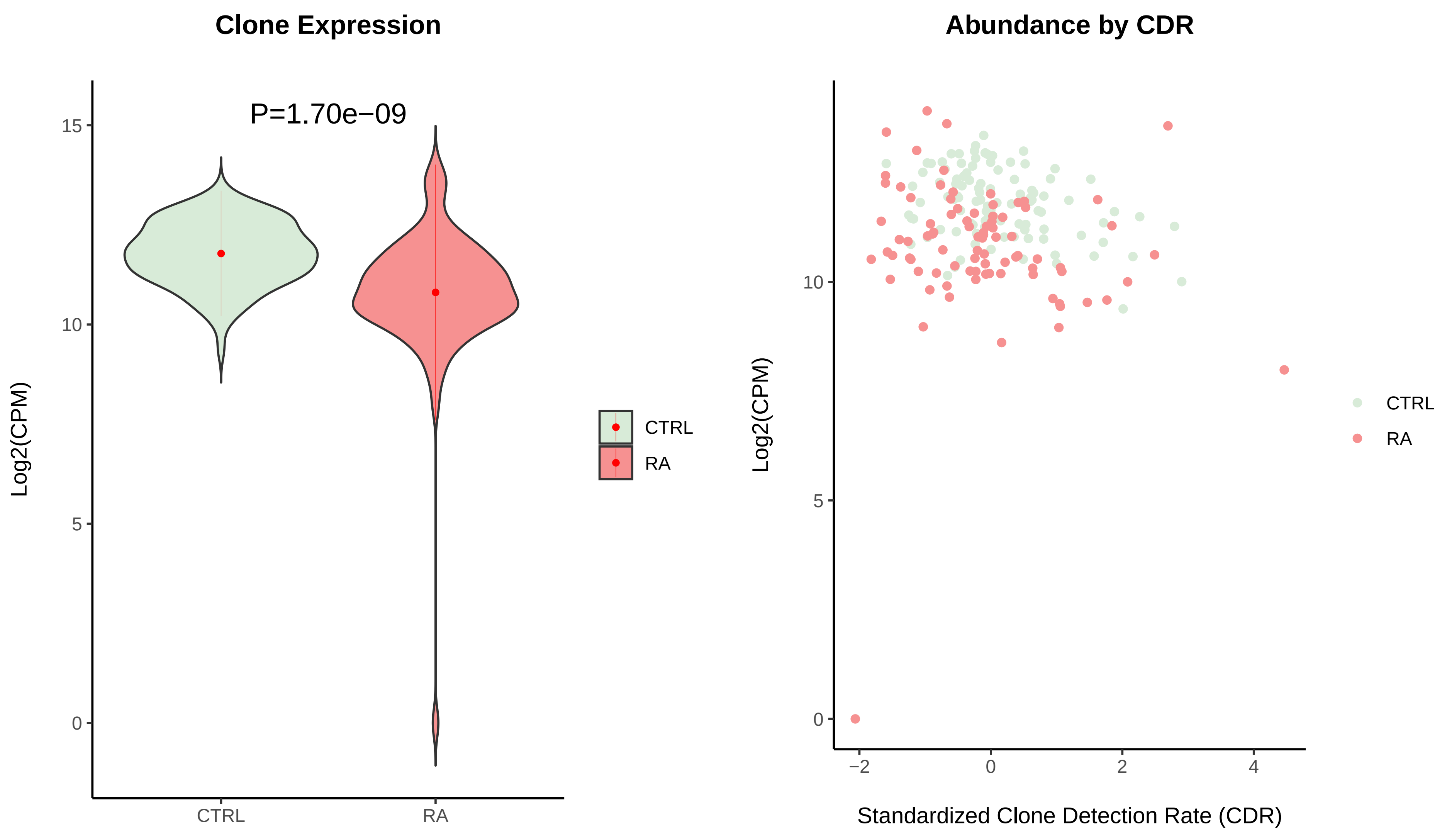

CMQALQTRTF from IGK chain significant in Cont model

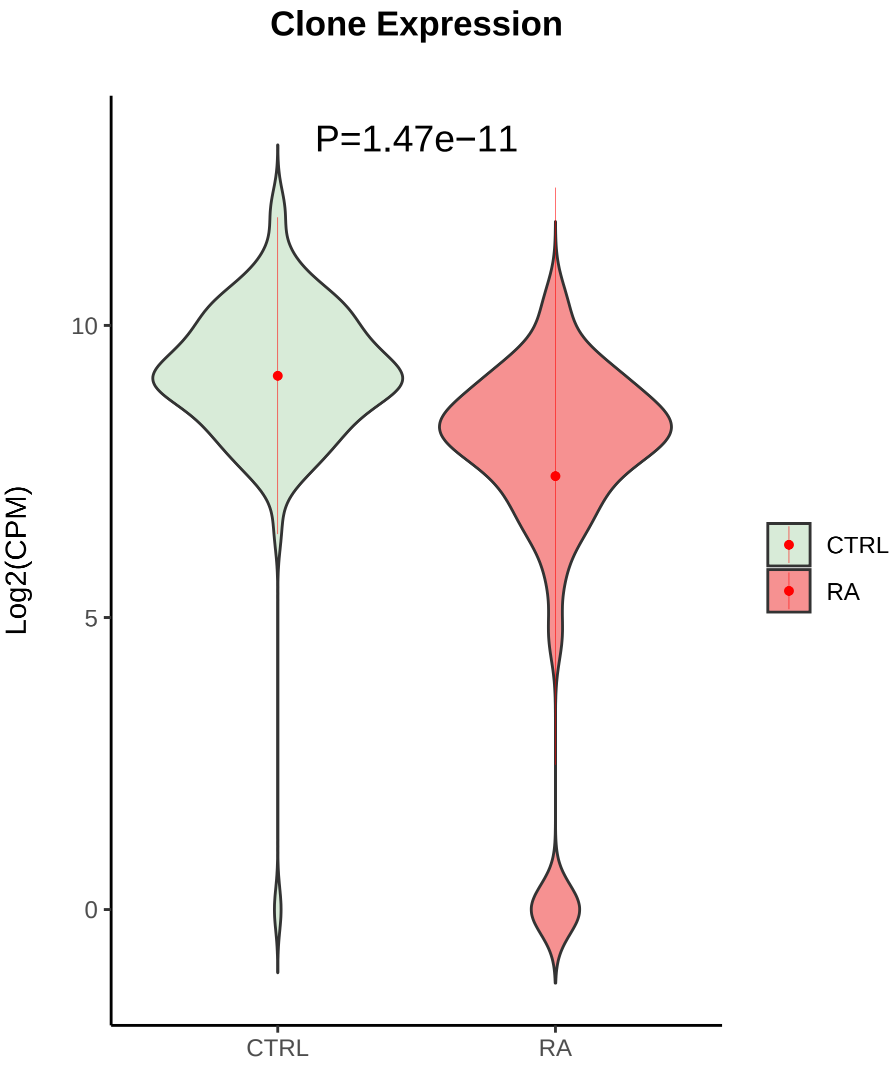

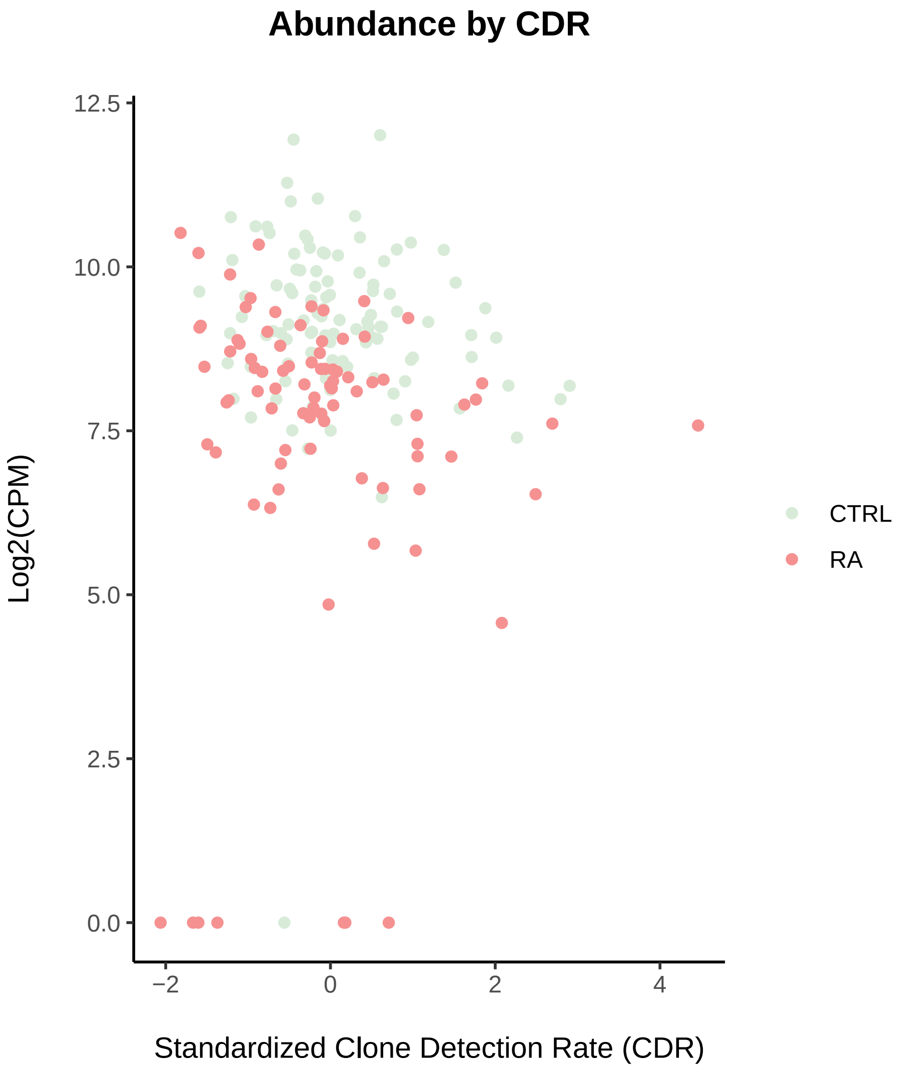

CMQALQTWTF from IGK chain significant in Cont model

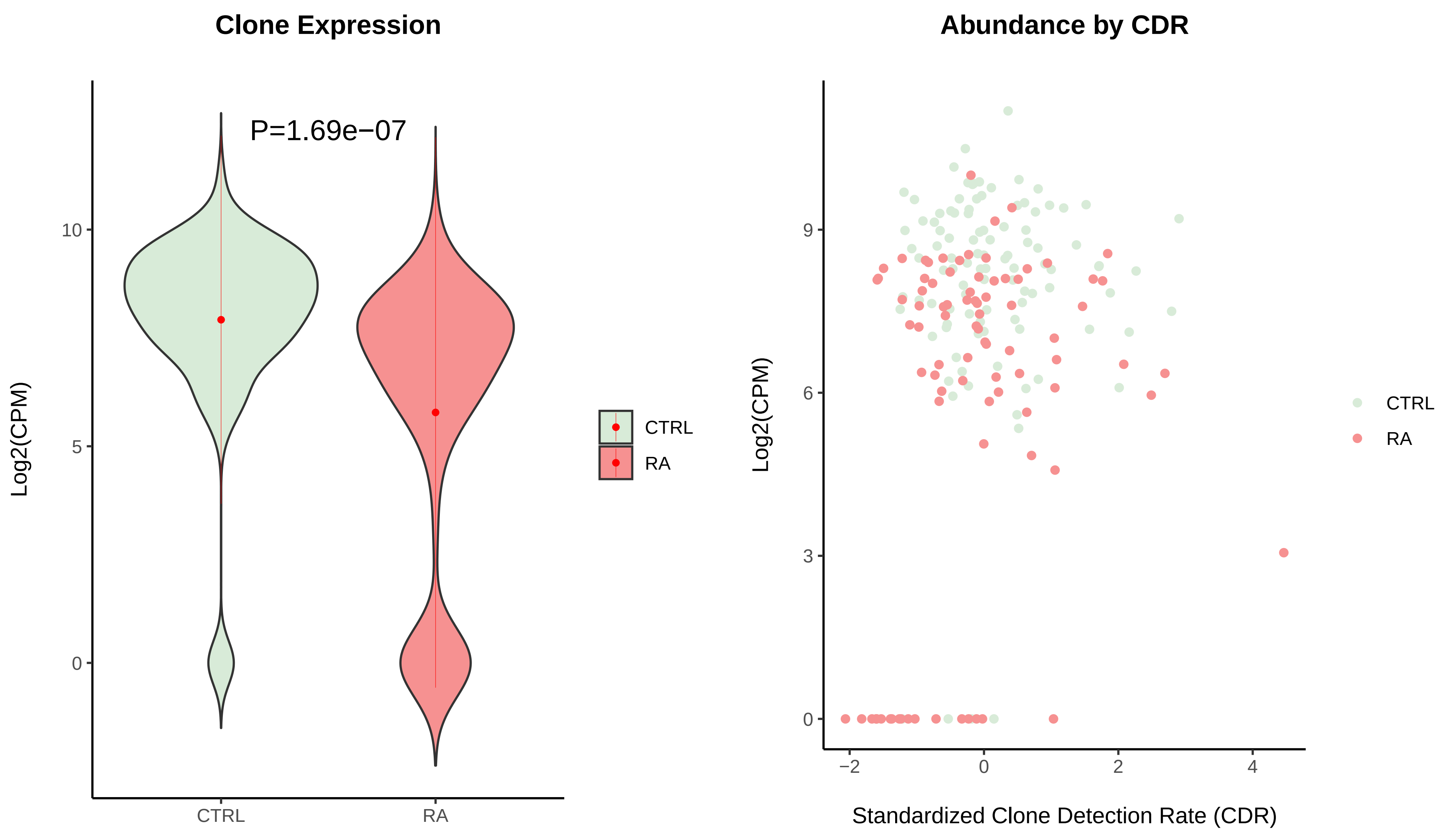

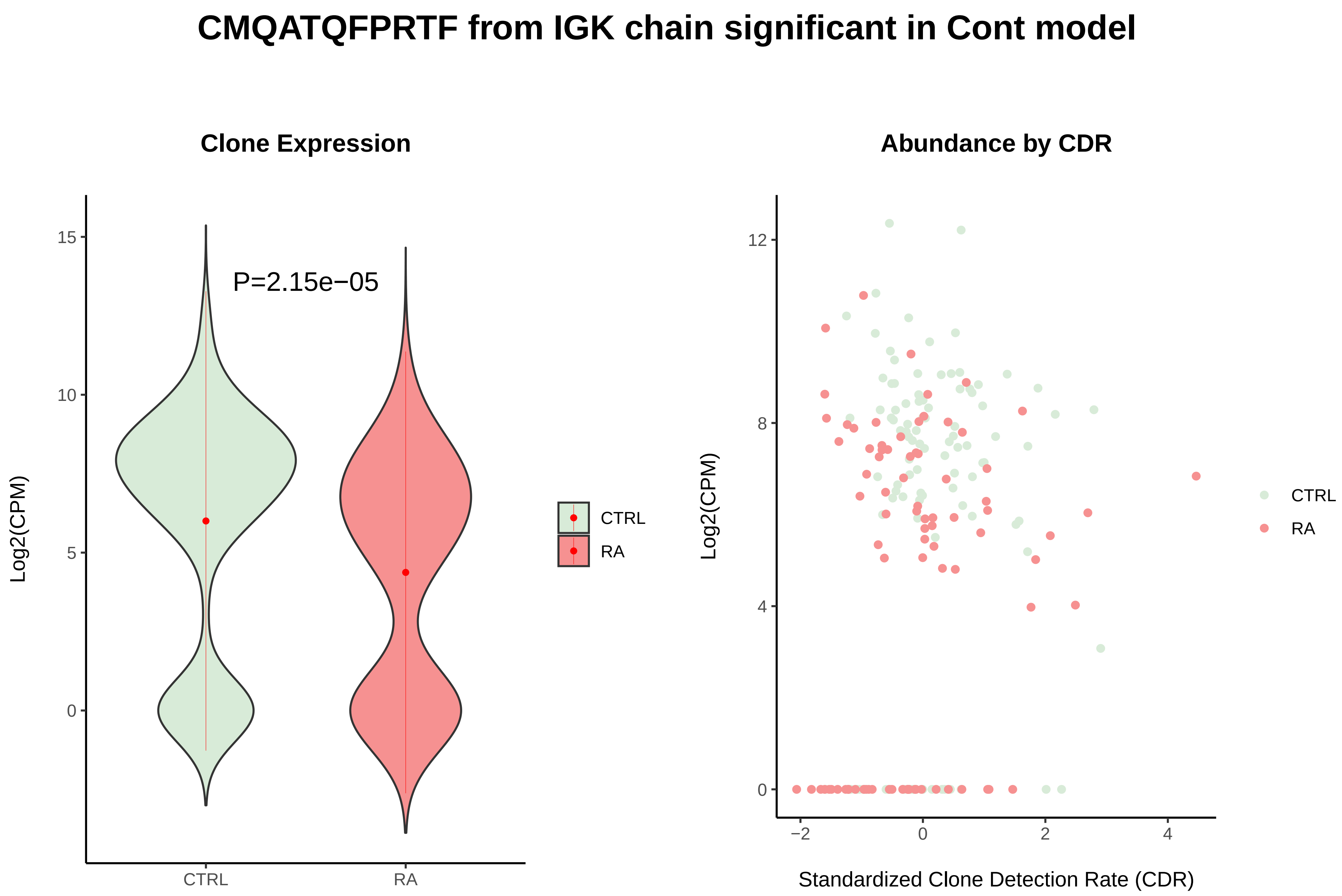
CMQATQFPRTF from IGK chain significant in Cont model
Clone Expression
P=2.15e−05
Log2(CPM)
15
10
5
0
CTRL
RA
CTRL
RA
Abundance by CDR
Log2(CPM)
12
8
4
0
−2
0
2
4
Standardized Clone Detection Rate (CDR)
CTRL
RA

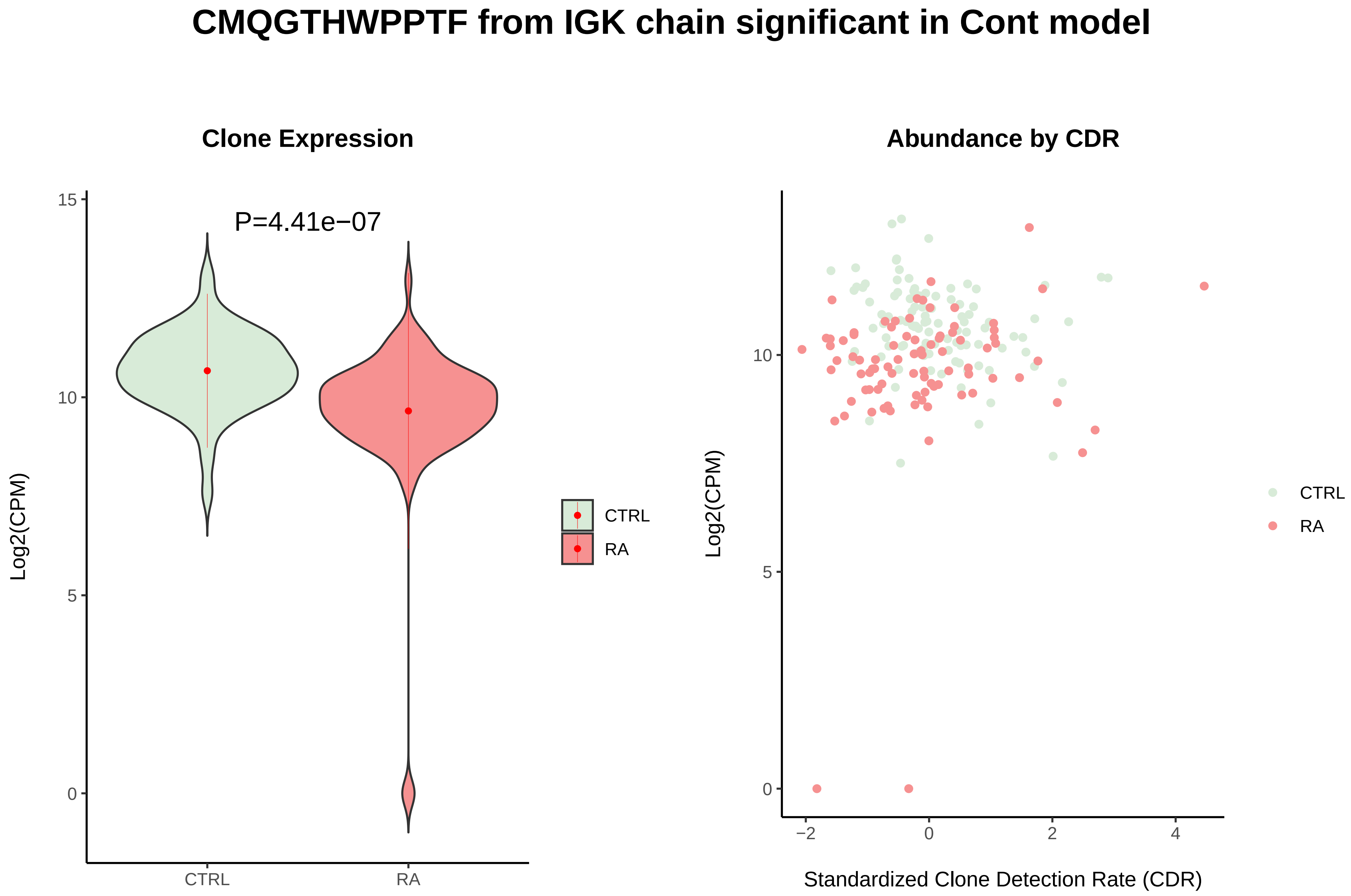

CMQGTHWPPTF from IGK chain significant in Cont model
Clone Expression
P=4.41e−07
Log2(CPM)
15
10
5
0
CTRL
RA
CTRL
RA
Abundance by CDR
Log2(CPM)
10
5
0
−2
0
2
4
Standardized Clone Detection Rate (CDR)
CTRL
RA

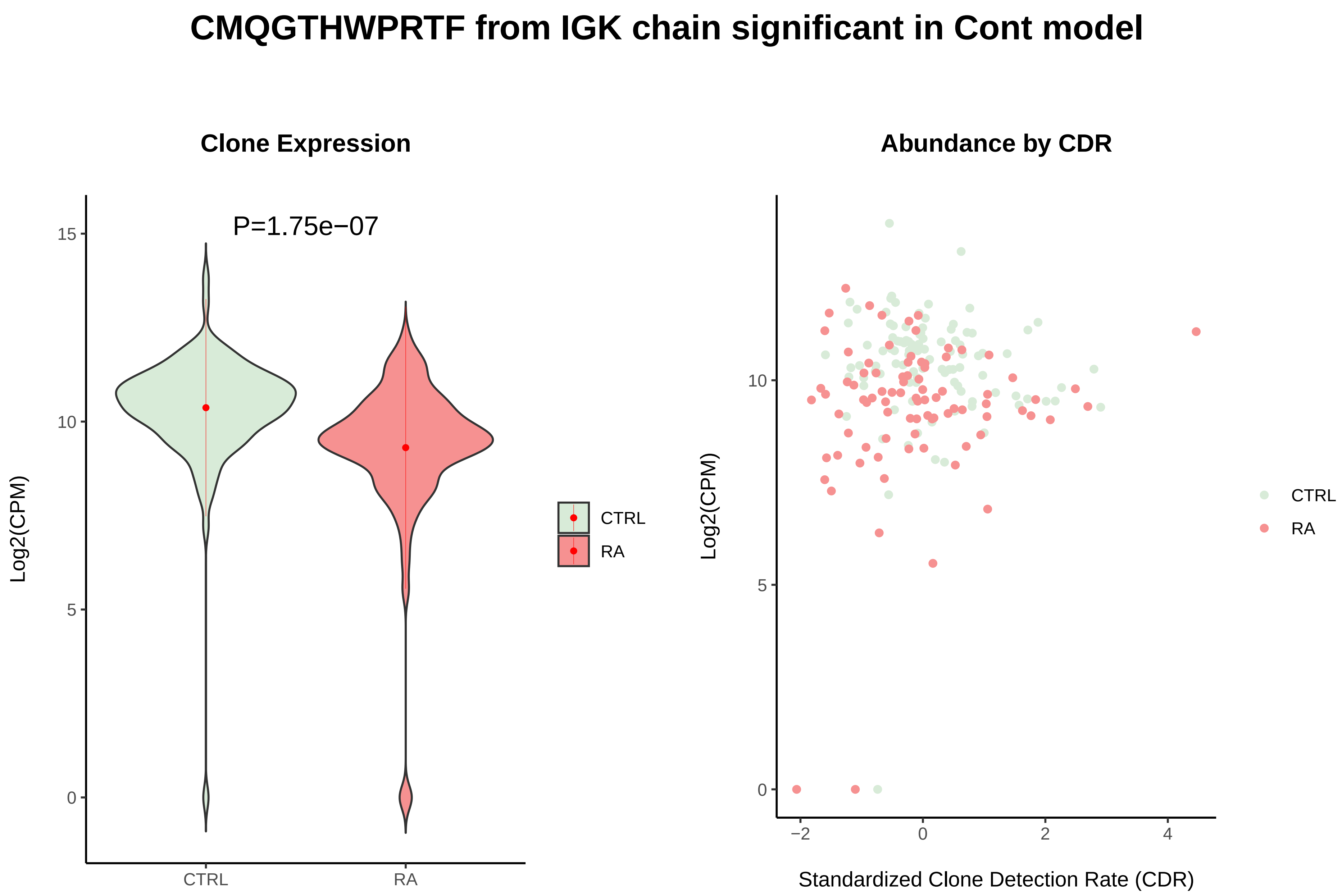

CMQGTHWPRTF from IGK chain significant in Cont model
Clone Expression
P=1.75e−07
Log2(CPM)
15
10
5
0
CTRL
RA
CTRL
RA
Abundance by CDR
Log2(CPM)
10
5
0
−2
0
2
4
Standardized Clone Detection Rate (CDR)
CTRL
RA

CMQGTHWPWTF from IGK chain significant in Cont model

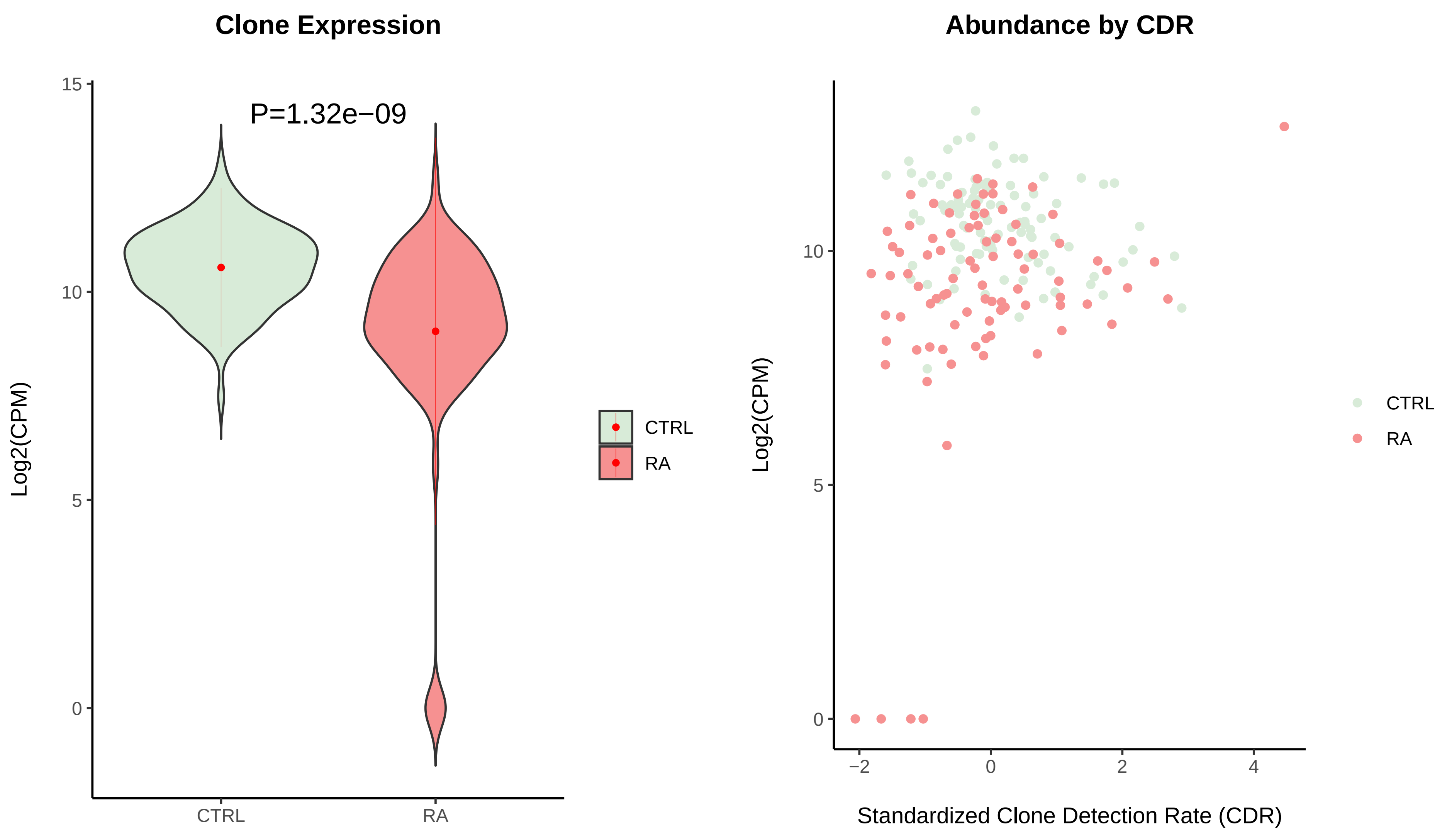

CMQRIEFPWTF from IGK chain significant in Cont model

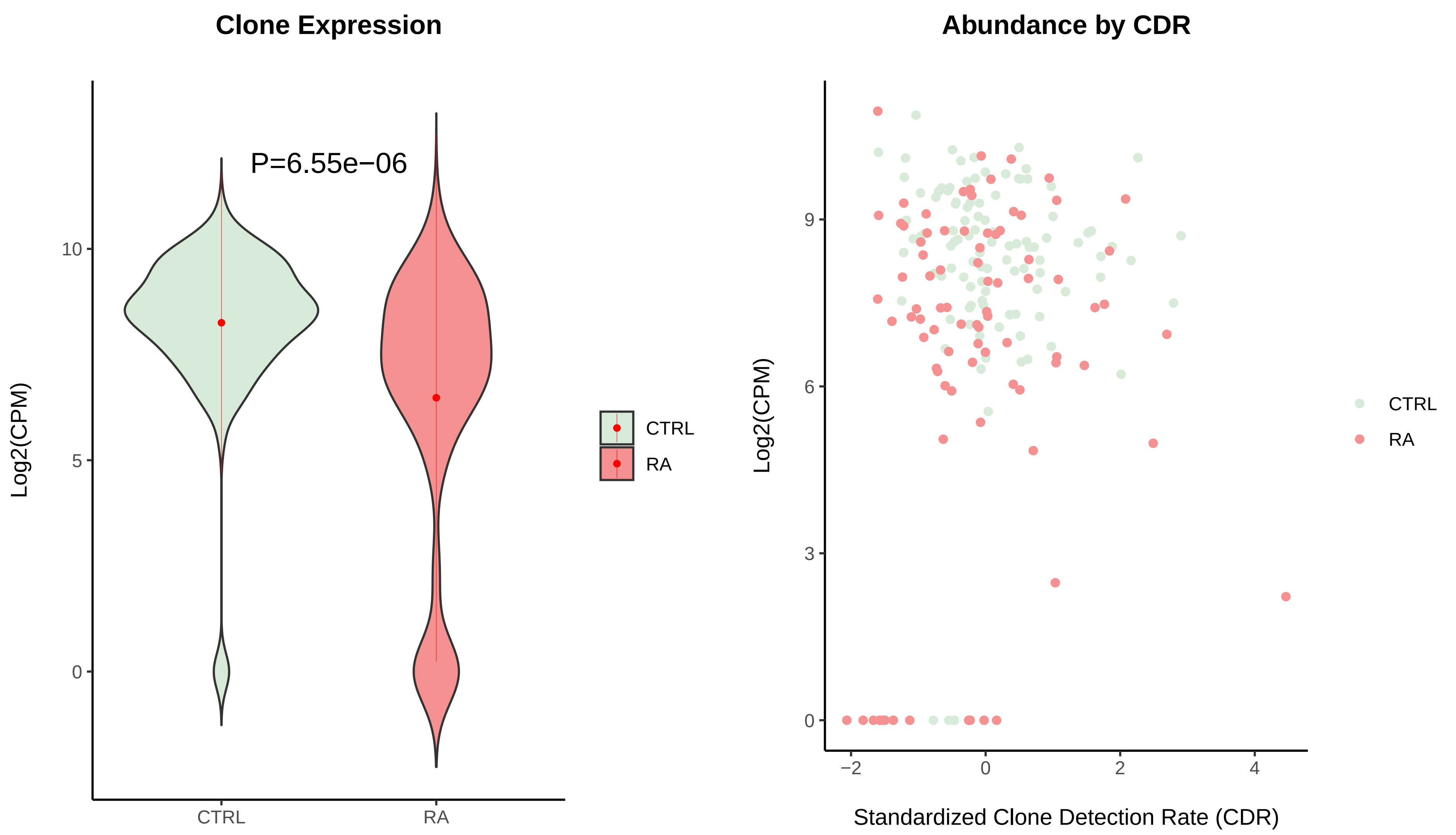

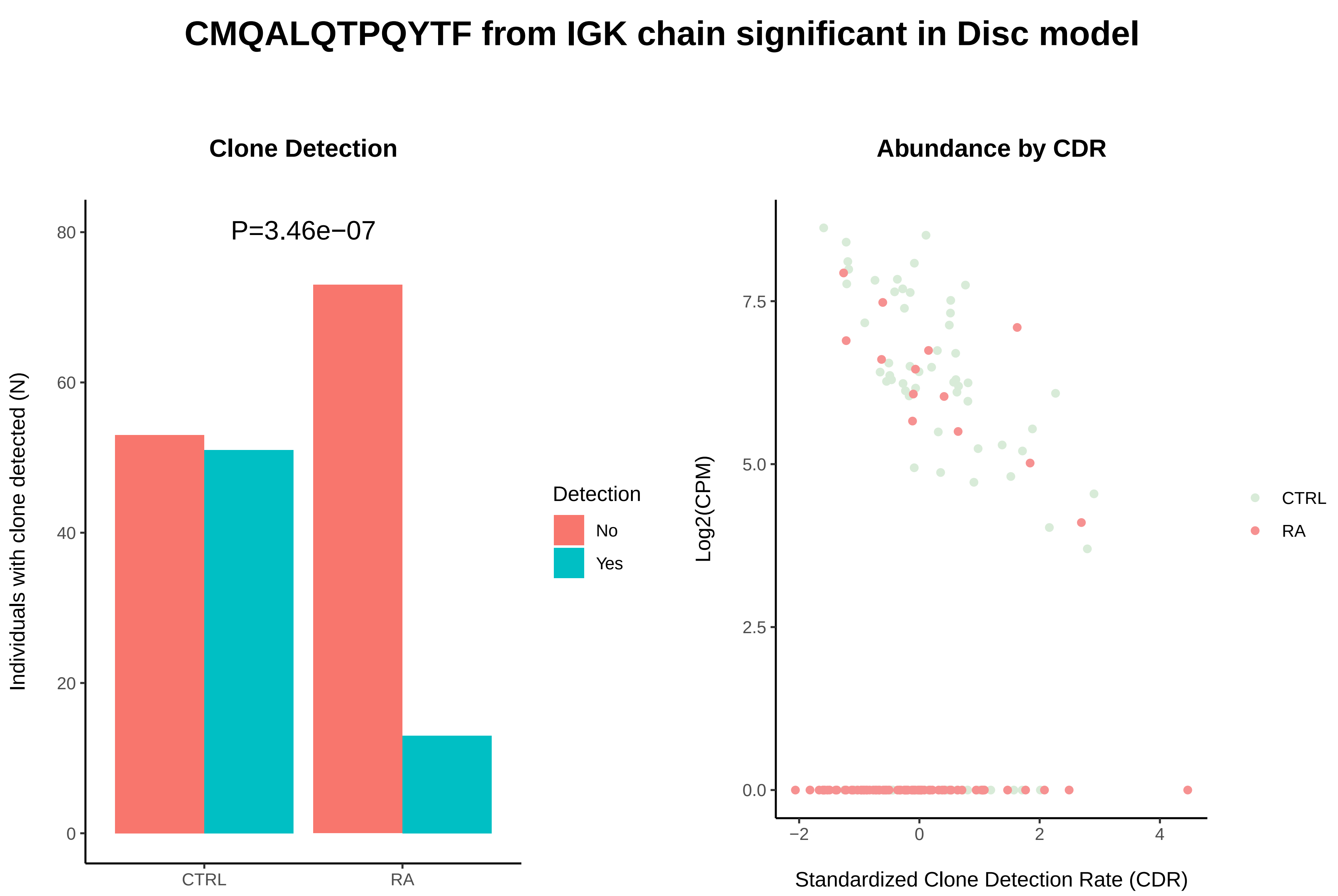

CMQALQTPQYTF from IGK chain significant in Disc model
Clone Detection
P=3.46e−07
Individuals with clone detected (N)
80
60
40
20
0
CTRL
RA
Detection
No
Yes
Abundance by CDR
Log2(CPM)
7.5
5.0
2.5
0.0
−2
0
2
4
Standardized Clone Detection Rate (CDR)
CTRL
RA

CMQALQTPRYTF from IGK chain significant in Disc model

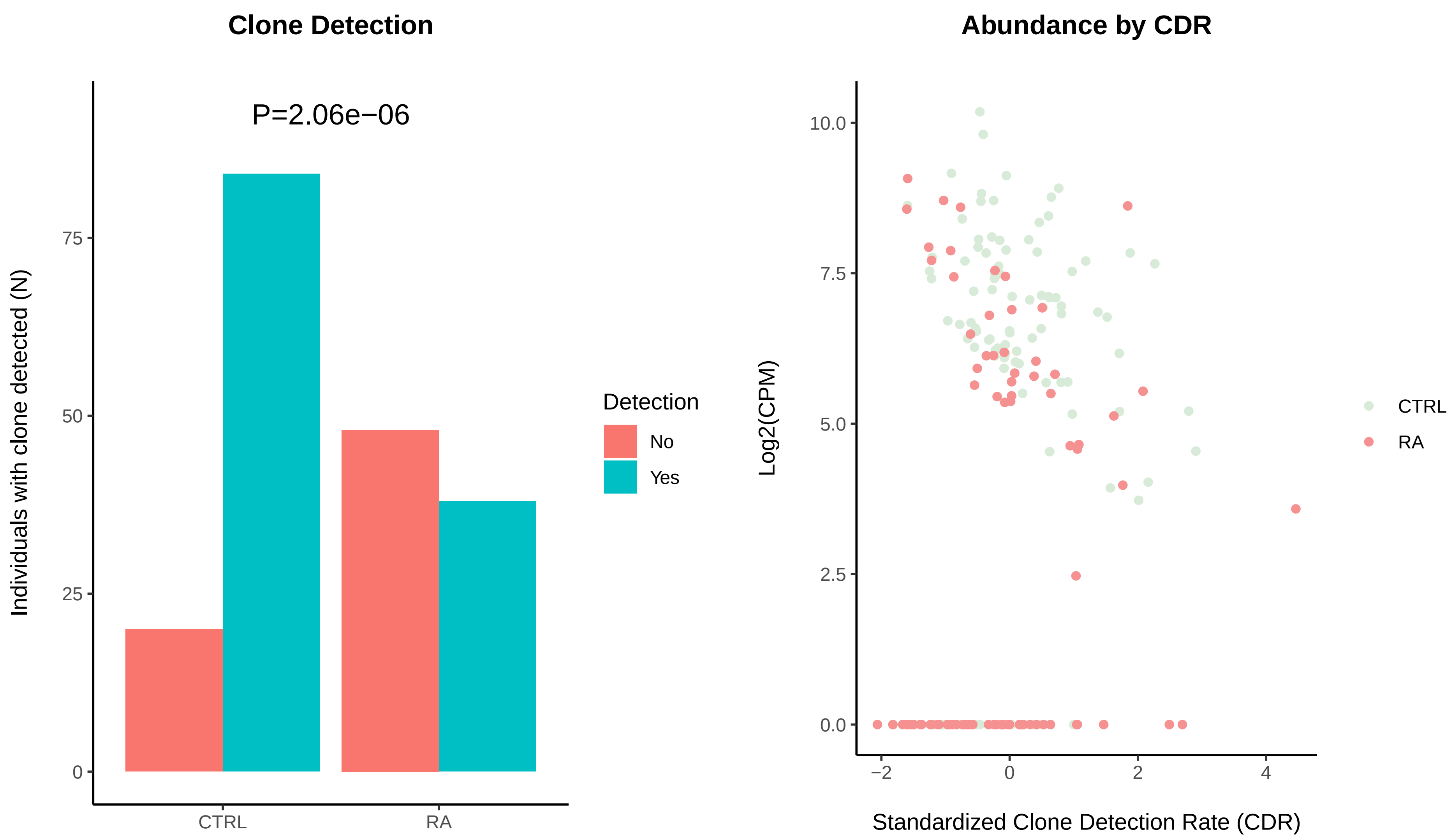

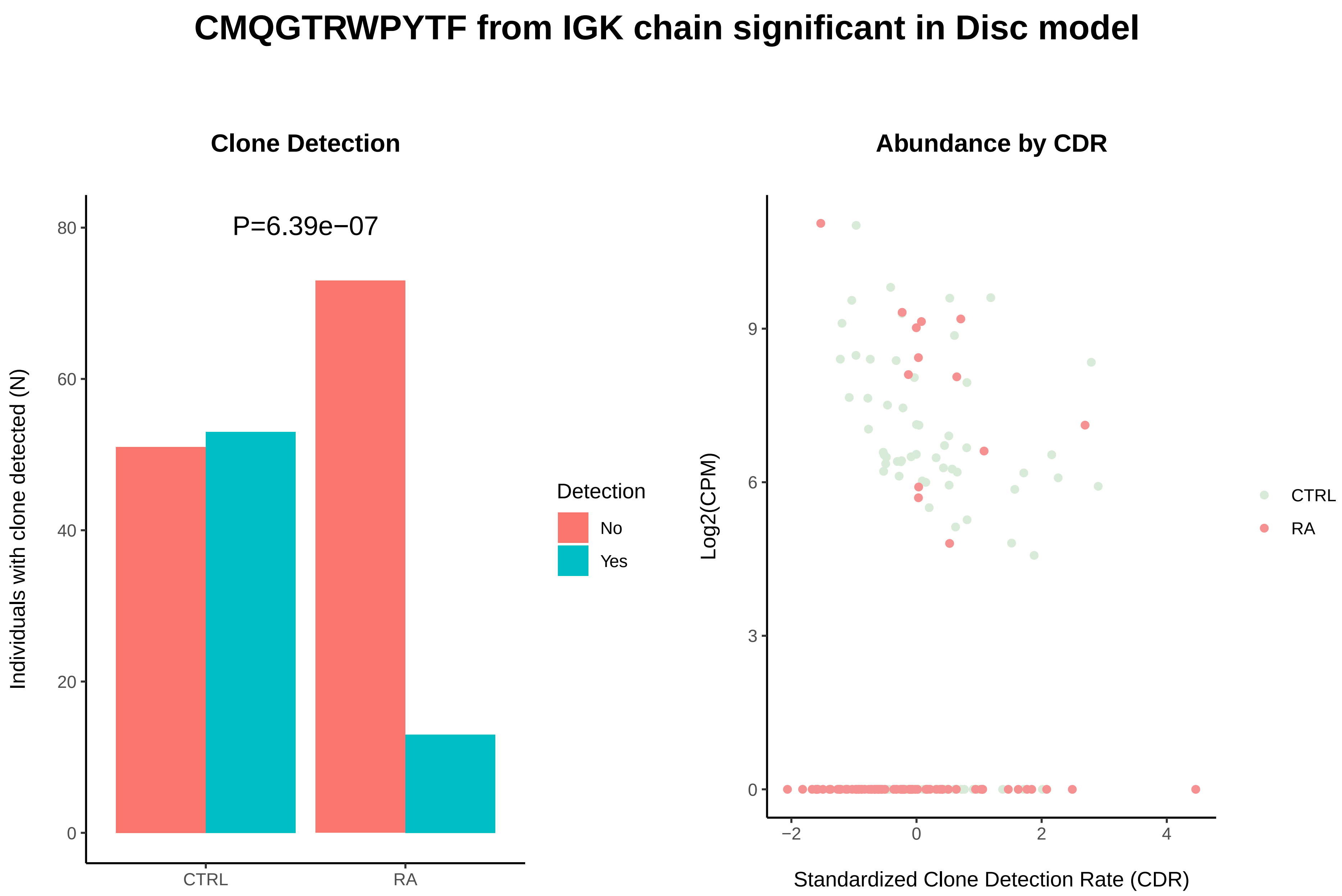
CMQGTRWPYTF from IGK chain significant in Disc model
Clone Detection
P=6.39e−07
Individuals with clone detected (N)
0
20
40
60
80
CTRL
RA
Detection
No
Yes
Abundance by CDR
Log2(CPM)
0
3
6
9
−2
0
2
4
Standardized Clone Detection Rate (CDR)
CTRL
RA

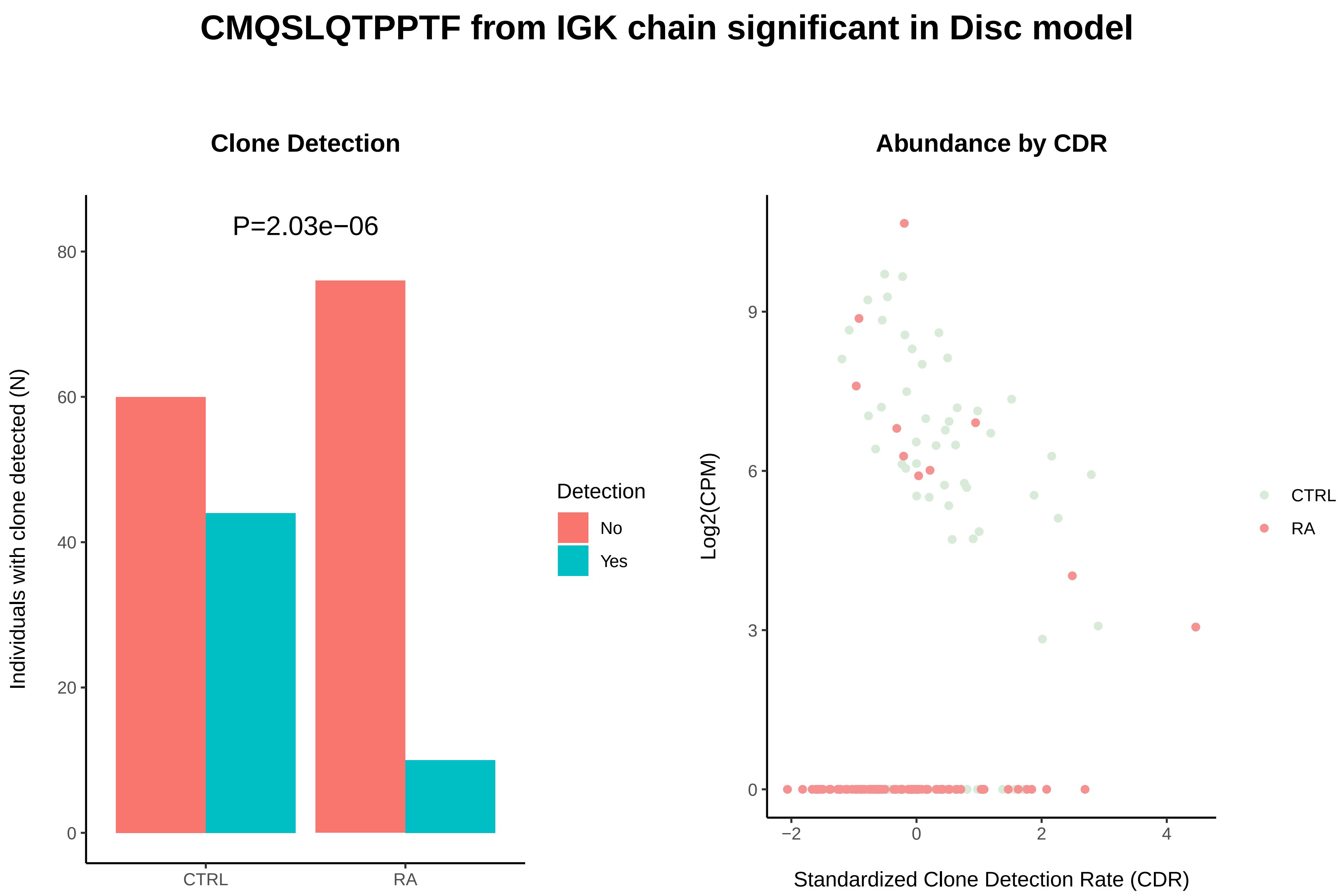

CMQSLQTPPTF from IGK chain significant in Disc model
Clone Detection
P=2.03e−06
Individuals with clone detected (N)
80
60
40
20
0
CTRL
RA
Detection
No
Yes
Abundance by CDR
Log2(CPM)
9
6
3
0
−2
0
2
4
Standardized Clone Detection Rate (CDR)
CTRL
RA
