# Supplementary material for: Seven-chain adaptive immune receptor repertoire analysis in rheumatoid arthritis reveals novel features associated with disease and clinically relevant phenotypes
Source: Genome Biol. 2024 Mar 11;25:68. doi: 10.1186/s13059-024-03210-0 (PMC10926600; doi:10.1186/s13059-024-03210-0)

Fig S5. Graphical representation of the significant associations between TRA/TRB/IGL/IGK k-mers and rheumatoid arthritis. Significant associations detected by the Hurdle or continuous models are represented using violin plots, where the k-mer expression is plotted separately for each phenotype. Significant associations detected by the discrete model are represented using bar plots, where the number of individuals with clones harboring the k-mer are plotted separately for each phenotype. For all significant associations (FDR<0.05), the k-mer expression is also plotted against the standardized k-mer detection rate. Abbreviations: Cont, continuous model; CPM, count per million on the logarithmic scale; CTRL, healthy individuals; Disc, discrete model; KDR, k-mer detection rate; P, p-value; RA, rheumatoid arthritis.

AENG from TRA chain significant in Hurdle model

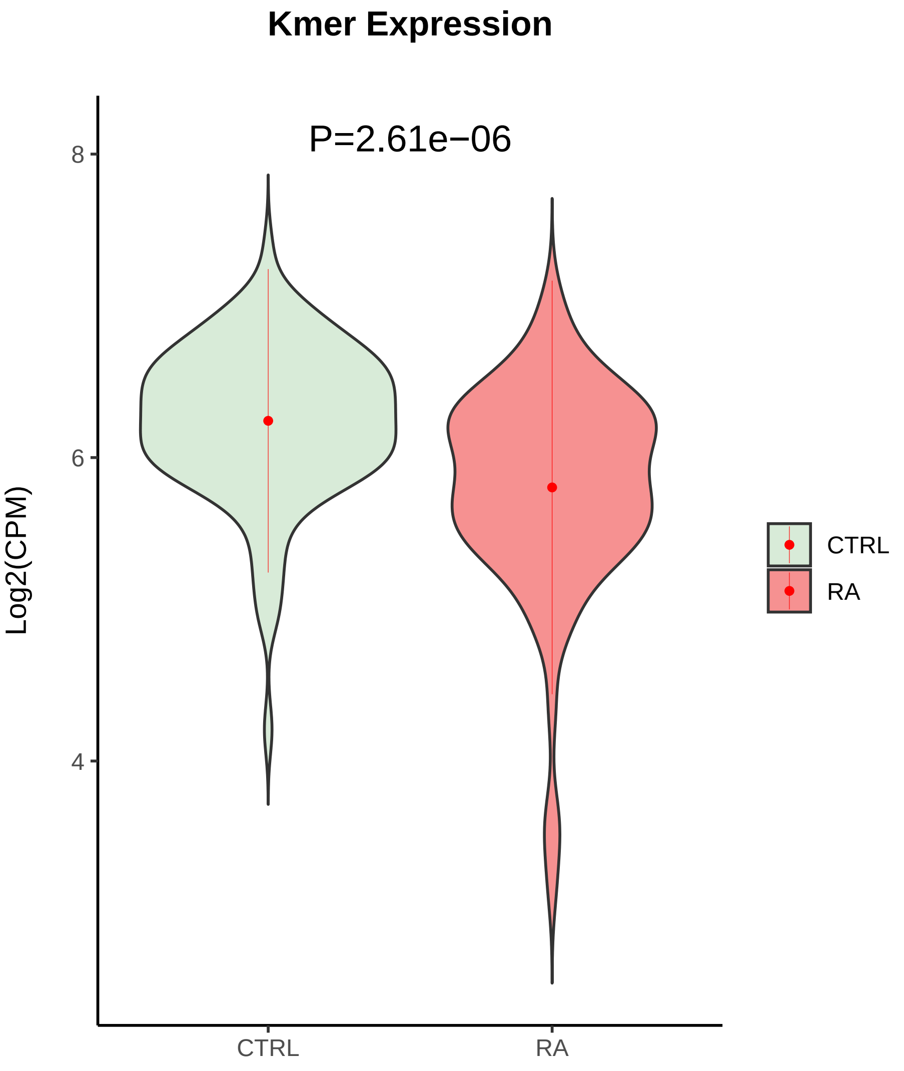

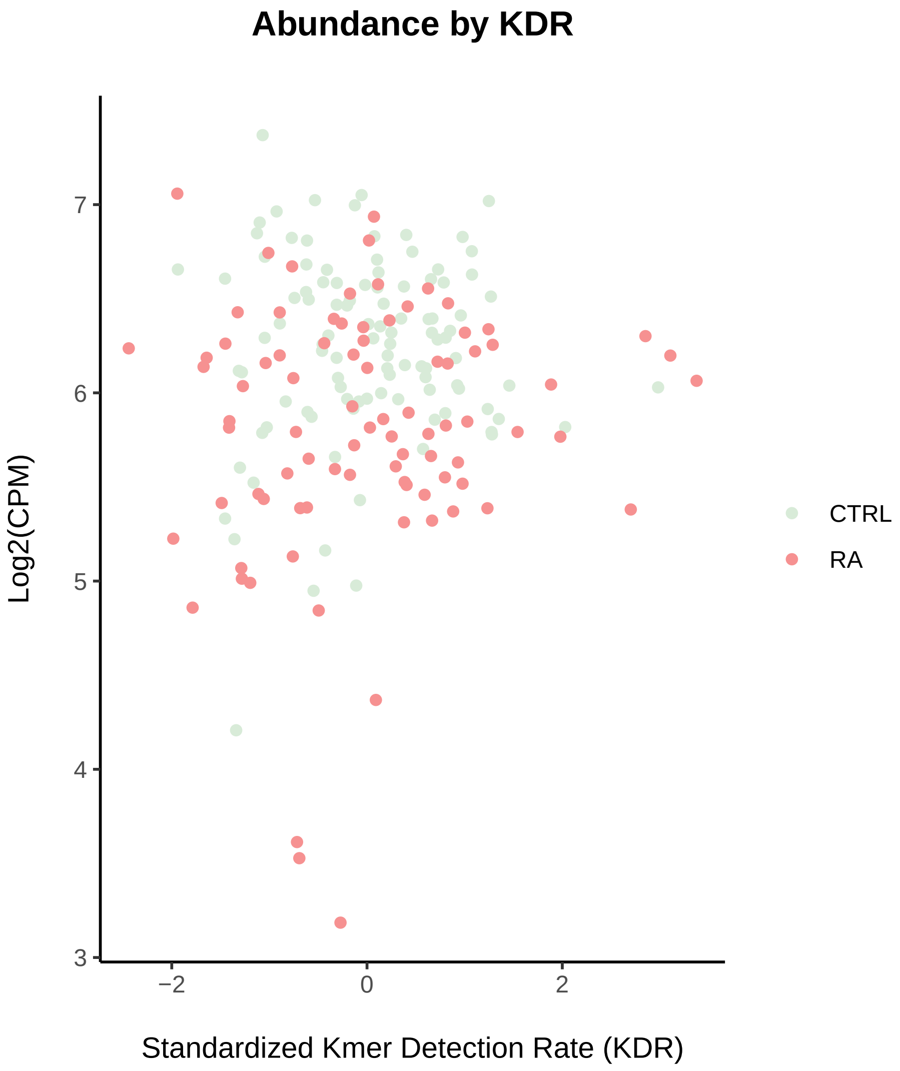

AENR from TRA chain significant in Hurdle model

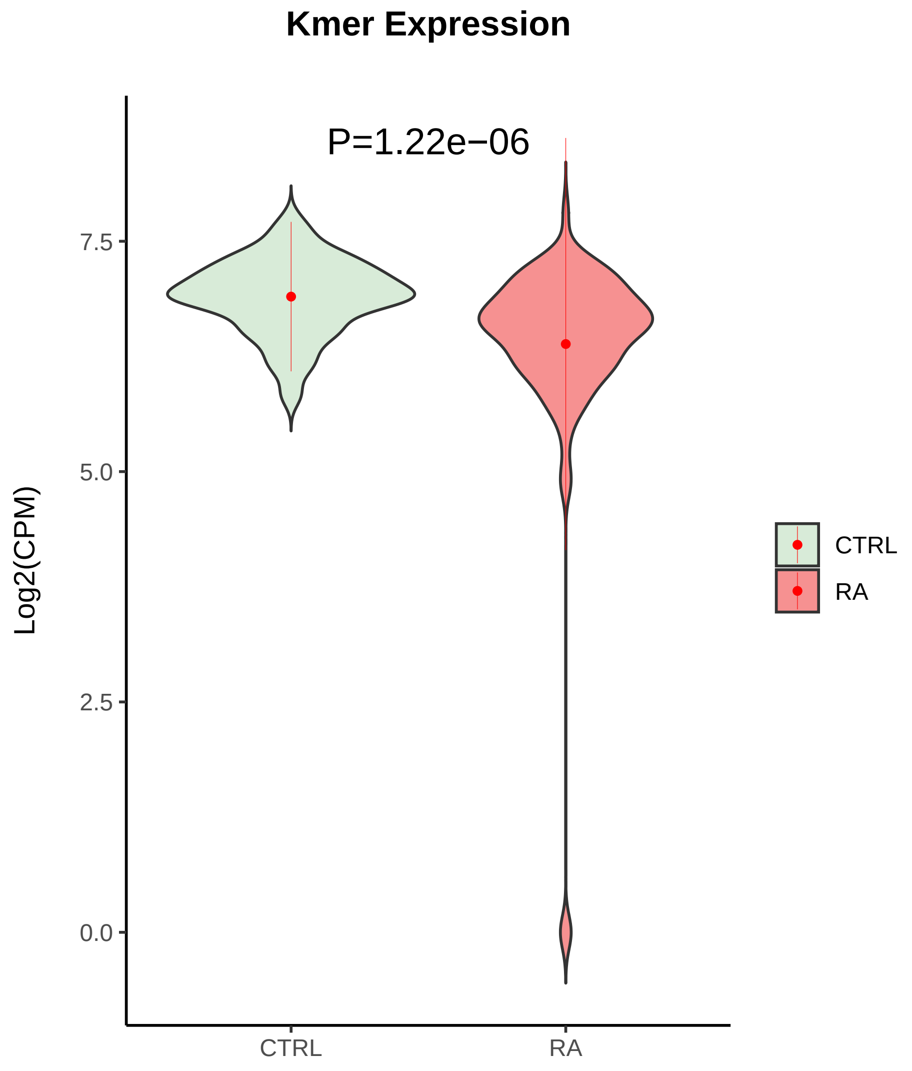

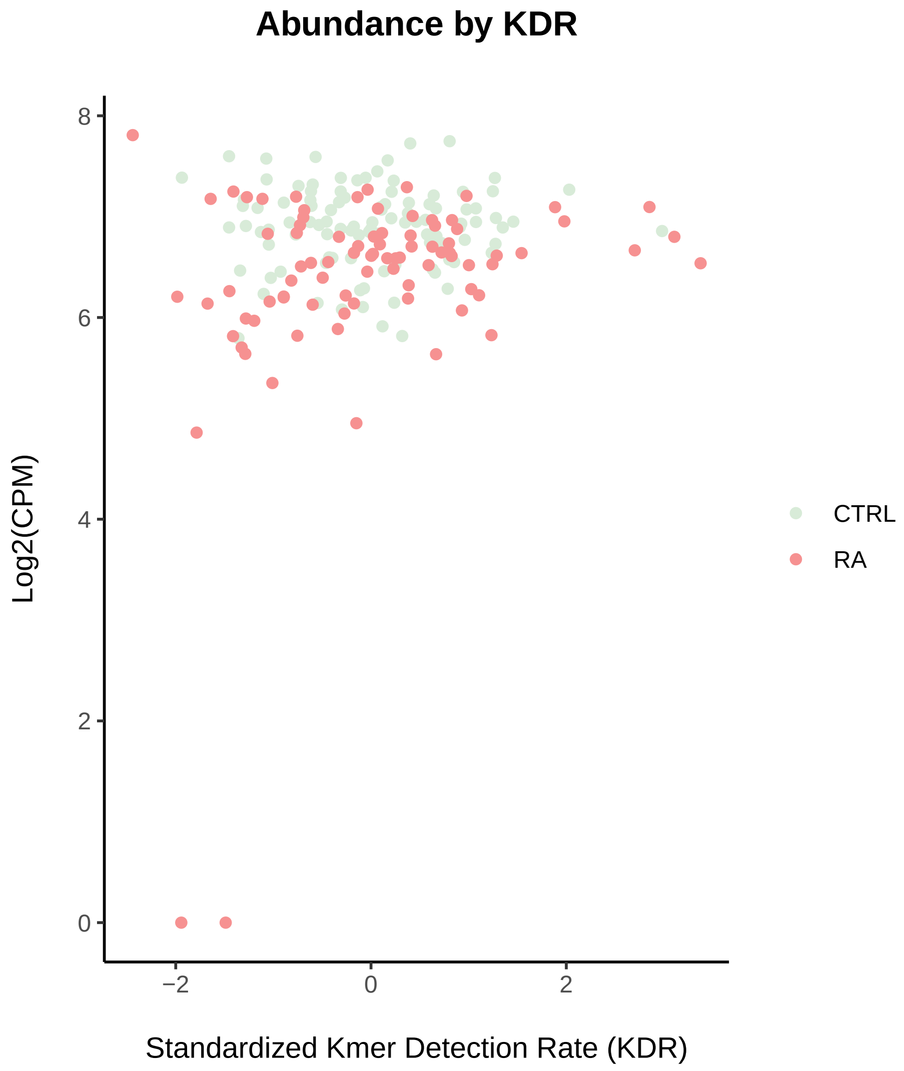

AVQA from TRA chain significant in Hurdle model

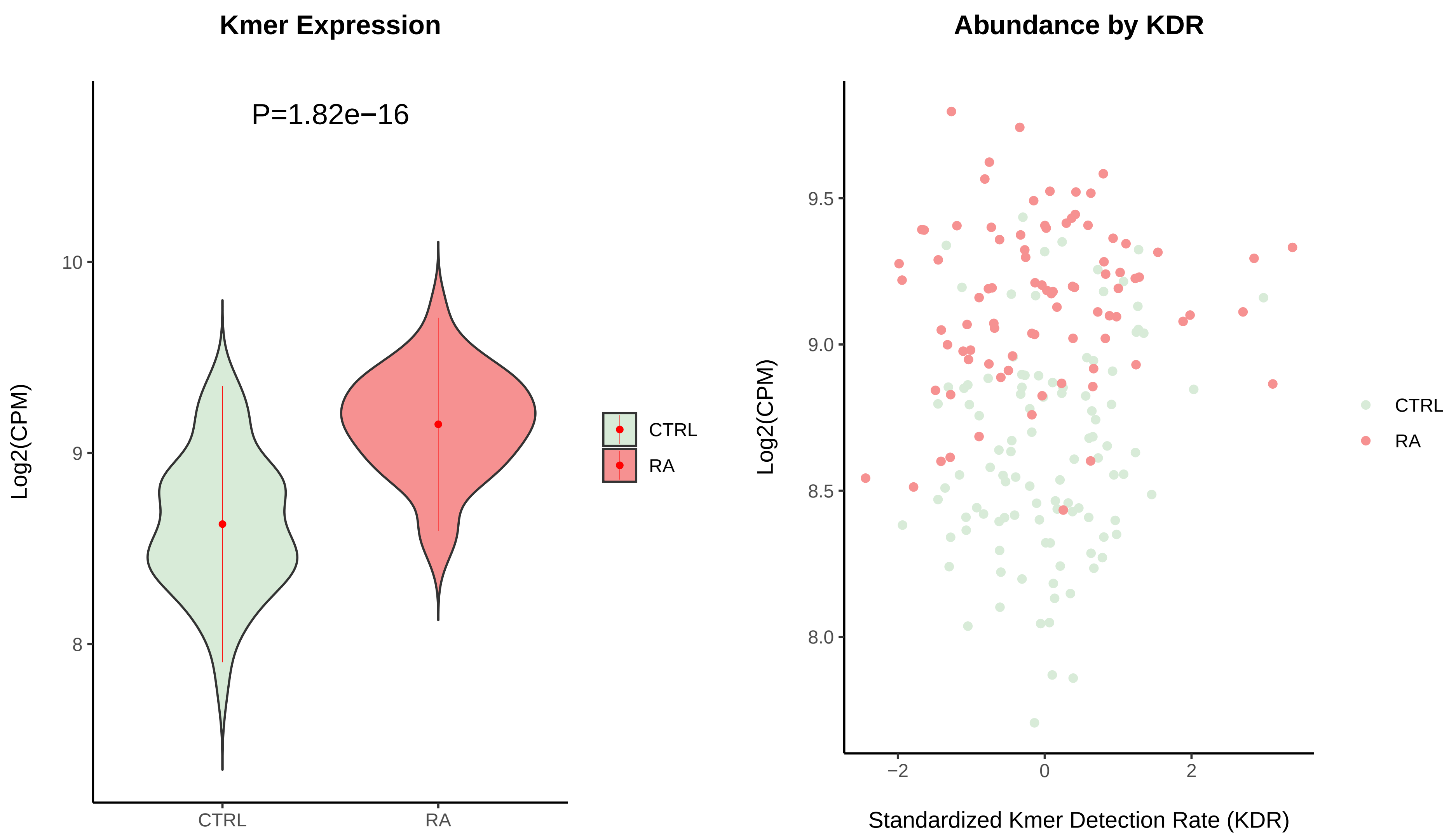

AVQG from TRA chain significant in Hurdle model

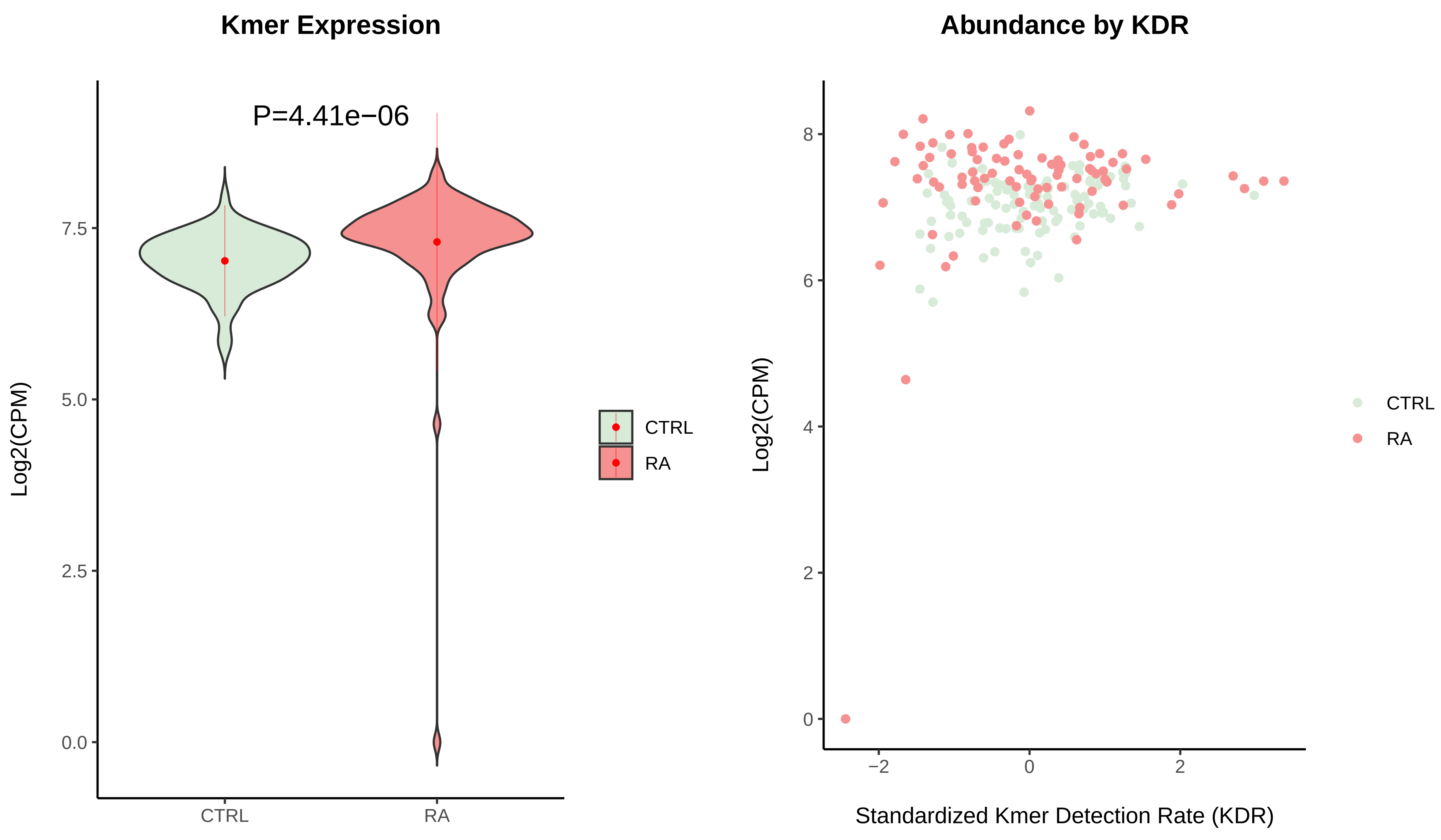

AVQS from TRA chain significant in Hurdle model

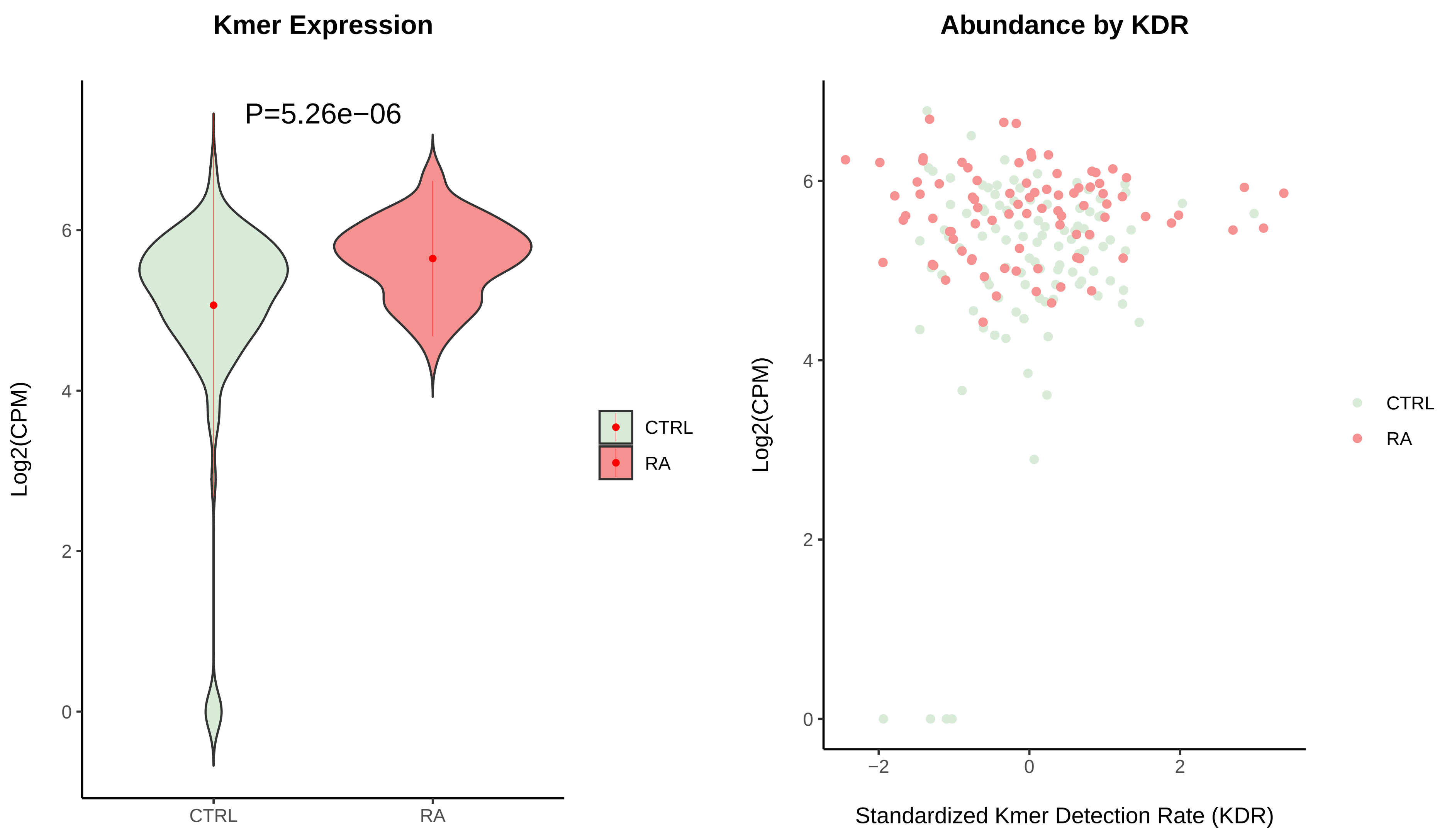

AVQV from TRA chain significant in Hurdle model

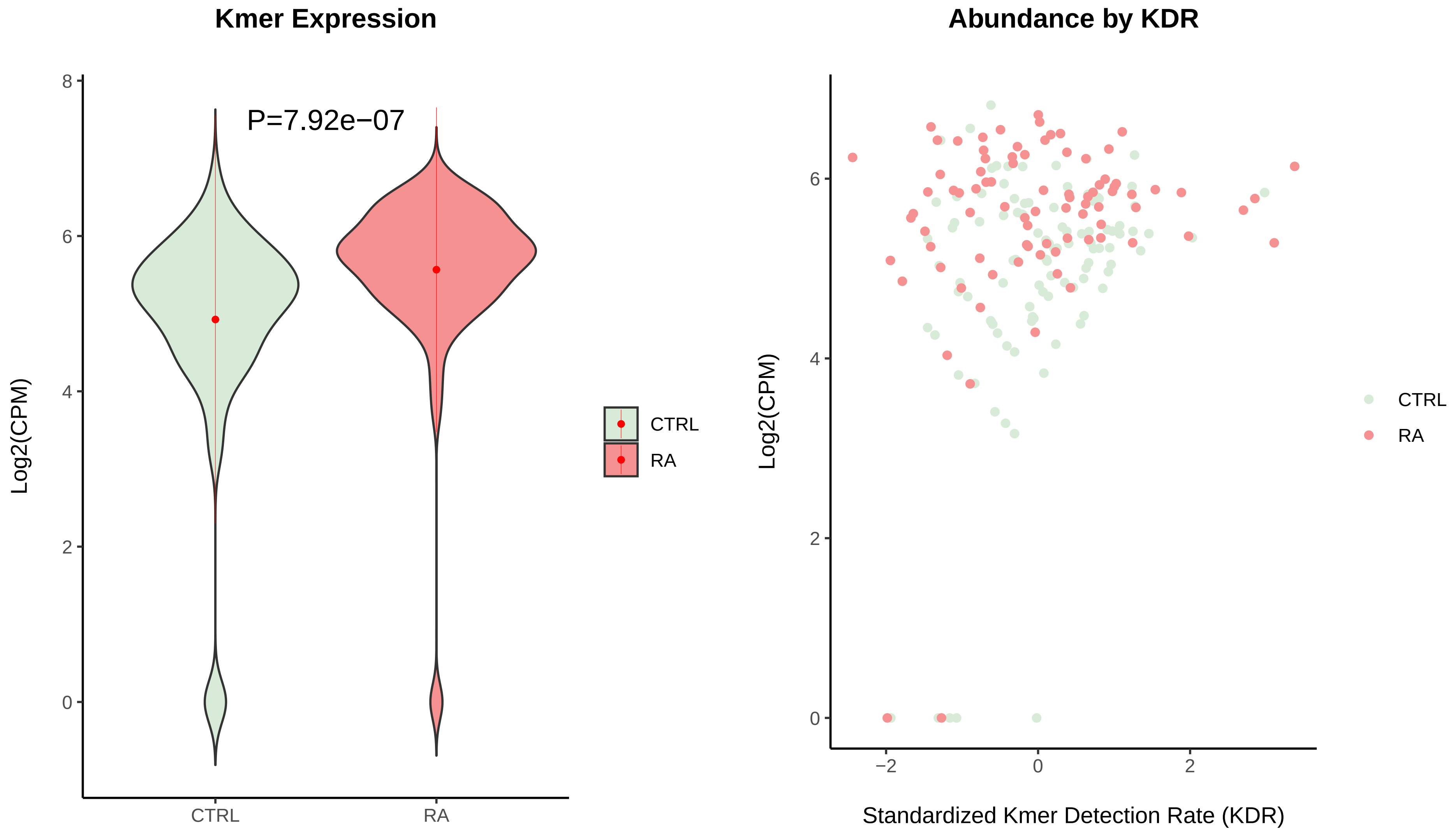

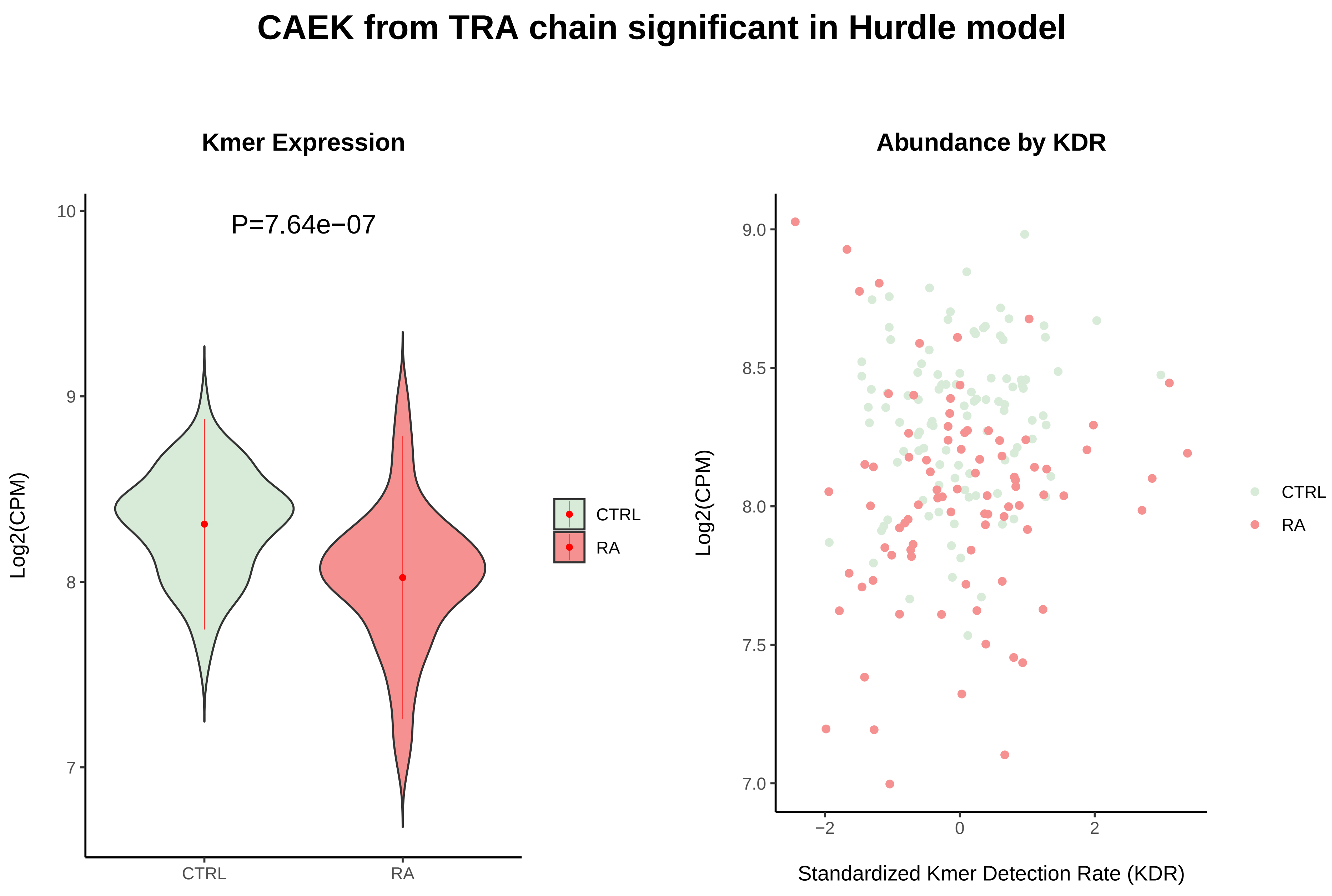

CAEK from TRA chain significant in Hurdle model
Kmer Expression
P=7.64e−07
Log2(CPM)
10
9
8
7
CTRL
RA
Abundance by KDR
Log2(CPM)
9.0
8.5
8.0
7.5
7.0
−2
0
2
Standardized Kmer Detection Rate (KDR)

CAEN from TRA chain significant in Hurdle model

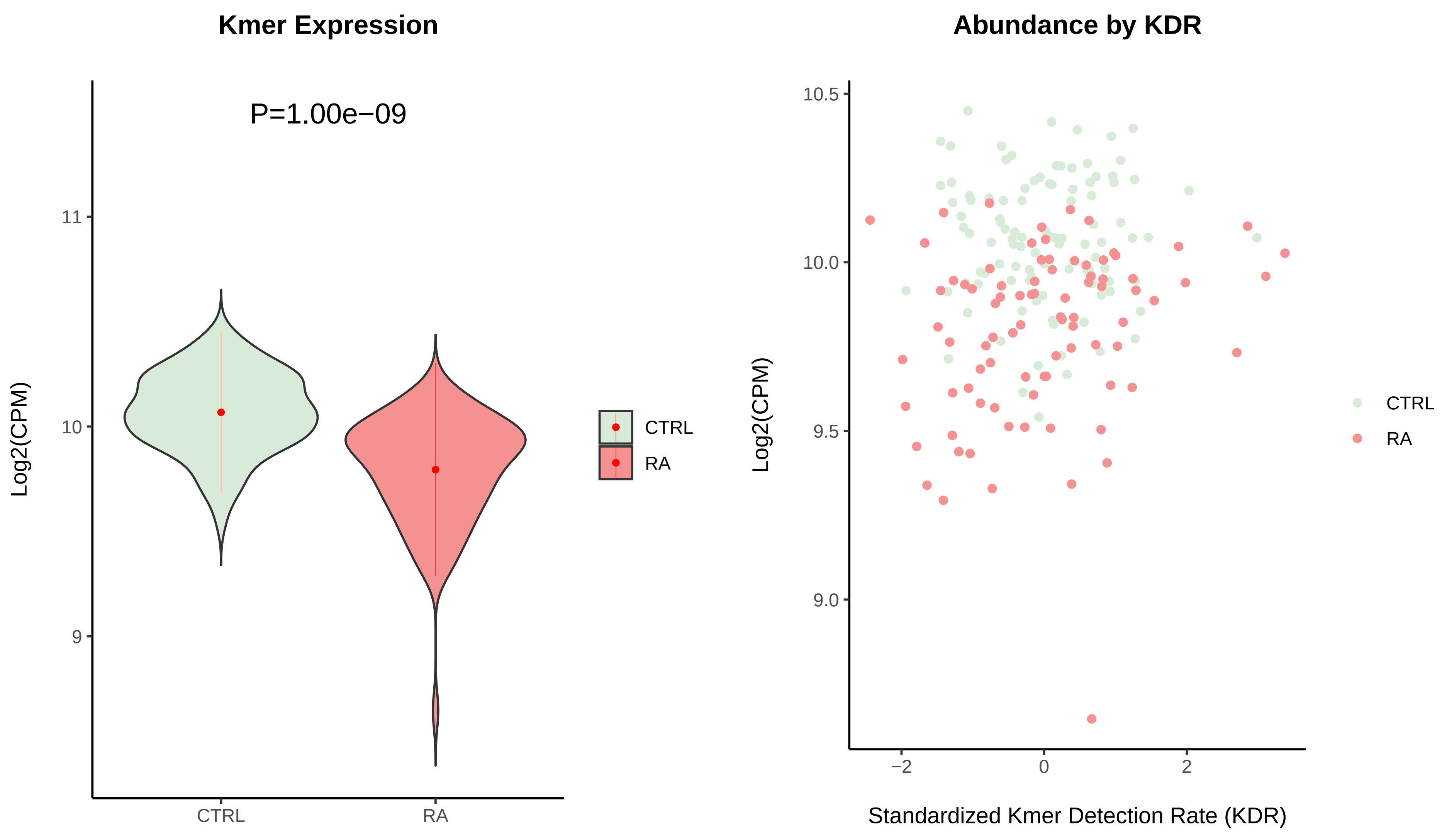

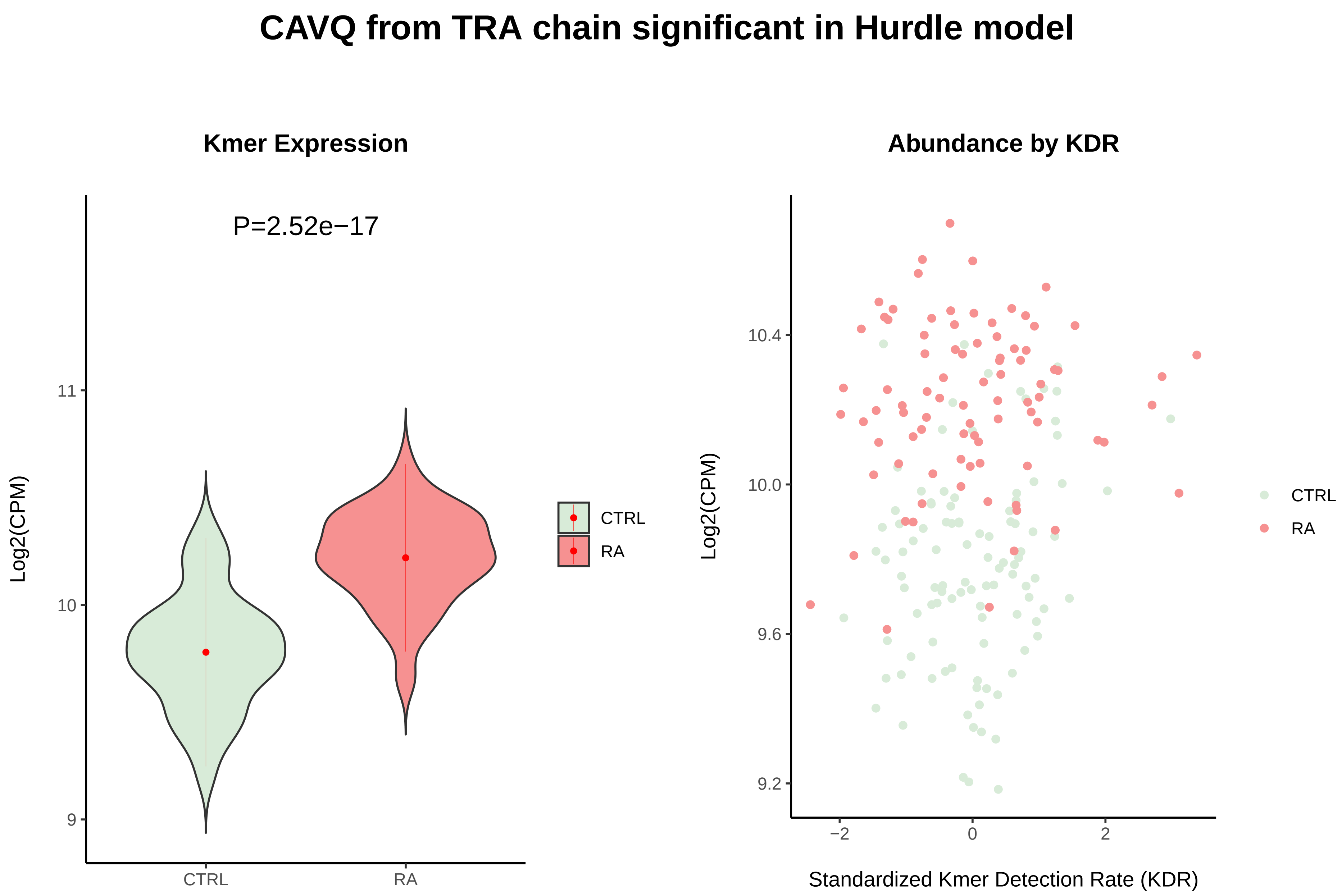
CAVQ from TRA chain significant in Hurdle model
Kmer Expression
P=2.52e−17
Log2(CPM)
11
10
9
CTRL
RA
CTRL
RA
Abundance by KDR
Log2(CPM)
10.4
10.0
9.6
9.2
−2
0
2
Standardized Kmer Detection Rate (KDR)
CTRL
RA

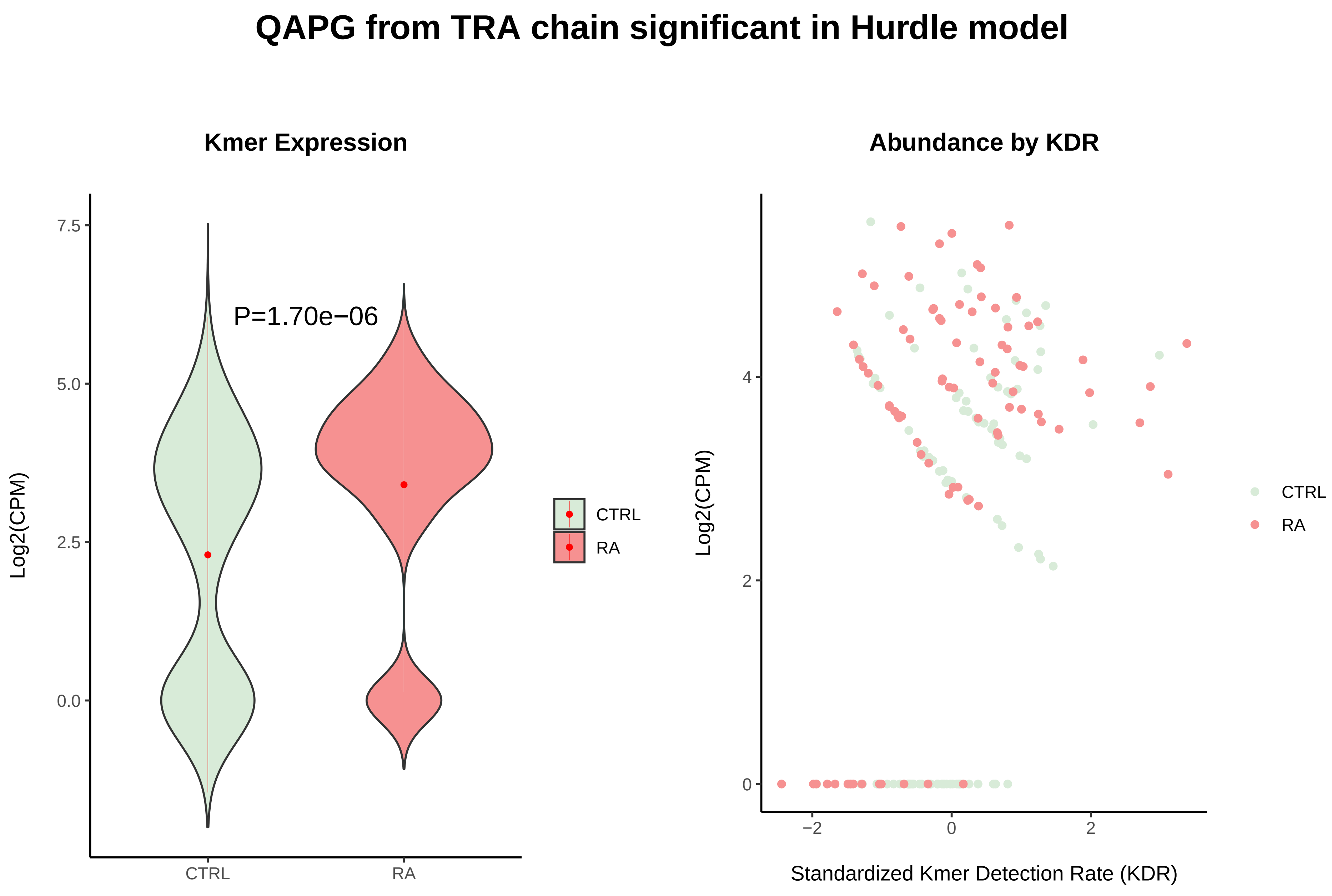
QAPG from TRA chain significant in Hurdle model
Kmer Expression
P=1.70e−06
Log2(CPM)
7.5
5.0
2.5
0.0
CTRL
RA
CTRL
RA
Abundance by KDR
Log2(CPM)
4
2
0
−2
0
2
Standardized Kmer Detection Rate (KDR)
CTRL
RA

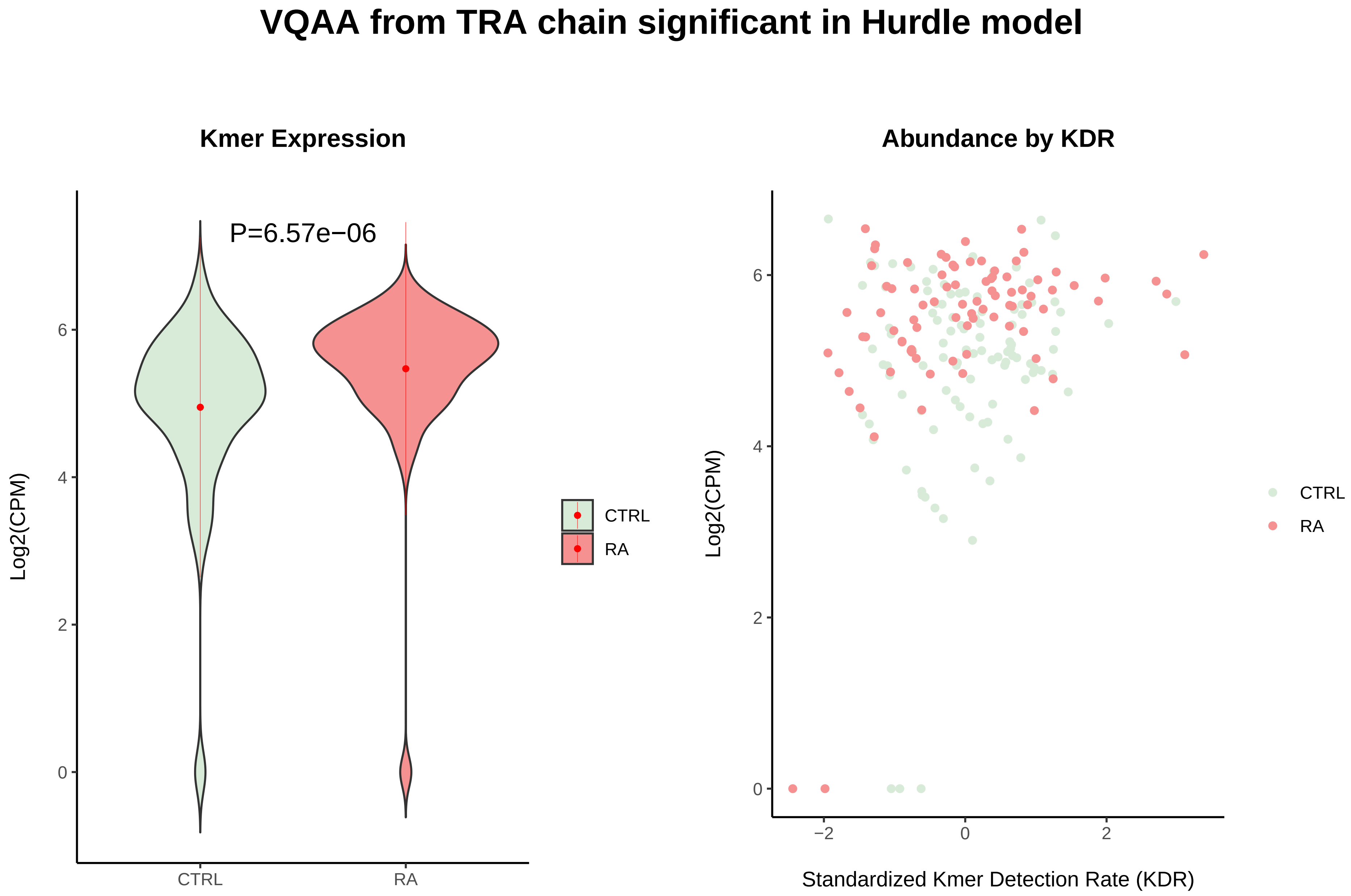
VQAA from TRA chain significant in Hurdle model
Kmer Expression
P=6.57e−06
Log2(CPM)
0
2
4
6
CTRL
RA
CTRL
RA
Abundance by KDR
Log2(CPM)
0
2
4
6
−2
0
2
Standardized Kmer Detection Rate (KDR)
CTRL
RA

VQAG from TRA chain significant in Hurdle model

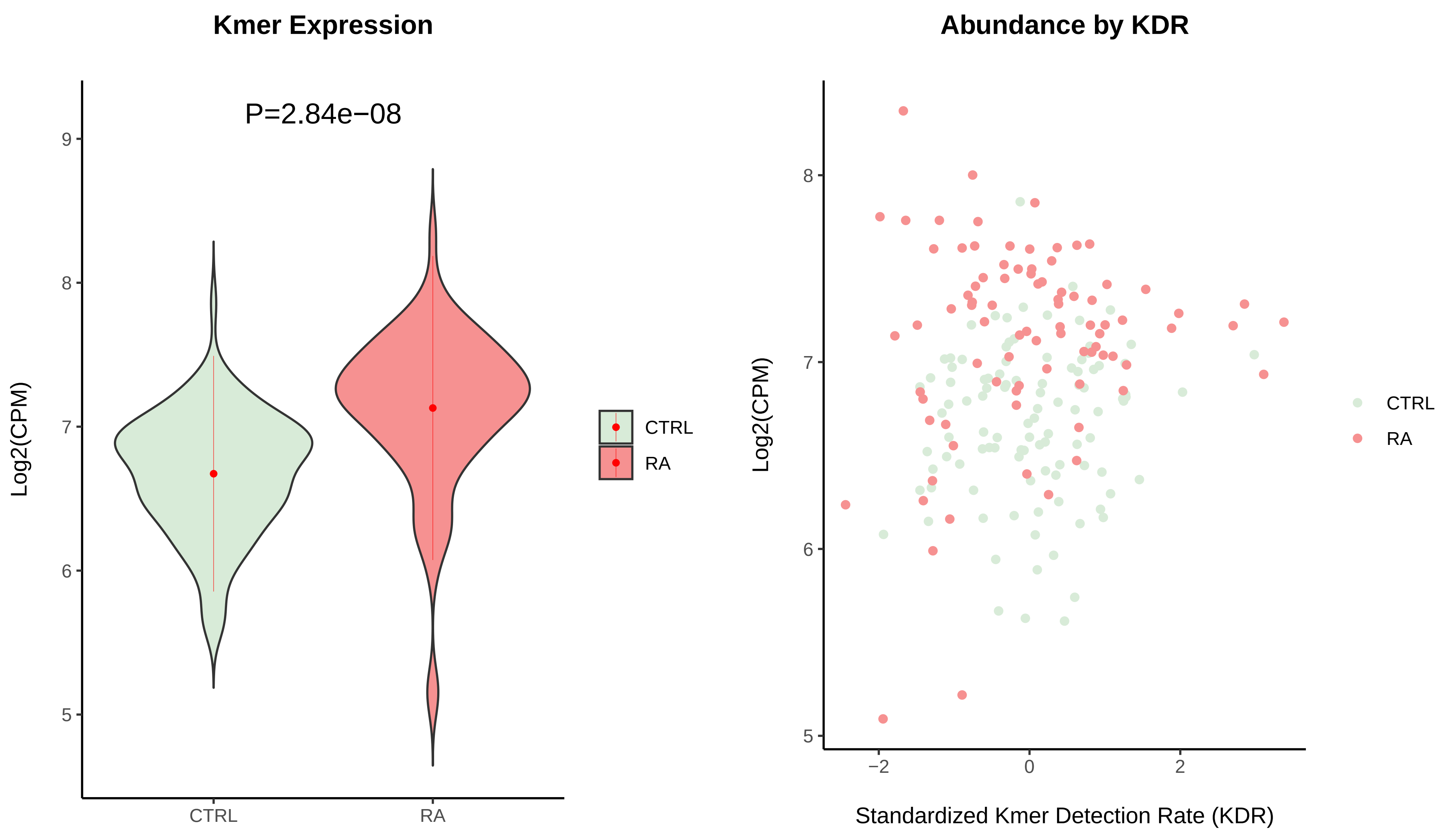

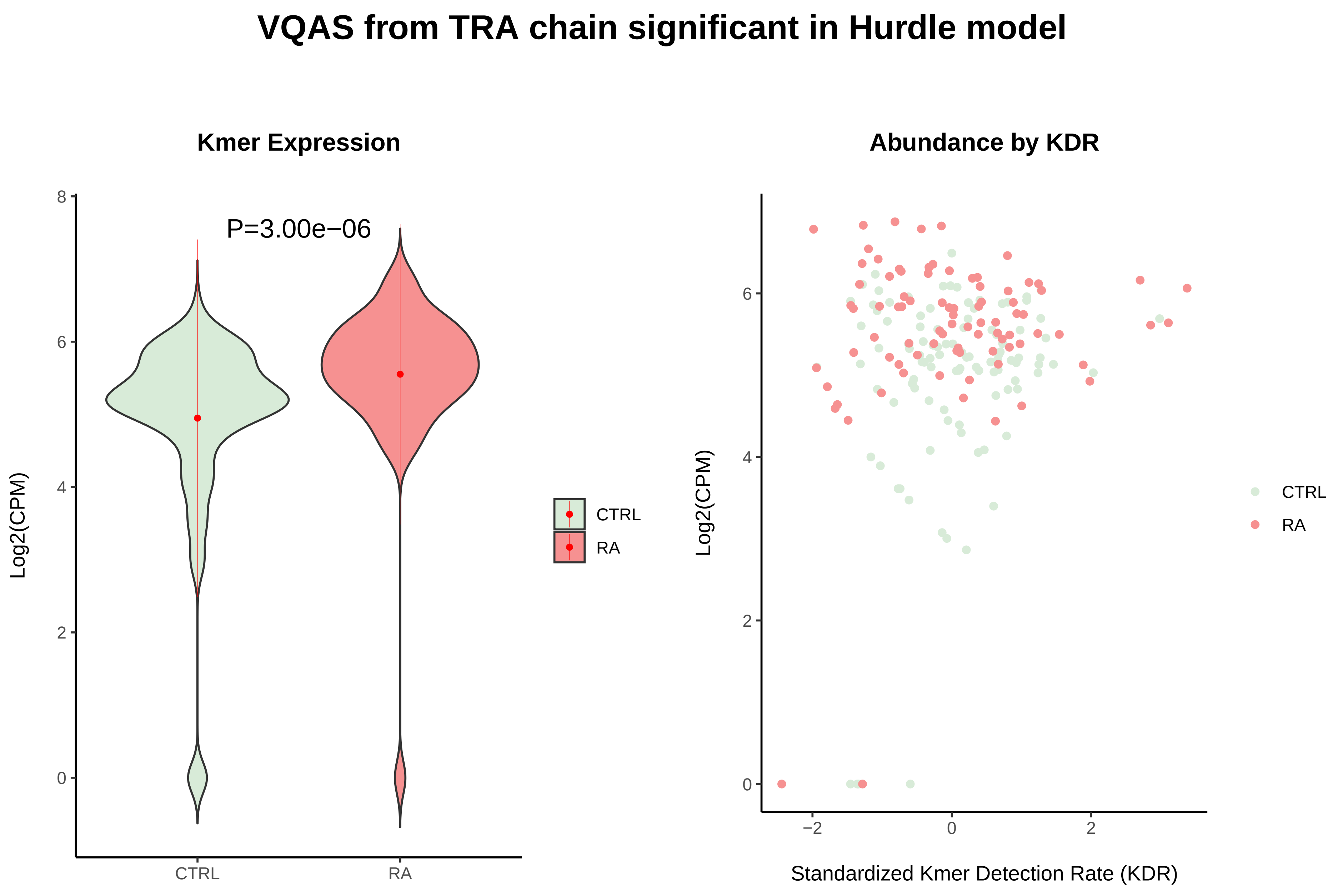
VQAS from TRA chain significant in Hurdle model
Kmer Expression
P=3.00e−06
Log2(CPM)
8
6
4
2
0
CTRL
RA
CTRL
RA
Abundance by KDR
Log2(CPM)
6
4
2
0
−2
0
2
Standardized Kmer Detection Rate (KDR)
CTRL
RA

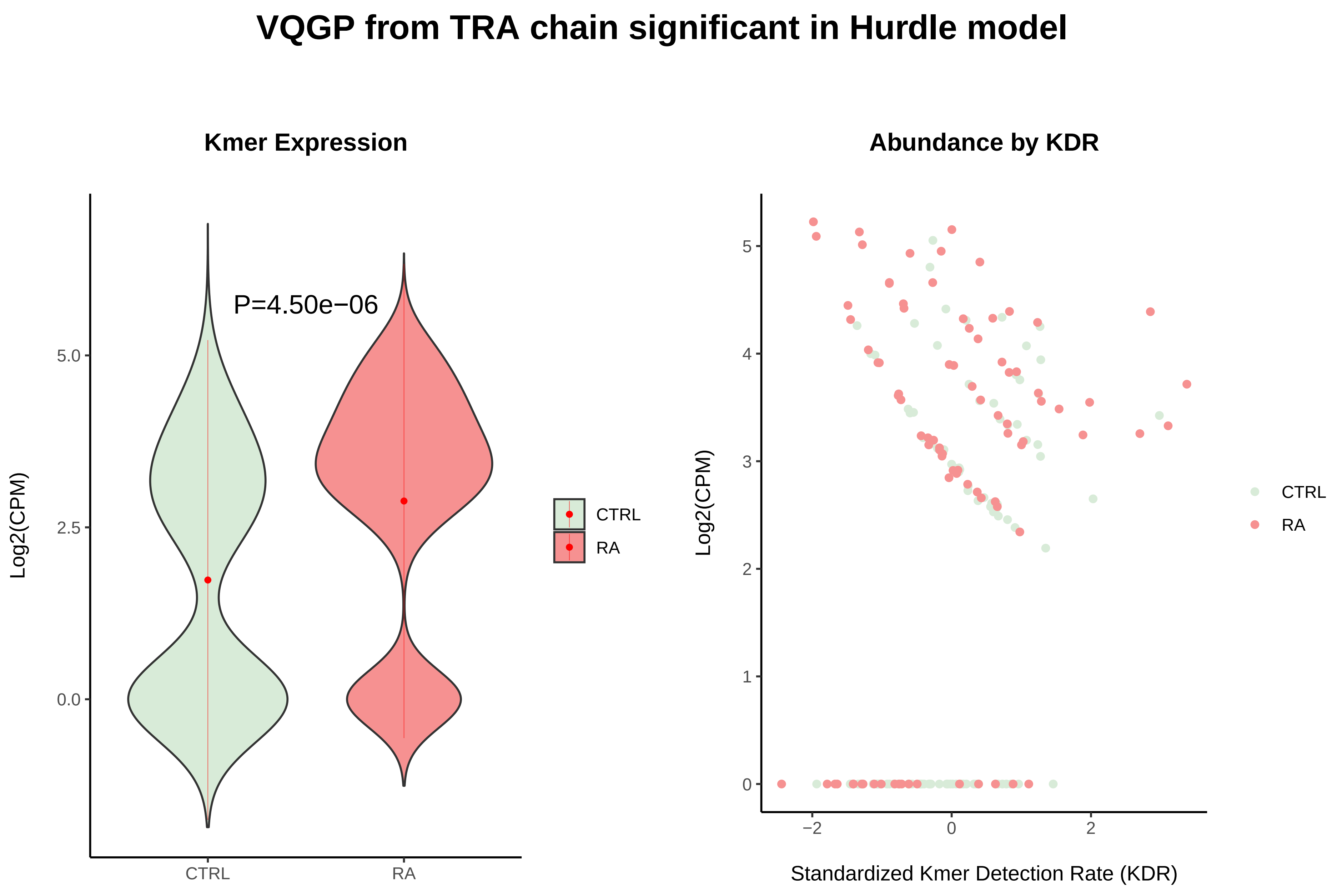

VQGP from TRA chain significant in Hurdle model
Kmer Expression
P=4.50e−06
Log2(CPM)
5.0
2.5
0.0
CTRL
RA
Abundance by KDR
Log2(CPM)
5
4
3
2
1
0
−2
0
2
Standardized Kmer Detection Rate (KDR)

VVNA from TRA chain significant in Hurdle model

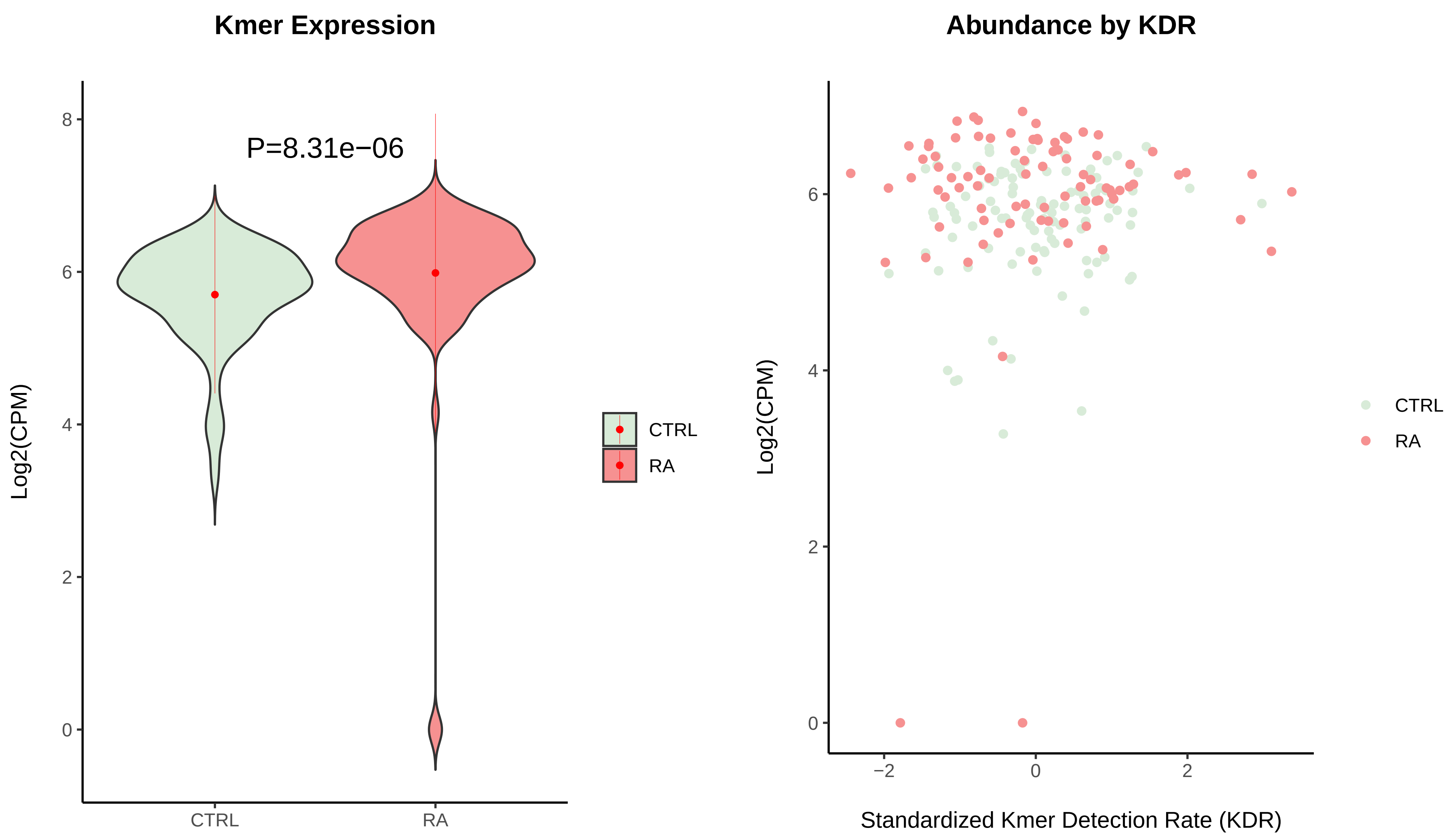

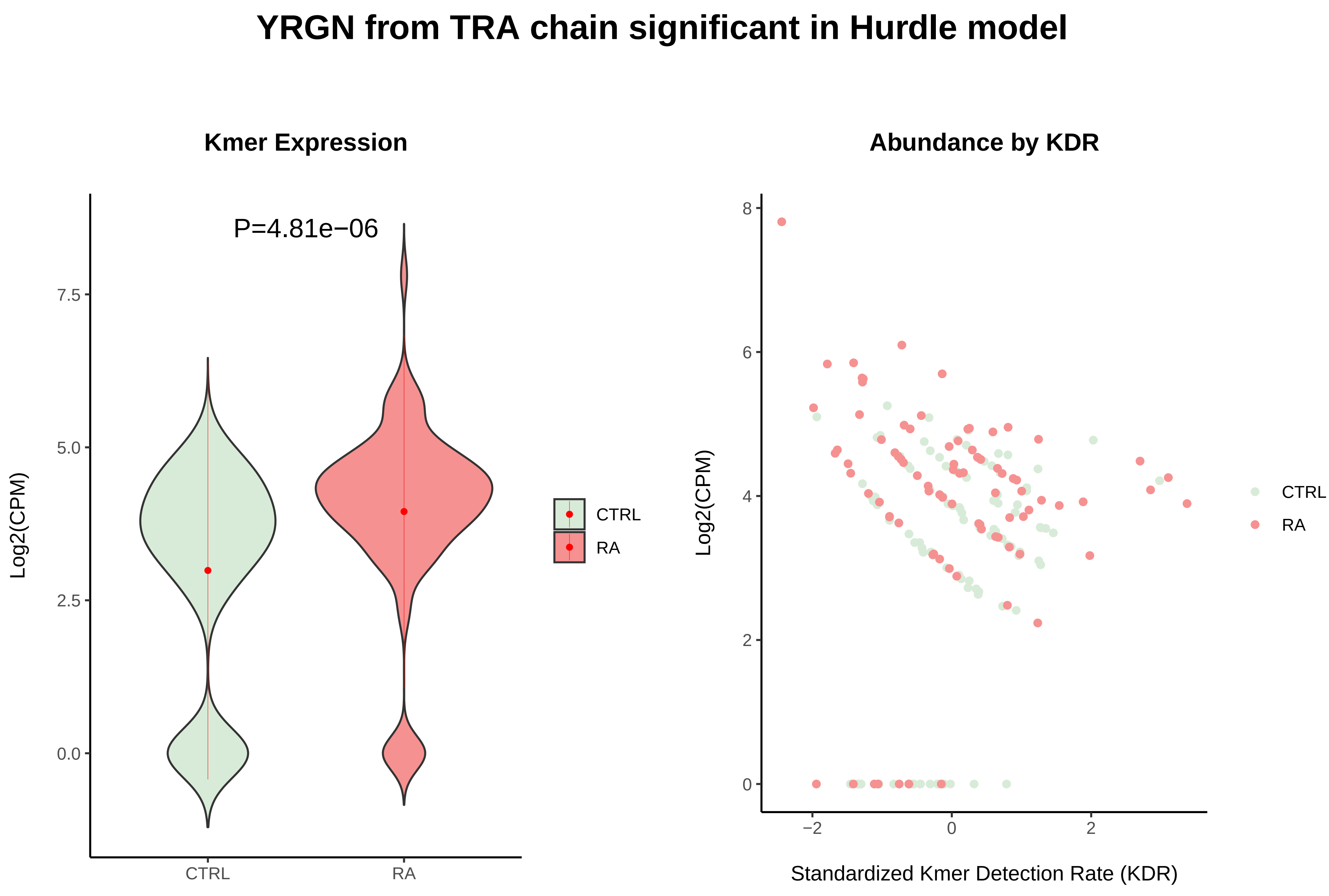
YRGN from TRA chain significant in Hurdle model
Kmer Expression
P=4.81e−06
Log2(CPM)
7.5
5.0
2.5
0.0
CTRL
RA
CTRL
RA
Abundance by KDR
Log2(CPM)
8
6
4
2
0
−2
0
2
Standardized Kmer Detection Rate (KDR)
CTRL
RA

AENG from TRA chain significant in Cont model

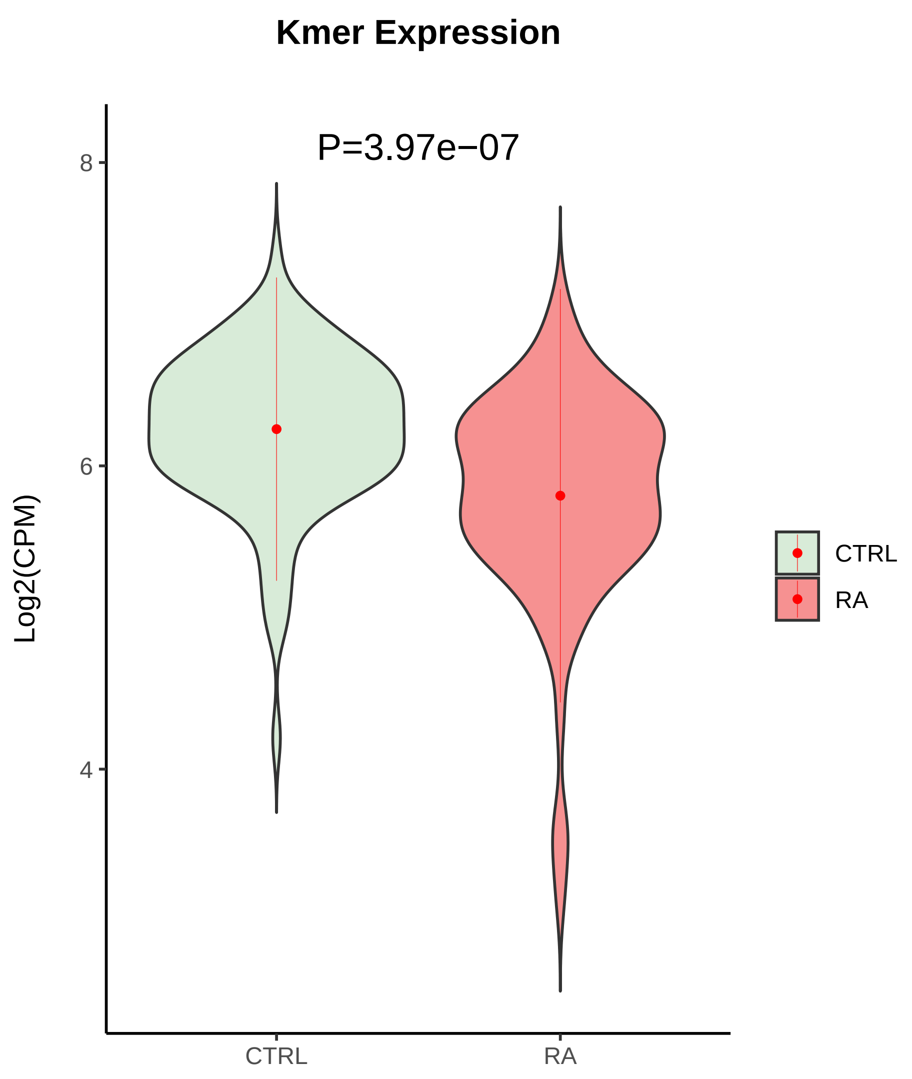

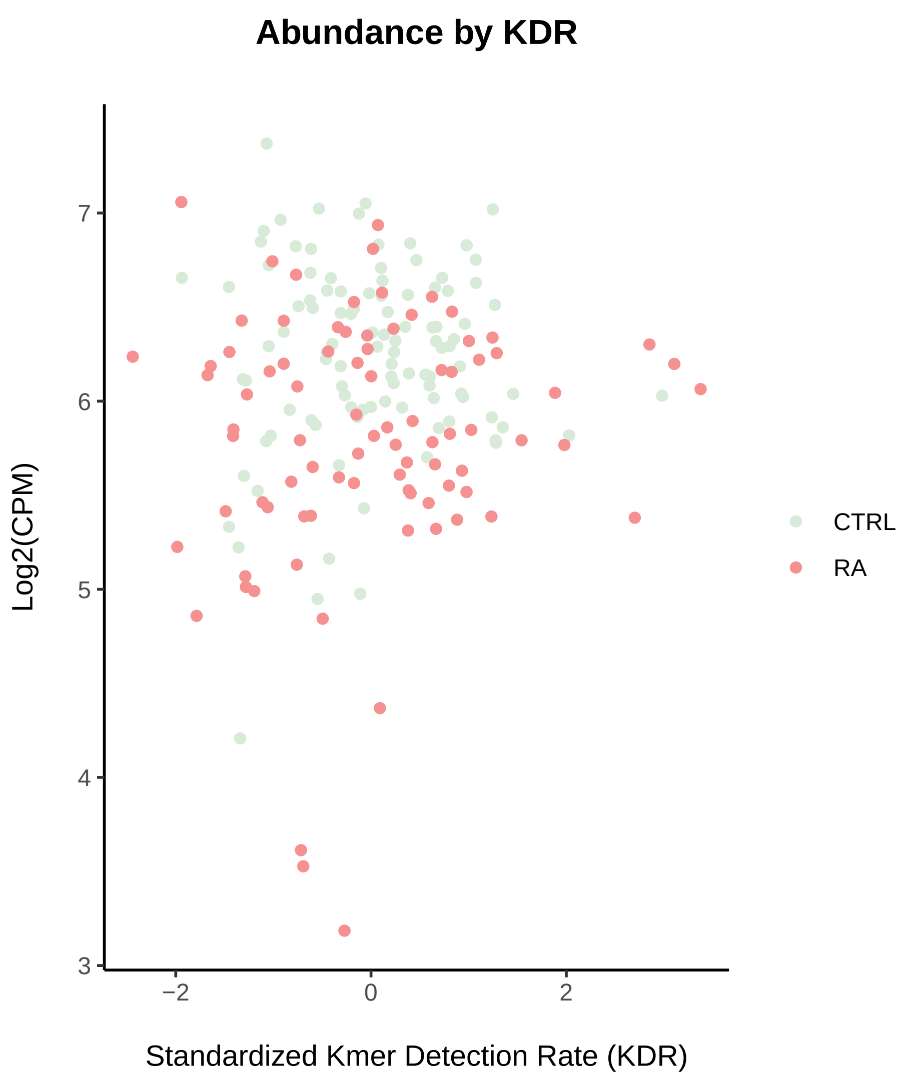

AENR from TRA chain significant in Cont model

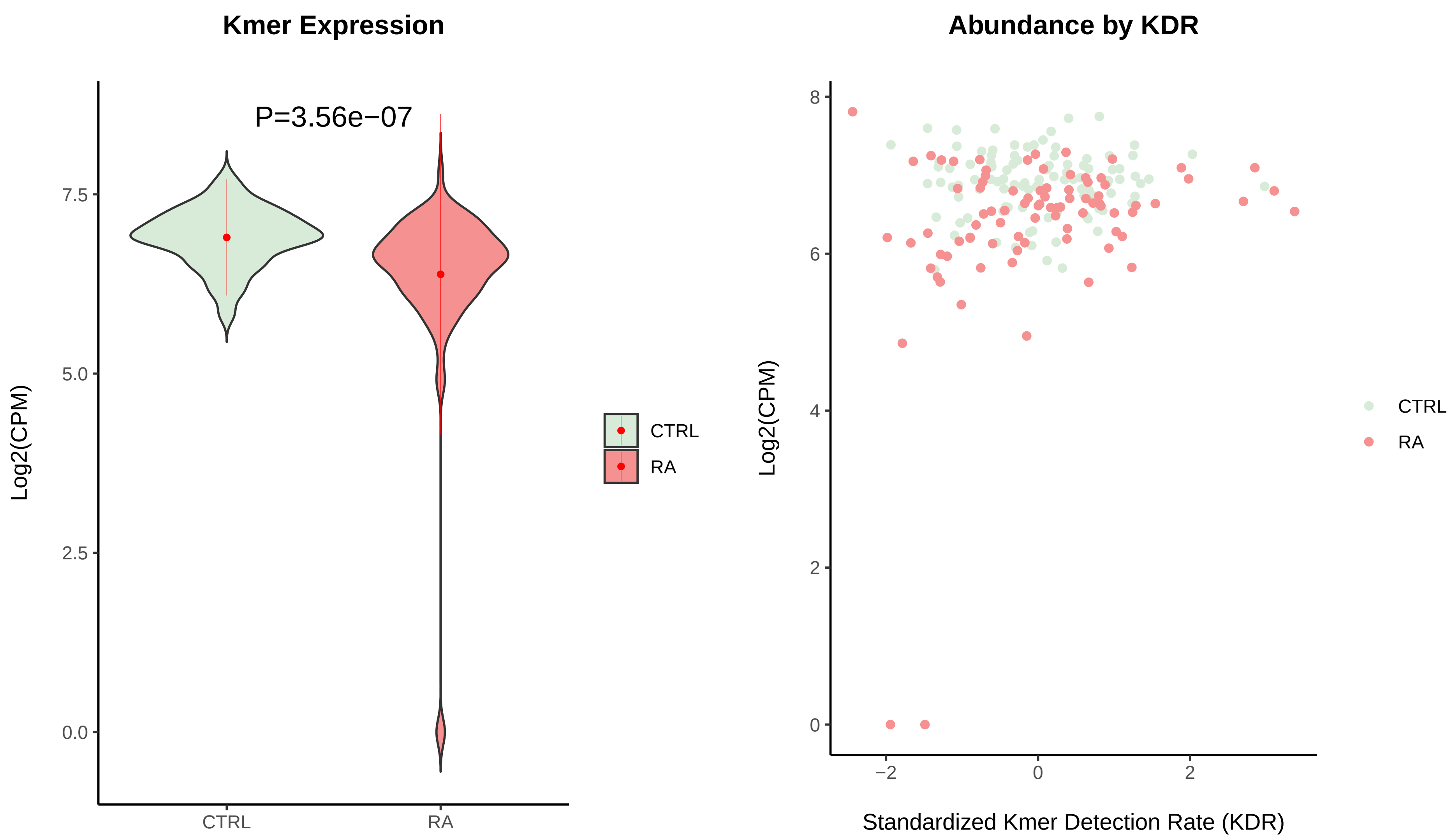

AVQA from TRA chain significant in Cont model

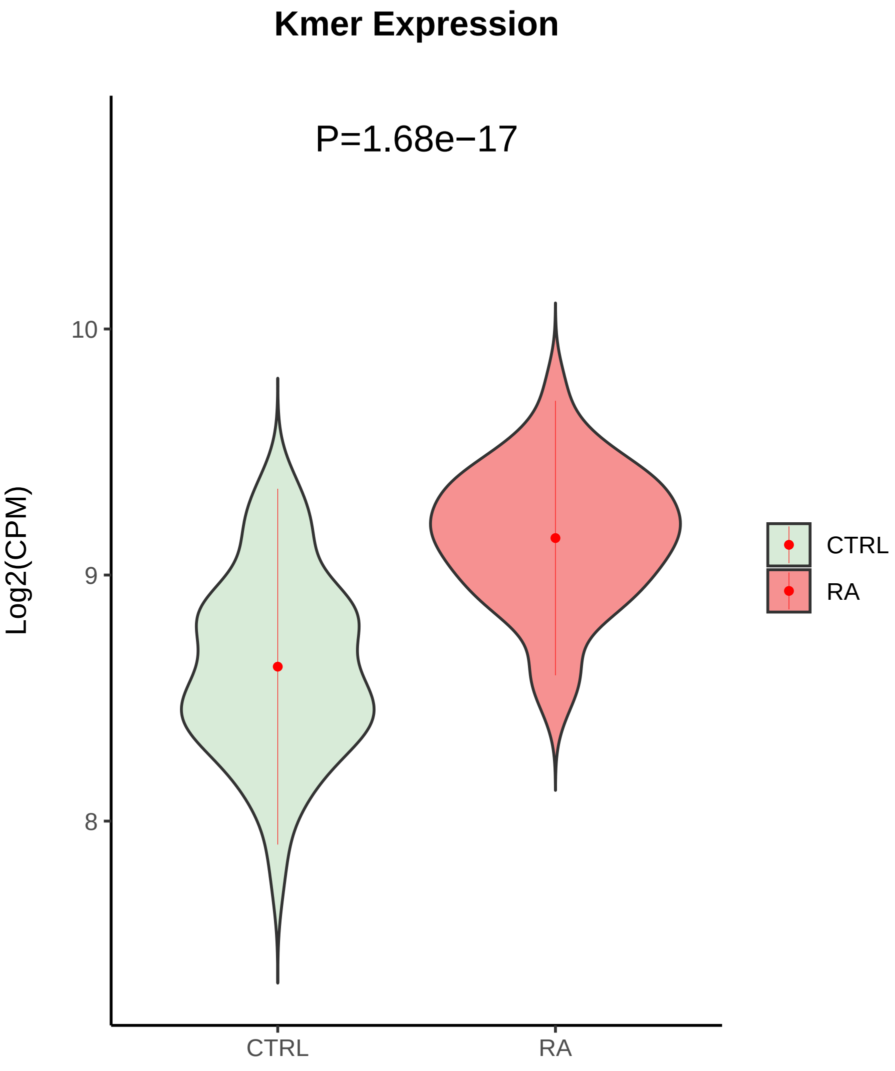

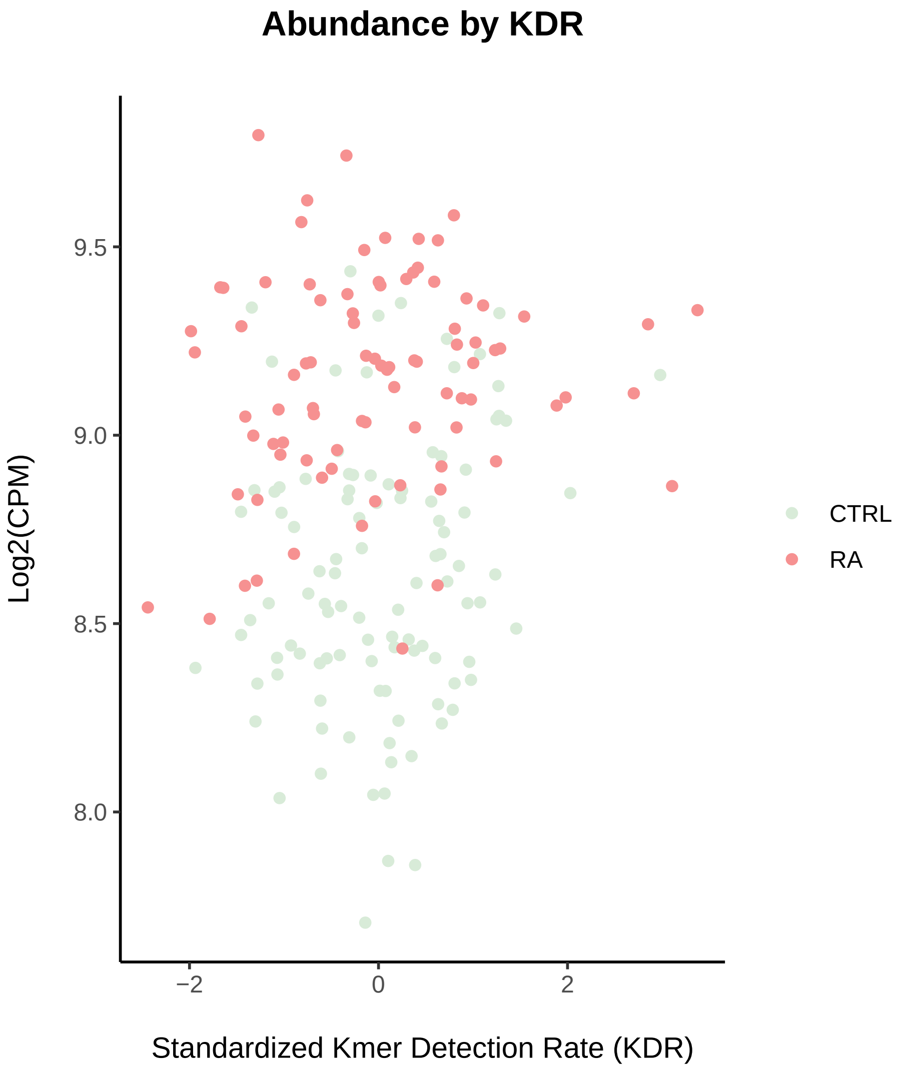

AVQD from TRA chain significant in Cont model

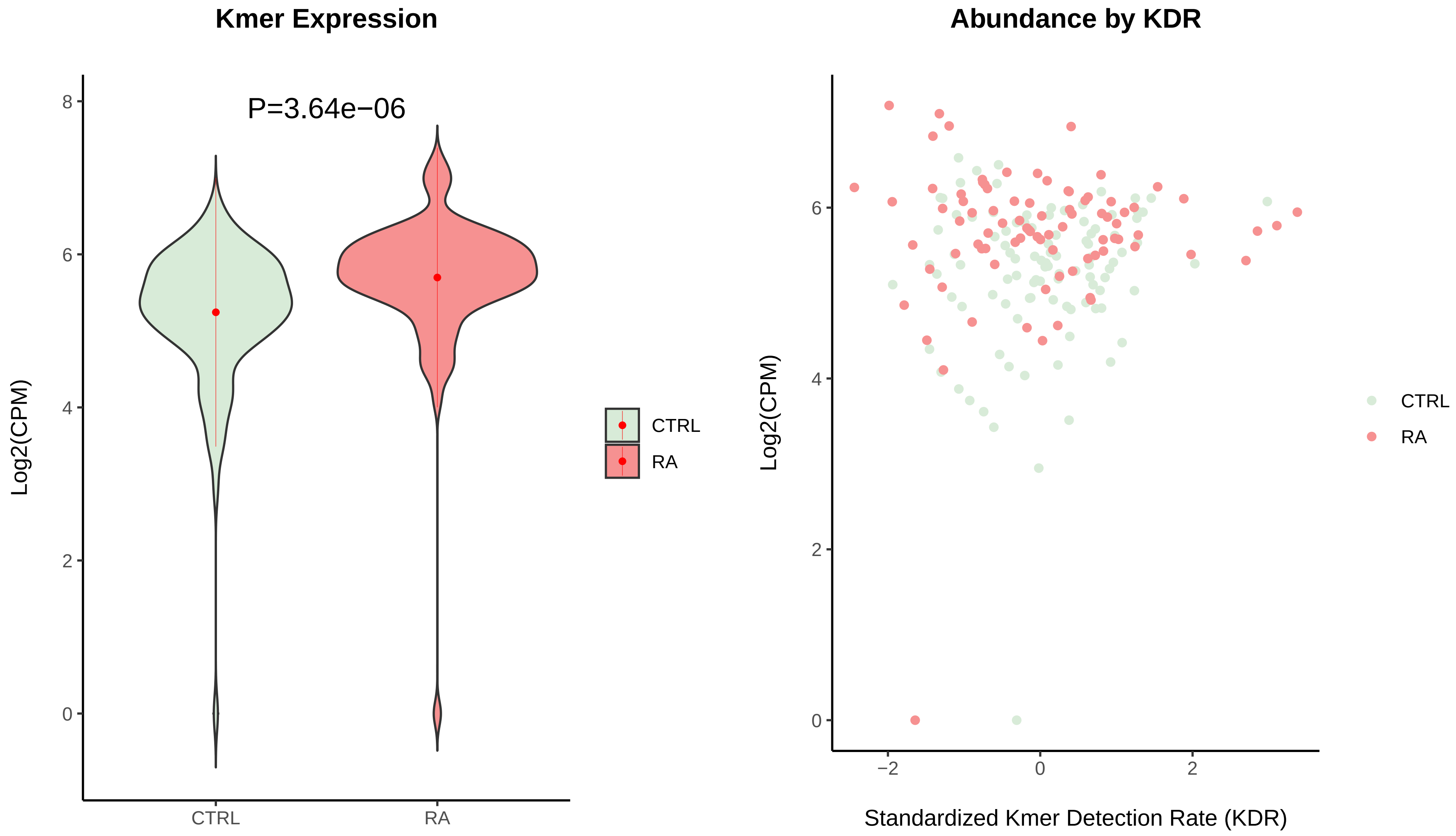

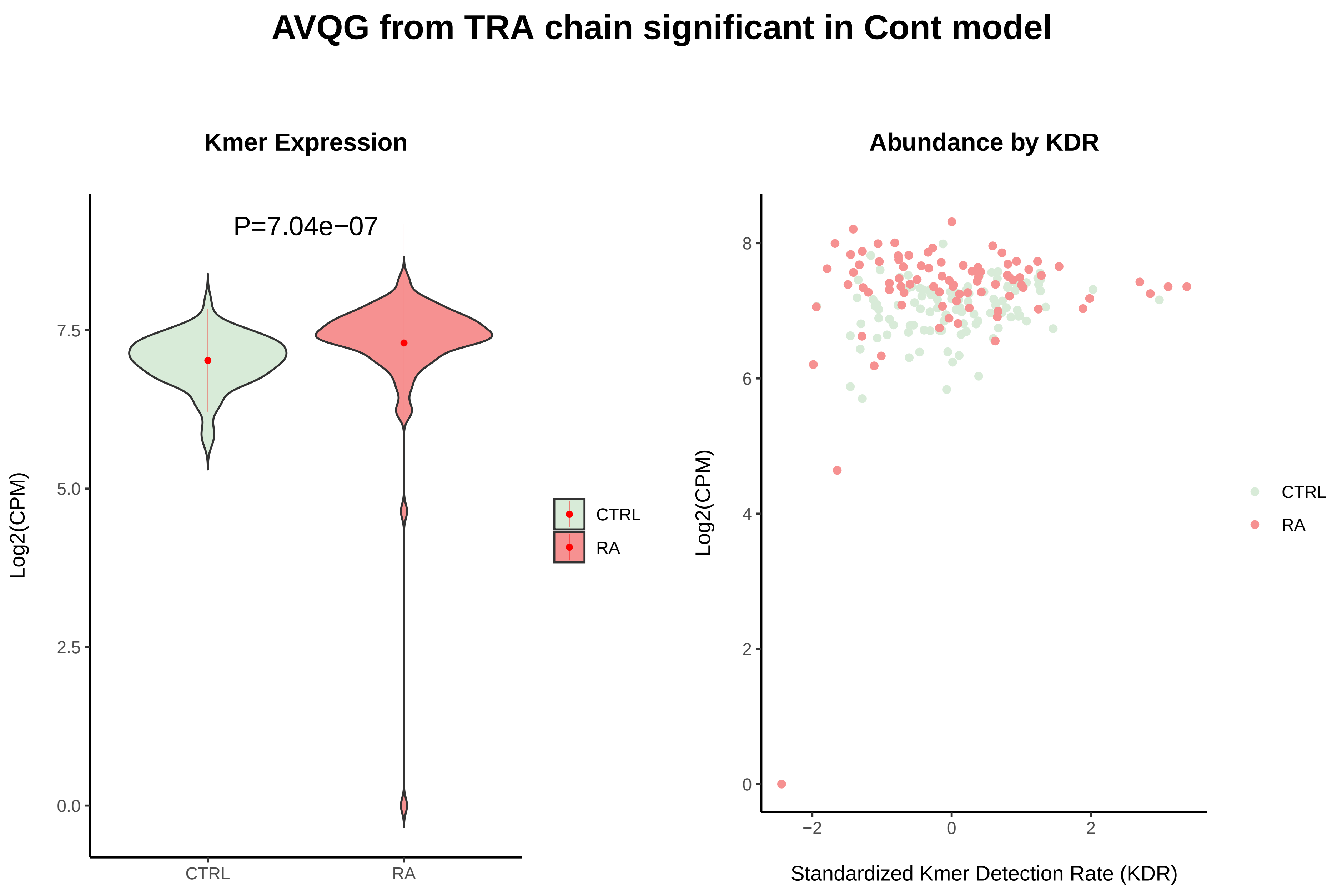
AVQG from TRA chain significant in Cont model
Kmer Expression
P=7.04e−07
Log2(CPM)
0.0
2.5
5.0
7.5
CTRL
RA
CTRL
RA
Abundance by KDR
Log2(CPM)
0
2
4
6
8
−2
0
2
Standardized Kmer Detection Rate (KDR)
CTRL
RA

AVQV from TRA chain significant in Cont model

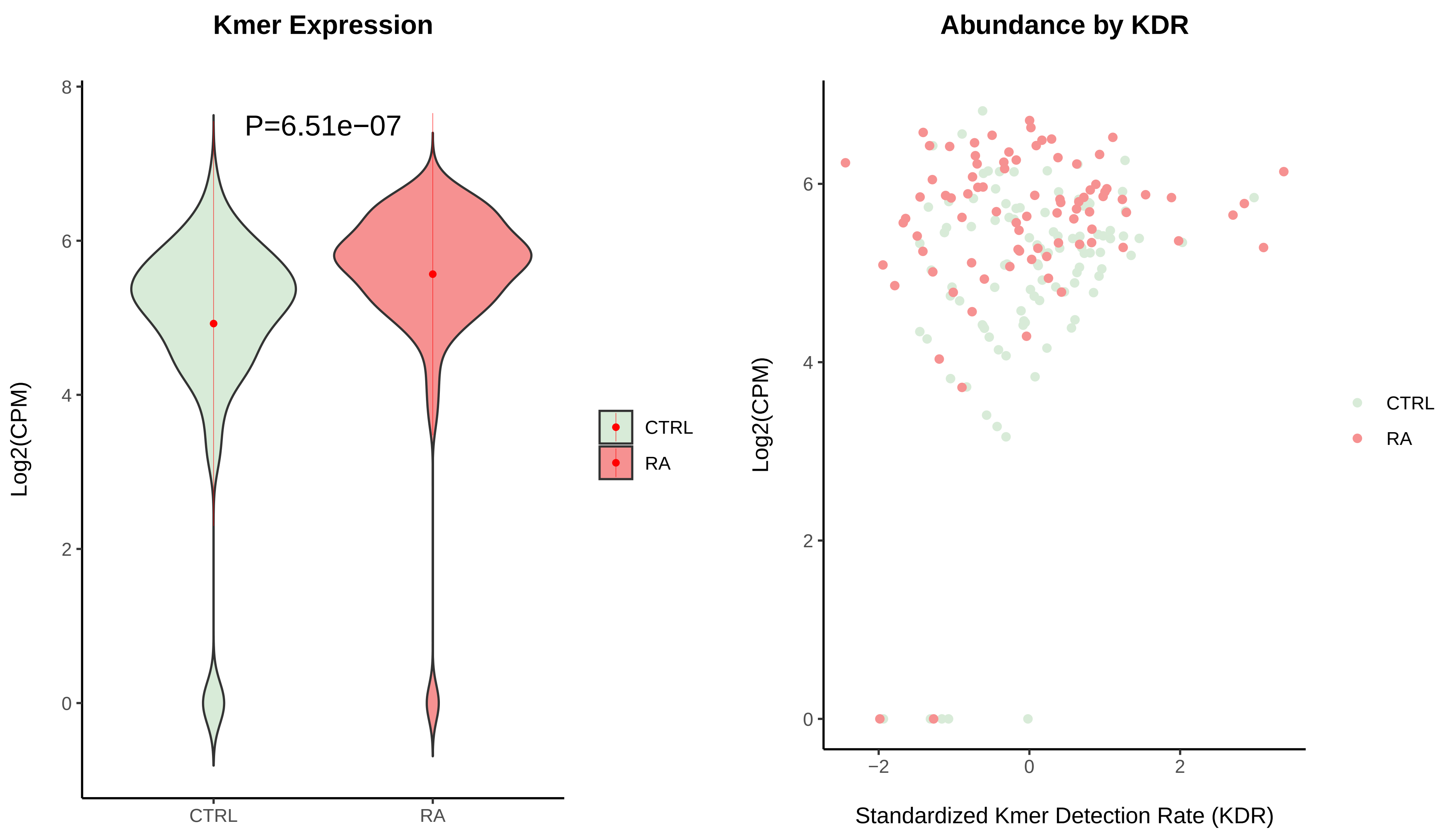

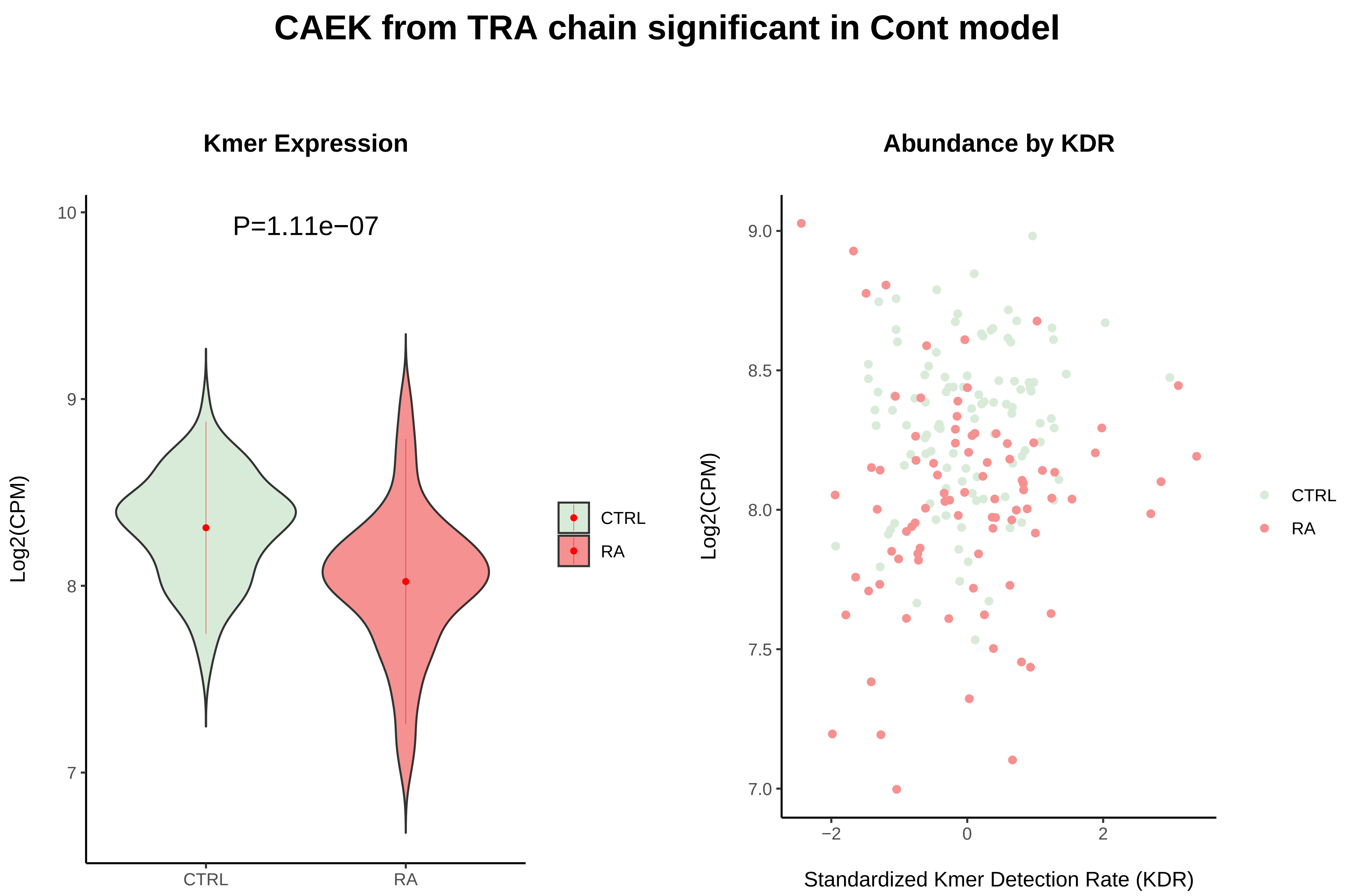
CAEK from TRA chain significant in Cont model
Kmer Expression
P=1.11e−07
Log2(CPM)
10
9
8
7
CTRL
RA
CTRL
RA
Abundance by KDR
Log2(CPM)
9.0
8.5
8.0
7.5
7.0
−2
0
2
Standardized Kmer Detection Rate (KDR)
CTRL
RA

CAEN from TRA chain significant in Cont model

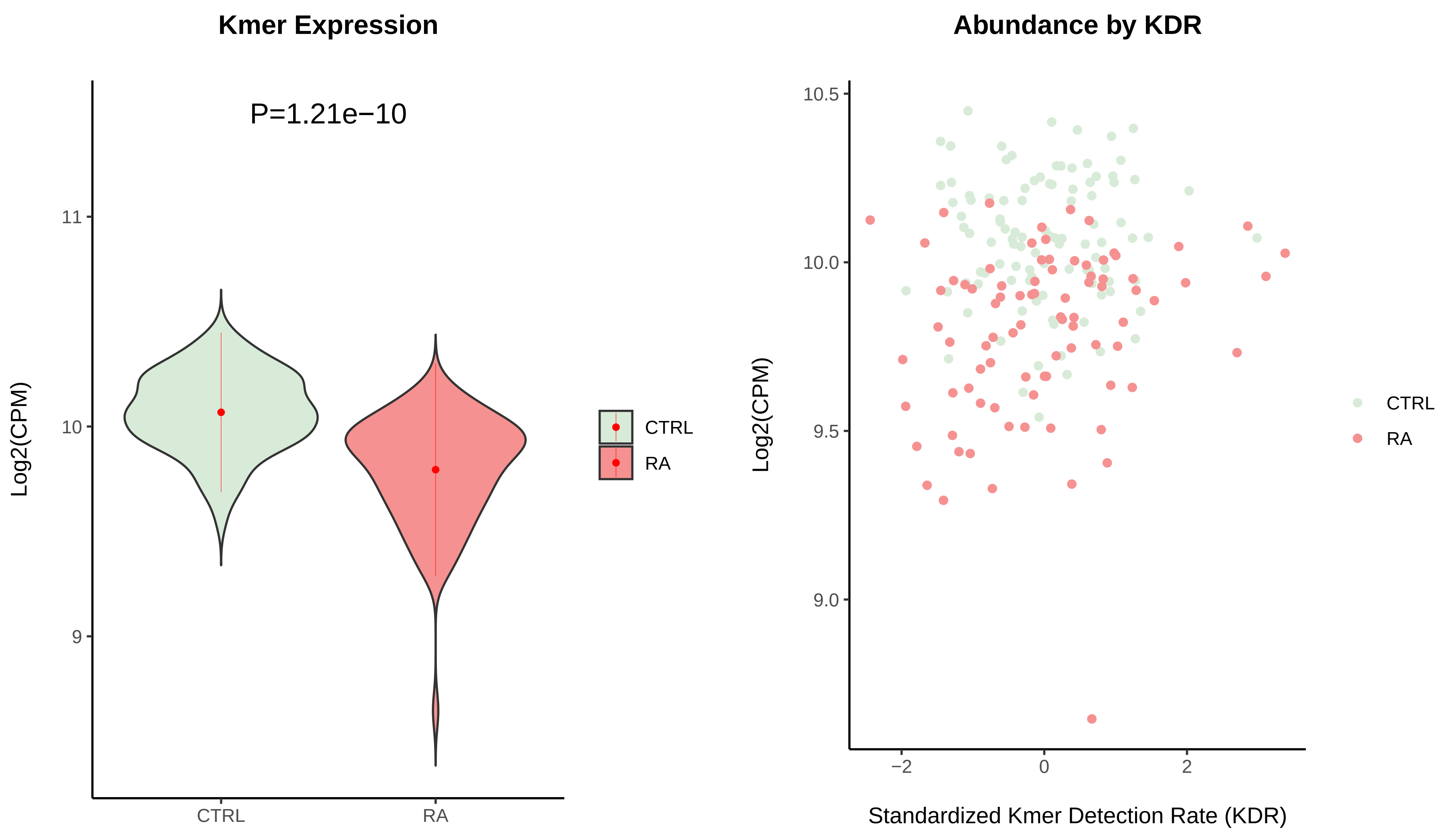

CAVQ from TRA chain significant in Cont model

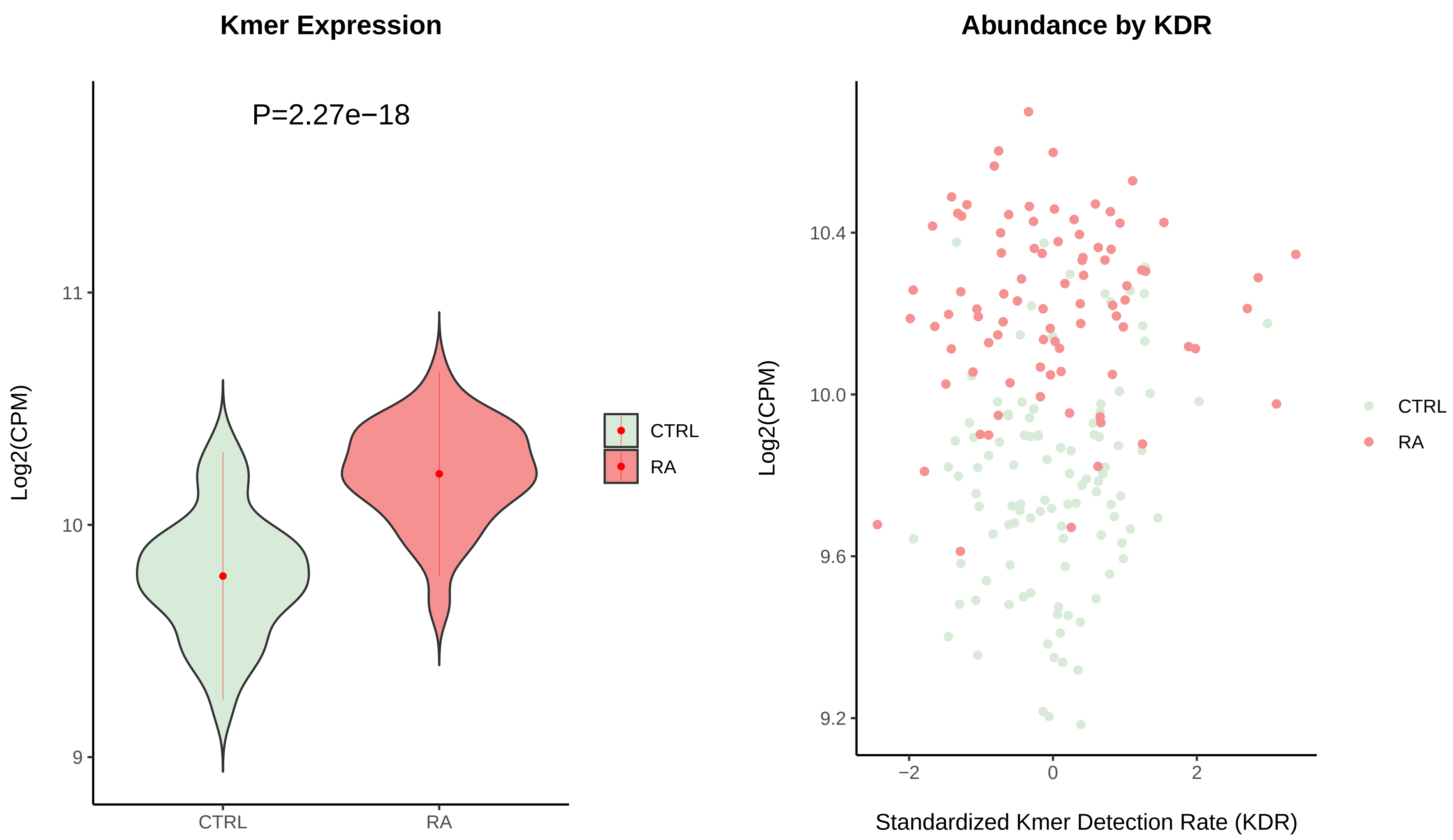

VQAA from TRA chain significant in Cont model

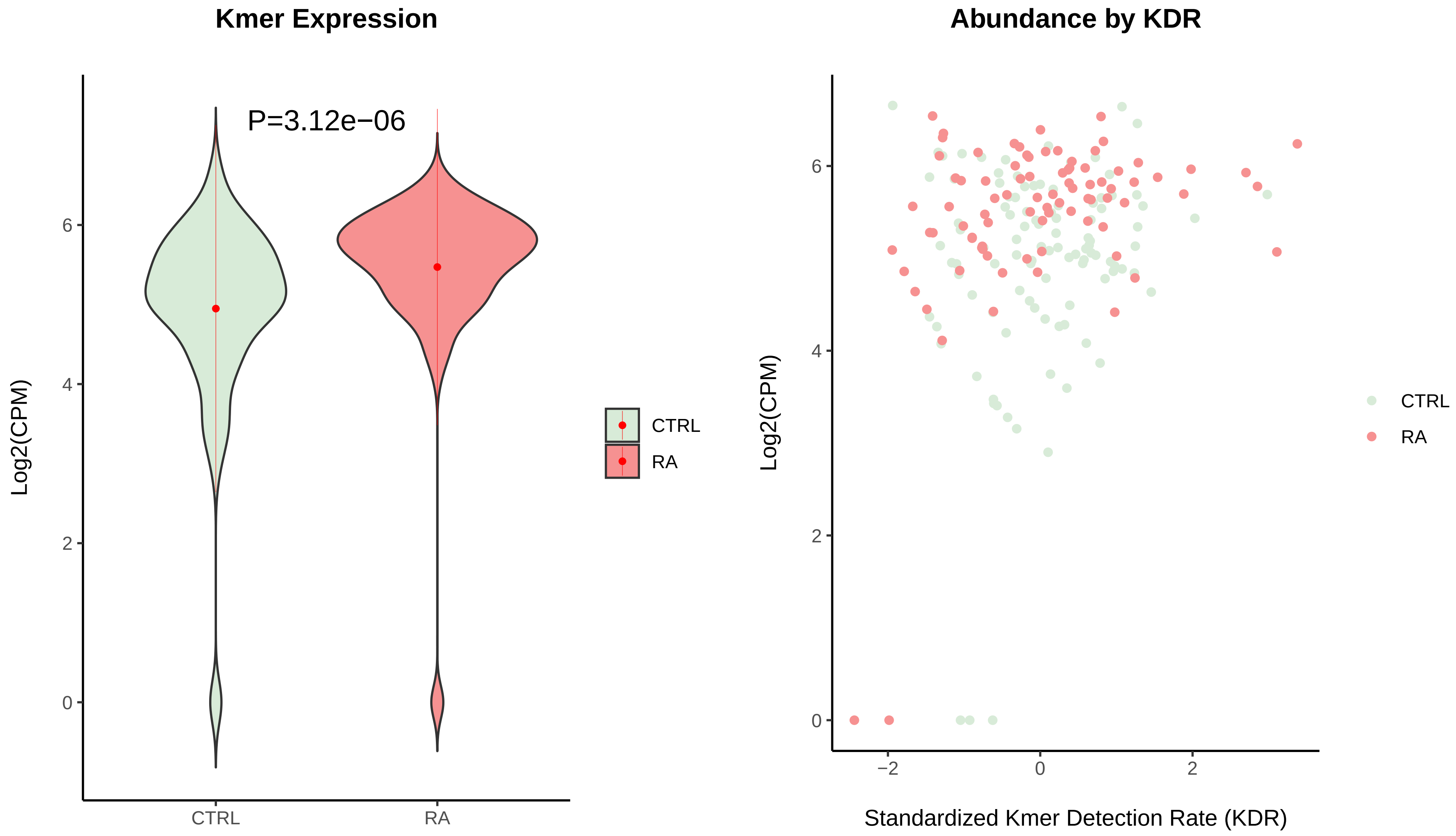

VQAG from TRA chain significant in Cont model

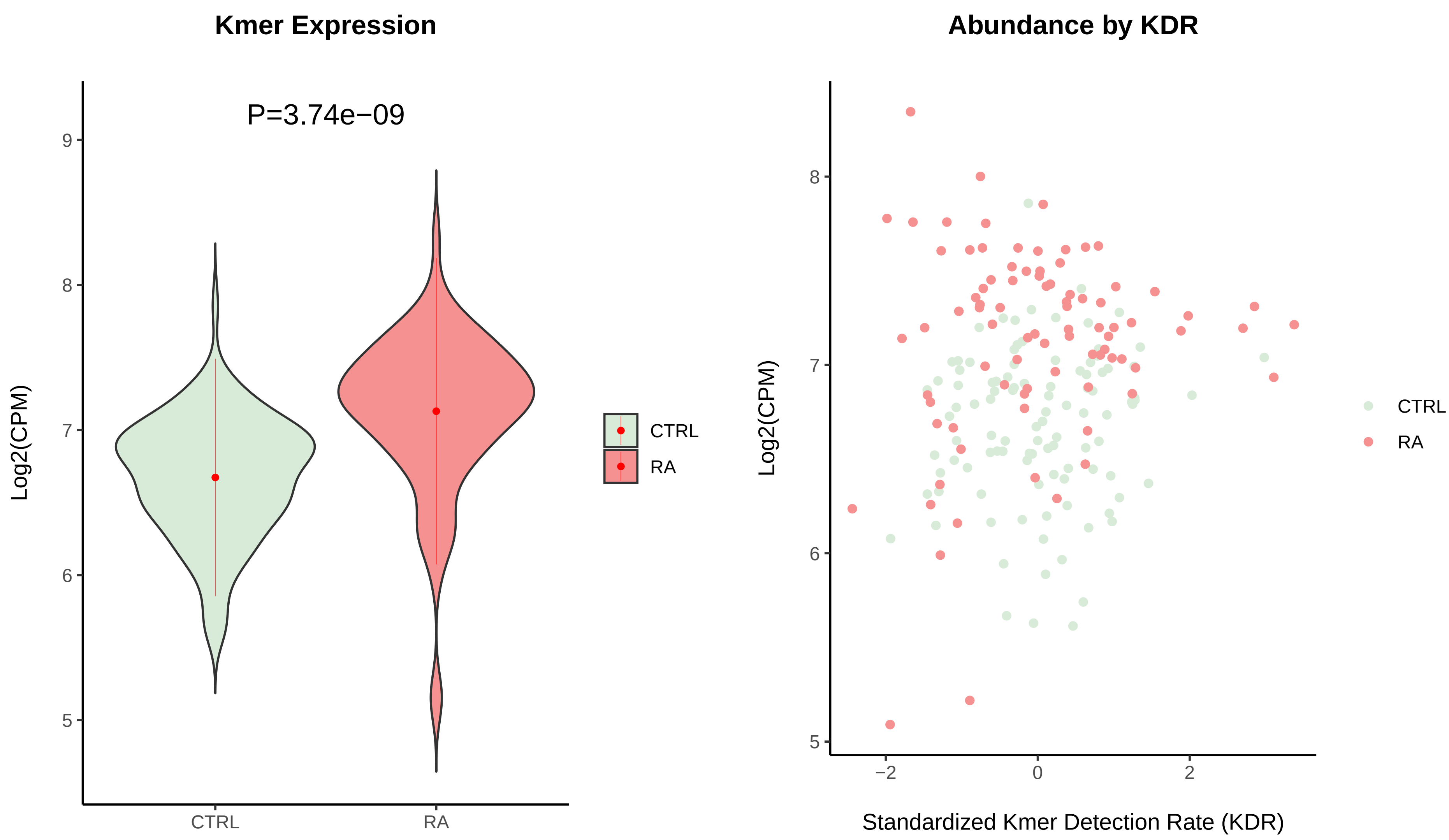

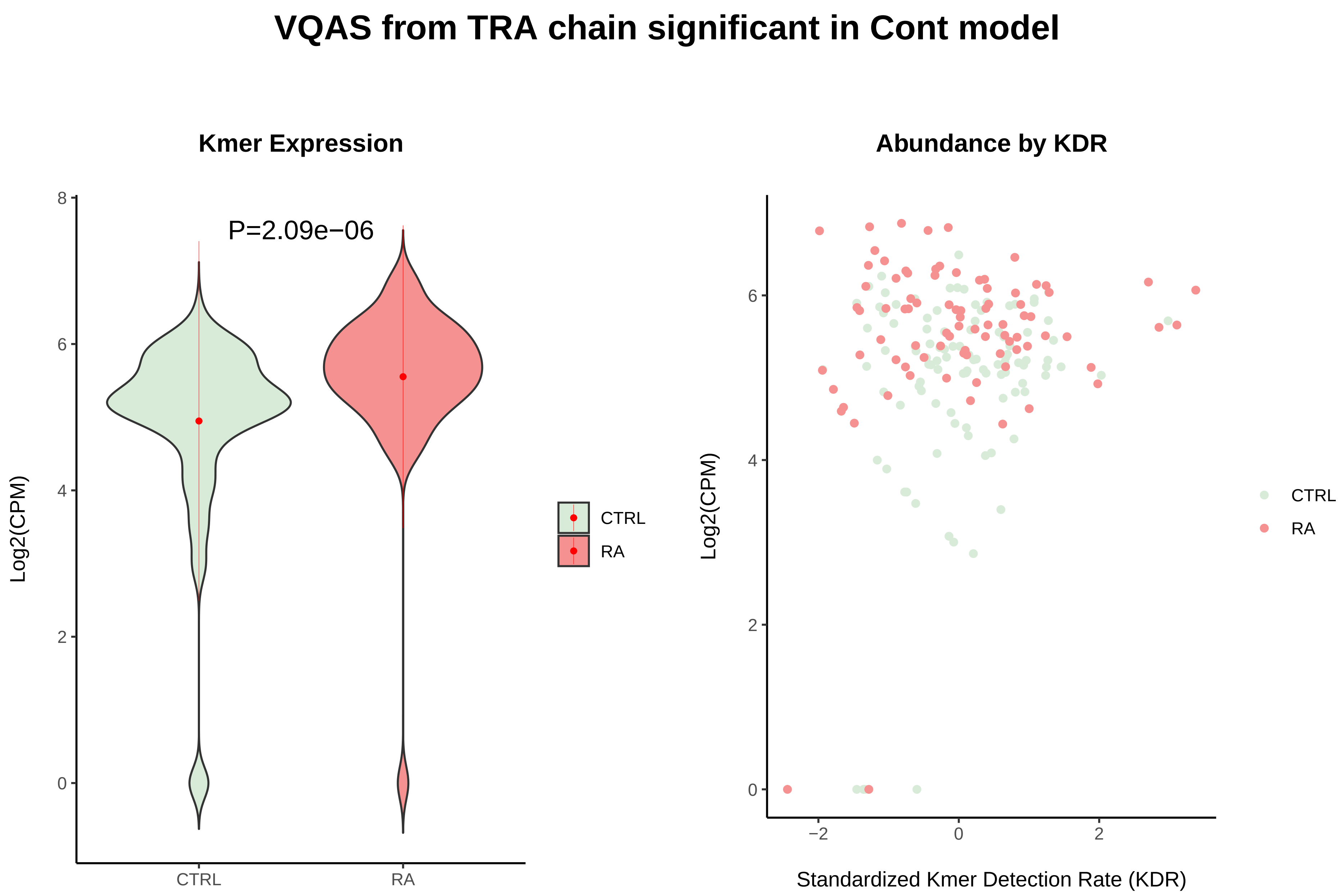
VQAS from TRA chain significant in Cont model
Kmer Expression
P=2.09e−06
Log2(CPM)
8
6
4
2
0
CTRL
RA
CTRL
RA
Abundance by KDR
Log2(CPM)
6
4
2
0
−2
0
2
Standardized Kmer Detection Rate (KDR)
CTRL
RA

VVNA from TRA chain significant in Cont model

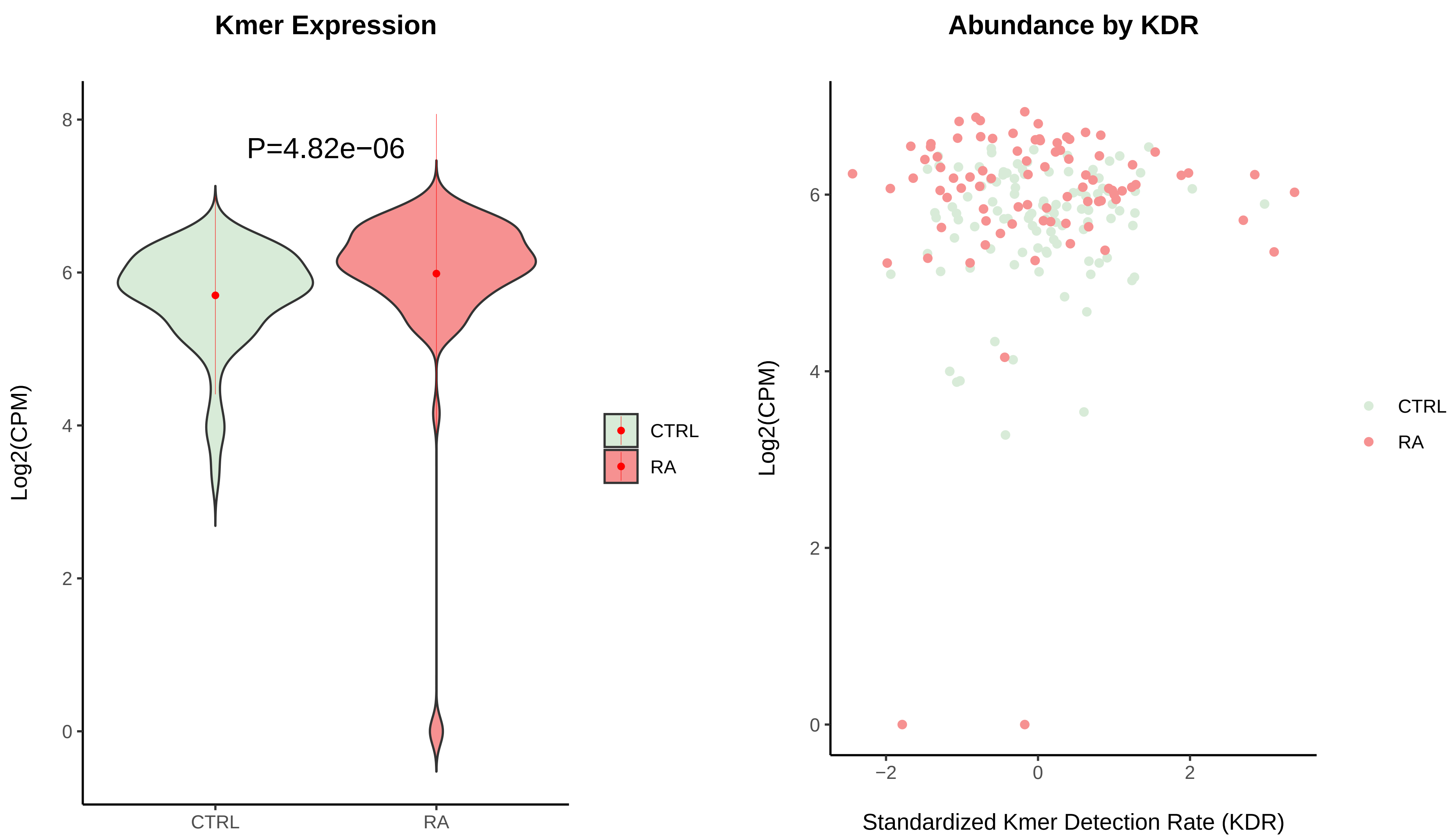

ELFF from TRB chain significant in Cont model

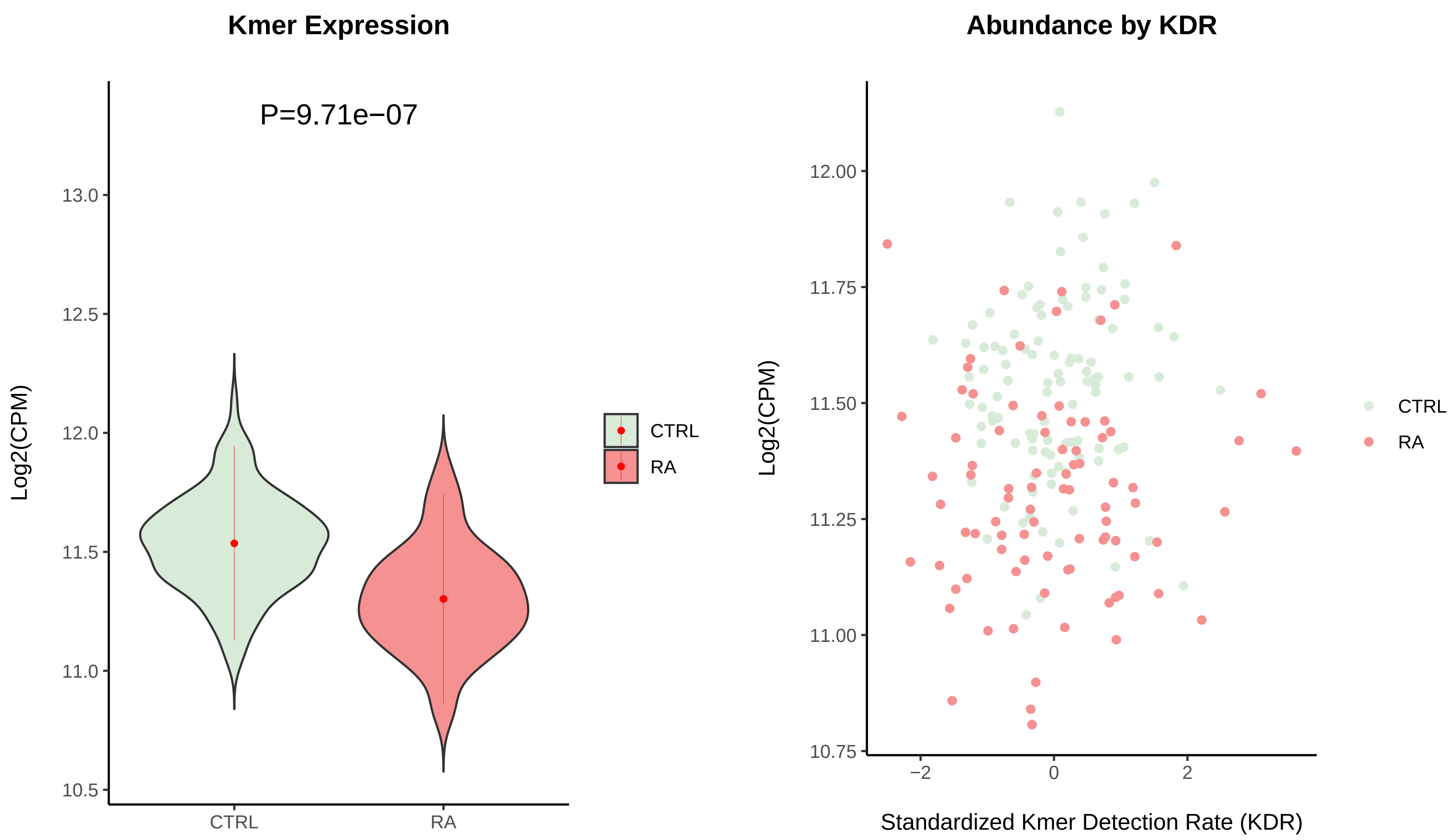

GELF from TRB chain significant in Cont model

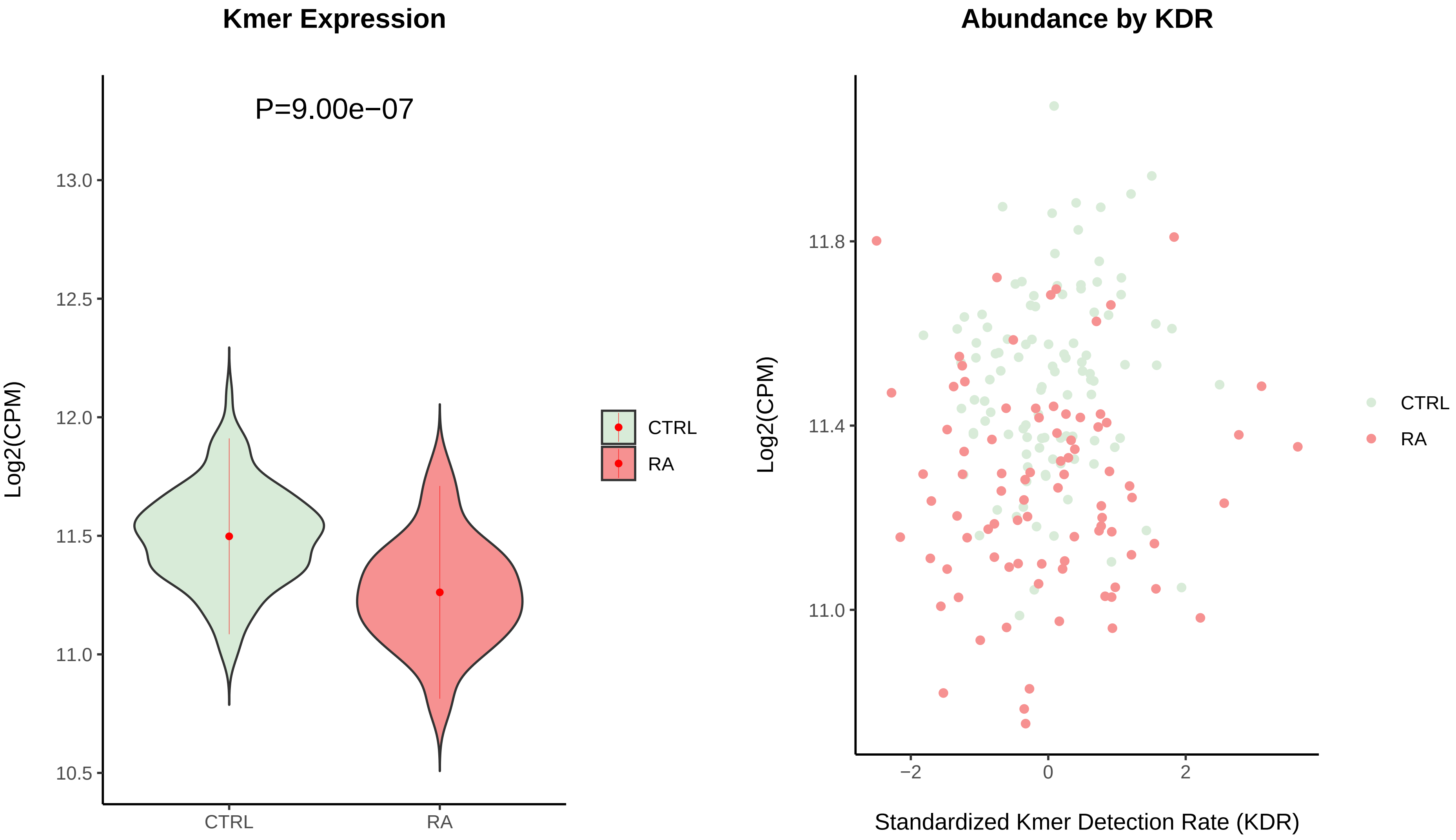

GETQ from TRB chain significant in Cont model

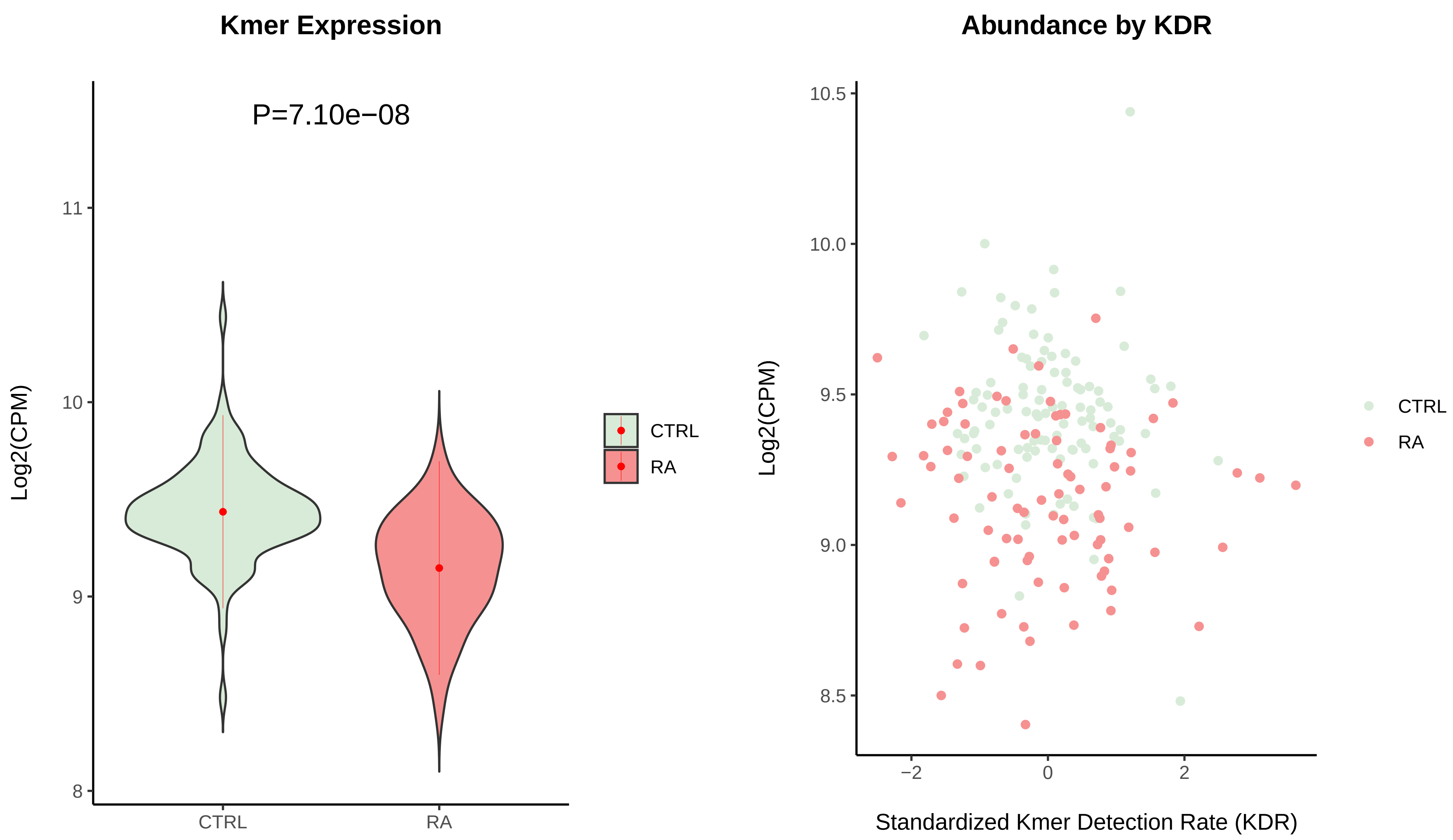

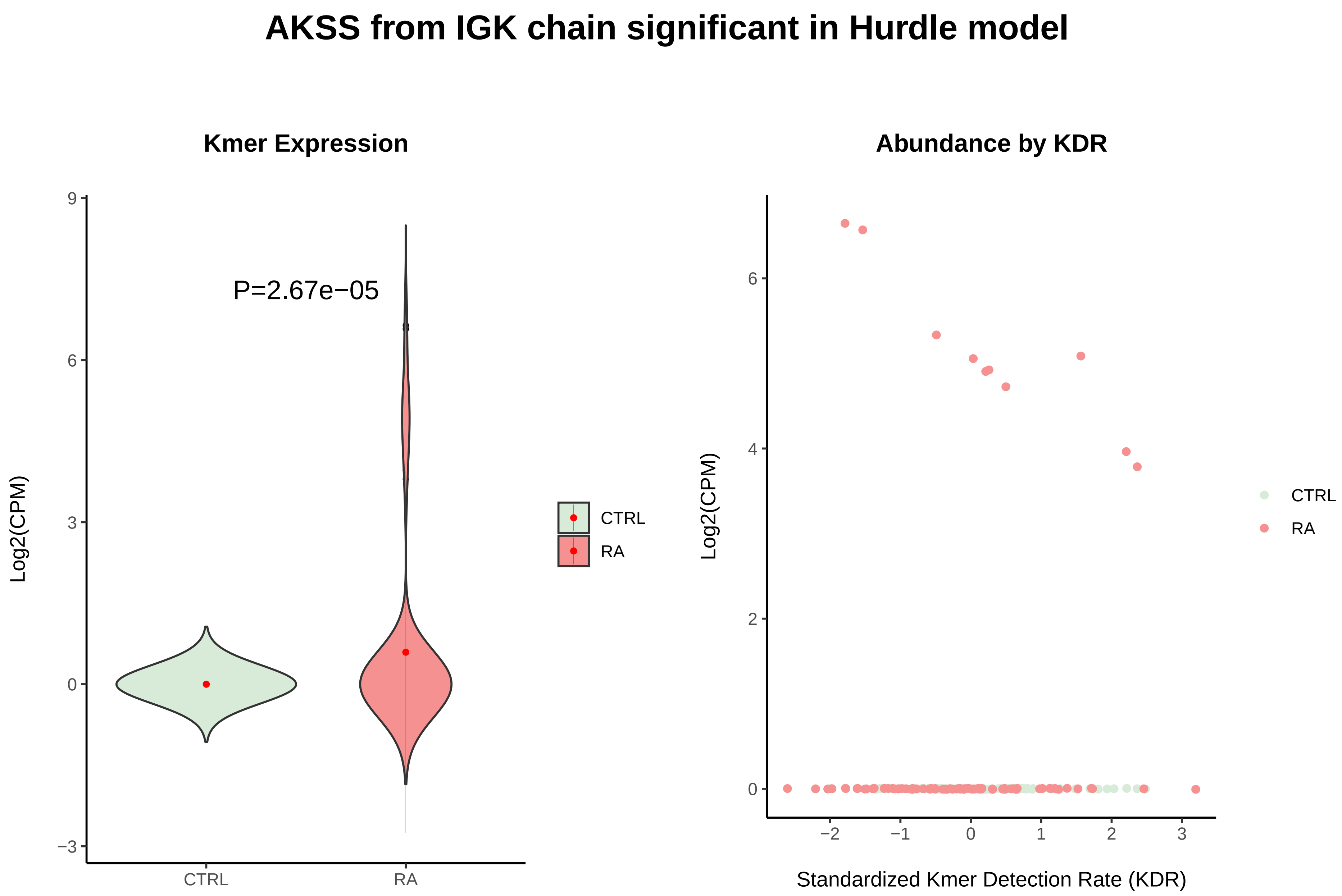

AKSS from IGK chain significant in Hurdle model
Kmer Expression
P=2.67e−05
Log2(CPM)
9
6
3
0
−3
CTRL
RA
CTRL
RA
Abundance by KDR
Log2(CPM)
6
4
2
0
−2
−1
0
1
2
3
Standardized Kmer Detection Rate (KDR)
CTRL
RA

ALQA from IGK chain significant in Hurdle model

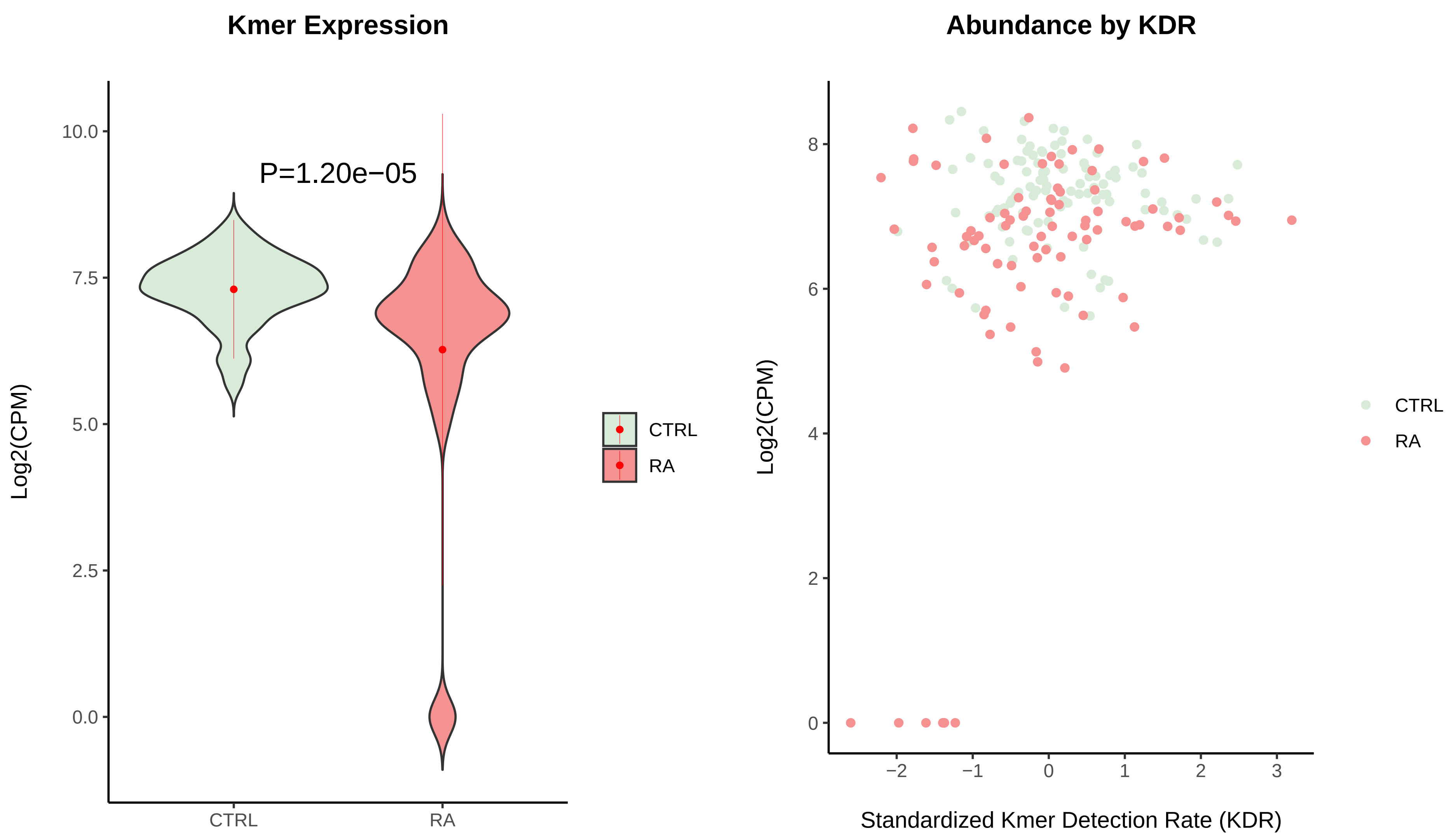

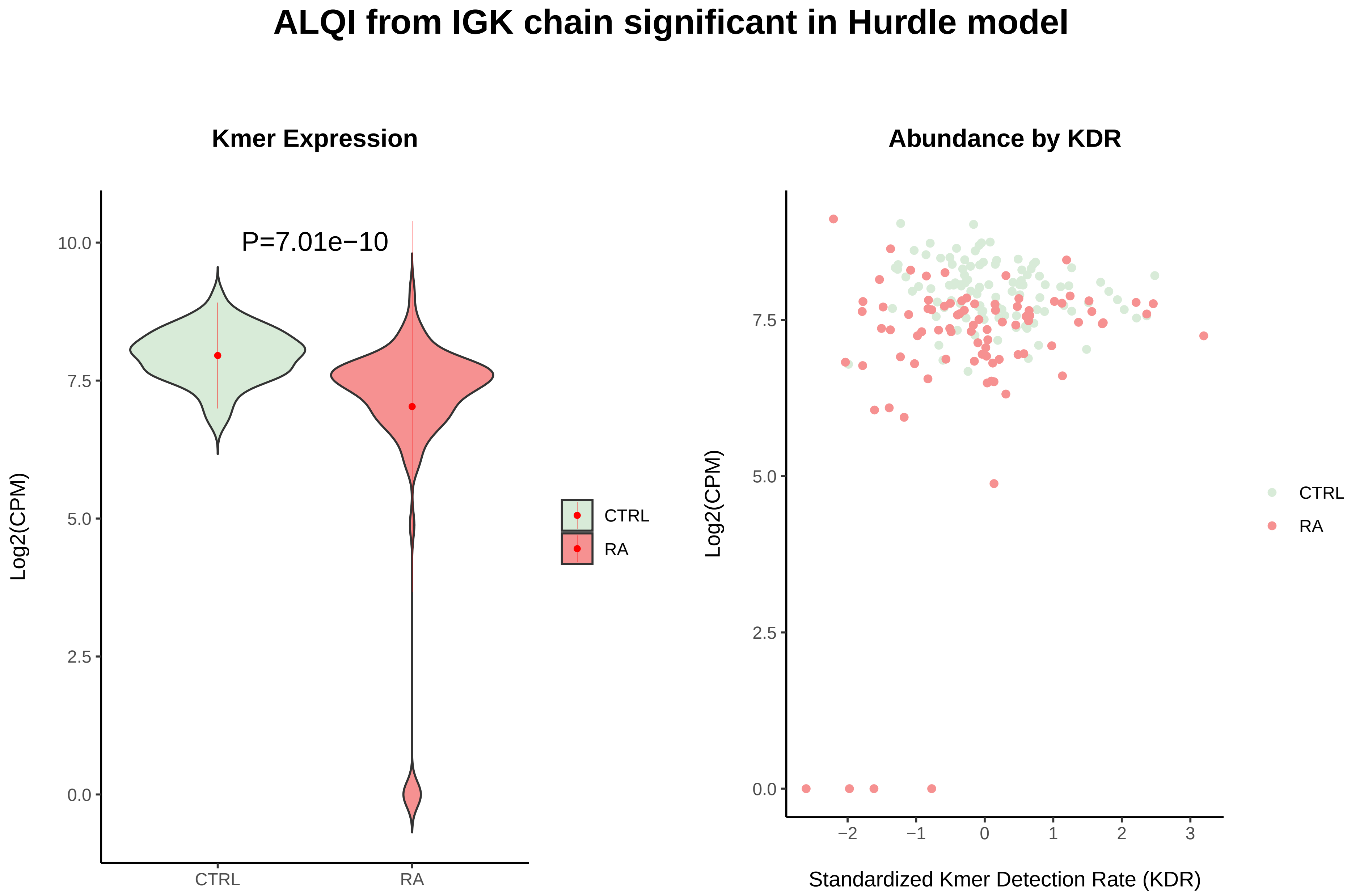
ALQI from IGK chain significant in Hurdle model
Kmer Expression
P=7.01e−10
Log2(CPM)
10.0
7.5
5.0
2.5
0.0
CTRL
RA
CTRL
RA
Abundance by KDR
Log2(CPM)
7.5
5.0
2.5
0.0
−2
−1
0
1
2
3
Standardized Kmer Detection Rate (KDR)
CTRL
RA

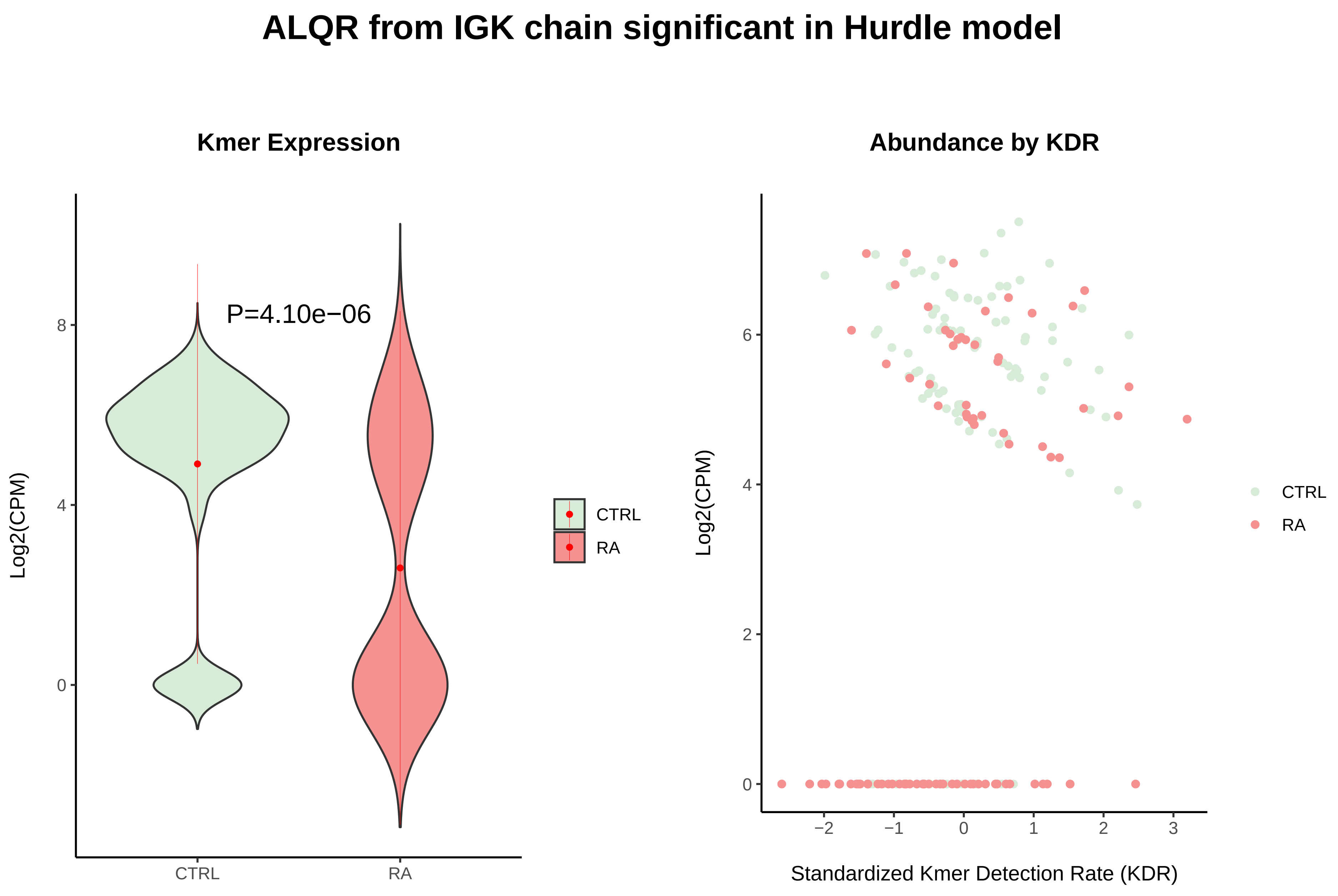
ALQR from IGK chain significant in Hurdle model
Kmer Expression
P=4.10e−06
Log2(CPM)
8
4
0
CTRL
RA
Abundance by KDR
Log2(CPM)
6
4
2
0
−2
−1
0
1
2
3
Standardized Kmer Detection Rate (KDR)
CTRL
RA

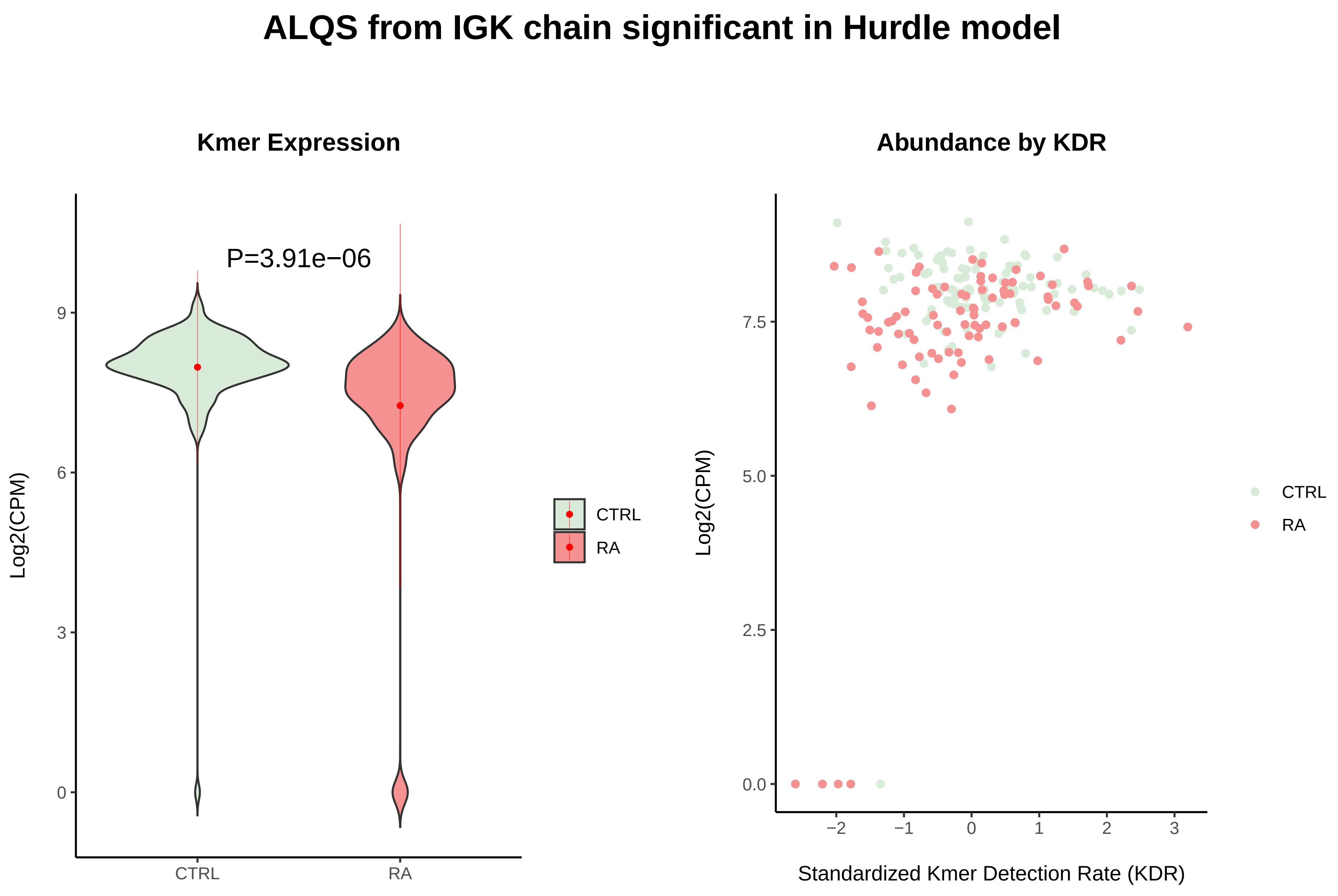
ALQS from IGK chain significant in Hurdle model
Kmer Expression
P=3.91e−06
Log2(CPM)
9
6
3
0
CTRL
RA
CTRL
RA
Abundance by KDR
Log2(CPM)
7.5
5.0
2.5
0.0
−2
−1
0
1
2
3
Standardized Kmer Detection Rate (KDR)
CTRL
RA

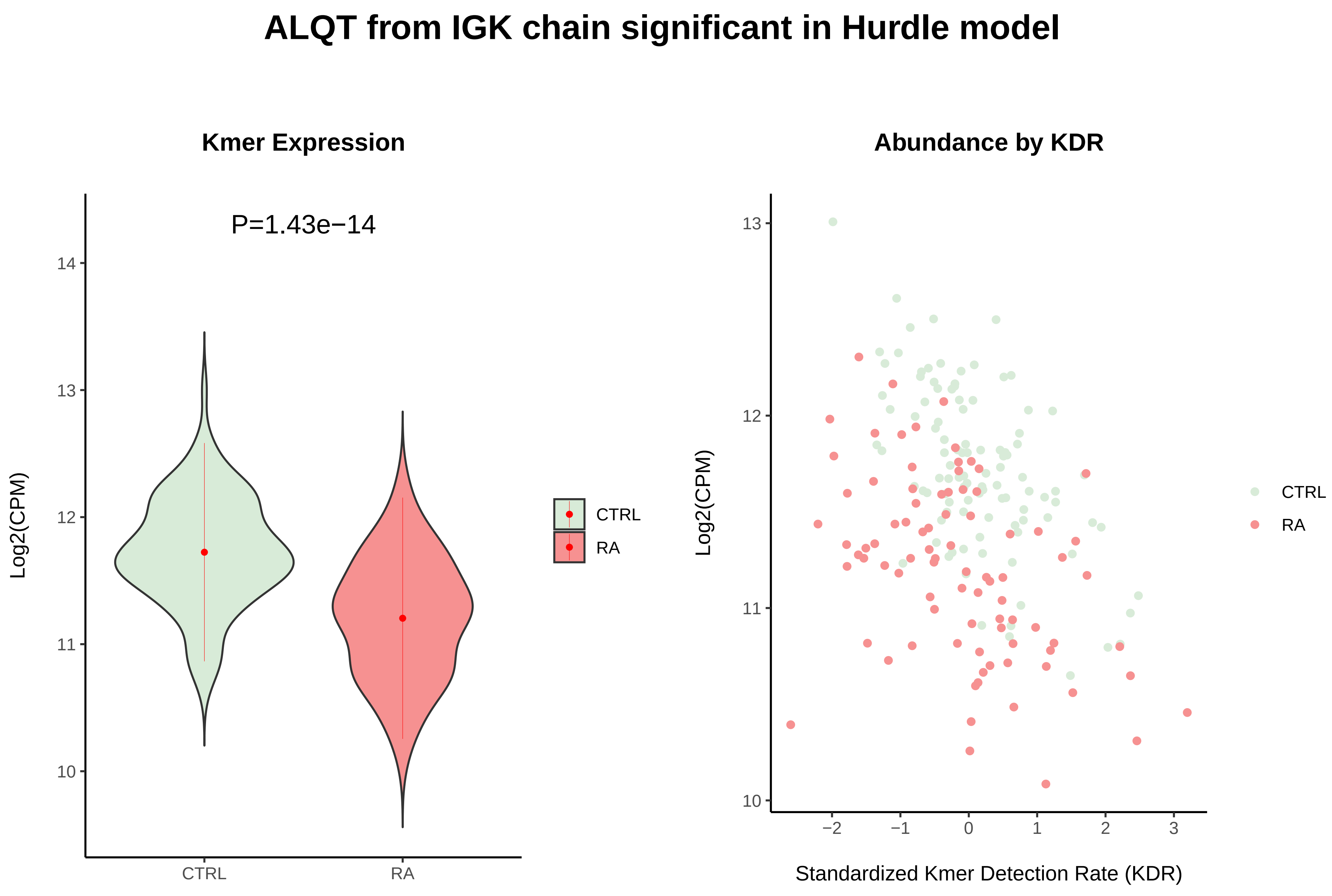
ALQT from IGK chain significant in Hurdle model
Kmer Expression
P=1.43e−14
Log2(CPM)
14
13
12
11
10
CTRL
RA
Abundance by KDR
Log2(CPM)
13
12
11
10
−2
−1
0
1
2
3
Standardized Kmer Detection Rate (KDR)
CTRL
RA

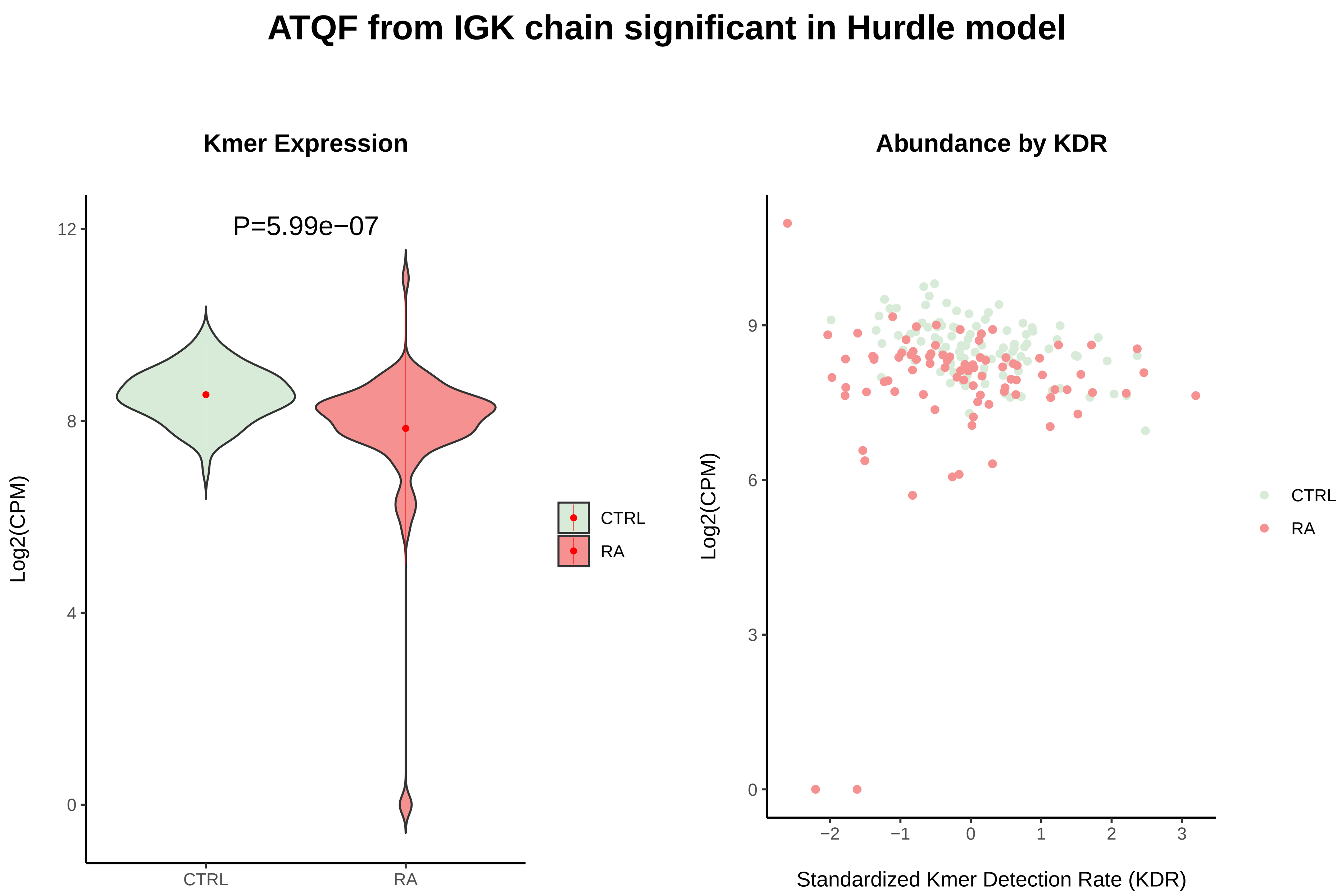
ATQF from IGK chain significant in Hurdle model
Kmer Expression
P=5.99e−07
Log2(CPM)
12
8
4
0
CTRL
RA
CTRL
RA
Abundance by KDR
Log2(CPM)
9
6
3
0
−2
−1
0
1
2
3
Standardized Kmer Detection Rate (KDR)
CTRL
RA

CMQA from IGK chain significant in Hurdle model

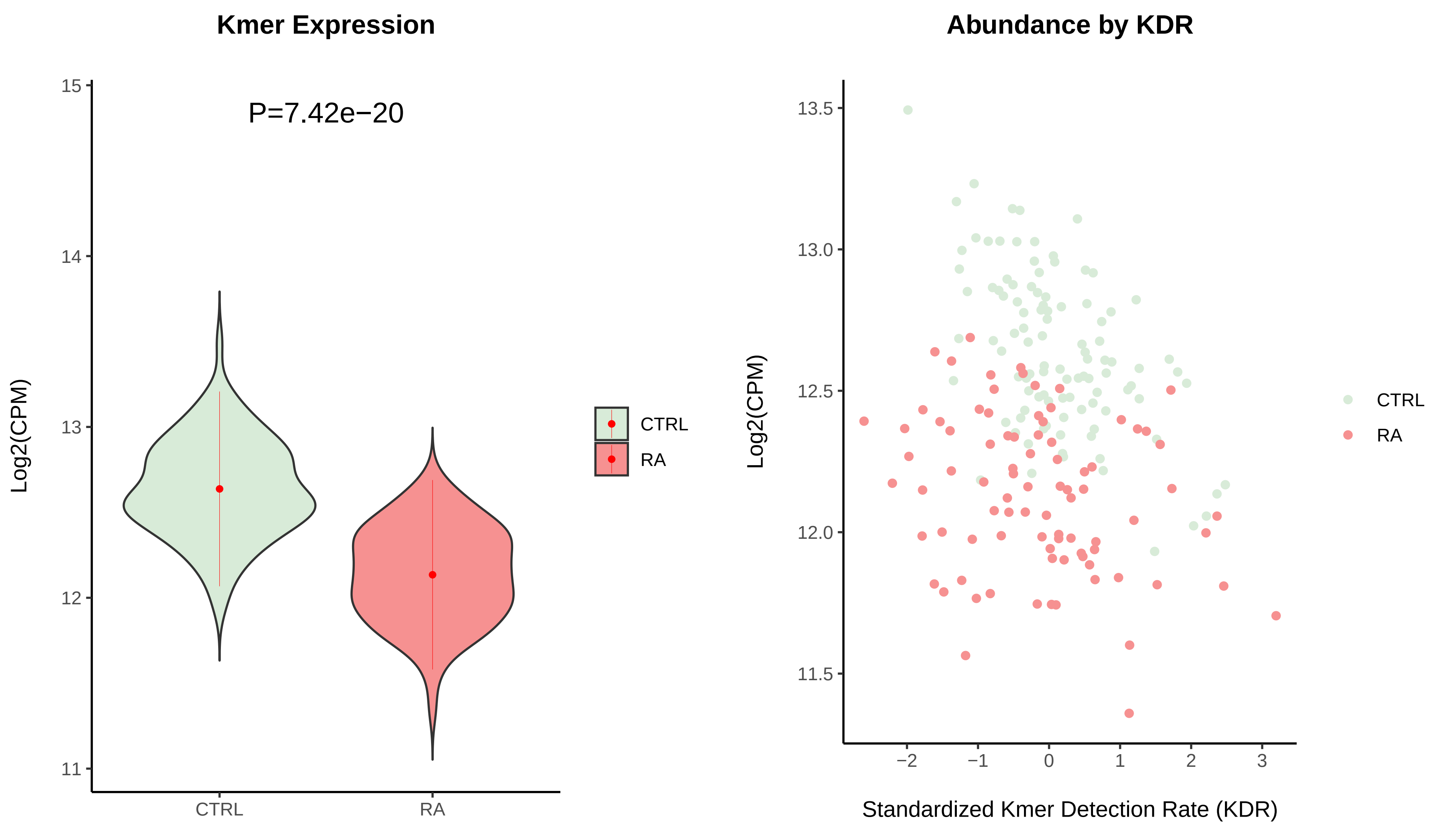

GTHW from IGK chain significant in Hurdle model

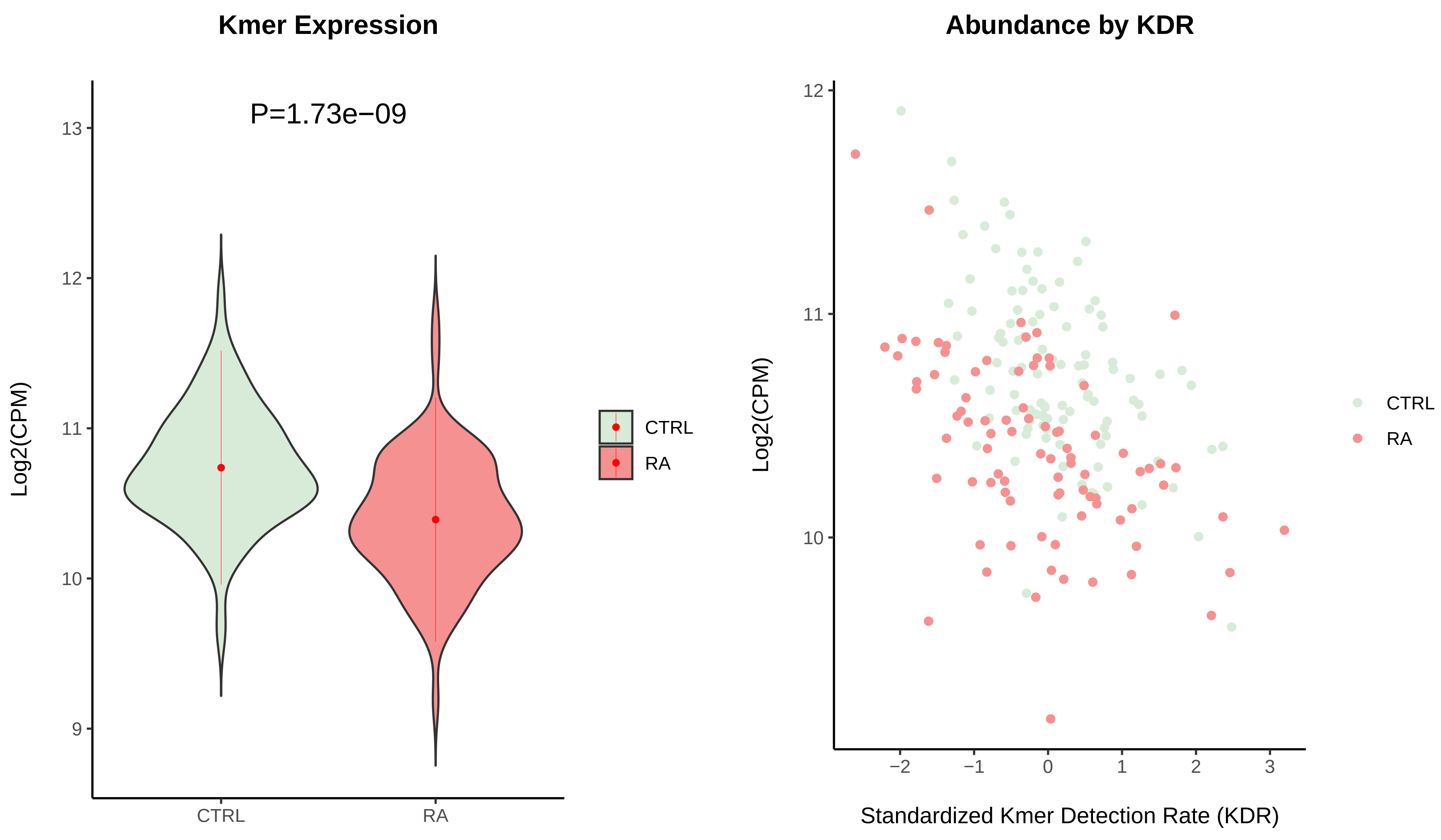

HWPP from IGK chain significant in Hurdle model

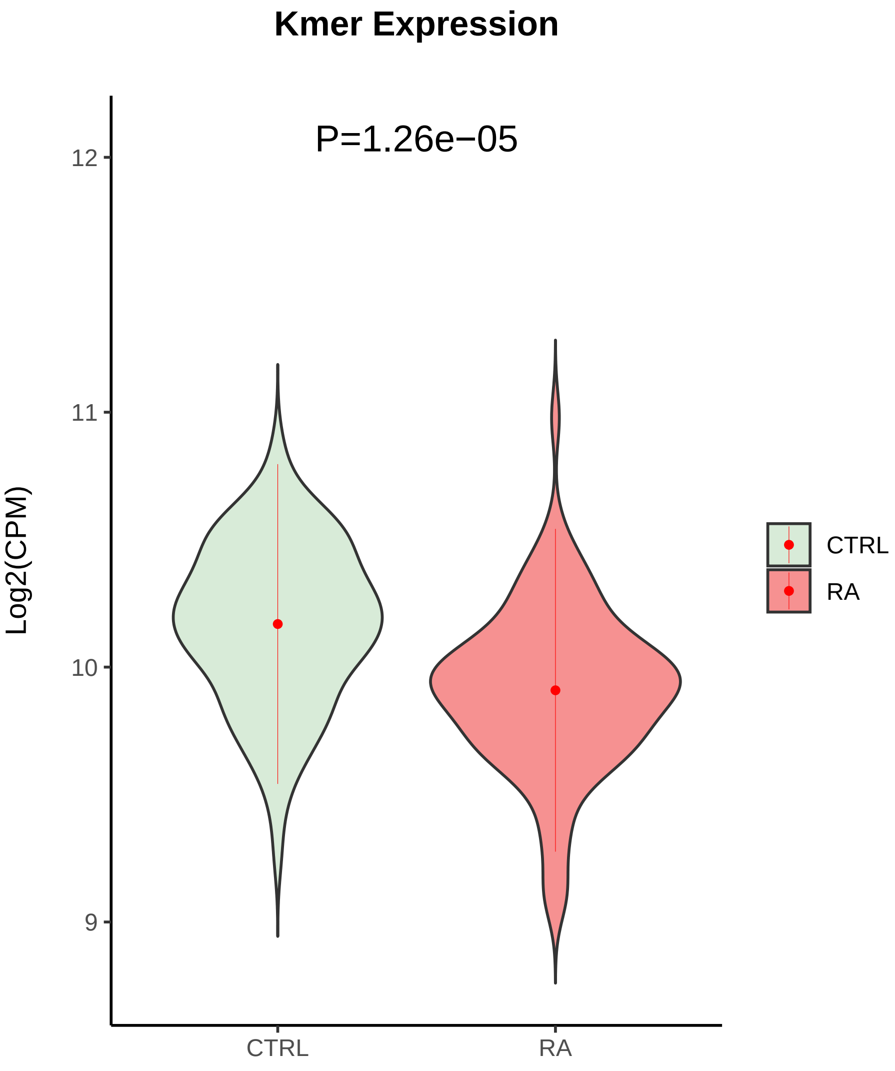

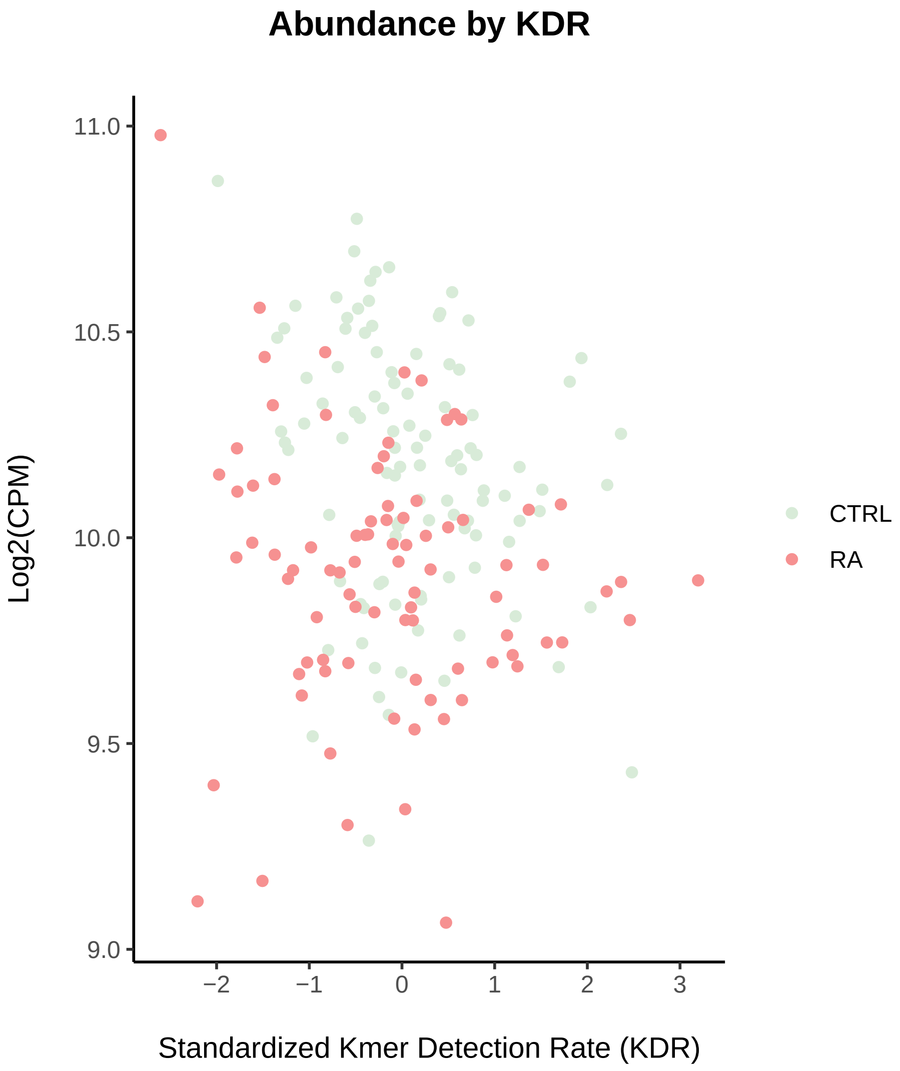

IQVP from IGK chain significant in Hurdle model

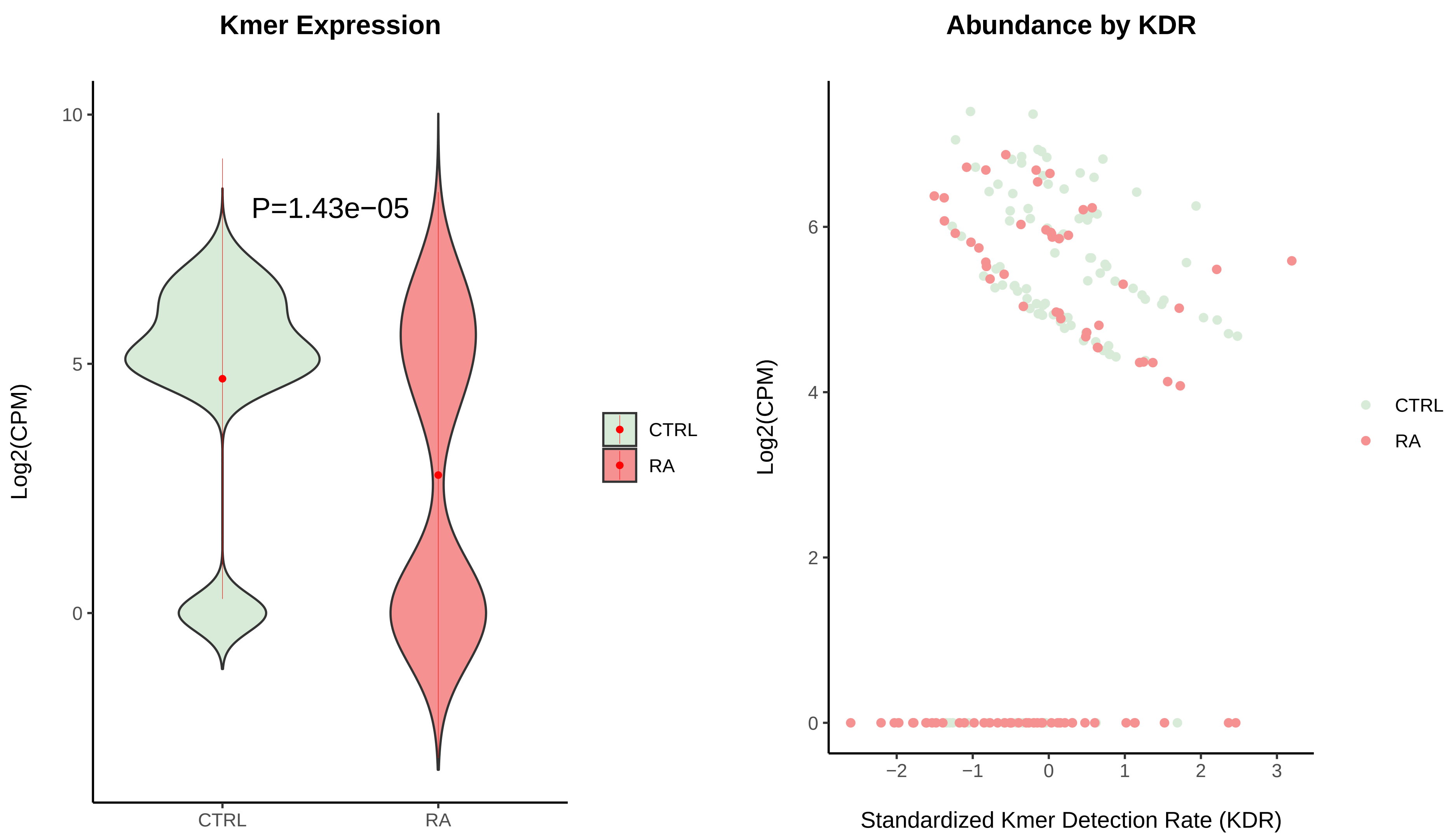

KSSP from IGK chain significant in Hurdle model

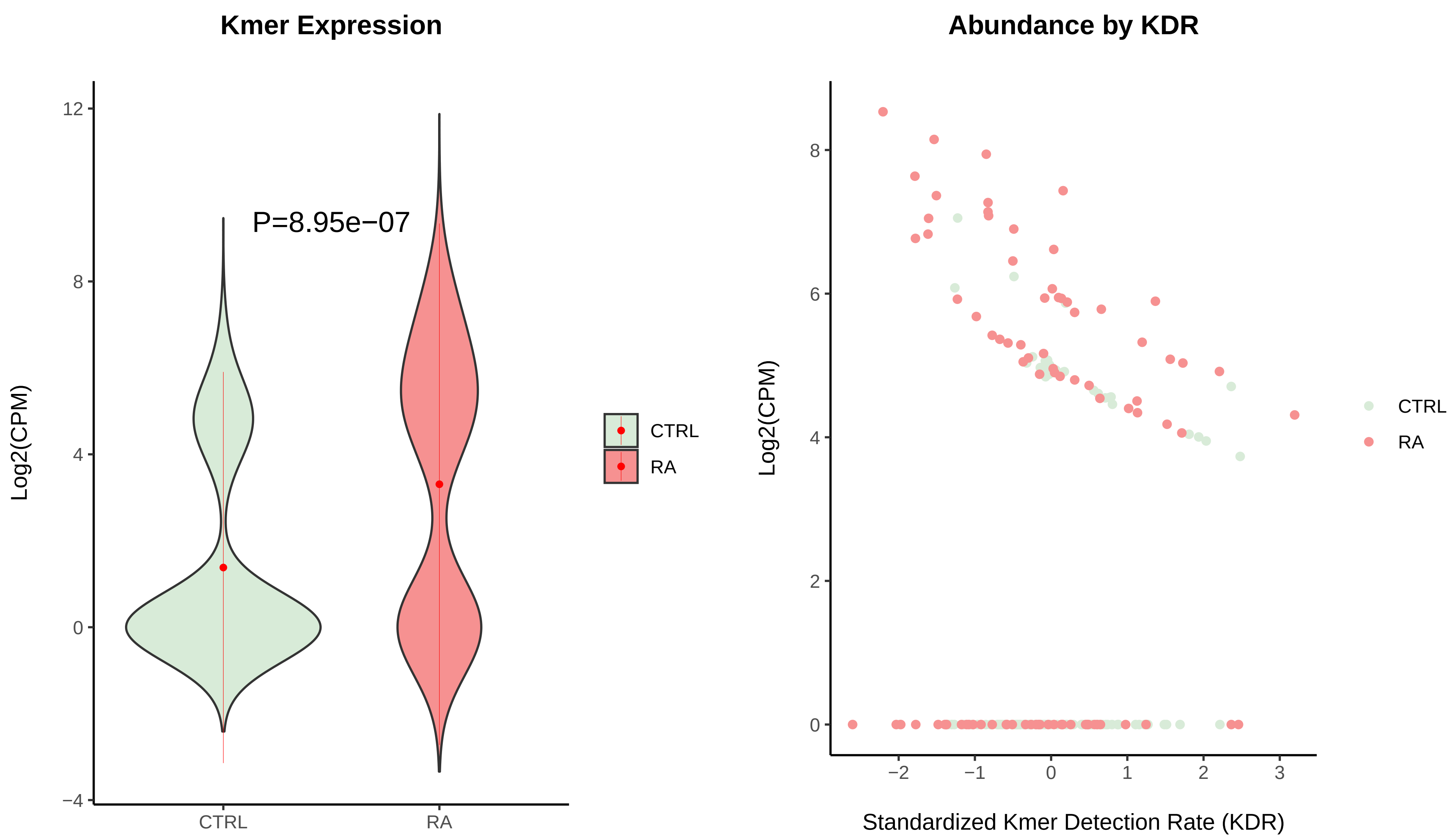

LQIP from IGK chain significant in Hurdle model

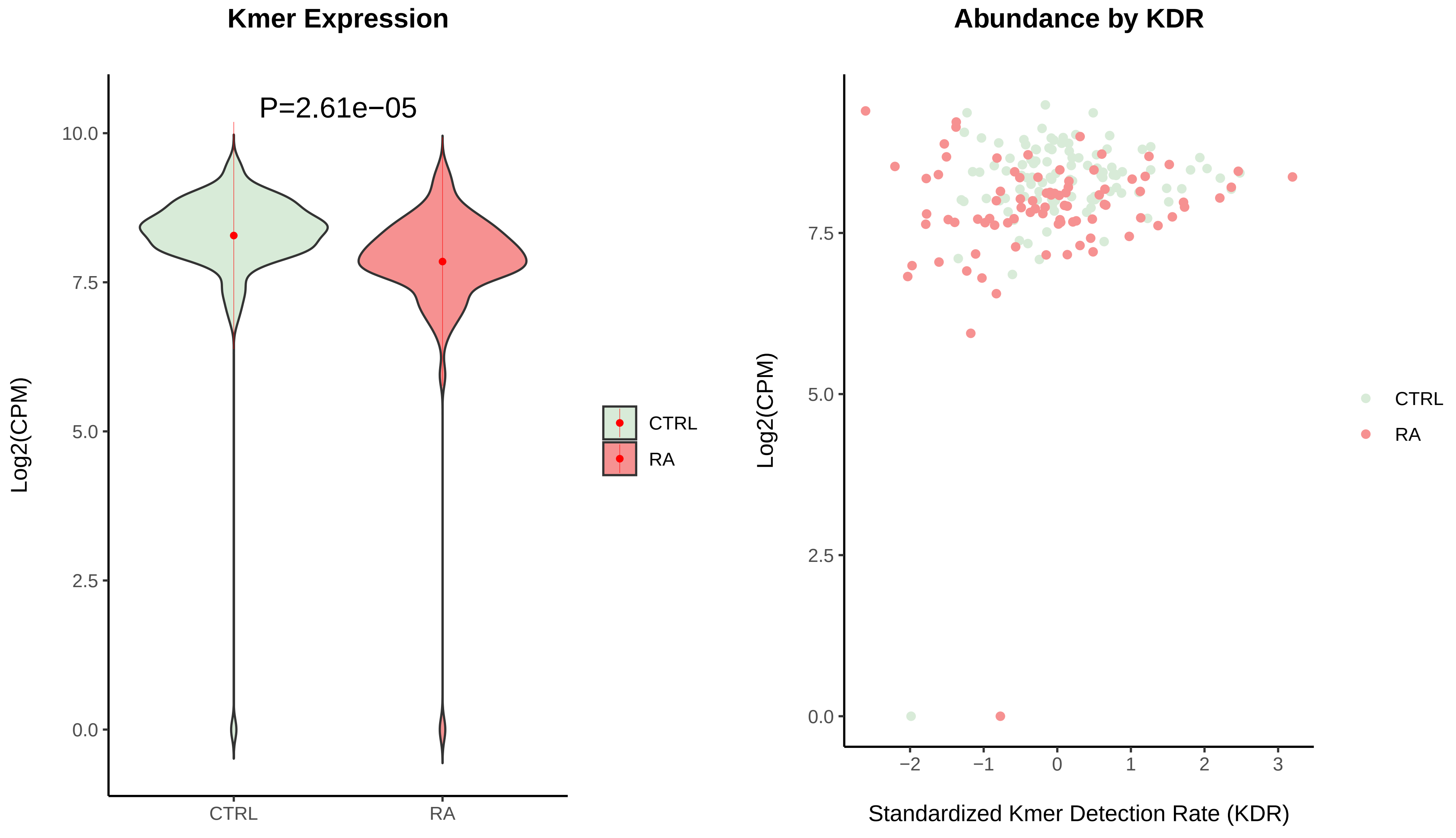

LQTA from IGK chain significant in Hurdle model

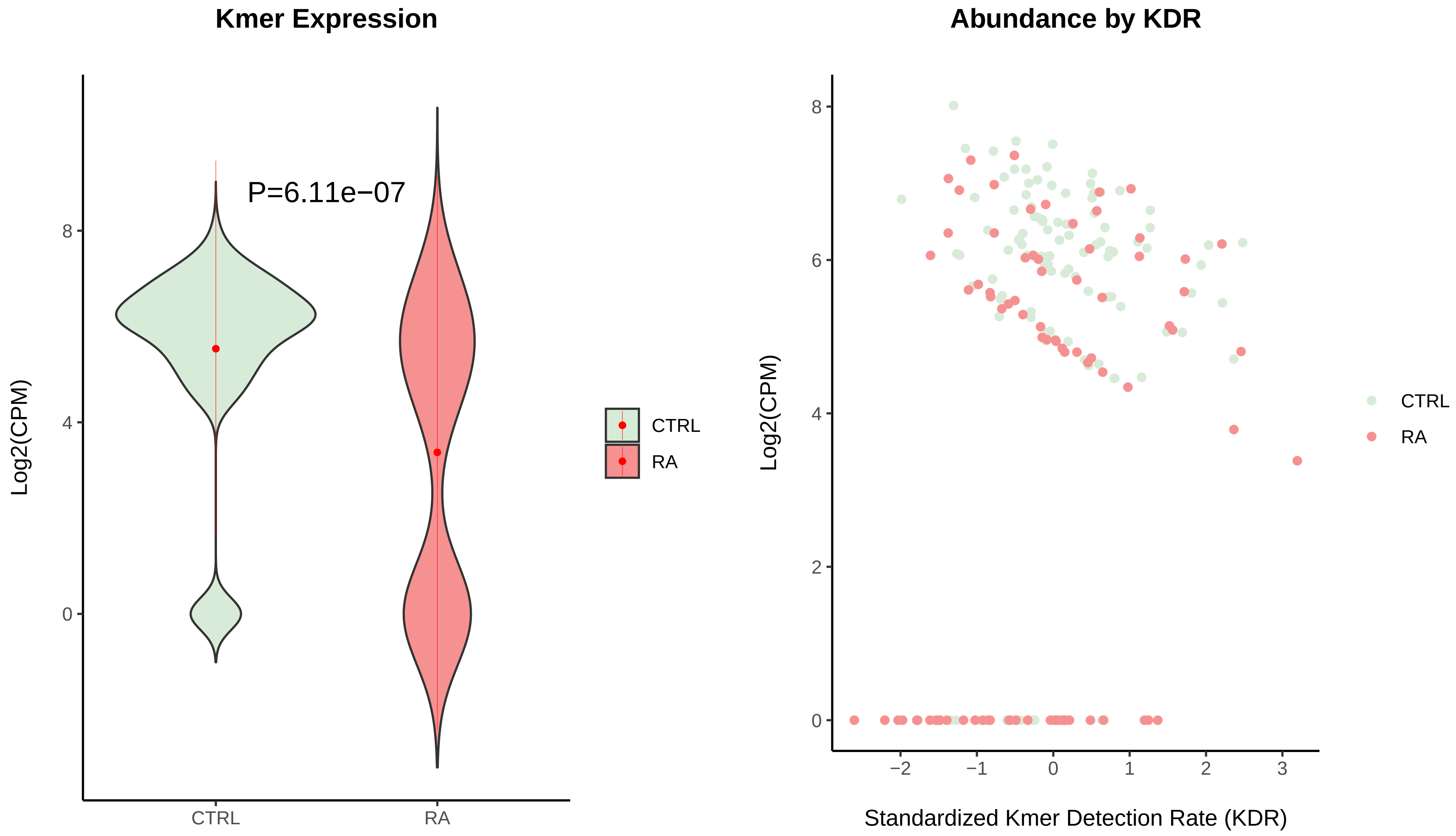

LQTG from IGK chain significant in Hurdle model

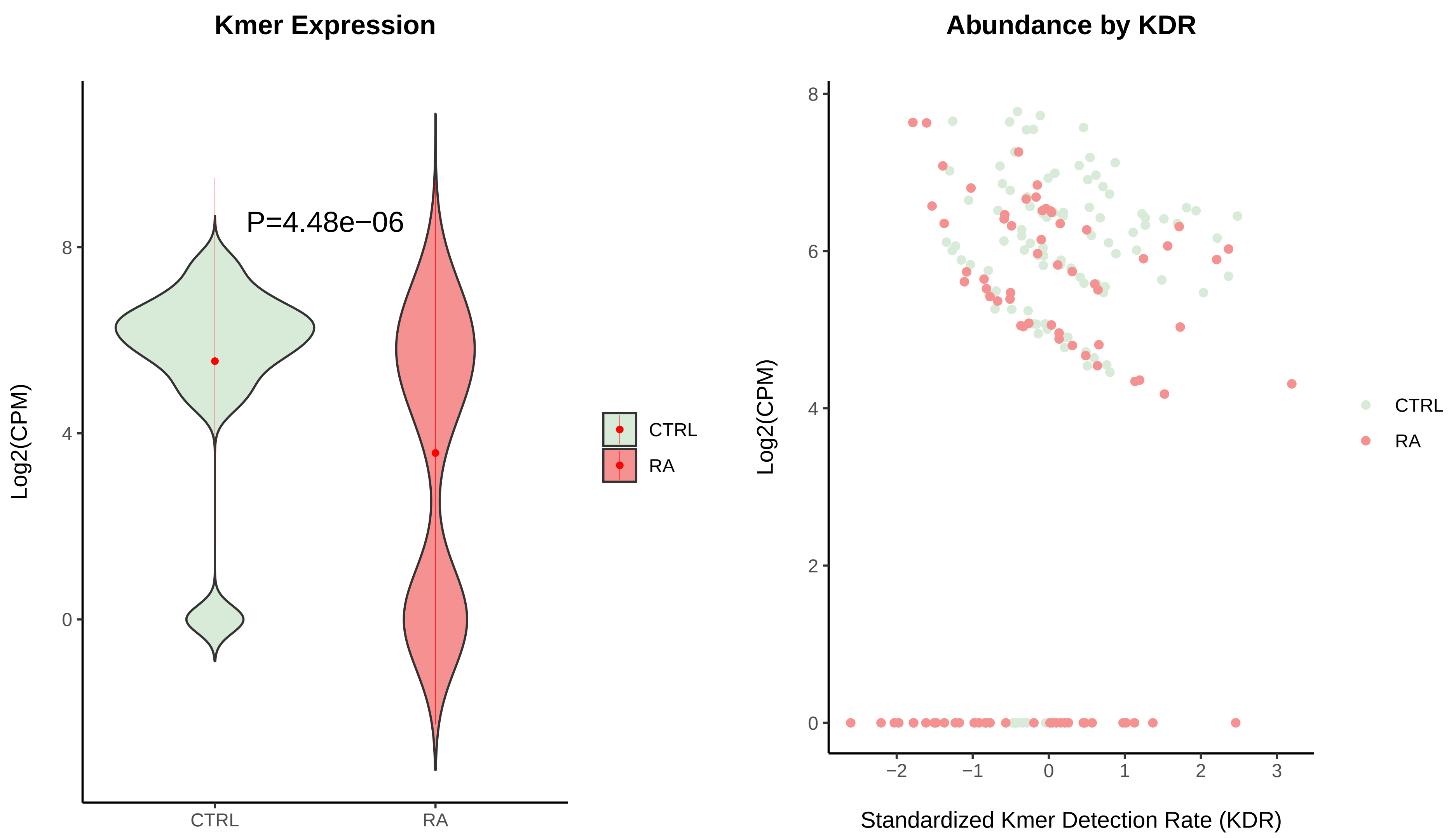

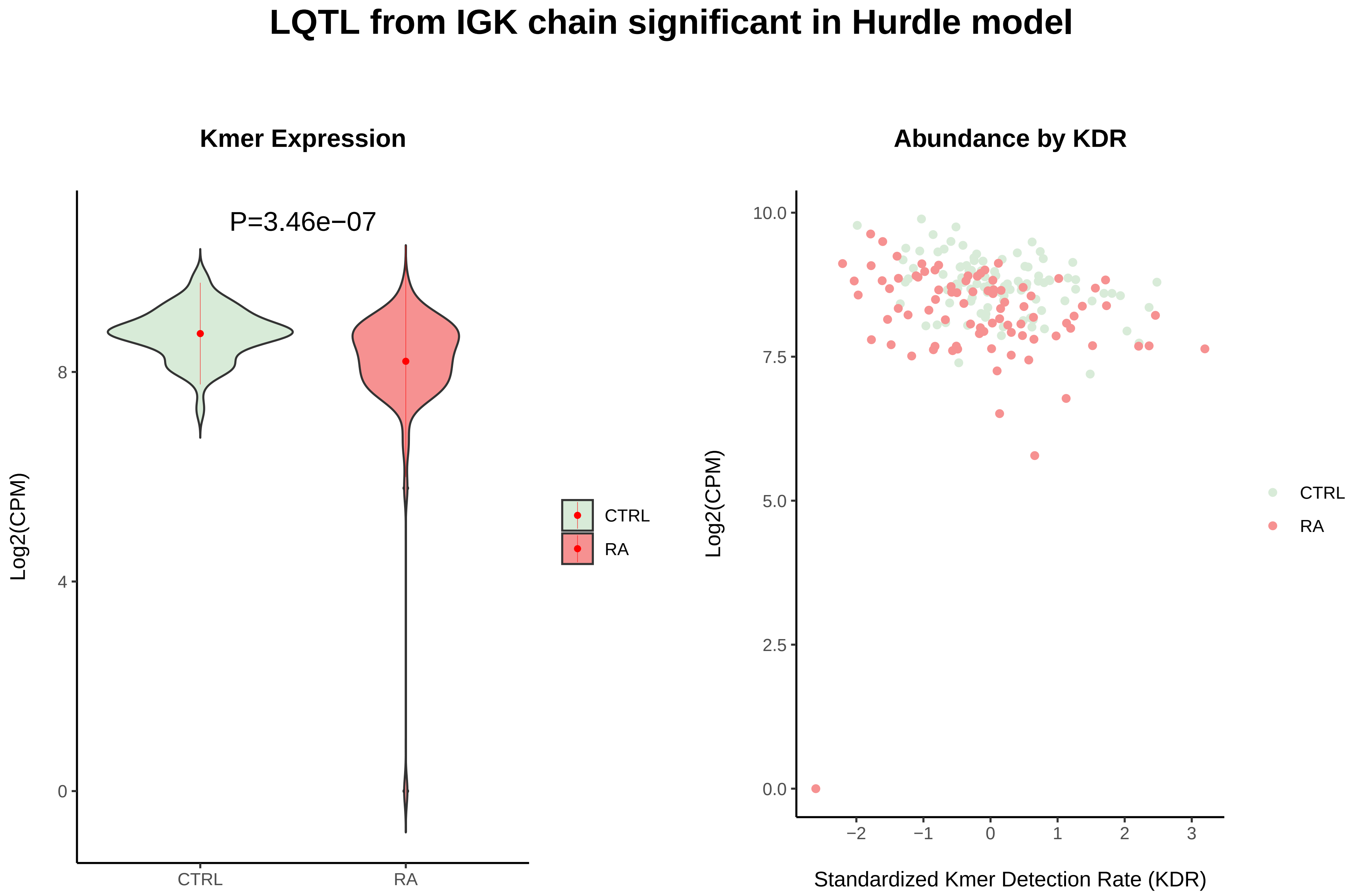
LQTL from IGK chain significant in Hurdle model
Kmer Expression
P=3.46e−07
Log2(CPM)
8
4
0
CTRL
RA
CTRL
RA
Abundance by KDR
10.0
7.5
5.0
2.5
0.0
Log2(CPM)
−2
−1
0
1
2
3
Standardized Kmer Detection Rate (KDR)
CTRL
RA

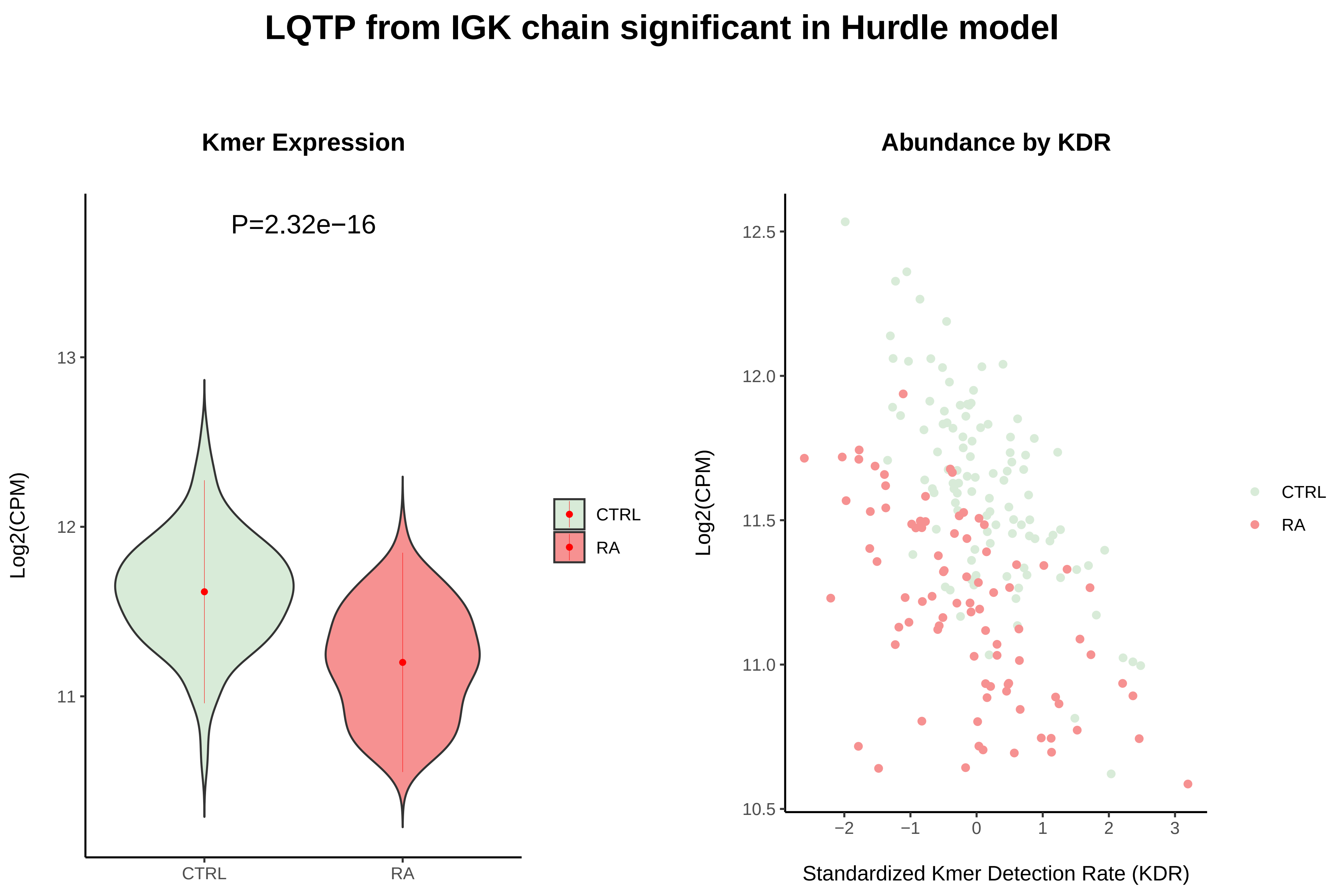
LQTP from IGK chain significant in Hurdle model
Kmer Expression
P=2.32e−16
Log2(CPM)
13
12
11
CTRL
RA
CTRL
RA
Abundance by KDR
Log2(CPM)
12.5
12.0
11.5
11.0
10.5
−2
−1
0
1
2
3
Standardized Kmer Detection Rate (KDR)
CTRL
RA

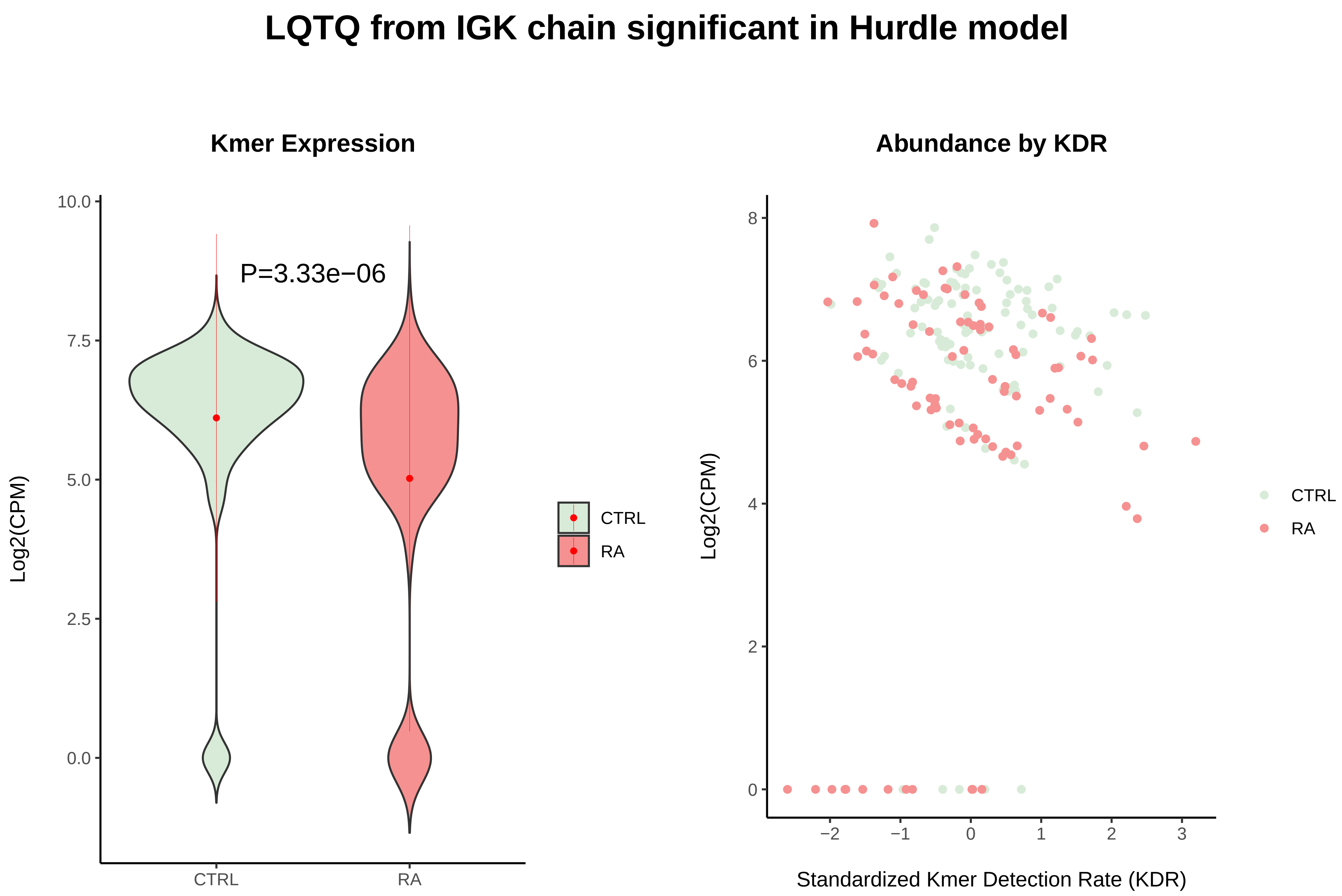

LQTQ from IGK chain significant in Hurdle model
Kmer Expression
P=3.33e−06
Log2(CPM)
10.0
7.5
5.0
2.5
0.0
CTRL
RA
CTRL
RA
Abundance by KDR
Log2(CPM)
8
6
4
2
0
−2
−1
0
1
2
3
Standardized Kmer Detection Rate (KDR)
CTRL
RA

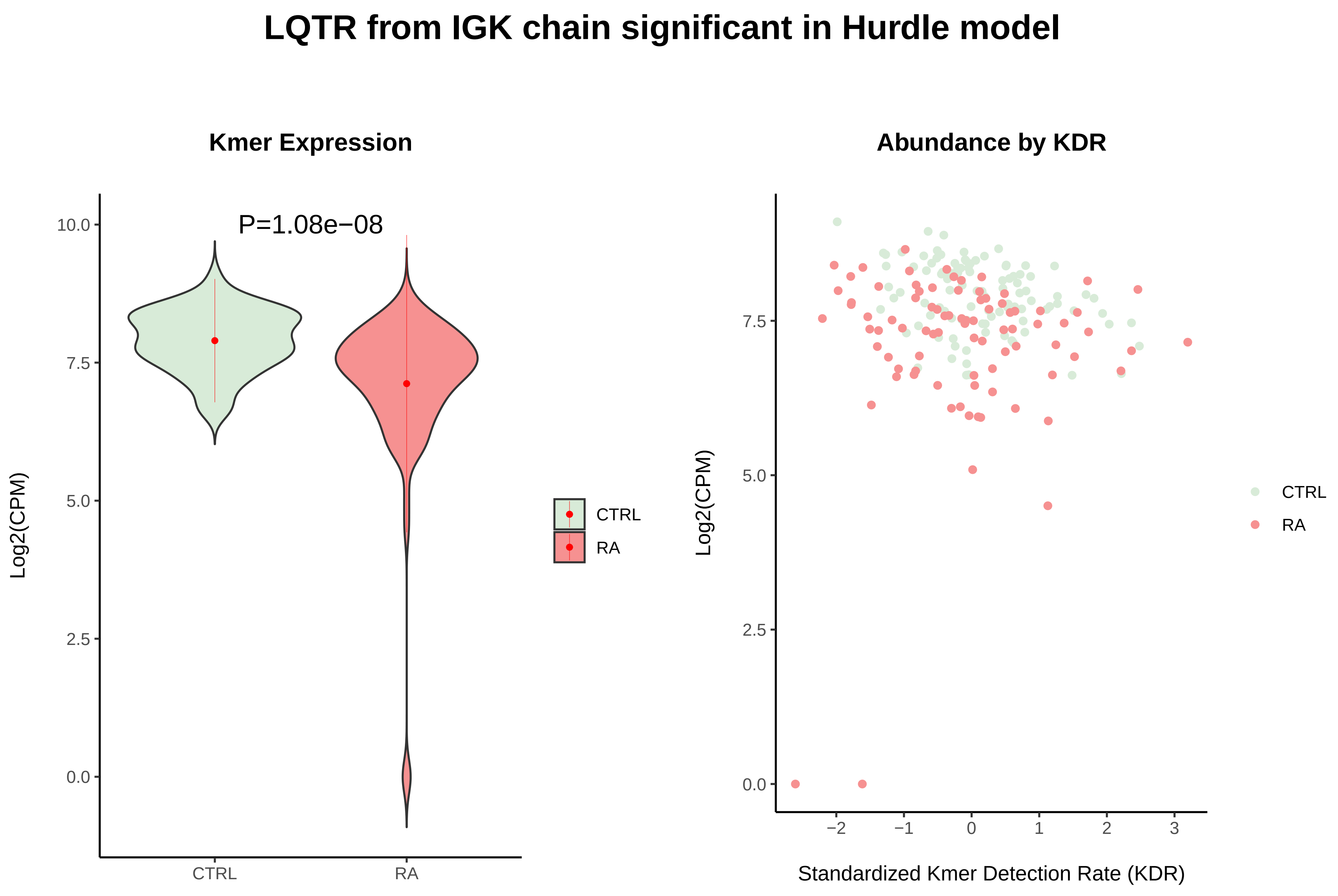
LQTR from IGK chain significant in Hurdle model
Kmer Expression
P=1.08e−08
Log2(CPM)
10.0
7.5
5.0
2.5
0.0
CTRL
RA
CTRL
RA
Abundance by KDR
Log2(CPM)
7.5
5.0
2.5
0.0
−2
−1
0
1
2
3
Standardized Kmer Detection Rate (KDR)
CTRL
RA

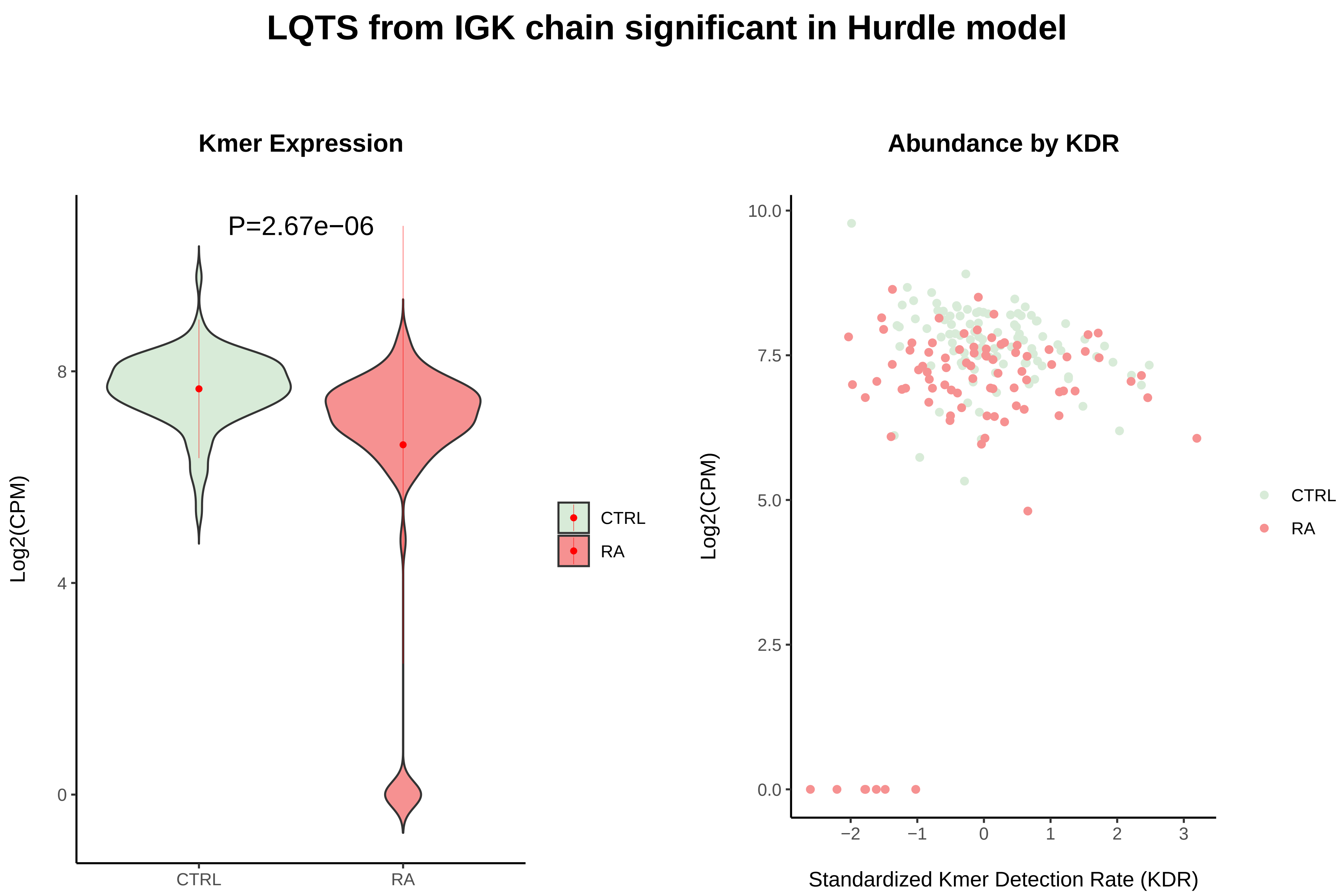
LQTS from IGK chain significant in Hurdle model
Kmer Expression
P=2.67e−06
Log2(CPM)
8
4
0
CTRL
RA
CTRL
RA
Abundance by KDR
10.0
7.5
5.0
2.5
0.0
Log2(CPM)
−2
−1
0
1
2
3
Standardized Kmer Detection Rate (KDR)
CTRL
RA

MQAL from IGK chain significant in Hurdle model

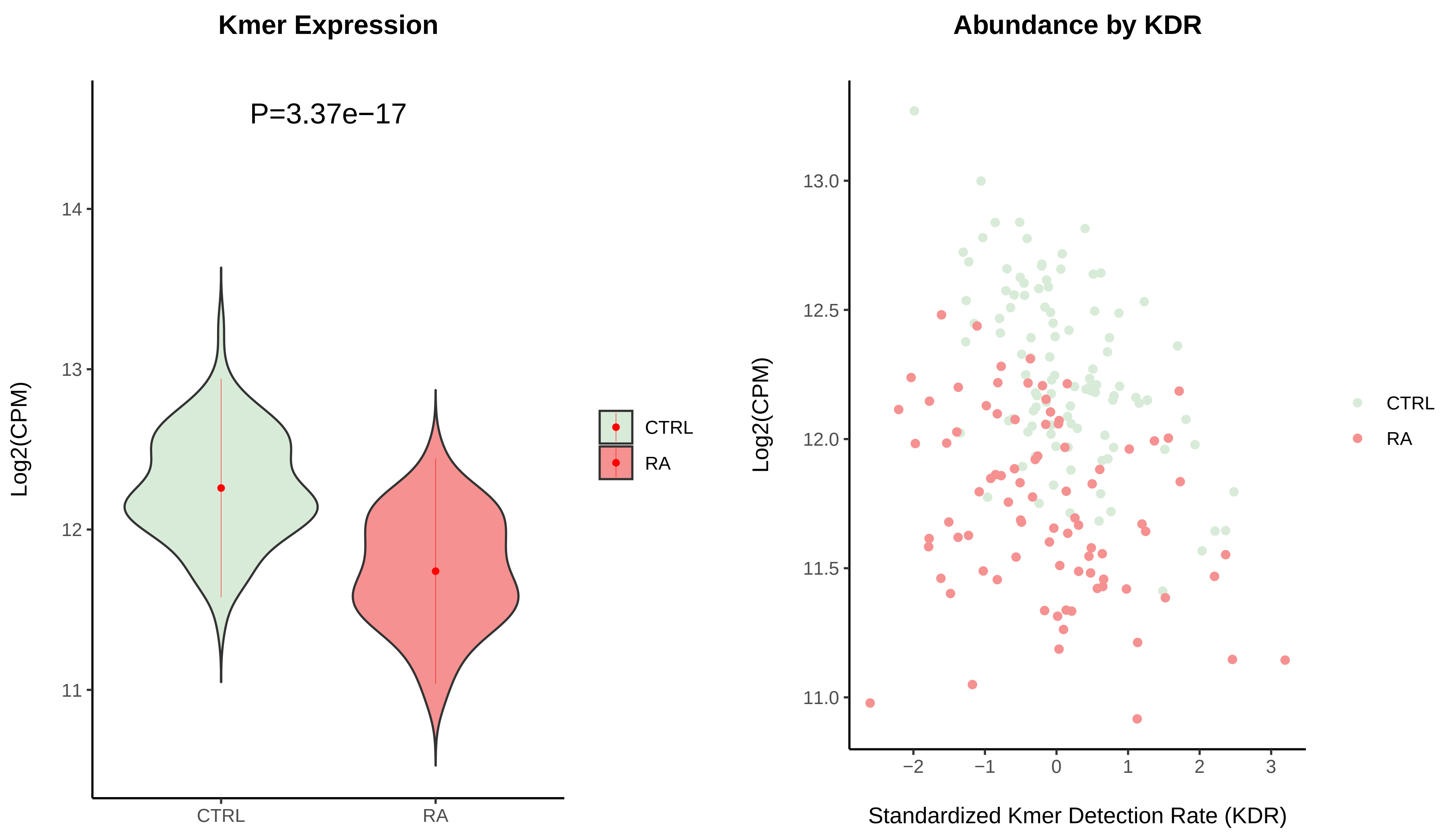

MQAT from IGK chain significant in Hurdle model

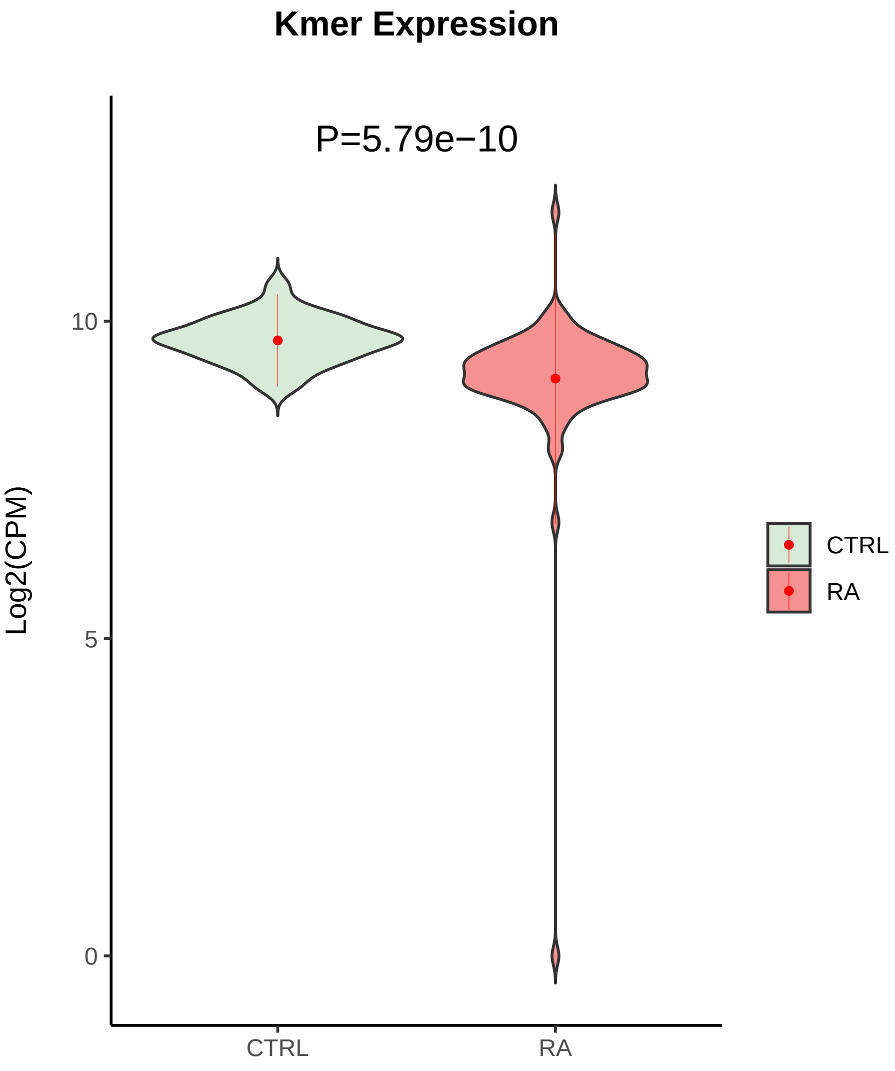

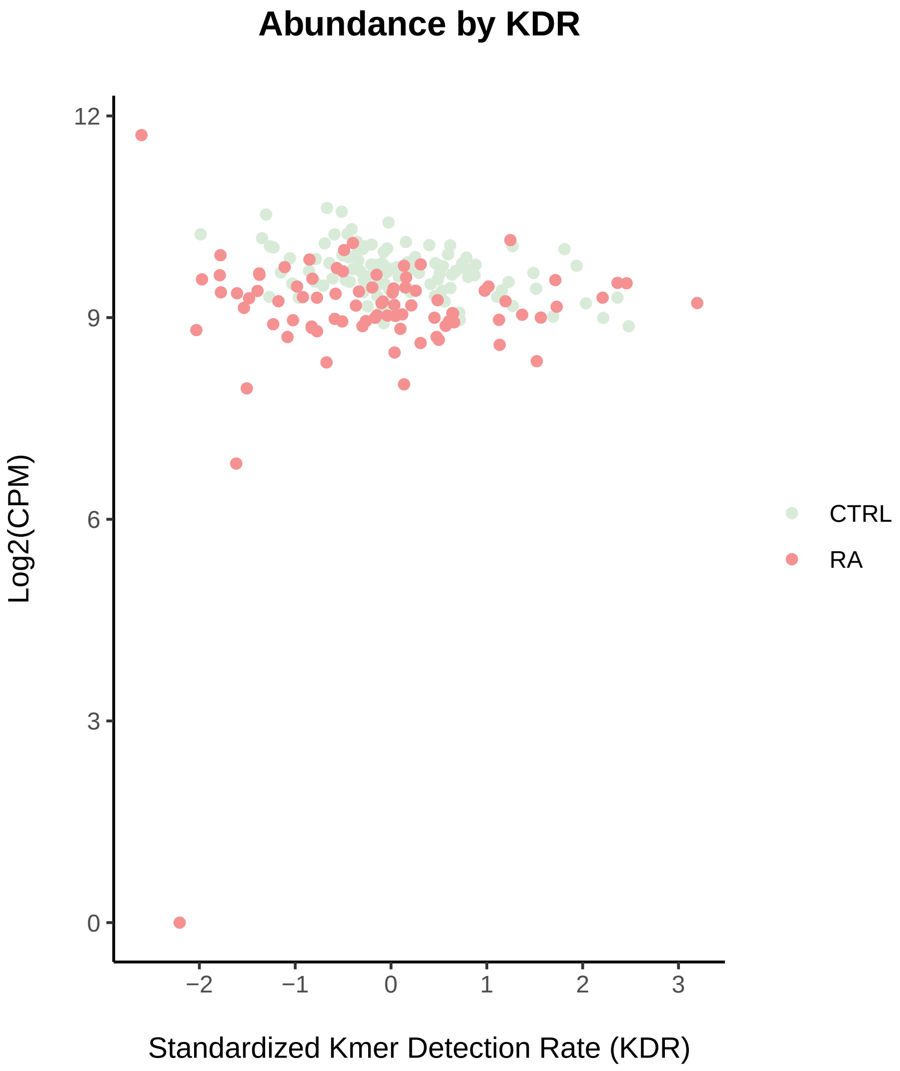

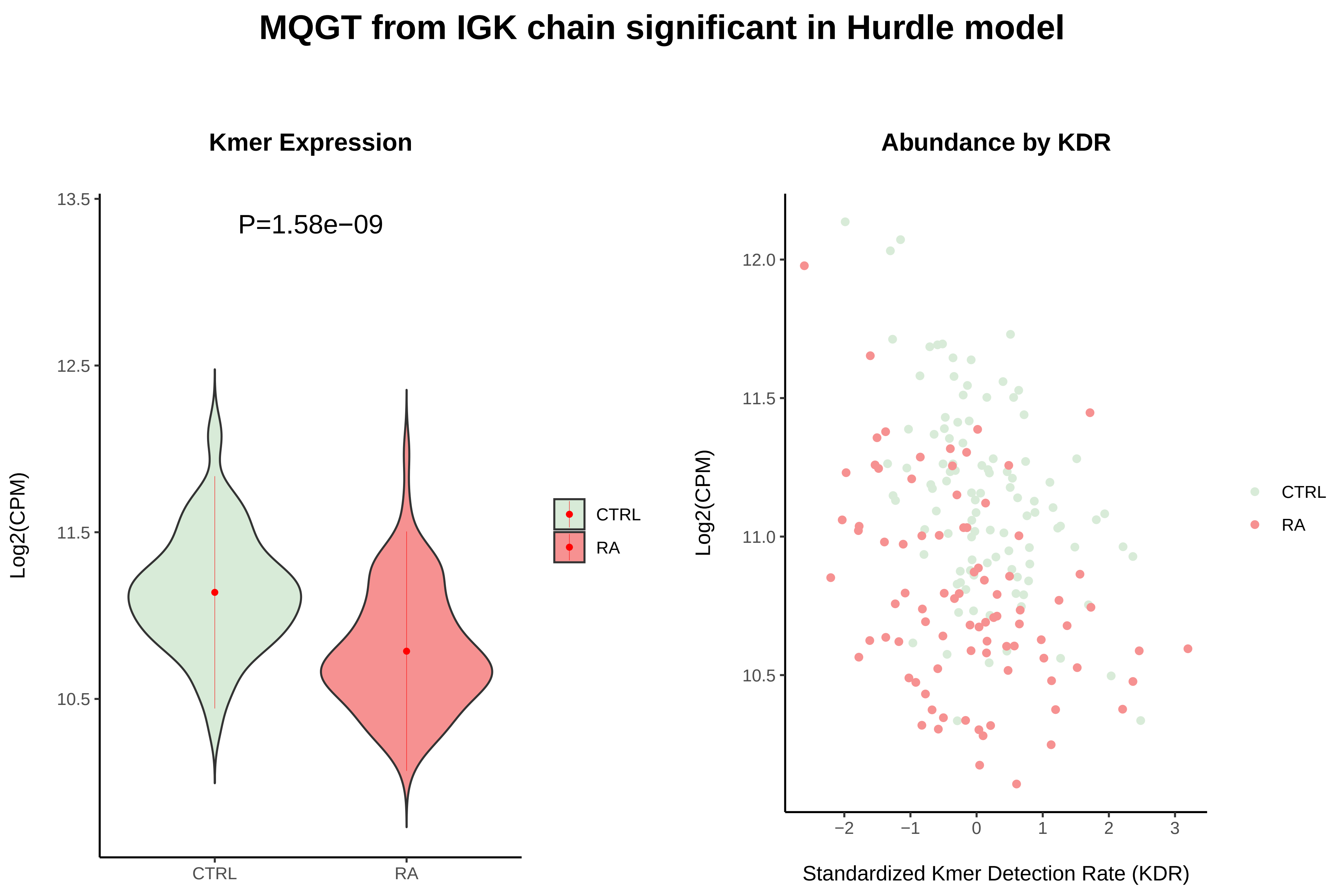
MQGT from IGK chain significant in Hurdle model
Kmer Expression
P=1.58e−09
Log2(CPM)
13.5
12.5
11.5
10.5
CTRL
RA
CTRL
RA
Abundance by KDR
Log2(CPM)
12.0
11.5
11.0
10.5
−2
−1
0
1
2
3
Standardized Kmer Detection Rate (KDR)
CTRL
RA

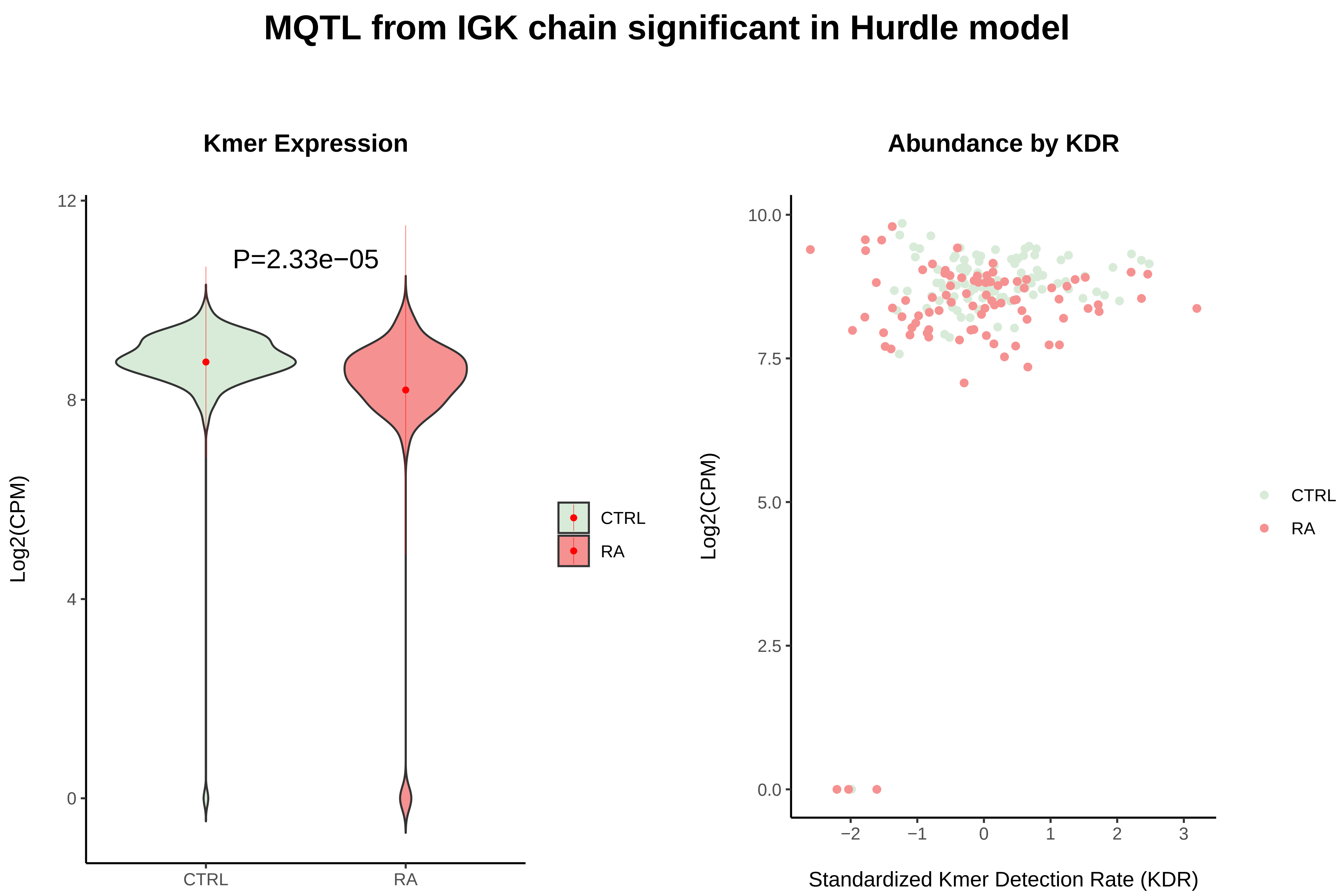
MQTL from IGK chain significant in Hurdle model
Kmer Expression
P=2.33e−05
Log2(CPM)
12
8
4
0
CTRL
RA
CTRL
RA
Abundance by KDR
Log2(CPM)
10.0
7.5
5.0
2.5
0.0
−2
−1
0
1
2
3
Standardized Kmer Detection Rate (KDR)
CTRL
RA

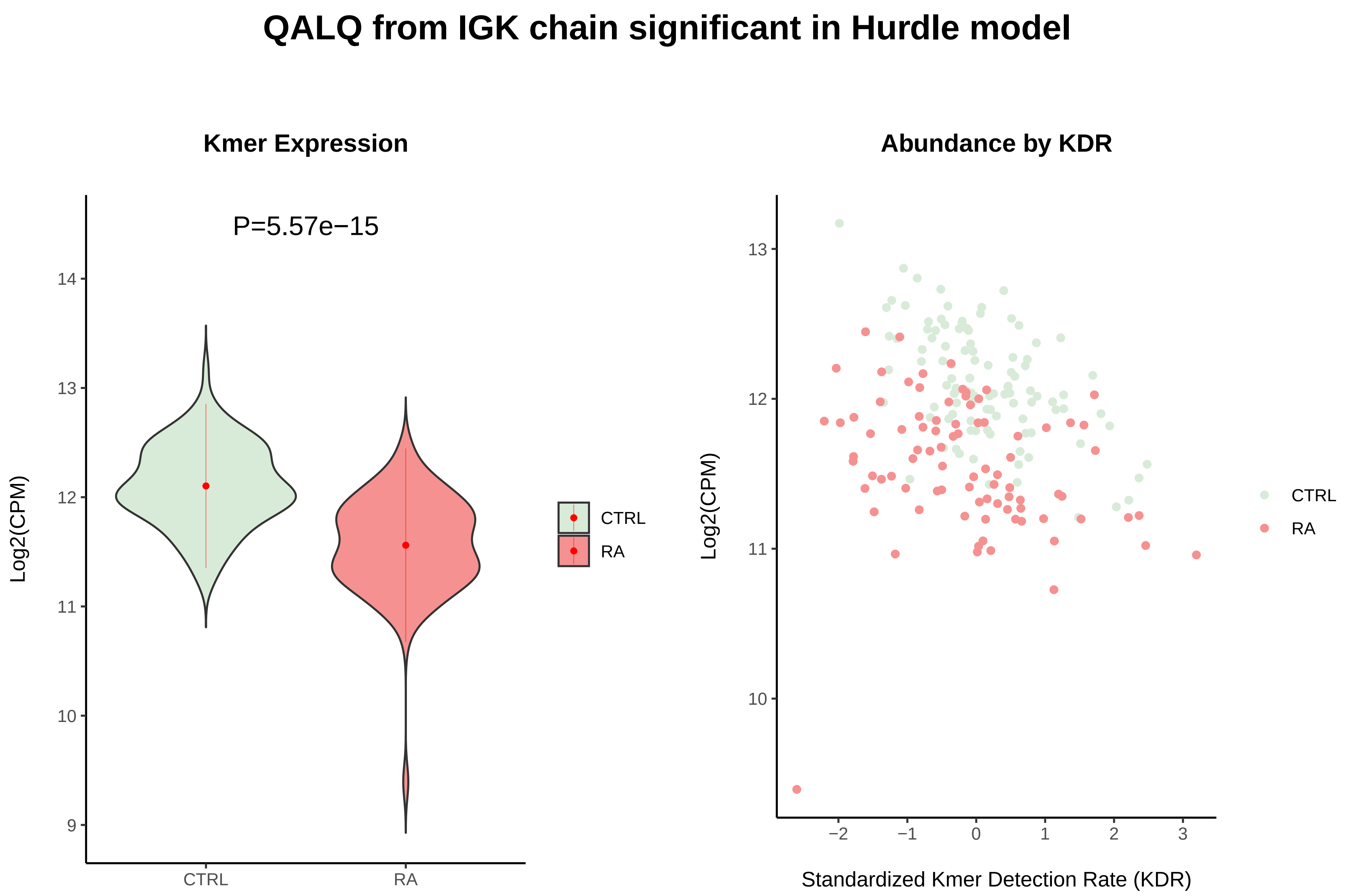
QALQ from IGK chain significant in Hurdle model
Kmer Expression
P=5.57e−15
Log2(CPM)
14
13
12
11
10
9
CTRL
RA
CTRL
RA
Abundance by KDR
Log2(CPM)
13
12
11
10
−2
−1
0
1
2
3
Standardized Kmer Detection Rate (KDR)
CTRL
RA

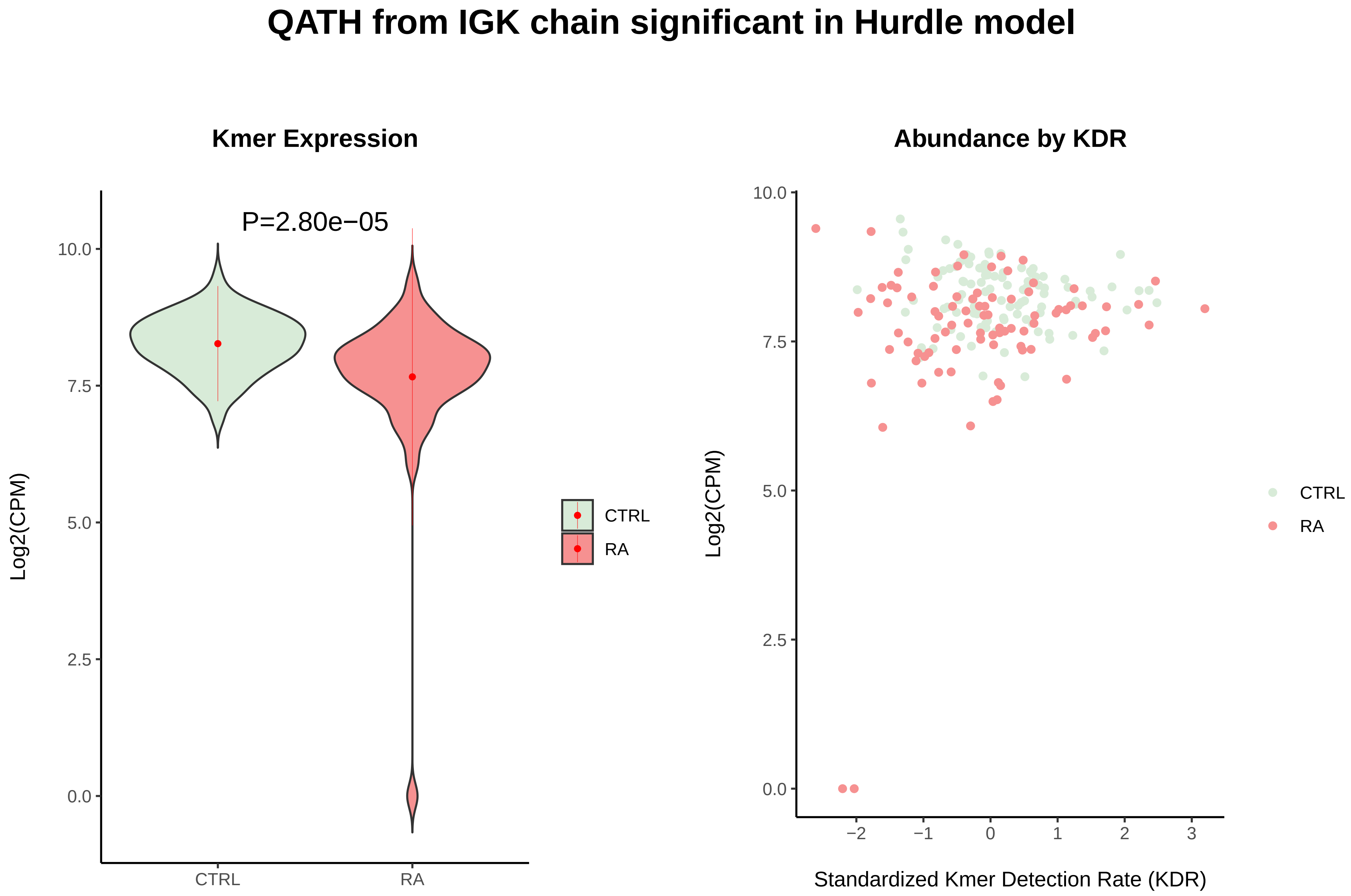
QATH from IGK chain significant in Hurdle model
Kmer Expression
P=2.80e−05
Log2(CPM)
10.0
7.5
5.0
2.5
0.0
CTRL
RA
CTRL
RA
Abundance by KDR
Log2(CPM)
10.0
7.5
5.0
2.5
0.0
−2
−1
0
1
2
3
Standardized Kmer Detection Rate (KDR)
CTRL
RA

QATQ from IGK chain significant in Hurdle model

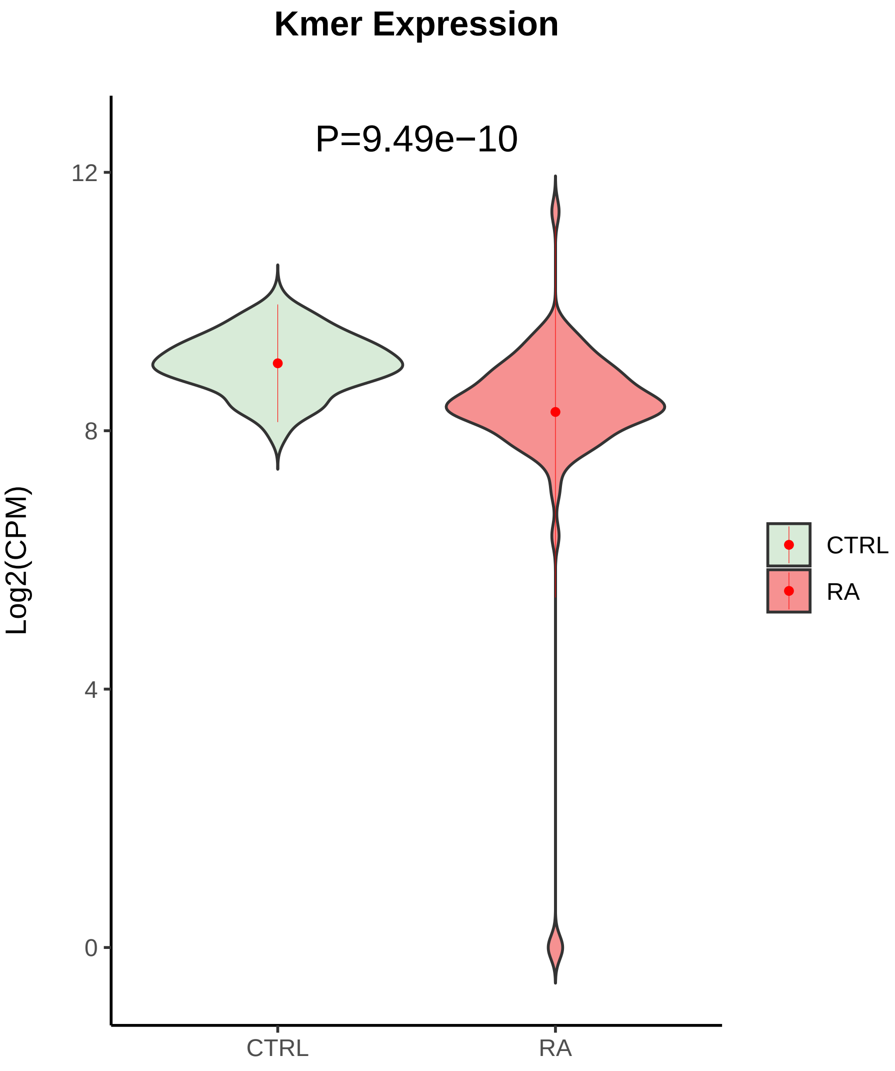

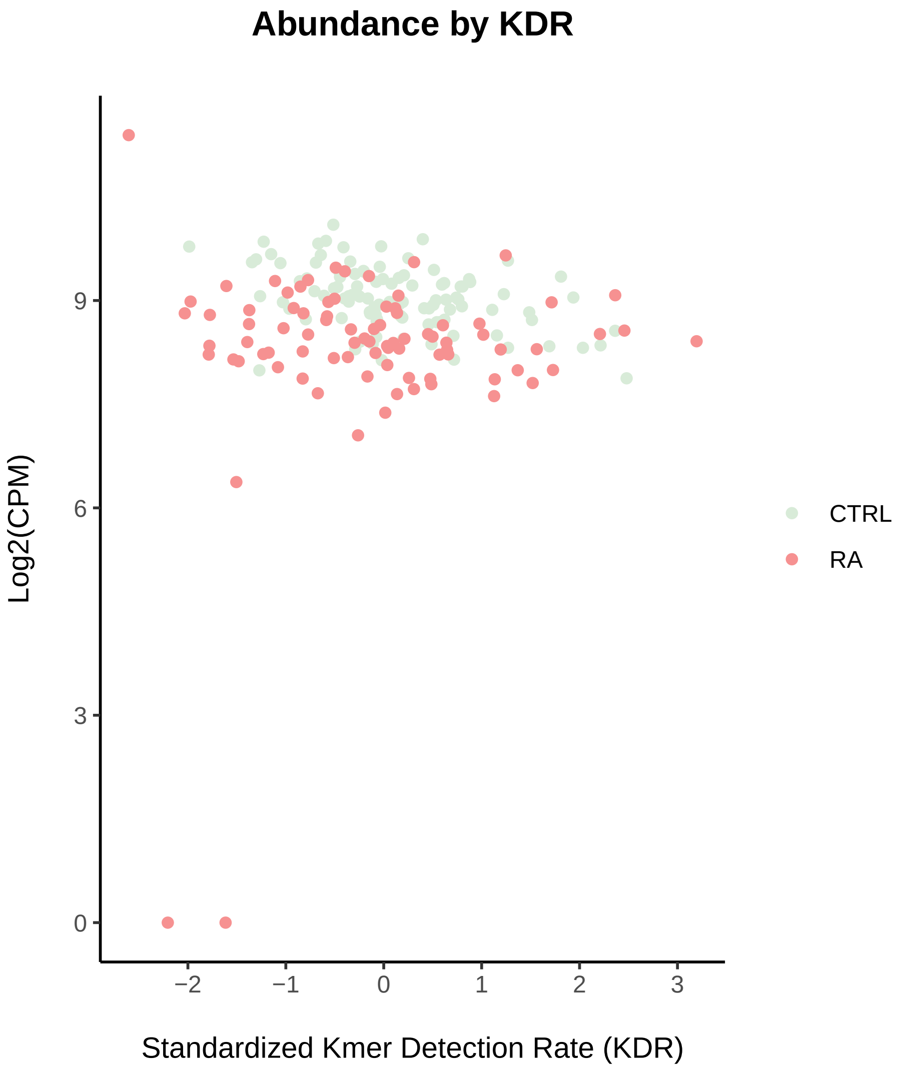

QFPA from IGK chain significant in Hurdle model

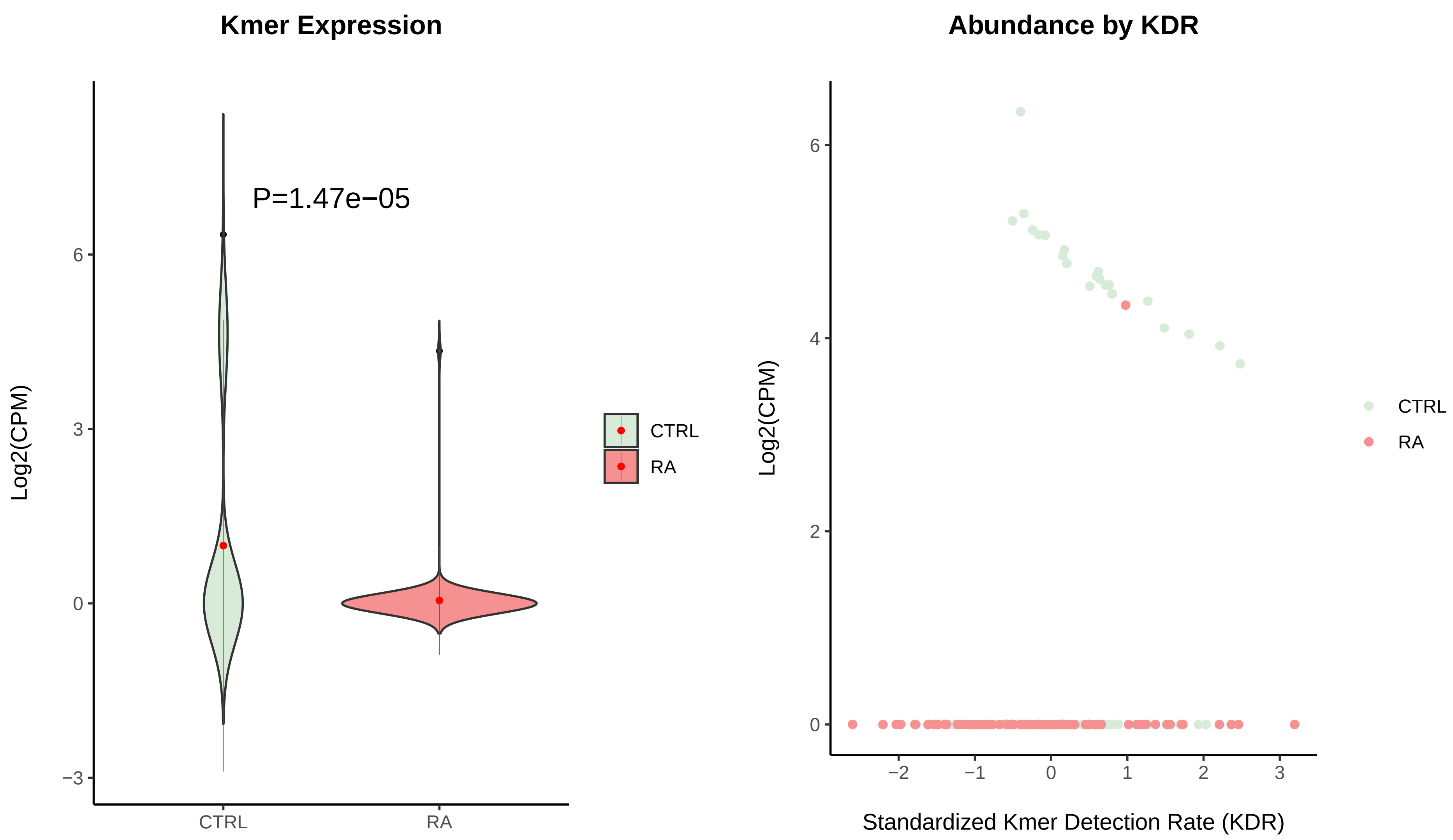

QGTH from IGK chain significant in Hurdle model

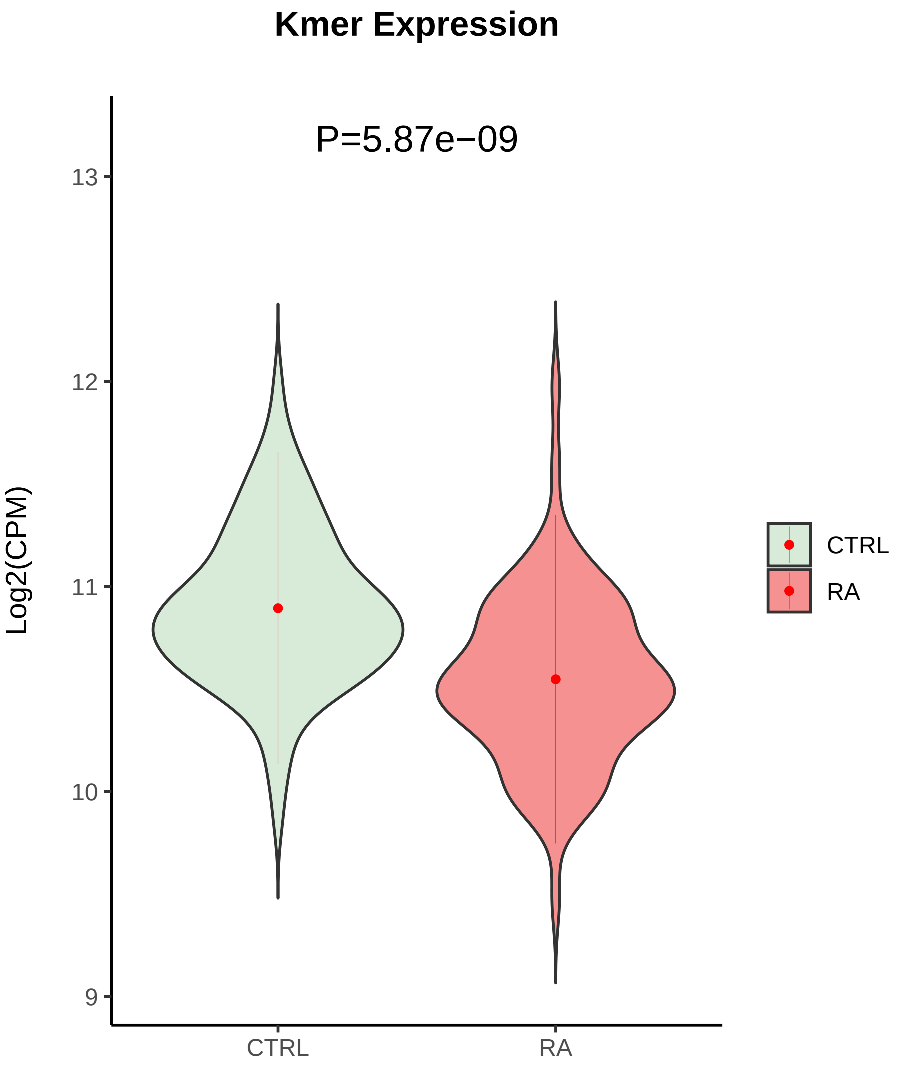

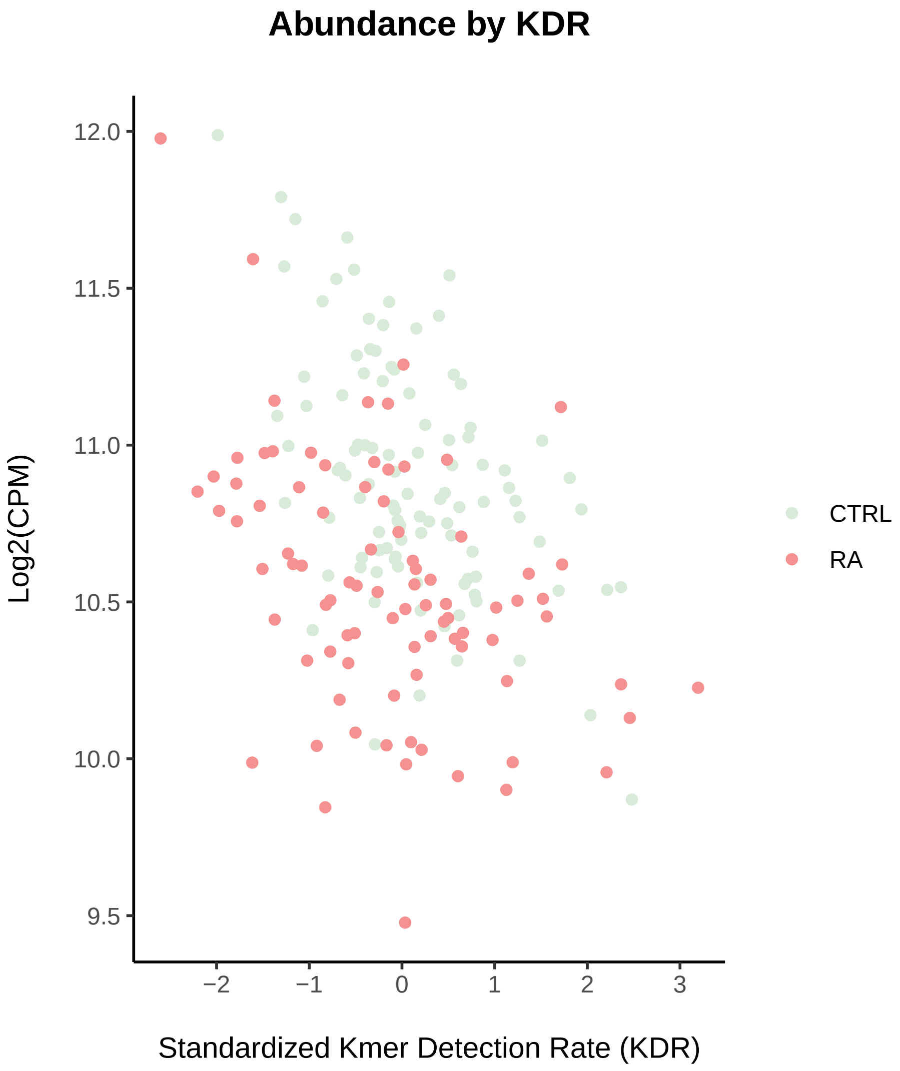

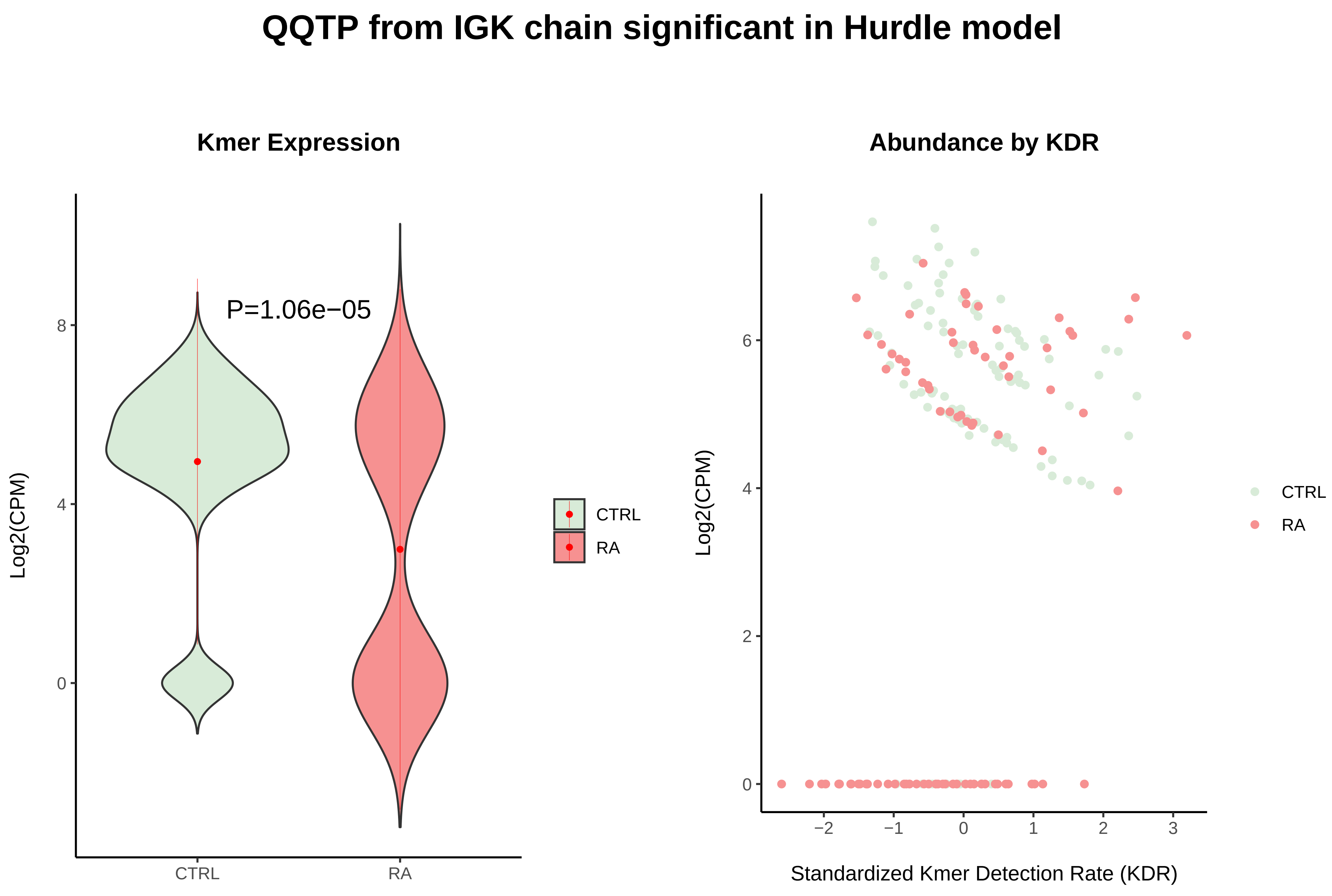
QQTP from IGK chain significant in Hurdle model
Kmer Expression
P=1.06e−05
Log2(CPM)
8
4
0
CTRL
RA
CTRL
RA
Abundance by KDR
Log2(CPM)
6
4
2
0
−2
−1
0
1
2
3
Standardized Kmer Detection Rate (KDR)
CTRL
RA

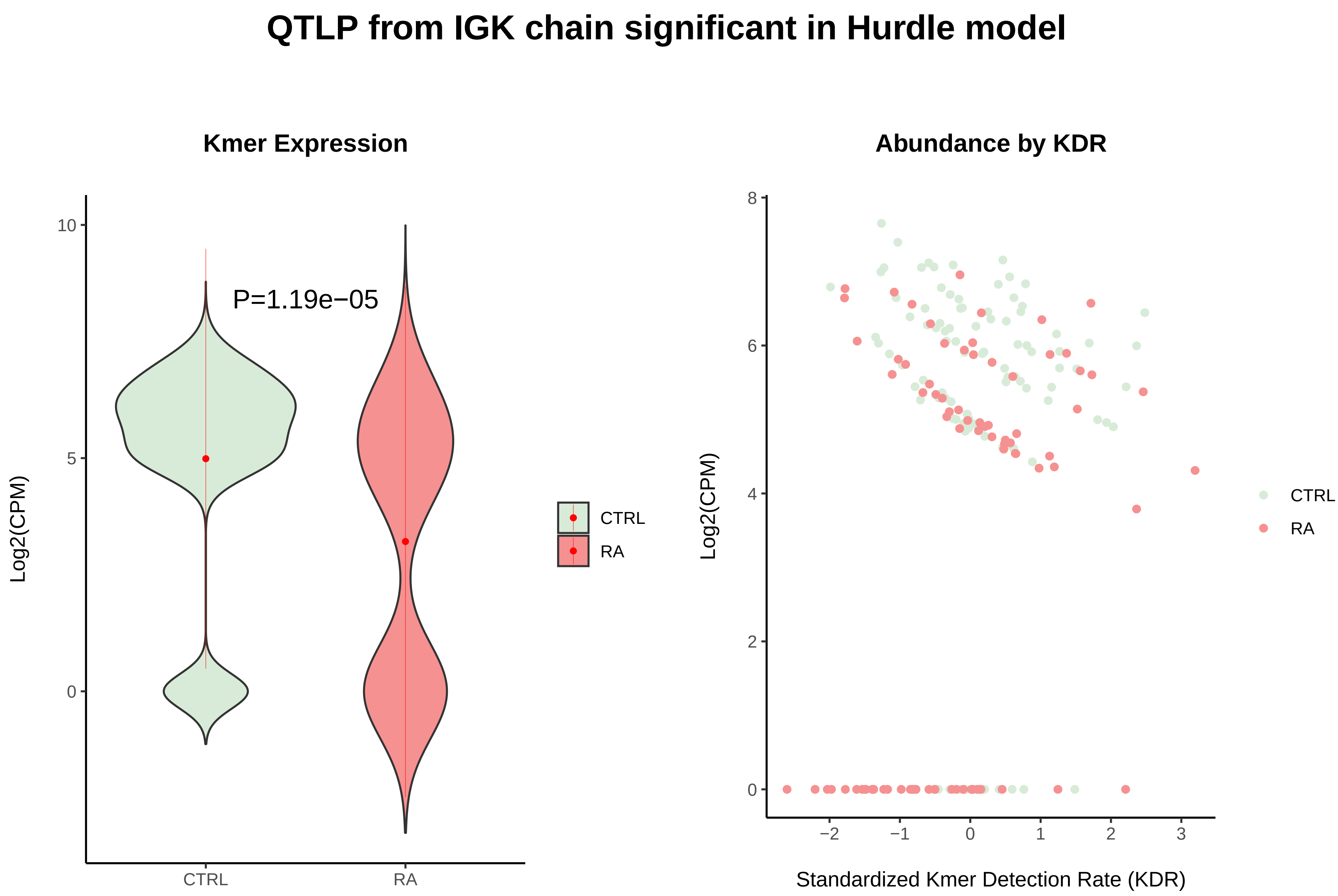

QTLP from IGK chain significant in Hurdle model
Kmer Expression
P=1.19e−05
Log2(CPM)
10
5
0
CTRL
RA
CTRL
RA
Abundance by KDR
Log2(CPM)
8
6
4
2
0
−2
−1
0
1
2
3
Standardized Kmer Detection Rate (KDR)
CTRL
RA

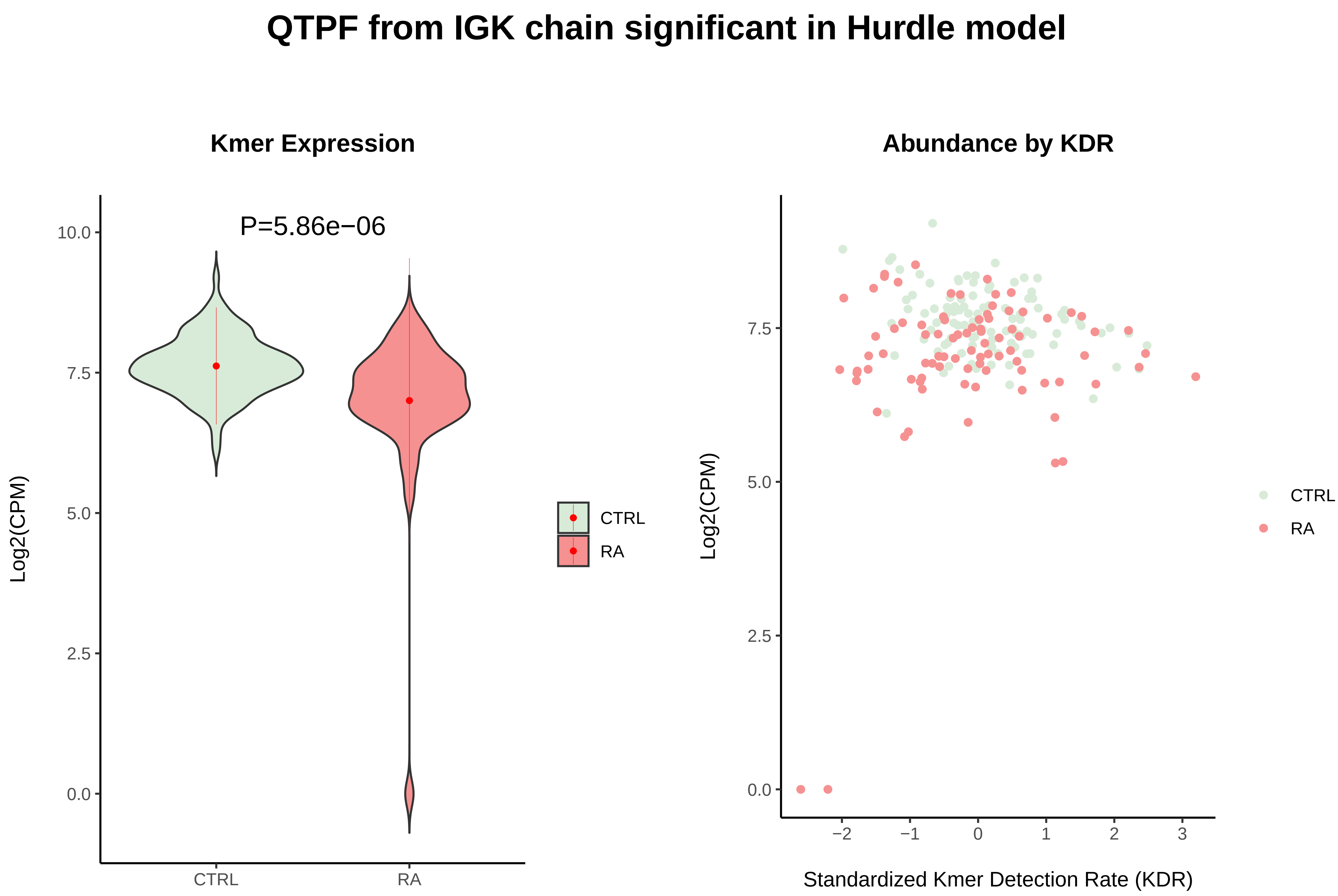

QTPF from IGK chain significant in Hurdle model
Kmer Expression
P=5.86e−06
Log2(CPM)
10.0
7.5
5.0
2.5
0.0
CTRL
RA
CTRL
RA
Abundance by KDR
Log2(CPM)
7.5
5.0
2.5
0.0
−2
−1
0
1
2
3
Standardized Kmer Detection Rate (KDR)
CTRL
RA

QTPG from IGK chain significant in Hurdle model

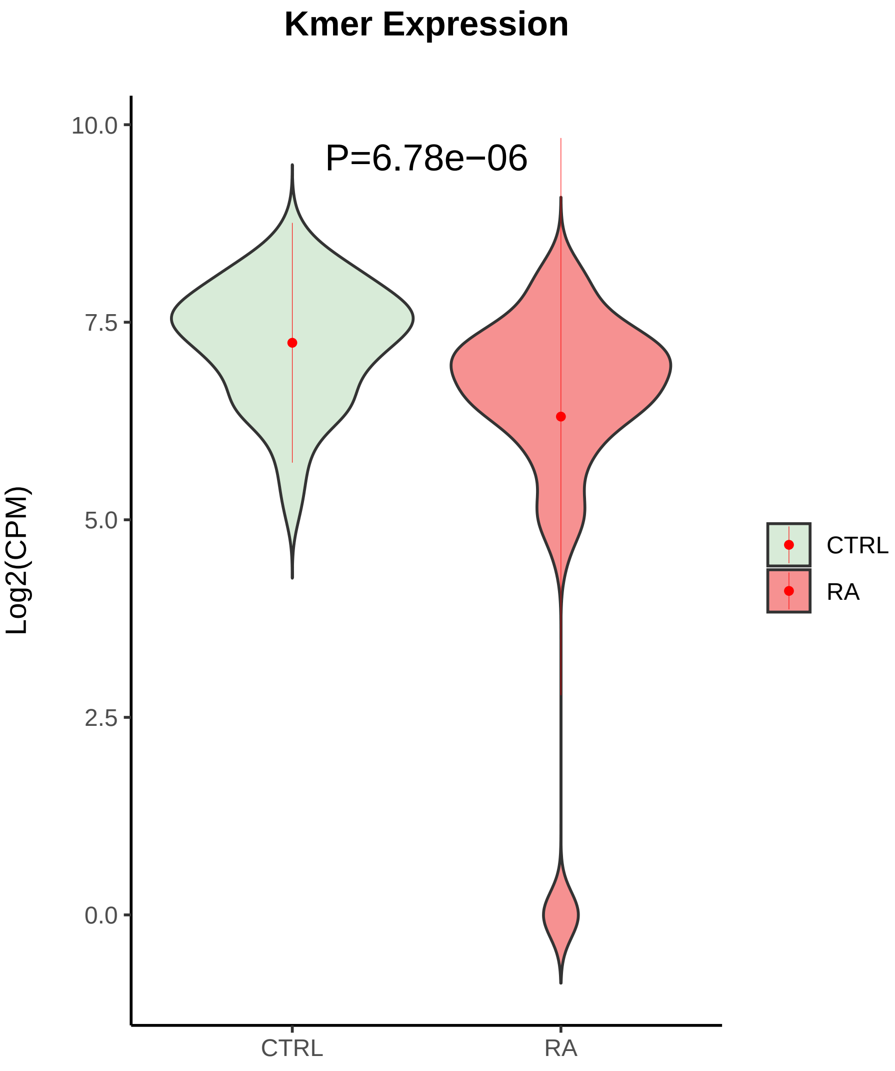

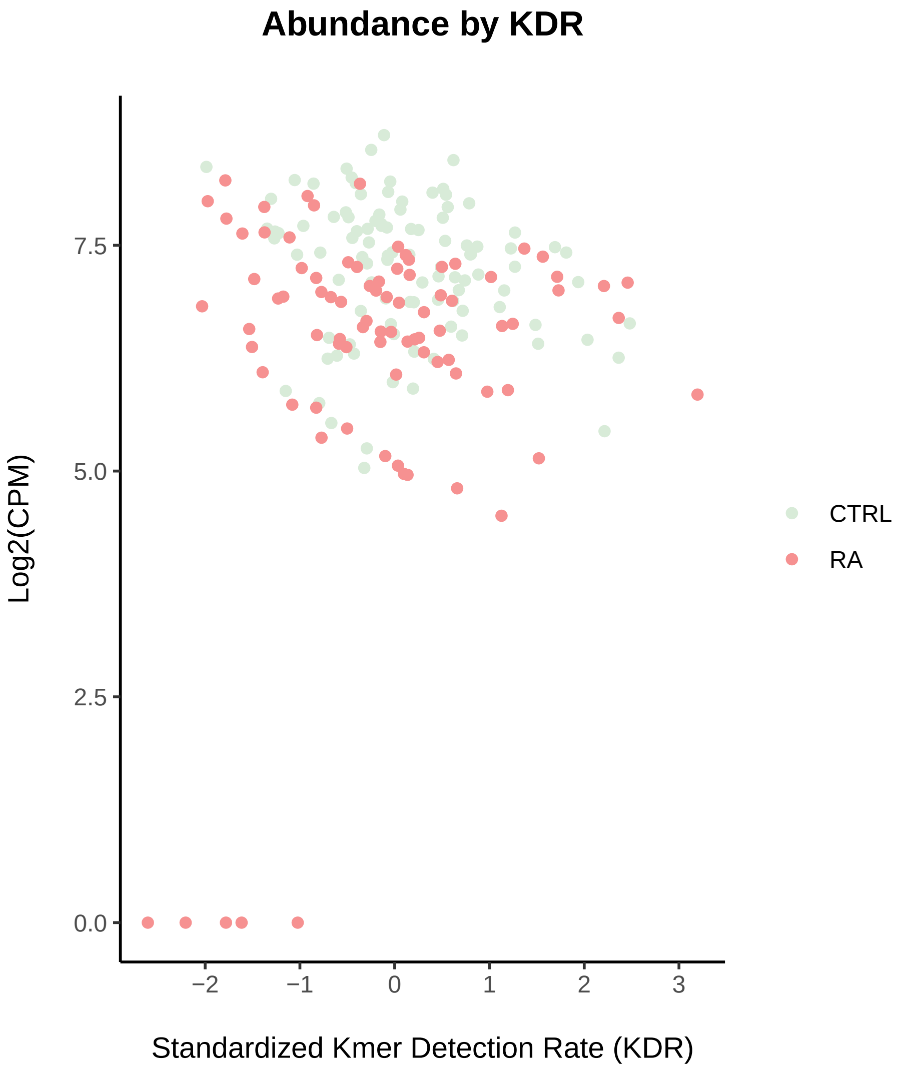

QTPL from IGK chain significant in Hurdle model

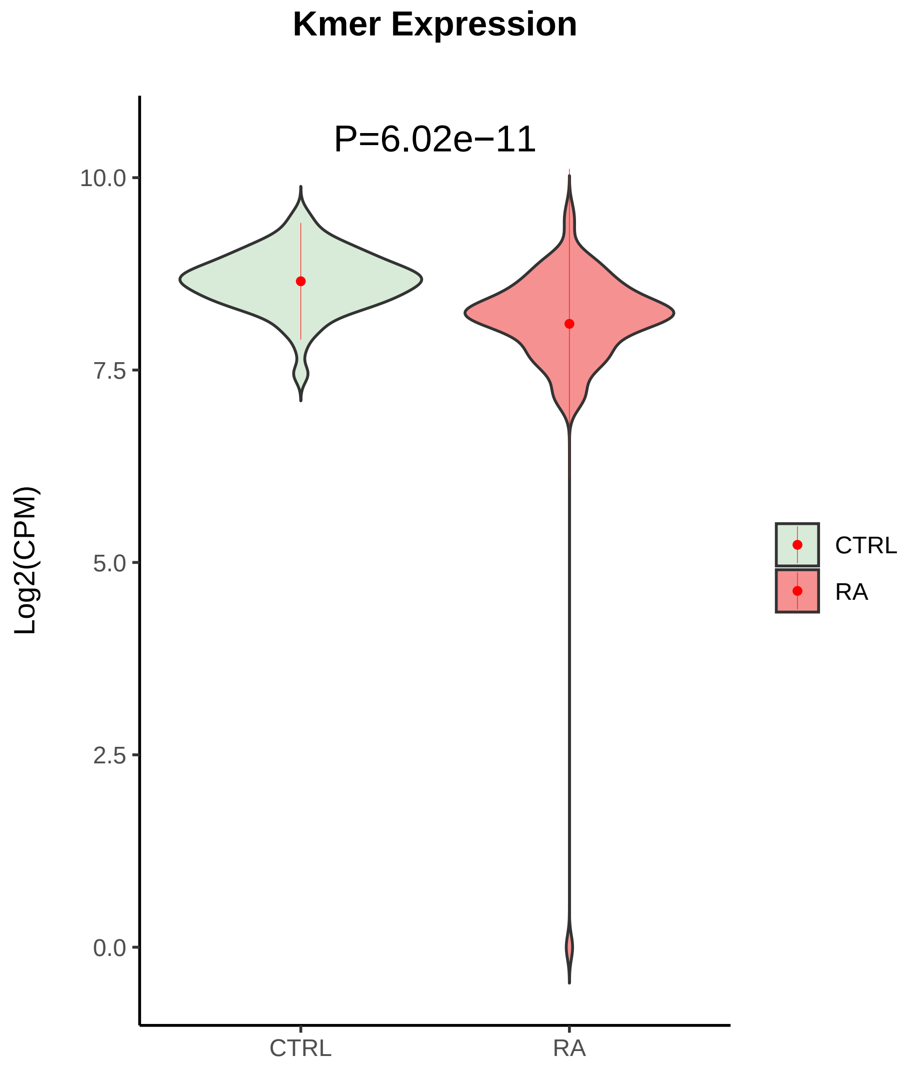

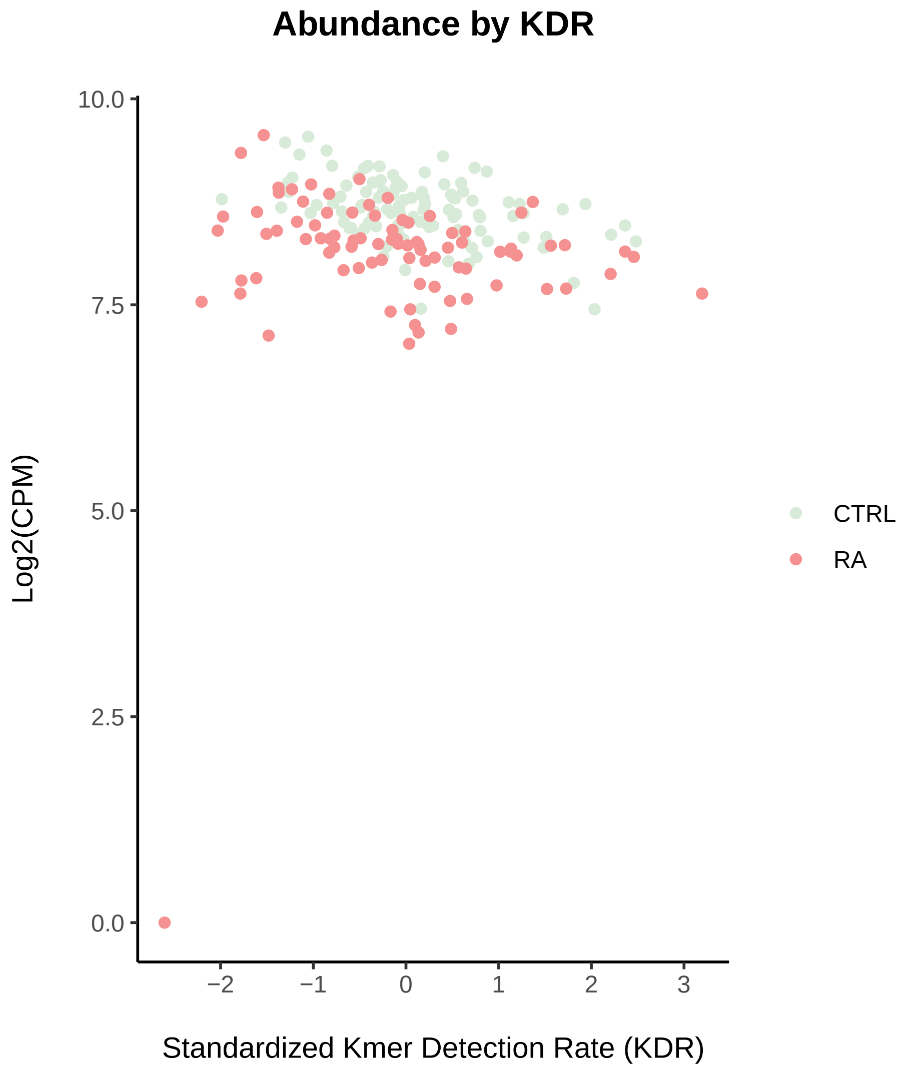

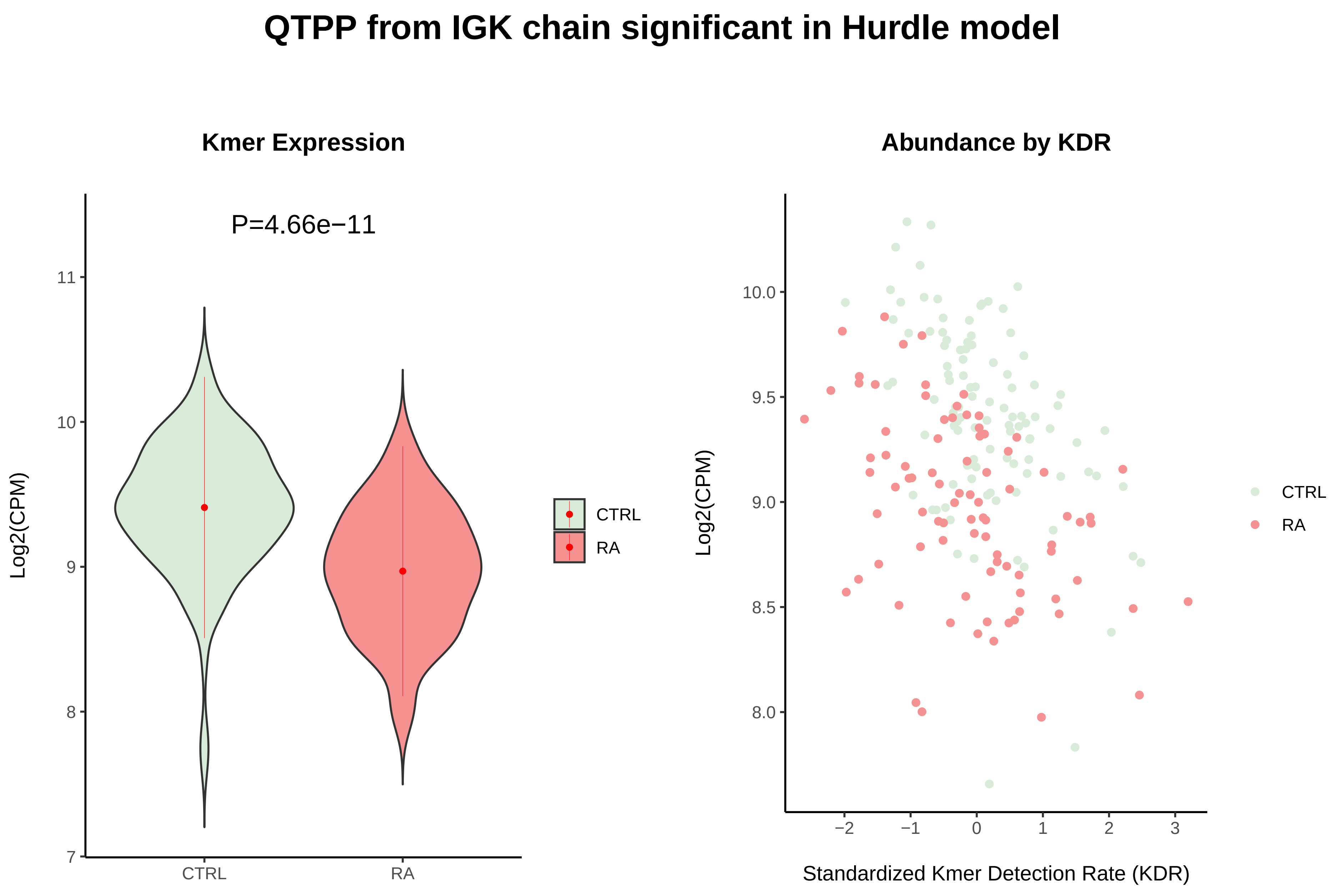
QTPP from IGK chain significant in Hurdle model
Kmer Expression
P=4.66e−11
Log2(CPM)
11
10
9
8
7
CTRL
RA
CTRL
RA
Abundance by KDR
Log2(CPM)
10.0
9.5
9.0
8.5
8.0
−2
−1
0
1
2
3
Standardized Kmer Detection Rate (KDR)
CTRL
RA

QTPQ from IGK chain significant in Hurdle model

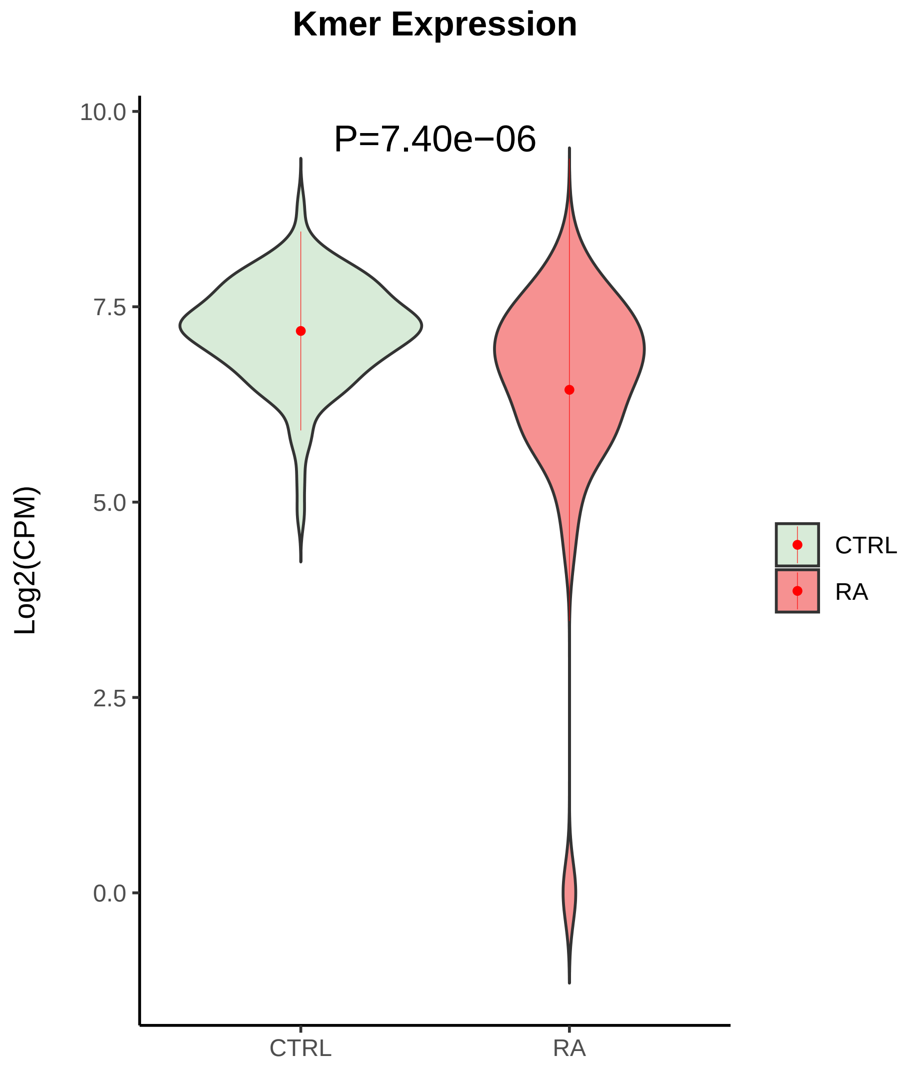

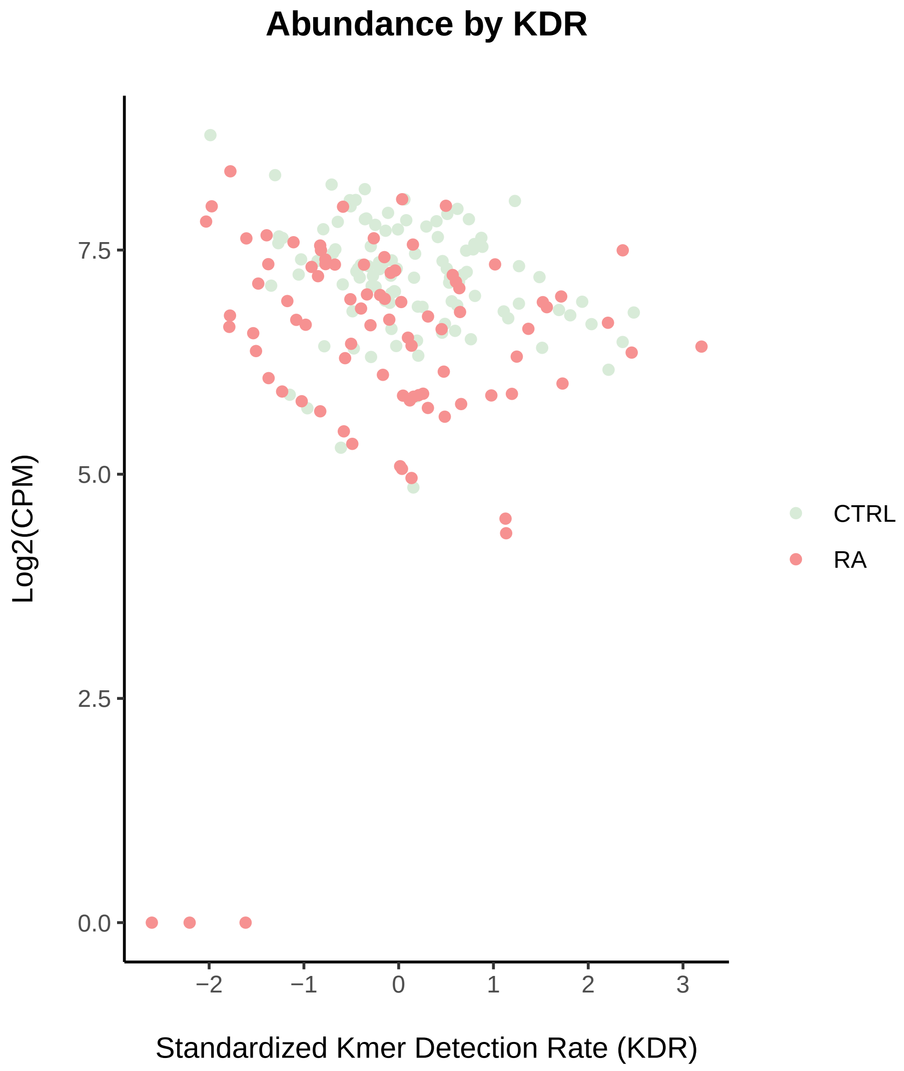

QTPR from IGK chain significant in Hurdle model

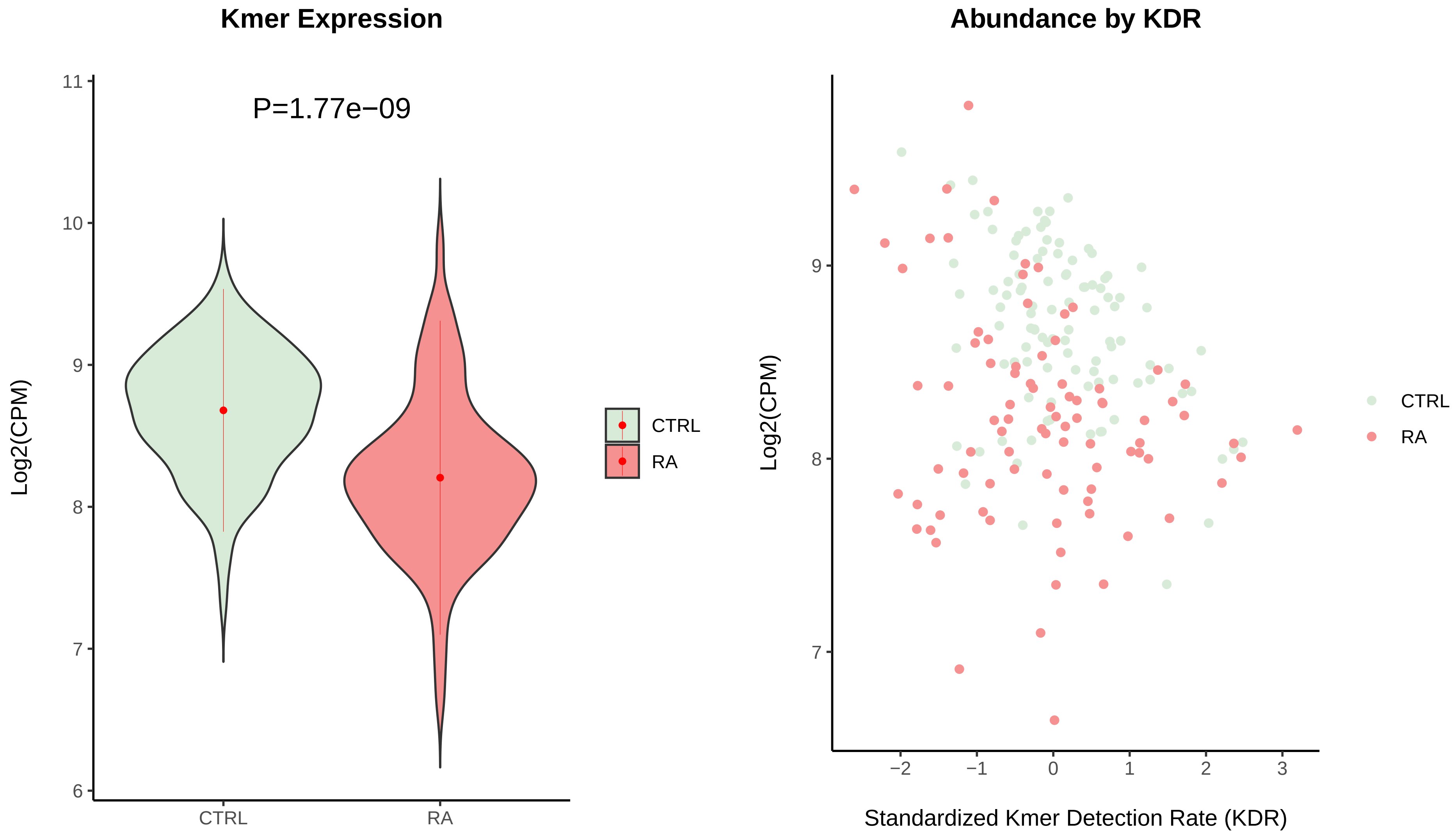

QVLQ from IGK chain significant in Hurdle model

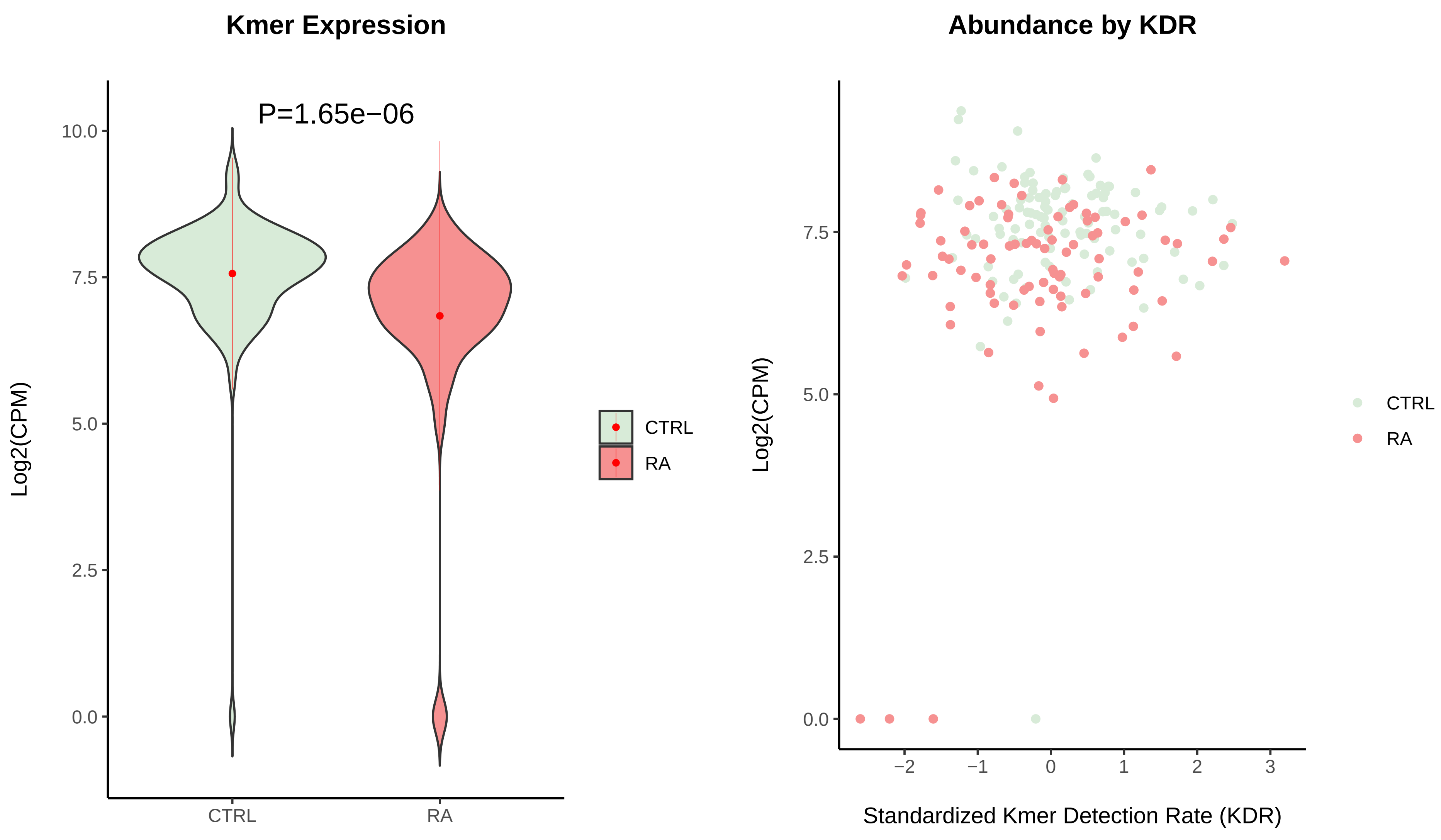

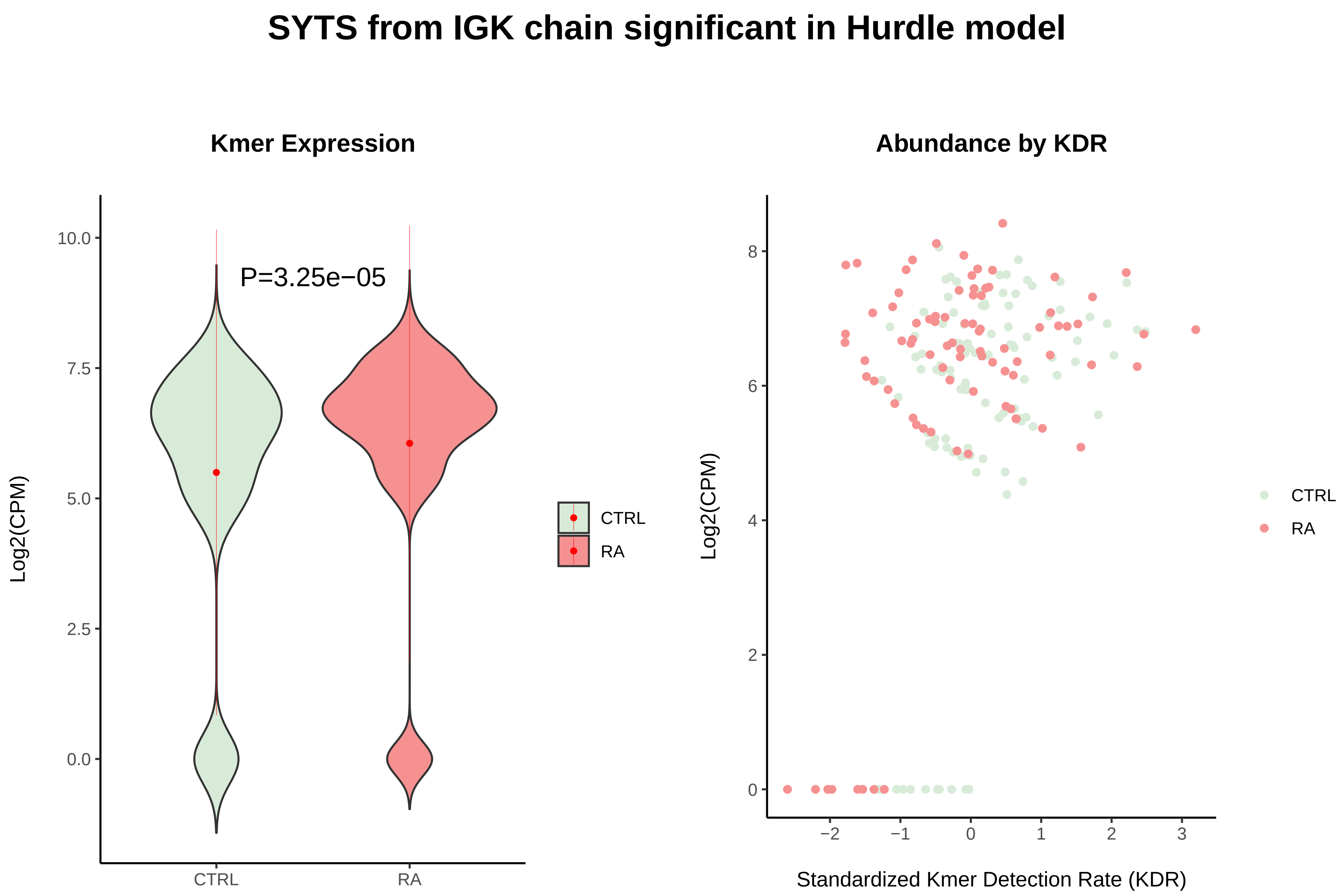

SYTS from IGK chain significant in Hurdle model
Kmer Expression
P=3.25e−05
Log2(CPM)
10.0
7.5
5.0
2.5
0.0
CTRL
RA
CTRL
RA
Abundance by KDR
Log2(CPM)
8
6
4
2
0
−2
−1
0
1
2
3
Standardized Kmer Detection Rate (KDR)
CTRL
RA

THWP from IGK chain significant in Hurdle model

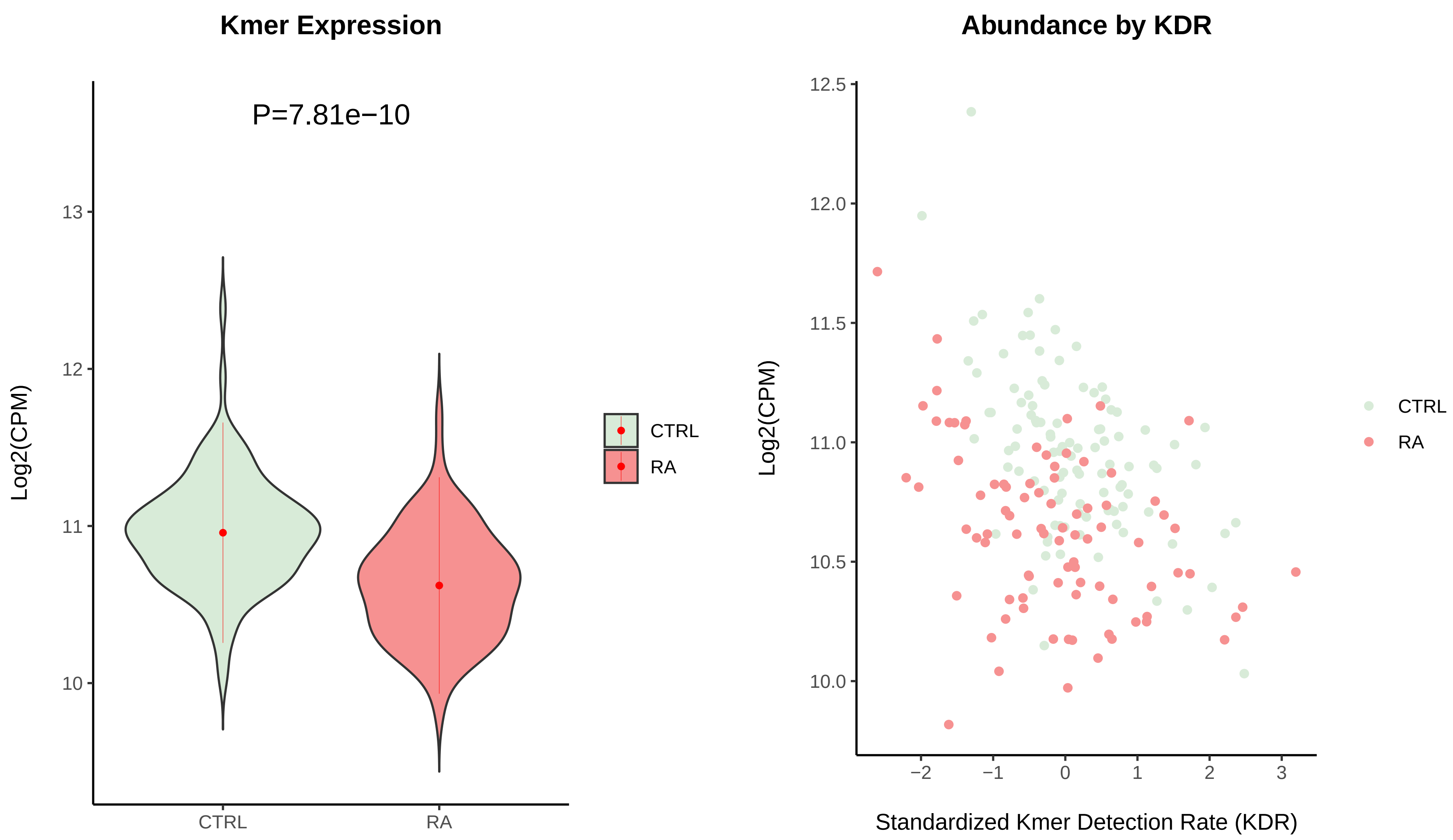

TQFP from IGK chain significant in Hurdle model

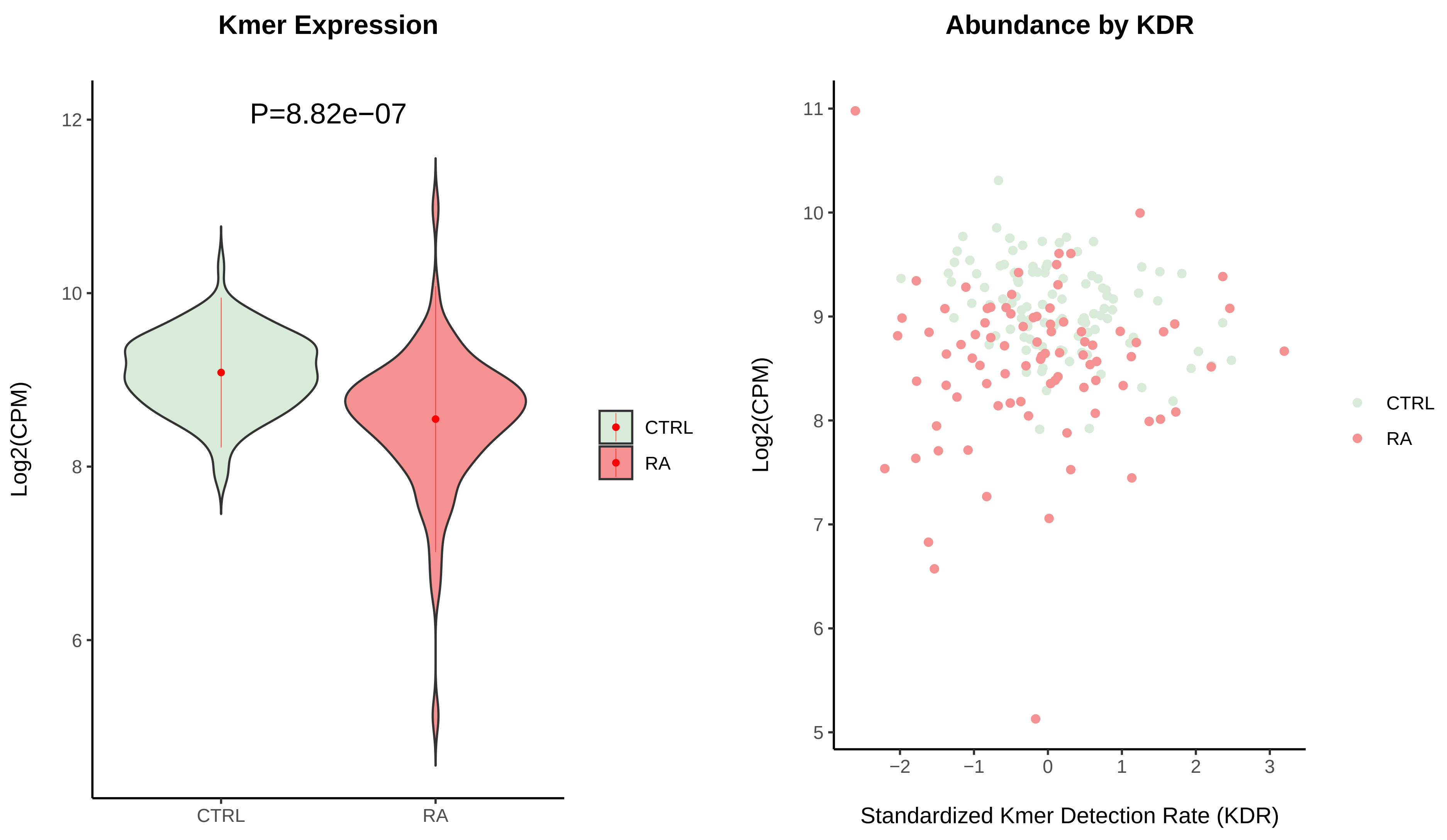

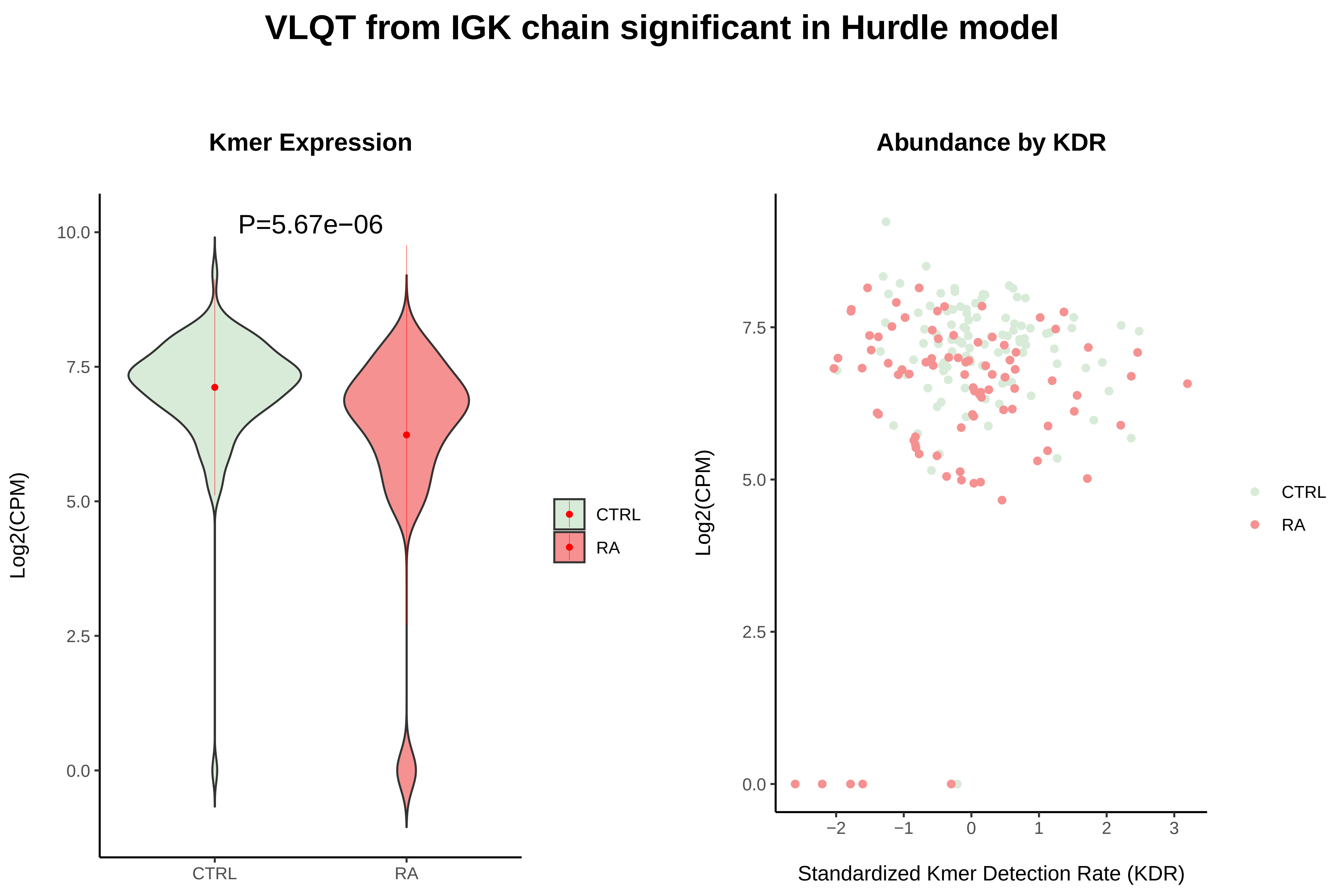
VLQT from IGK chain significant in Hurdle model
Kmer Expression
P=5.67e−06
Log2(CPM)
10.0
7.5
5.0
2.5
0.0
CTRL
RA
CTRL
RA
Abundance by KDR
Log2(CPM)
7.5
5.0
2.5
0.0
−2
−1
0
1
2
3
Standardized Kmer Detection Rate (KDR)
CTRL
RA

YKSS from IGK chain significant in Hurdle model

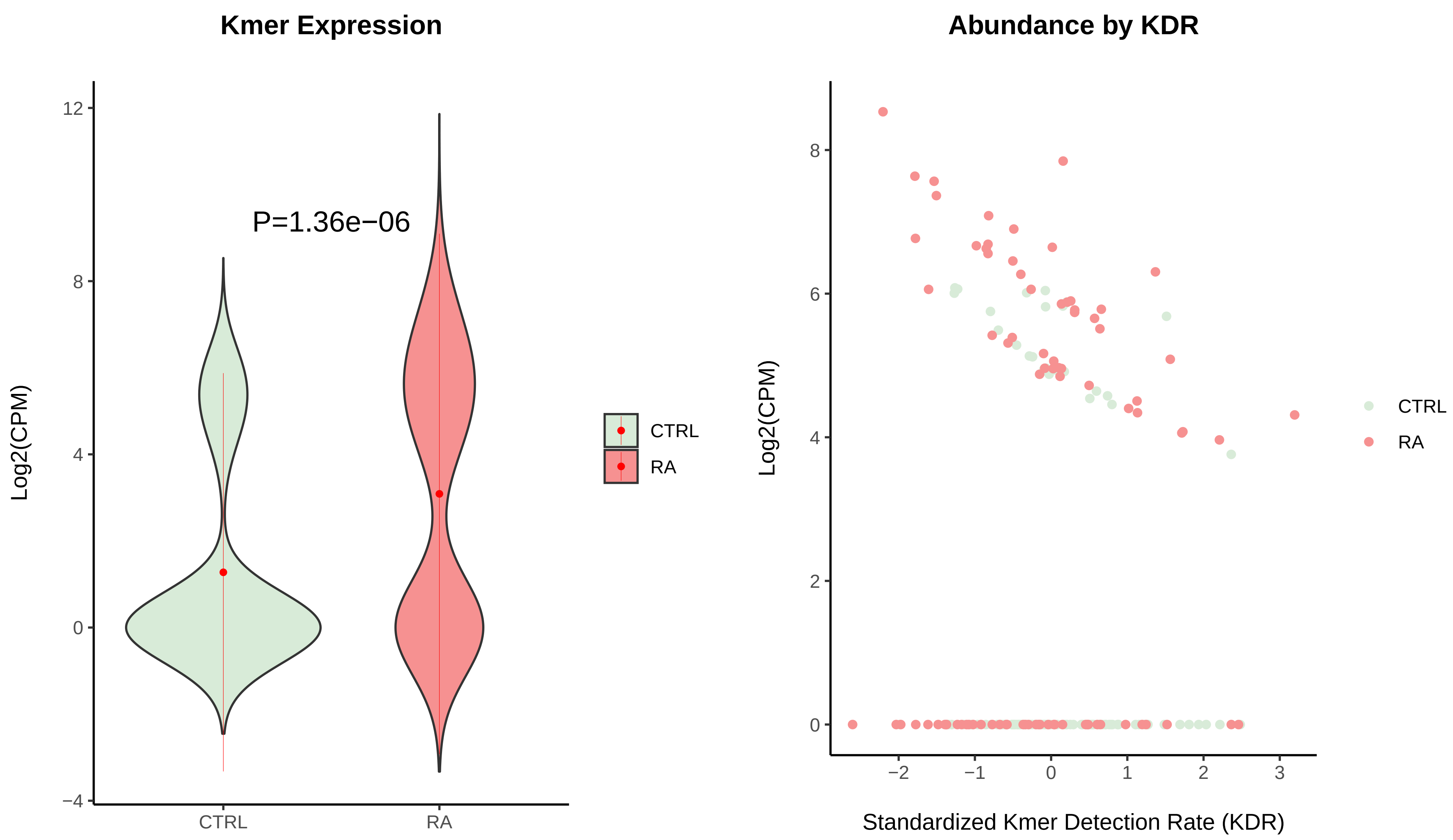

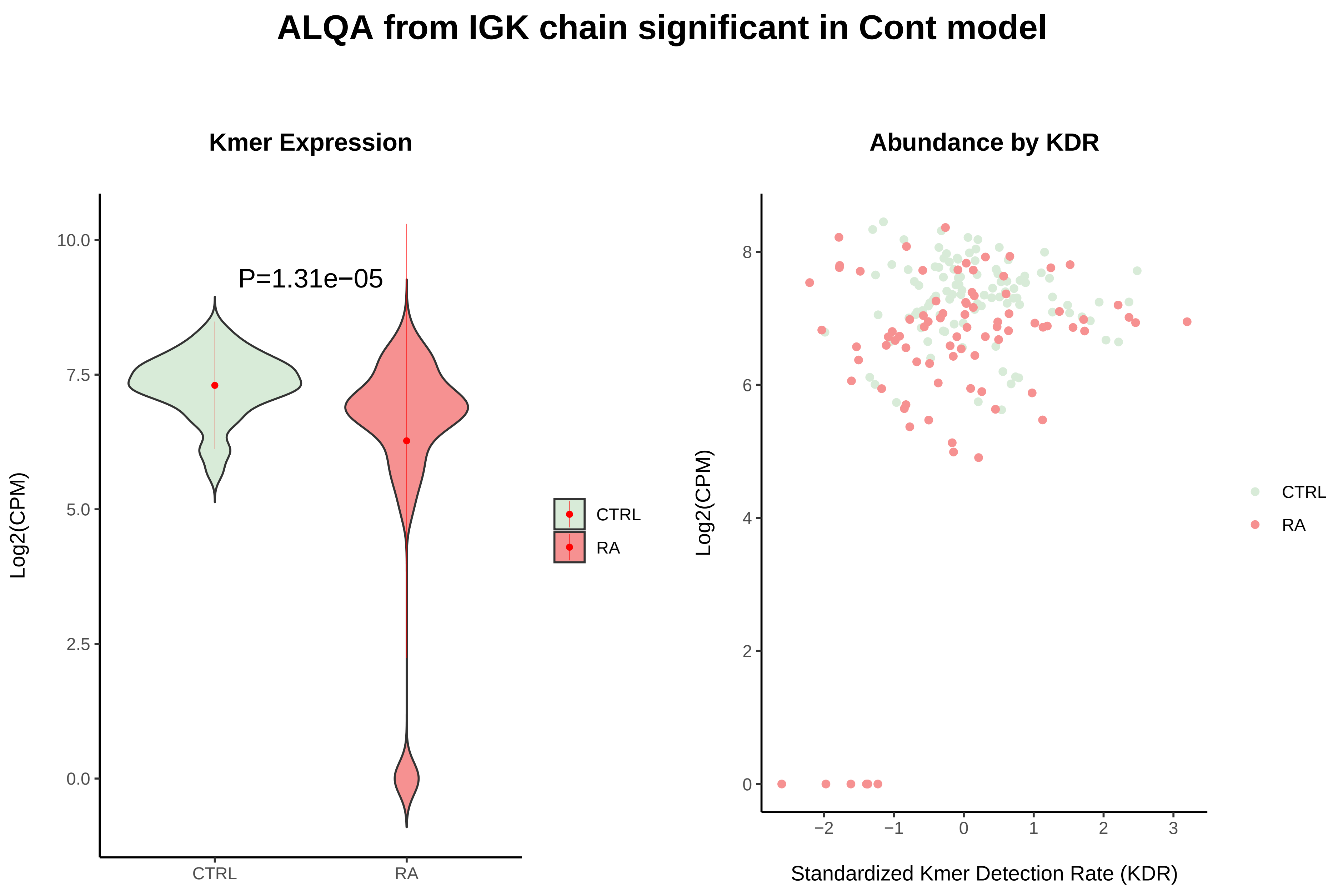

ALQA from IGK chain significant in Cont model
Kmer Expression
P=1.31e−05
Log2(CPM)
10.0
7.5
5.0
2.5
0.0
CTRL
RA
CTRL
RA
Abundance by KDR
Log2(CPM)
8
6
4
2
0
−2
−1
0
1
2
3
Standardized Kmer Detection Rate (KDR)
CTRL
RA

ALQI from IGK chain significant in Cont model

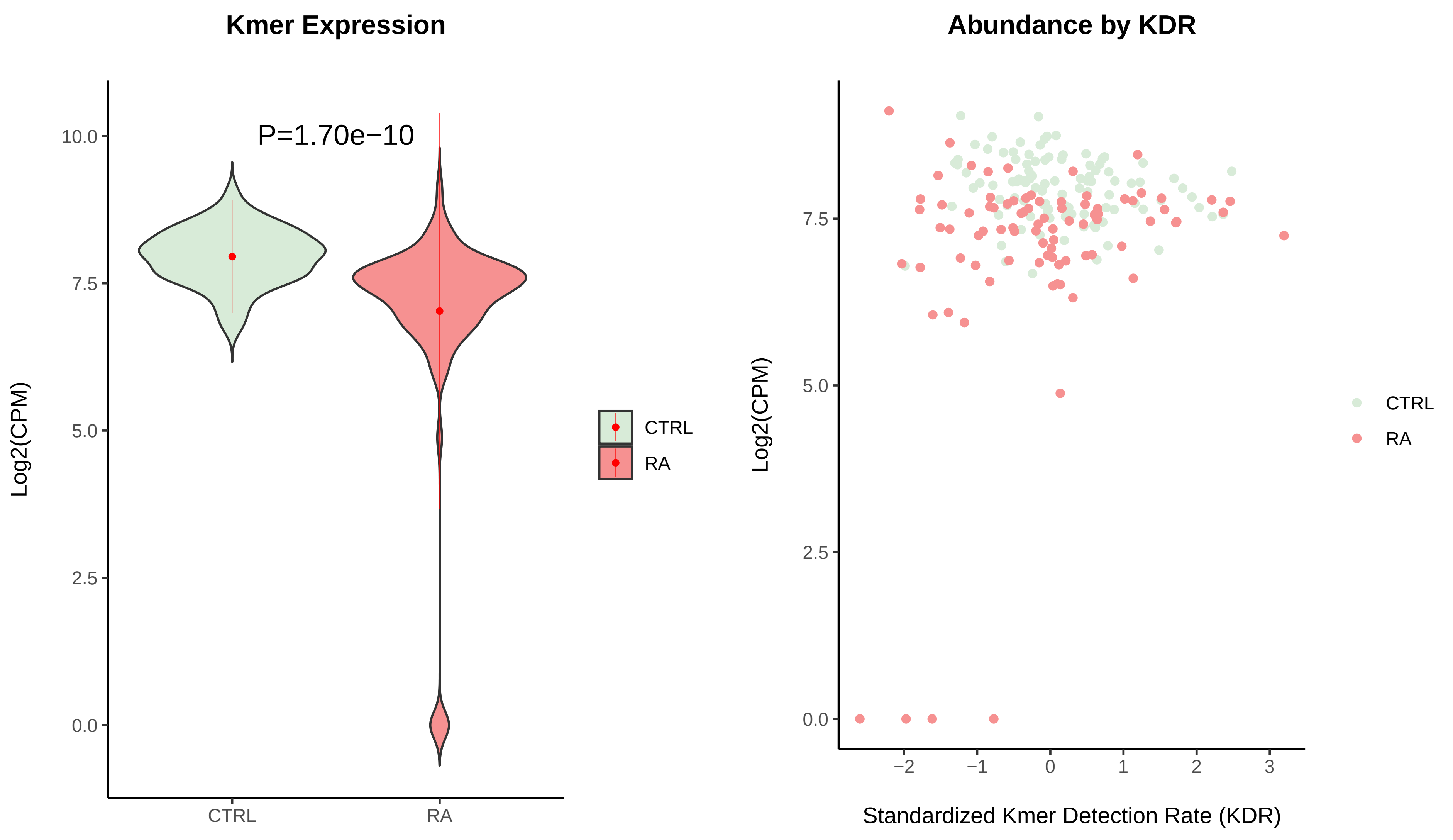

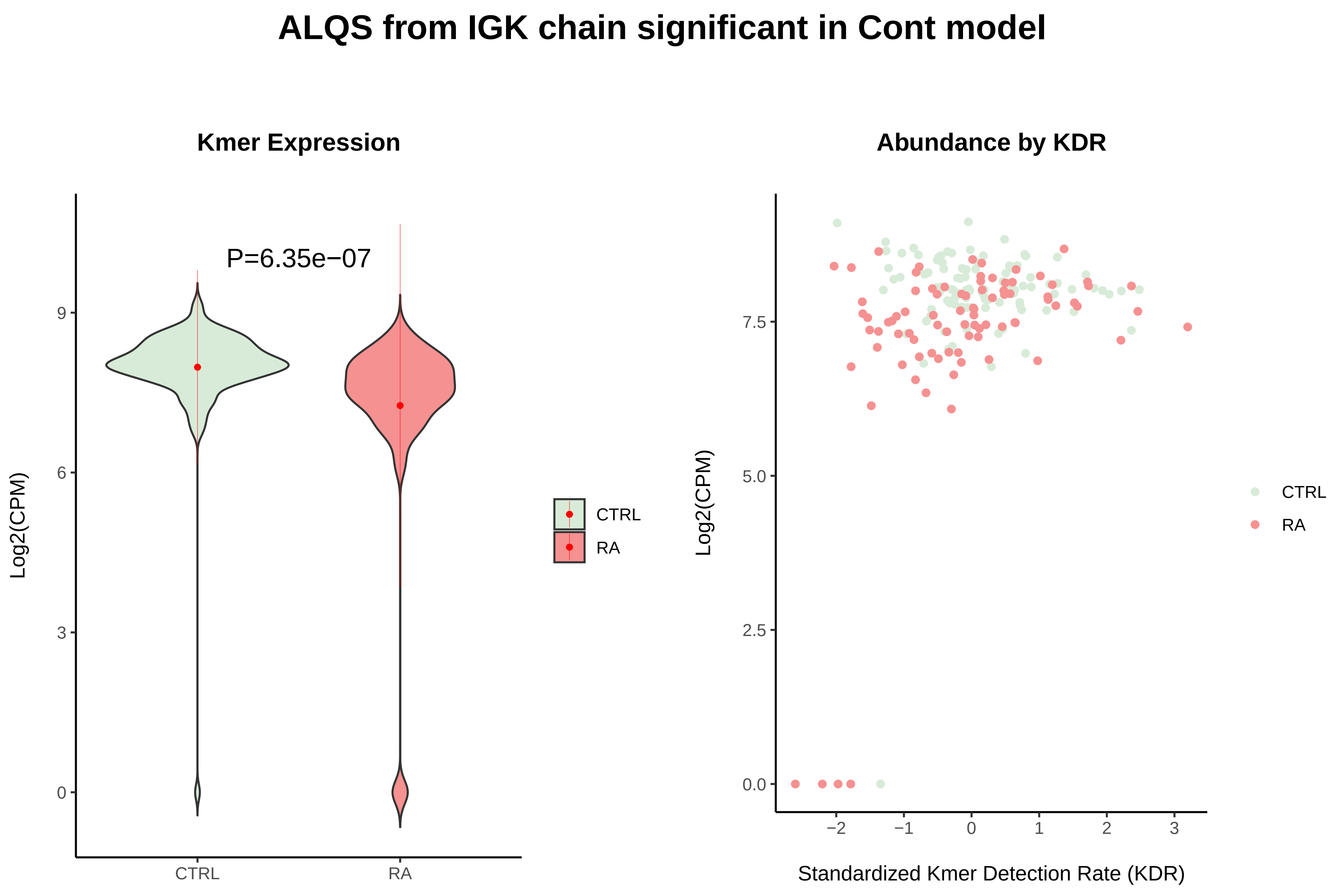
ALQS from IGK chain significant in Cont model
Kmer Expression
P=6.35e−07
Log2(CPM)
9
6
3
0
CTRL
RA
CTRL
RA
Abundance by KDR
Log2(CPM)
7.5
5.0
2.5
0.0
−2
−1
0
1
2
3
Standardized Kmer Detection Rate (KDR)
CTRL
RA

ALQT from IGK chain significant in Cont model

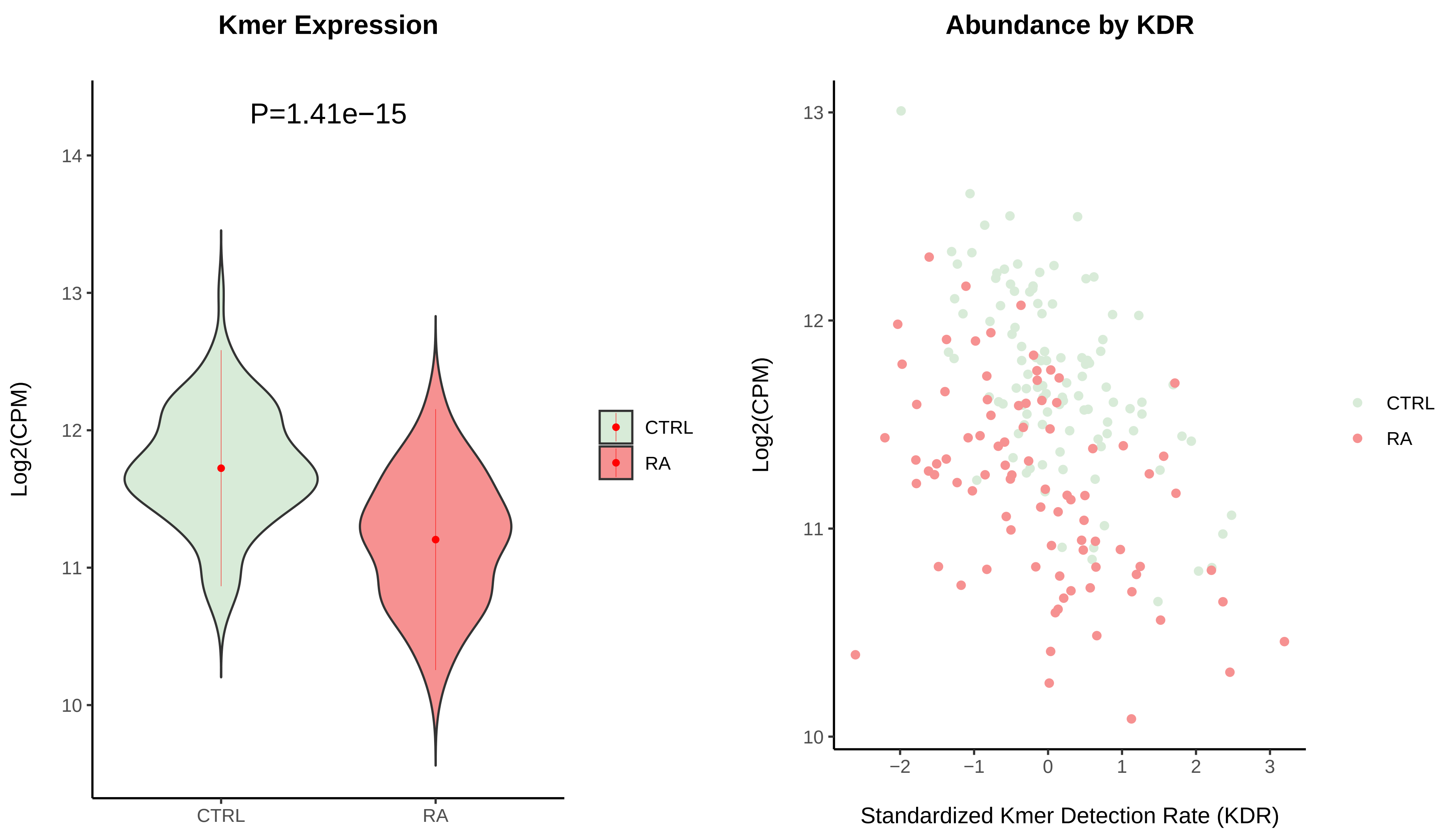

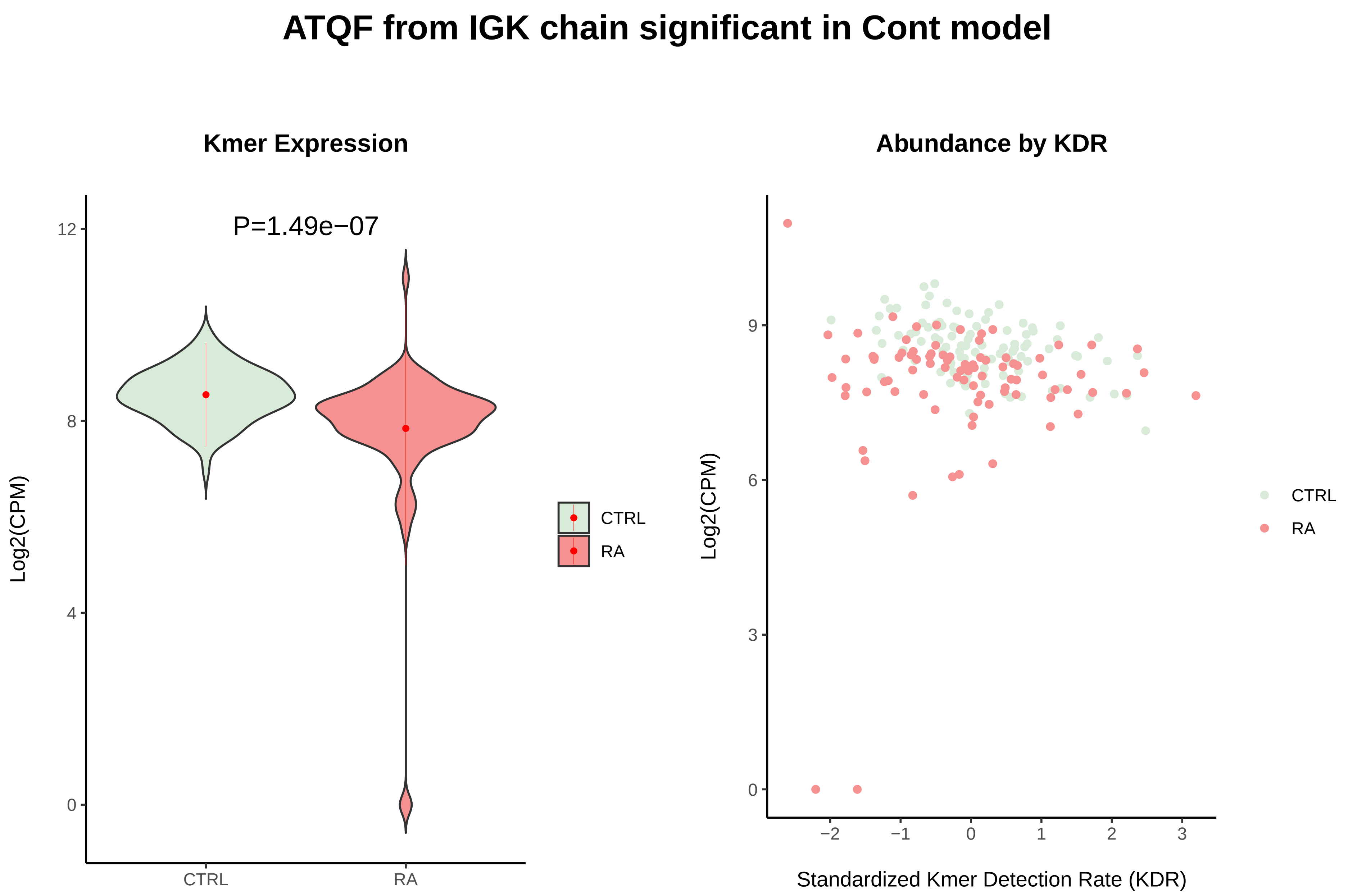
ATQF from IGK chain significant in Cont model
Kmer Expression
P=1.49e−07
Log2(CPM)
12
8
4
0
CTRL
RA
CTRL
RA
Abundance by KDR
Log2(CPM)
9
6
3
0
−2
−1
0
1
2
3
Standardized Kmer Detection Rate (KDR)
CTRL
RA

CMQA from IGK chain significant in Cont model

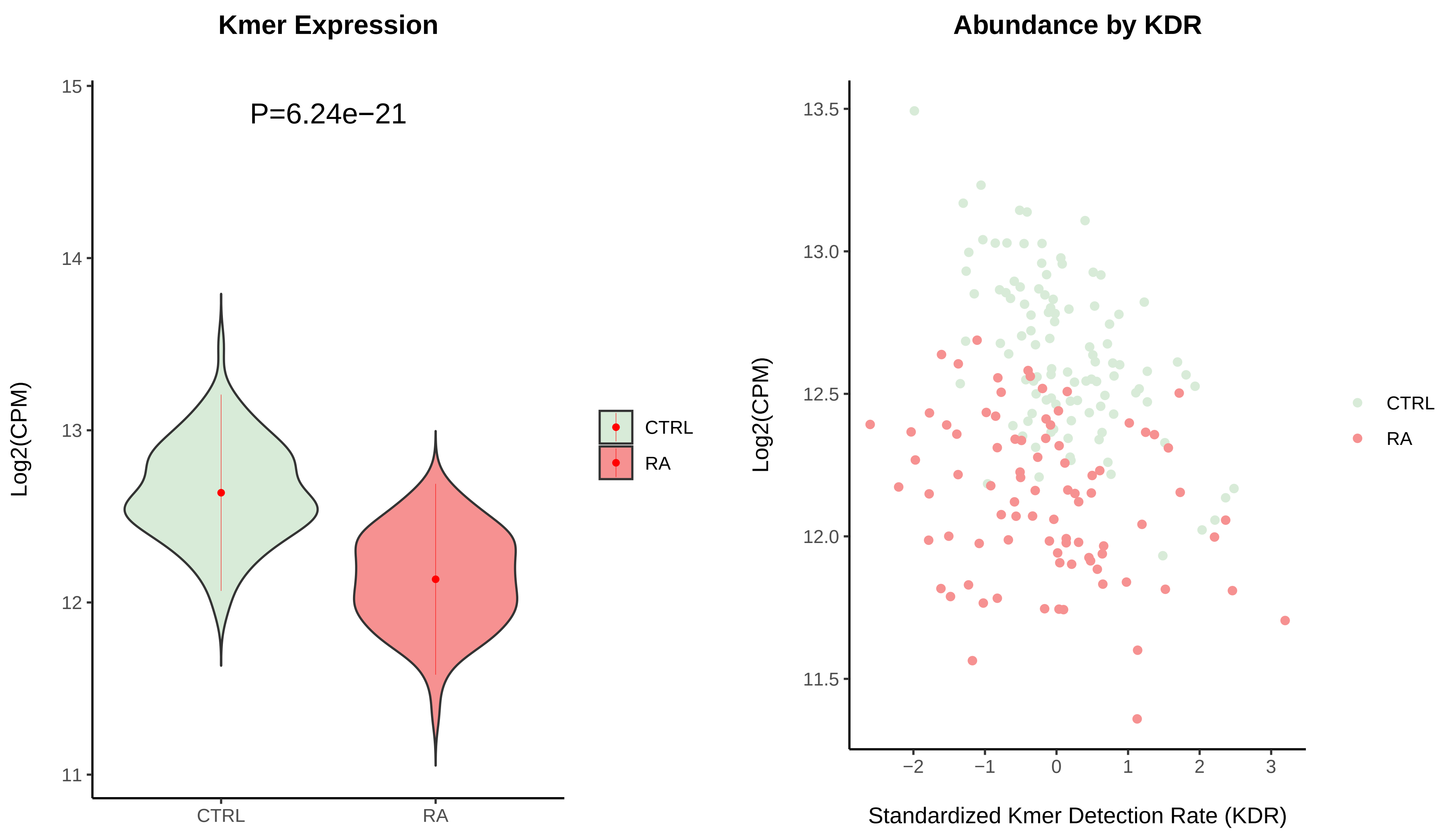

CMQG from IGK chain significant in Cont model

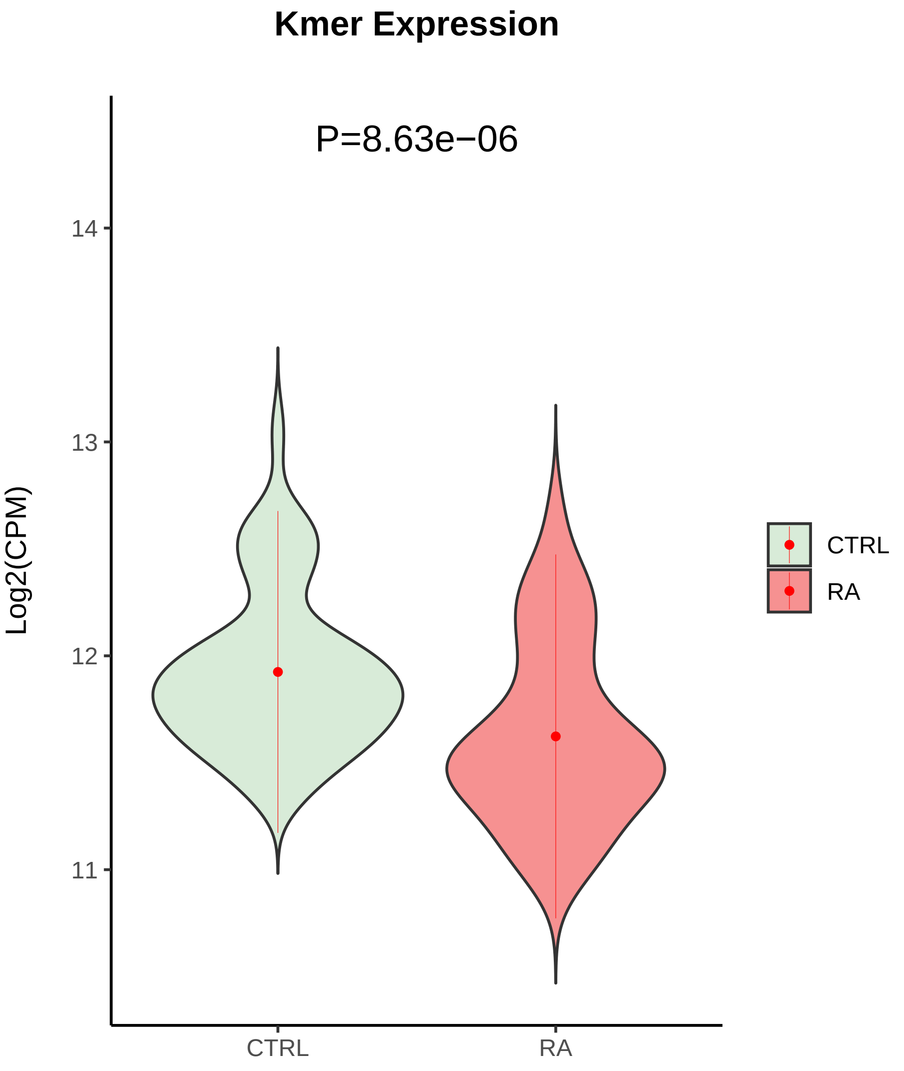

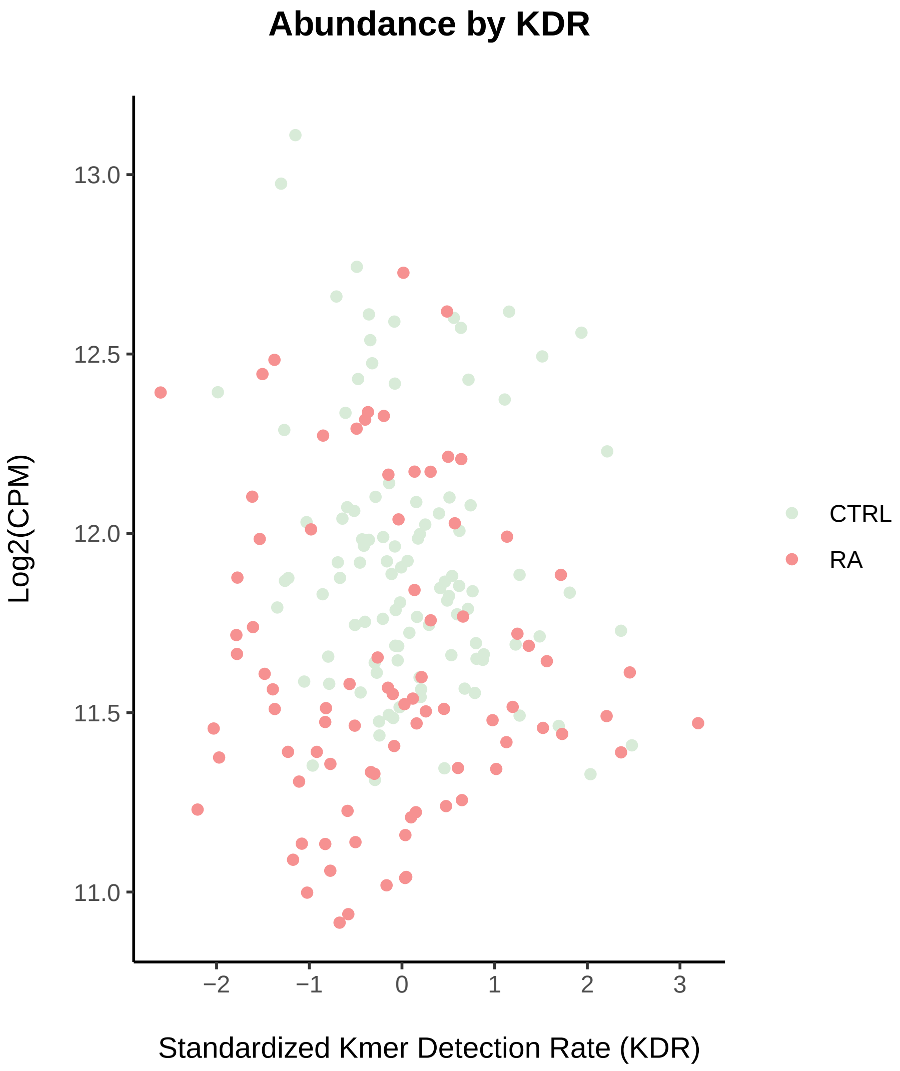

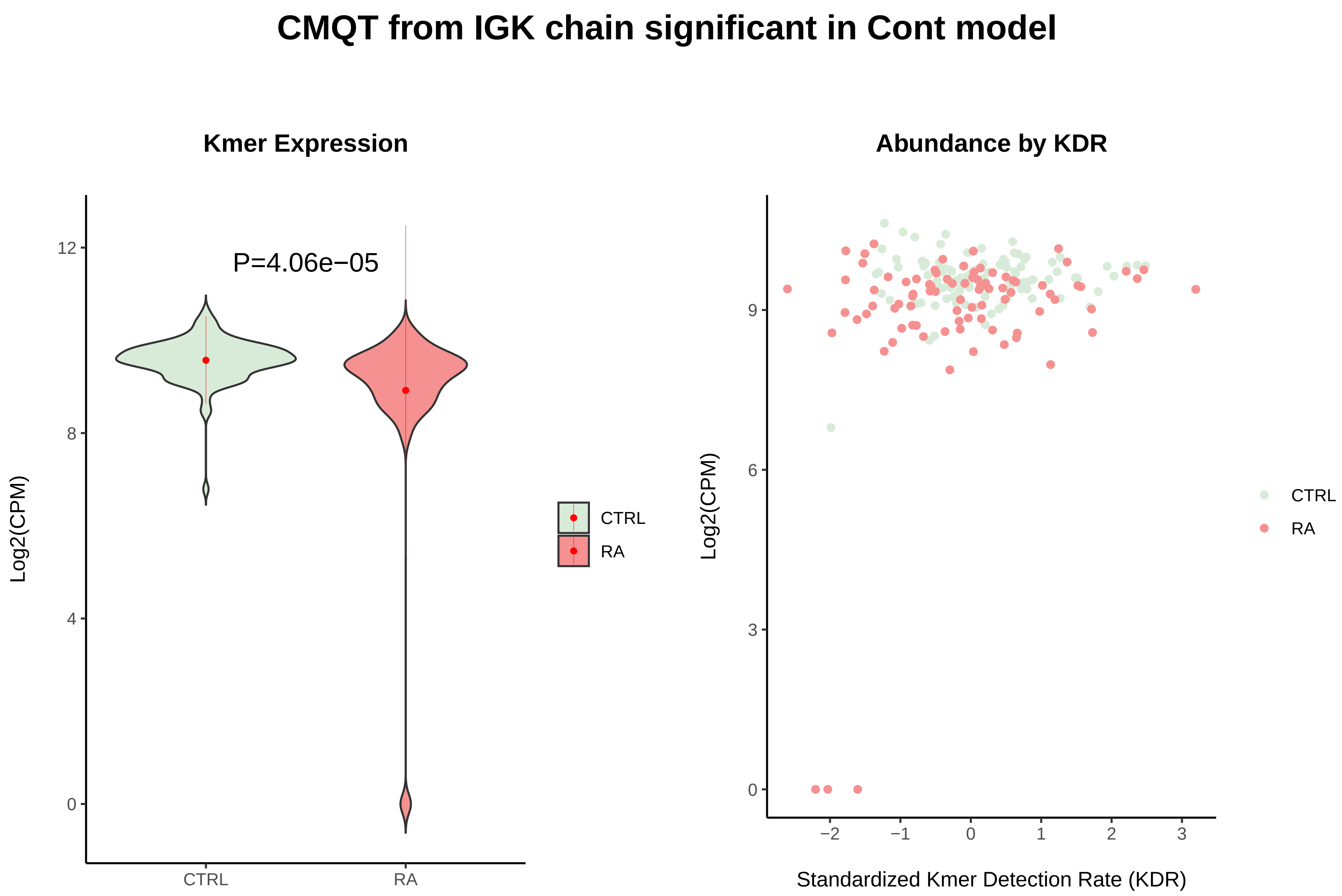
CMQT from IGK chain significant in Cont model
Kmer Expression
P=4.06e−05
Log2(CPM)
12
8
4
0
CTRL
RA
CTRL
RA
Abundance by KDR
Log2(CPM)
9
6
3
0
−2
−1
0
1
2
3
Standardized Kmer Detection Rate (KDR)
CTRL
RA

CMQV from IGK chain significant in Cont model

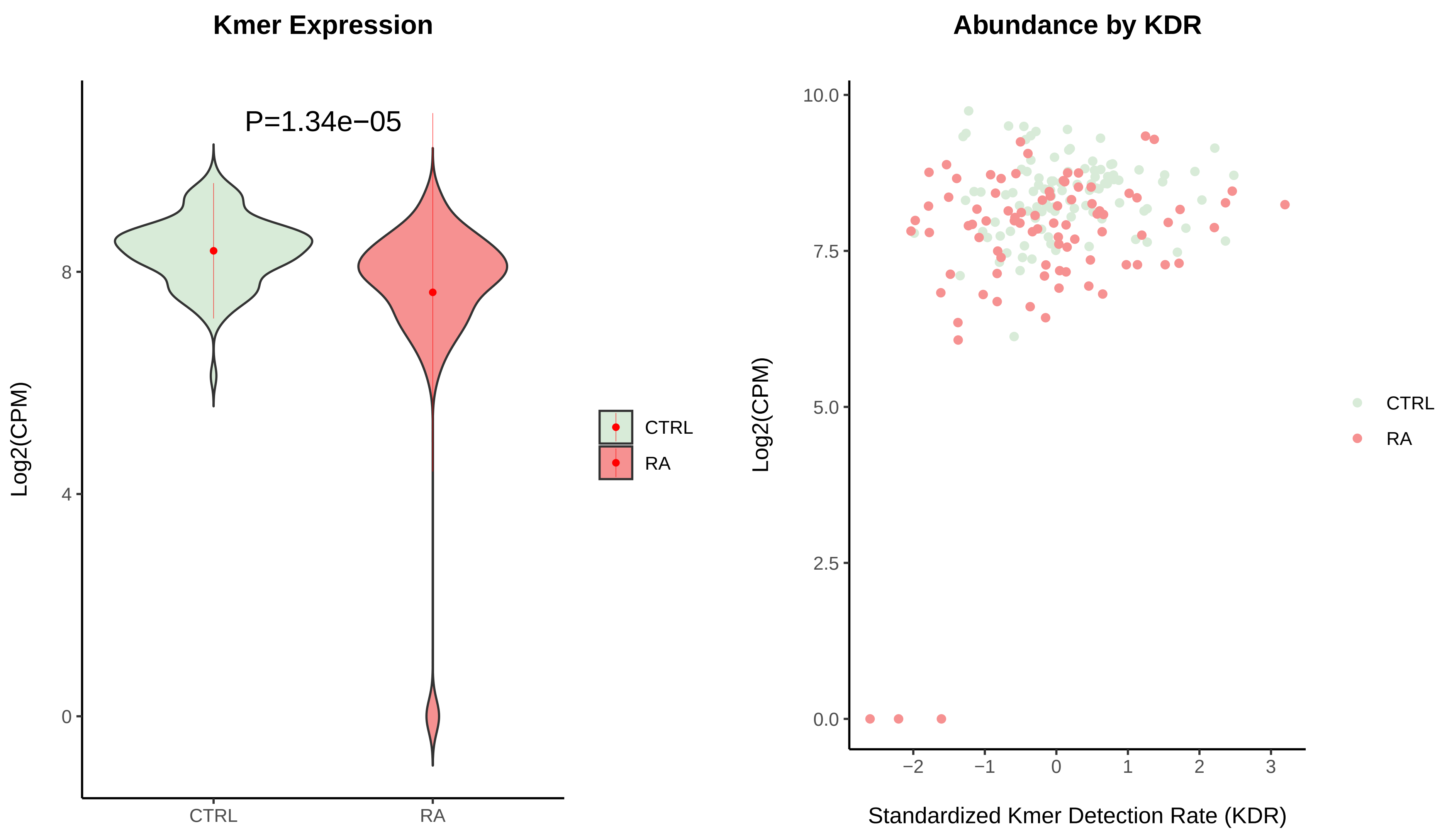

GTHW from IGK chain significant in Cont model

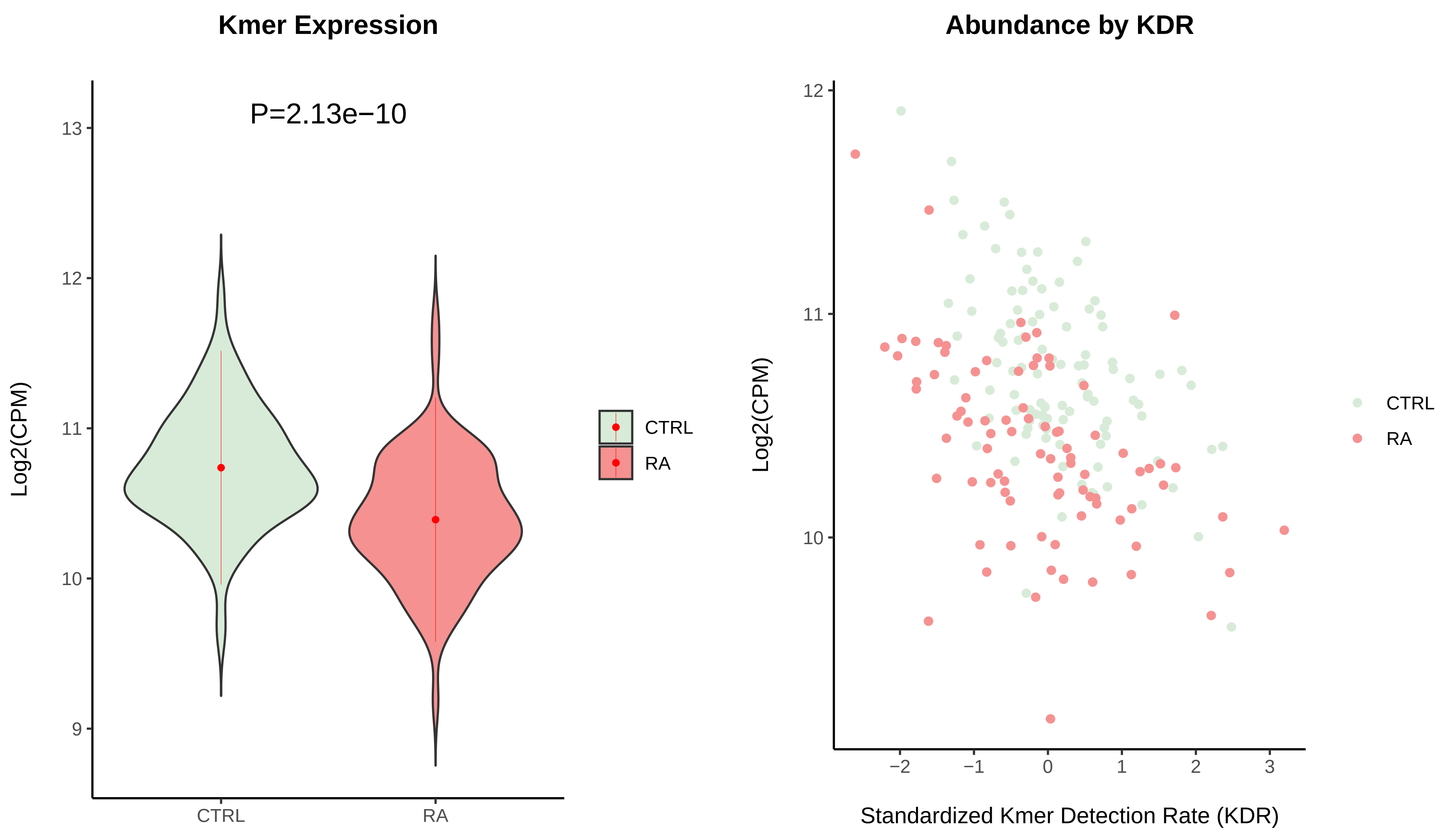

HWPP from IGK chain significant in Cont model

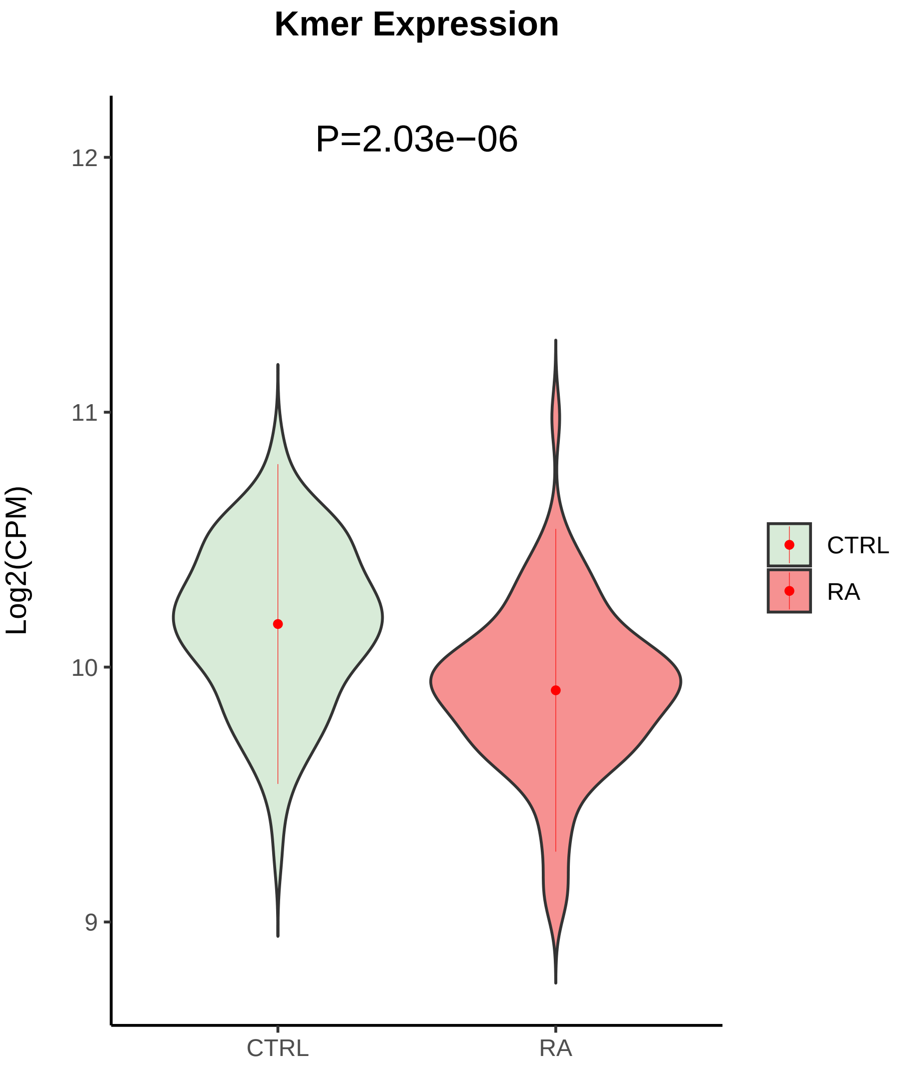

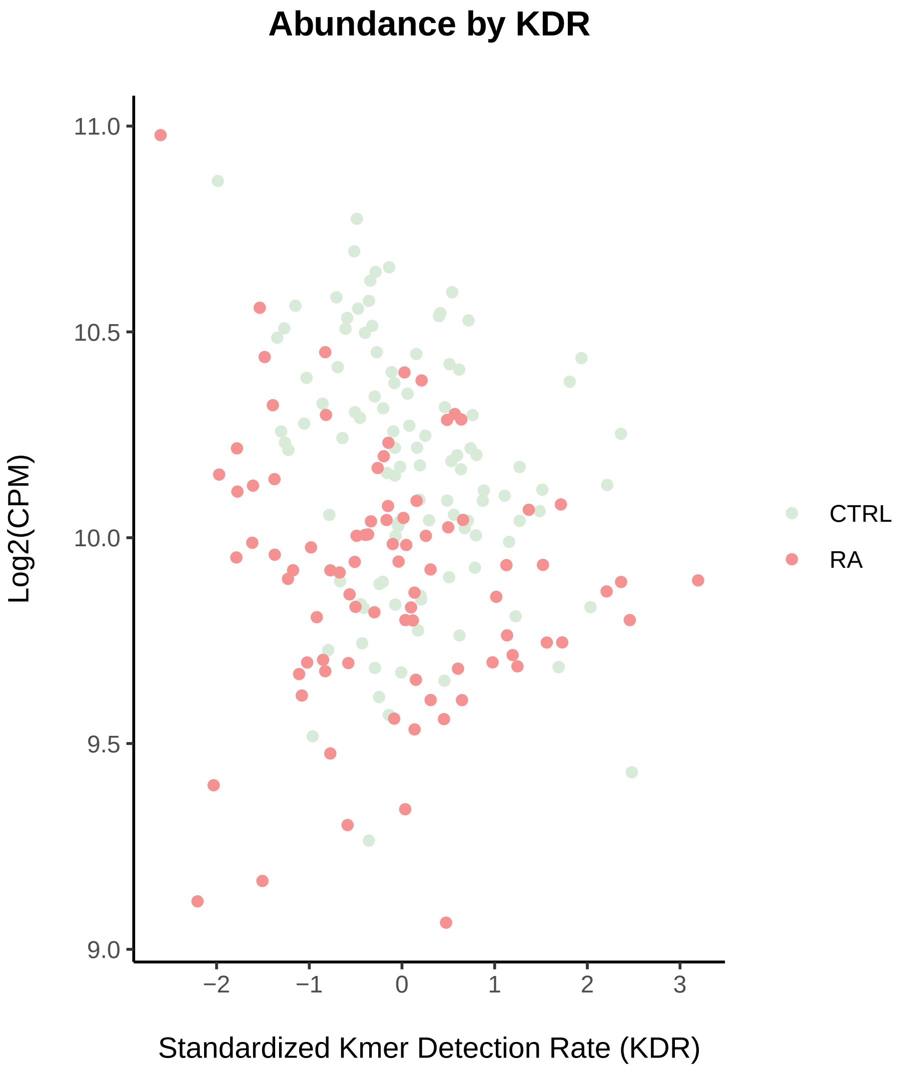

LQIP from IGK chain significant in Cont model

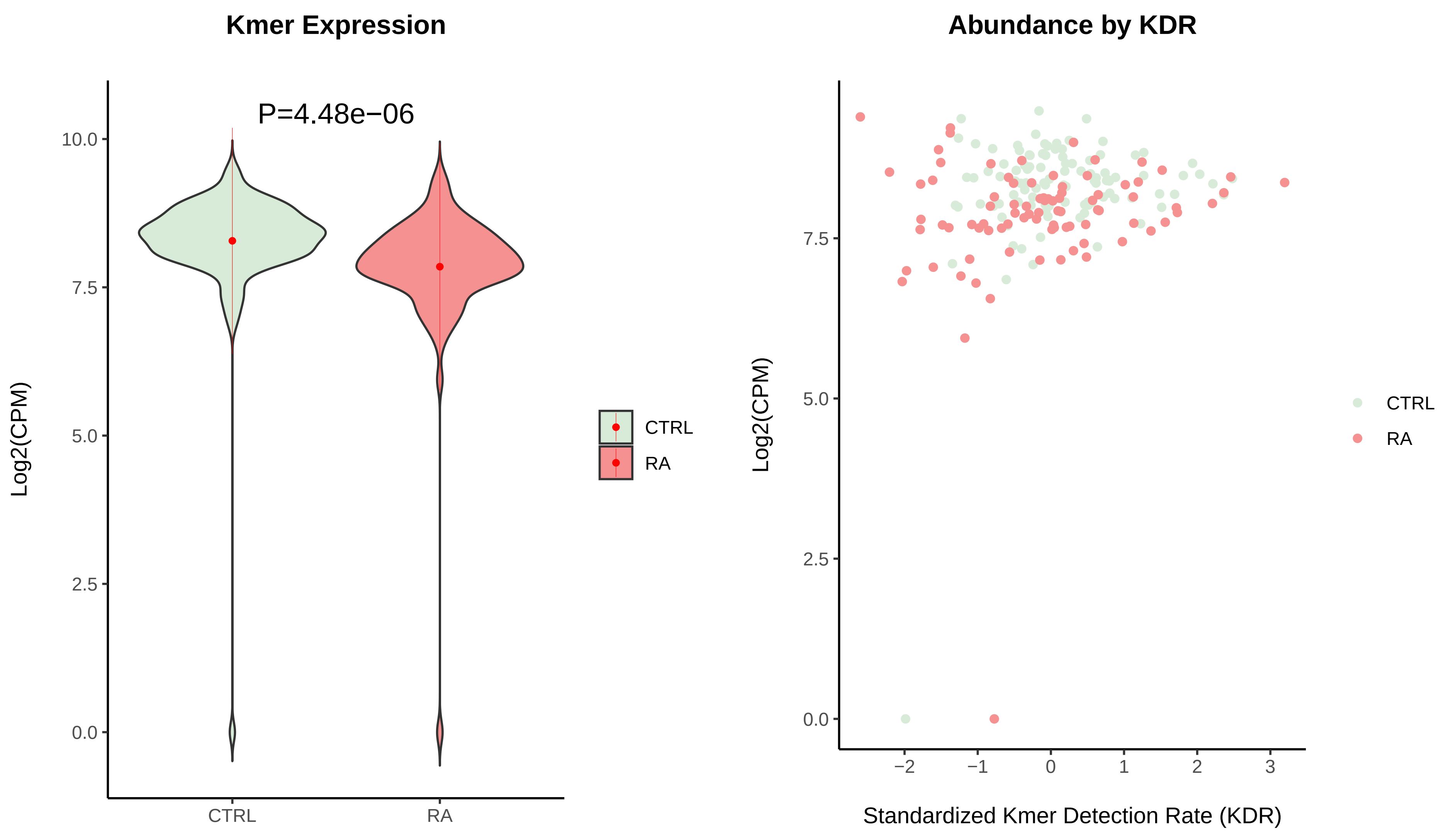

LQTL from IGK chain significant in Cont model

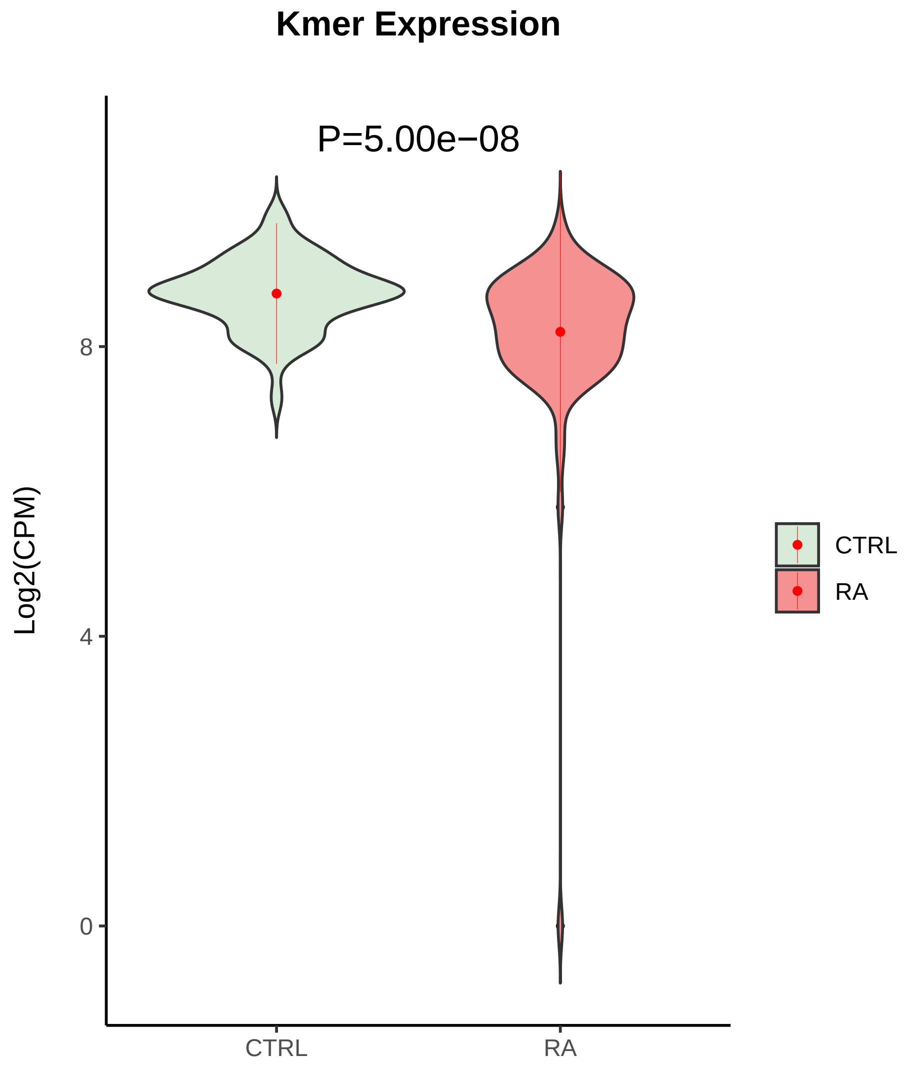

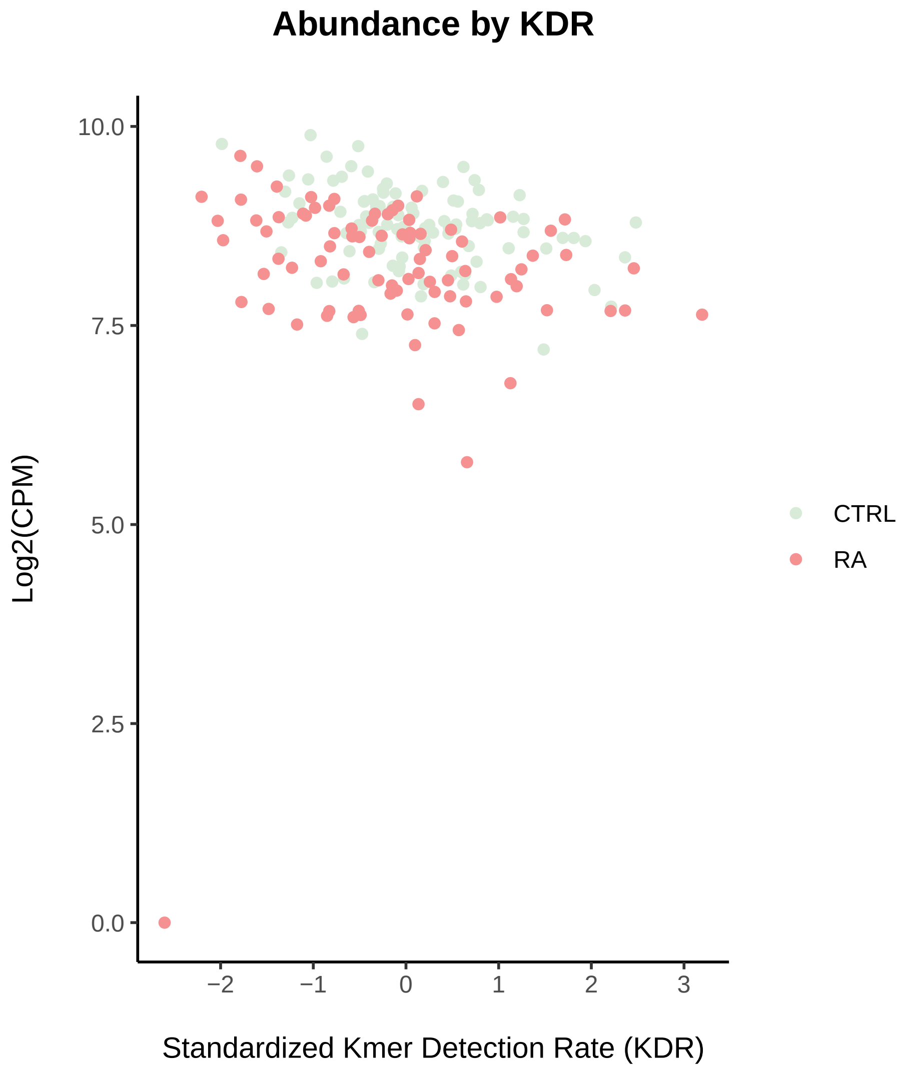

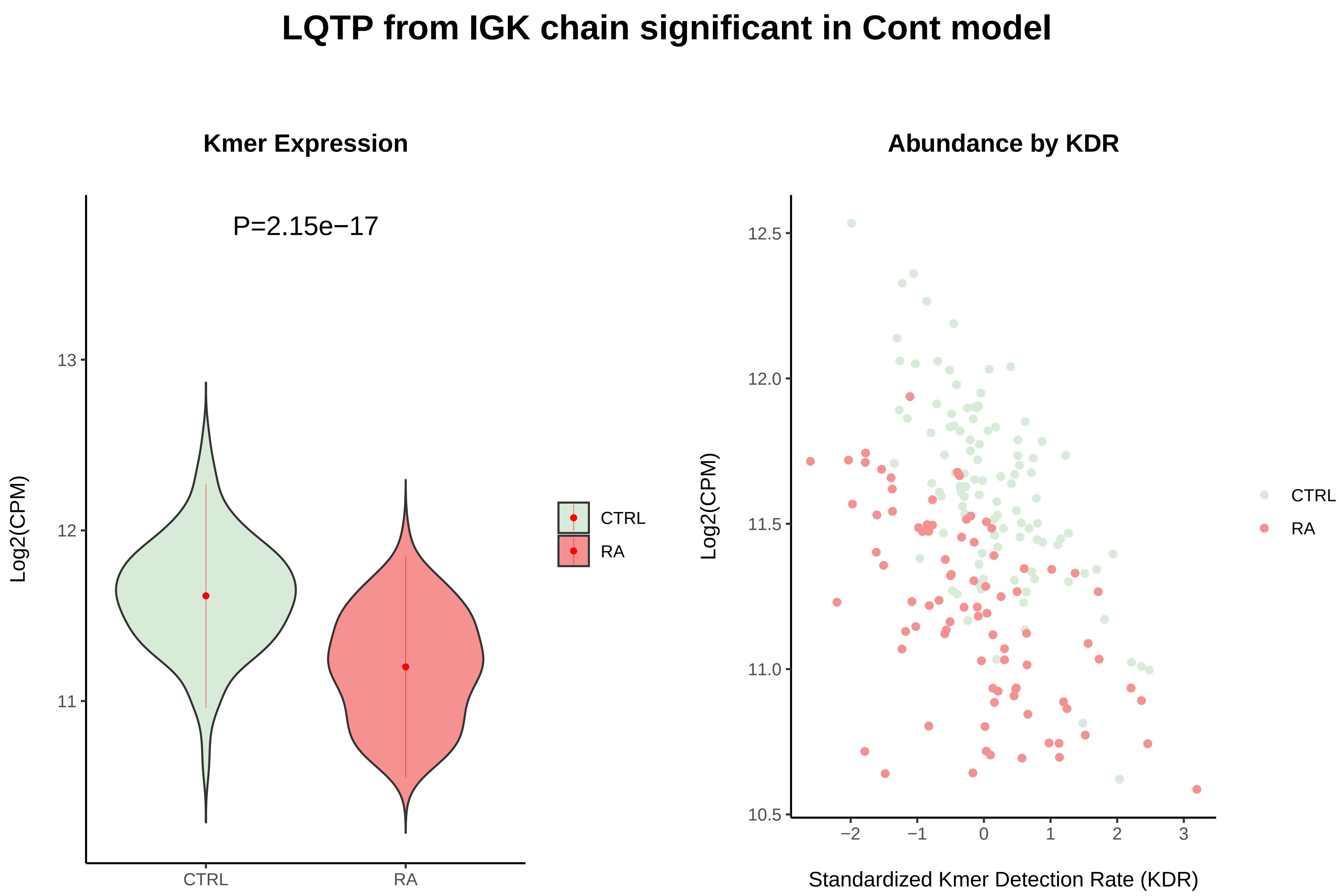
LQTP from IGK chain significant in Cont model
Kmer Expression
P=2.15e−17
Log2(CPM)
13
12
11
CTRL
RA
CTRL
RA
Abundance by KDR
Log2(CPM)
12.5
12.0
11.5
11.0
10.5
−2
−1
0
1
2
3
Standardized Kmer Detection Rate (KDR)
CTRL
RA

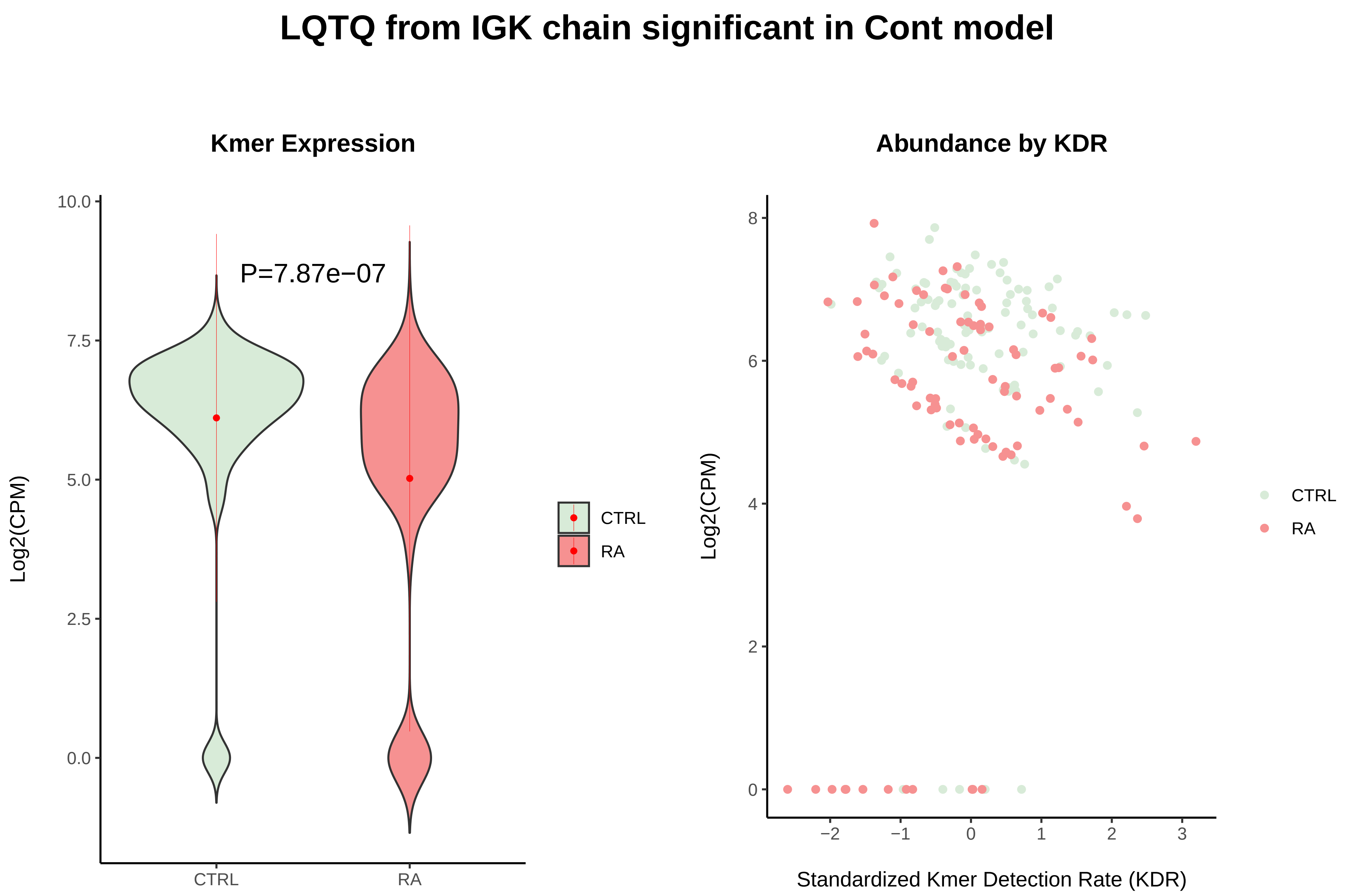
LQTQ from IGK chain significant in Cont model
Kmer Expression
Abundance by KDR
P=7.87e−07
Log2(CPM)
10.0
7.5
5.0
2.5
0.0
CTRL
RA
8
6
4
2
0
−2
−1
0
1
2
3
Standardized Kmer Detection Rate (KDR)

LQTR from IGK chain significant in Cont model

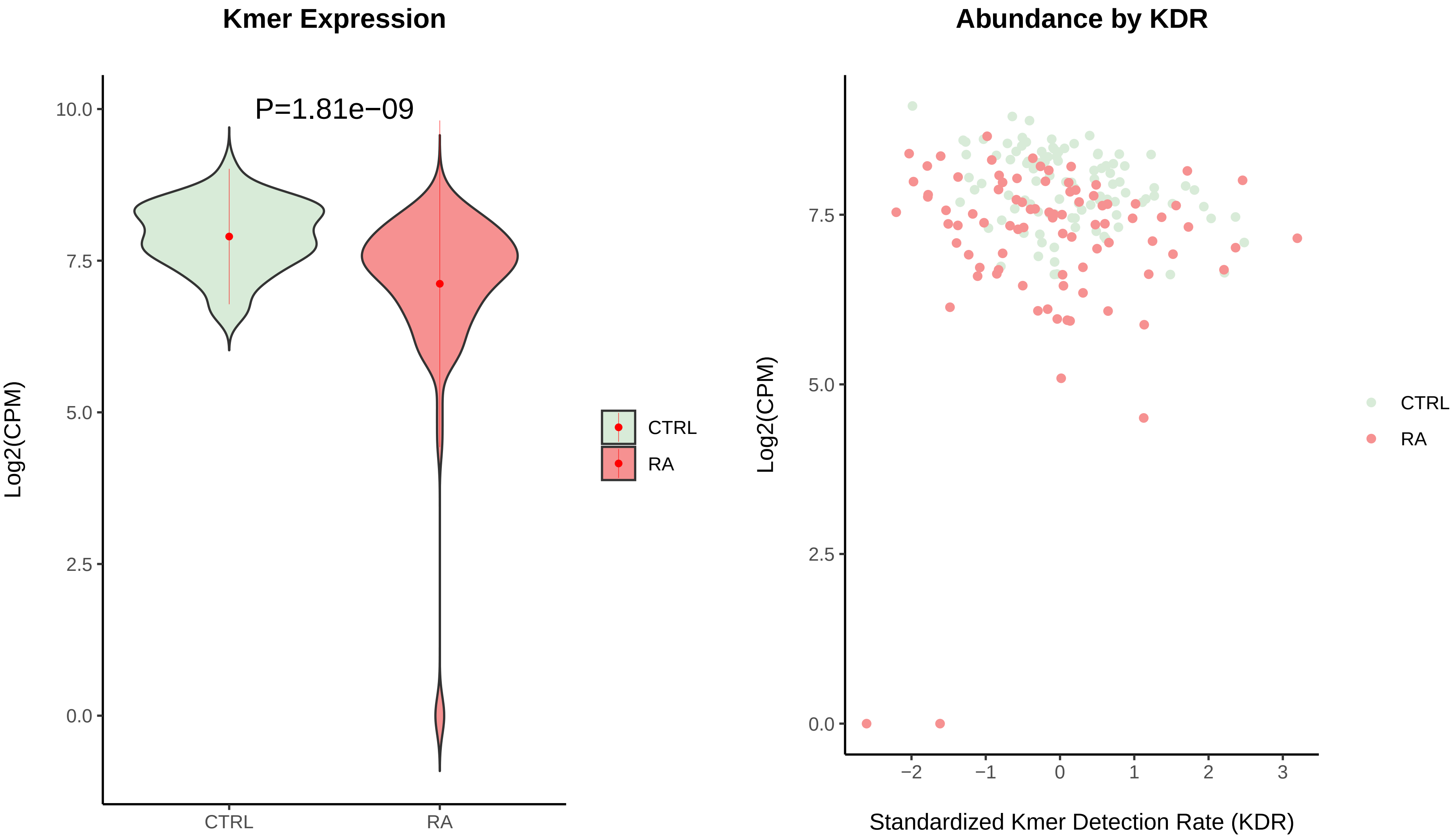

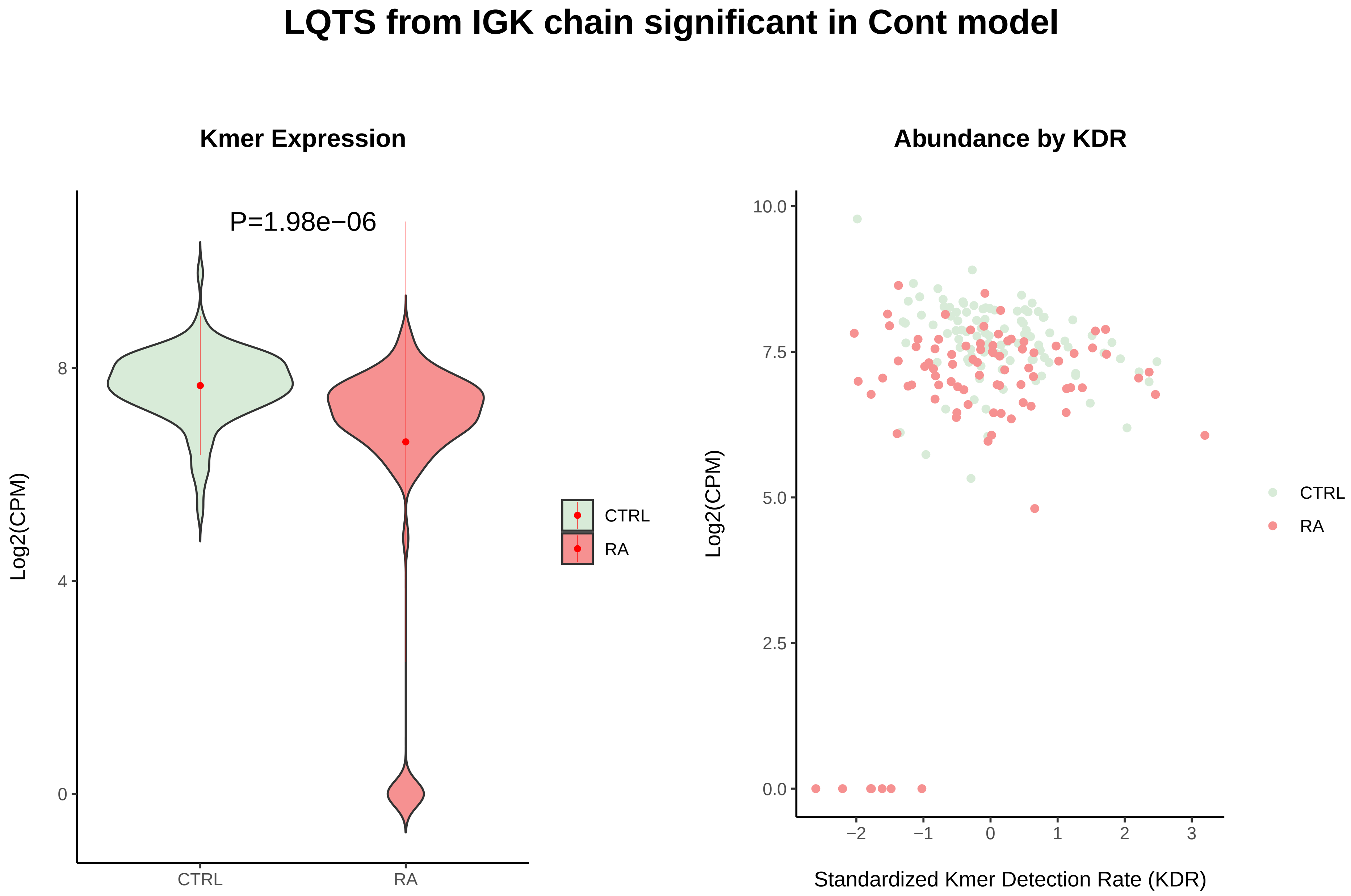
LQTS from IGK chain significant in Cont model
Kmer Expression
P=1.98e−06
Log2(CPM)
8
4
0
CTRL
RA
CTRL
RA
Abundance by KDR
Log2(CPM)
10.0
7.5
5.0
2.5
0.0
−2
−1
0
1
2
3
Standardized Kmer Detection Rate (KDR)
CTRL
RA

MQAL from IGK chain significant in Cont model

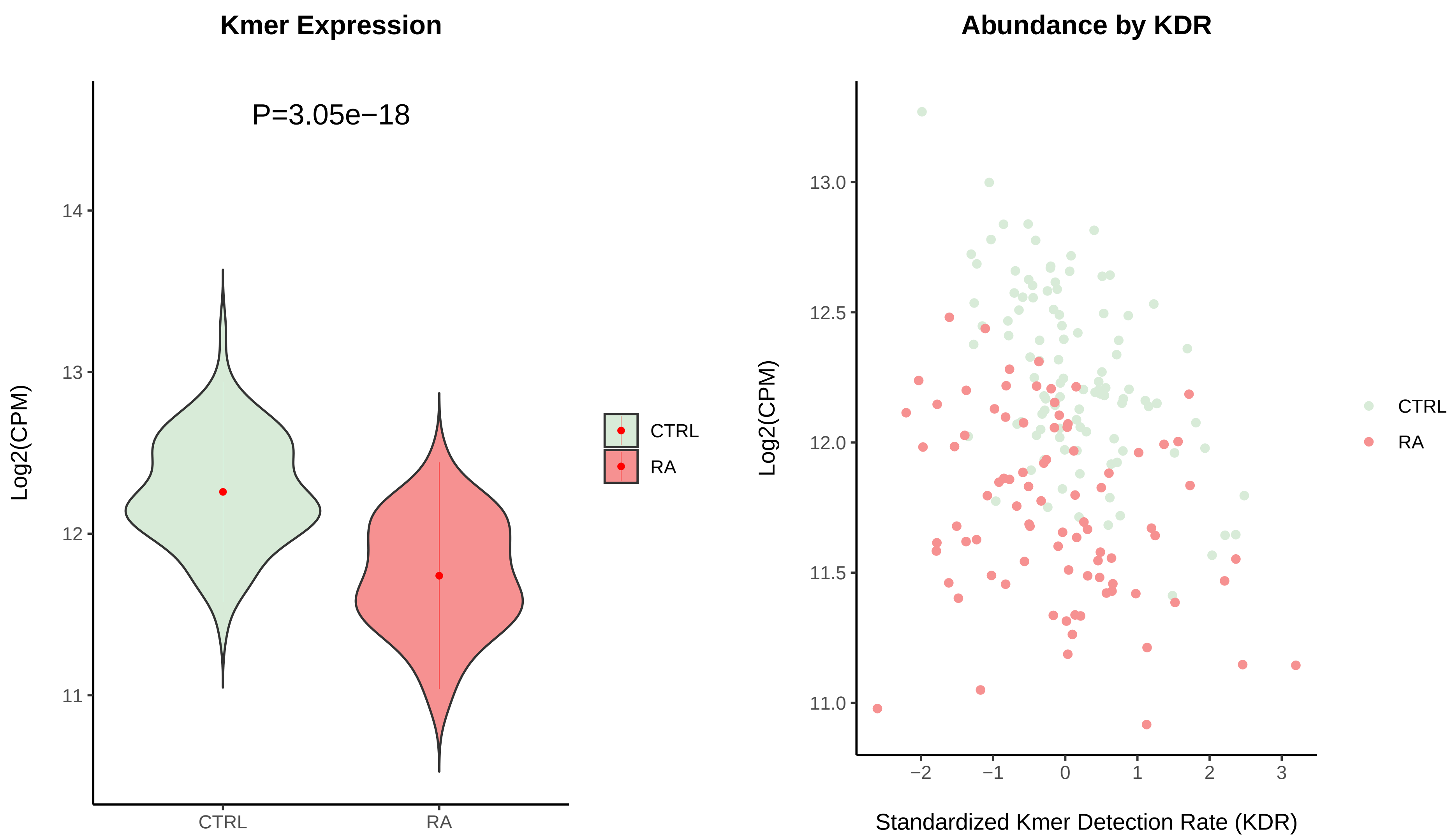

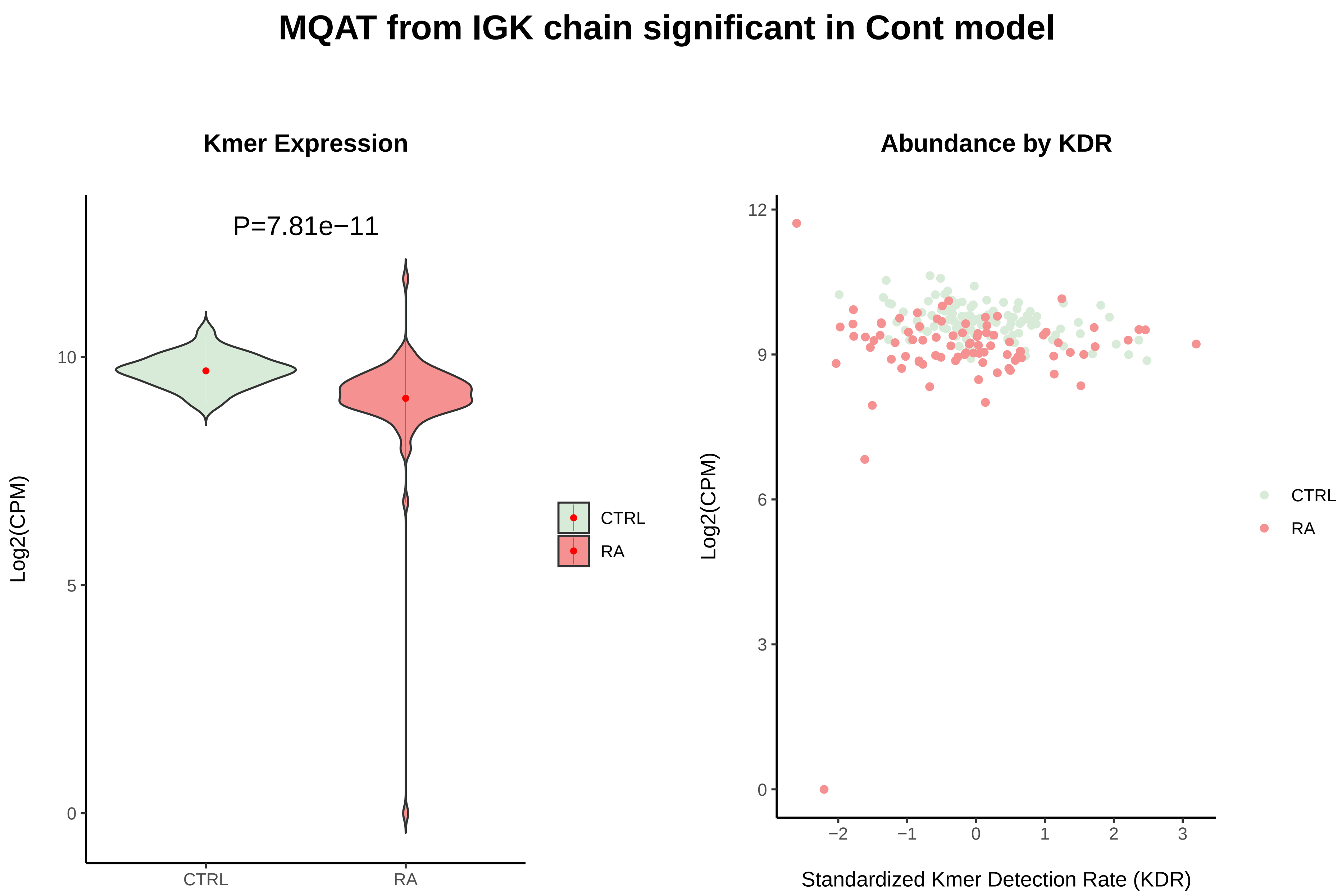
MQAT from IGK chain significant in Cont model
Kmer Expression
P=7.81e−11
Log2(CPM)
0
5
10
CTRL
RA
CTRL
RA
Abundance by KDR
Log2(CPM)
0
3
6
9
12
−2
−1
0
1
2
3
Standardized Kmer Detection Rate (KDR)
CTRL
RA

MQGT from IGK chain significant in Cont model

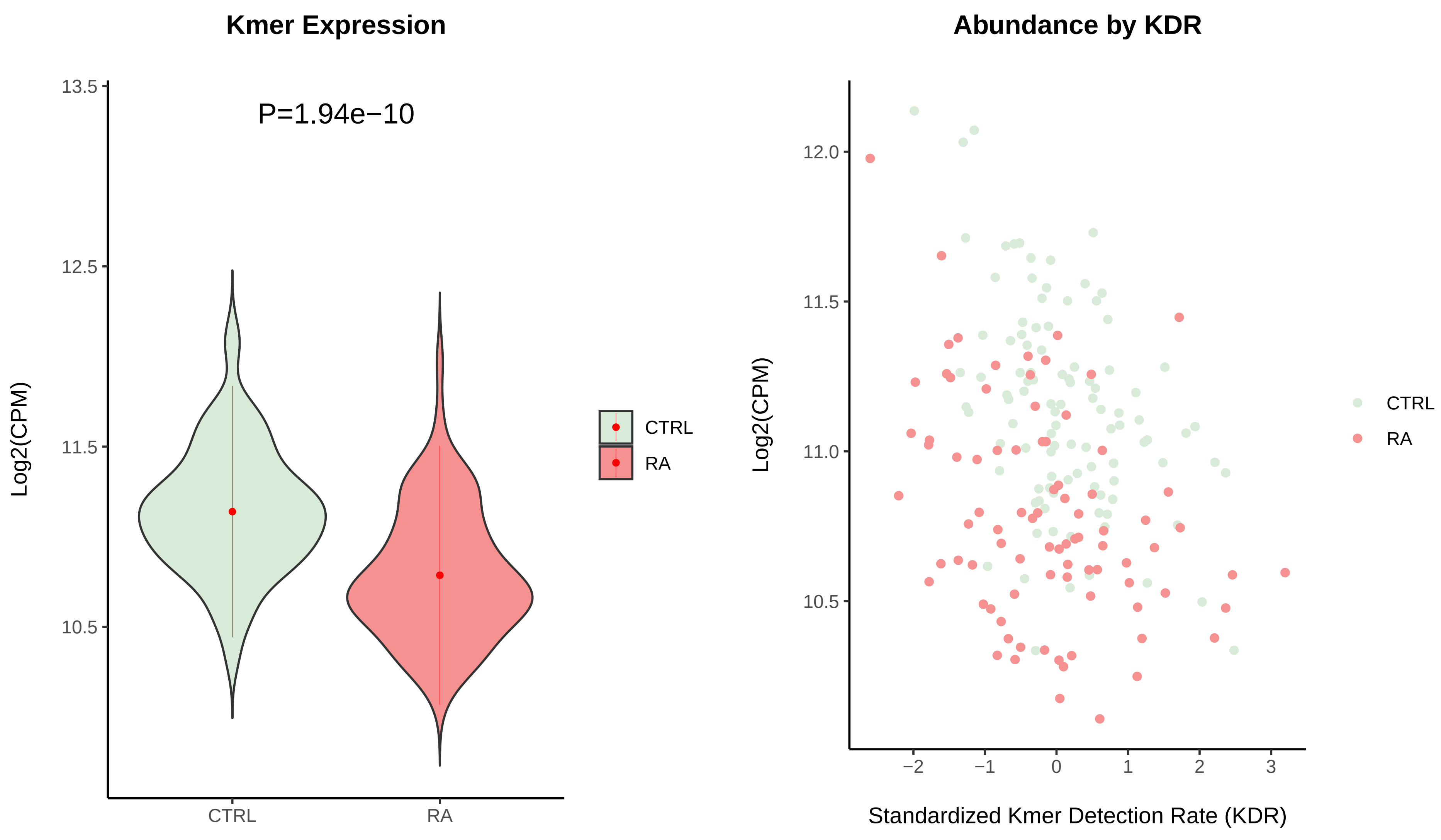

MQTL from IGK chain significant in Cont model

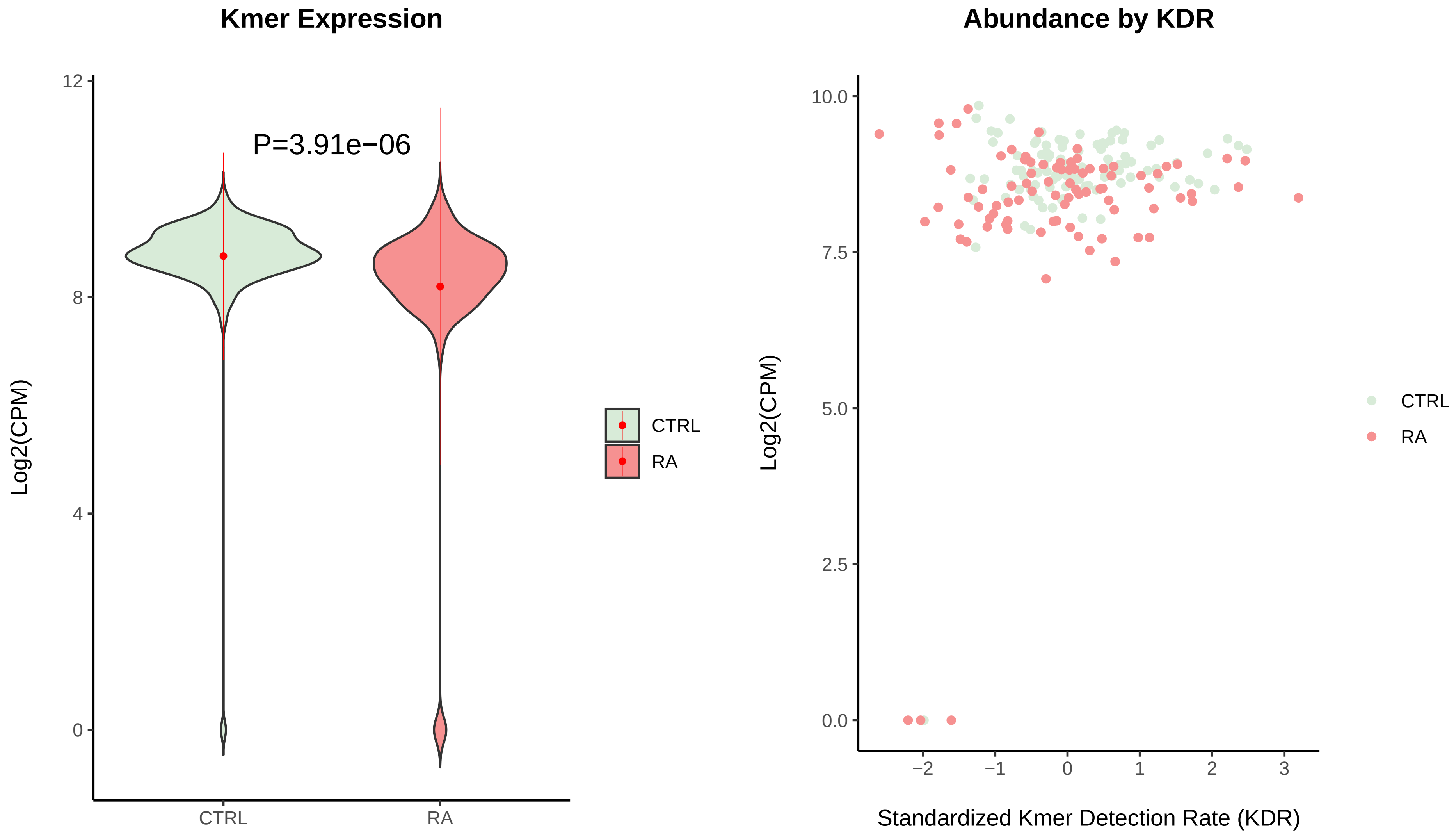

MQVL from IGK chain significant in Cont model

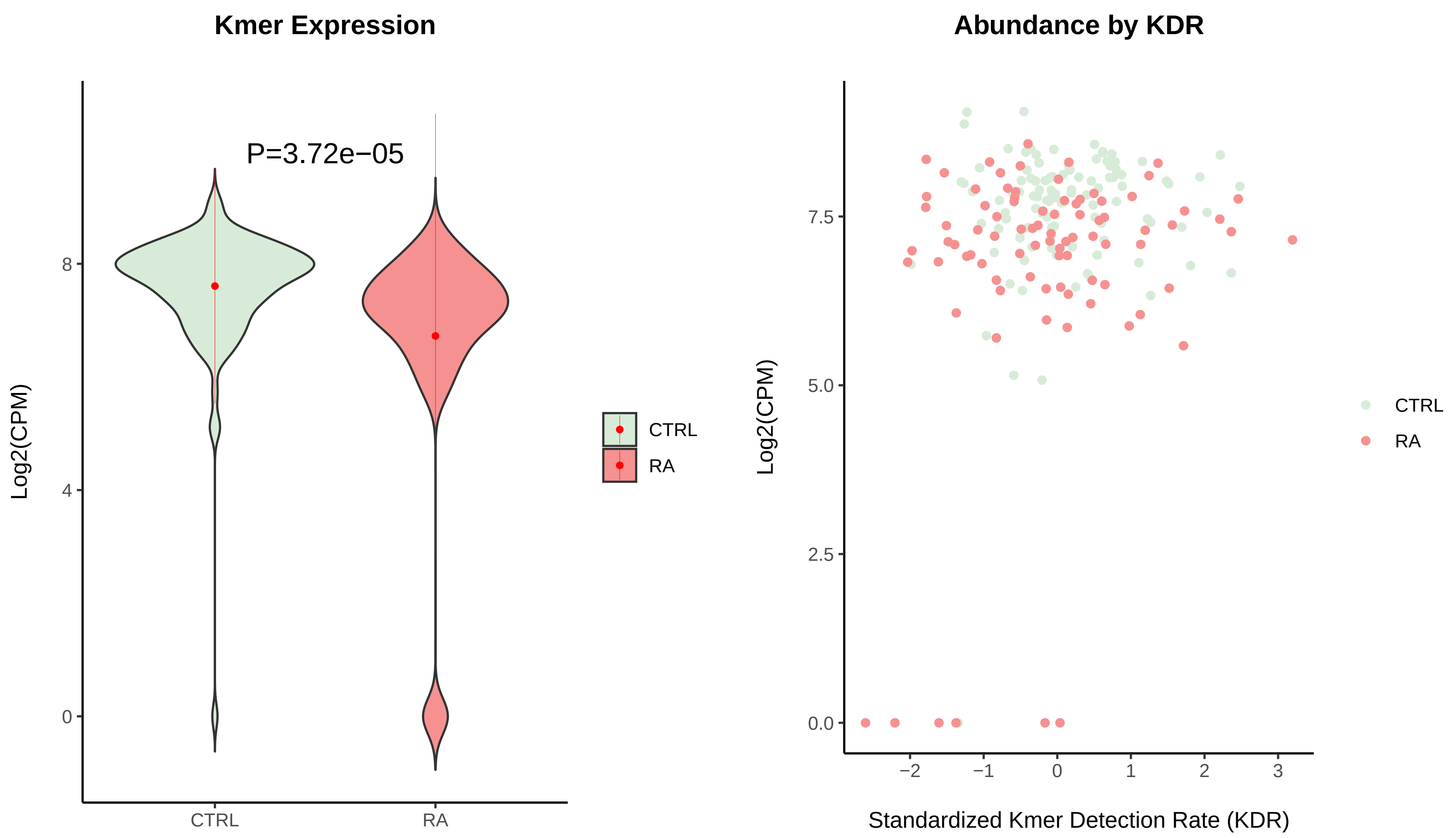

QALQ from IGK chain significant in Cont model

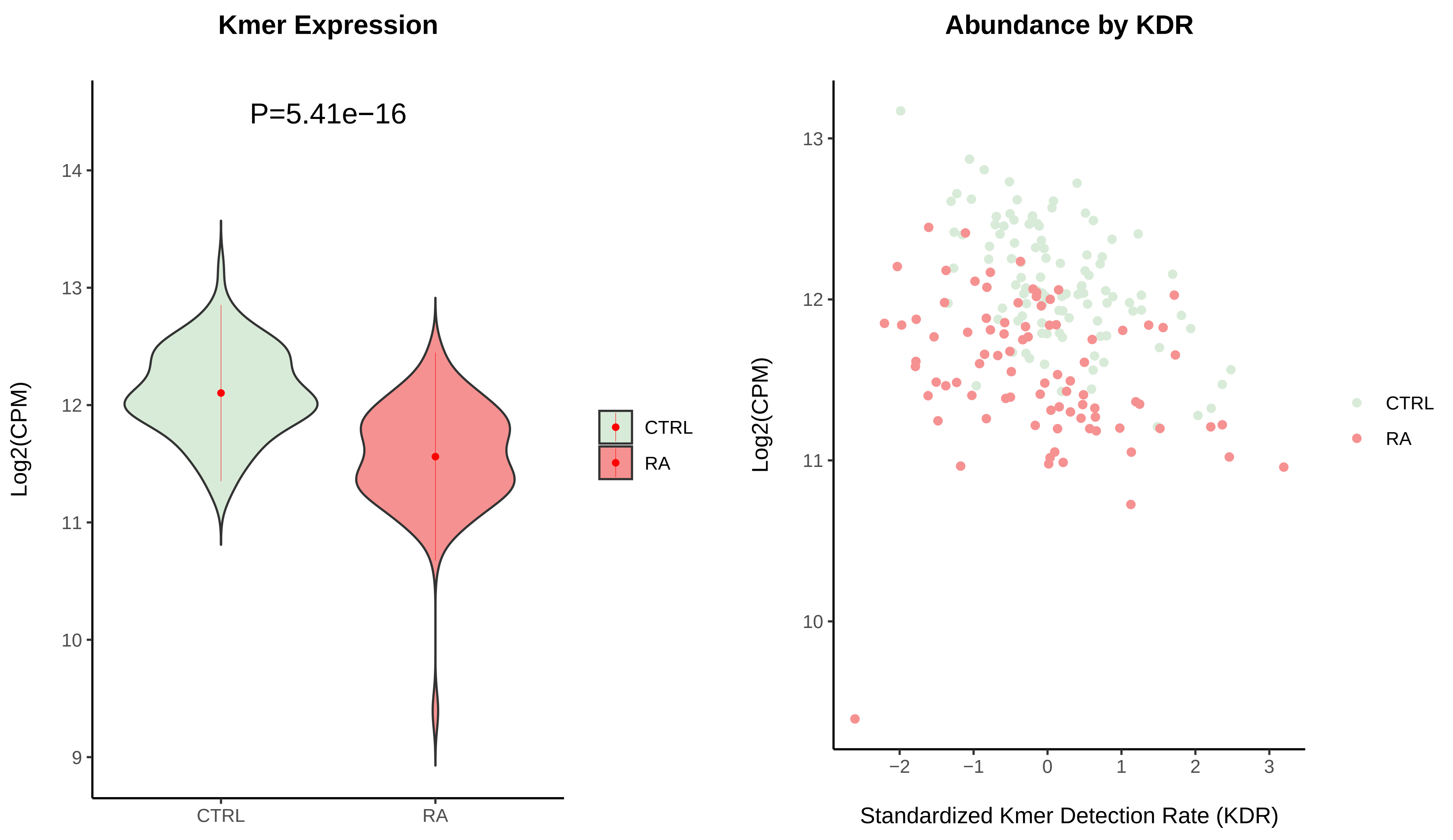

QATH from IGK chain significant in Cont model

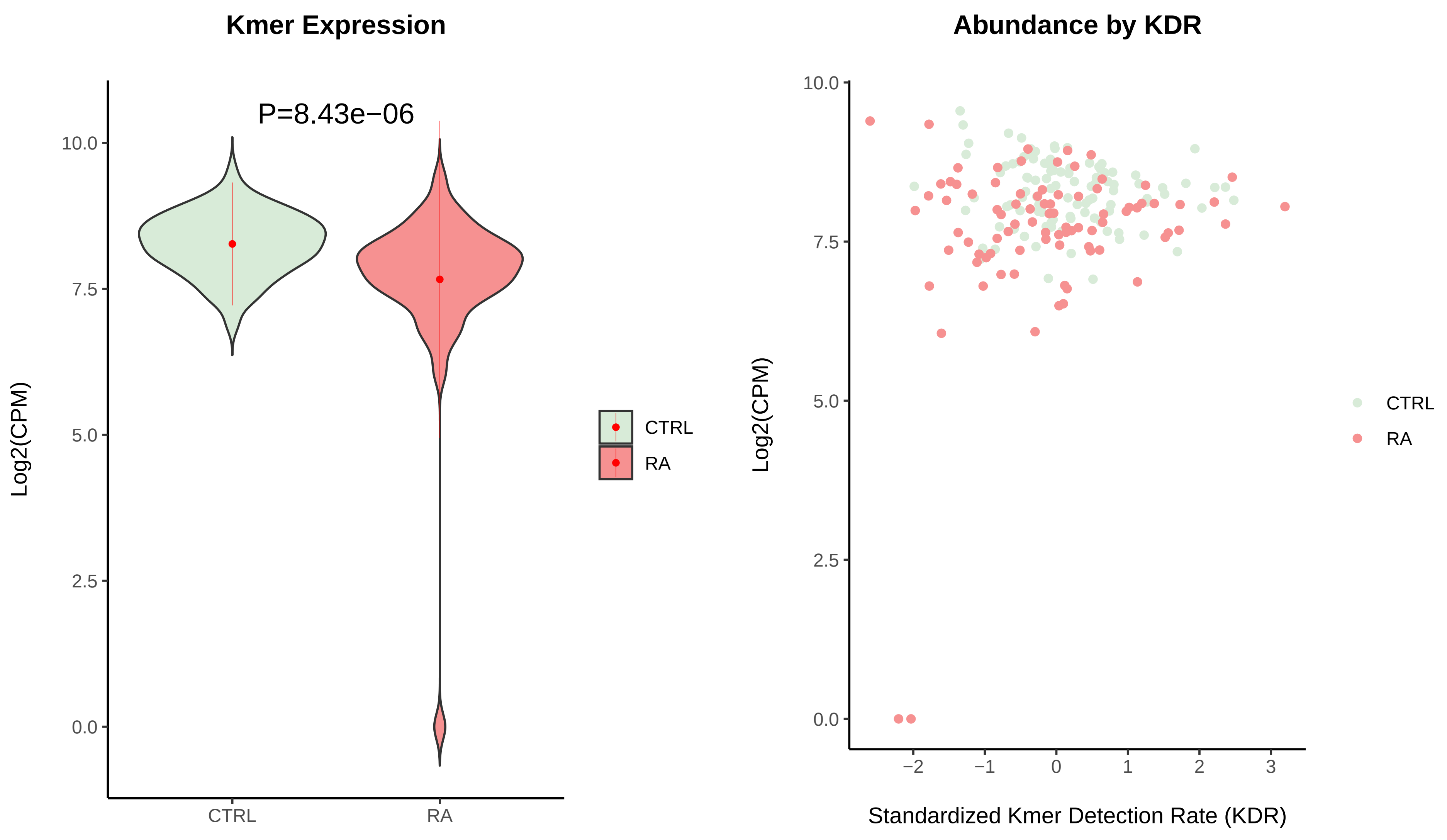

QATQ from IGK chain significant in Cont model

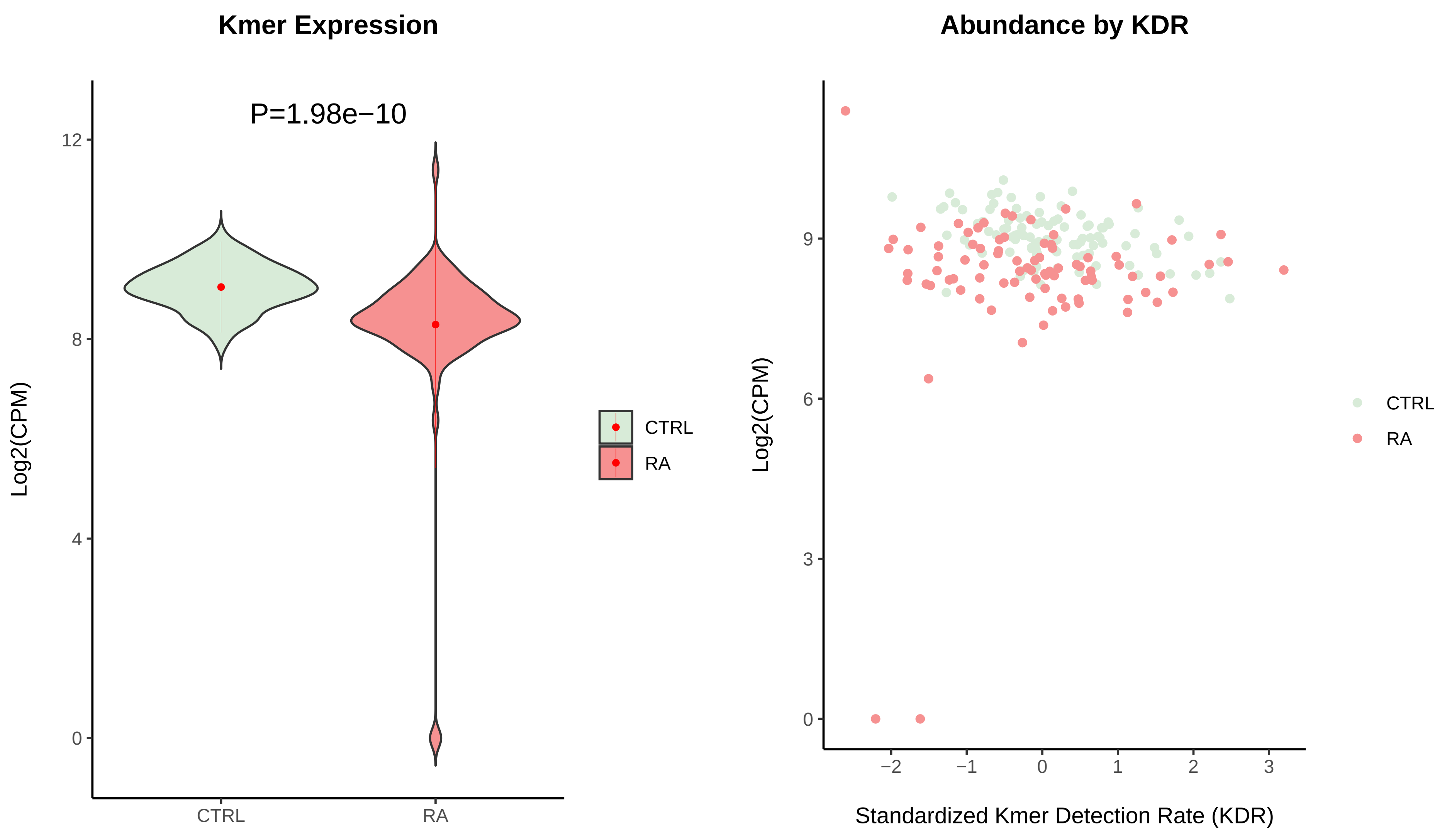

QGTH from IGK chain significant in Cont model

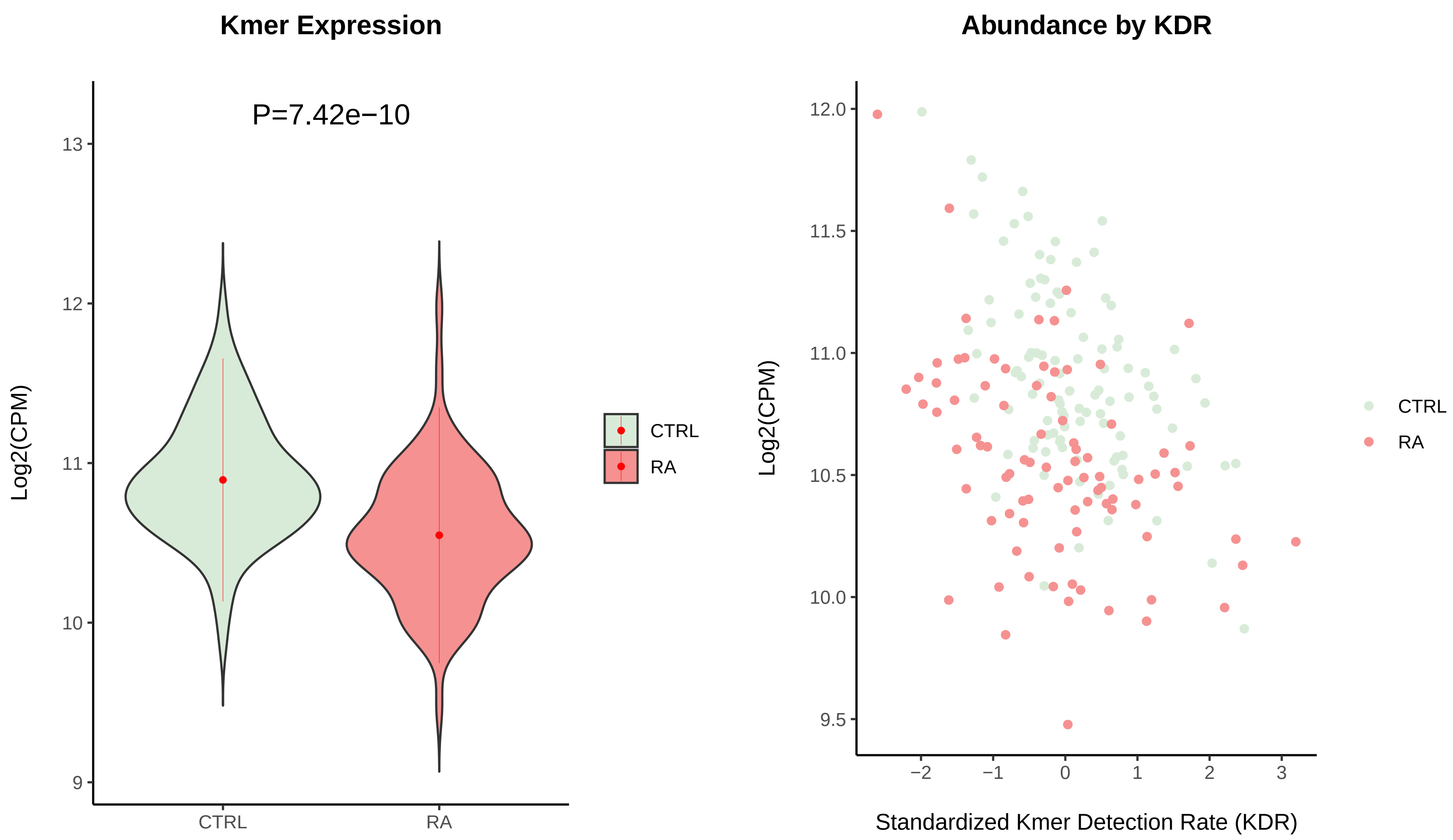

QTPF from IGK chain significant in Cont model

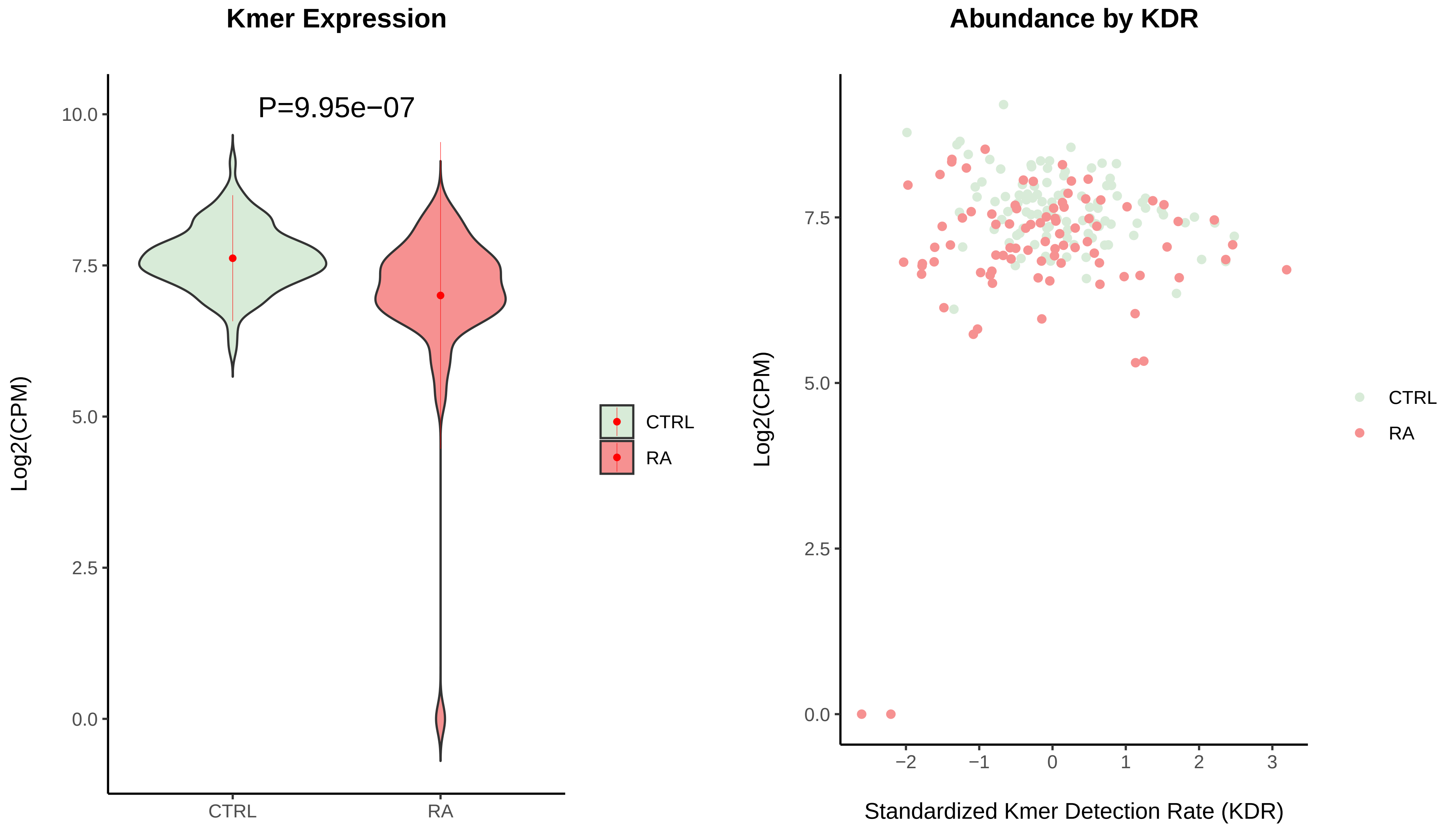

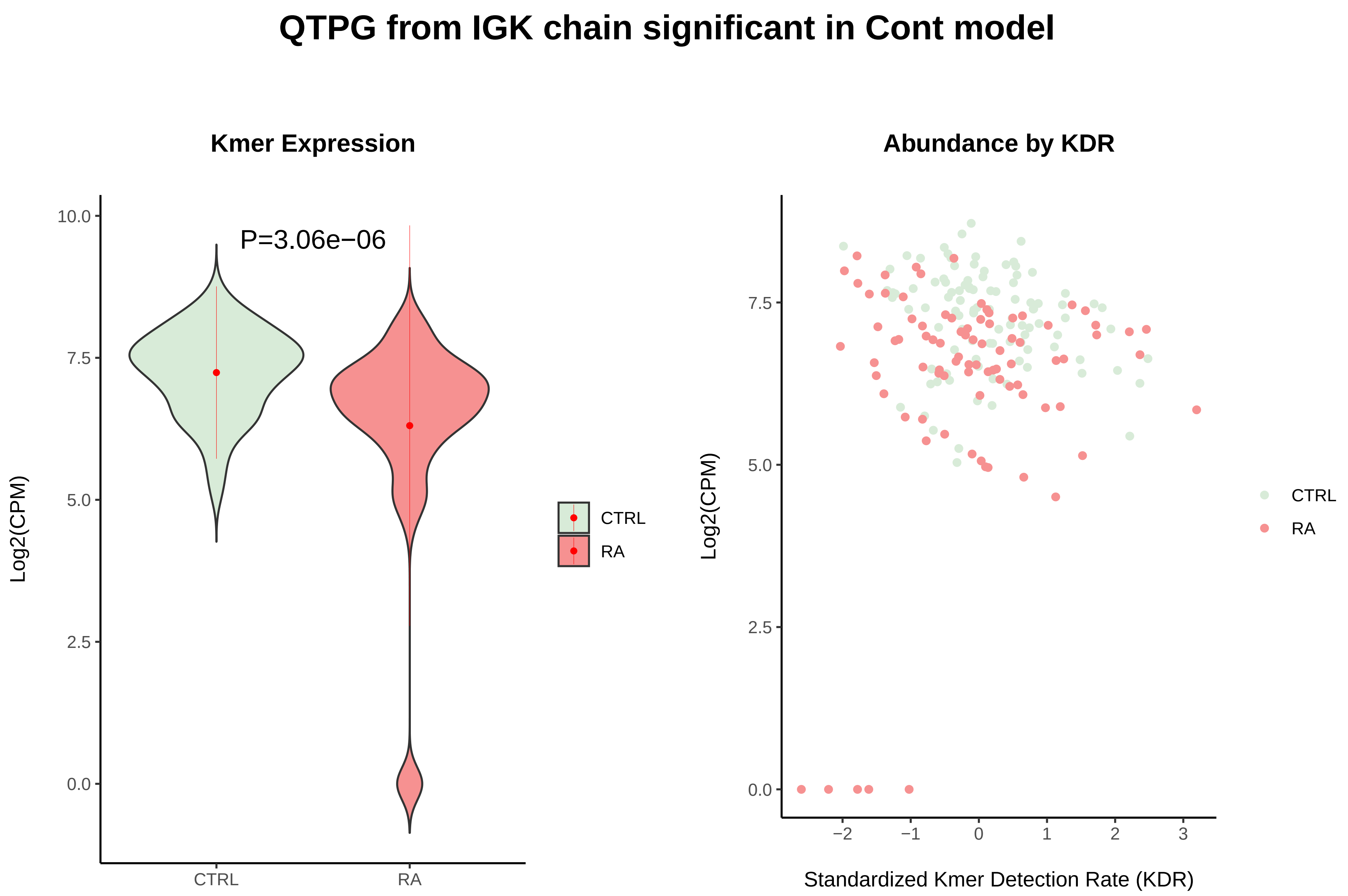
QTPG from IGK chain significant in Cont model
Kmer Expression
P=3.06e−06
Log2(CPM)
10.0
7.5
5.0
2.5
0.0
CTRL
RA
Abundance by KDR
Log2(CPM)
7.5
5.0
2.5
0.0
−2
−1
0
1
2
3
Standardized Kmer Detection Rate (KDR)
CTRL
RA

QTPI from IGK chain significant in Cont model

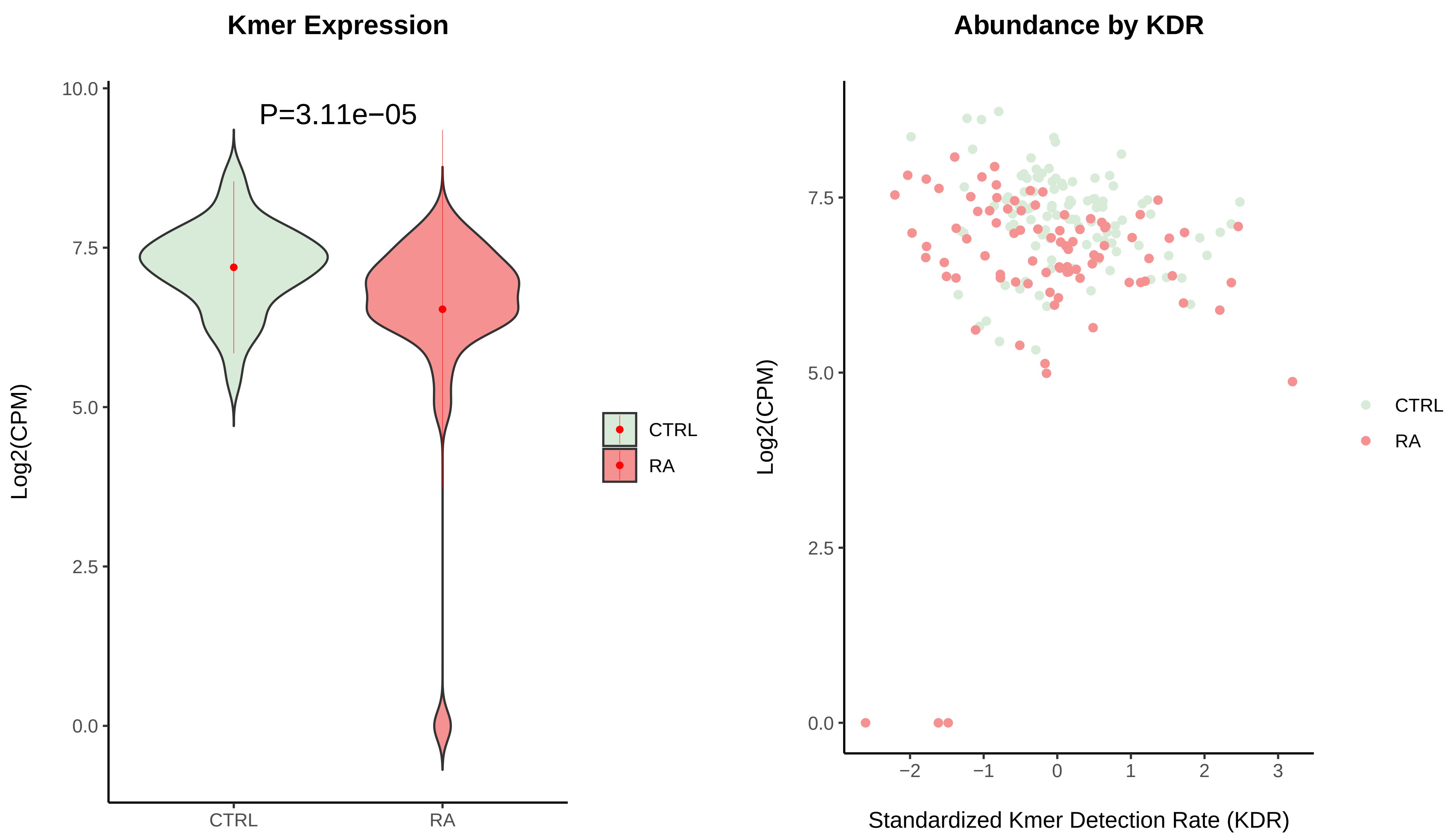

QTPL from IGK chain significant in Cont model

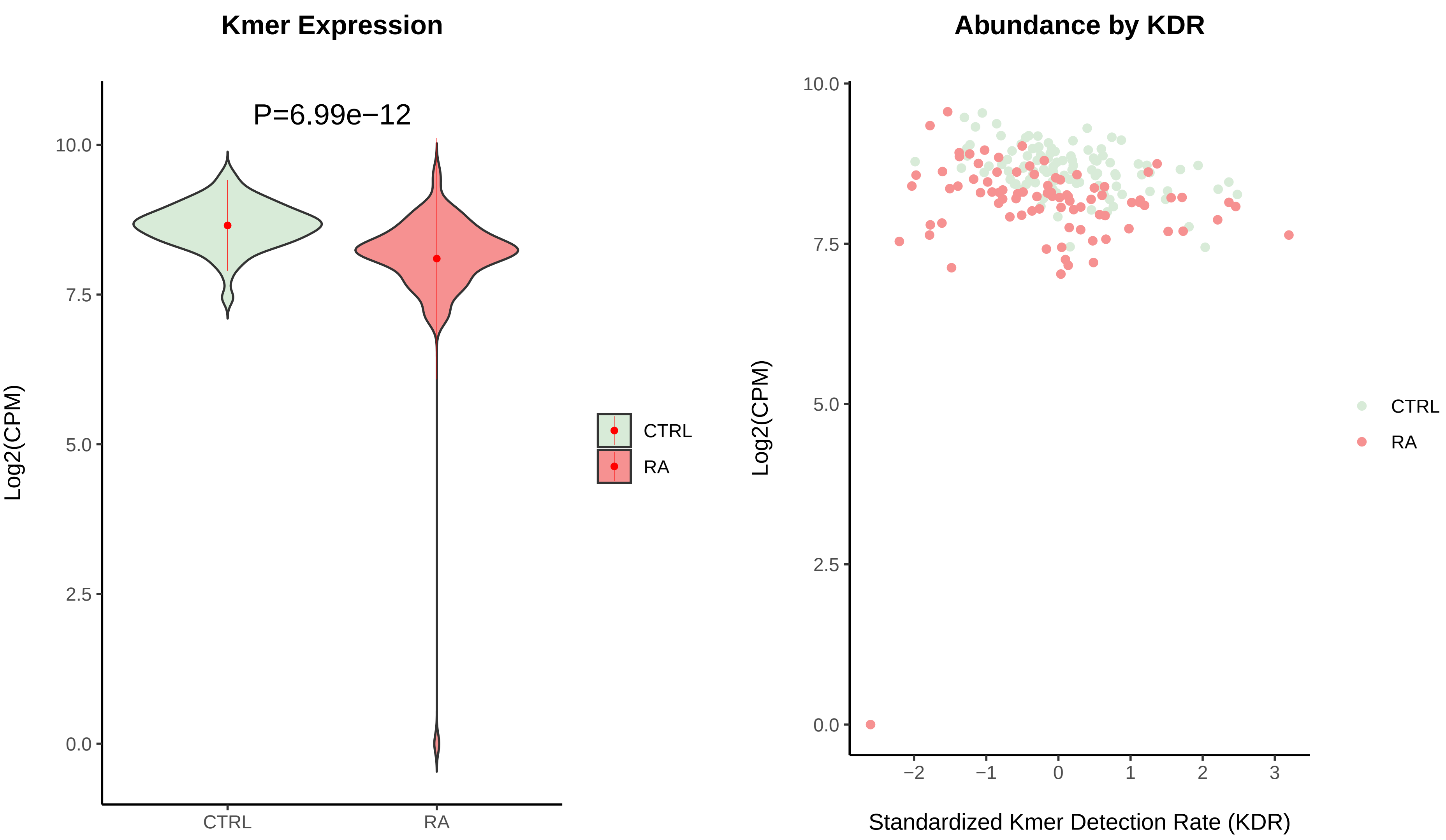

QTPP from IGK chain significant in Cont model

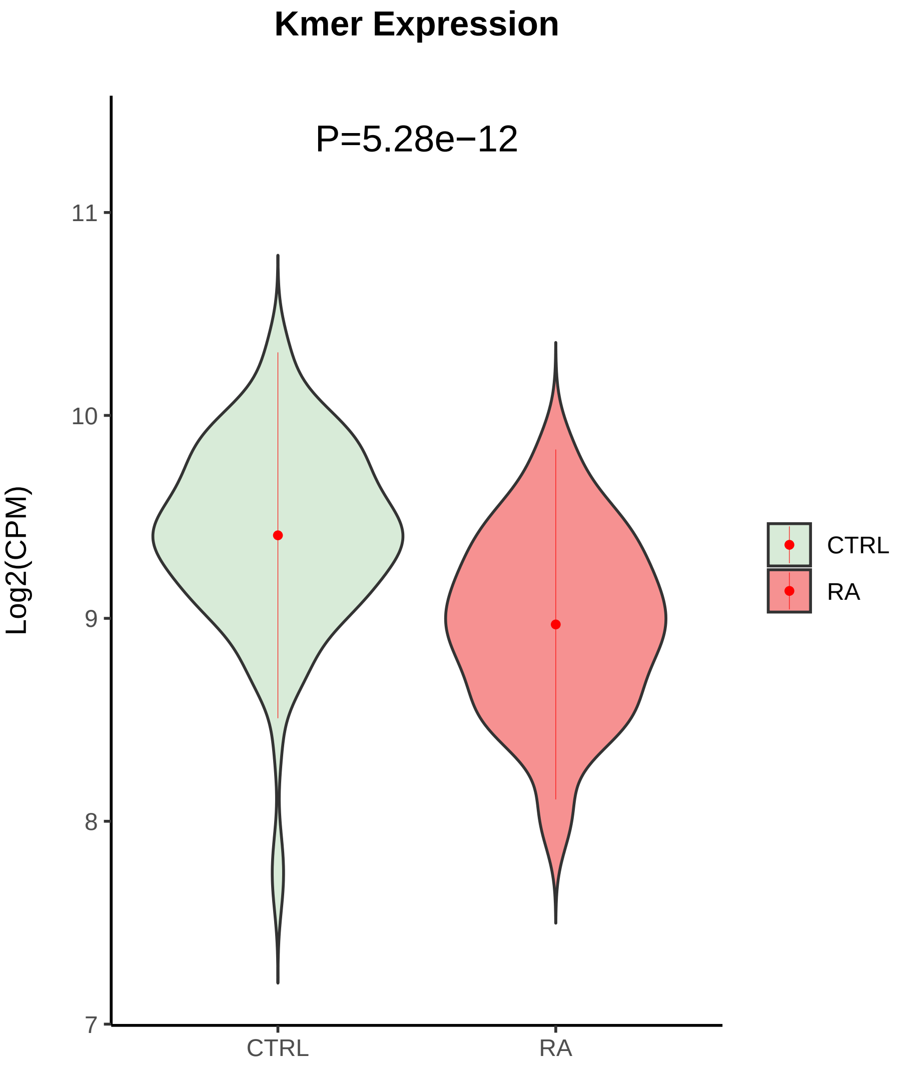

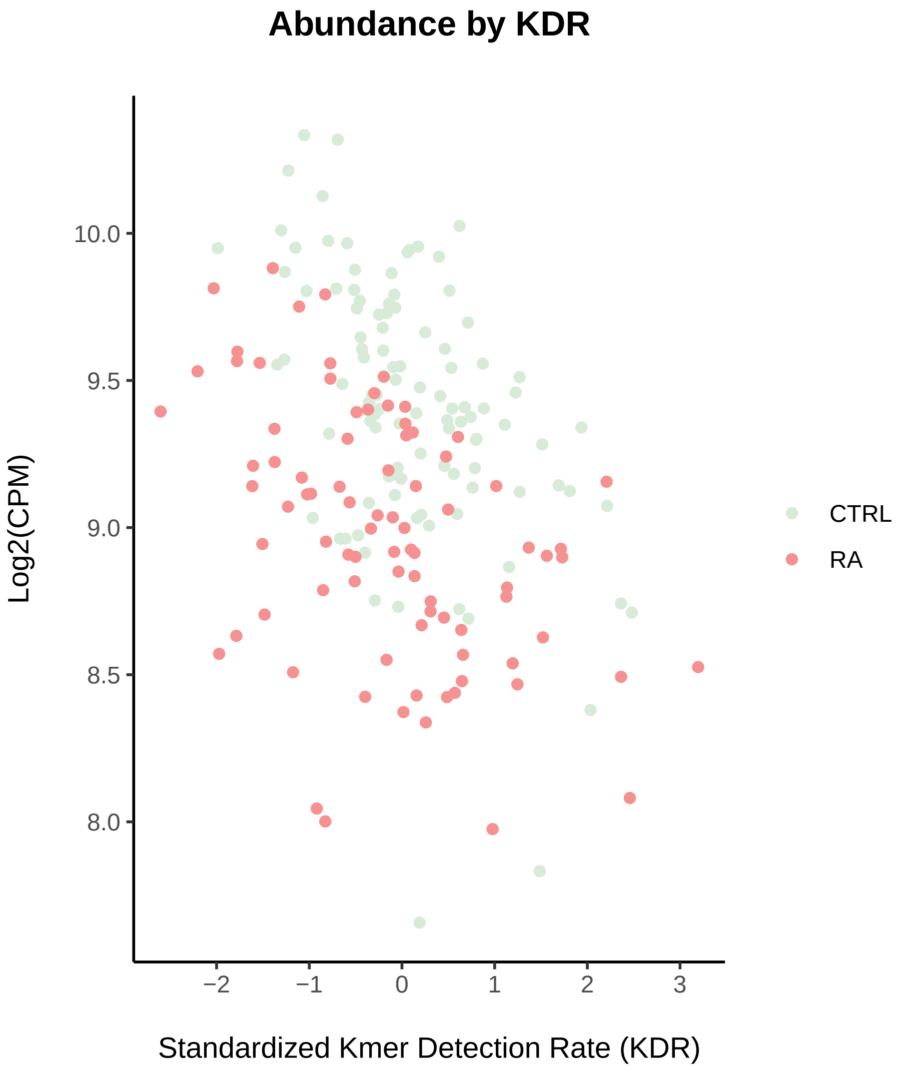

QTPQ from IGK chain significant in Cont model

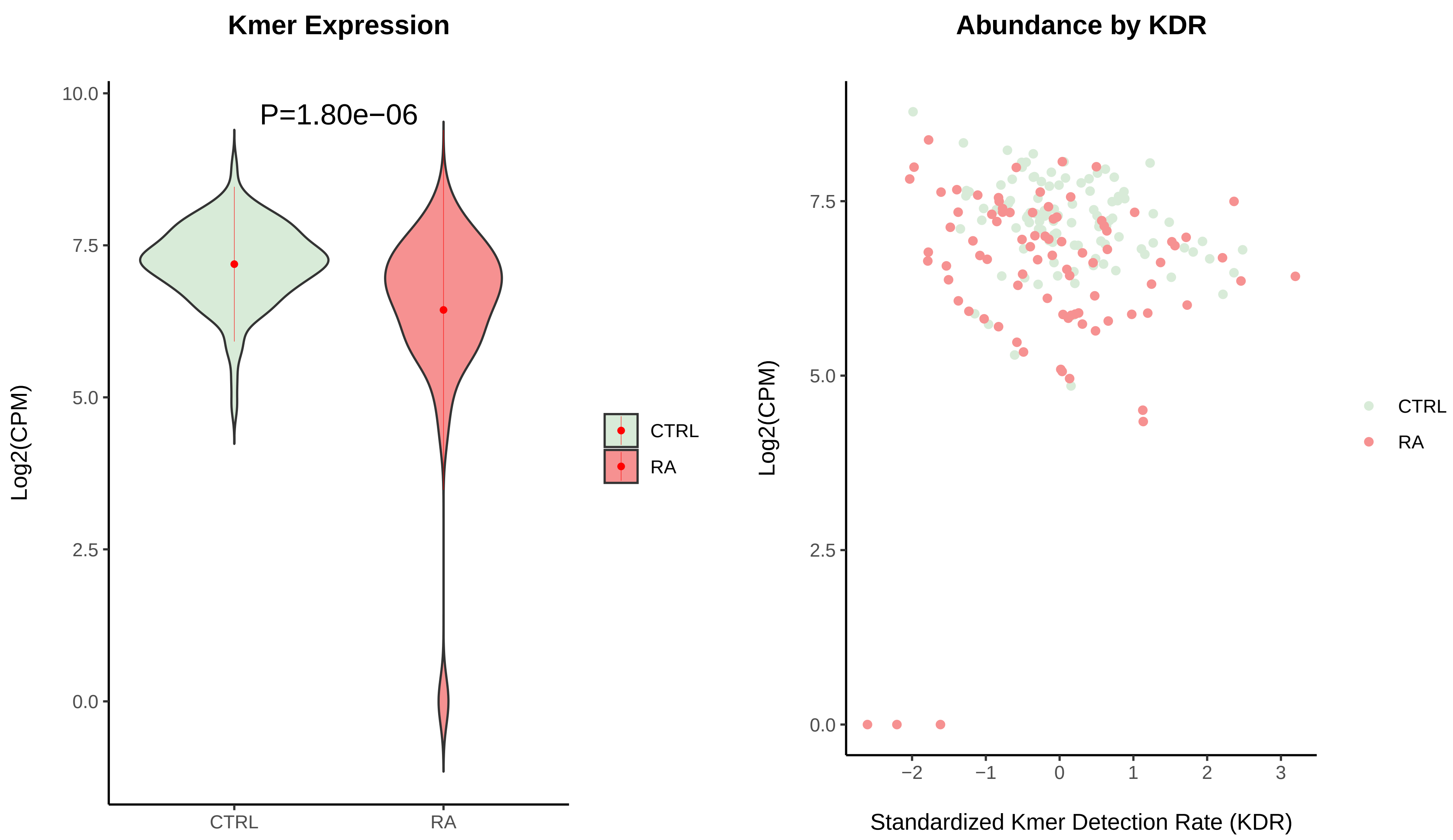

QTPR from IGK chain significant in Cont model

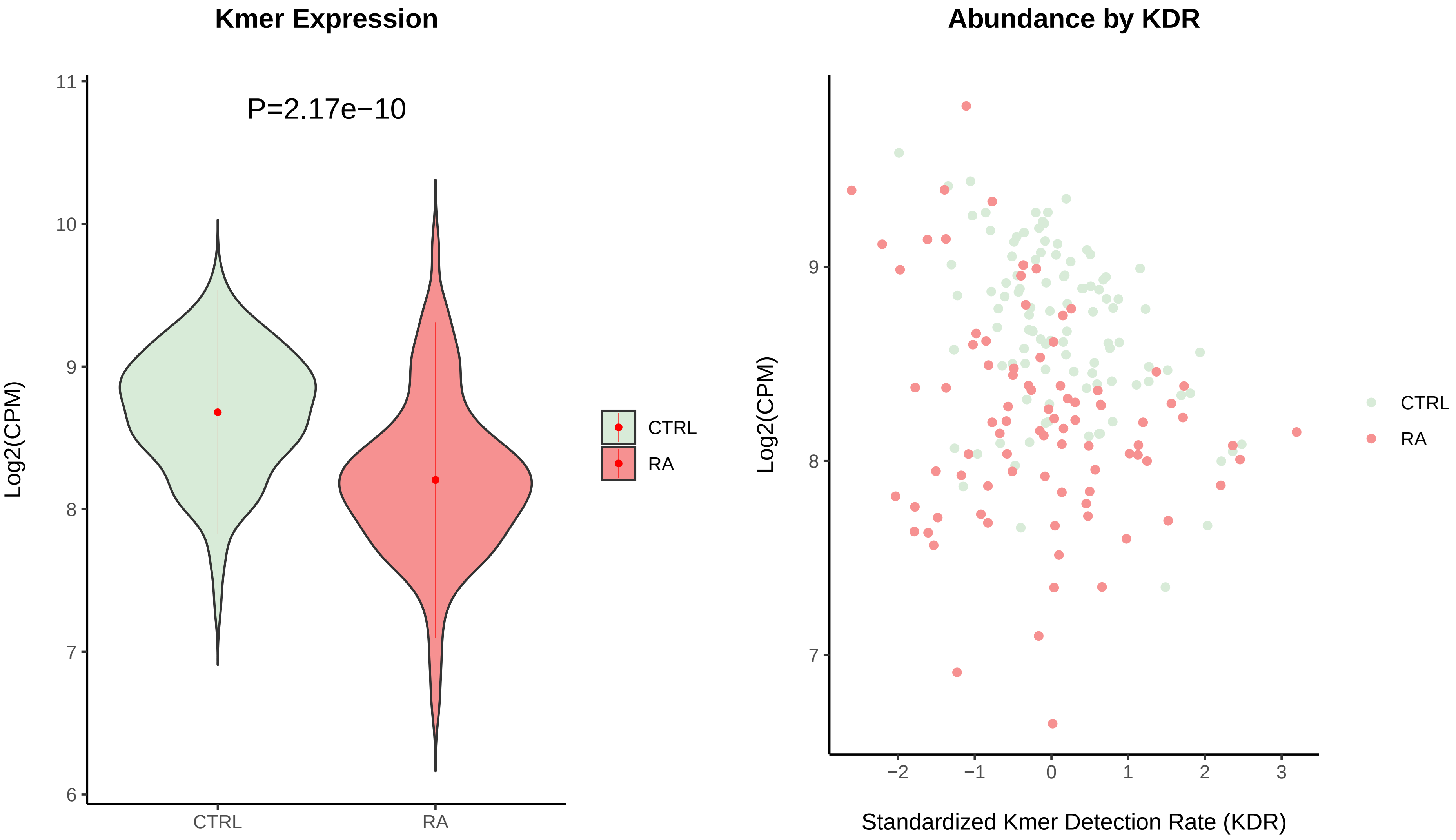

QVLQ from IGK chain significant in Cont model

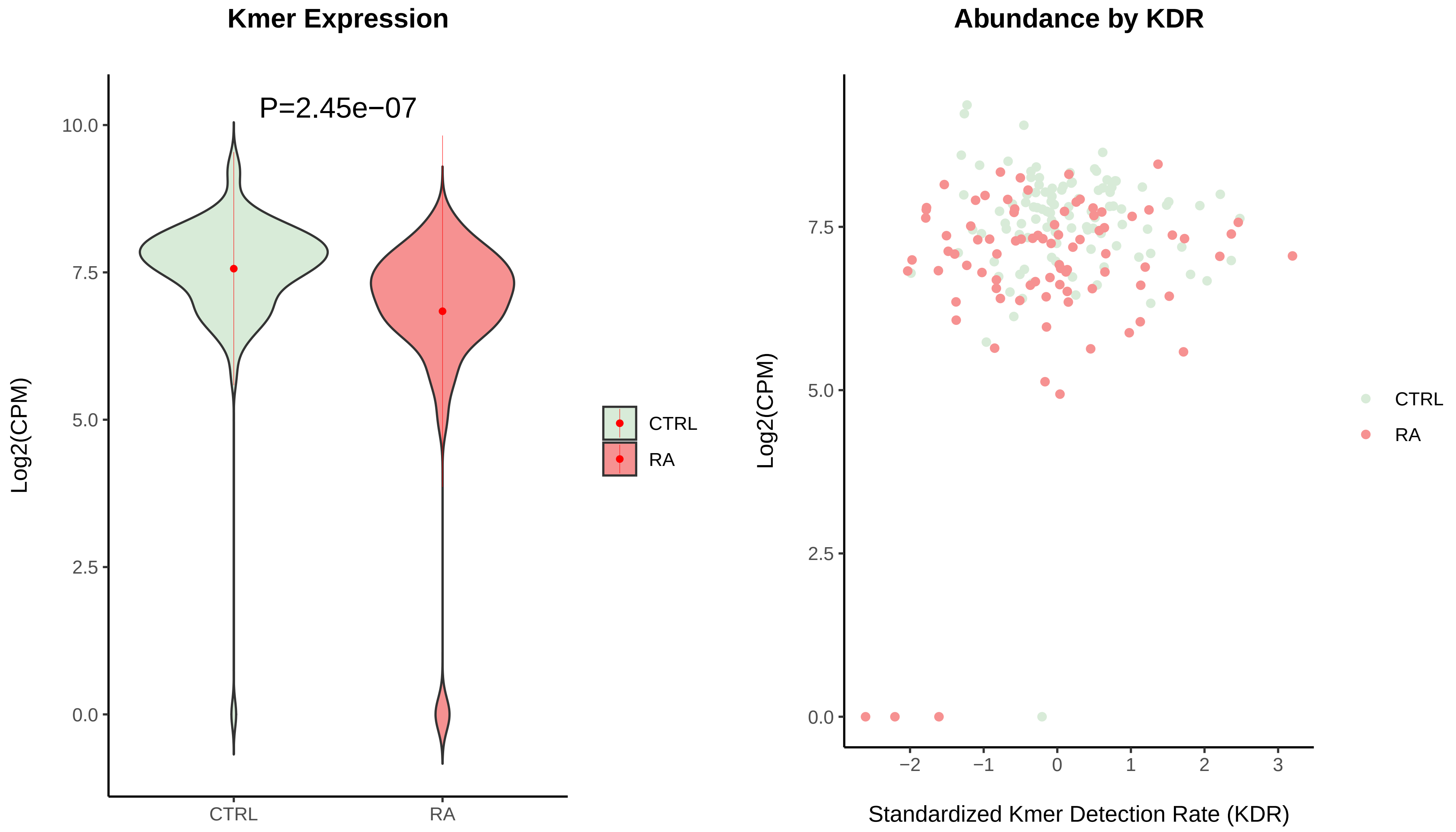

THWP from IGK chain significant in Cont model

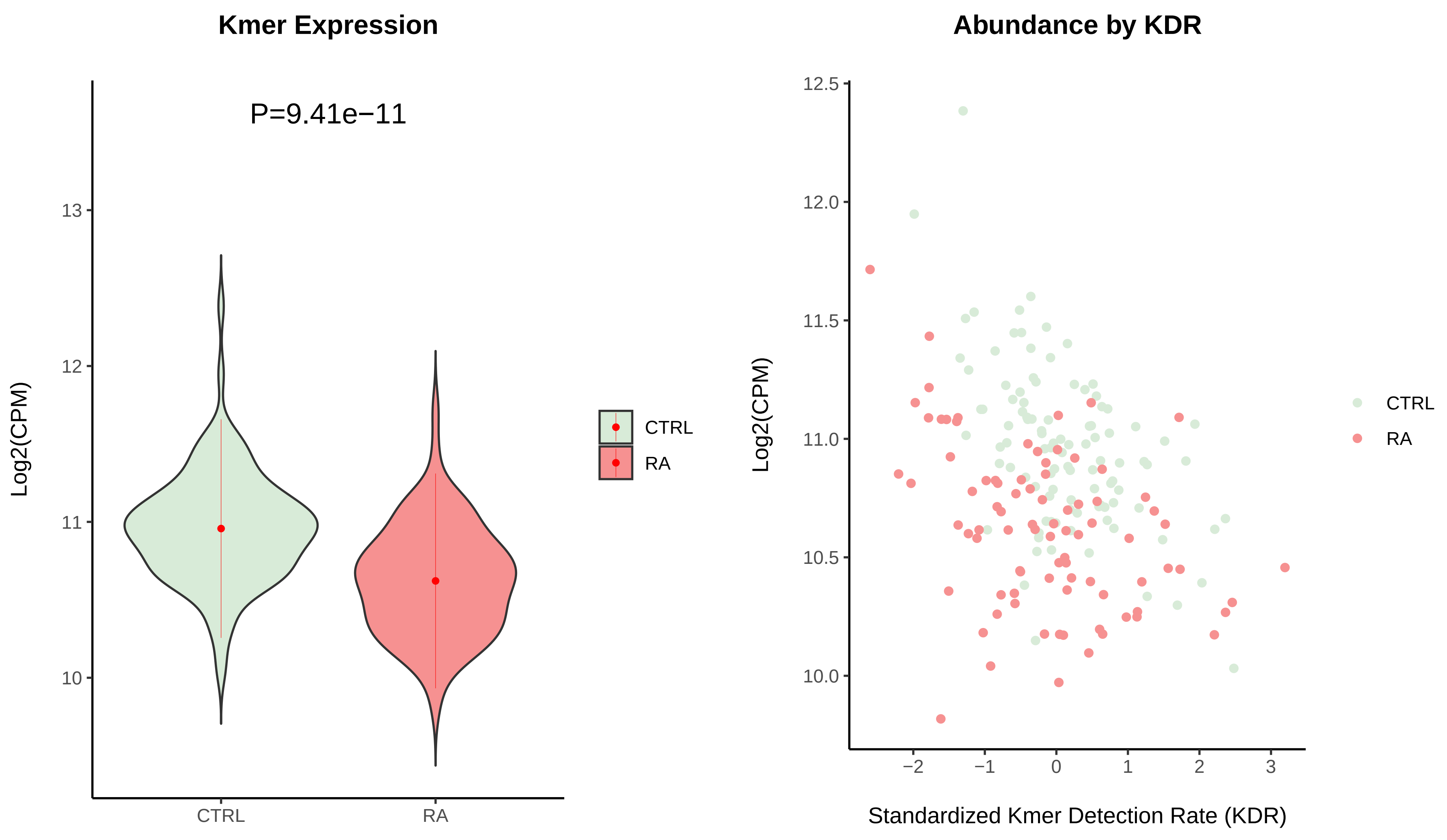

TLQT from IGK chain significant in Cont model

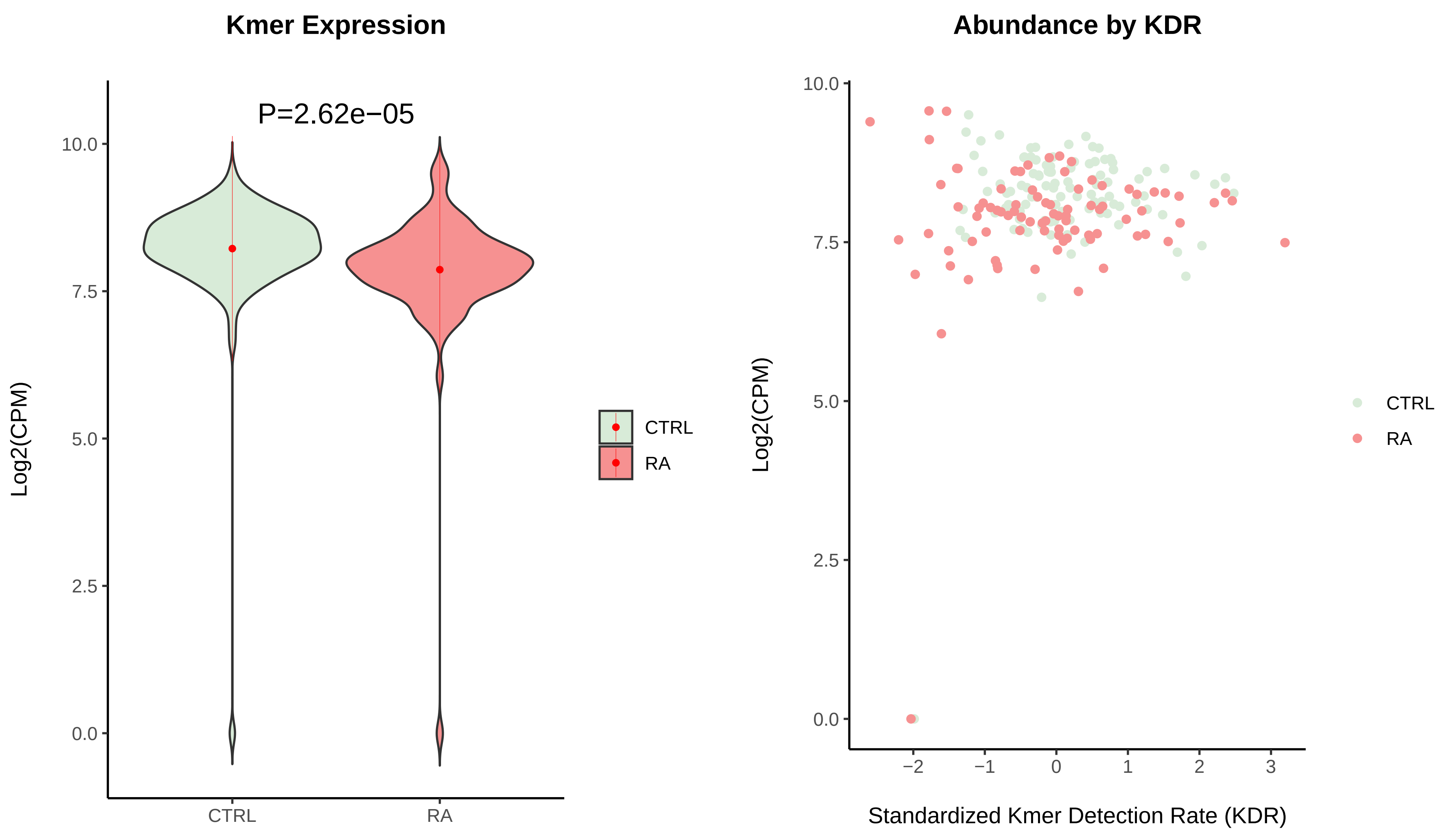

TQFP from IGK chain significant in Cont model

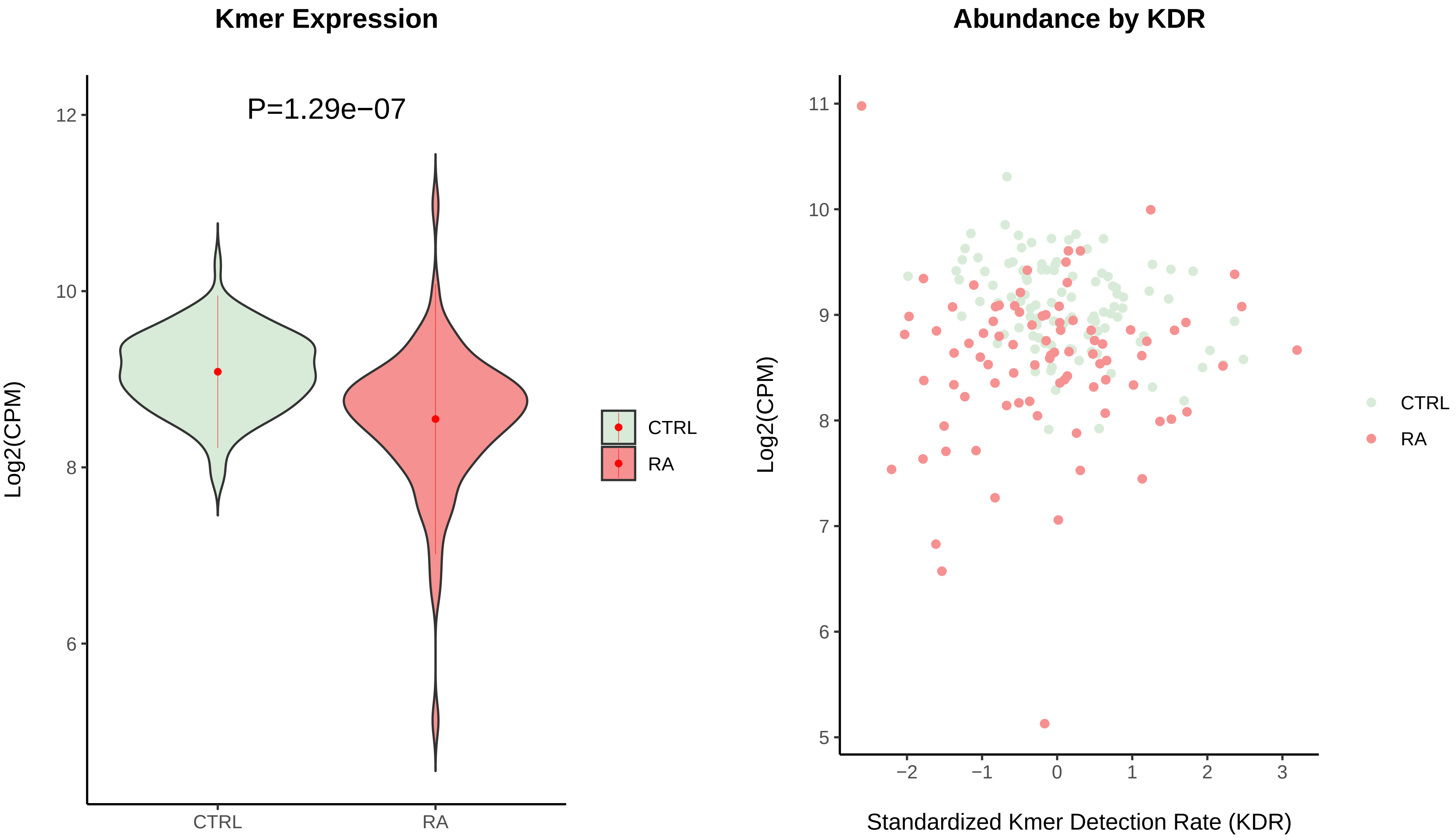

VLQT from IGK chain significant in Cont model

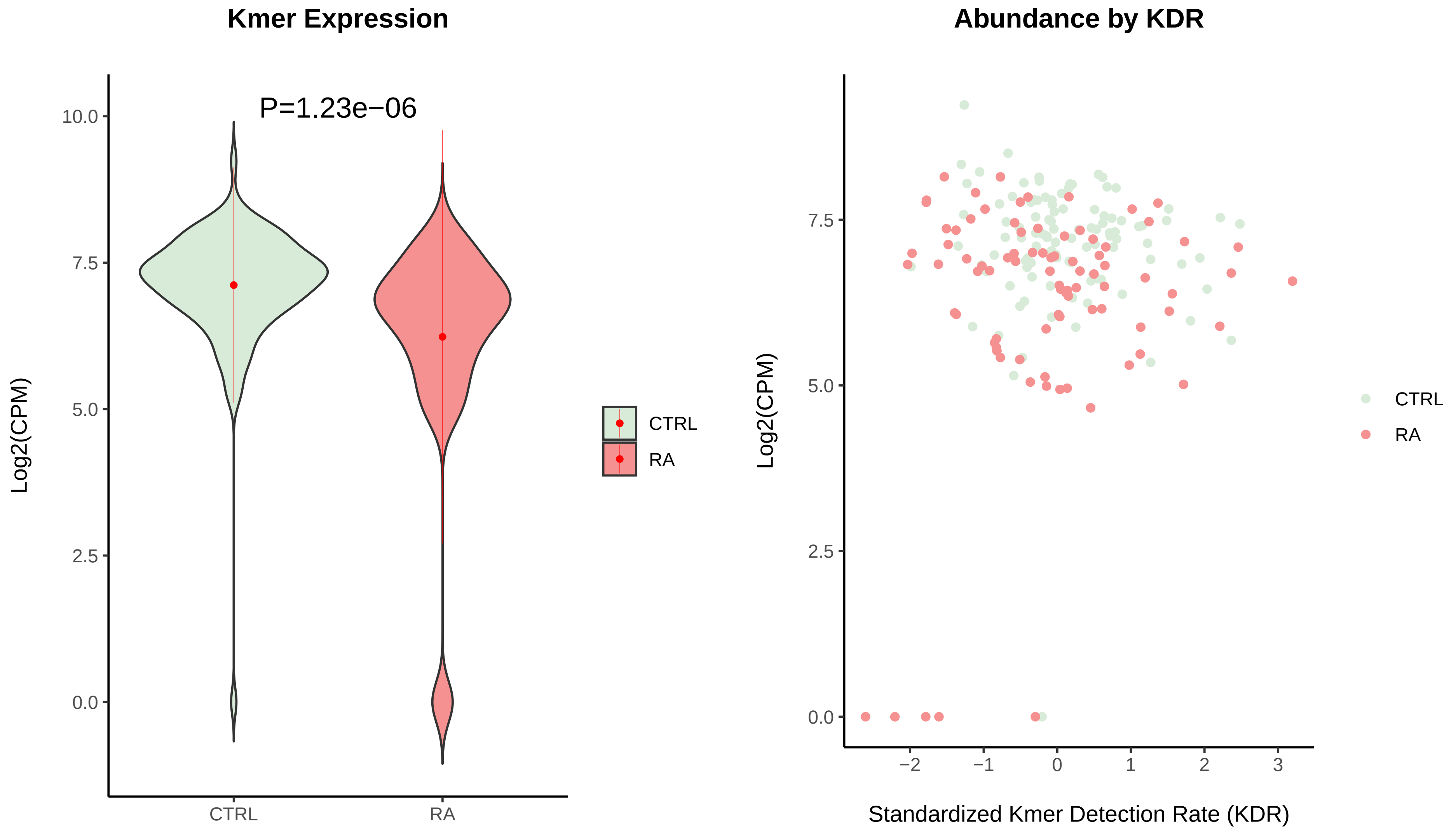

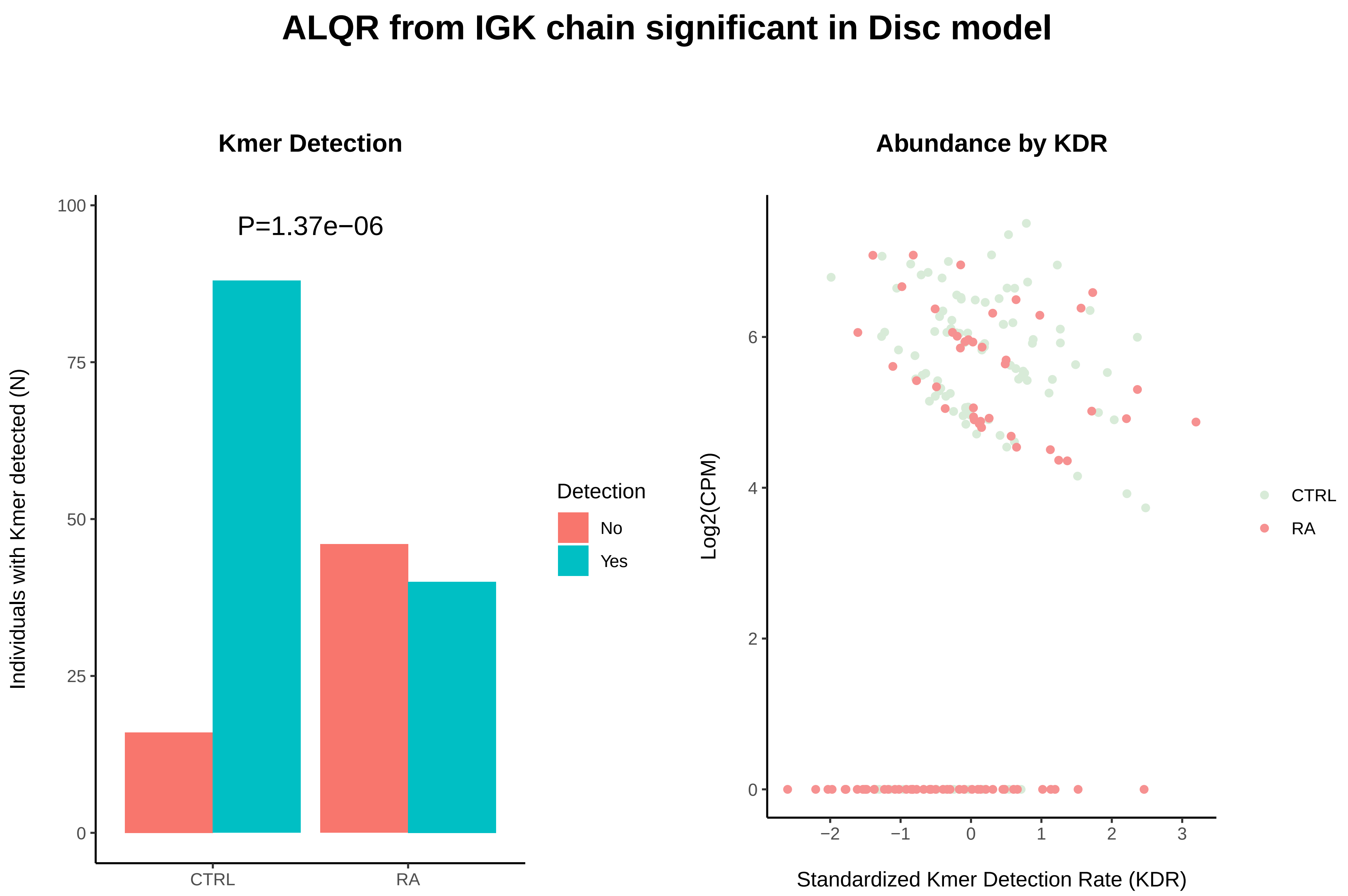
ALQR from IGK chain significant in Disc model
Kmer Detection
P=1.37e−06
Individuals with Kmer detected (N)
100
75
50
25
0
CTRL
RA
Detection
No
Yes
Abundance by KDR
Log2(CPM)
6
4
2
0
−2
−1
0
1
2
3
Standardized Kmer Detection Rate (KDR)
CTRL
RA

IQVP from IGK chain significant in Disc model

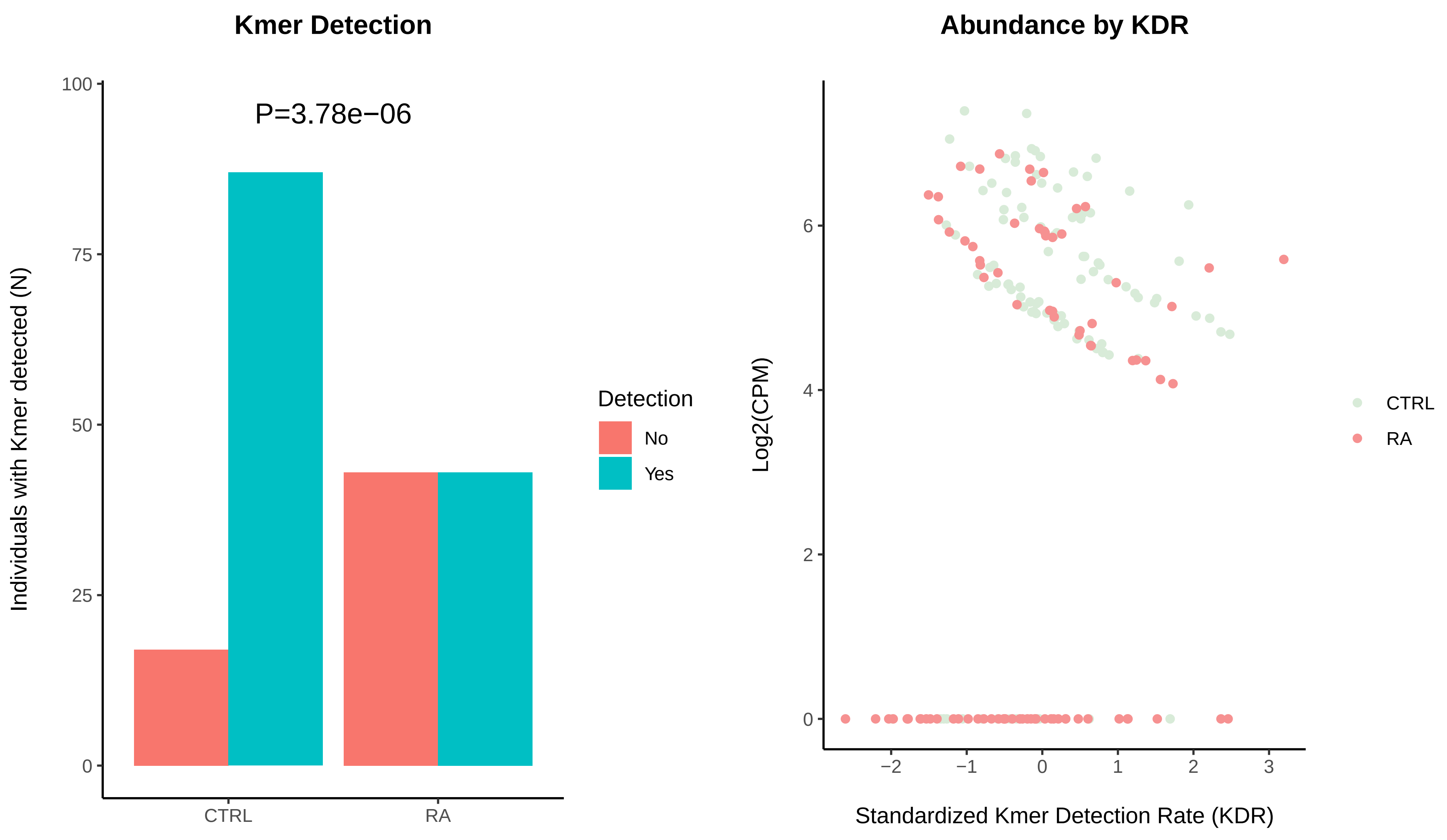

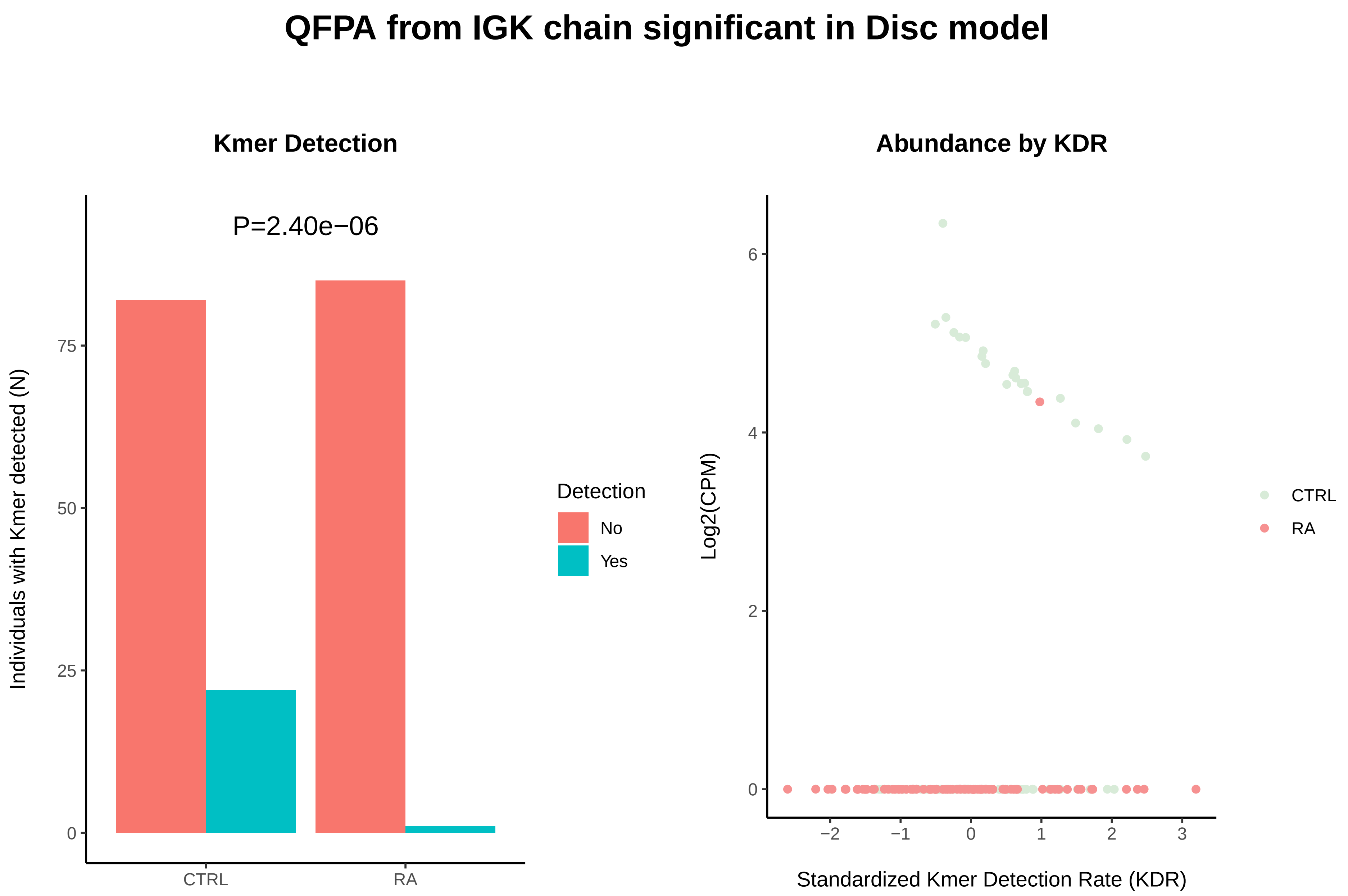

QFPA from IGK chain significant in Disc model
Kmer Detection
P=2.40e−06
Individuals with Kmer detected (N)
0
25
50
75
CTRL
RA
Detection
No
Yes
Abundance by KDR
Log2(CPM)
0
2
4
6
−2
−1
0
1
2
3
Standardized Kmer Detection Rate (KDR)
CTRL
RA

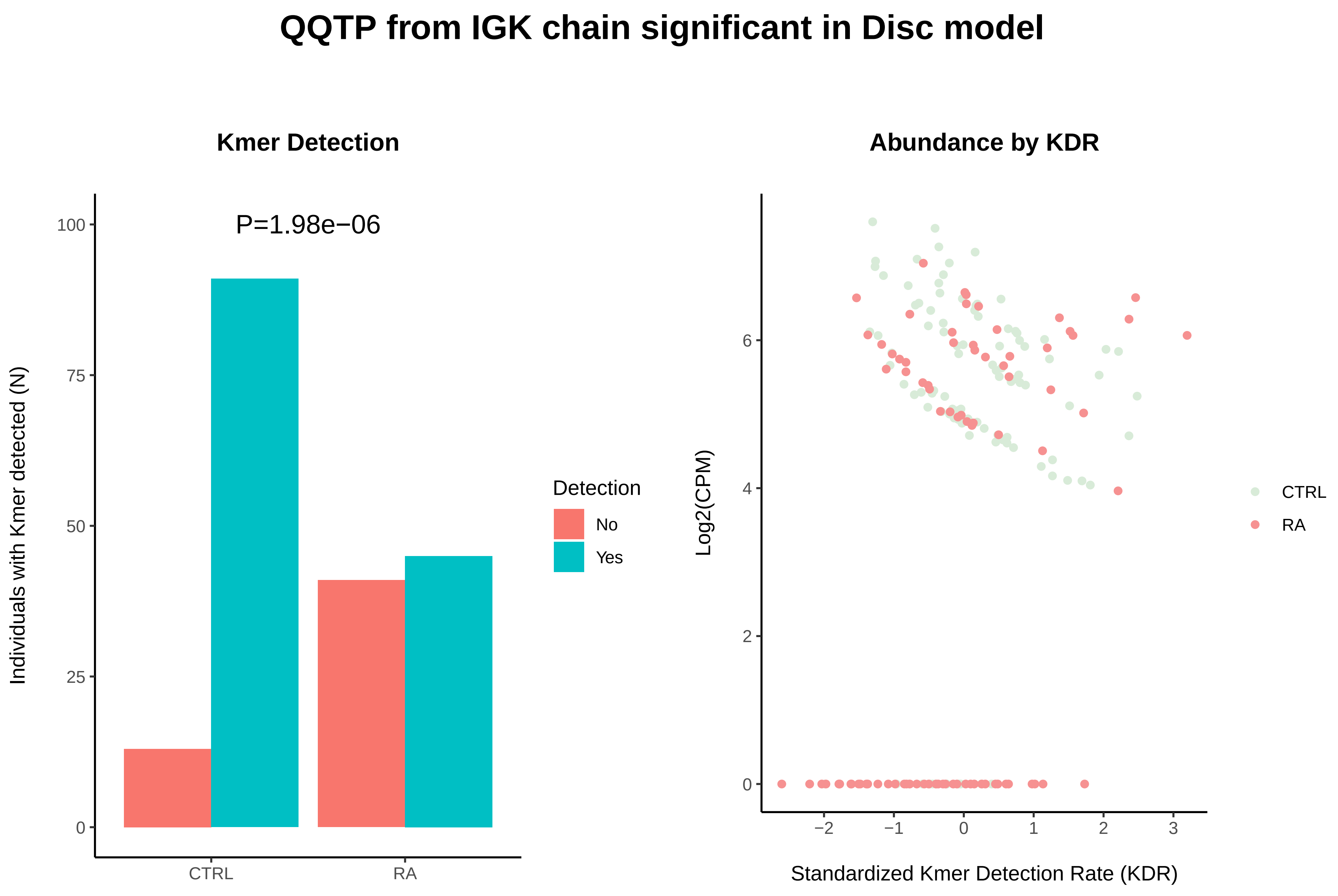
QQTP from IGK chain significant in Disc model
Kmer Detection
P=1.98e−06
Individuals with Kmer detected (N)
100
75
50
25
0
CTRL
RA
Detection
No
Yes
Abundance by KDR
Log2(CPM)
6
4
2
0
−2
−1
0
1
2
3
Standardized Kmer Detection Rate (KDR)
CTRL
RA

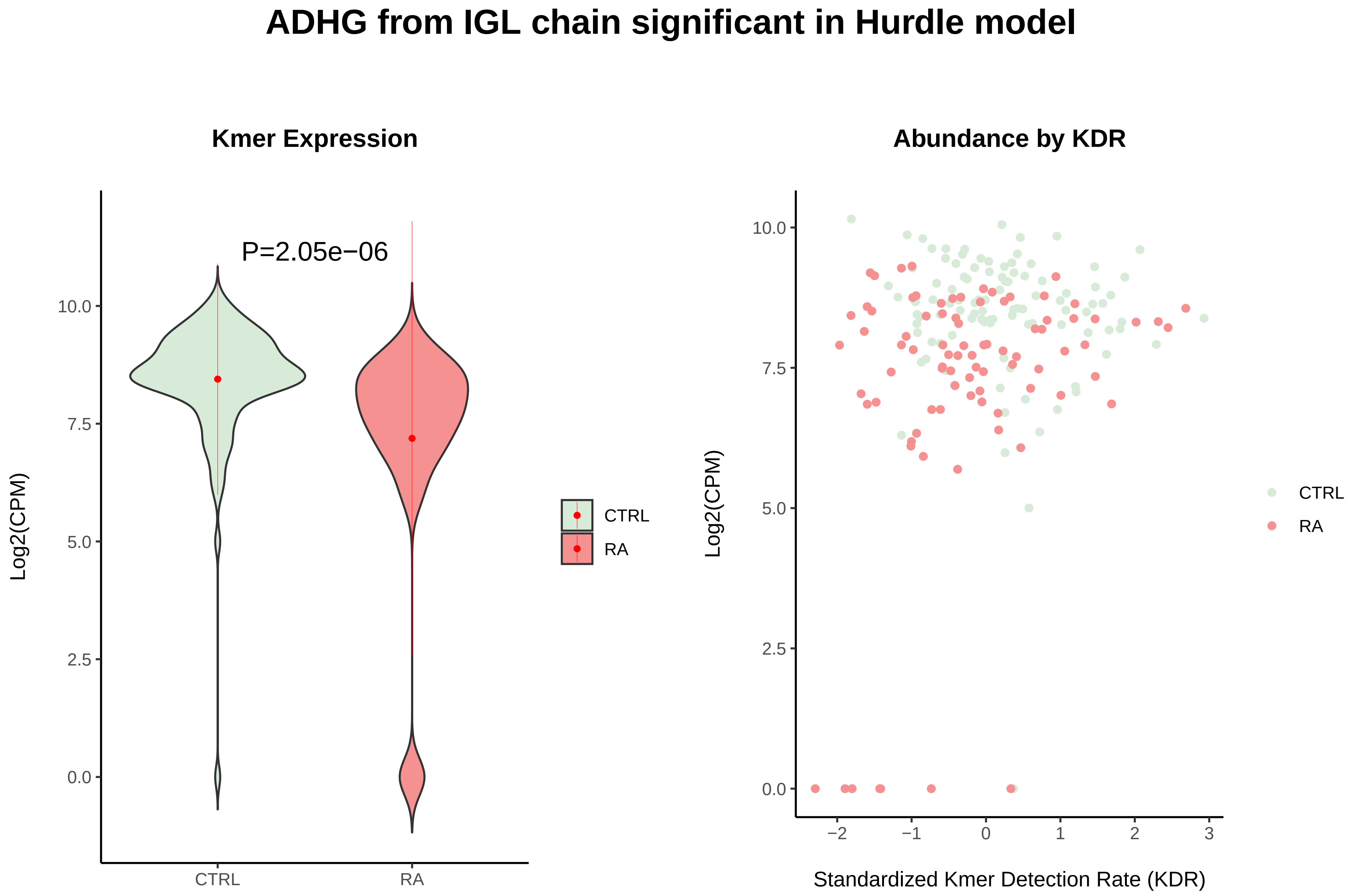
ADHG from IGL chain significant in Hurdle model
Kmer Expression
P=2.05e−06
Log2(CPM)
10.0
7.5
5.0
2.5
0.0
CTRL
RA
Abundance by KDR
Log2(CPM)
Standardized Kmer Detection Rate (KDR)
−2
−1
0
1
2
3

CFLS from IGL chain significant in Hurdle model

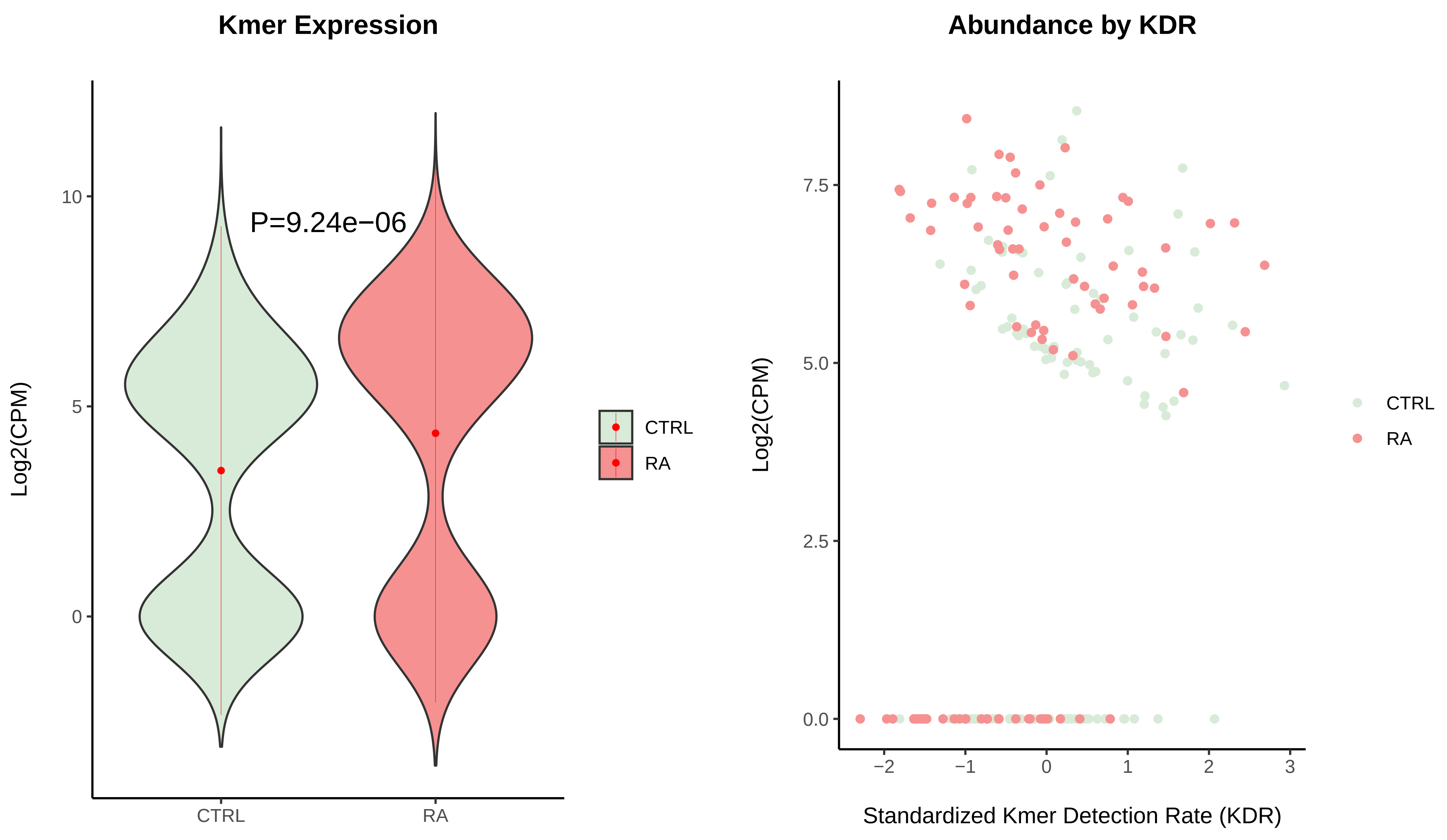

CGAD from IGL chain significant in Hurdle model

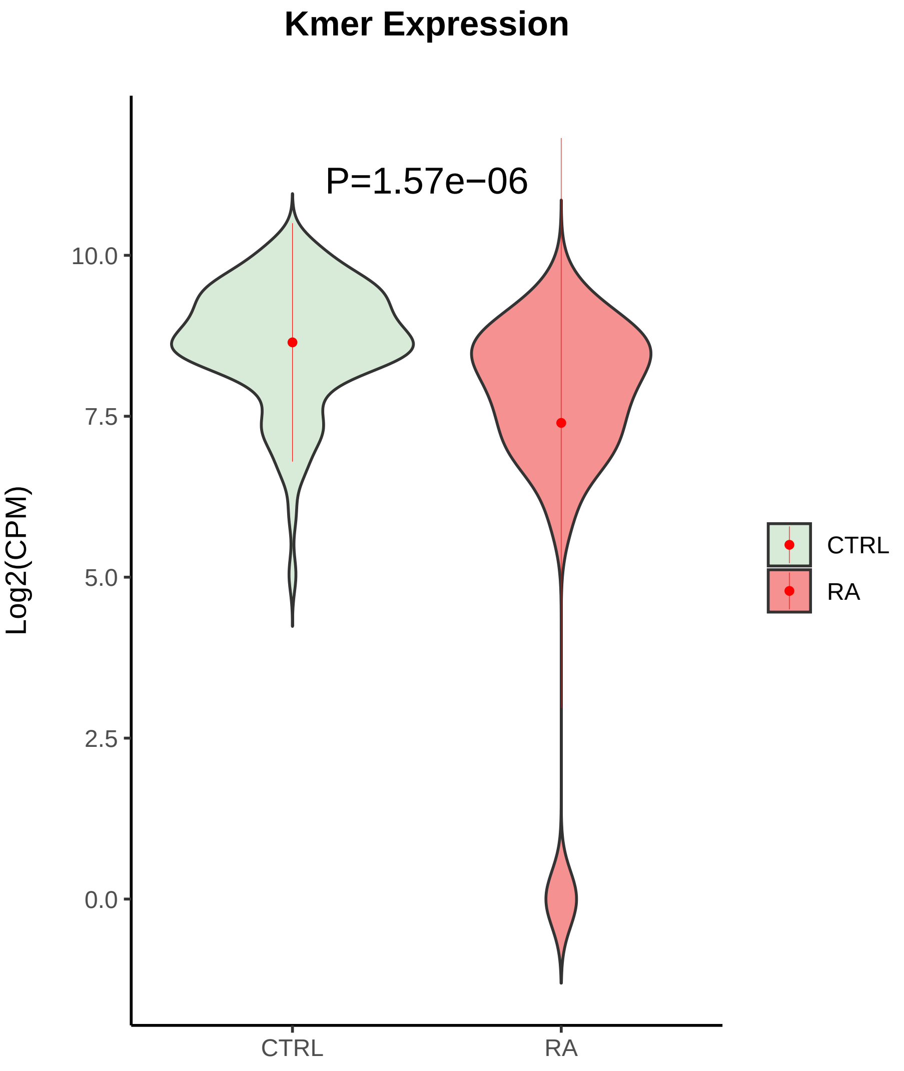

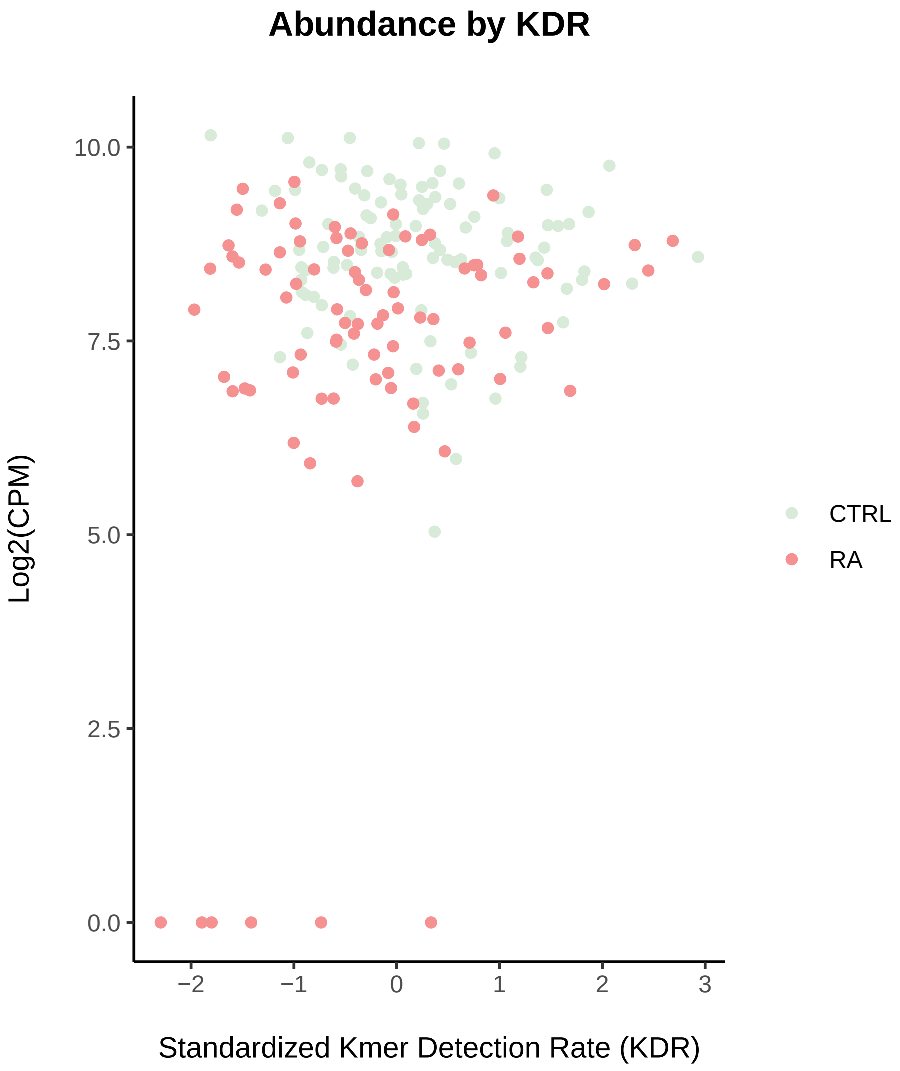

CGES from IGL chain significant in Hurdle model

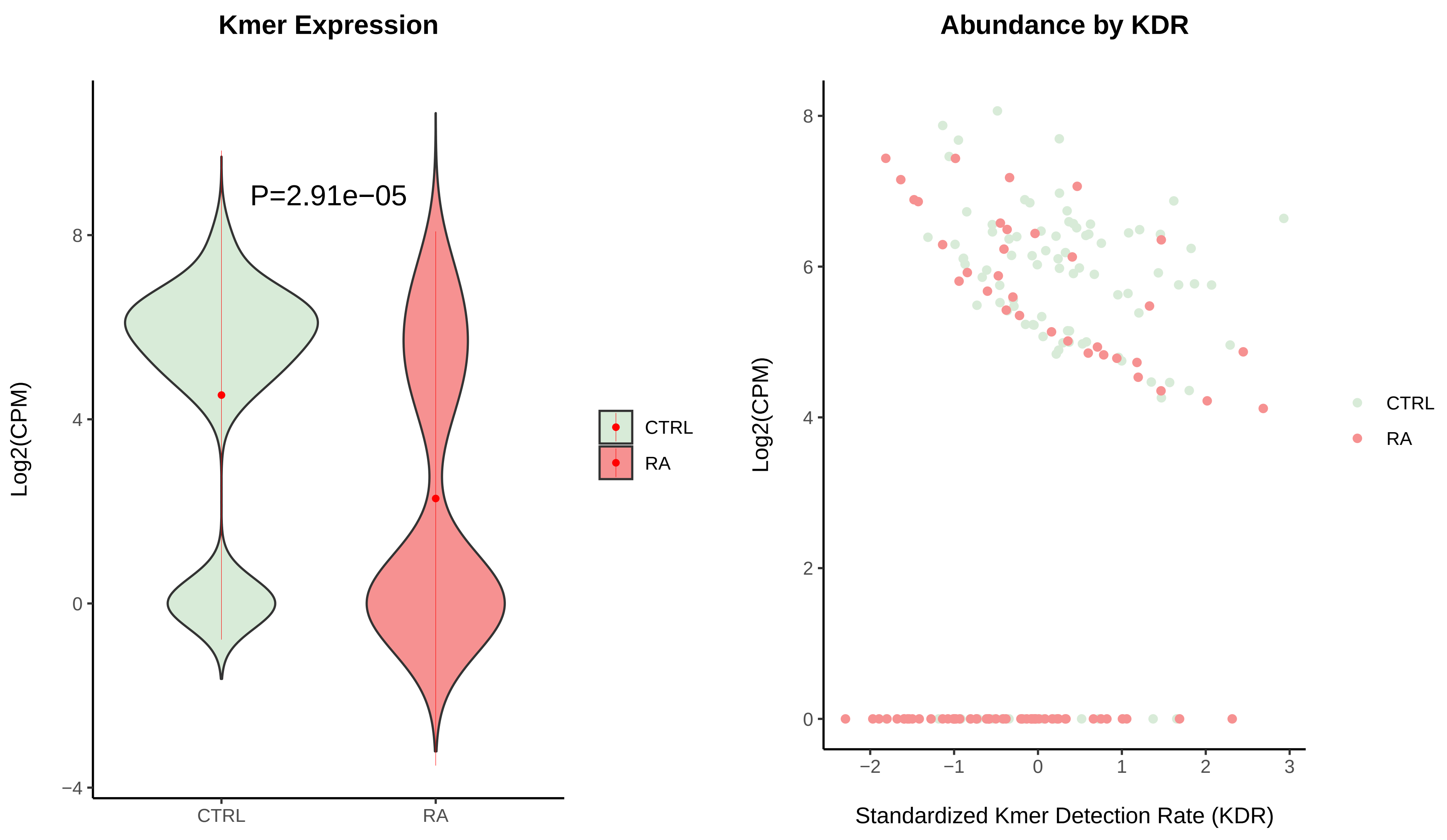

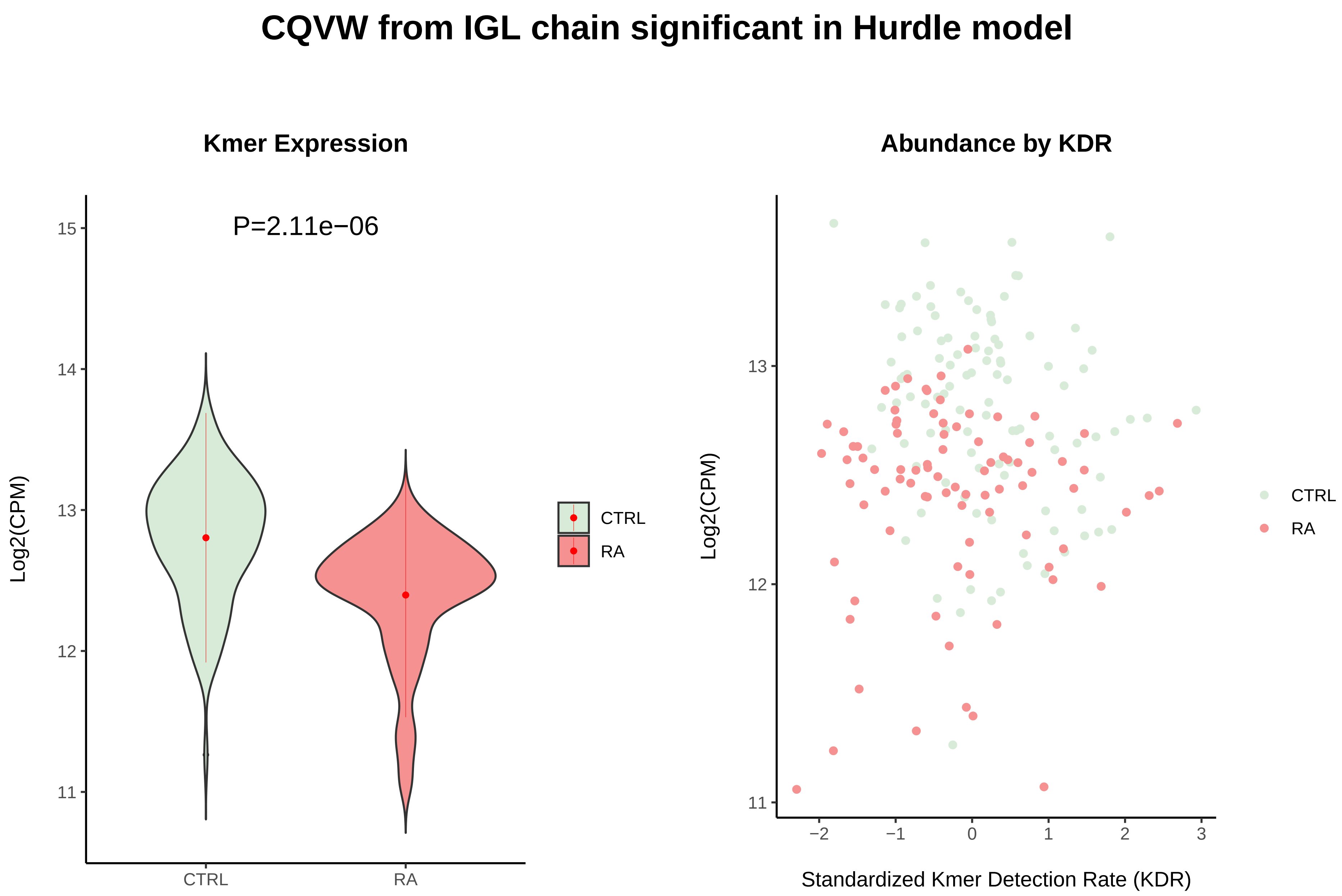
CQVW from IGL chain significant in Hurdle model
Kmer Expression
P=2.11e−06
Log2(CPM)
15
14
13
12
11
CTRL
RA
CTRL
RA
Abundance by KDR
Log2(CPM)
13
12
11
−2
−1
0
1
2
3
Standardized Kmer Detection Rate (KDR)
CTRL
RA

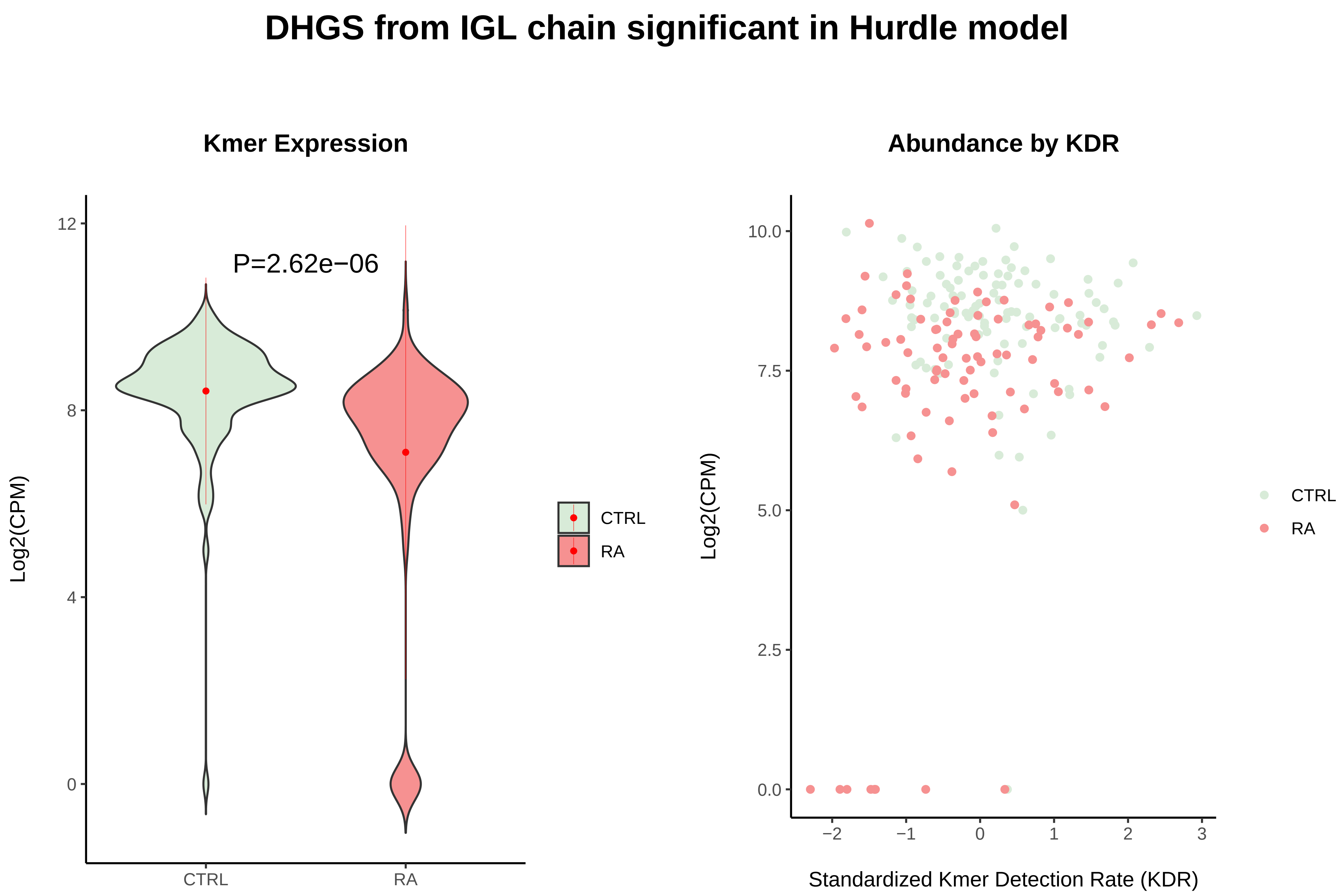
DHGS from IGL chain significant in Hurdle model
Kmer Expression
P=2.62e−06
Log2(CPM)
12
8
4
0
CTRL
RA
CTRL
RA
Abundance by KDR
Log2(CPM)
10.0
7.5
5.0
2.5
0.0
−2
−1
0
1
2
3
Standardized Kmer Detection Rate (KDR)
CTRL
RA

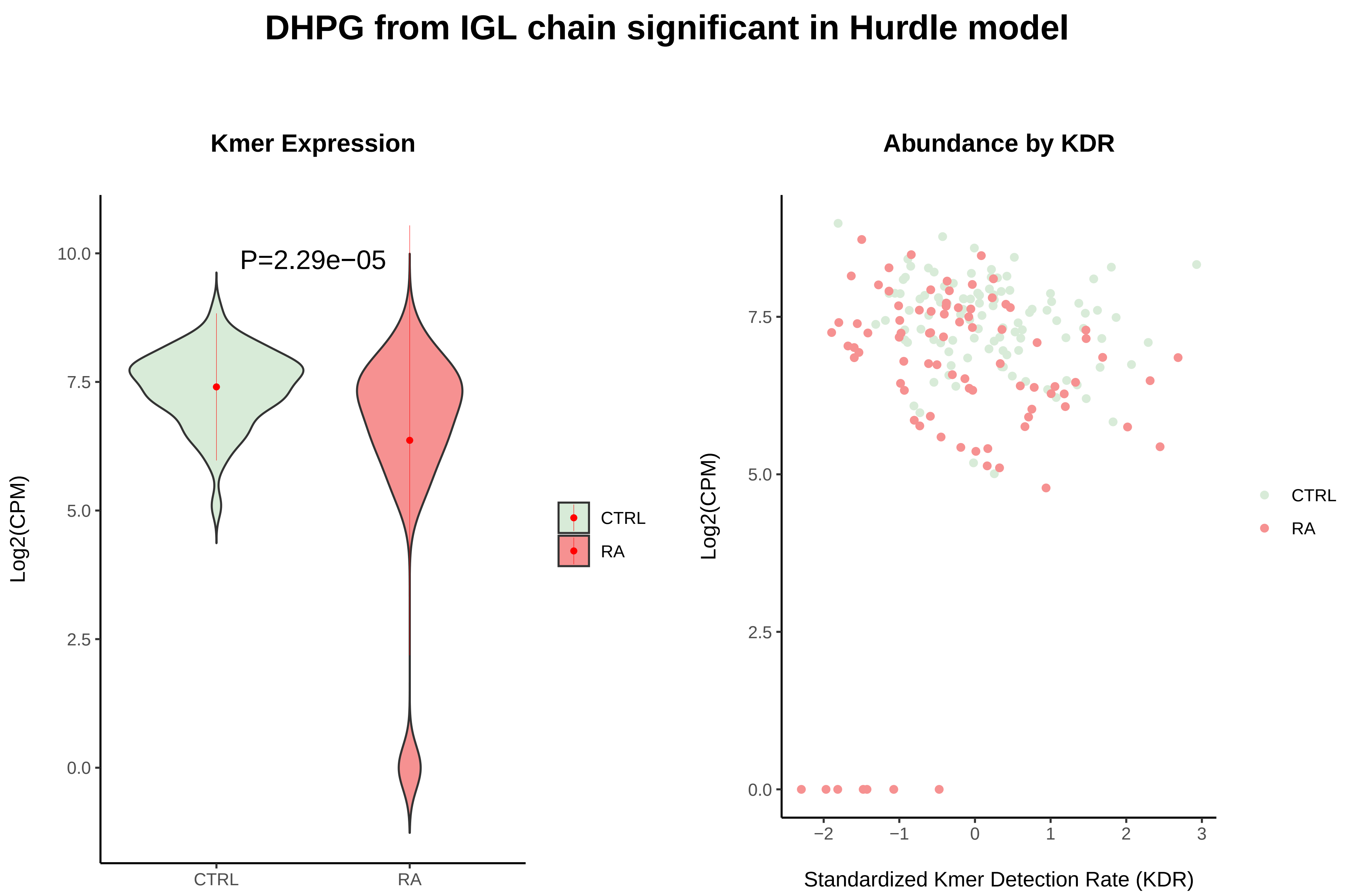
DHPG from IGL chain significant in Hurdle model
Kmer Expression
P=2.29e−05
Log2(CPM)
10.0
7.5
5.0
2.5
0.0
CTRL
RA
Abundance by KDR
Log2(CPM)
Standardized Kmer Detection Rate (KDR)
−2
−1
0
1
2
3

DHRE from IGL chain significant in Hurdle model

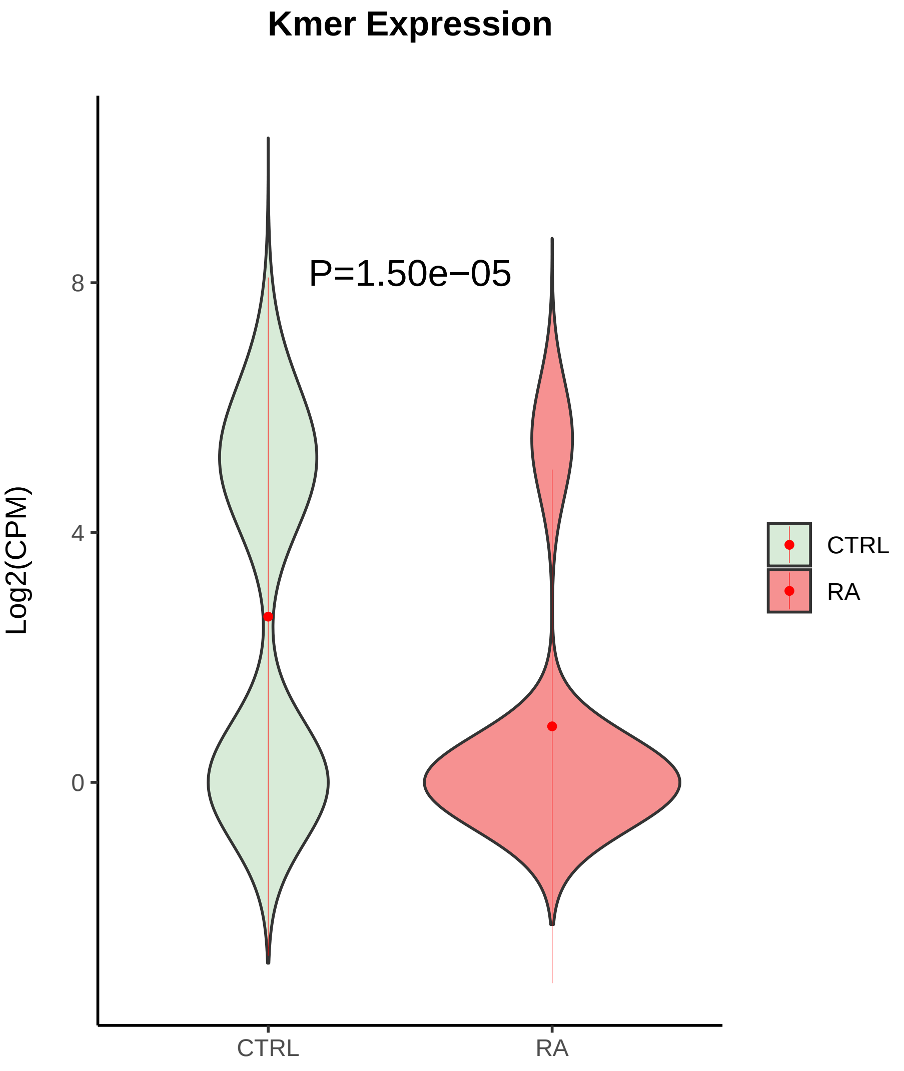

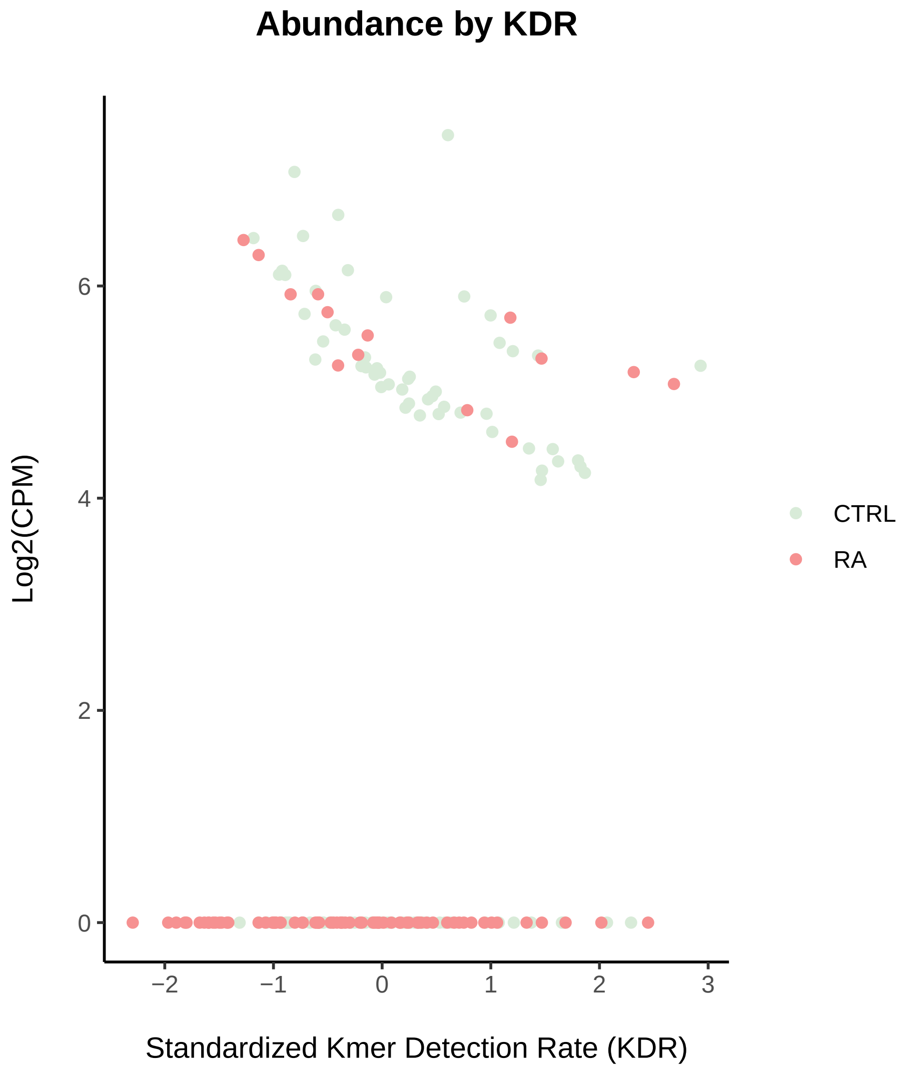

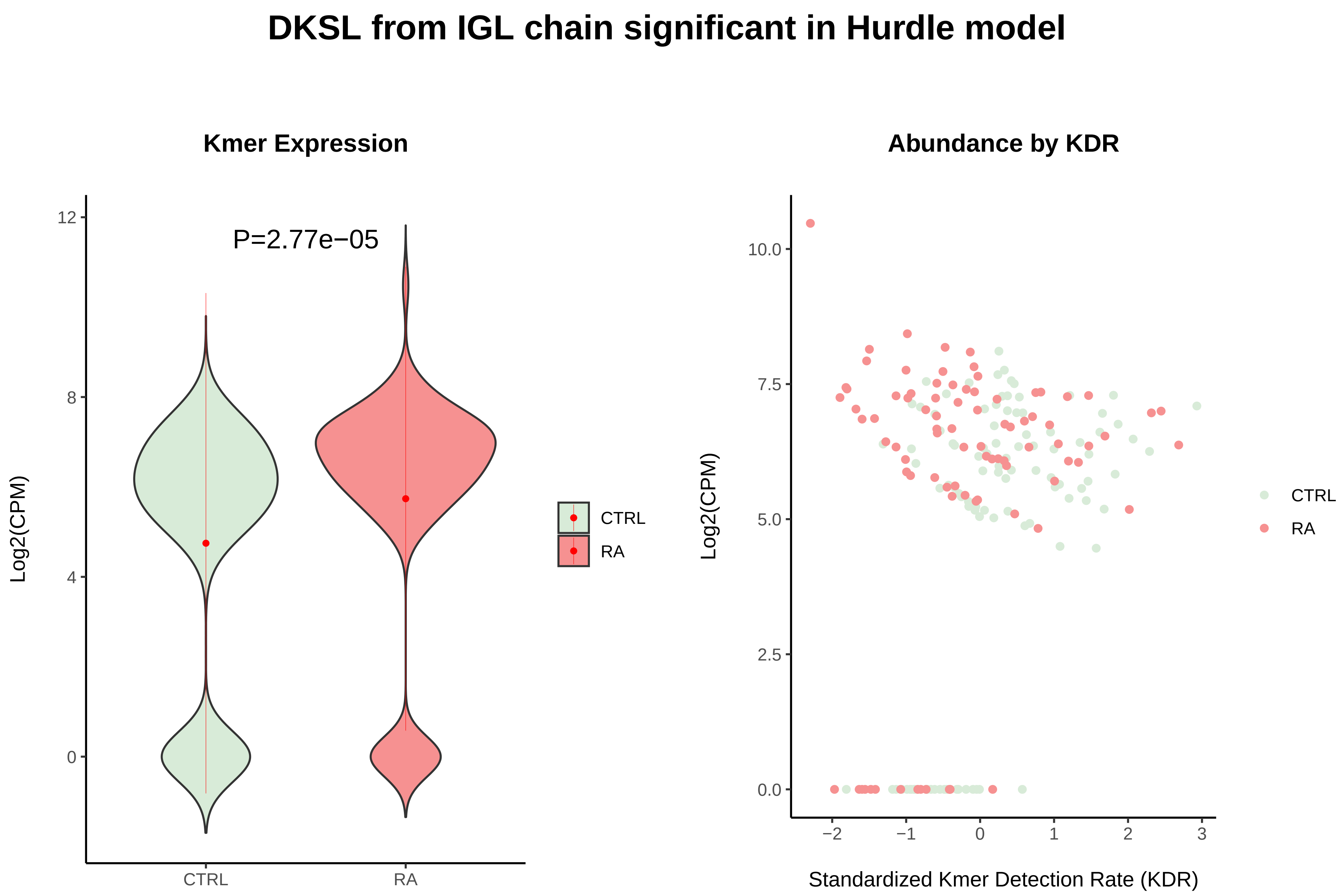
DKSL from IGL chain significant in Hurdle model
Kmer Expression
P=2.77e−05
Log2(CPM)
12
8
4
0
CTRL
RA
CTRL
RA
Abundance by KDR
Log2(CPM)
10.0
7.5
5.0
2.5
0.0
−2
−1
0
1
2
3
Standardized Kmer Detection Rate (KDR)
CTRL
RA

DQEV from IGL chain significant in Hurdle model

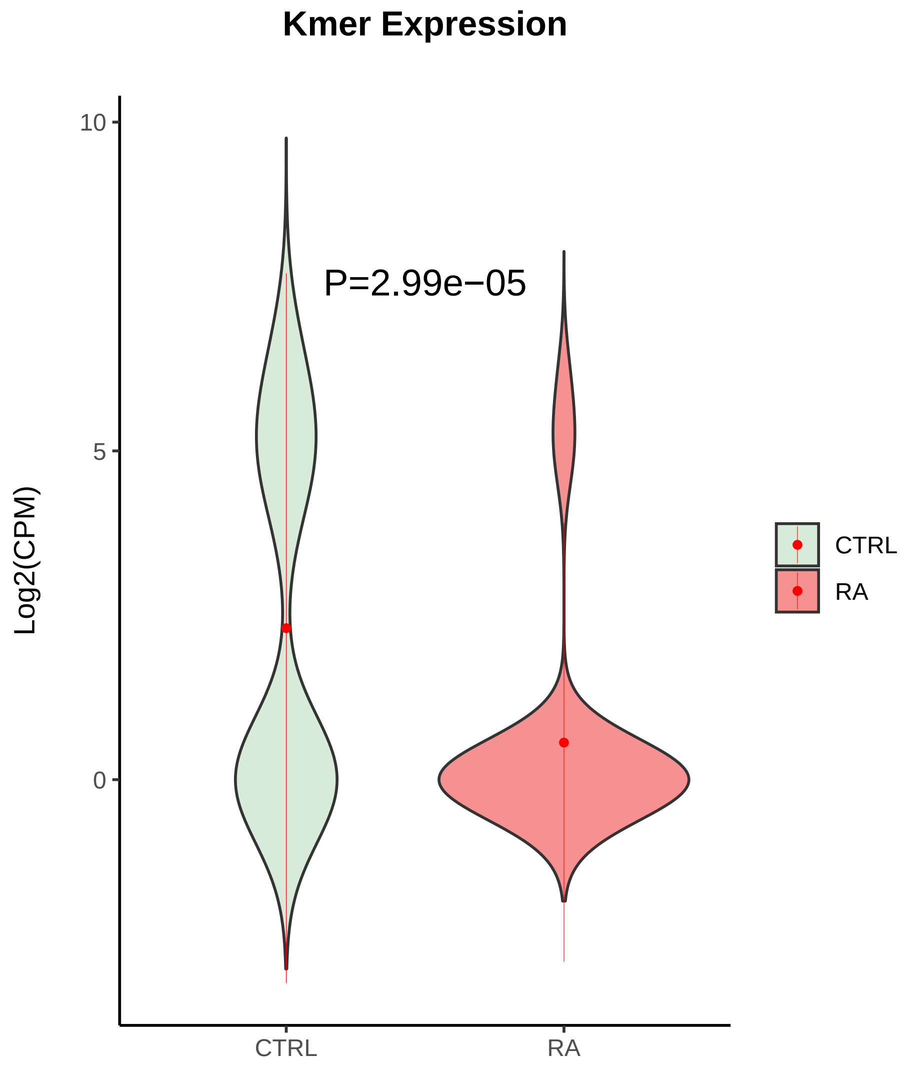

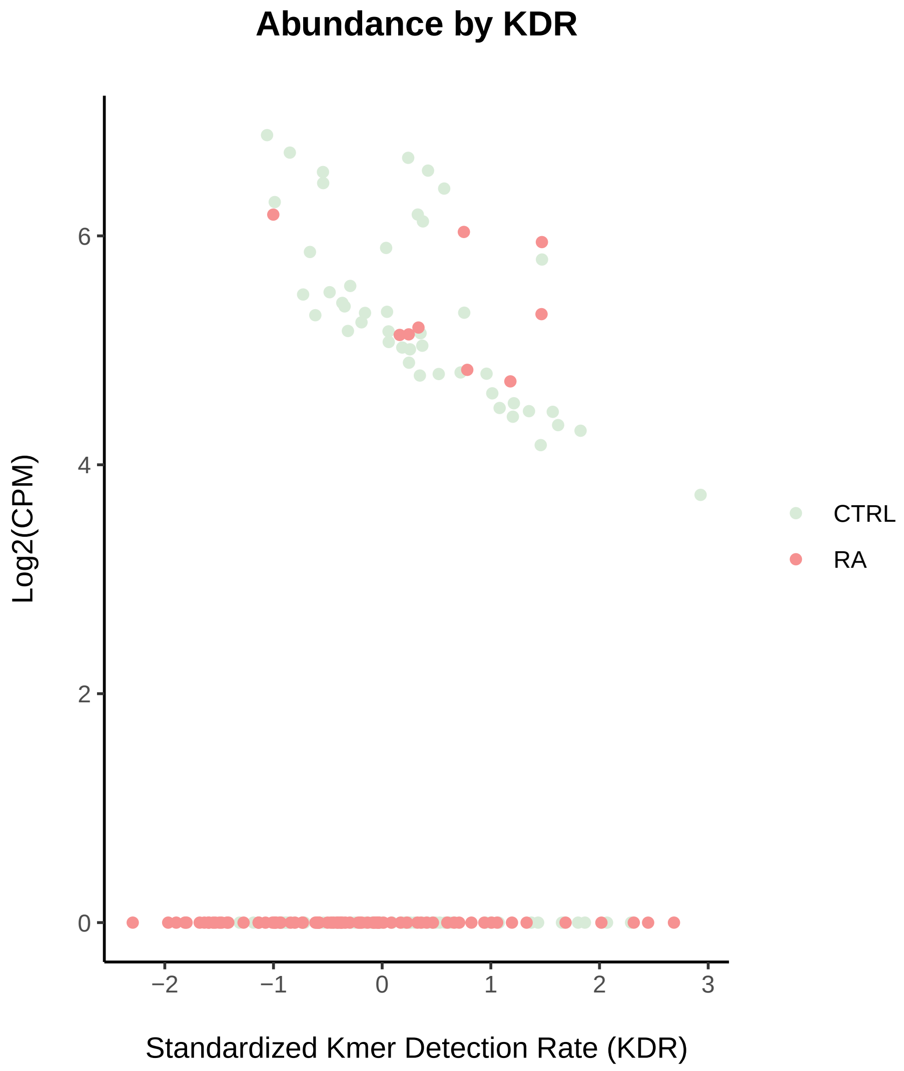

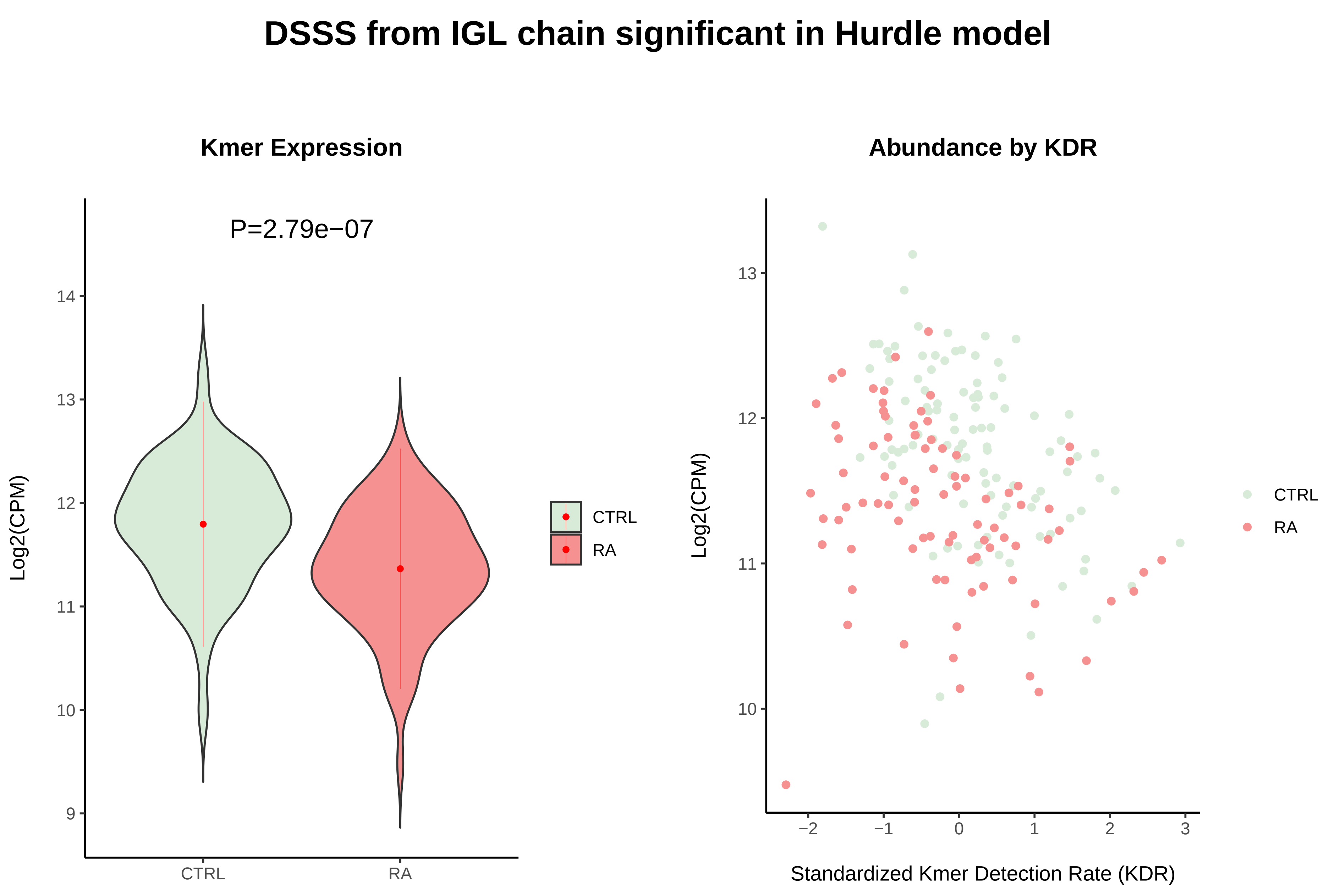
DSSS from IGL chain significant in Hurdle model
Kmer Expression
P=2.79e−07
Log2(CPM)
14
13
12
11
10
9
CTRL
RA
CTRL
RA
Abundance by KDR
Log2(CPM)
13
12
11
10
−2
−1
0
1
2
3
Standardized Kmer Detection Rate (KDR)
CTRL
RA

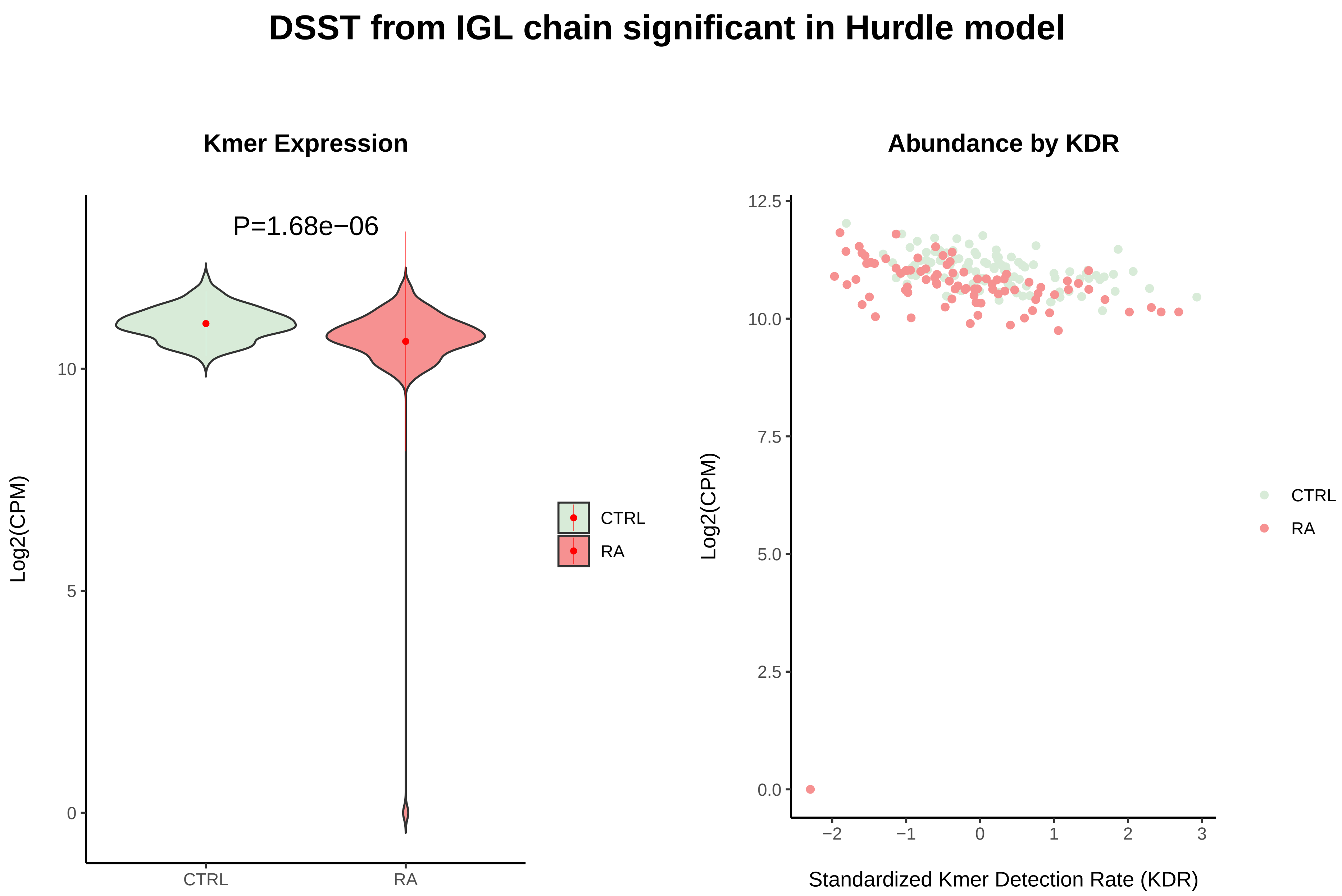
DSST from IGL chain significant in Hurdle model
Kmer Expression
P=1.68e−06
Log2(CPM)
0
5
10
CTRL
RA
CTRL
RA
Abundance by KDR
Log2(CPM)
0.0
2.5
5.0
7.5
10.0
12.5
−2
−1
0
1
2
3
Standardized Kmer Detection Rate (KDR)
CTRL
RA

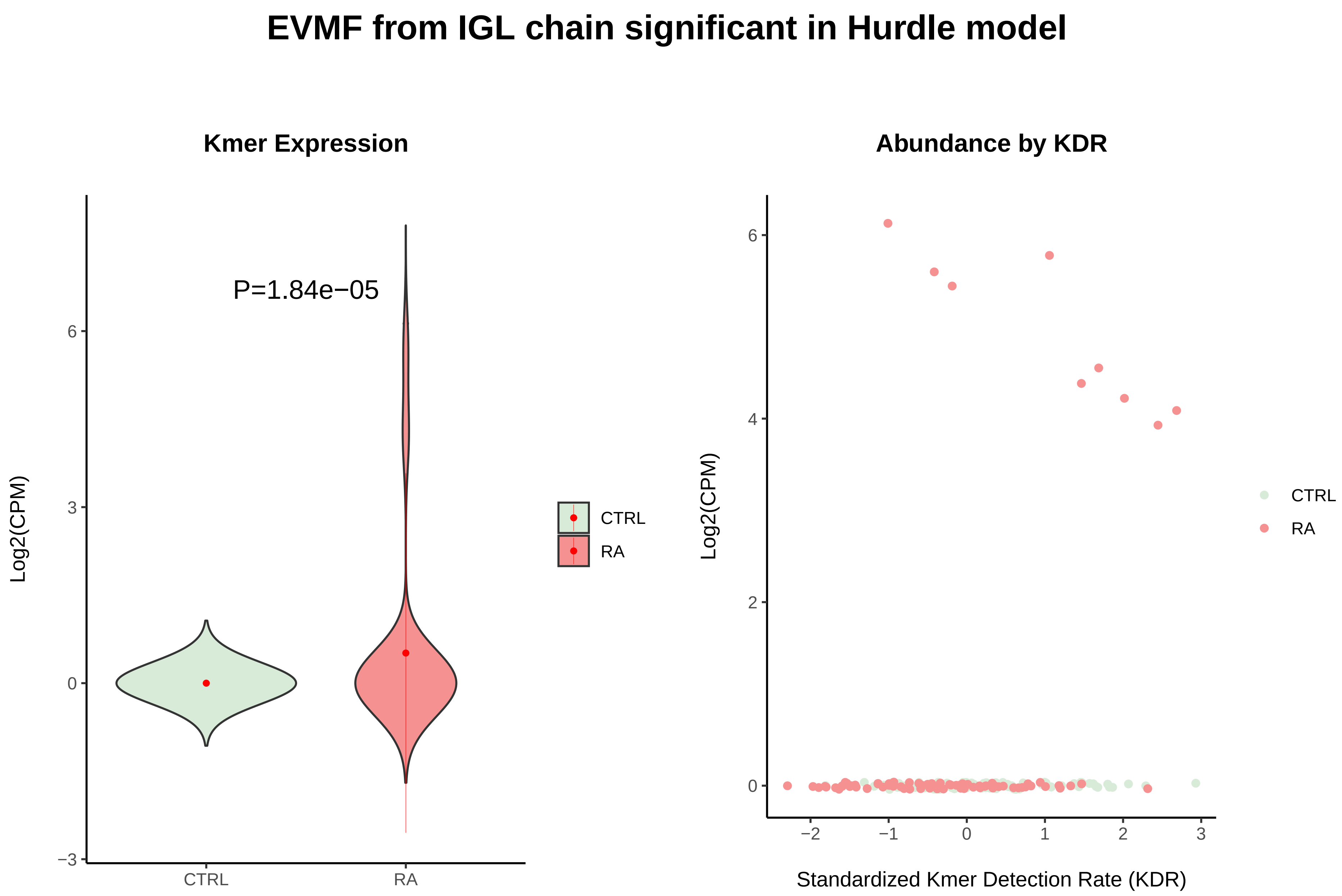
EVMF from IGL chain significant in Hurdle model
Kmer Expression
P=1.84e−05
Log2(CPM)
6
3
0
−3
CTRL
RA
CTRL
RA
Abundance by KDR
Log2(CPM)
6
4
2
0
−2
−1
0
1
2
3
Standardized Kmer Detection Rate (KDR)
CTRL
RA

GADH from IGL chain significant in Hurdle model

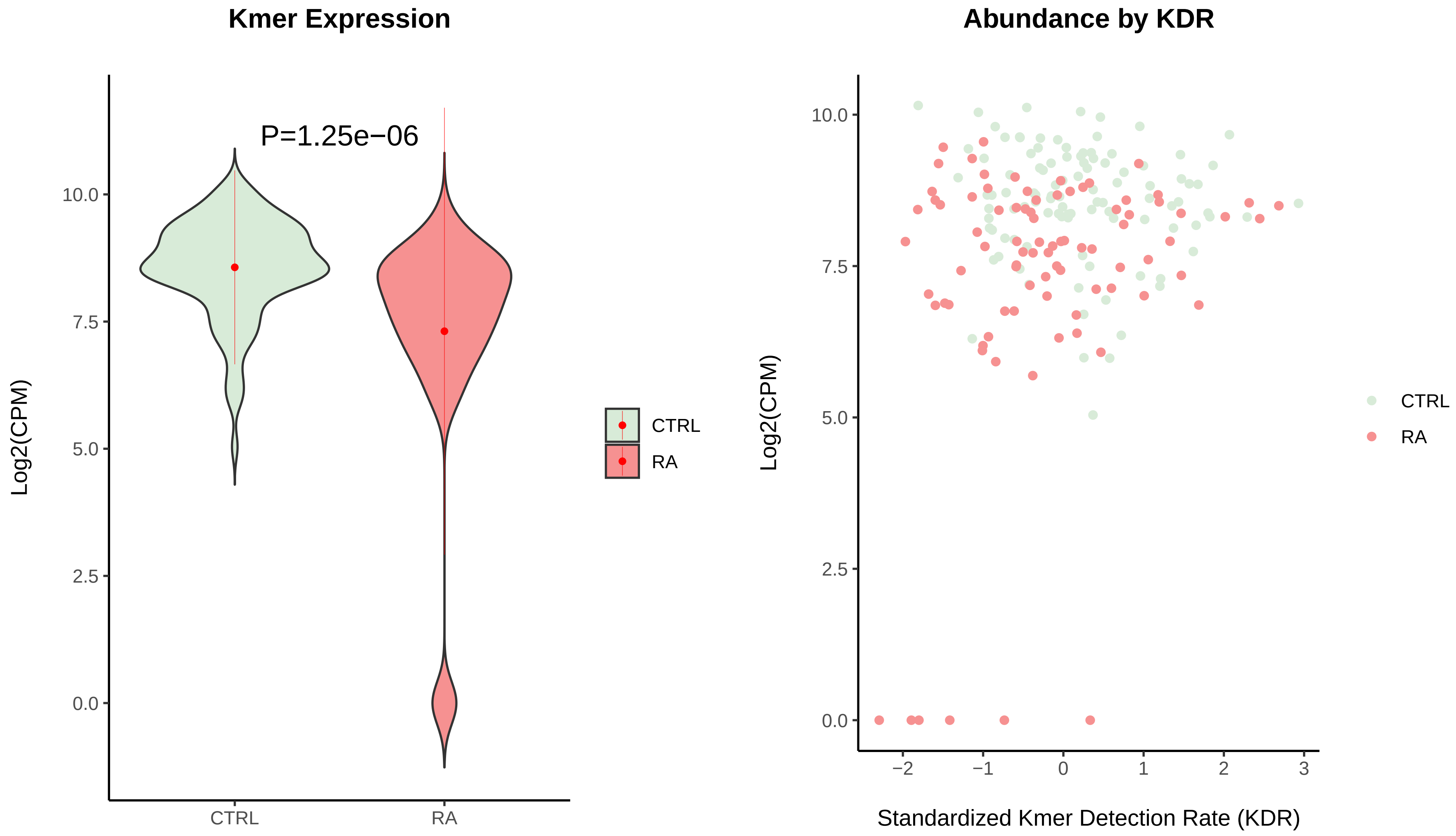

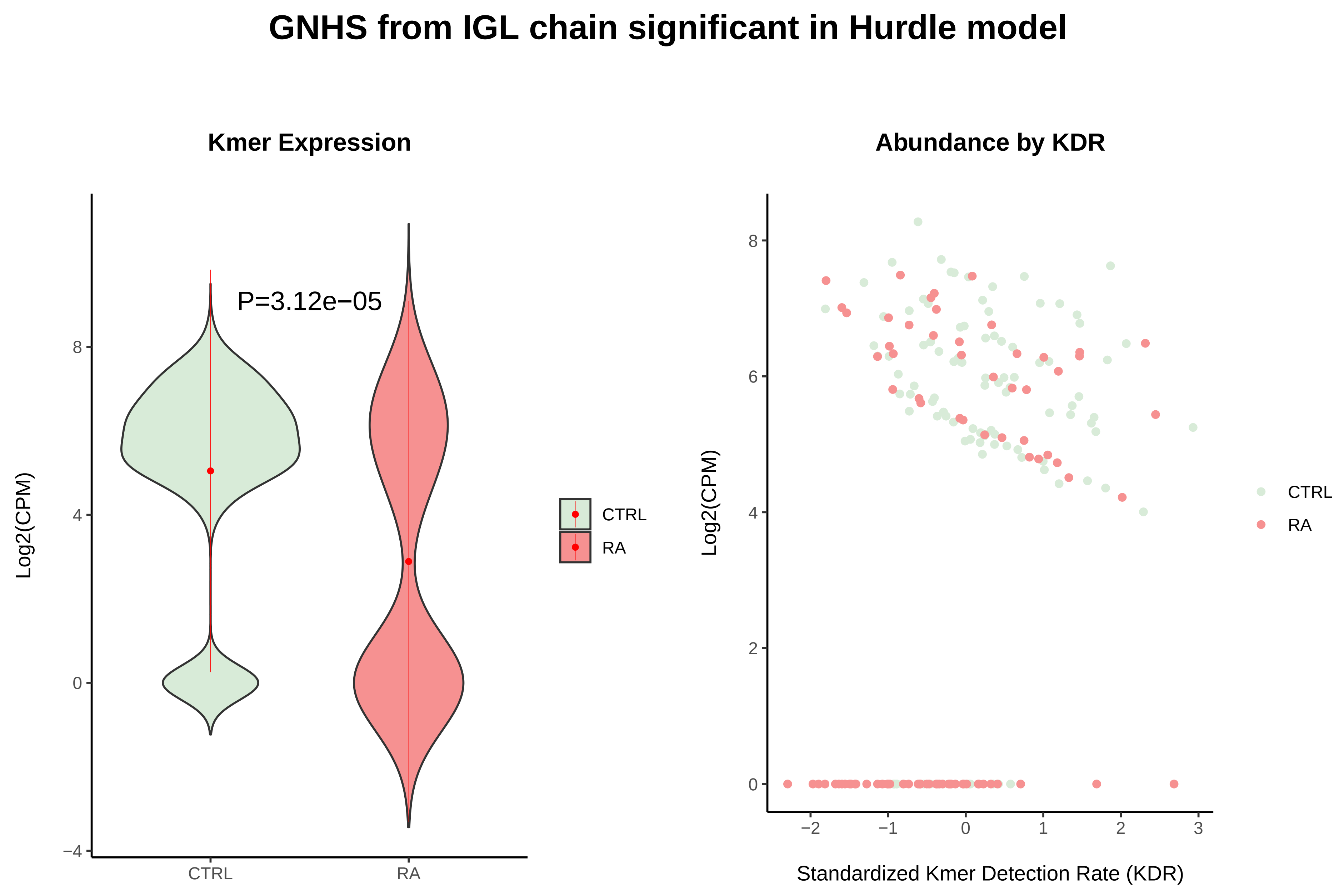
GNHS from IGL chain significant in Hurdle model
Kmer Expression
P=3.12e−05
Log2(CPM)
8
4
0
−4
CTRL
RA
CTRL
RA
Abundance by KDR
Log2(CPM)
8
6
4
2
0
−2
−1
0
1
2
3
Standardized Kmer Detection Rate (KDR)
CTRL
RA

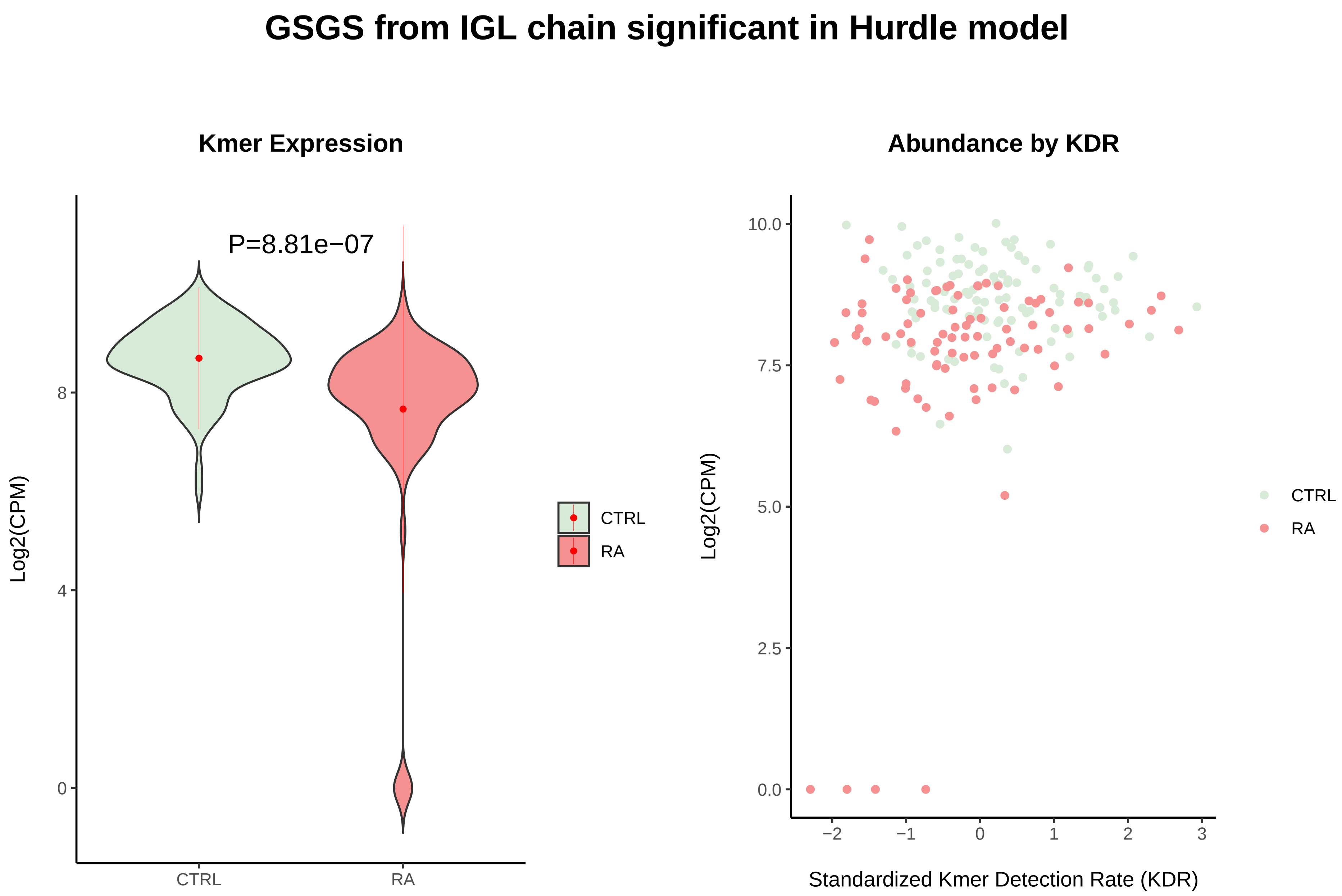
GSGS from IGL chain significant in Hurdle model
Kmer Expression
P=8.81e−07
Log2(CPM)
0
4
8
CTRL
RA
CTRL
RA
Abundance by KDR
Log2(CPM)
0.0
2.5
5.0
7.5
10.0
−2
−1
0
1
2
3
Standardized Kmer Detection Rate (KDR)
CTRL
RA

GSNF from IGL chain significant in Hurdle model

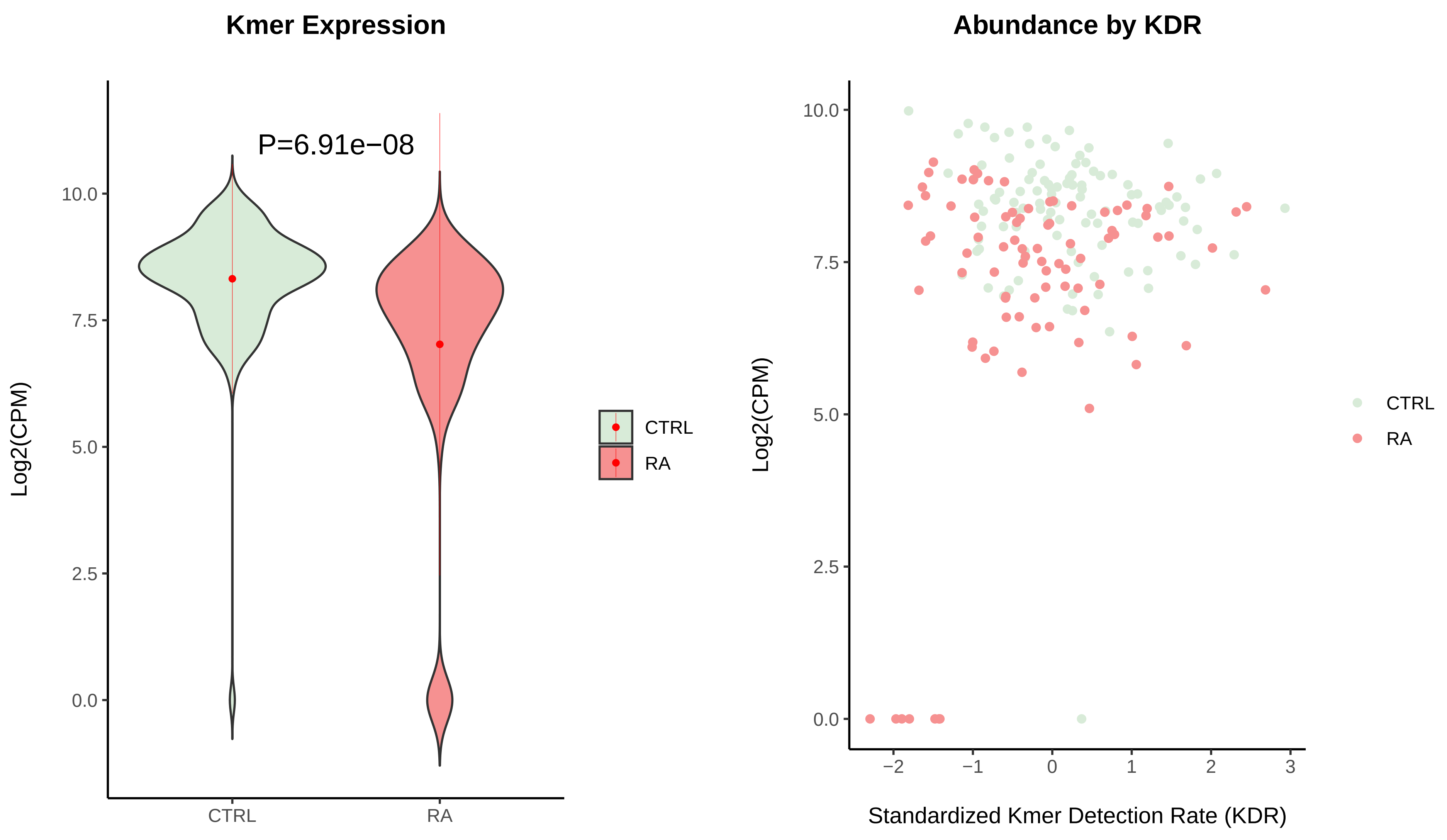

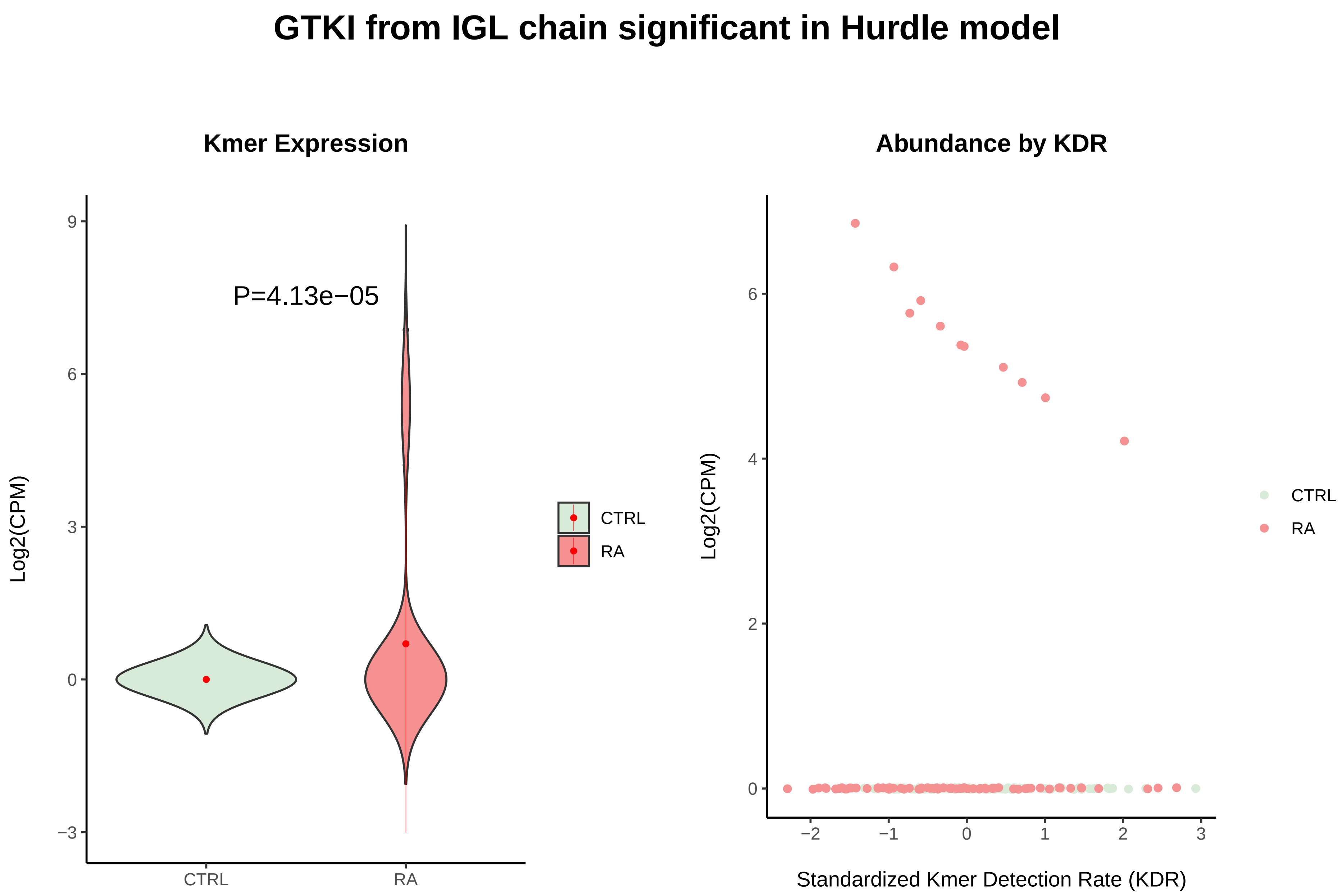
GTKI from IGL chain significant in Hurdle model
Kmer Expression
P=4.13e−05
Log2(CPM)
9
6
3
0
−3
CTRL
RA
CTRL
RA
Abundance by KDR
Log2(CPM)
6
4
2
0
−2
−1
0
1
2
3
Standardized Kmer Detection Rate (KDR)
CTRL
RA

HGSG from IGL chain significant in Hurdle model

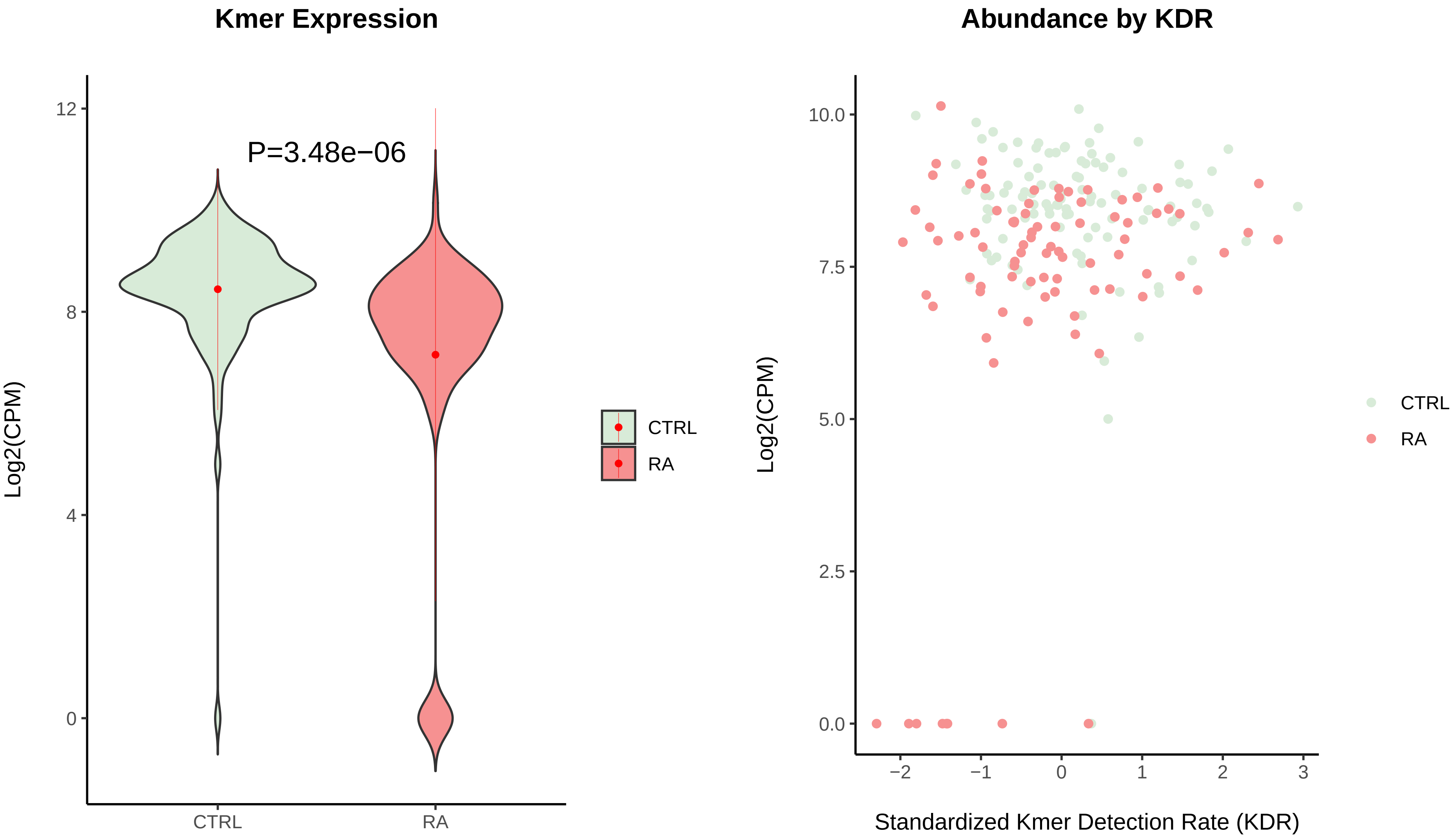

HTID from IGL chain significant in Hurdle model

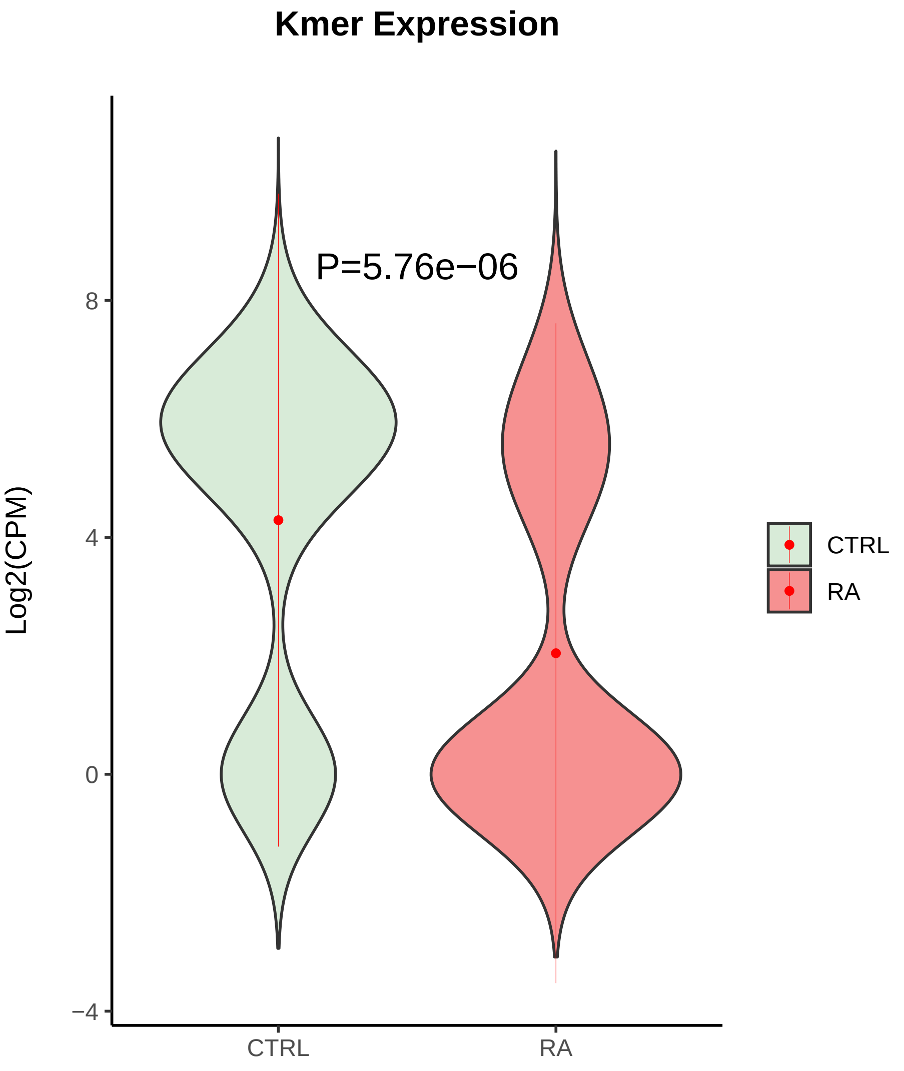

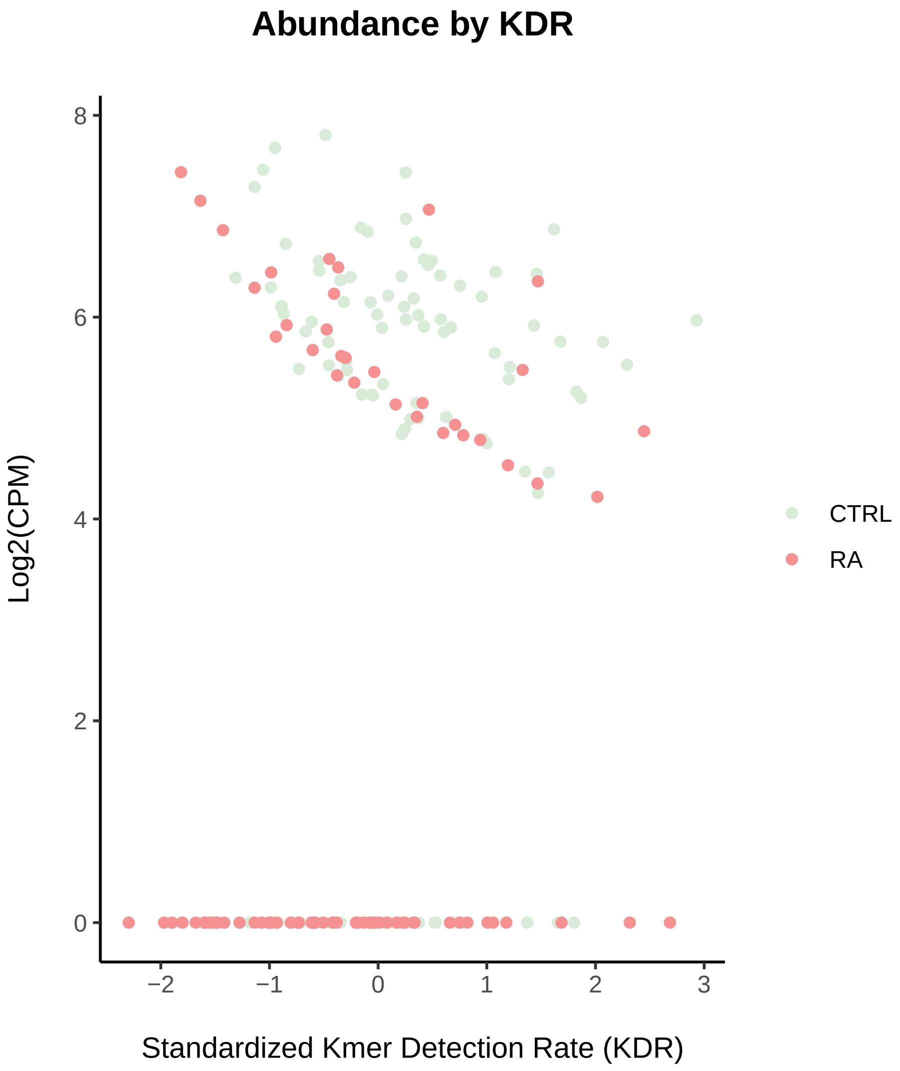

IDGQ from IGL chain significant in Hurdle model

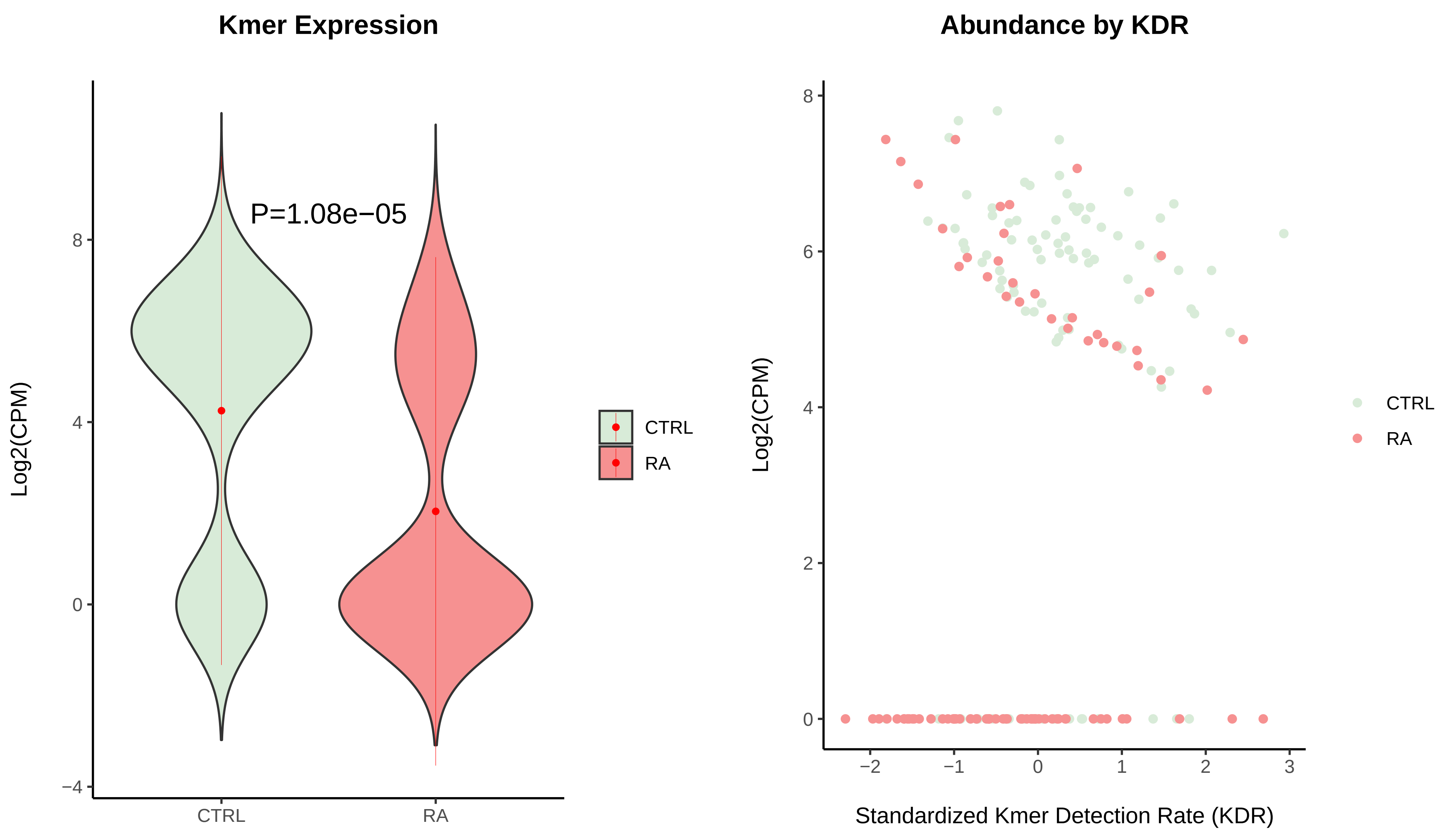

LLSY from IGL chain significant in Hurdle model

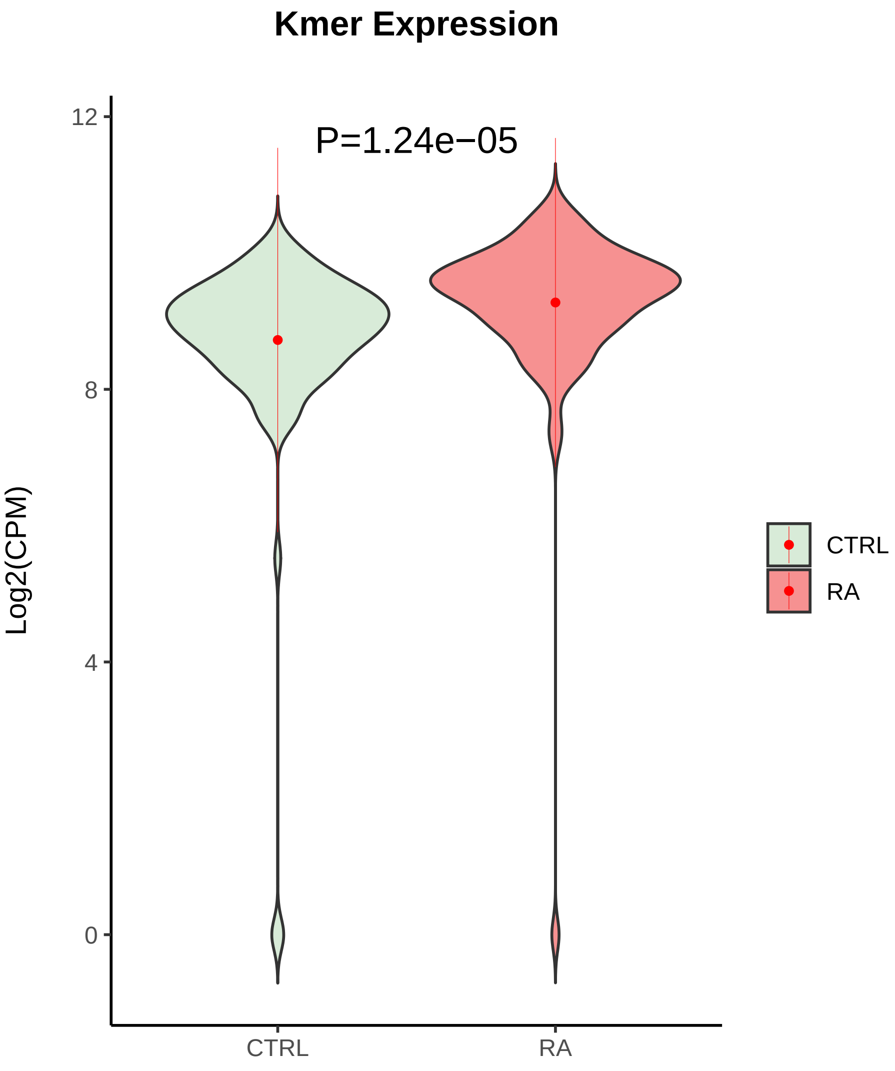

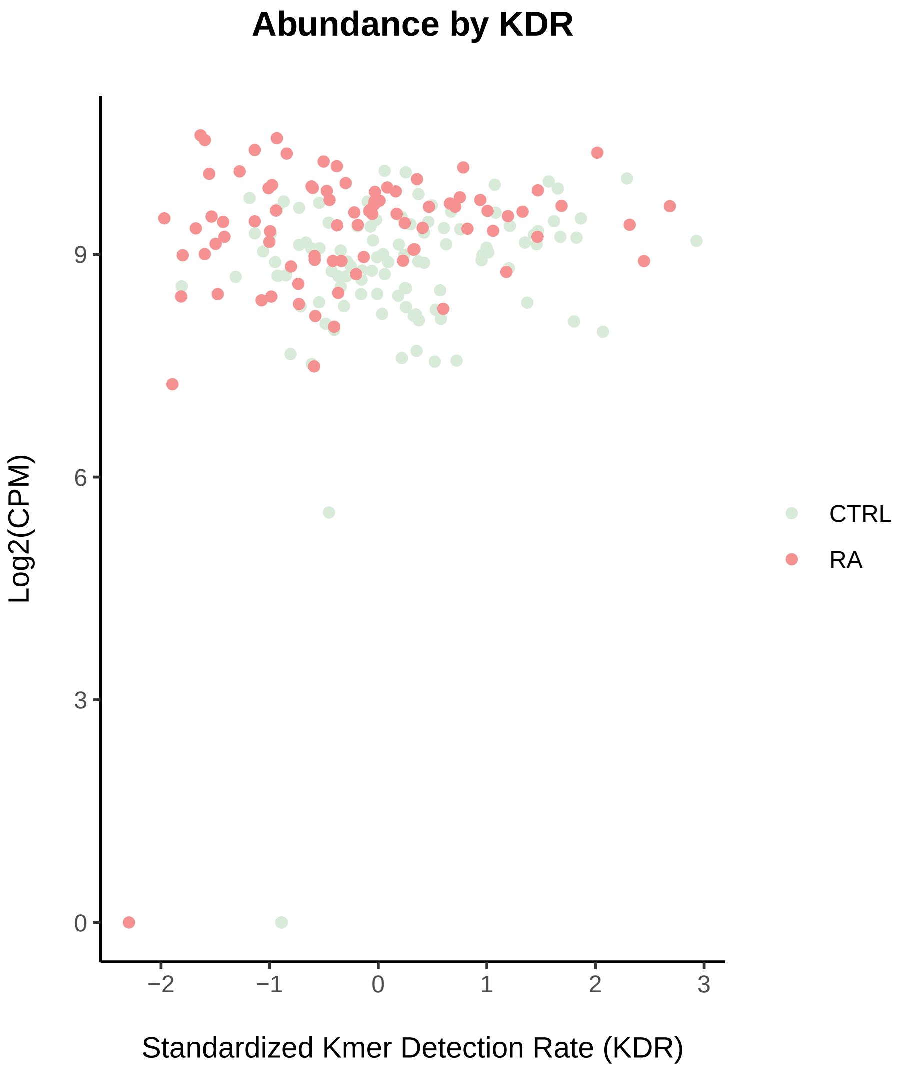

LSYT from IGL chain significant in Hurdle model

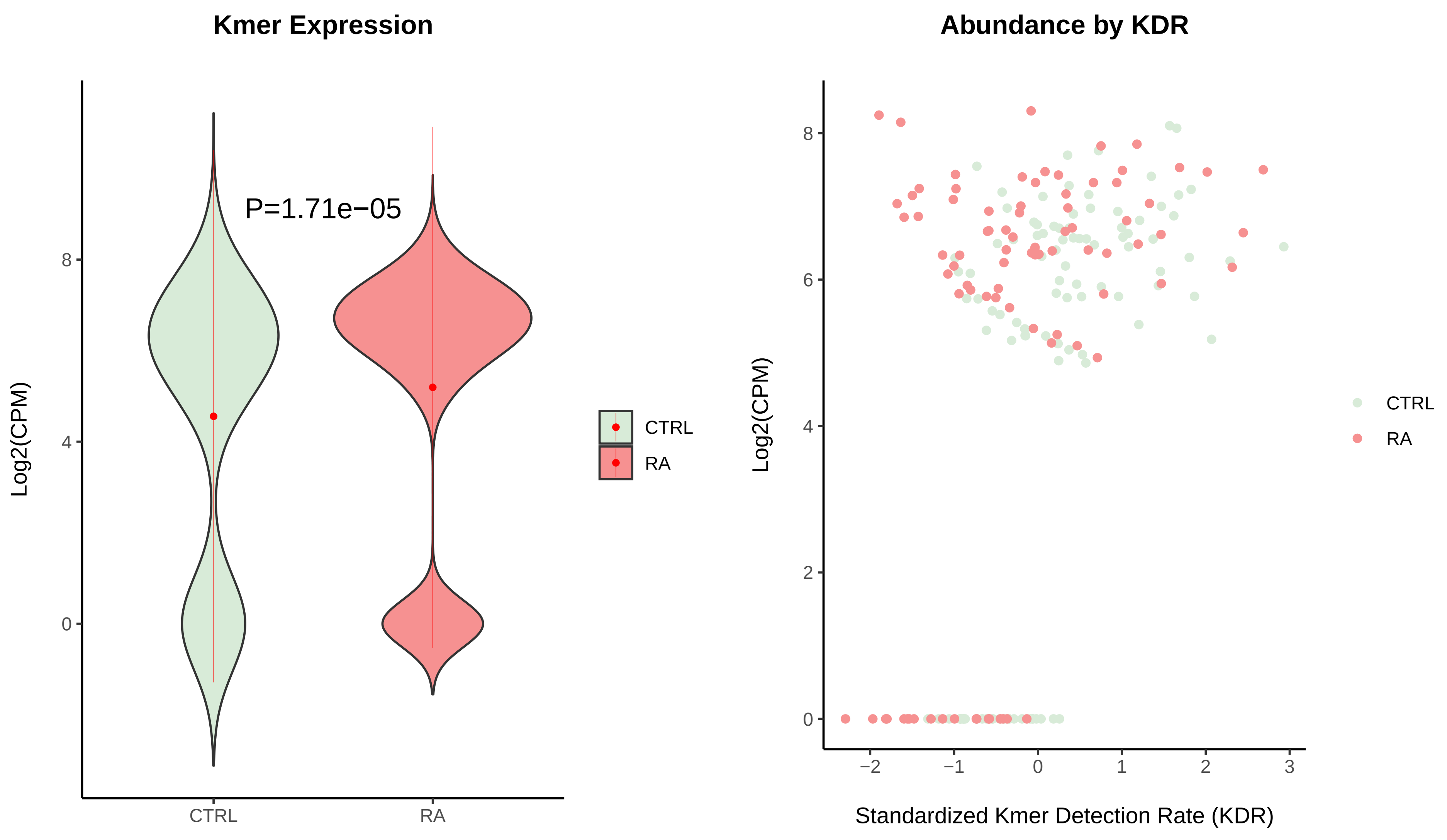

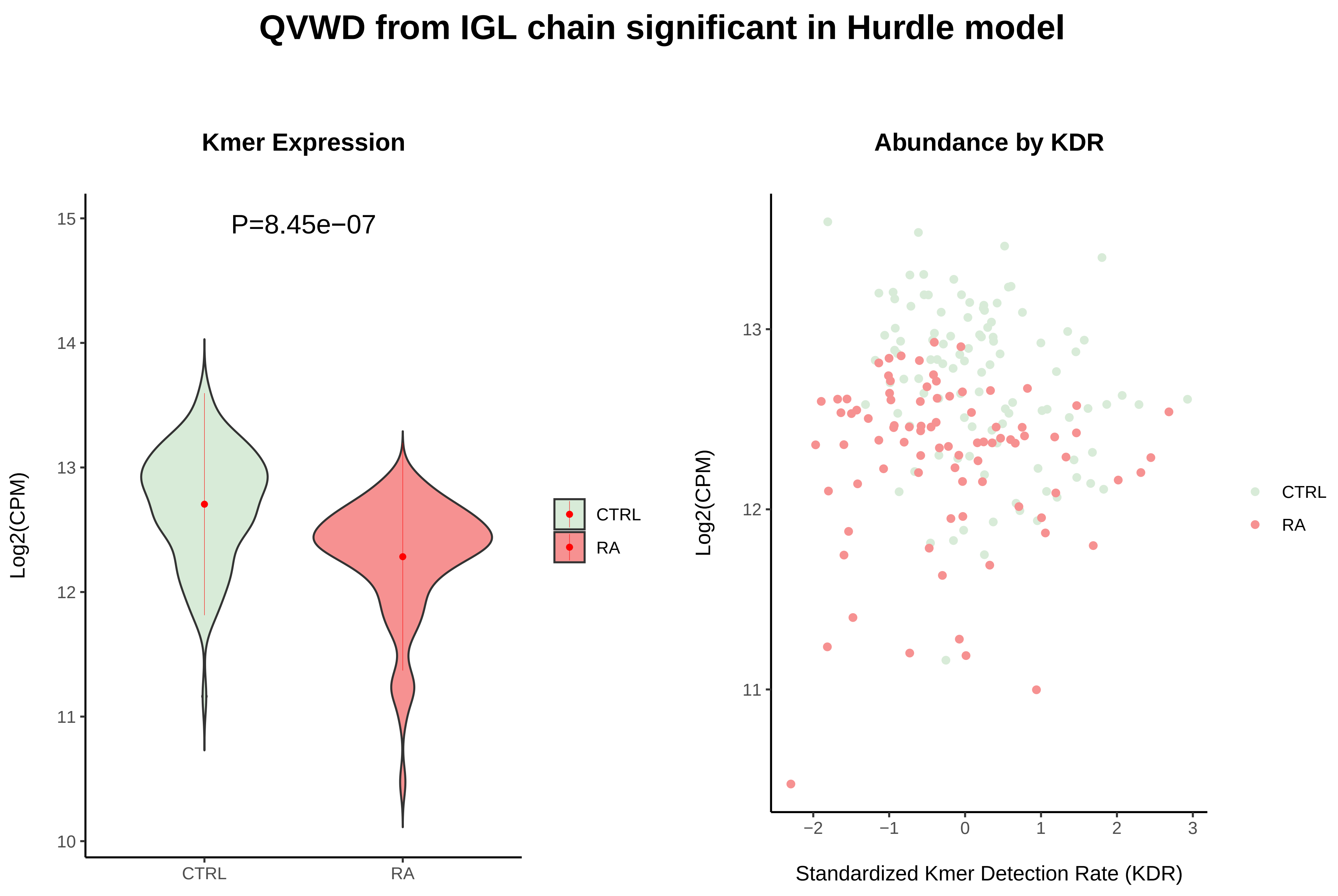
QVWD from IGL chain significant in Hurdle model
Kmer Expression
P=8.45e−07
Log2(CPM)
15
14
13
12
11
10
CTRL
RA
CTRL
RA
Abundance by KDR
Log2(CPM)
13
12
11
−2
−1
0
1
2
3
Standardized Kmer Detection Rate (KDR)
CTRL
RA

RDNT from IGL chain significant in Hurdle model

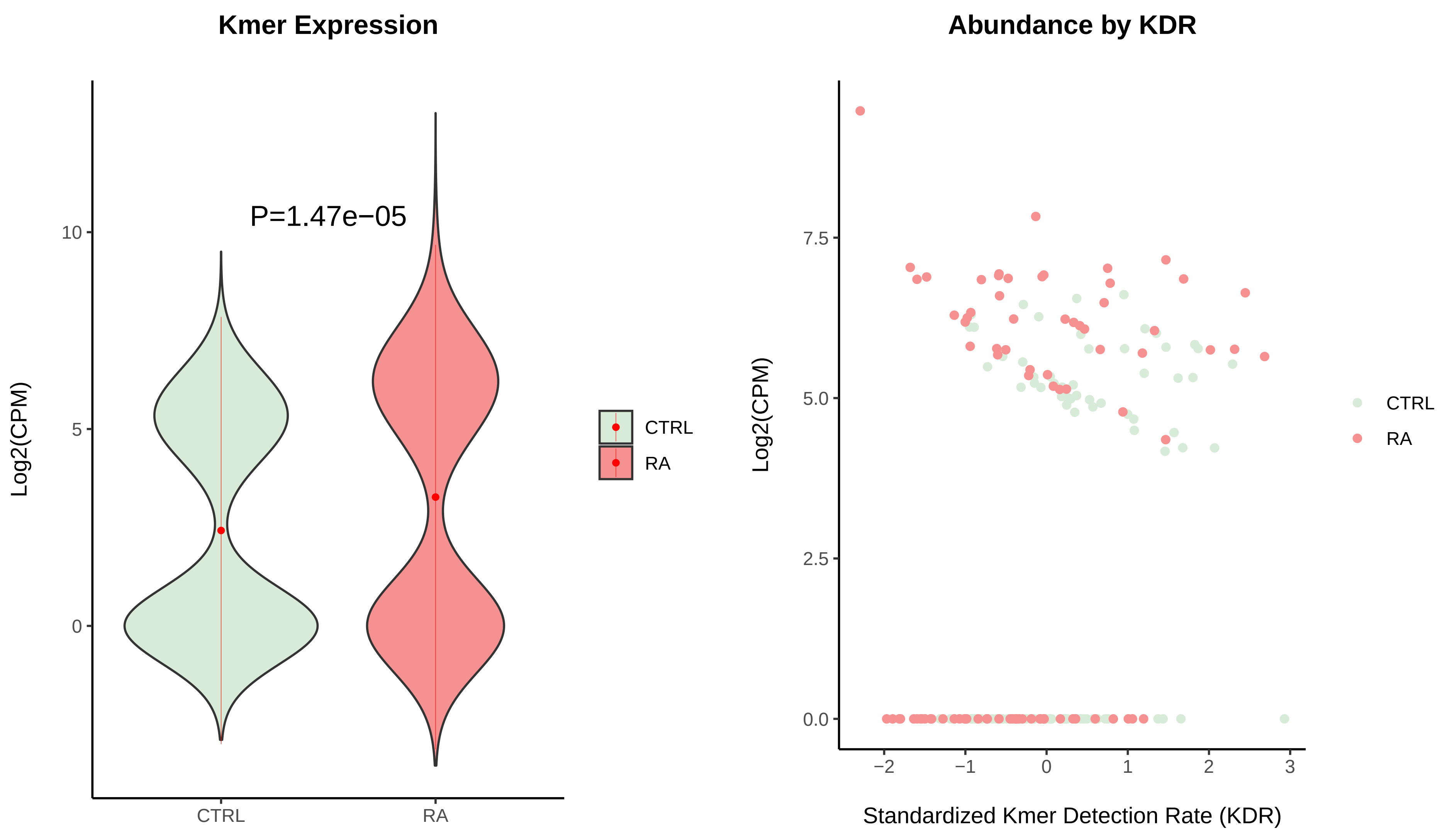

SDHP from IGL chain significant in Hurdle model

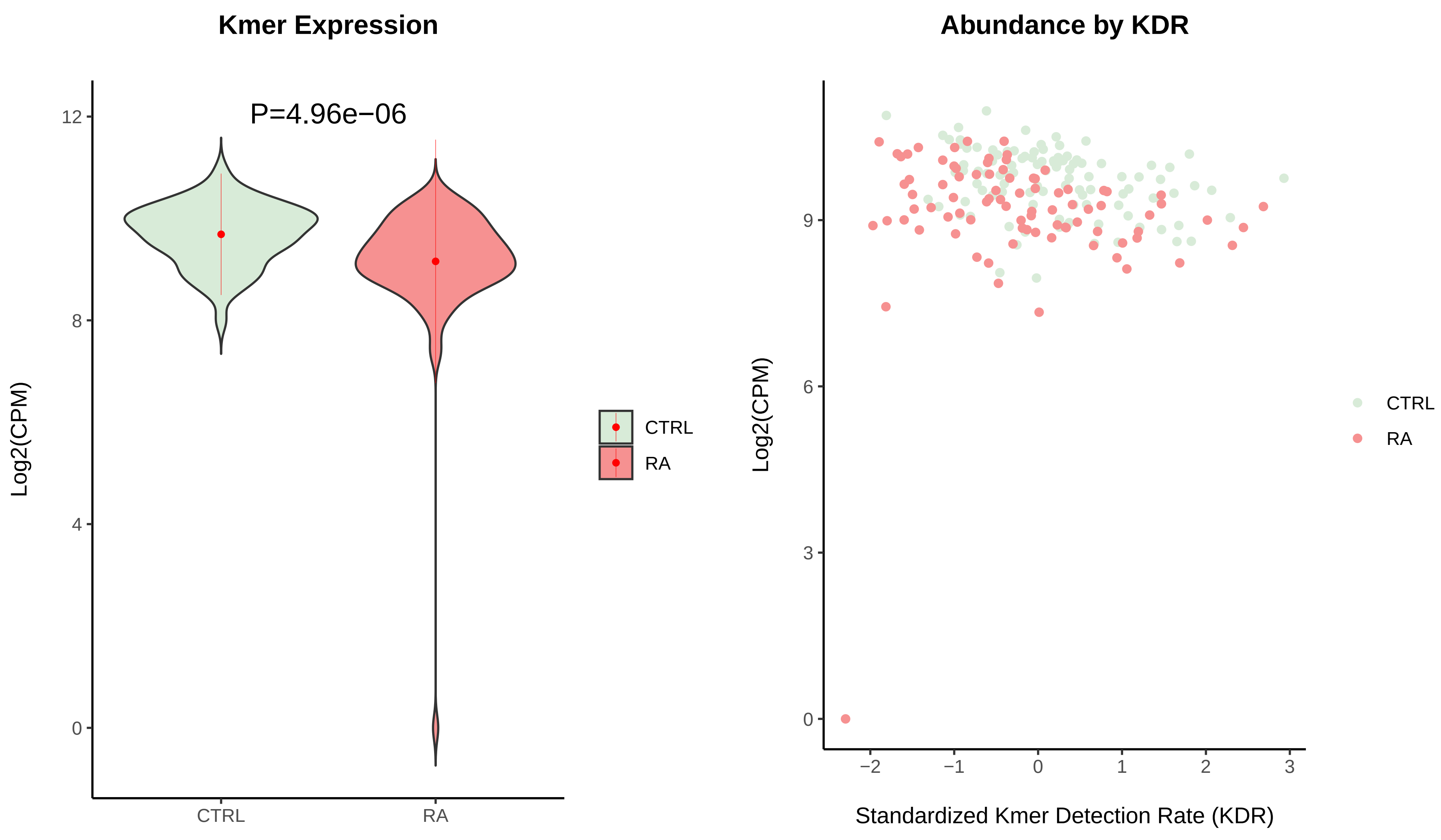

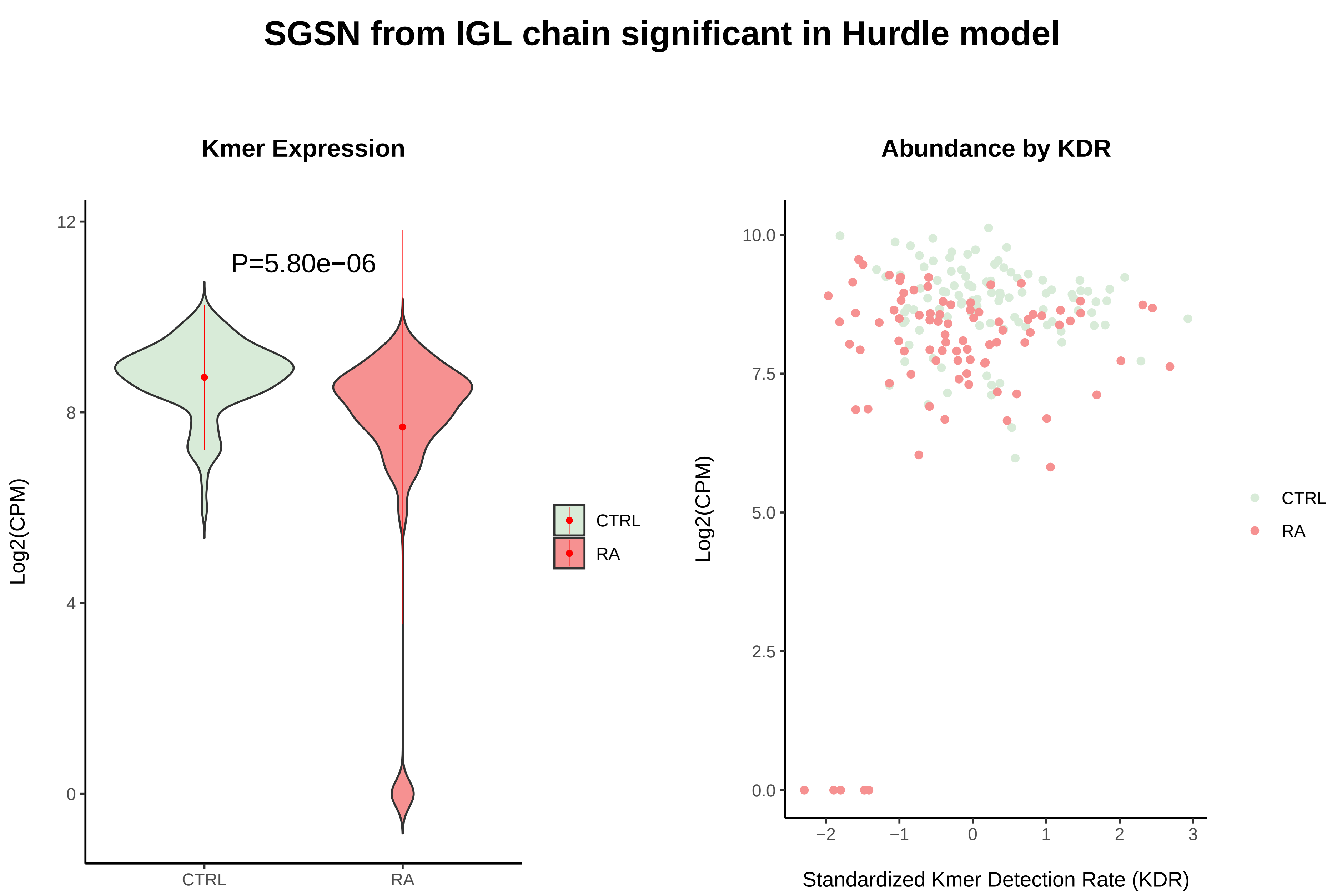
SGSN from IGL chain significant in Hurdle model
Kmer Expression
P=5.80e−06
Log2(CPM)
12
8
4
0
CTRL
RA
CTRL
RA
Abundance by KDR
Log2(CPM)
10.0
7.5
5.0
2.5
0.0
−2
−1
0
1
2
3
Standardized Kmer Detection Rate (KDR)
CTRL
RA

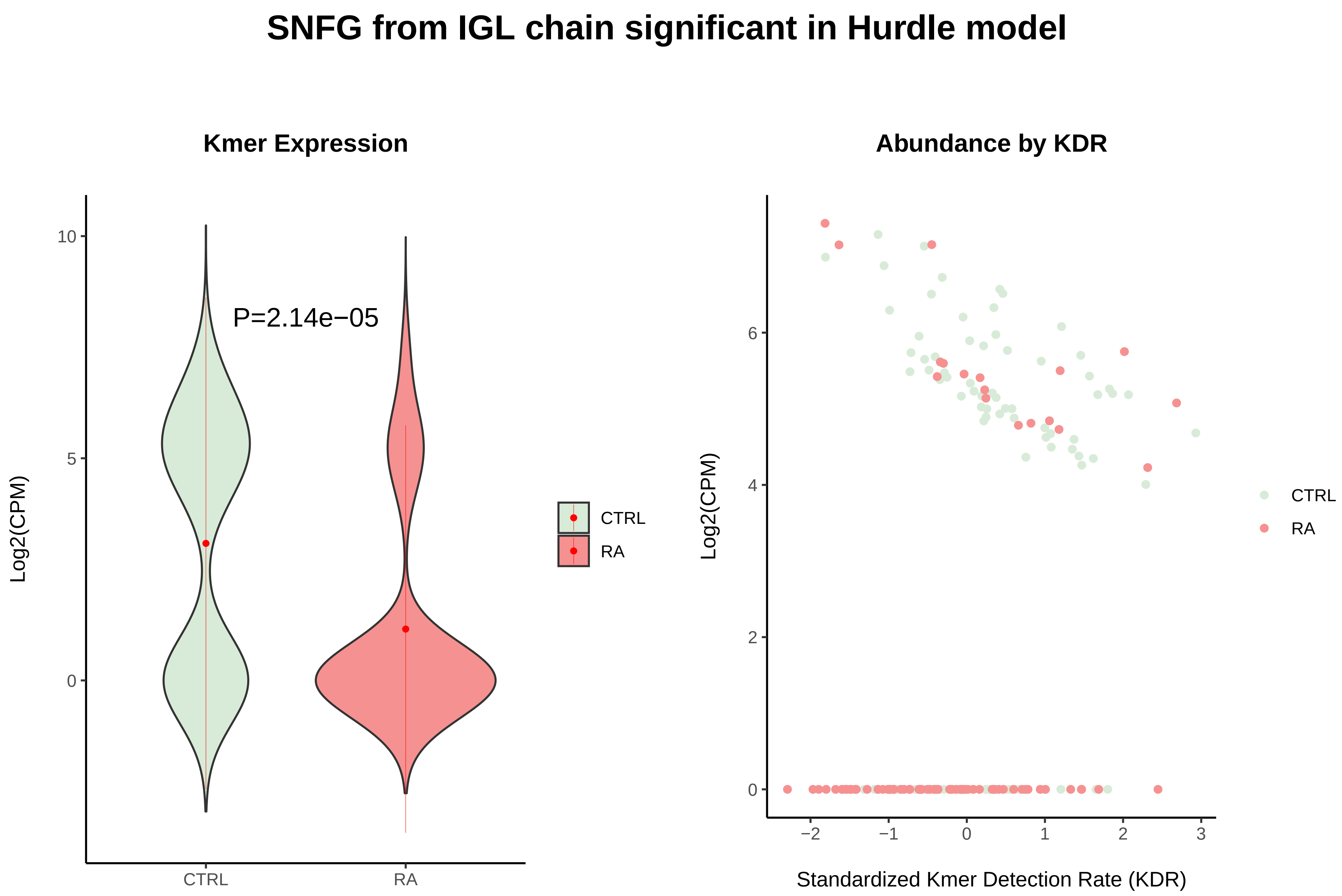

SNFG from IGL chain significant in Hurdle model
Kmer Expression
P=2.14e−05
Log2(CPM)
10
5
0
CTRL
RA
CTRL
RA
Abundance by KDR
Log2(CPM)
6
4
2
0
−2
−1
0
1
2
3
Standardized Kmer Detection Rate (KDR)
CTRL
RA

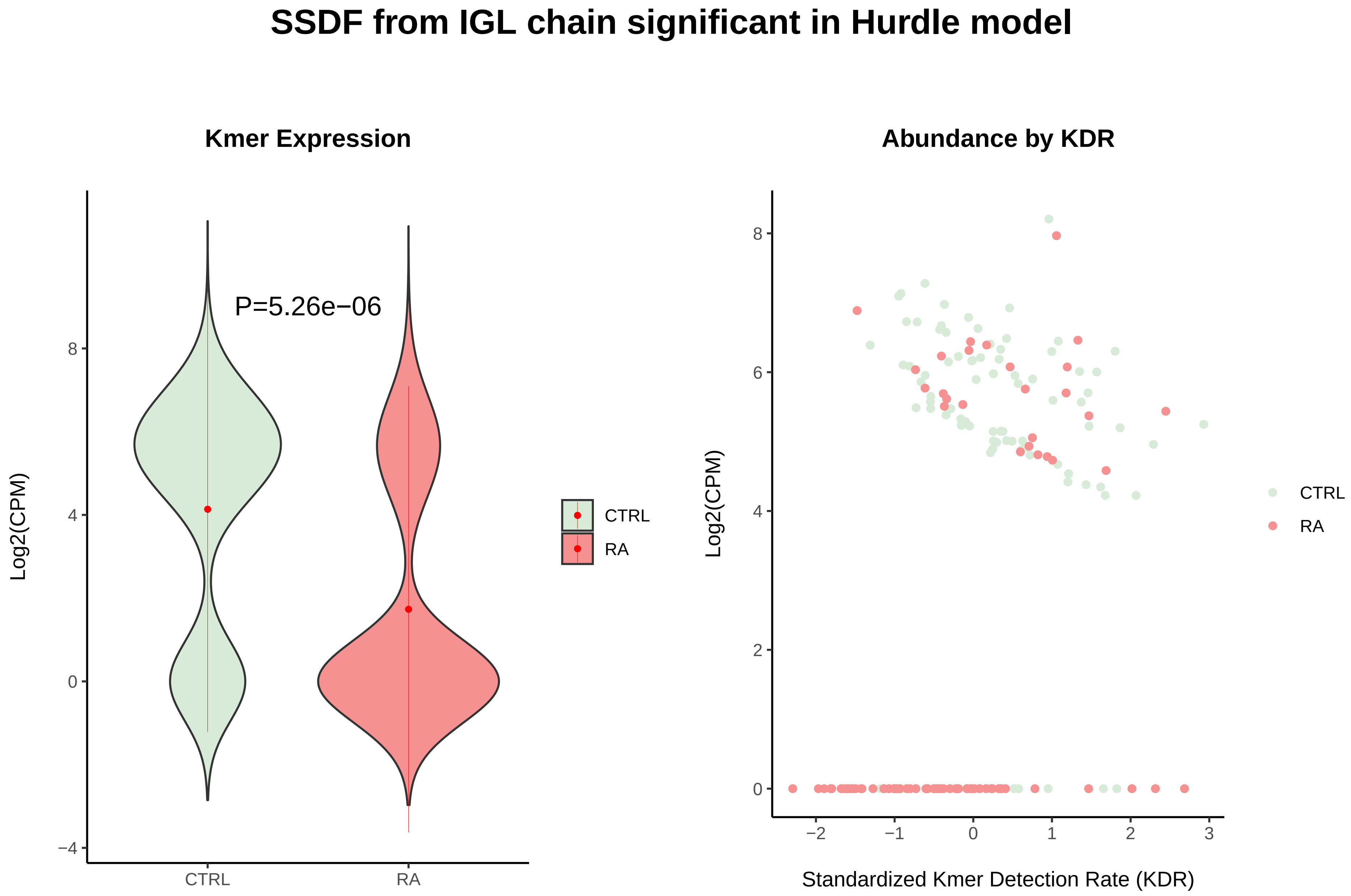
SSDF from IGL chain significant in Hurdle model
Kmer Expression
P=5.26e−06
Log2(CPM)
8
4
0
−4
CTRL
RA
CTRL
RA
Abundance by KDR
Log2(CPM)
8
6
4
2
0
−2
−1
0
1
2
3
Standardized Kmer Detection Rate (KDR)
CTRL
RA

SSDH from IGL chain significant in Hurdle model

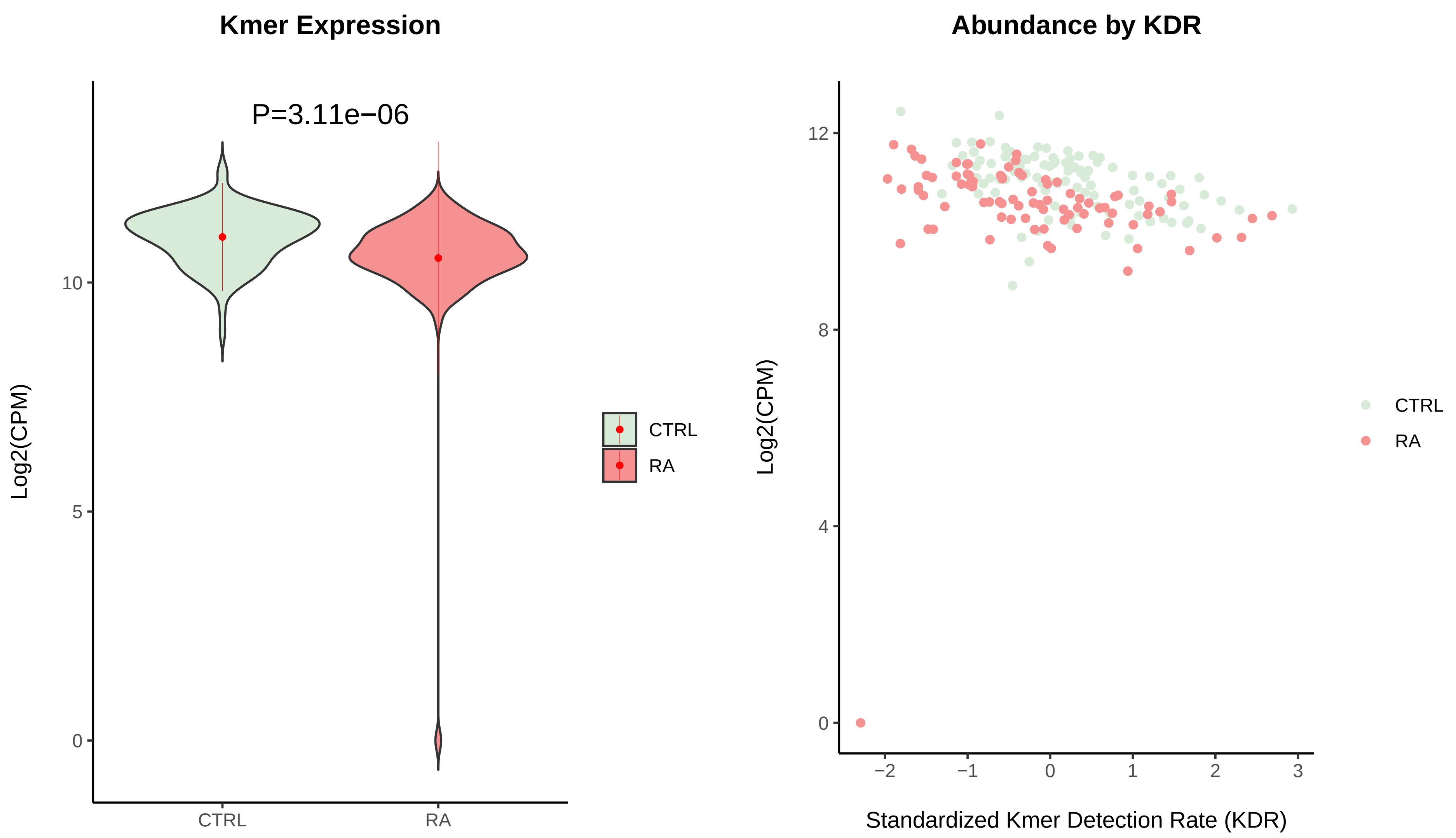

SSDQ from IGL chain significant in Hurdle model

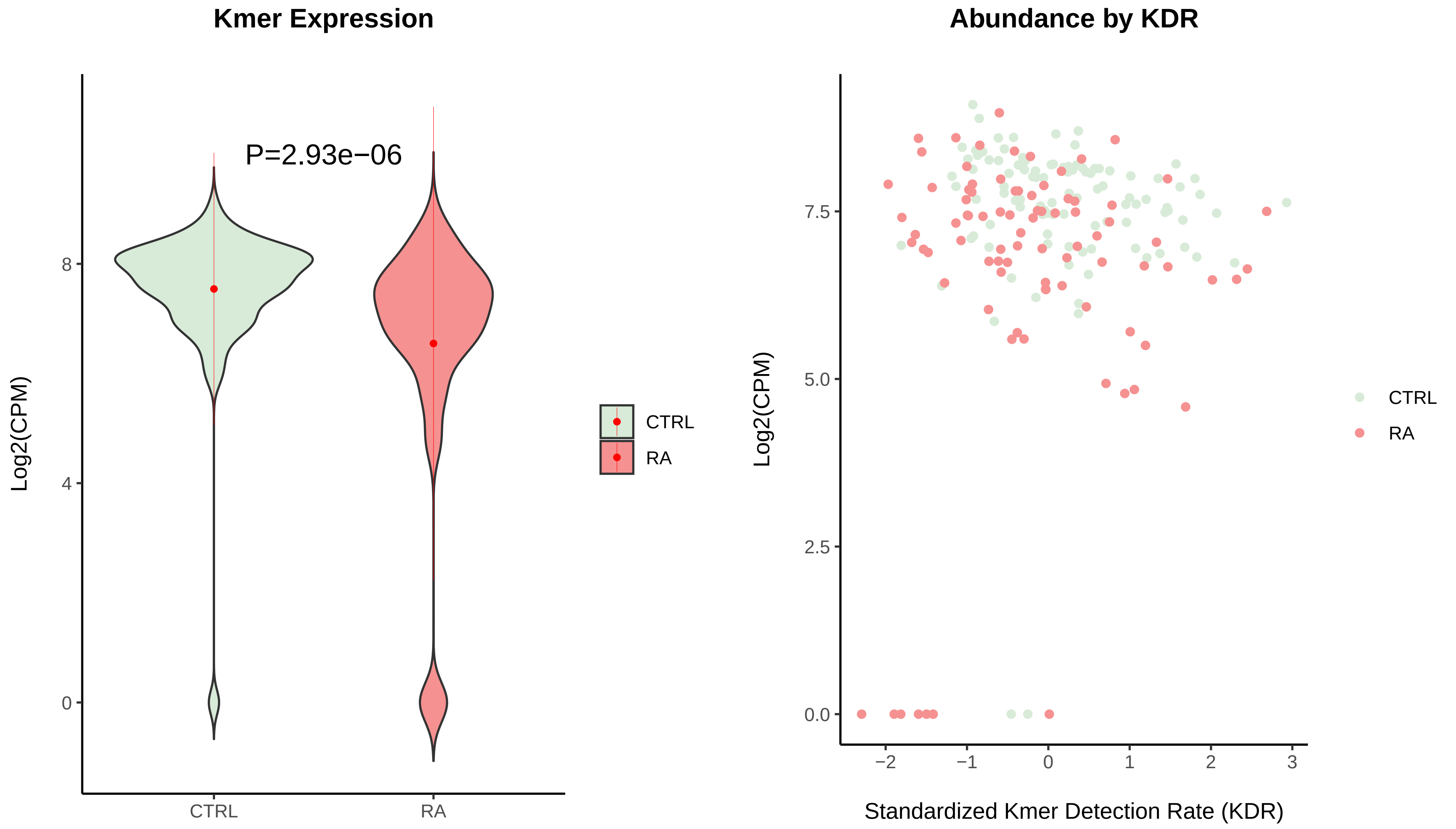

SSSD from IGL chain significant in Hurdle model

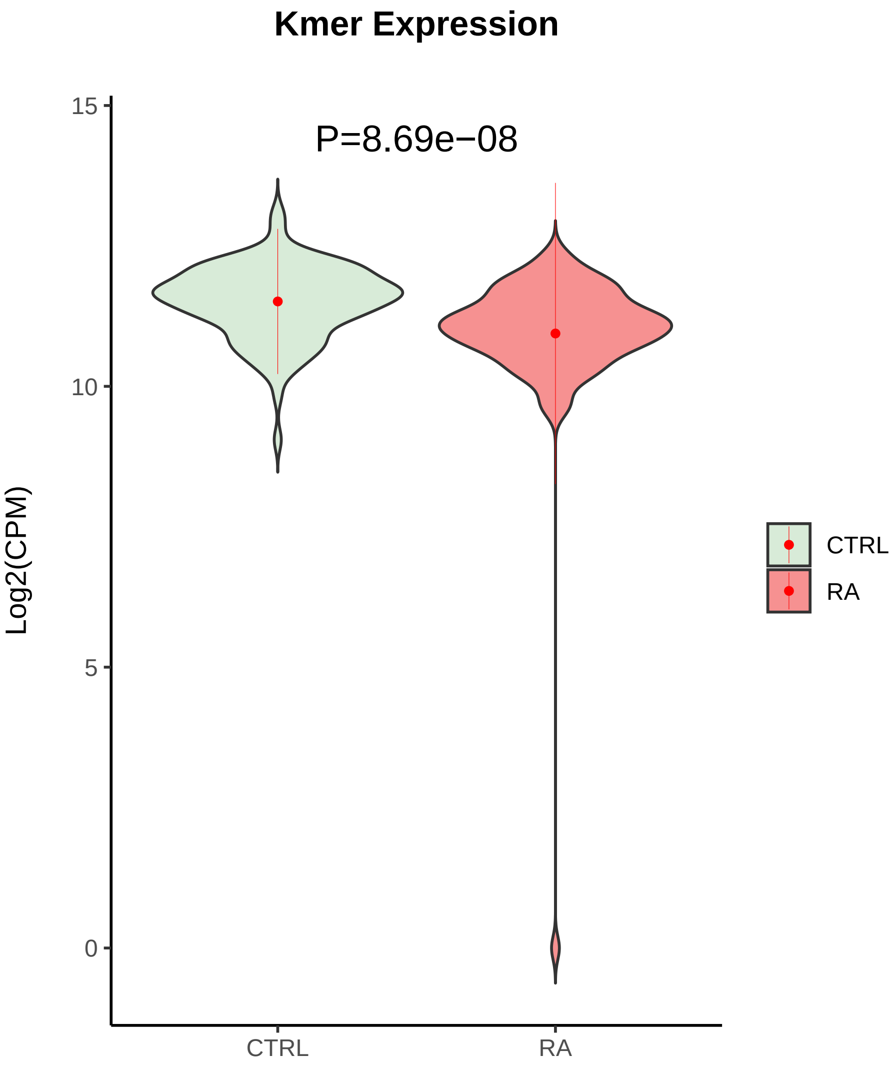

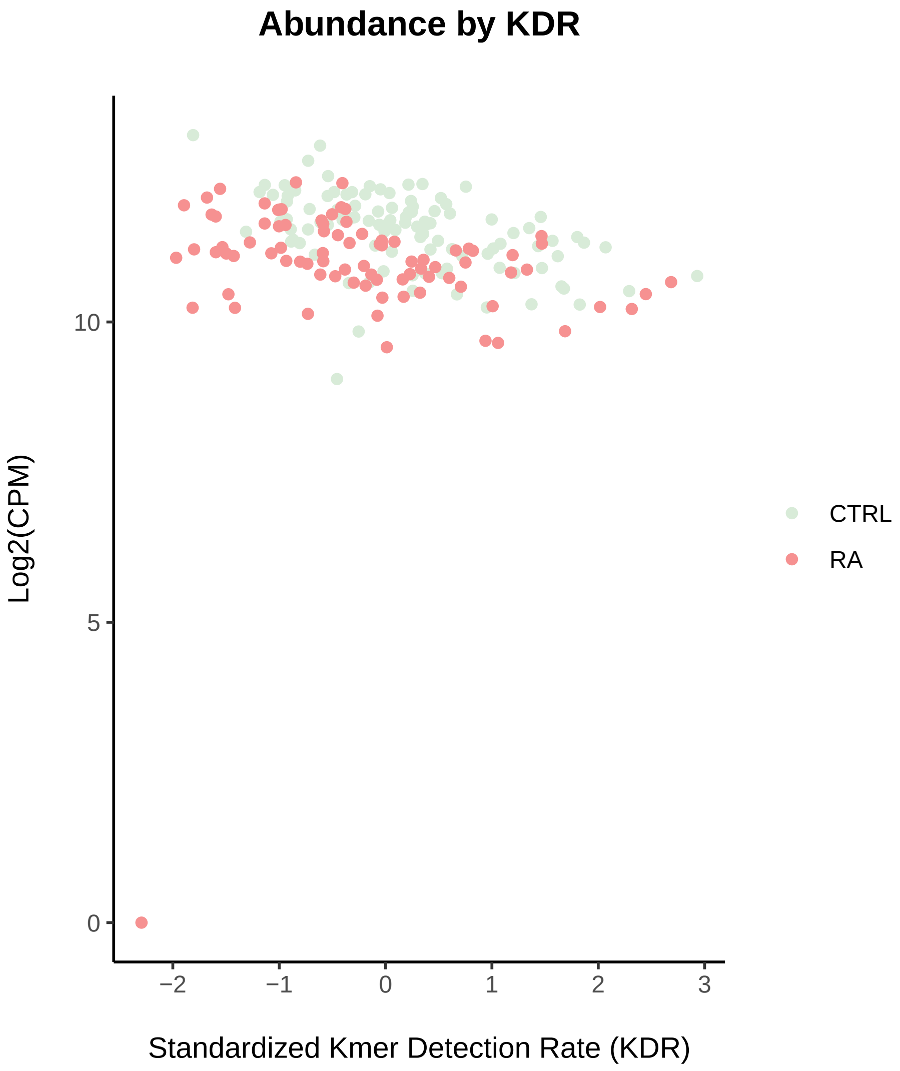

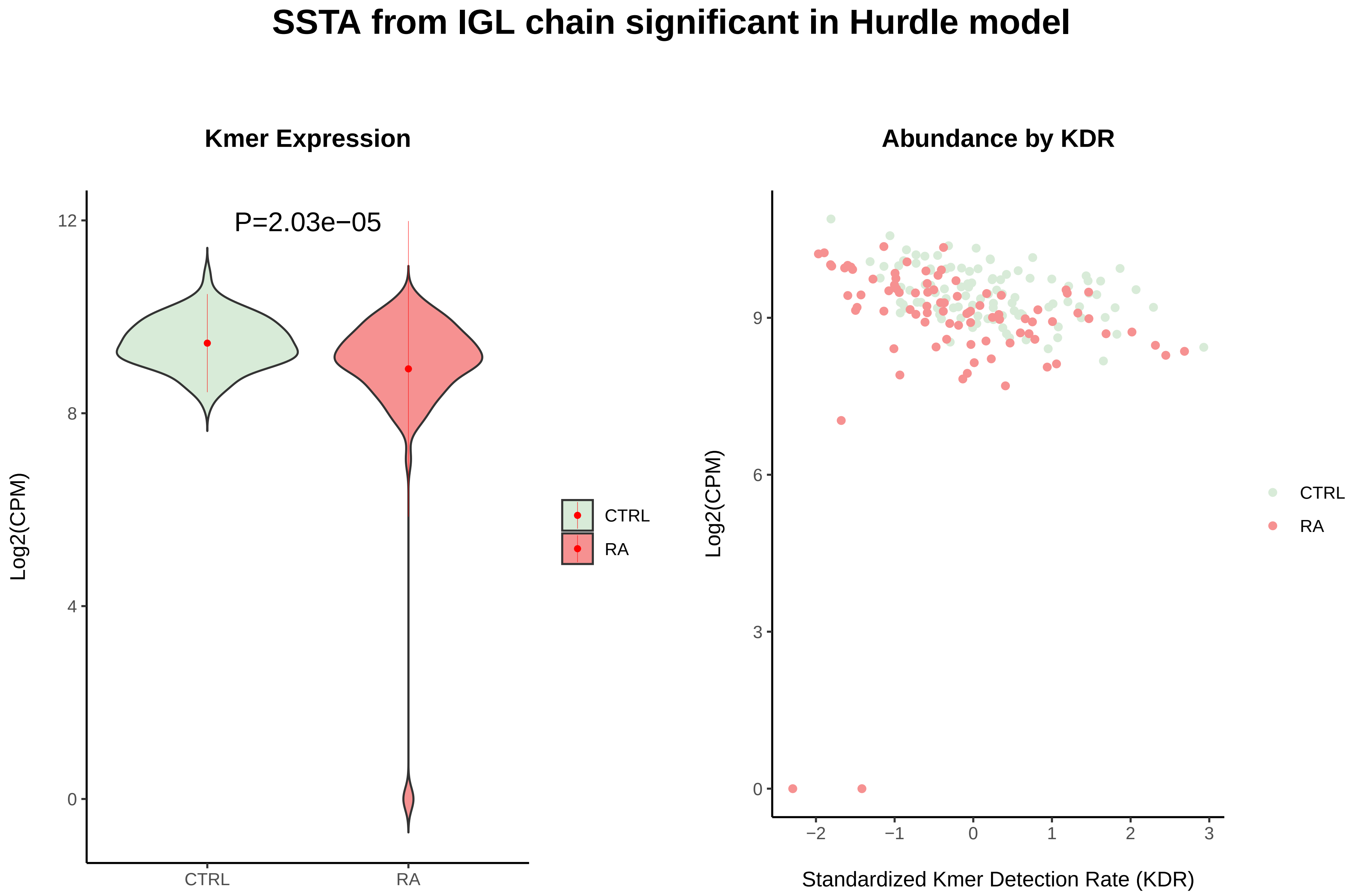
SSTA from IGL chain significant in Hurdle model
Kmer Expression
P=2.03e−05
Log2(CPM)
12
8
4
0
CTRL
RA
CTRL
RA
Abundance by KDR
Log2(CPM)
9
6
3
0
−2
−1
0
1
2
3
Standardized Kmer Detection Rate (KDR)
CTRL
RA

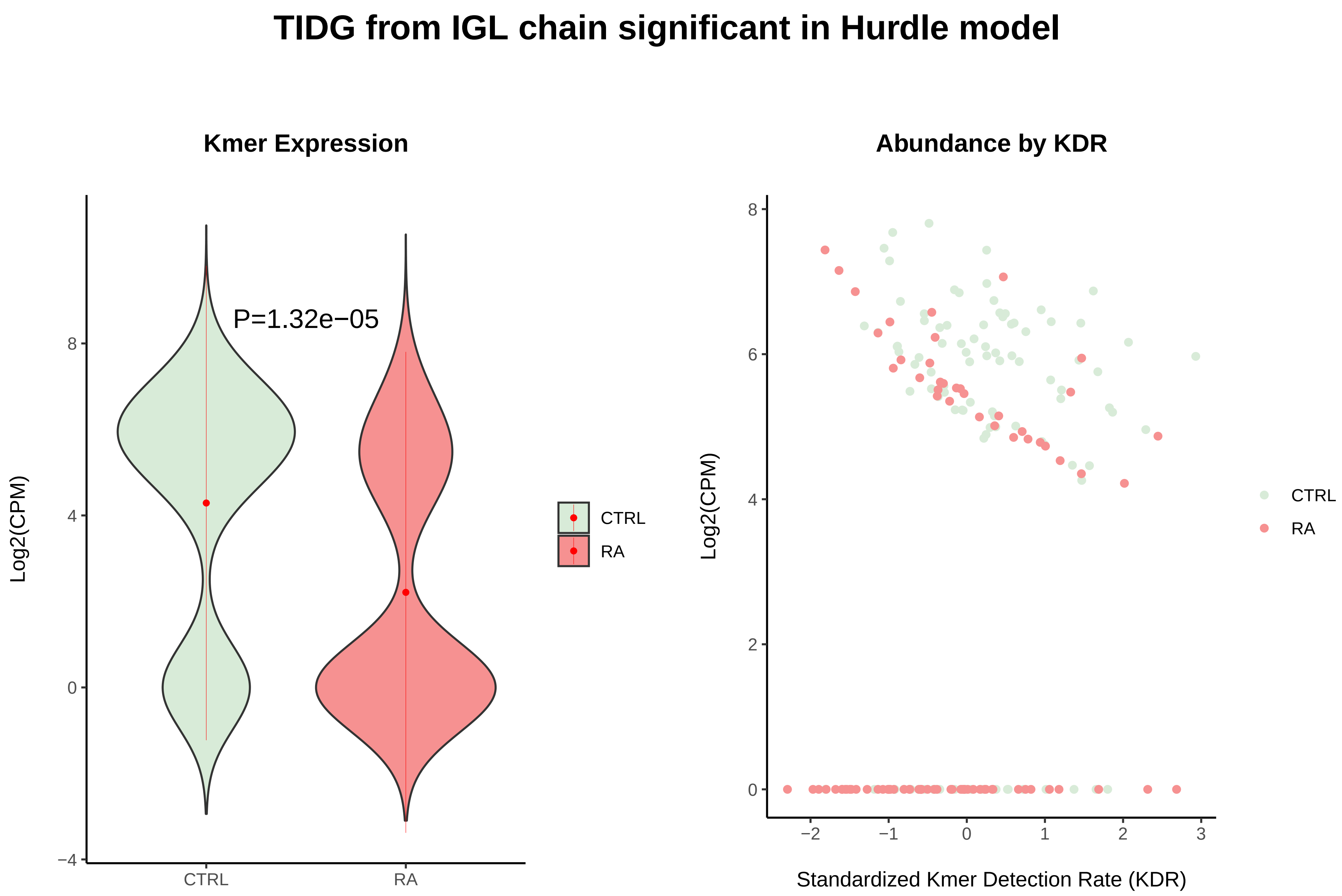
TIDG from IGL chain significant in Hurdle model
Kmer Expression
P=1.32e−05
Log2(CPM)
8
4
0
−4
CTRL
RA
Abundance by KDR
Log2(CPM)
8
6
4
2
0
−2
−1
0
1
2
3
Standardized Kmer Detection Rate (KDR)

TTLS from IGL chain significant in Hurdle model

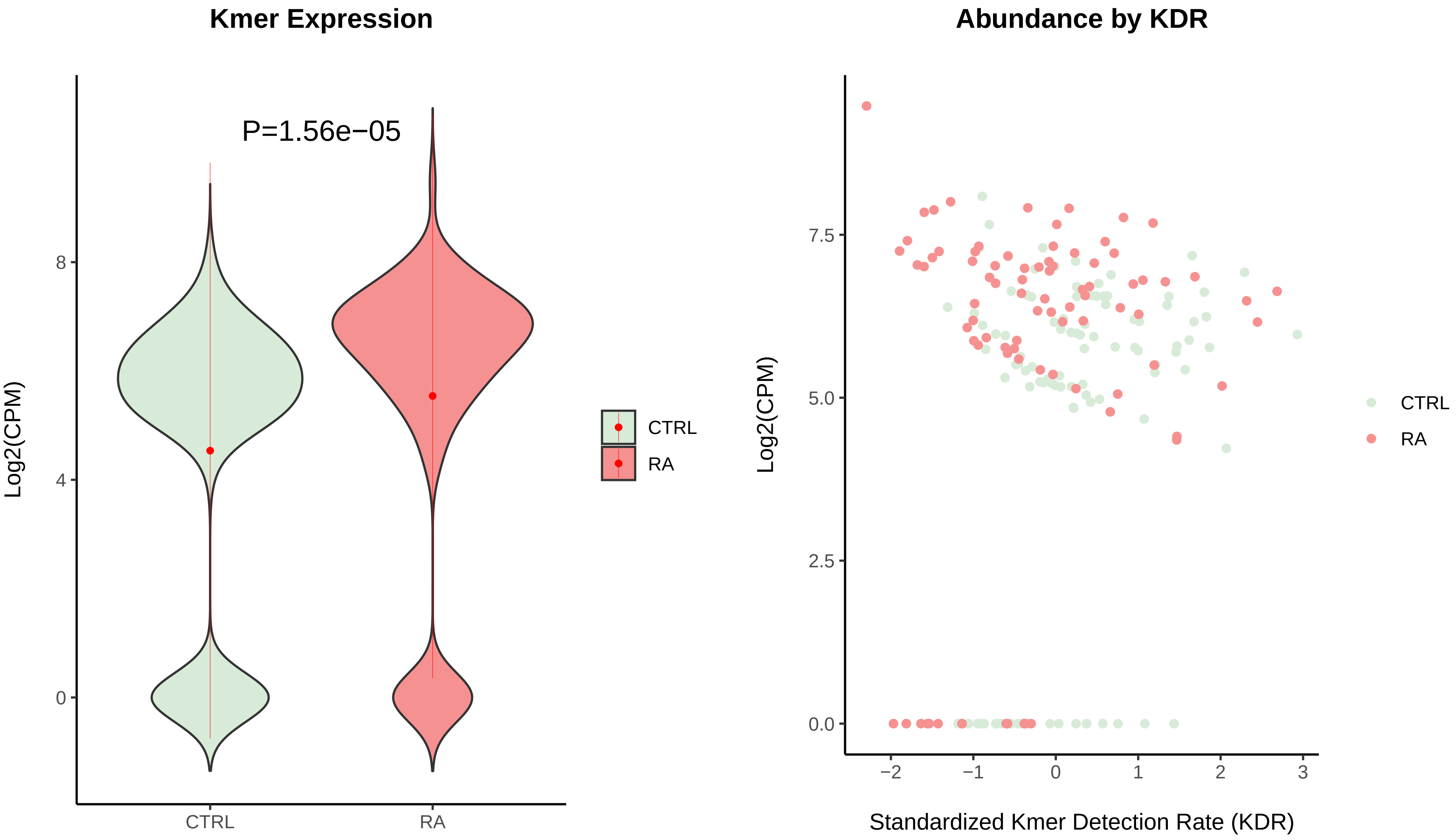

VWDS from IGL chain significant in Hurdle model

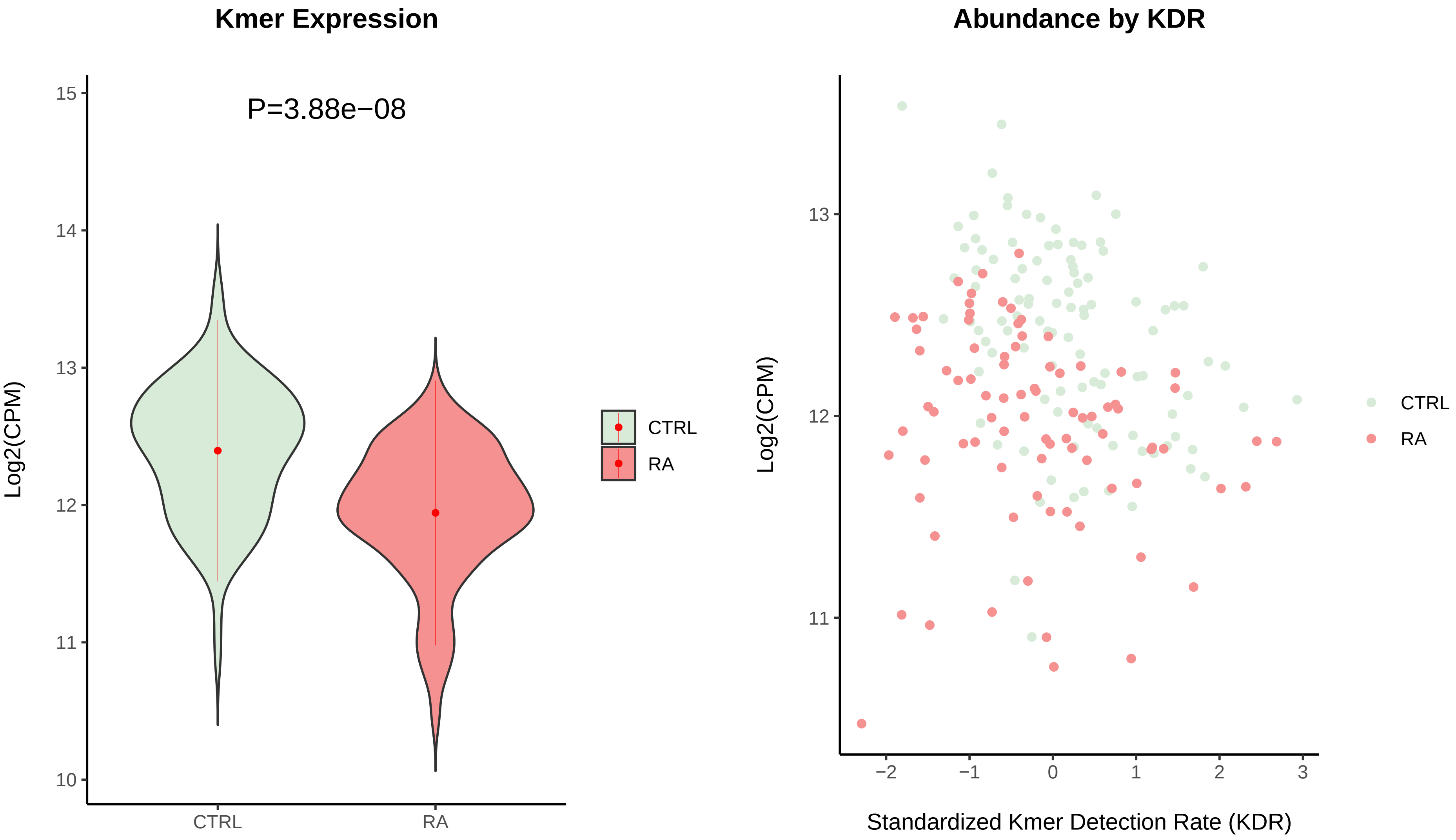

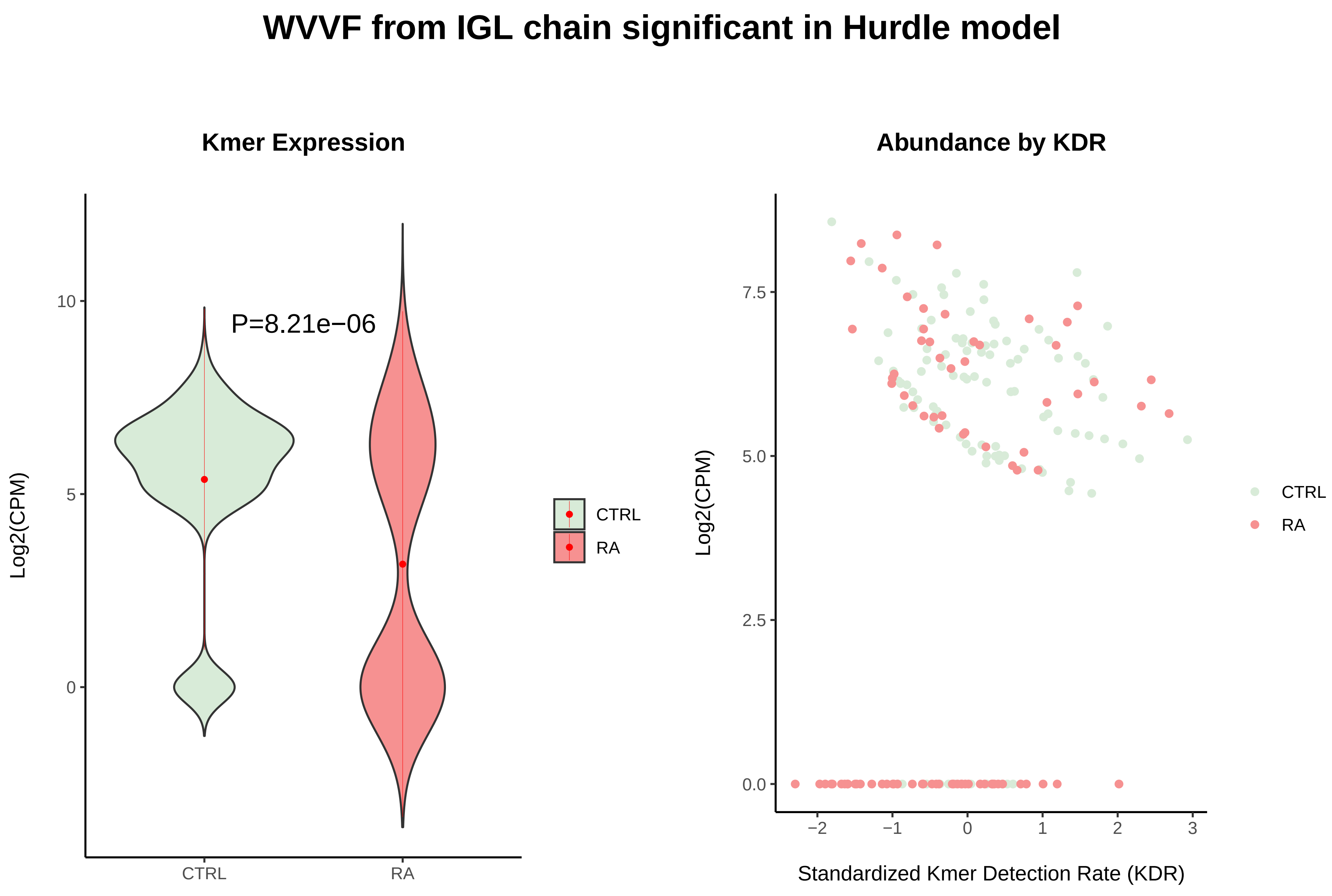
WVVF from IGL chain significant in Hurdle model
Kmer Expression
P=8.21e−06
Log2(CPM)
10
5
0
CTRL
RA
CTRL
RA
Abundance by KDR
Log2(CPM)
7.5
5.0
2.5
0.0
−2
−1
0
1
2
3
Standardized Kmer Detection Rate (KDR)
CTRL
RA

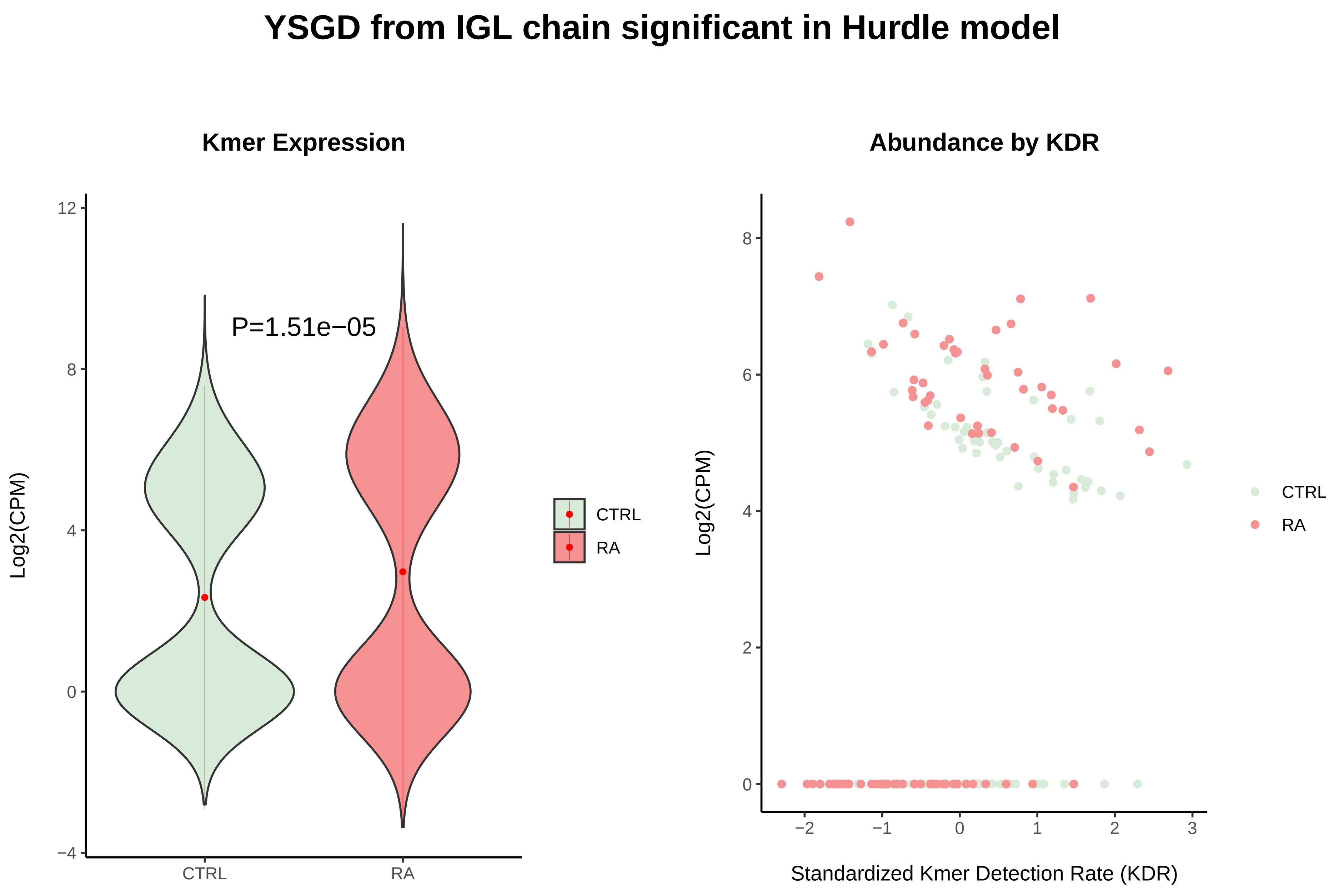
YSGD from IGL chain significant in Hurdle model
Kmer Expression
Abundance by KDR
P=1.51e−05
Log2(CPM)
12
8
4
0
−4
CTRL
RA
CTRL
RA
Log2(CPM)
8
6
4
2
0
−2
−1
0
1
2
3
Standardized Kmer Detection Rate (KDR)
CTRL
RA

ADHG from IGL chain significant in Cont model

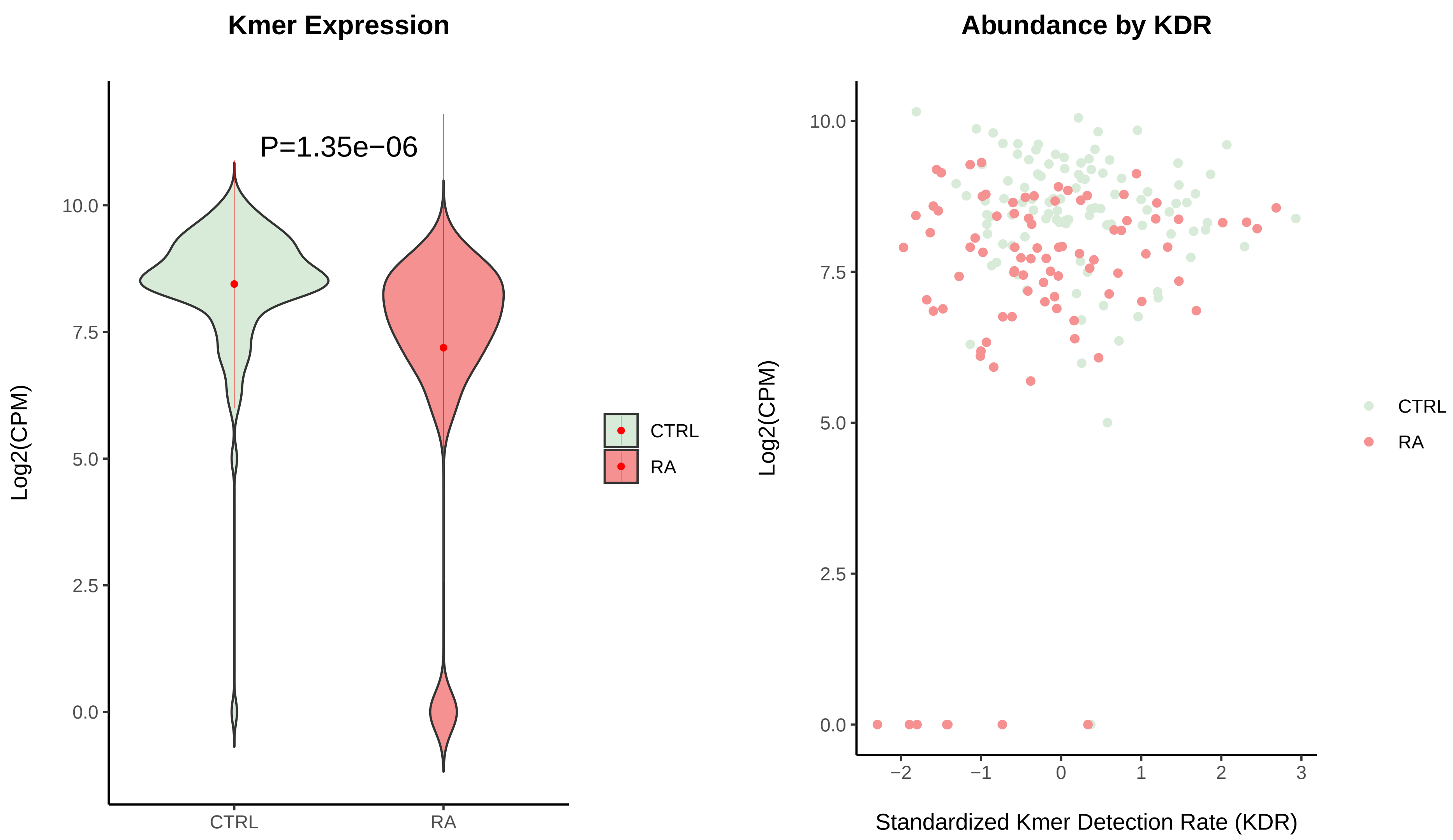

CFLS from IGL chain significant in Cont model

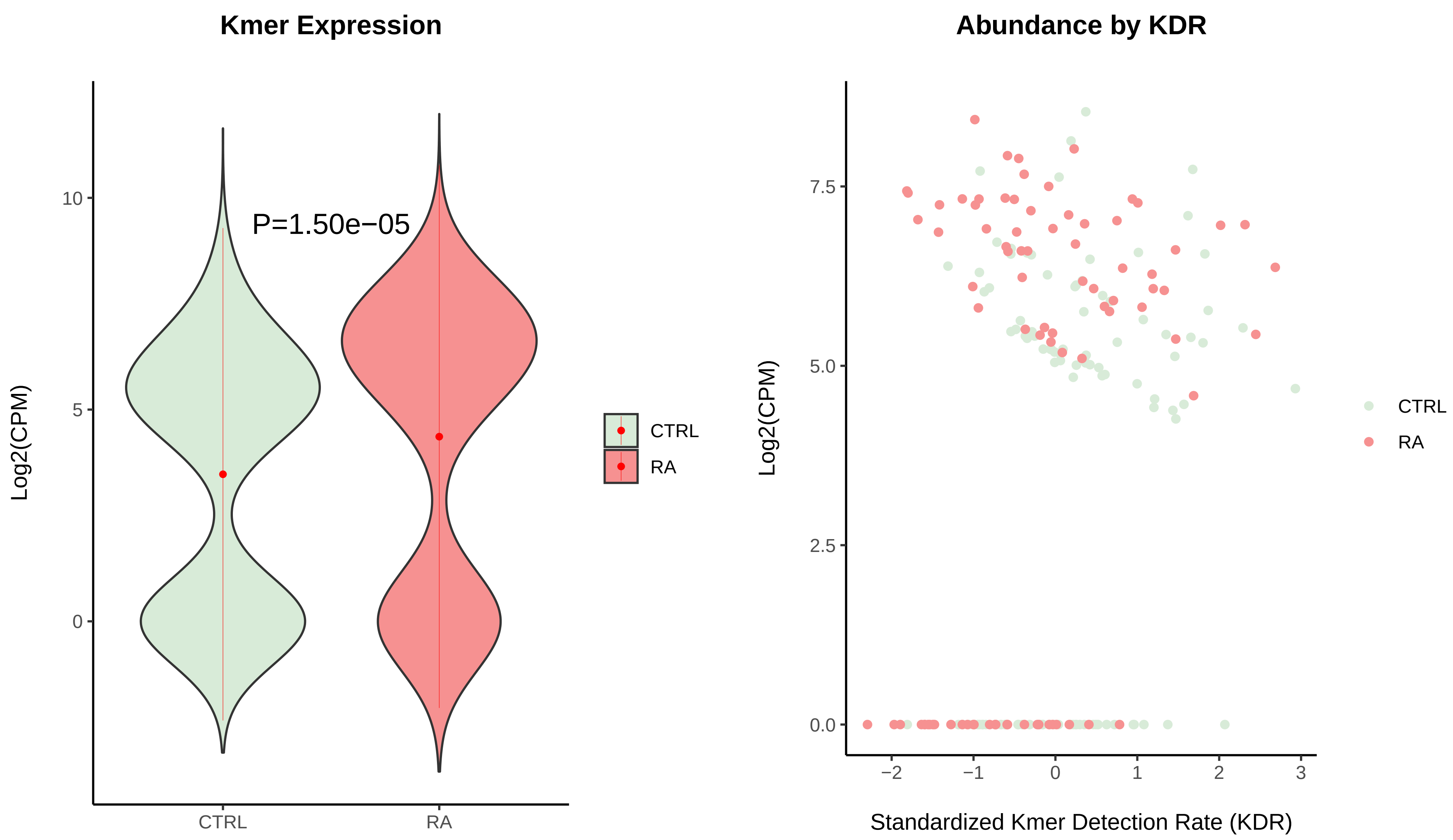

CGAD from IGL chain significant in Cont model

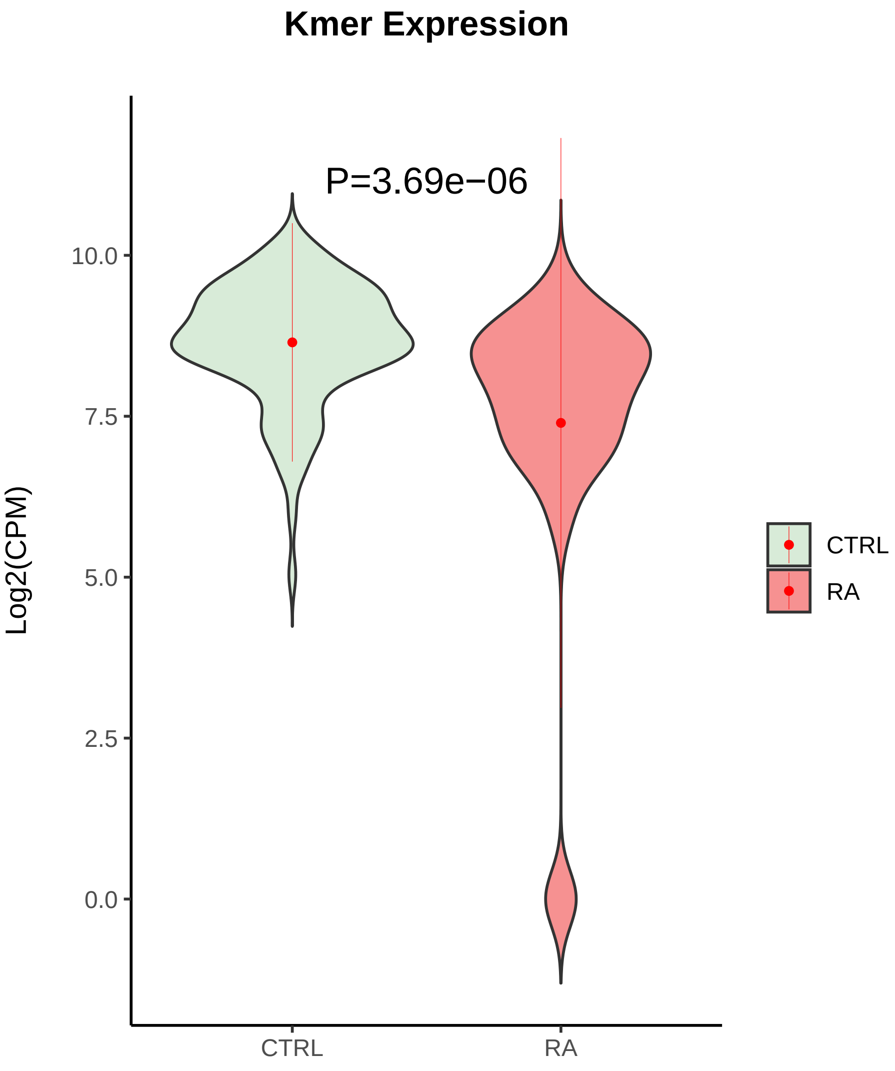

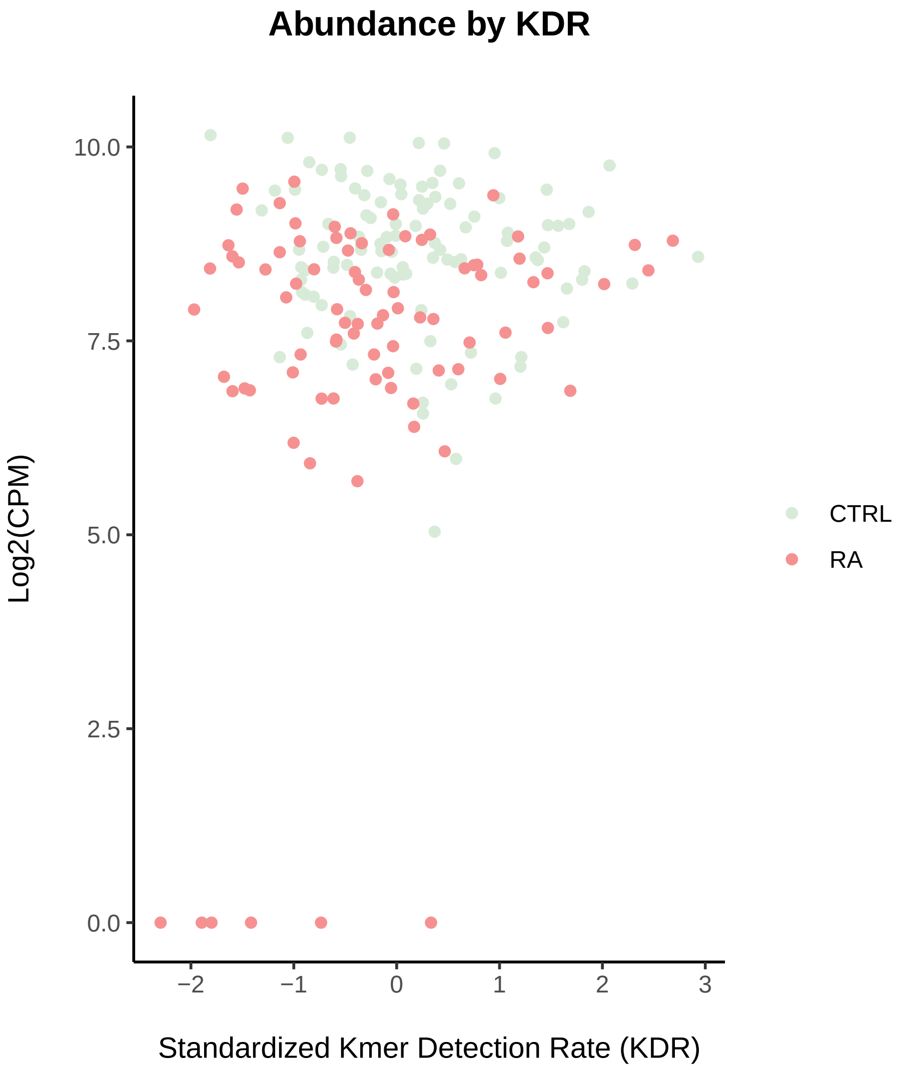

CQVW from IGL chain significant in Cont model

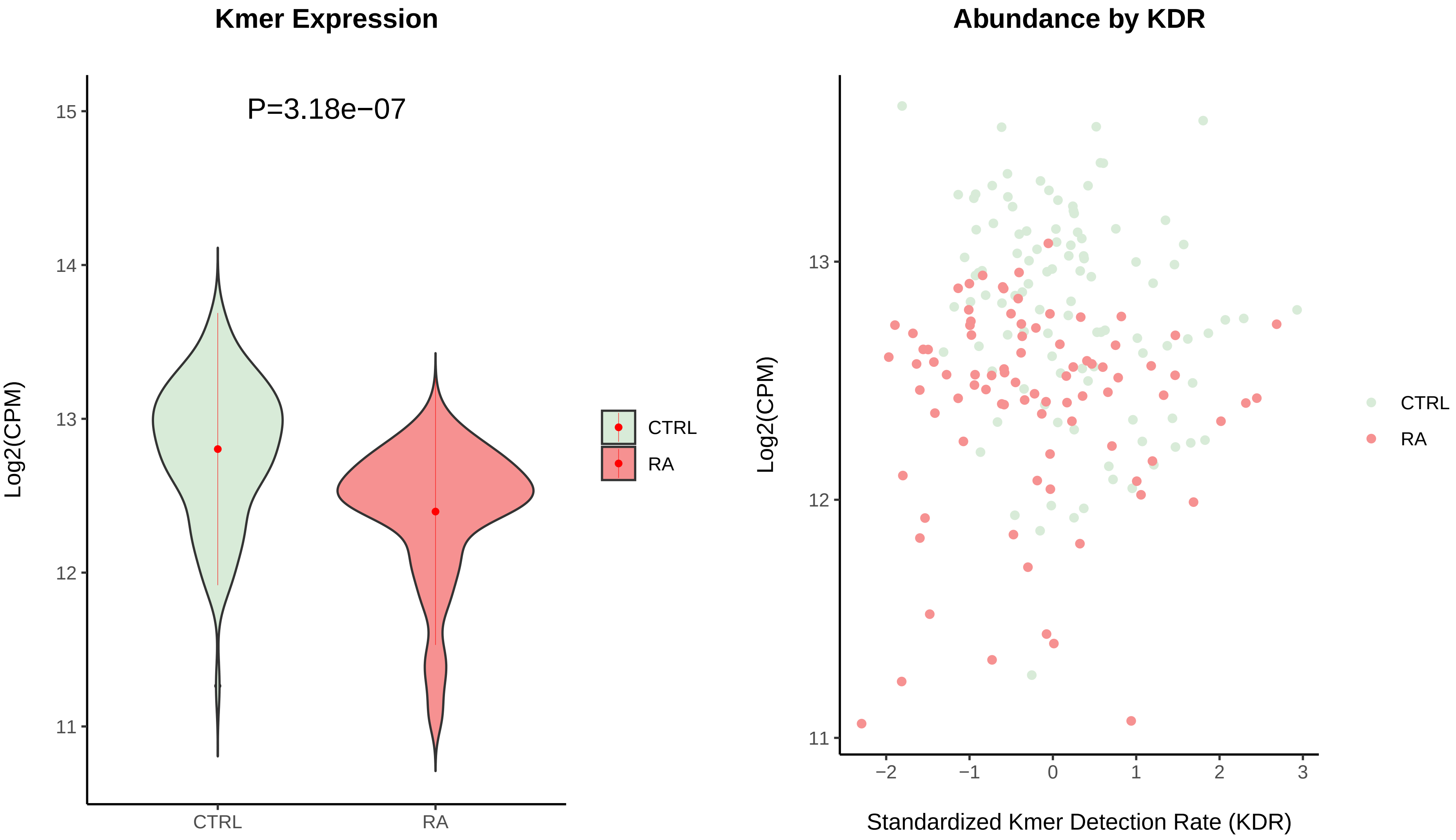

DHGS from IGL chain significant in Cont model

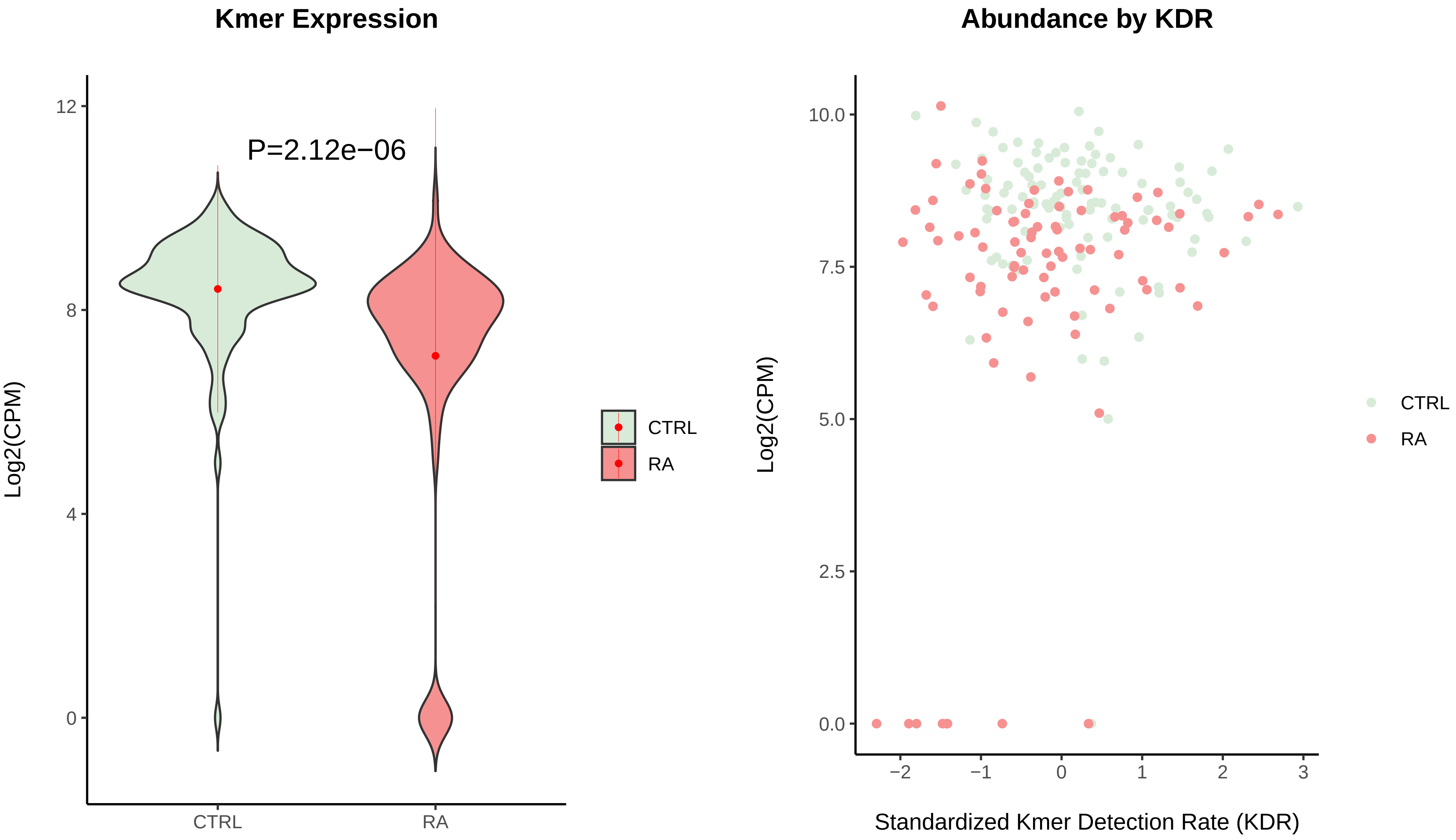

DHPG from IGL chain significant in Cont model

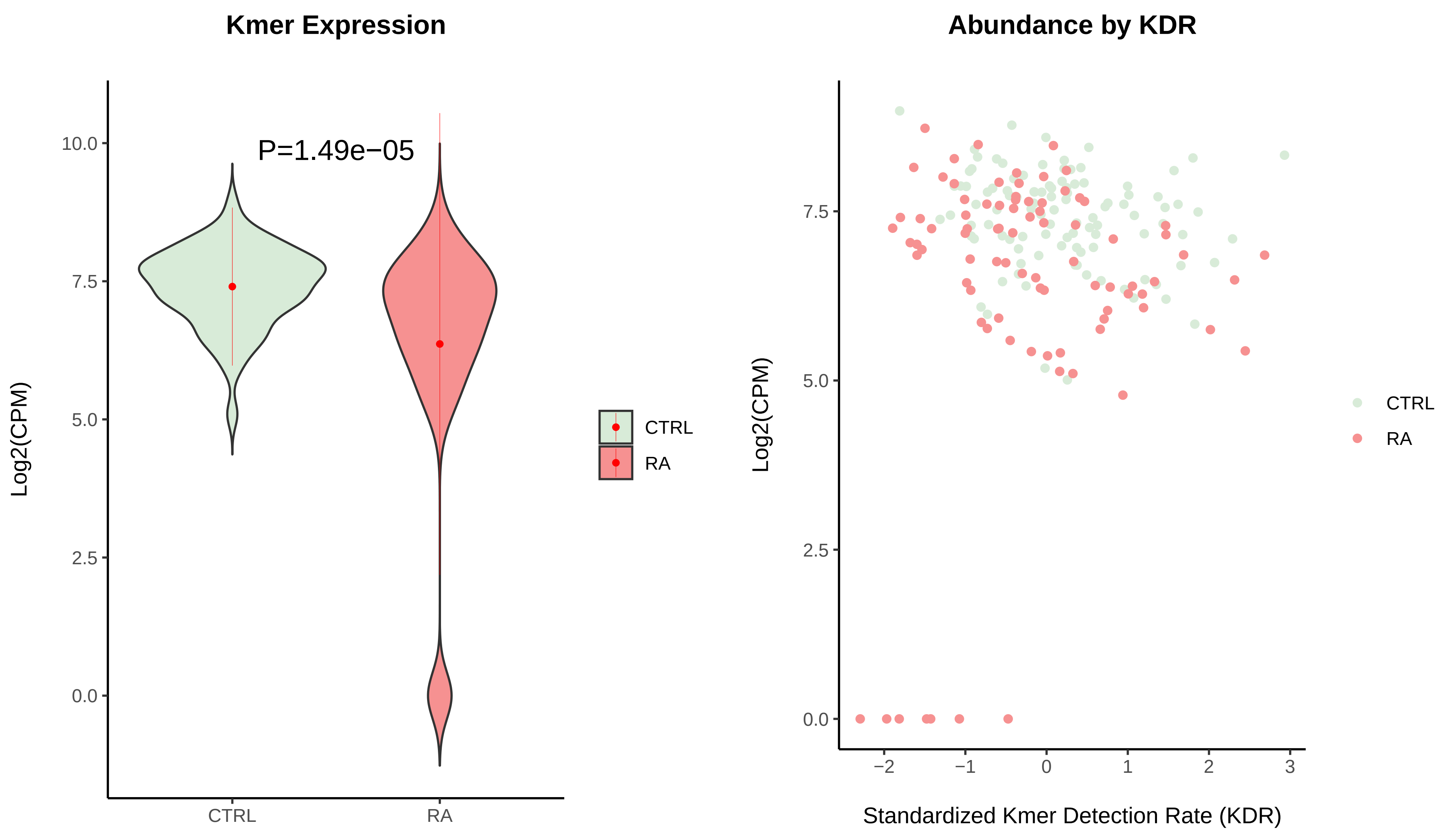

DSSS from IGL chain significant in Cont model

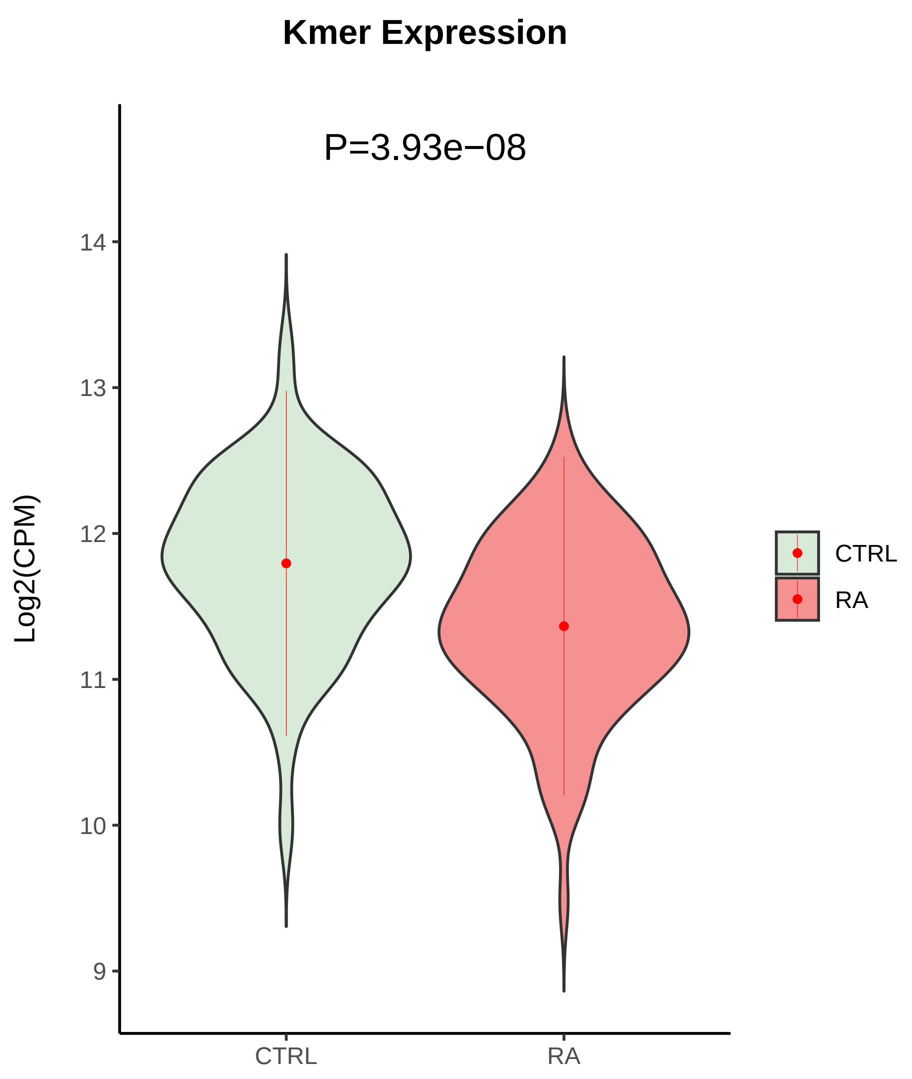

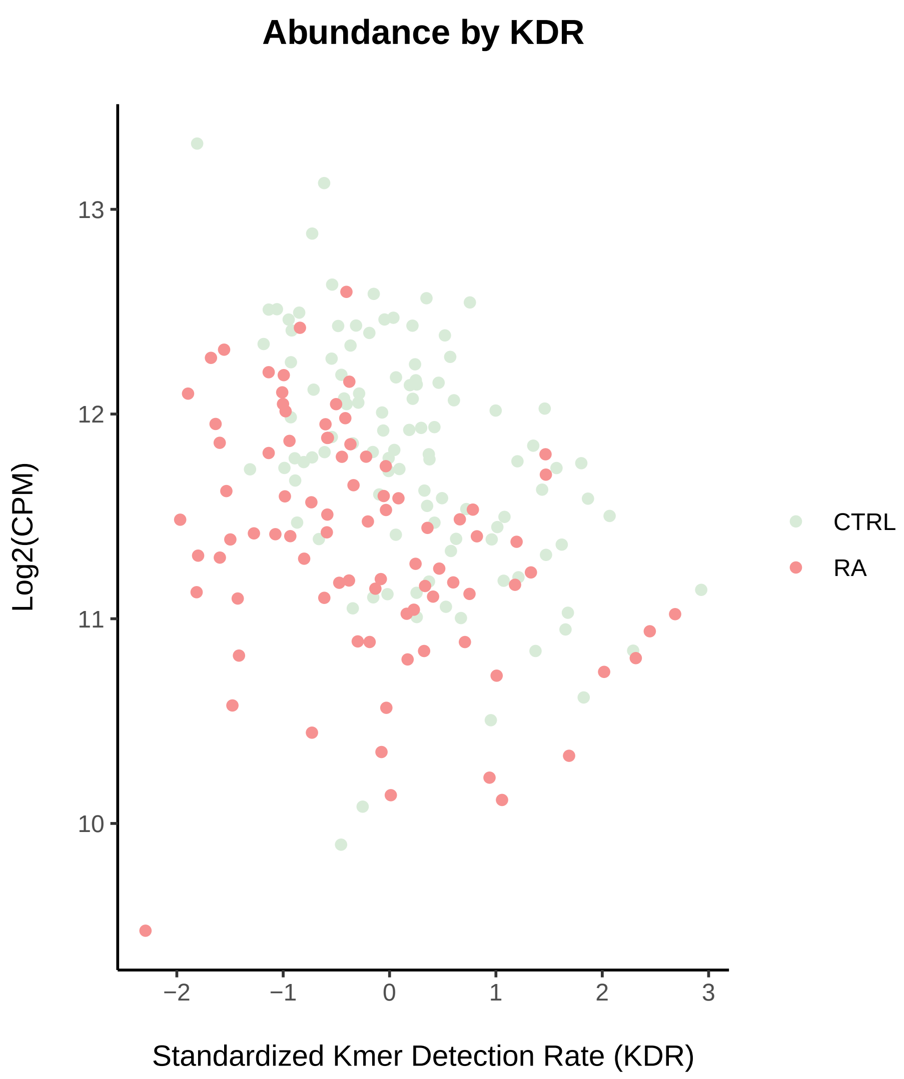

DSST from IGL chain significant in Cont model

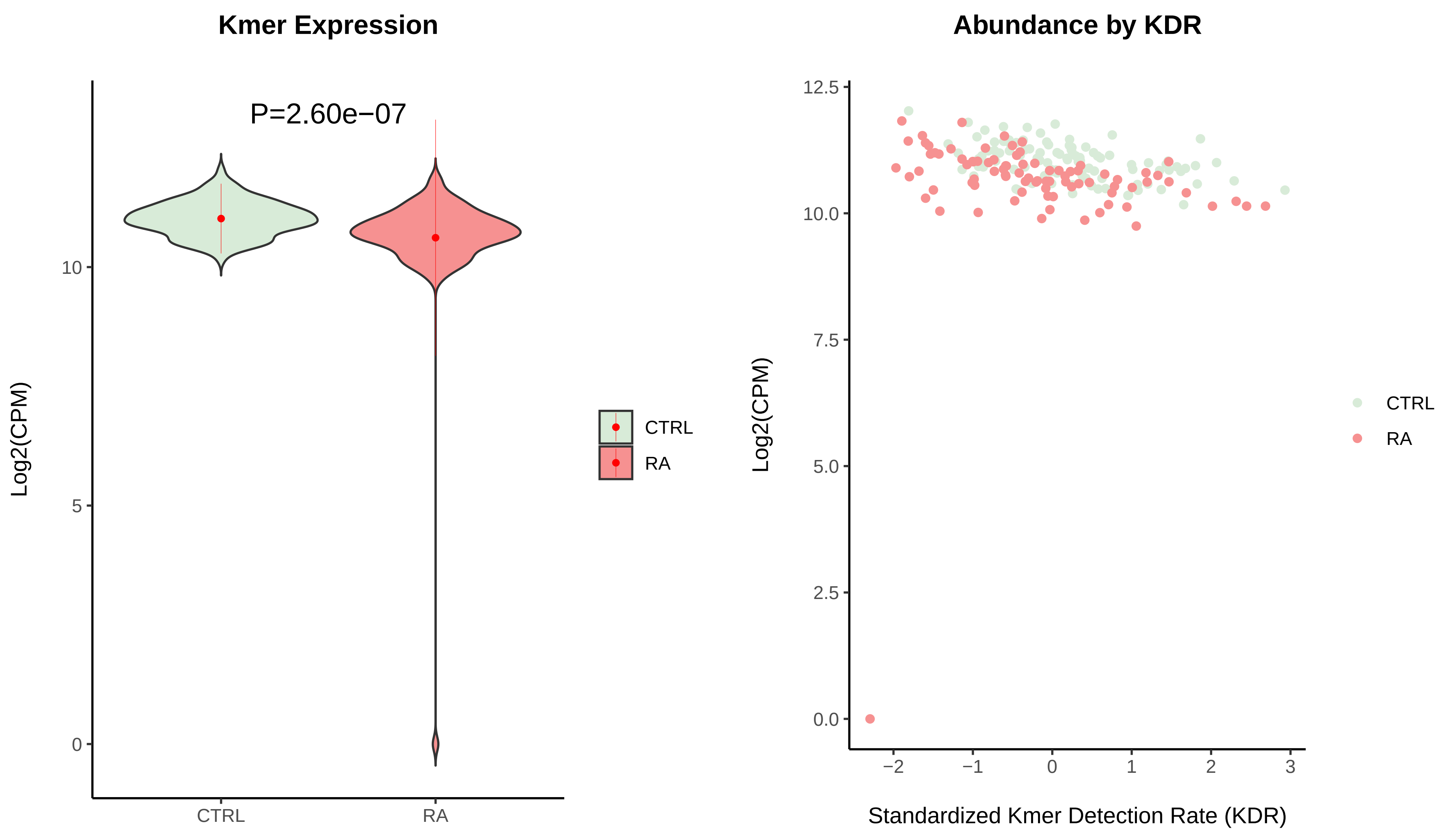

GADH from IGL chain significant in Cont model

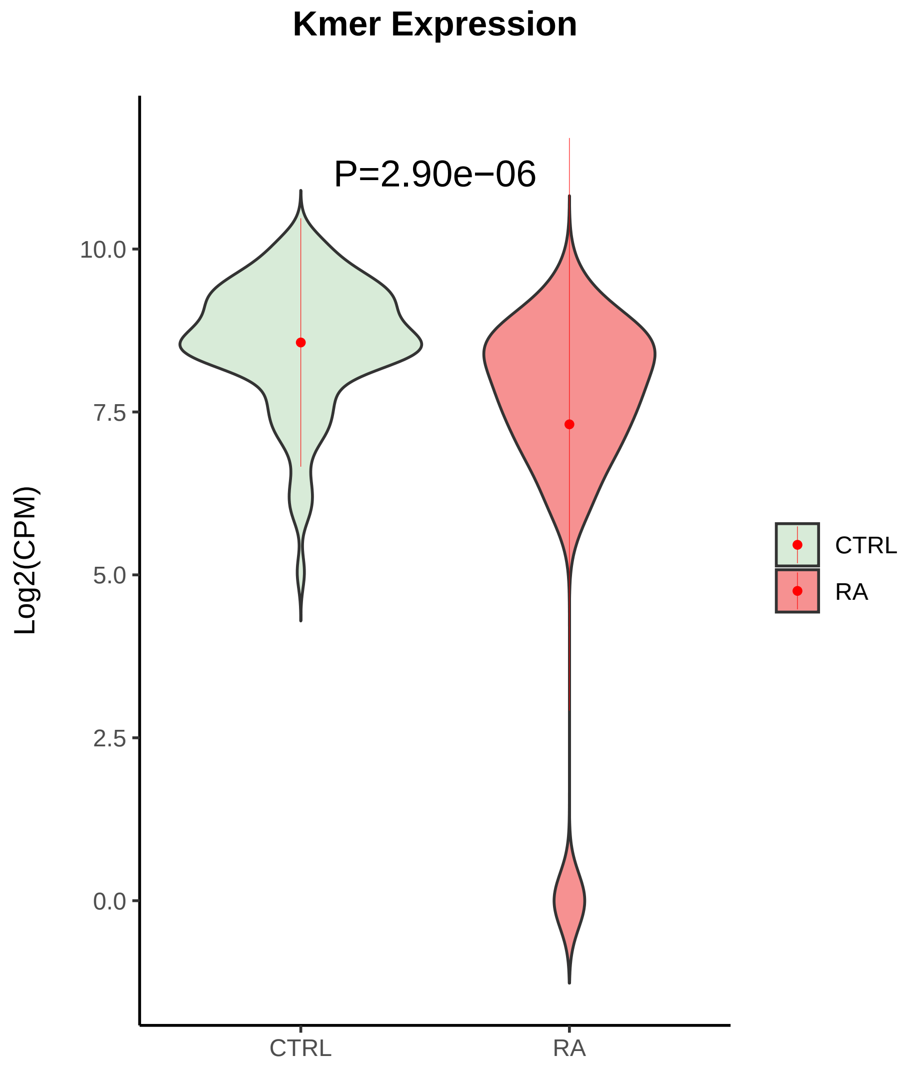

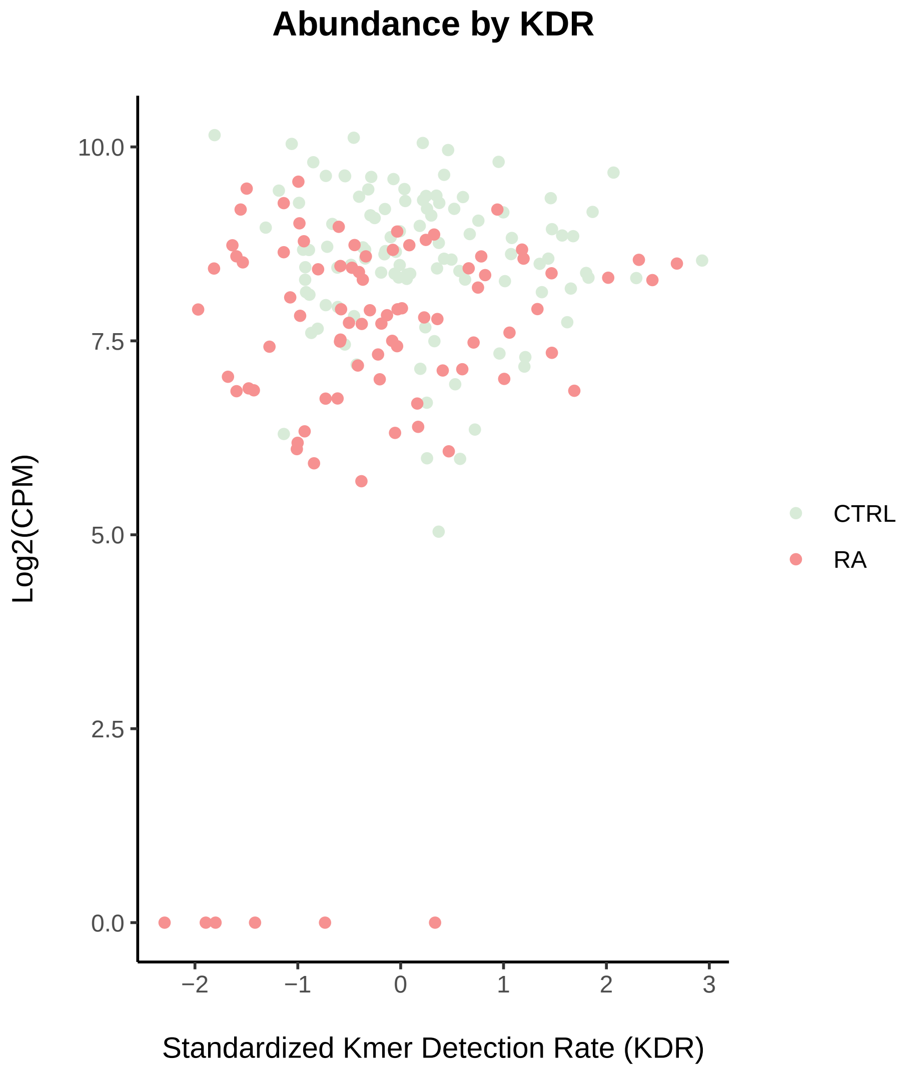

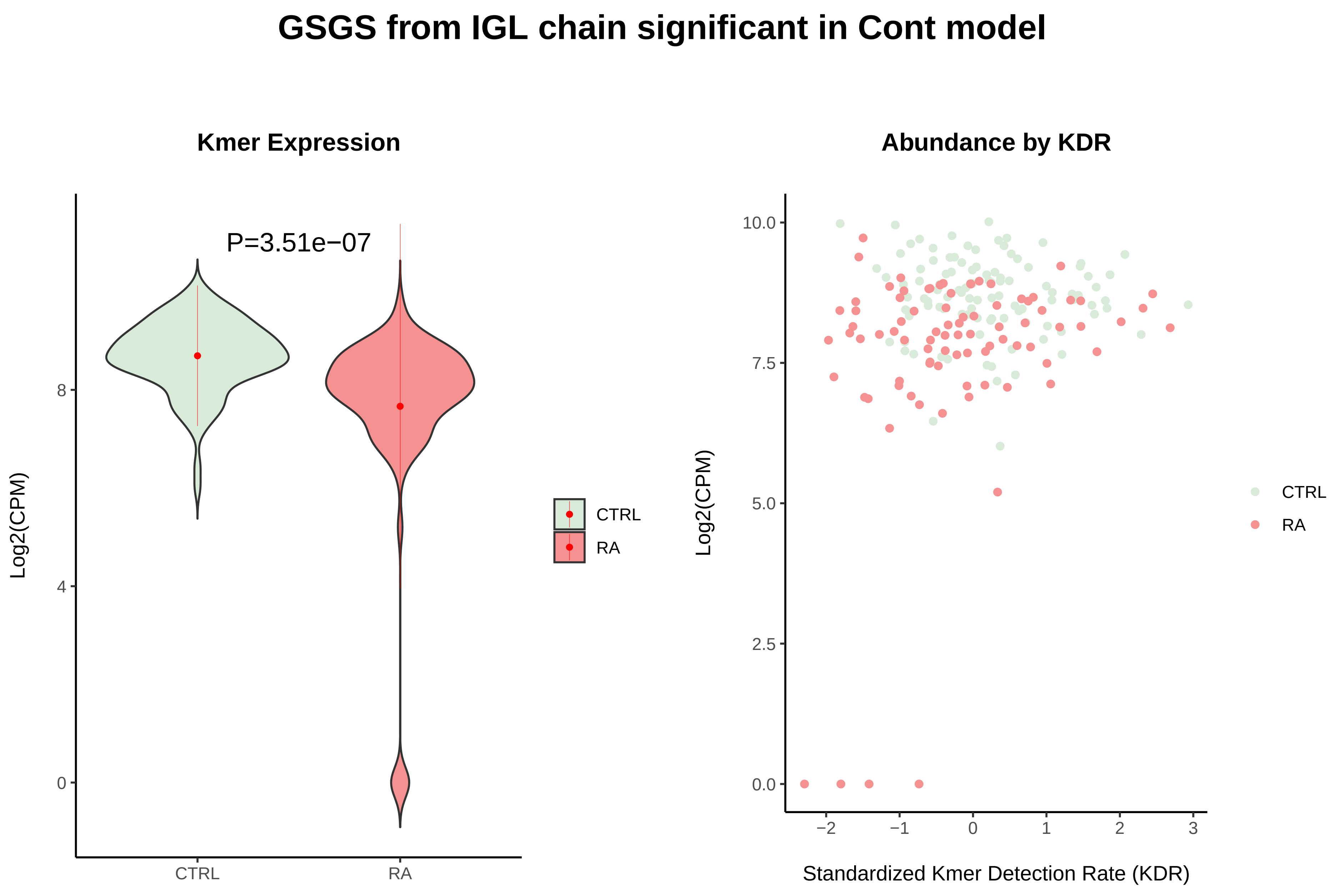

GSGS from IGL chain significant in Cont model
Kmer Expression
P=3.51e−07
Log2(CPM)
8
4
0
CTRL
RA
CTRL
RA
Abundance by KDR
Log2(CPM)
10.0
7.5
5.0
2.5
0.0
−2
−1
0
1
2
3
Standardized Kmer Detection Rate (KDR)
CTRL
RA

GSNF from IGL chain significant in Cont model

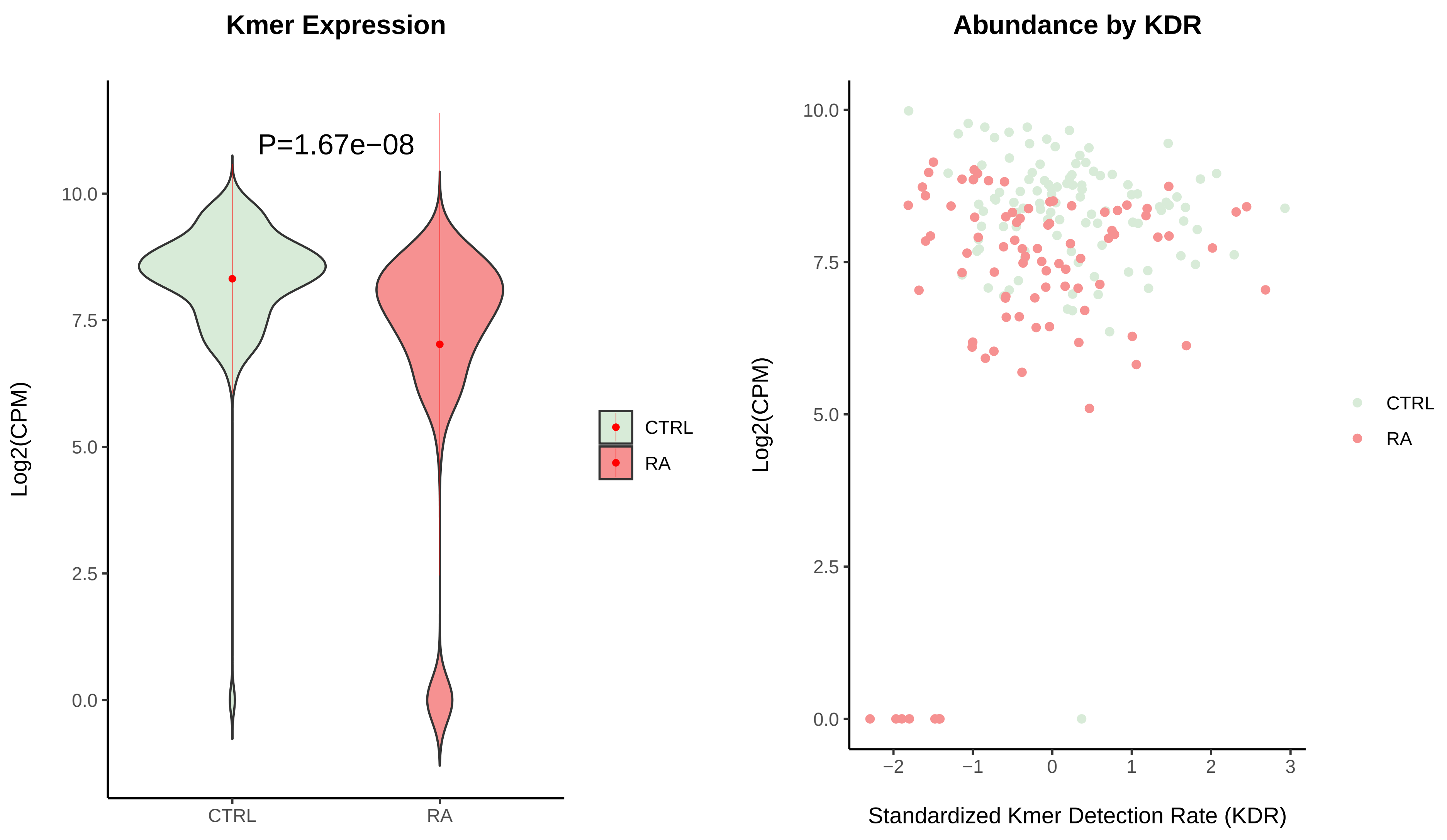

HGSG from IGL chain significant in Cont model

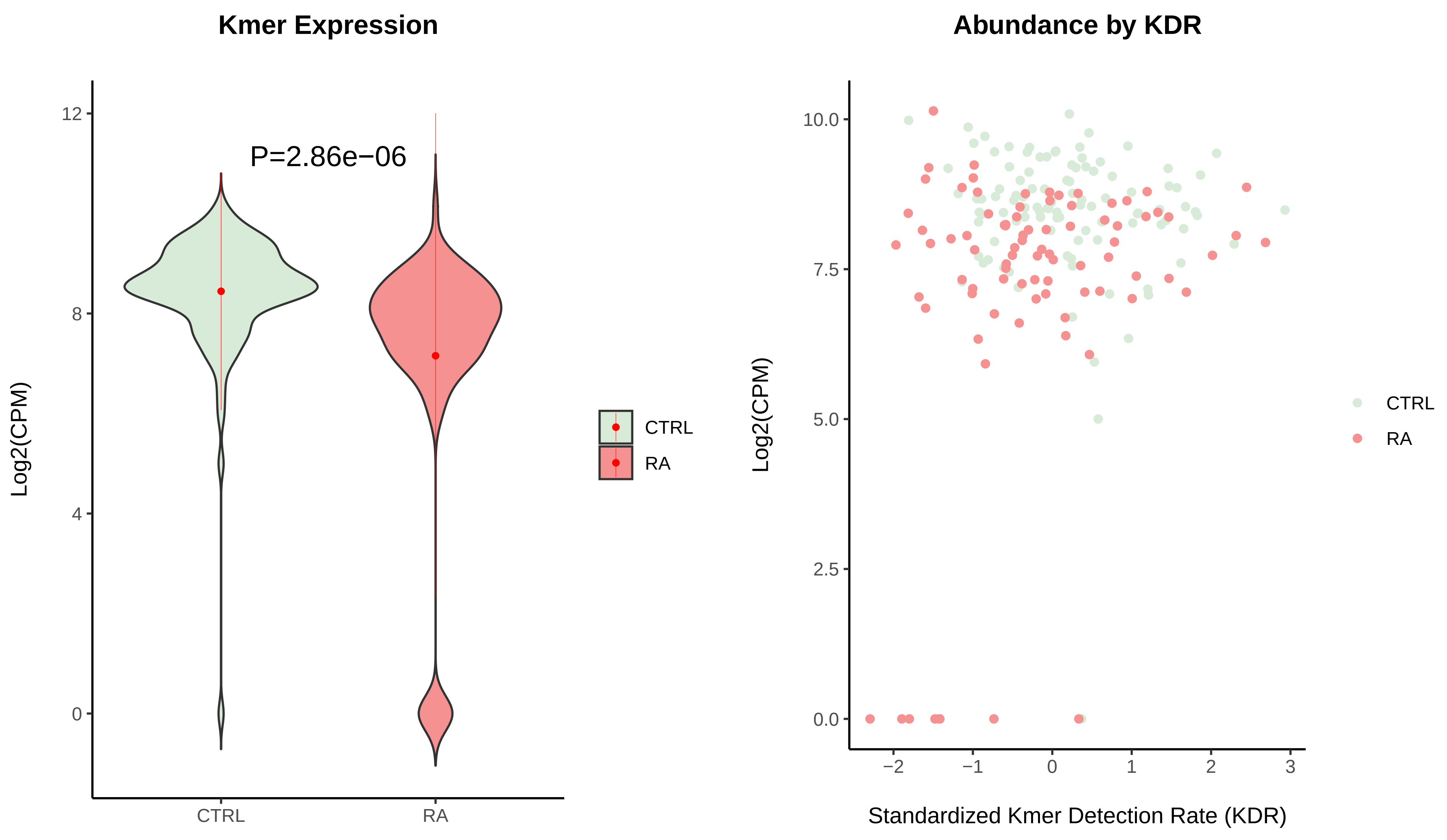

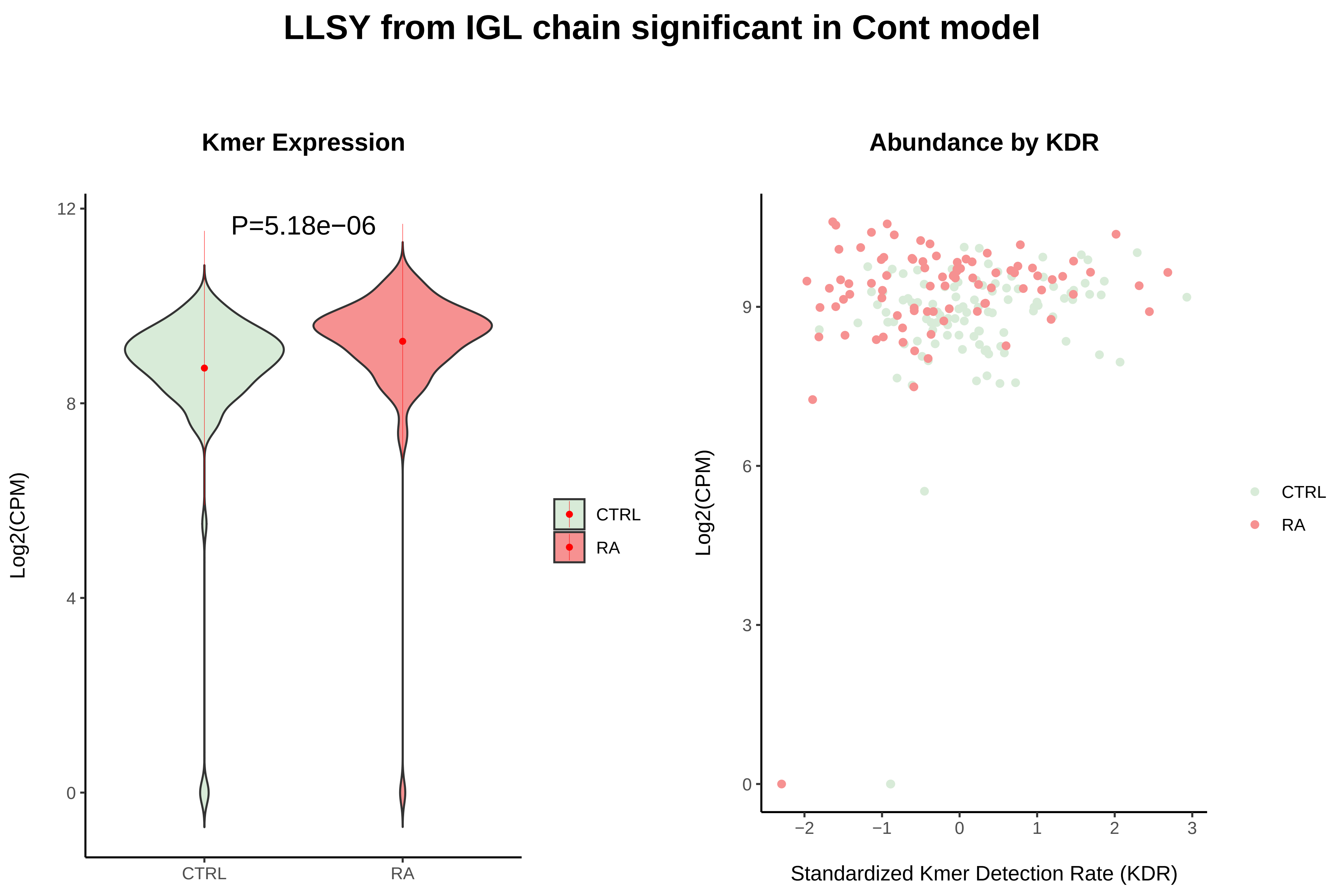
LLSY from IGL chain significant in Cont model
Kmer Expression
P=5.18e−06
Log2(CPM)
12
8
4
0
CTRL
RA
CTRL
RA
Abundance by KDR
Log2(CPM)
9
6
3
0
−2
−1
0
1
2
3
Standardized Kmer Detection Rate (KDR)
CTRL
RA

NRLS from IGL chain significant in Cont model

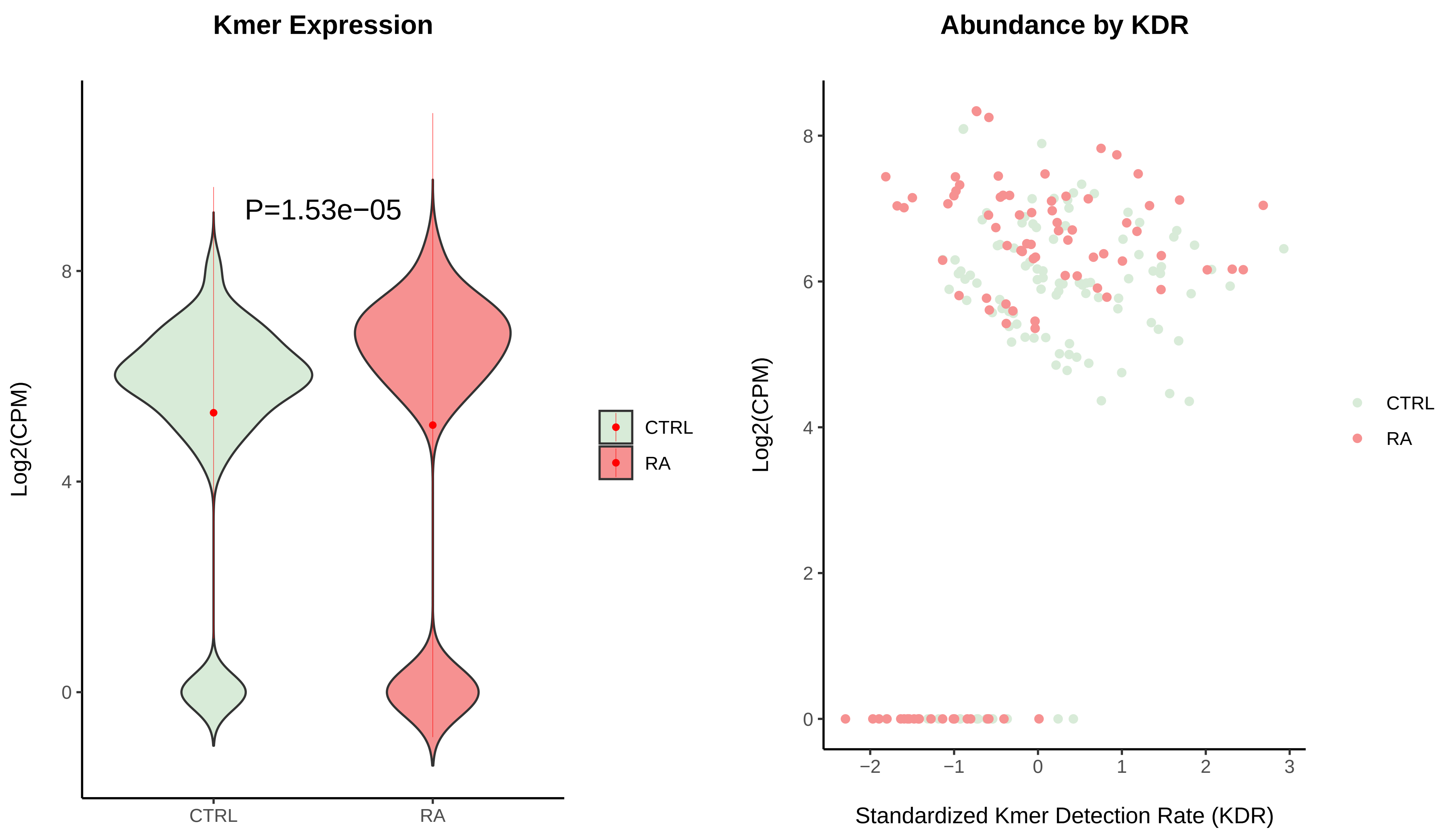

QVWD from IGL chain significant in Cont model

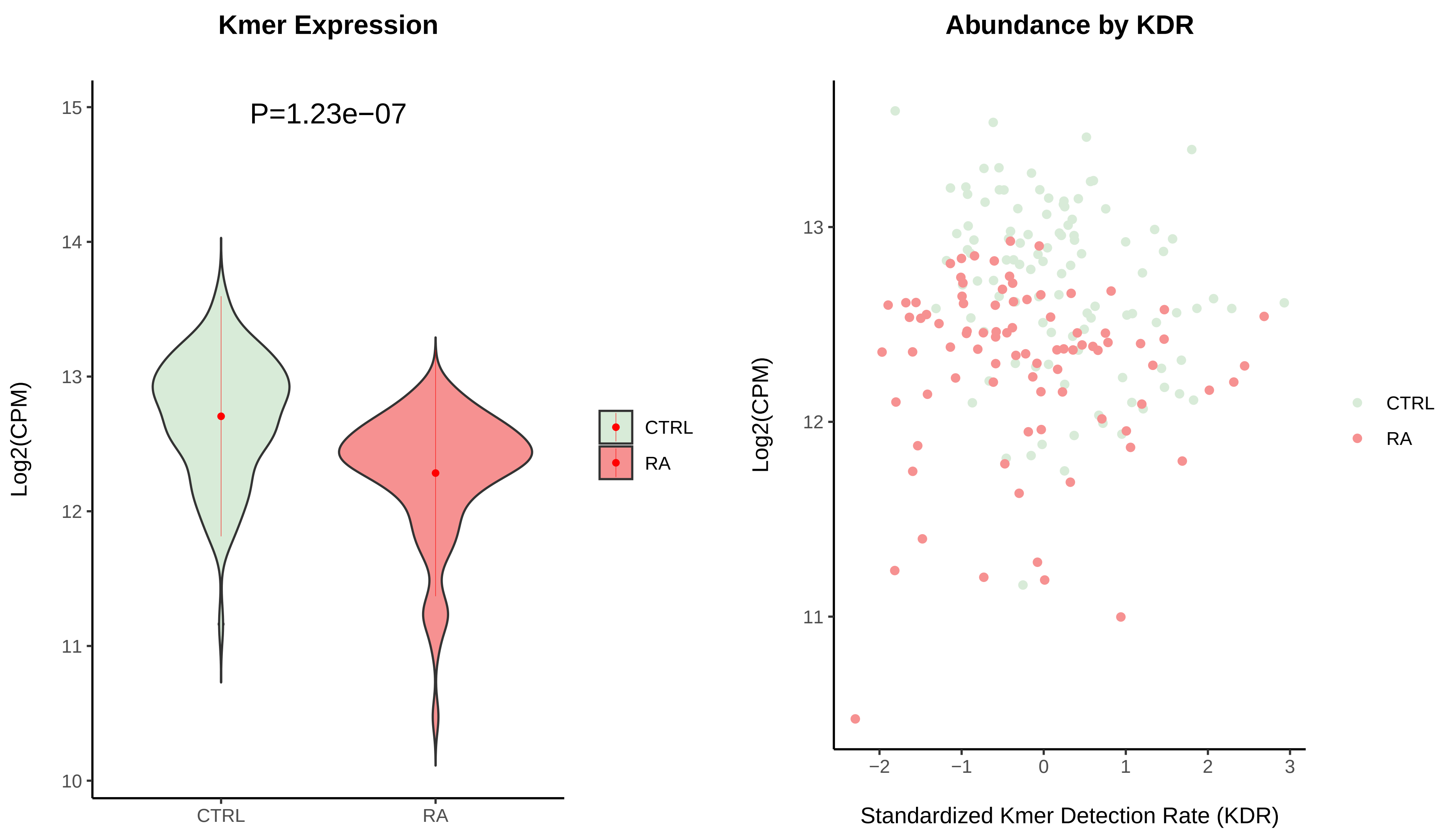

RDNT from IGL chain significant in Cont model

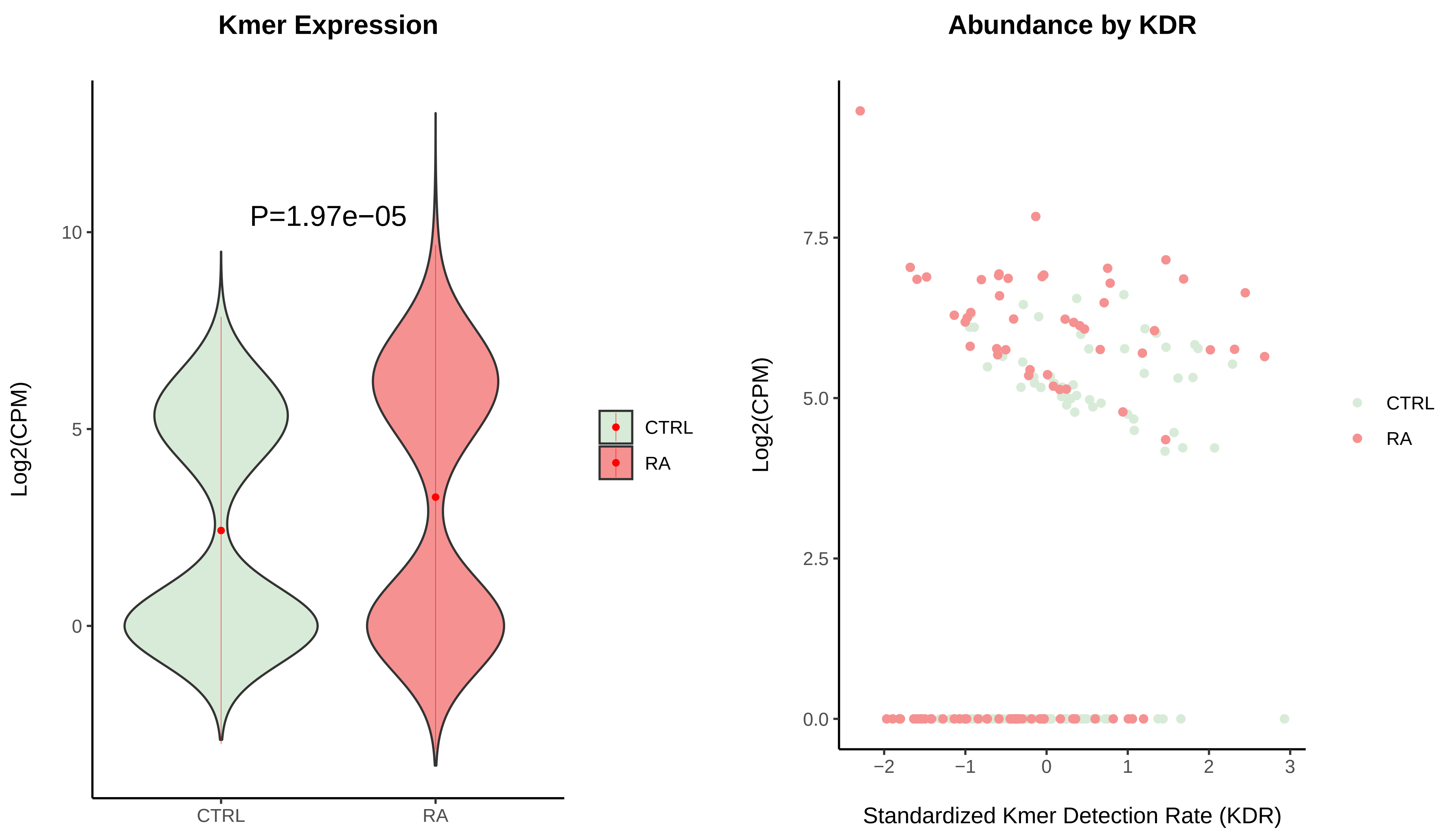

SDHP from IGL chain significant in Cont model

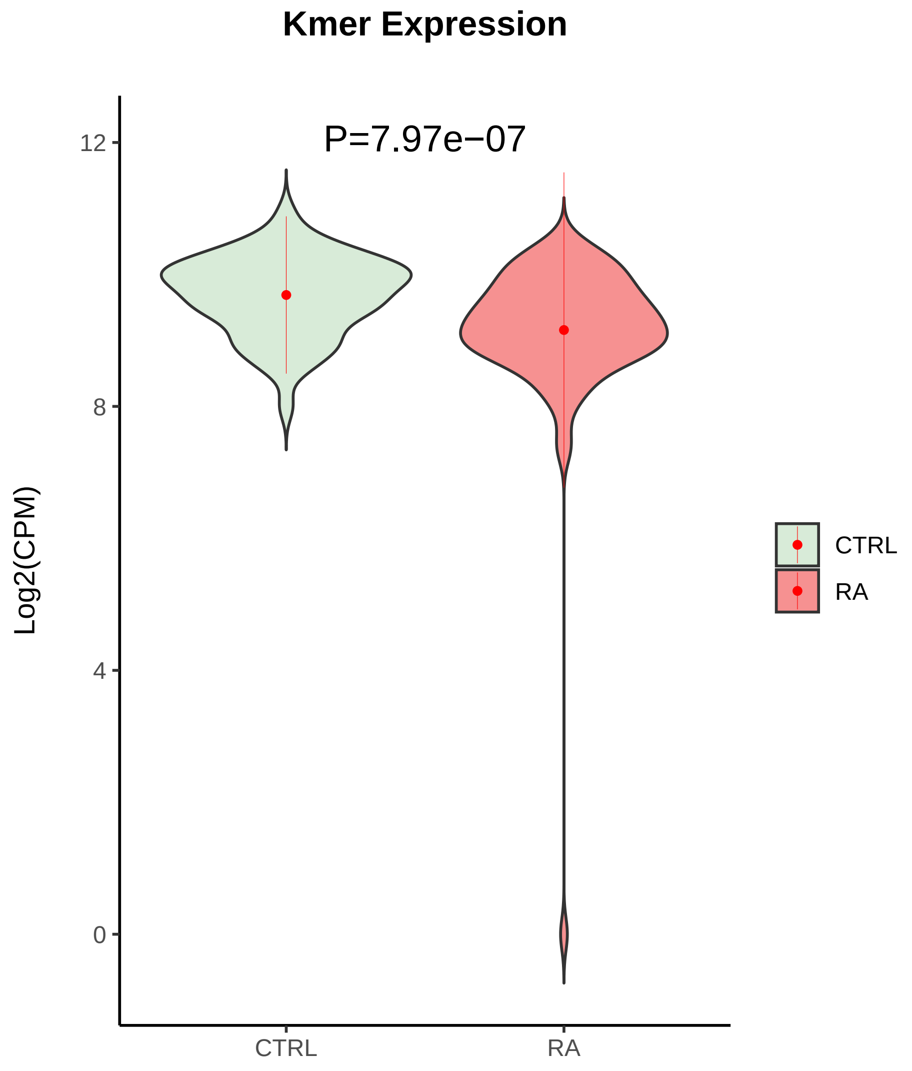

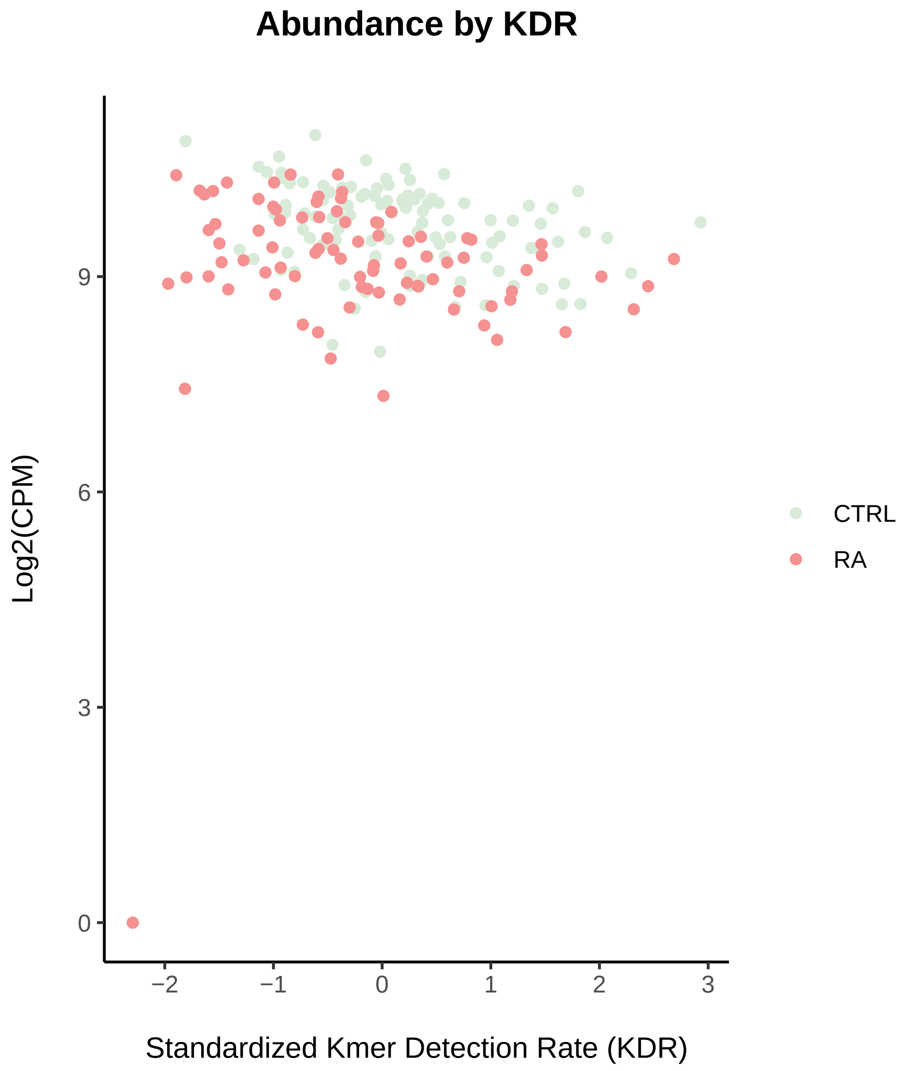

SGSN from IGL chain significant in Cont model

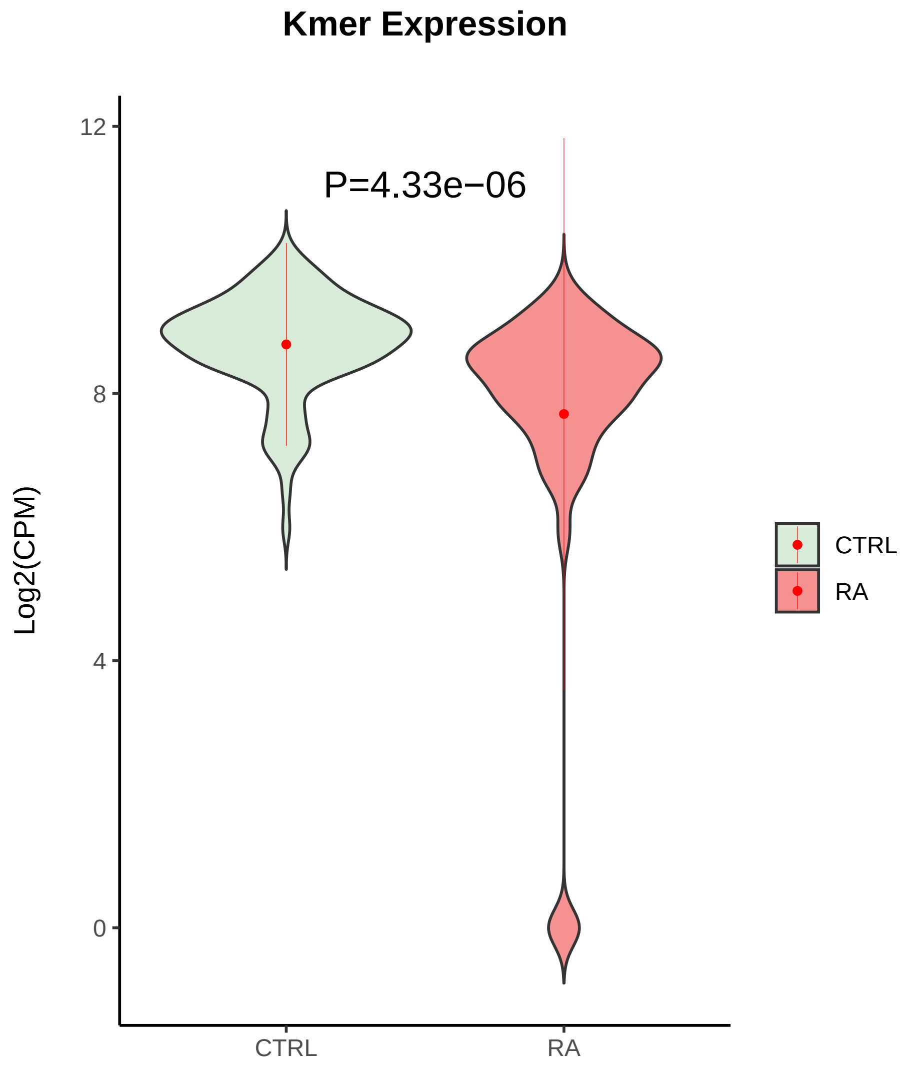

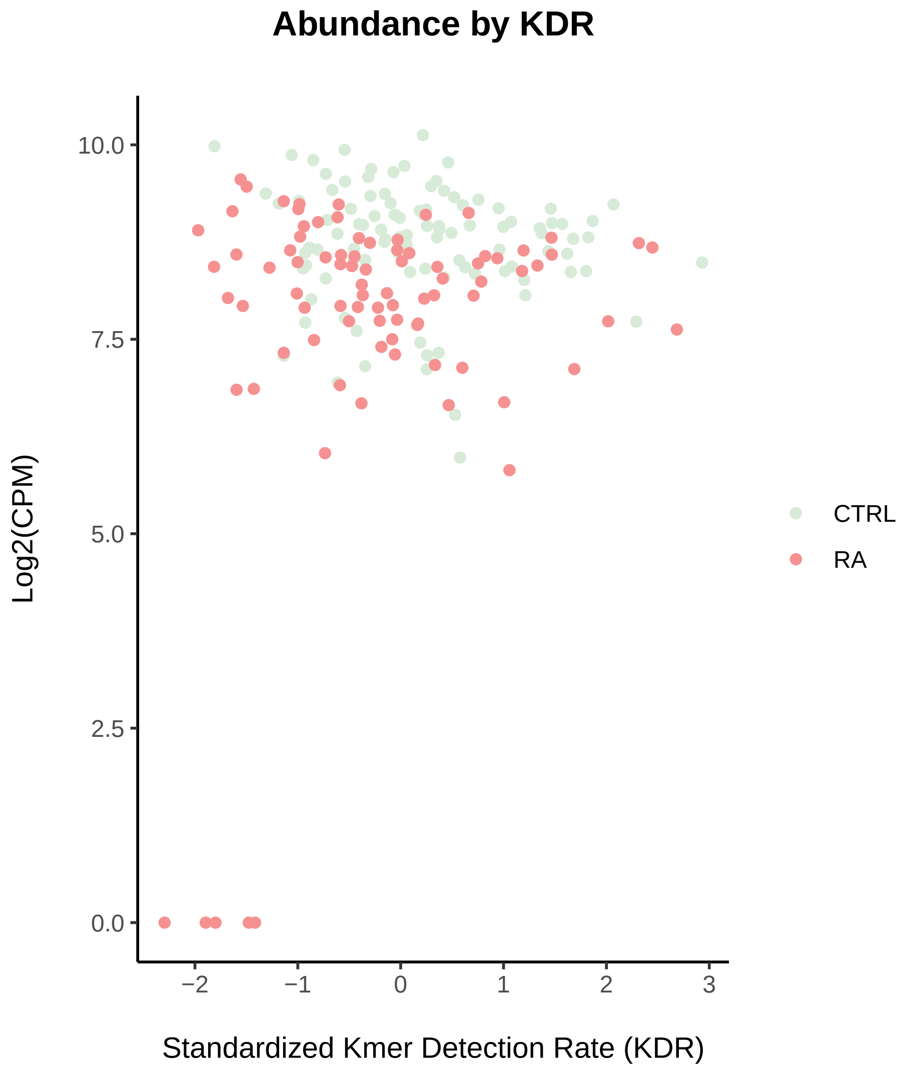

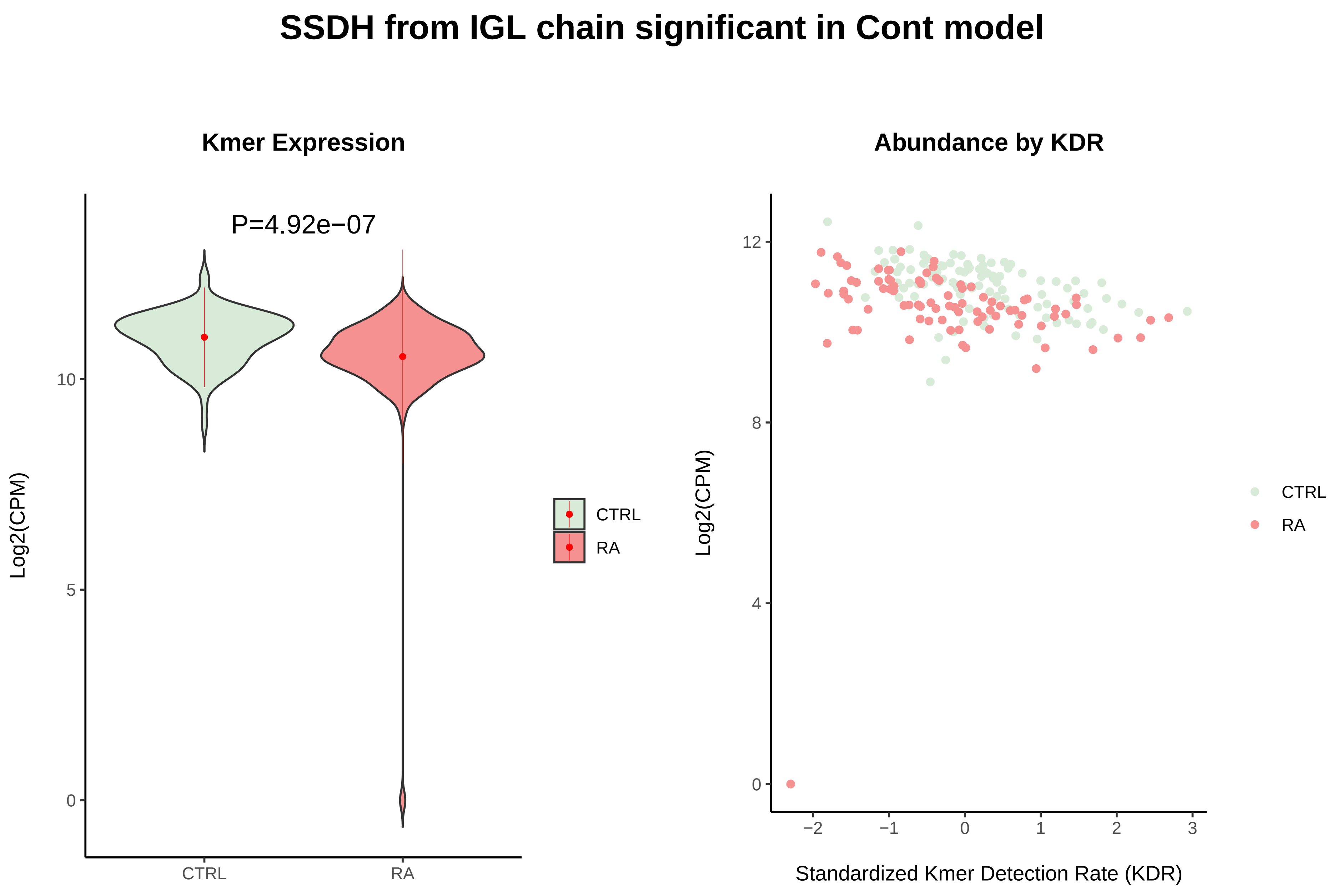
SSDH from IGL chain significant in Cont model
Kmer Expression
P=4.92e−07
Log2(CPM)
0
5
10
CTRL
RA
CTRL
RA
Abundance by KDR
Log2(CPM)
0
4
8
12
−2
−1
0
1
2
3
Standardized Kmer Detection Rate (KDR)
CTRL
RA

SSDQ from IGL chain significant in Cont model

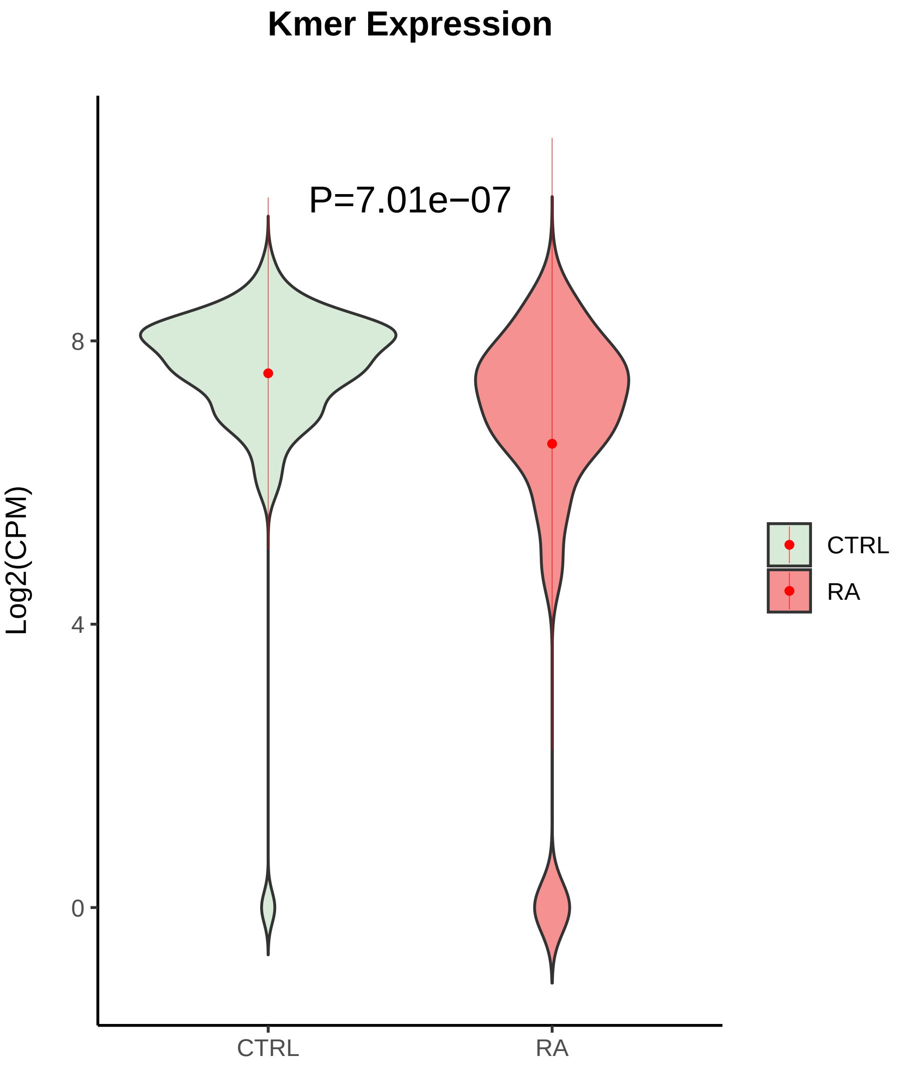

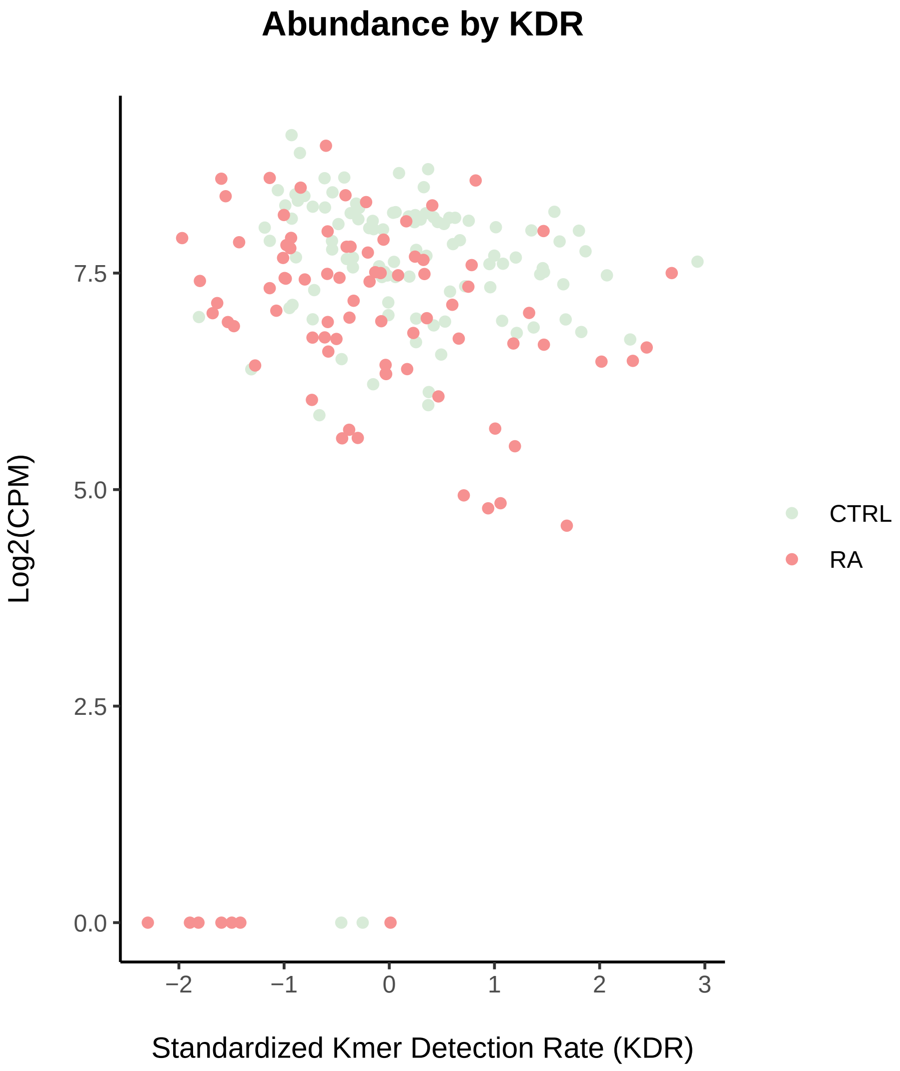

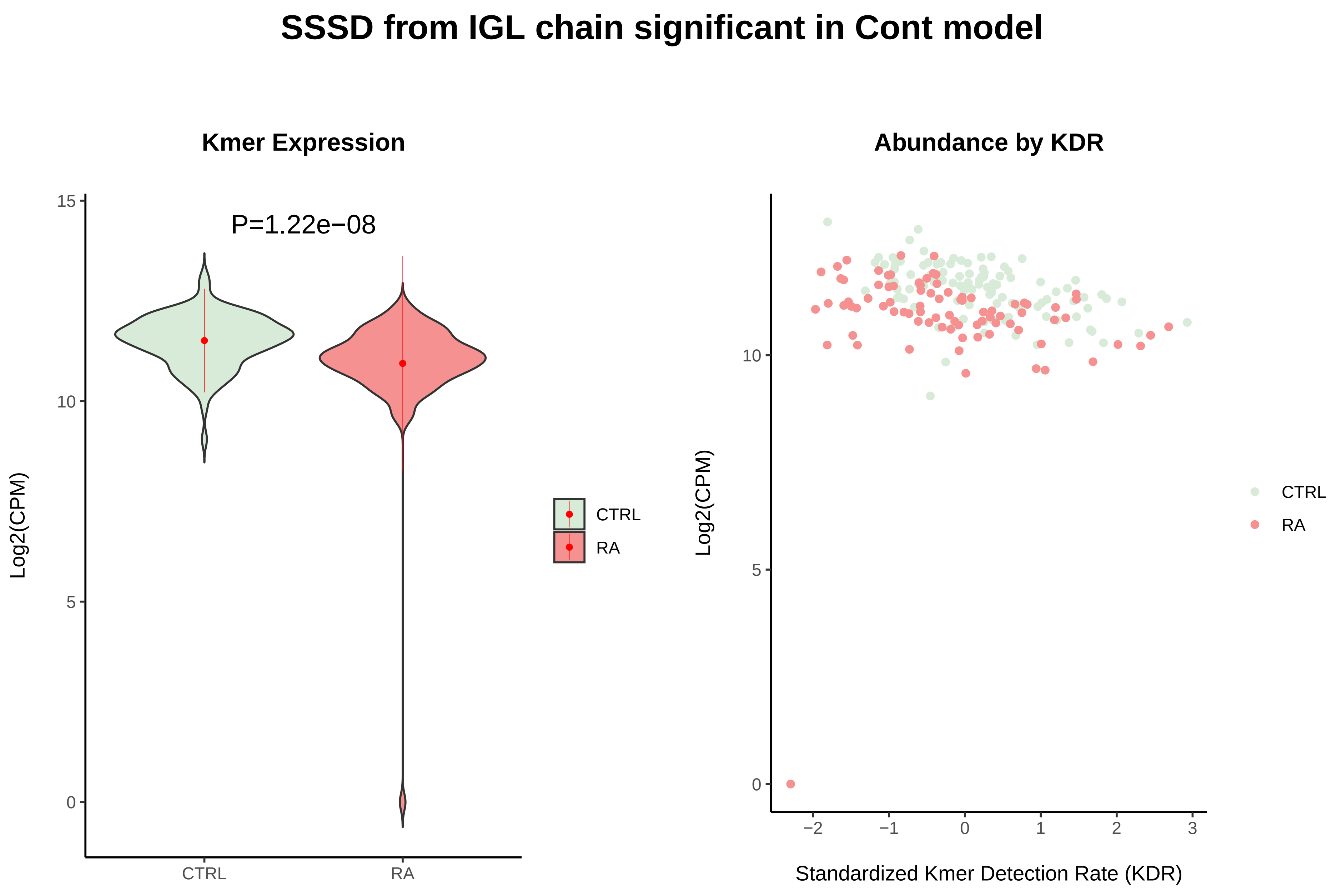

SSSD from IGL chain significant in Cont model
Kmer Expression
P=1.22e−08
Log2(CPM)
15
10
5
0
CTRL
RA
CTRL
RA
Abundance by KDR
Log2(CPM)
10
5
0
−2
−1
0
1
2
3
Standardized Kmer Detection Rate (KDR)
CTRL
RA

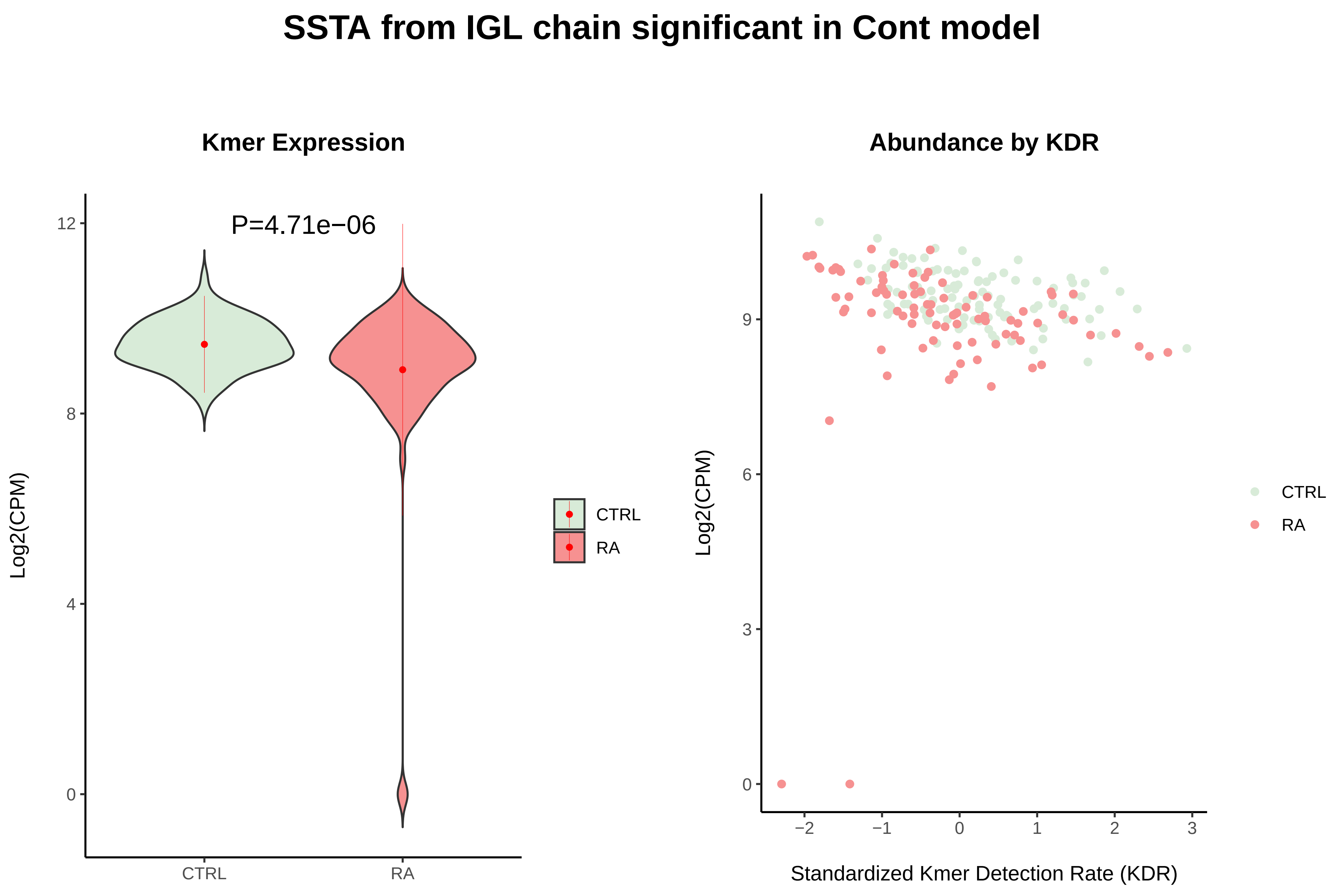

SSTA from IGL chain significant in Cont model
Kmer Expression
P=4.71e−06
Log2(CPM)
12
8
4
0
CTRL
RA
CTRL
RA
Abundance by KDR
Log2(CPM)
9
6
3
0
−2
−1
0
1
2
3
Standardized Kmer Detection Rate (KDR)
CTRL
RA

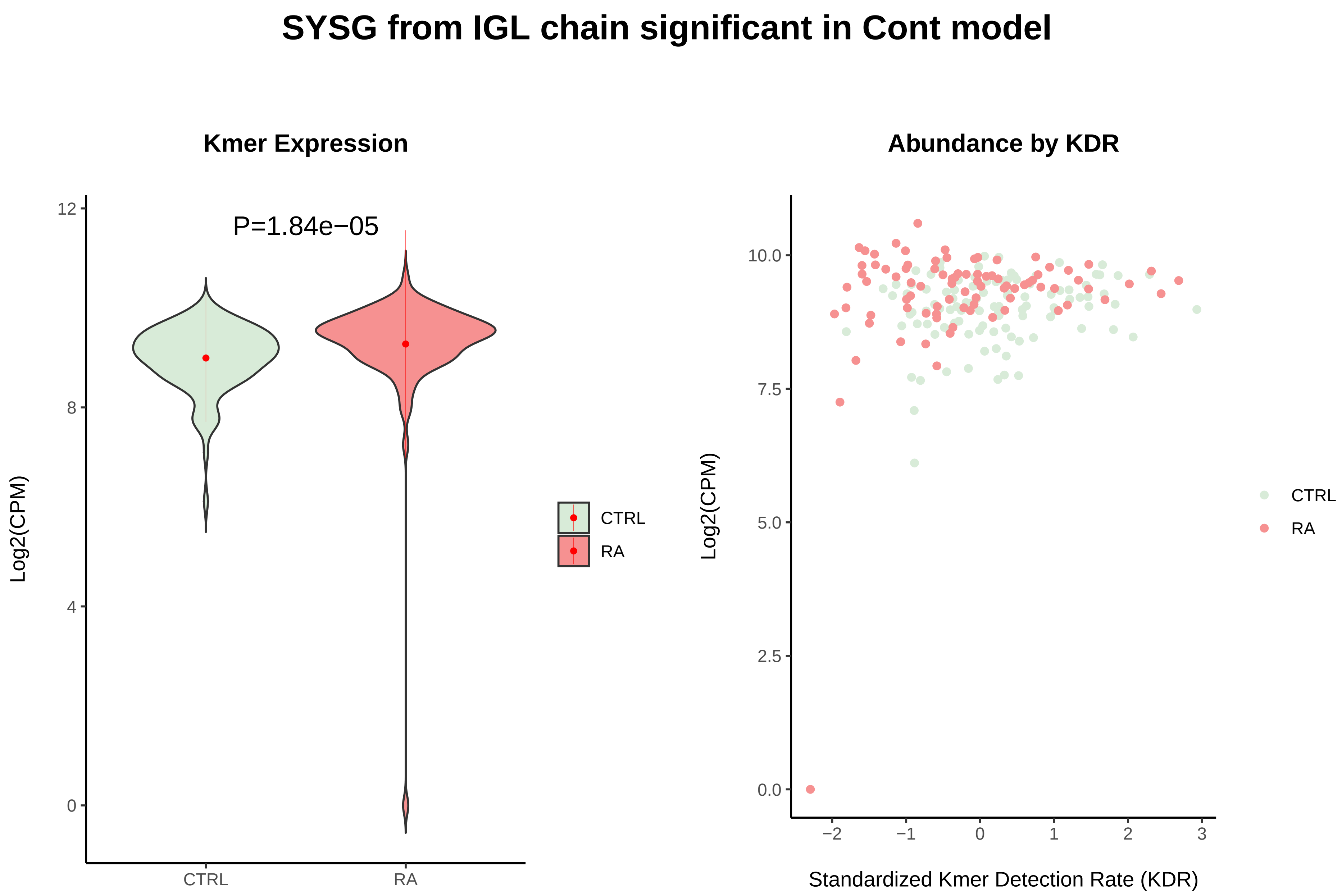
SYSG from IGL chain significant in Cont model
Kmer Expression
P=1.84e−05
Log2(CPM)
12
8
4
0
CTRL
RA
CTRL
RA
Abundance by KDR
Log2(CPM)
10.0
7.5
5.0
2.5
0.0
−2
−1
0
1
2
3
Standardized Kmer Detection Rate (KDR)
CTRL
RA

VWDS from IGL chain significant in Cont model

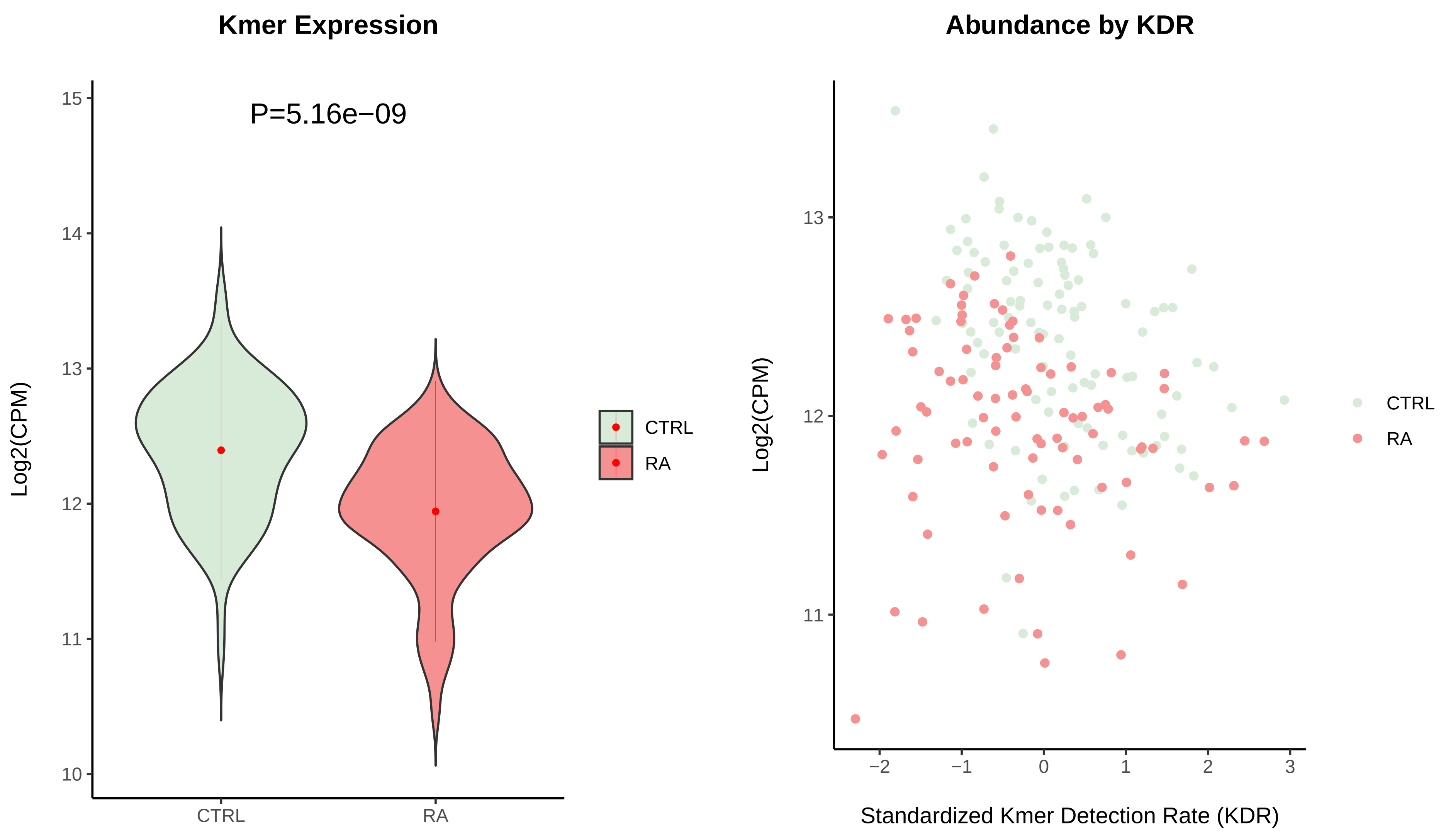

WDSS from IGL chain significant in Cont model

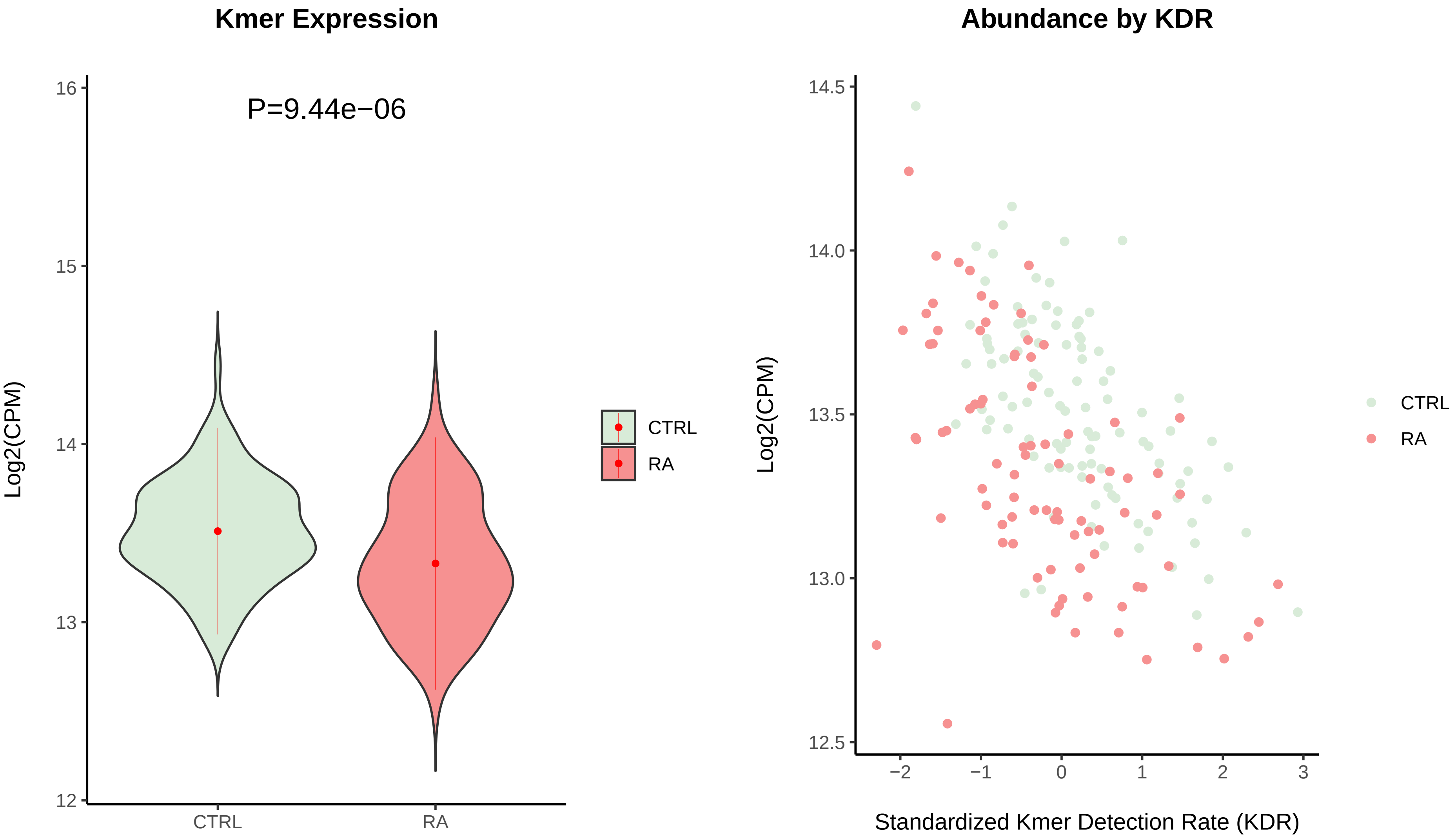

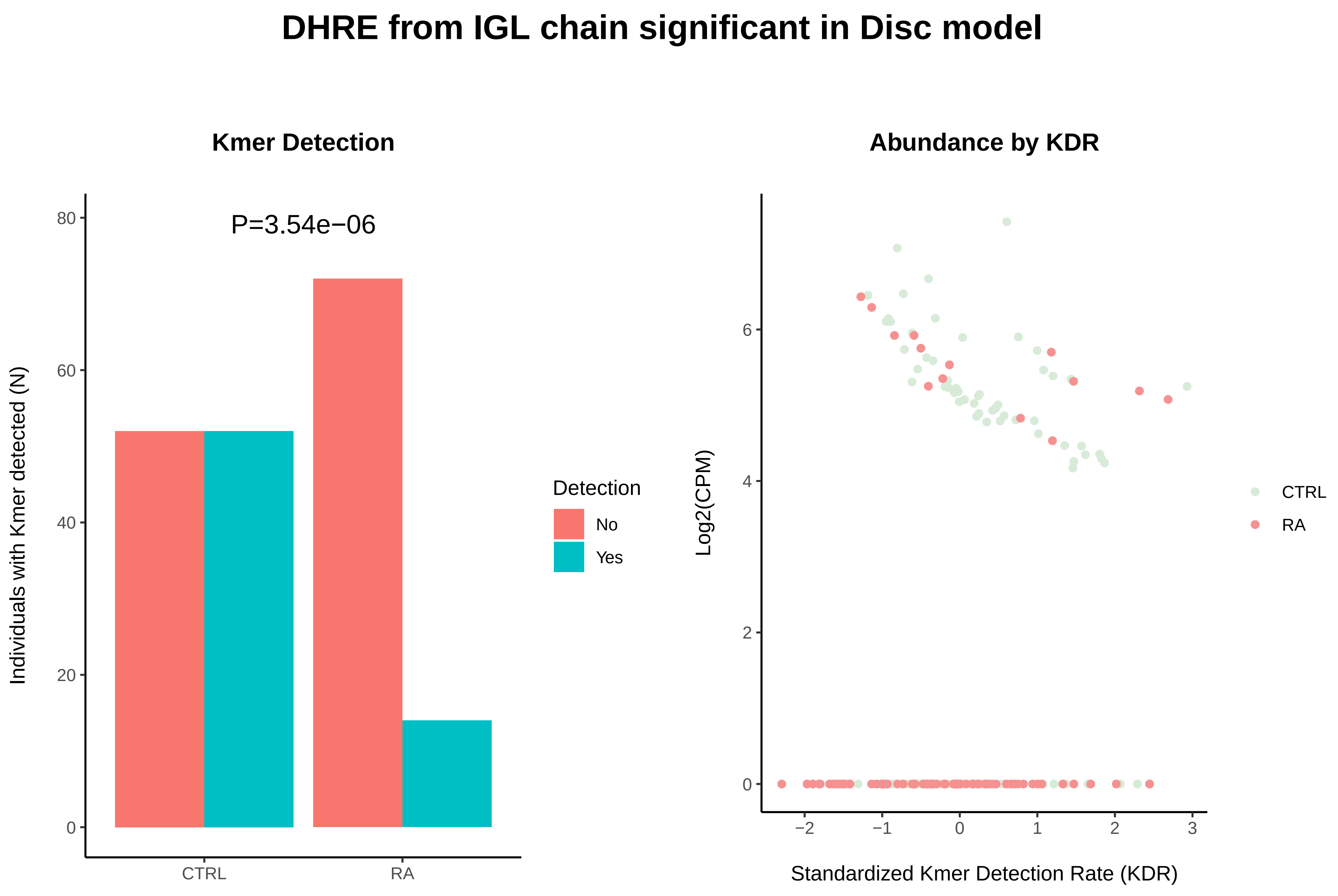
DHRE from IGL chain significant in Disc model
Kmer Detection
P=3.54e−06
Individuals with Kmer detected (N)
80
60
40
20
0
CTRL
RA
Detection
No
Yes
Abundance by KDR
Log2(CPM)
6
4
2
0
−2
−1
0
1
2
3
Standardized Kmer Detection Rate (KDR)
CTRL
RA

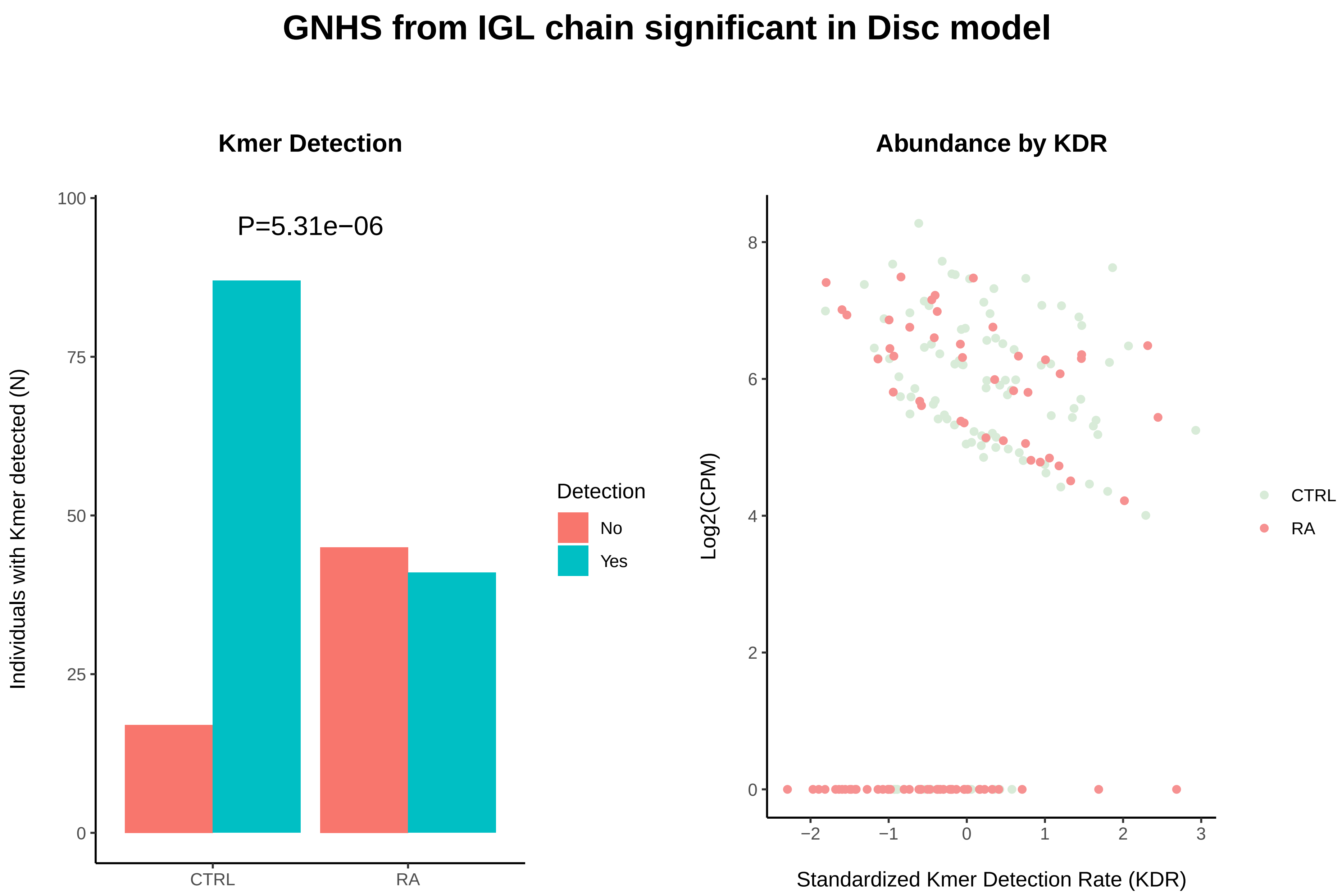
GNHS from IGL chain significant in Disc model
Kmer Detection
P=5.31e−06
Individuals with Kmer detected (N)
100
75
50
25
0
CTRL
RA
Detection
No
Yes
Abundance by KDR
Log2(CPM)
8
6
4
2
0
−2
−1
0
1
2
3
Standardized Kmer Detection Rate (KDR)
CTRL
RA

SNFG from IGL chain significant in Disc model

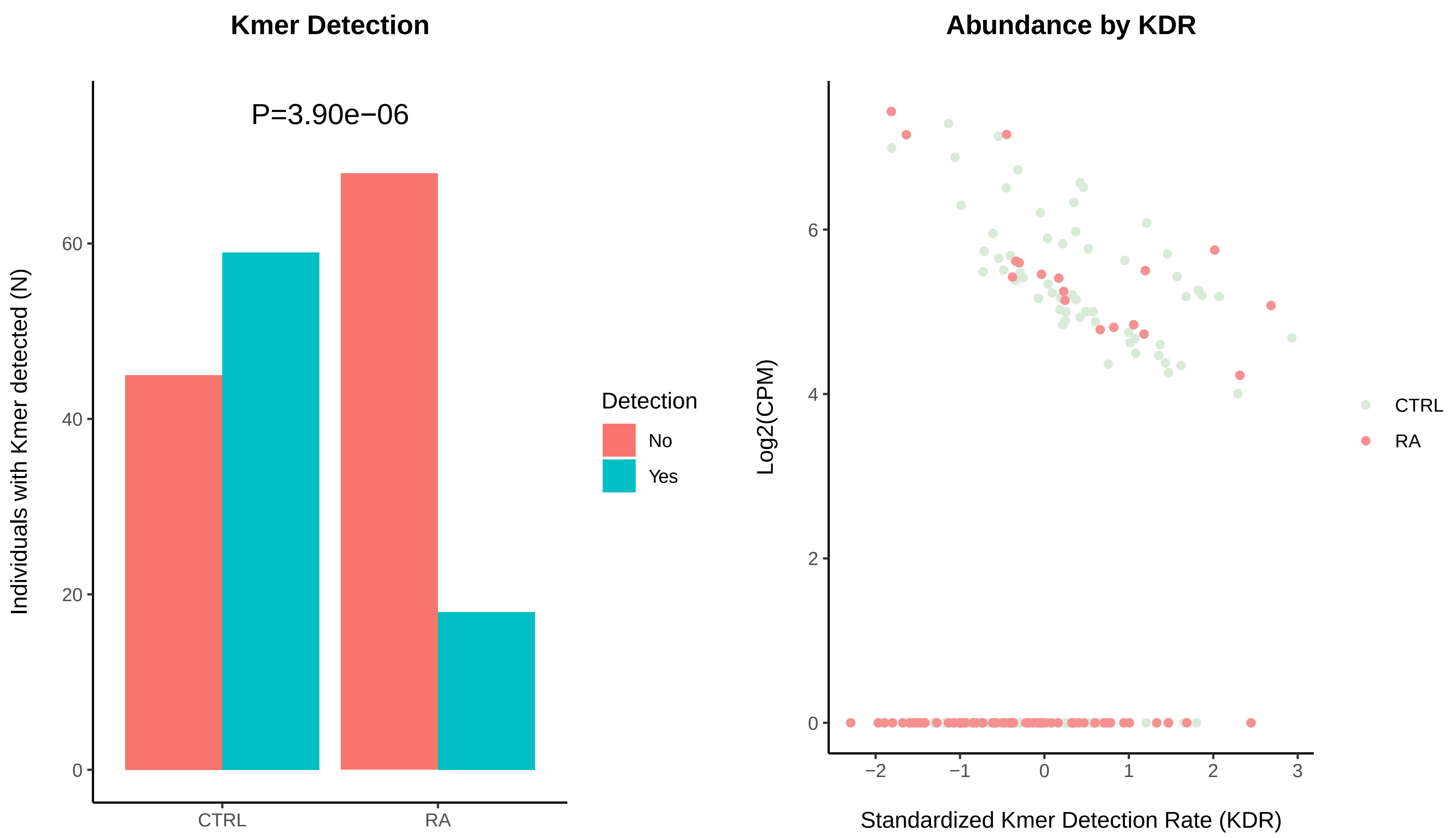

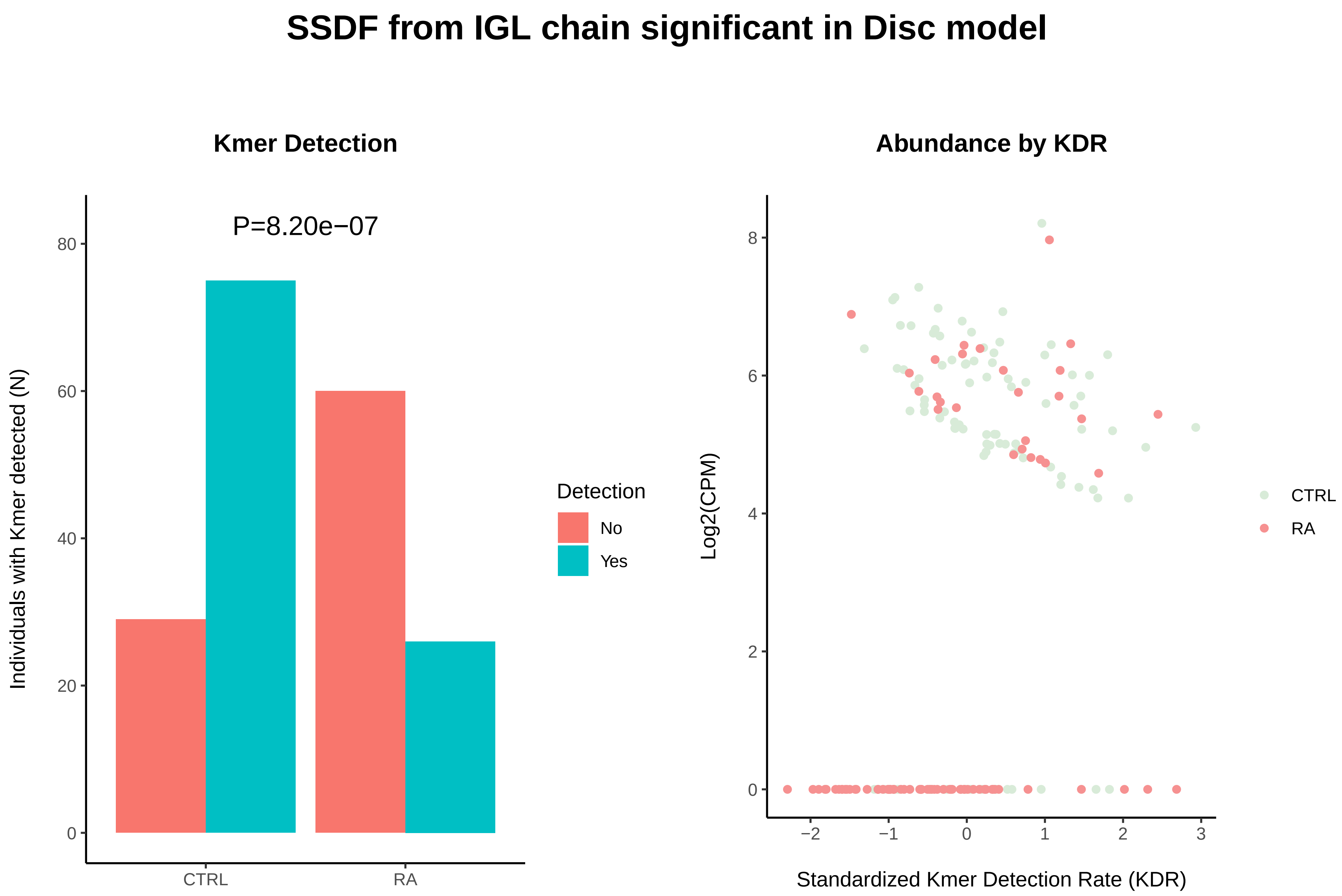

SSDF from IGL chain significant in Disc model
Kmer Detection
P=8.20e−07
Individuals with Kmer detected (N)
80
60
40
20
0
CTRL
RA
Detection
No
Yes
Abundance by KDR
Log2(CPM)
8
6
4
2
0
−2
−1
0
1
2
3
Standardized Kmer Detection Rate (KDR)
CTRL
RA

WVVF from IGL chain significant in Disc model

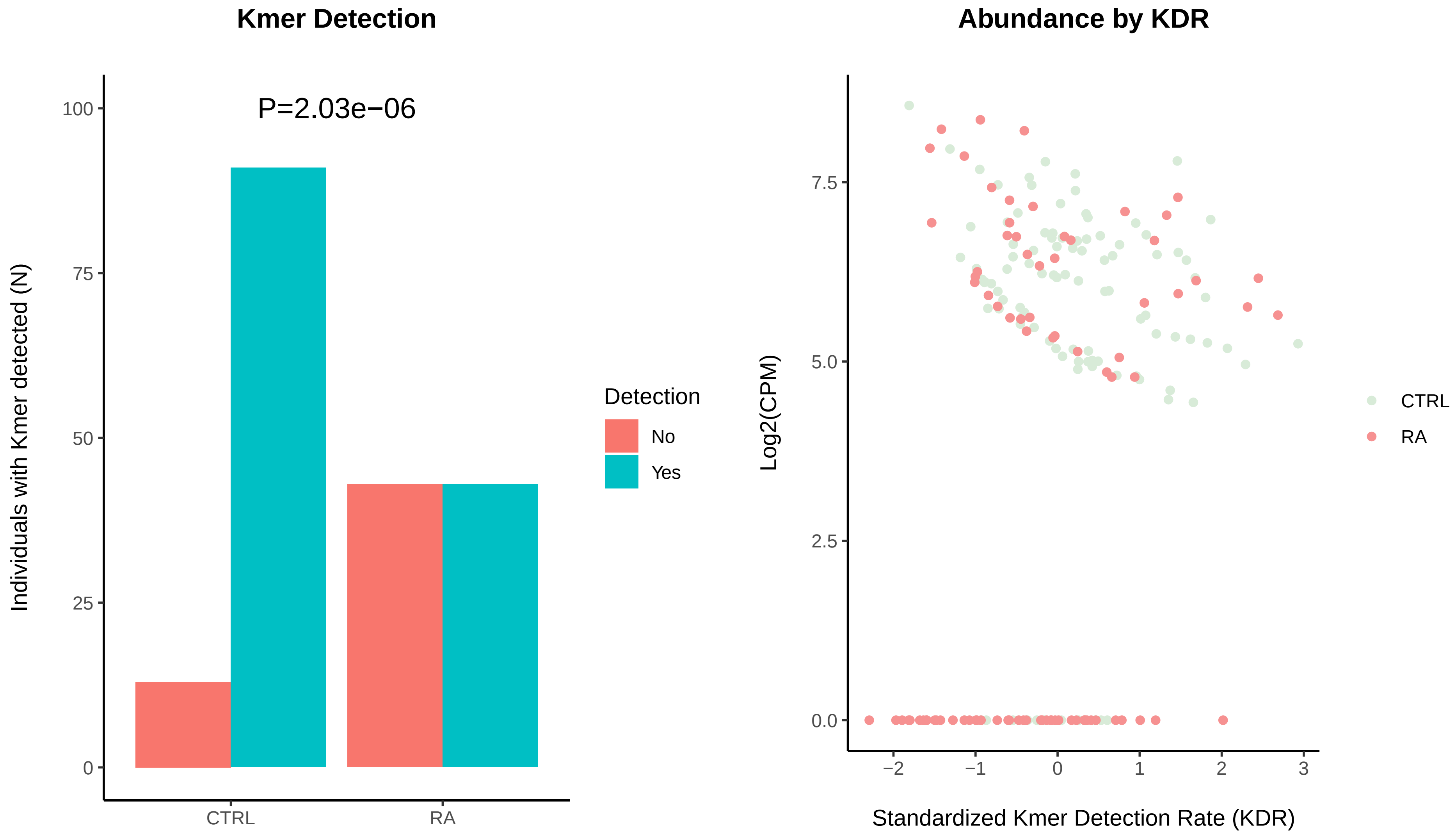

Supplement: Supplementary file 25 — Additional file 25: Figure S5. Graphical representation of the significant associations between TRA/TRB/IGL/IGK k-mers and rheumatoid arthritis. Significant associations detected by the Hurdle or continuous models are represented using violin plots, where the k-mer expression is plotted separately for each phenotype. Significant associations detected by the discrete model are represented using bar plots, where the number of individuals with clones harboring the k-mer are plotted separately for each phenotype. For all significant associations (FDR<0.05), the k-mer expression is also plotted against the standardized k-mer detection rate. Abbreviations: Cont, continuous model; CPM, count per million on the logarithmic scale; CTRL, healthy individuals; Disc, discrete model; KDR, k-mer detection rate; P, p-value; RA, rheumatoid arthritis. [file 13059_2024_3210_MOESM25_ESM.pdf]
