# Supplementary material for: Seven-chain adaptive immune receptor repertoire analysis in rheumatoid arthritis reveals novel features associated with disease and clinically relevant phenotypes
Source: Genome Biol. 2024 Mar 11;25:68. doi: 10.1186/s13059-024-03210-0 (PMC10926600; doi:10.1186/s13059-024-03210-0)

Fig S6. Pairwise dissimilarity matrix among the amino acid sequences of TRA/TRB/IGL/IGK k-mers associated with rheumatoid arthritis. K-mers over- and under-represented in rheumatoid arthritis are represented in dark and light blue, respectively. The dissimilarity index was computed using the Levenshtein distance measure so that the higher the Levenshtein distance, the higher the dissimilarity index between two k-mer sequences. Abbreviations: Cont, continuous model; Disc, discrete model; RA, rheumatoid arthritis.

Kmers from IGK significant in Cont model

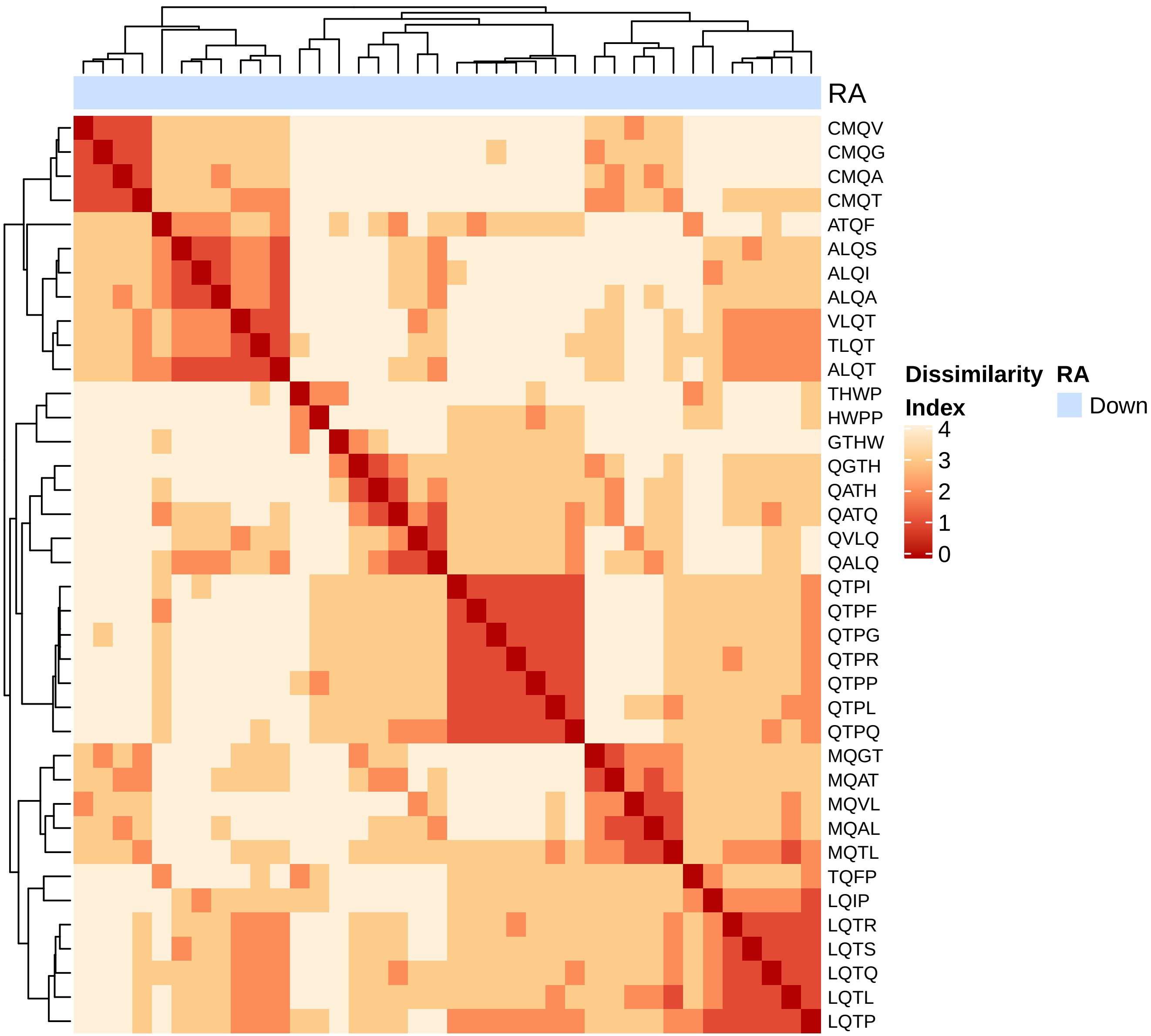

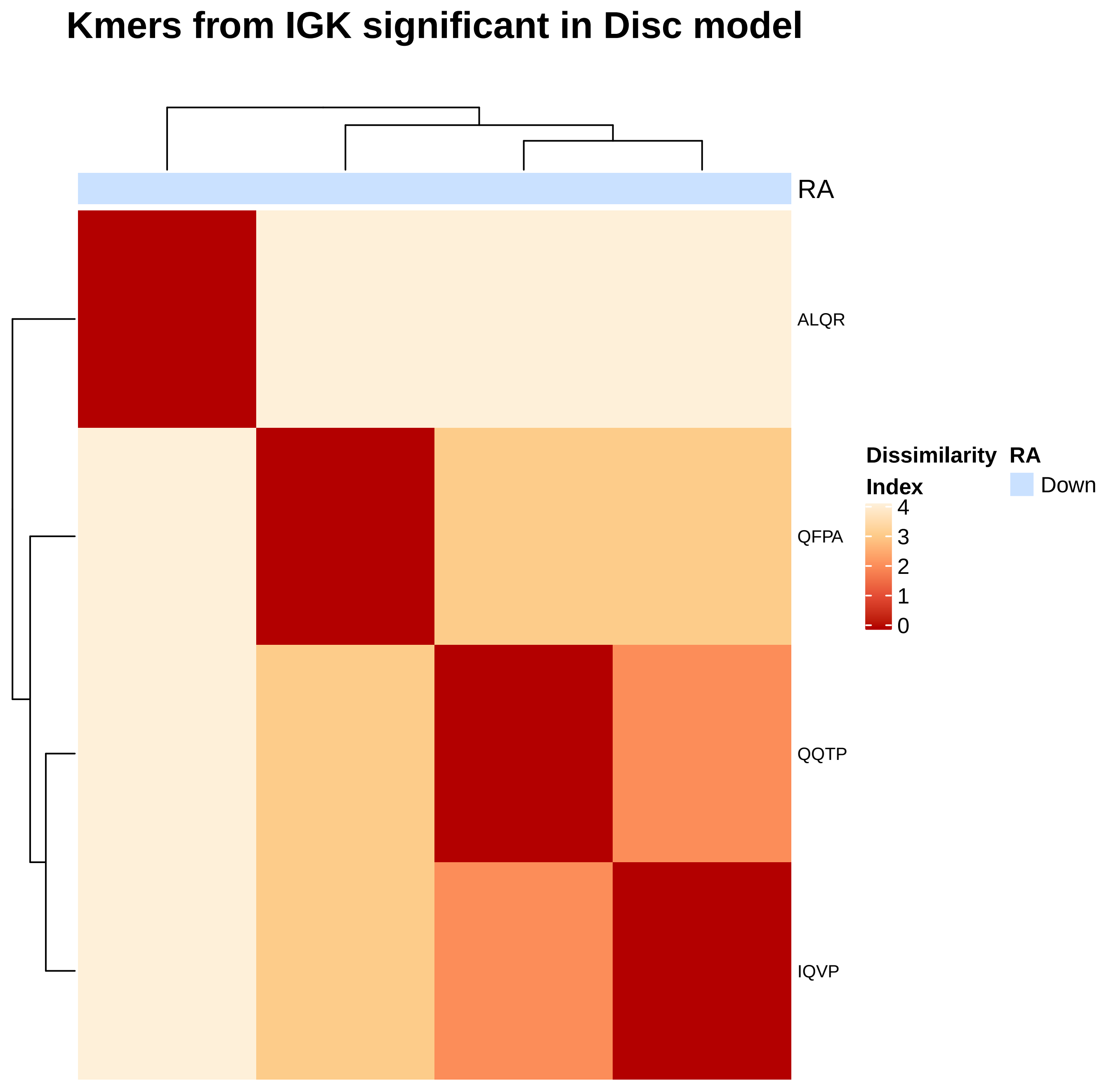
Kmers from IGK significant in Disc model
RA
ALQR
QFPA
QQTP
IQVP
Dissimilarity Index
4
3
2
1
0
RA
Down

Kmers from IGK significant in Hurdle model

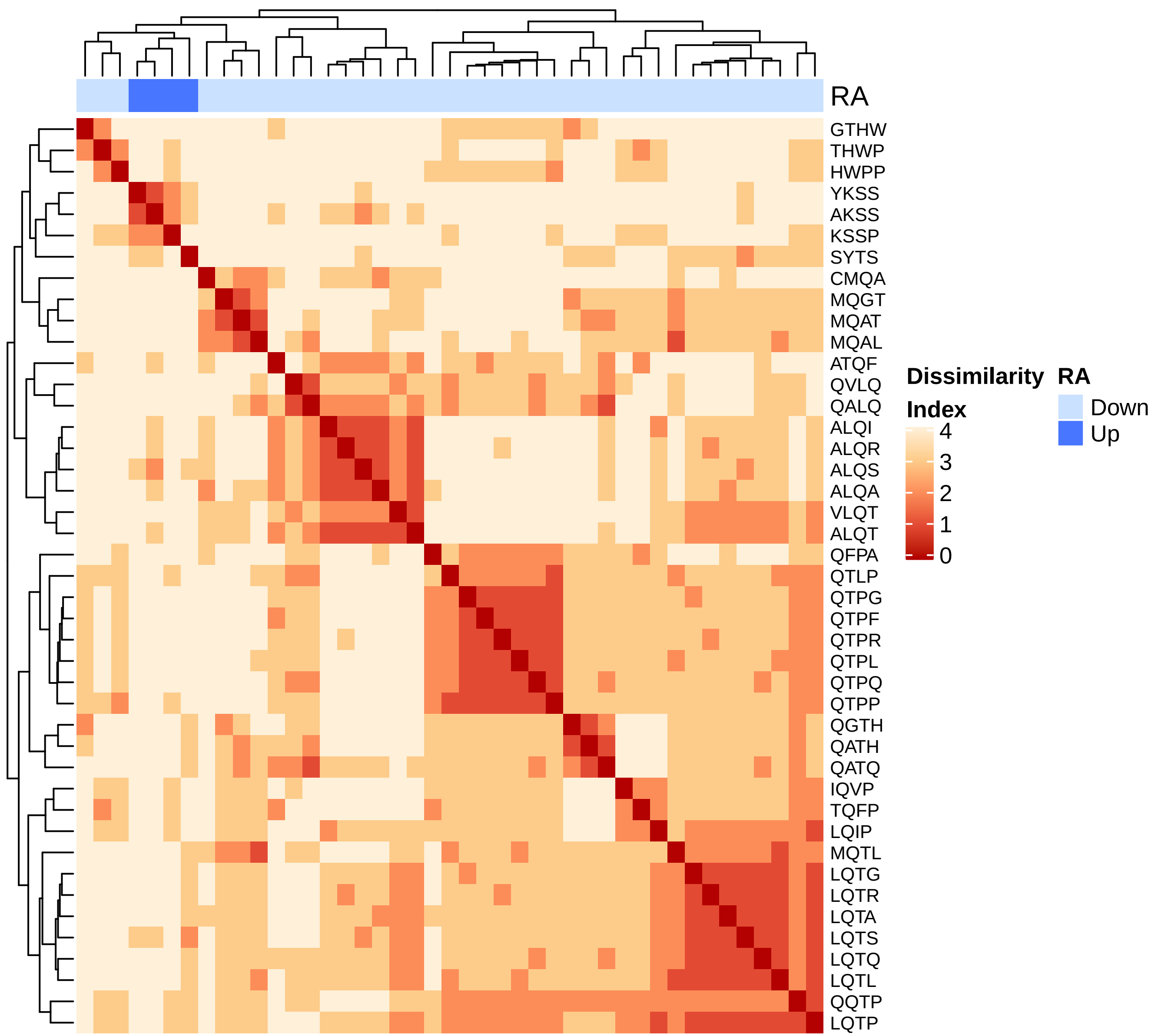

Kmers from IGL significant in Cont model

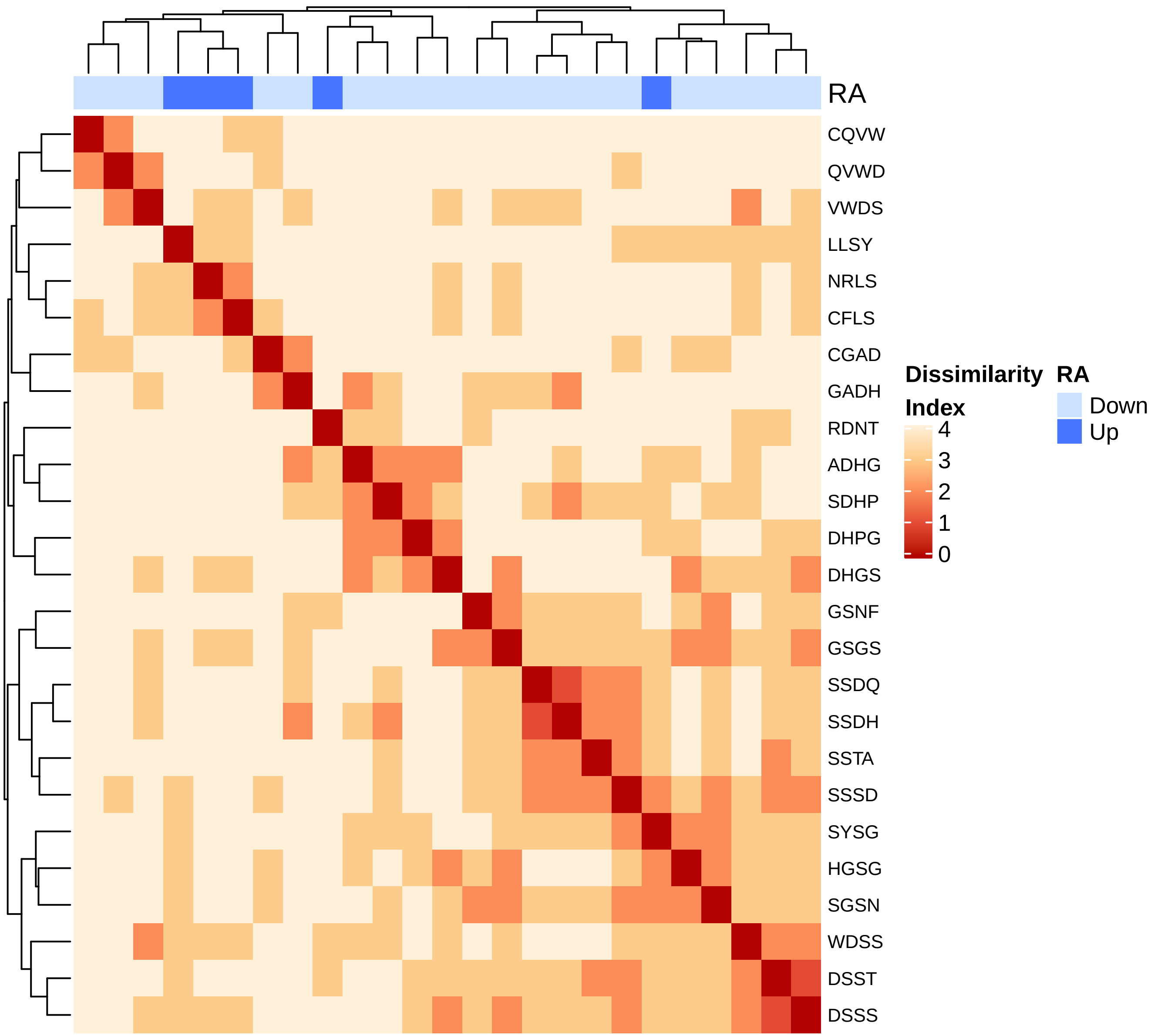

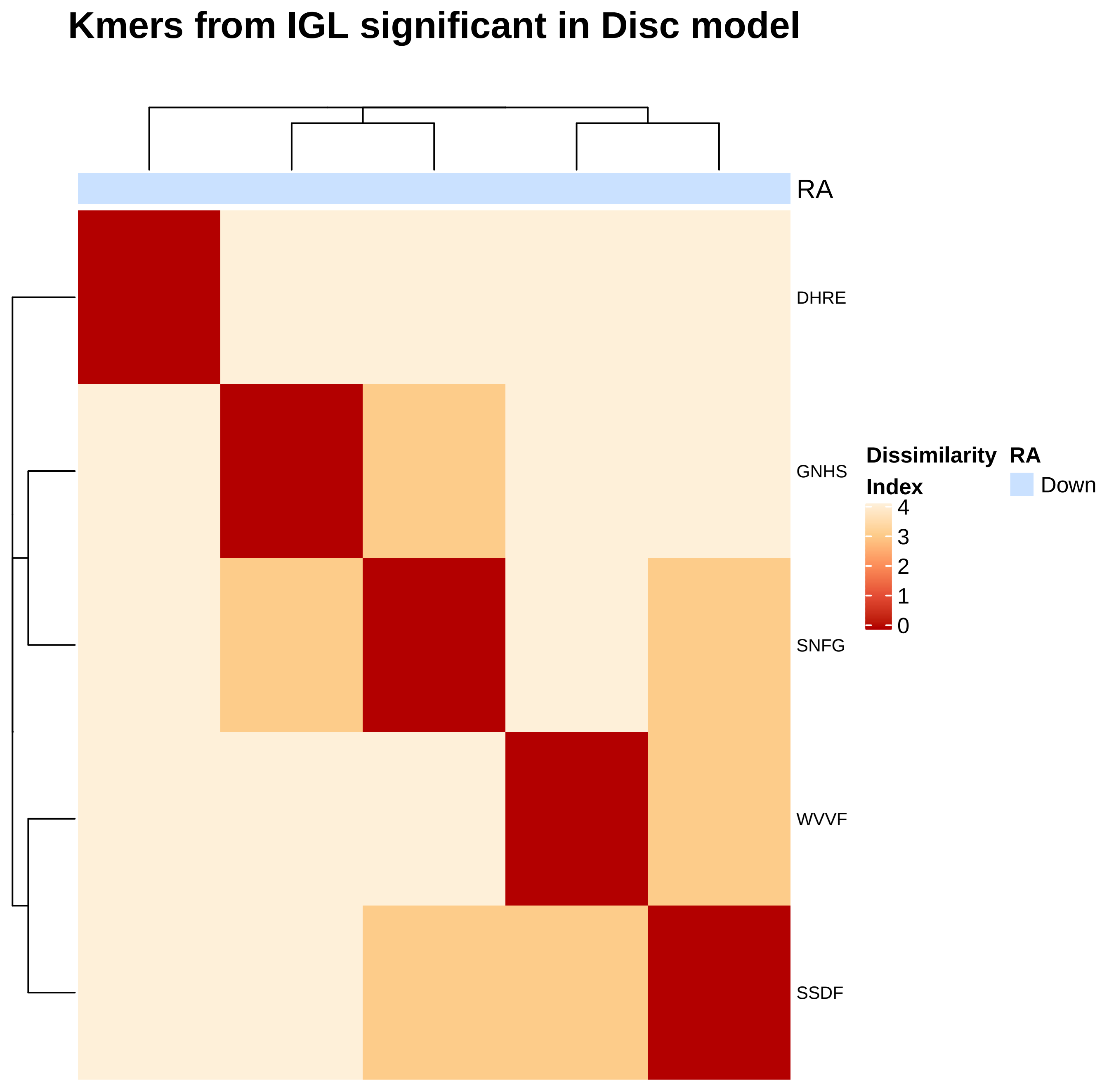
Kmers from IGL significant in Disc model
RA
DHRE
GNHS
SNFG
WVVF
SSDF
Dissimilarity
Index
4
3
2
1
0
RA
Down

Kmers from IGL significant in Hurdle model

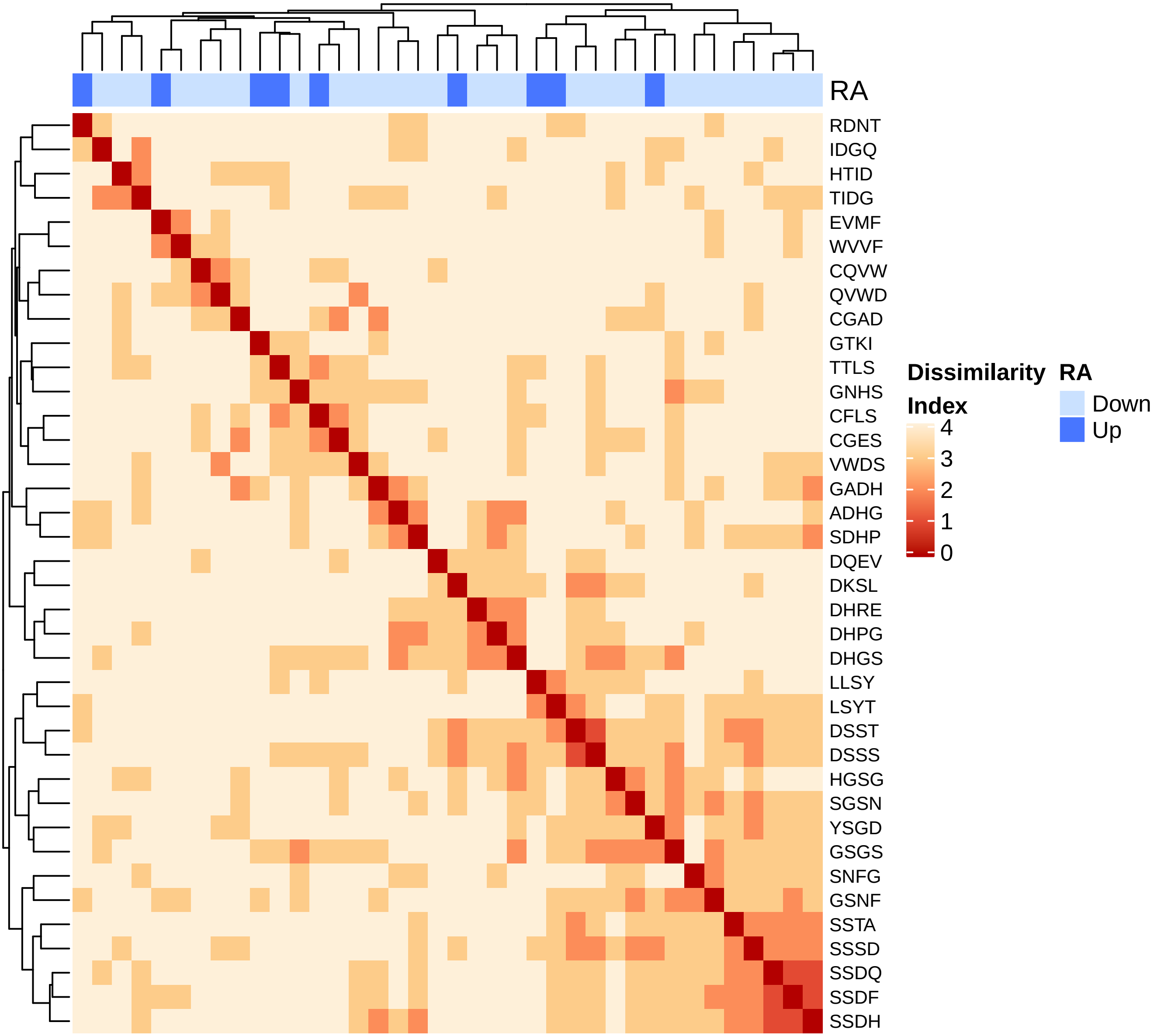

Kmers from TRA significant in Cont model

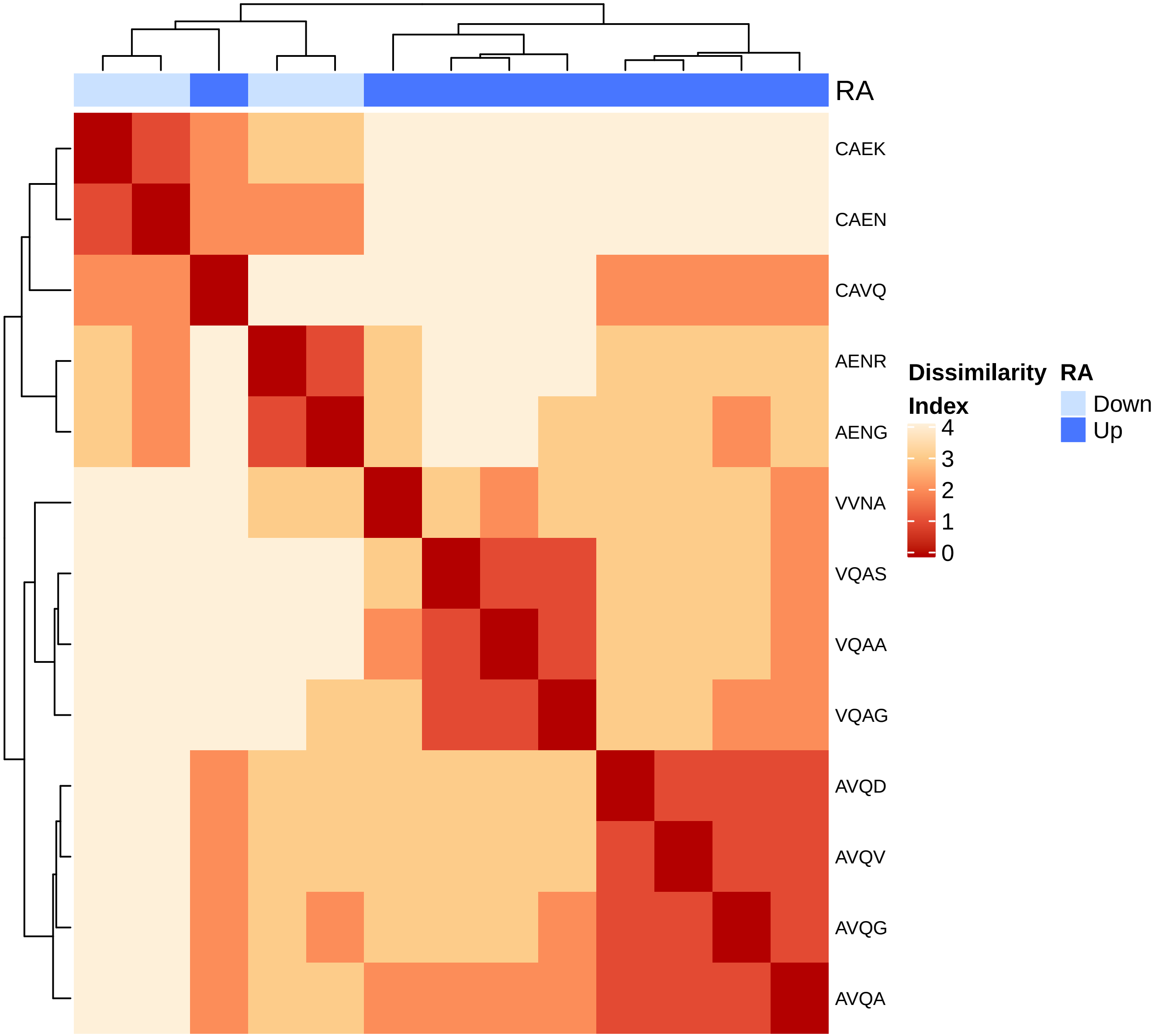

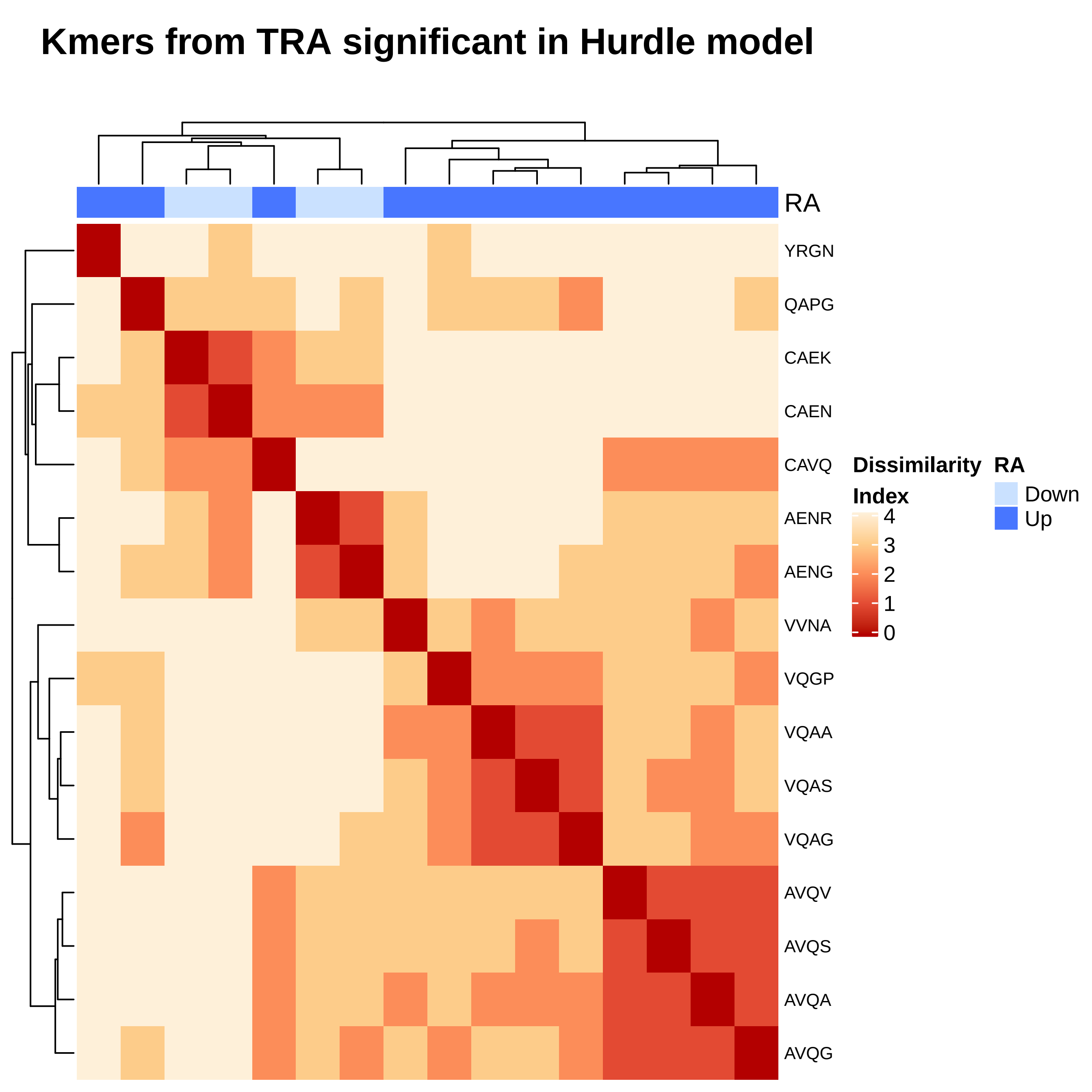
Kmers from TRA significant in Hurdle model
RA
YRGN
QAPG
CAEK
CAEN
CAVQ
AENR
AENG
VVNA
VQGP
VQAA
VQAS
VQAG
AVQV
AVQS
AVQA
AVQG
Dissimilarity Index
4
3
2
1
0
RA
Down
Up

Kmers from TRB significant in Cont model

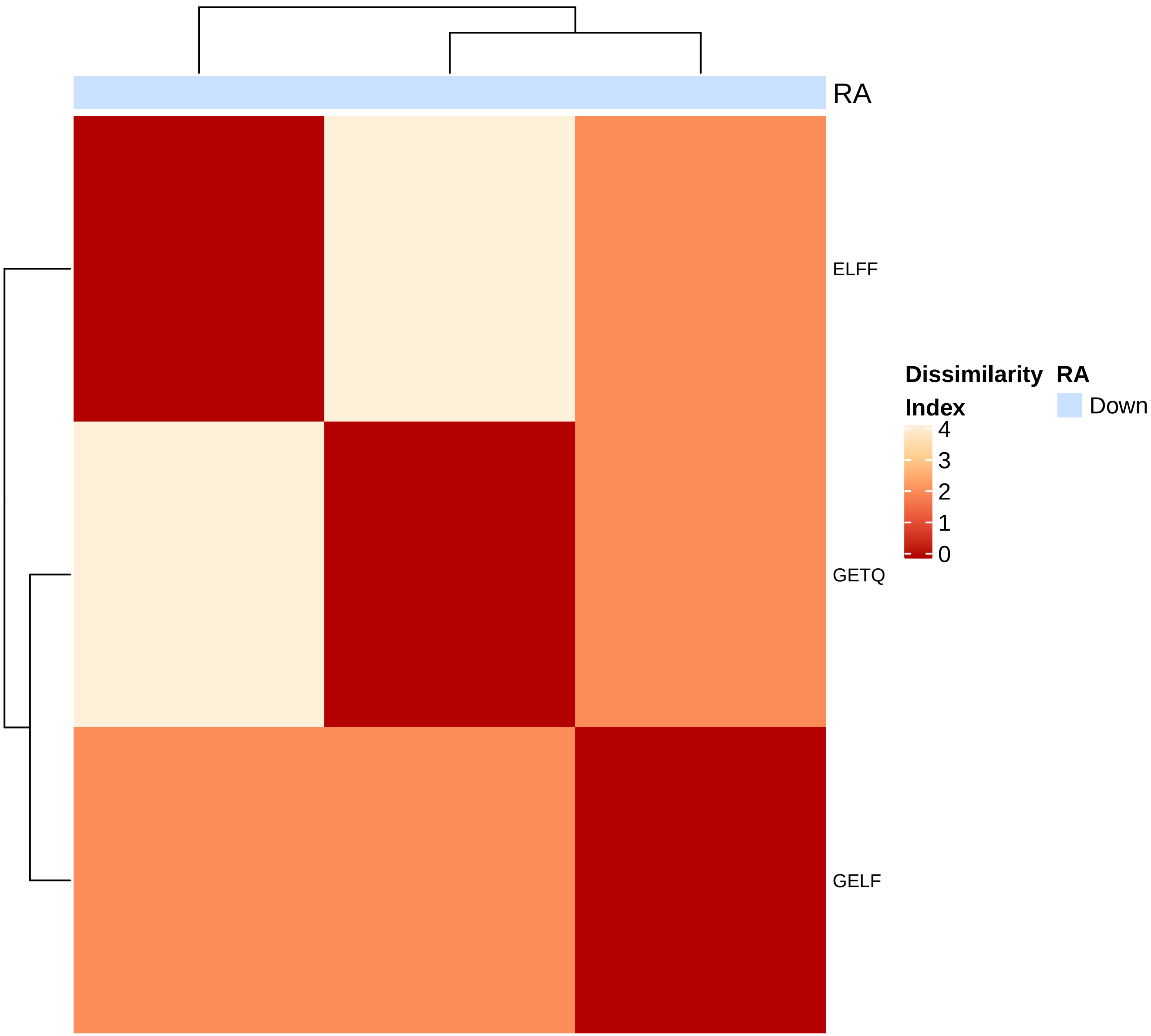

Supplement: Supplementary file 27 — Additional file 27: Figure S6. Pairwise dissimilarity matrix among the amino acid sequences of TRA/TRB/IGL/IGK k-mers associated with rheumatoid arthritis. K-mers over- and under-represented in rheumatoid arthritis are represented in dark and light blue, respectively. The dissimilarity index was computed using the Levenshtein distance measure so that the higher the Levenshtein distance, the higher the dissimilarity index between two k-mer sequences. Abbreviations: Cont, continuous model; Disc, discrete model; RA, rheumatoid arthritis. [file 13059_2024_3210_MOESM27_ESM.pdf]
