# Supplementary material for: Seven-chain adaptive immune receptor repertoire analysis in rheumatoid arthritis reveals novel features associated with disease and clinically relevant phenotypes
Source: Genome Biol. 2024 Mar 11;25:68. doi: 10.1186/s13059-024-03210-0 (PMC10926600; doi:10.1186/s13059-024-03210-0)

Fig S7. Graphical representation of the association between the length of the CDR3 amino acid sequences and rheumatoid arthritis. The results of the case-control, case-case (i.e., association analysis with clinical phenotypes in rheumatoid arthritis) and longitudinal analysis (i.e., baseline vs. week 12 and baseline vs. week 12 stratified by clinical response) are provided at the chain level. Shifts in the clone length distribution and the statistical significance of the difference in the abundance of clones with a particular length of the CDR3 amino acid sequence between the two indicated conditions are shown on the left side. This plot also shows the summary statistics detected for each condition. In the middle, the empirical cumulative density distribution of the abundance of CDR3 amino acid sequences is shown separately for each condition. On the right side, the empirical quantile-quantile plot computed for the two indicated conditions is shown. Abbreviations: P, p-value of the Wilcoxon test; *, p-value<0.05 in the Fisher test; **, p-value<0.005 in the Fisher test.

CASE−CONTROL ANALYSIS

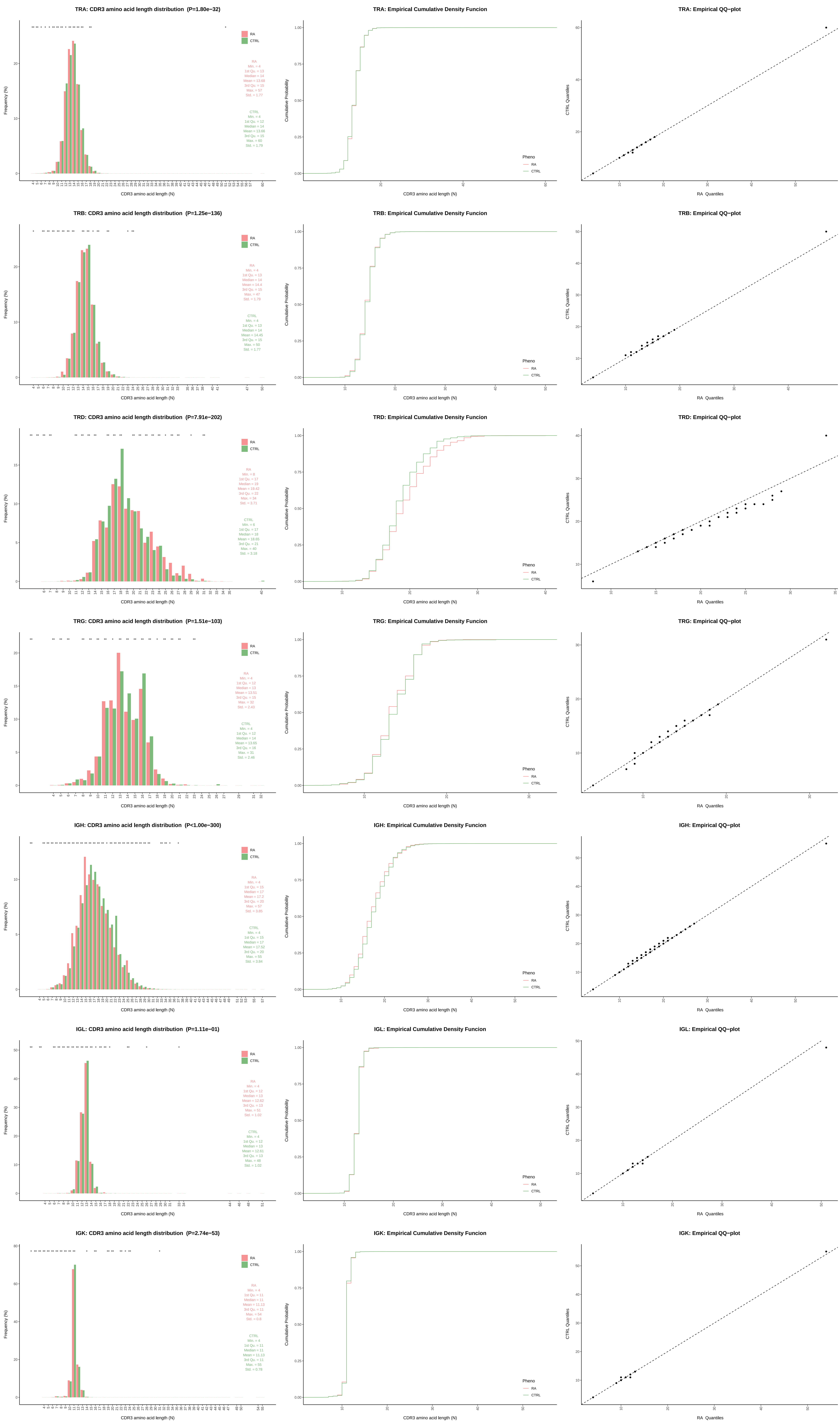

CASE−CASE ANALYSIS: RESPONSE TO TNFi THERAPY

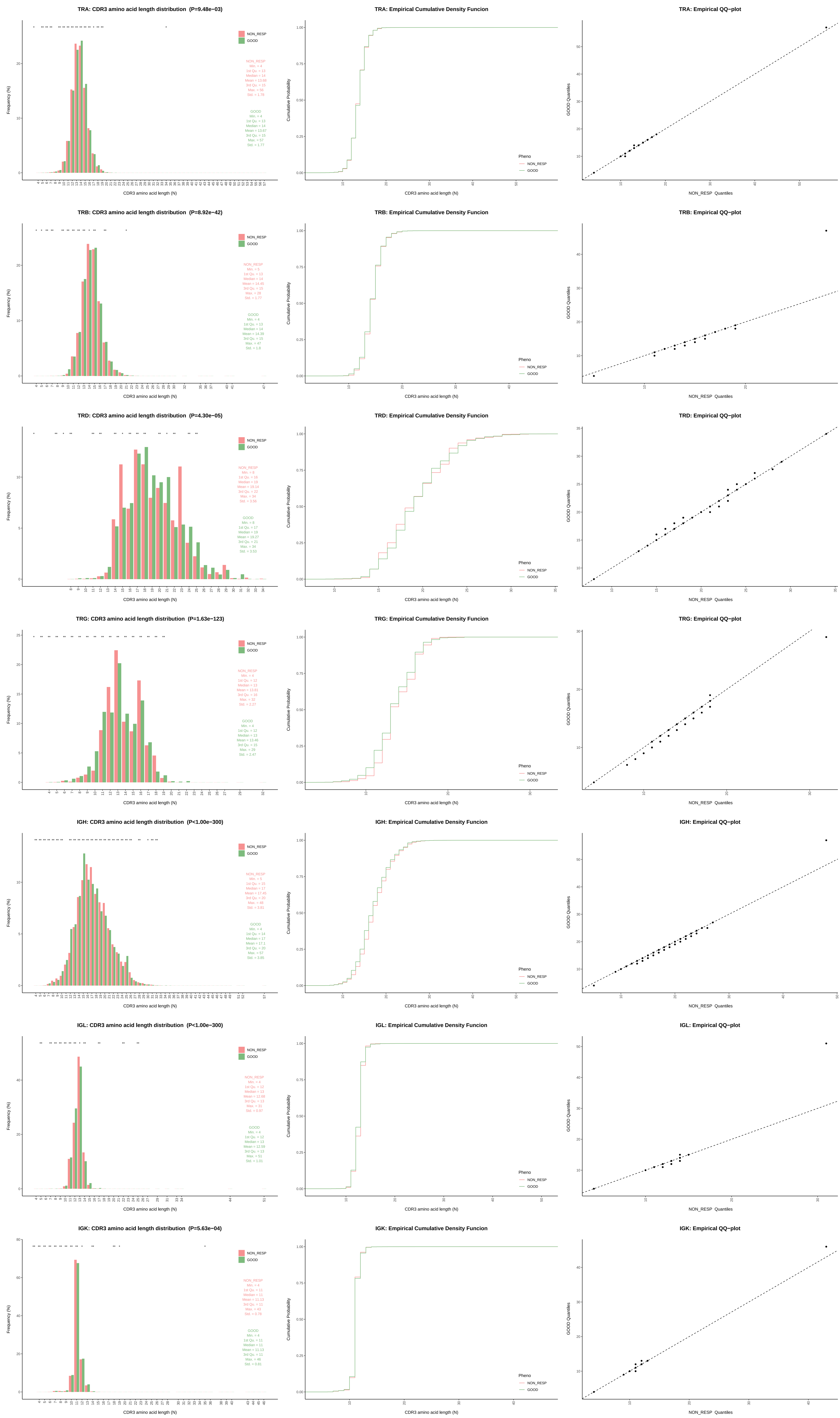

CASE−CASE ANALYSIS: DISEASE ACTIVITY

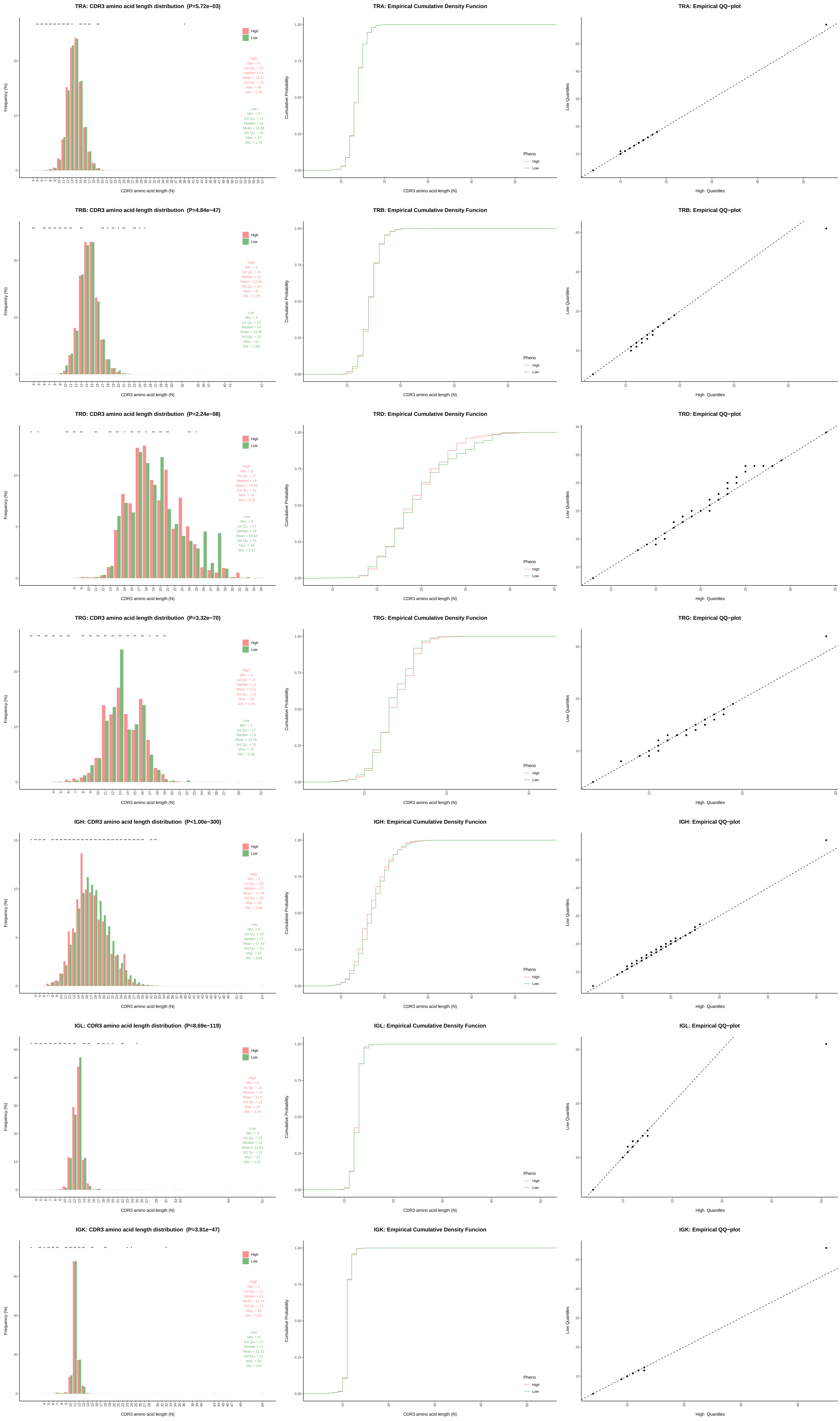

CASE−CASE ANALYSIS: ACPA PHENOTYPE

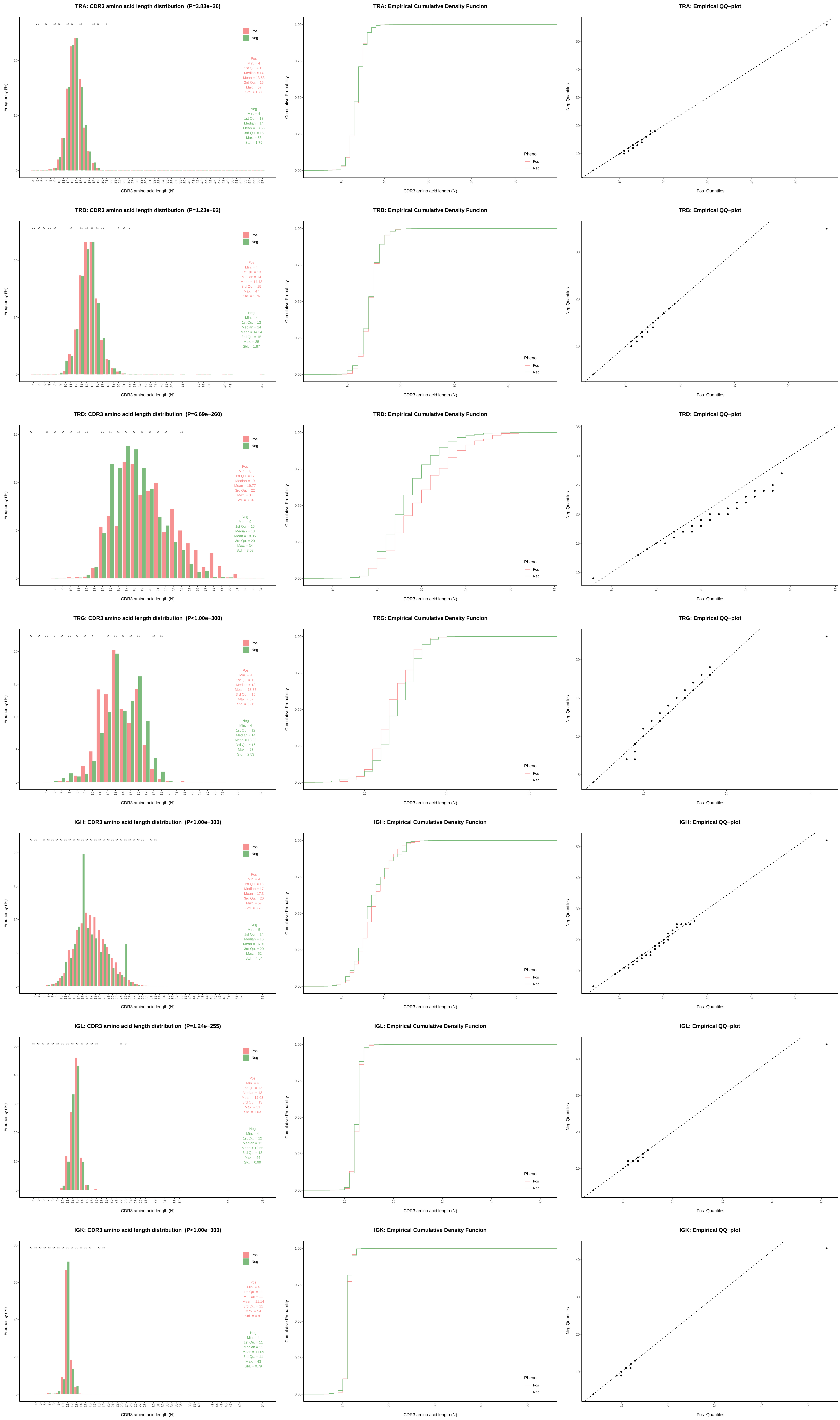

CASE−CASE ANALYSIS: RF PHENOTYPE

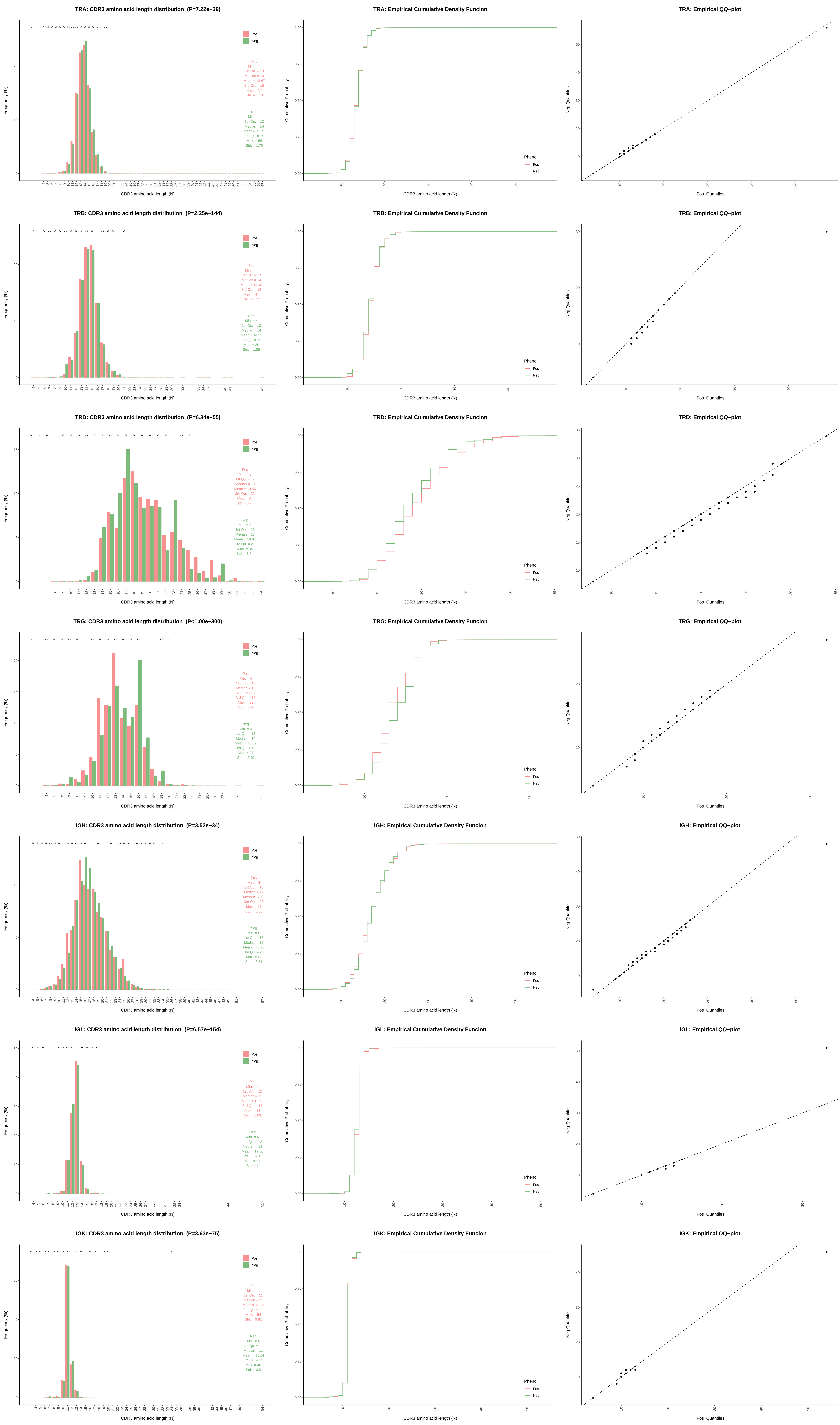

LONGITUDINAL ANALYSIS: BASELINE vs. TNFi THERAPY

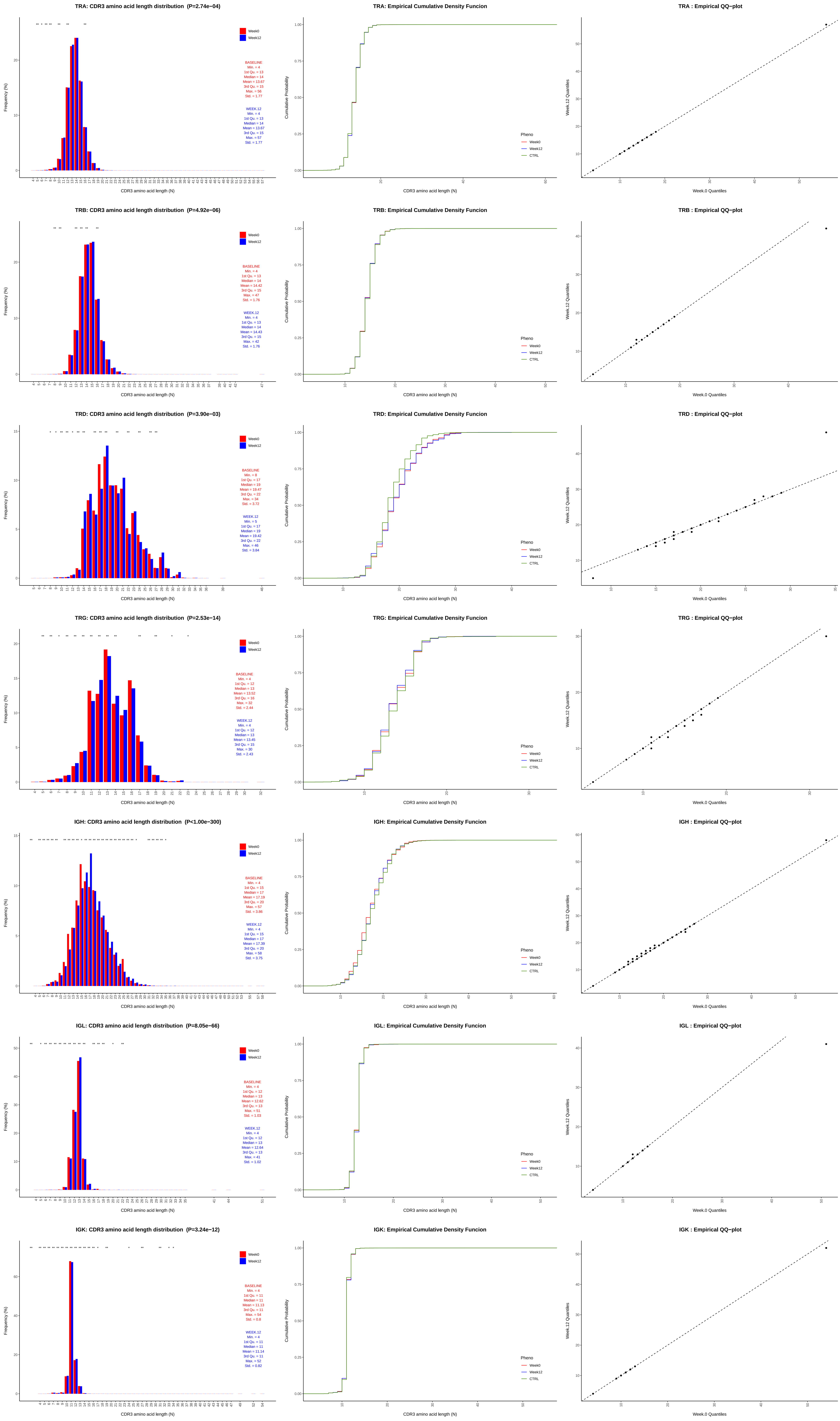

LONGITUDINAL ANALYSIS STRATIFIED BY CLINICAL RESPONSE TO TNFi THERAPY

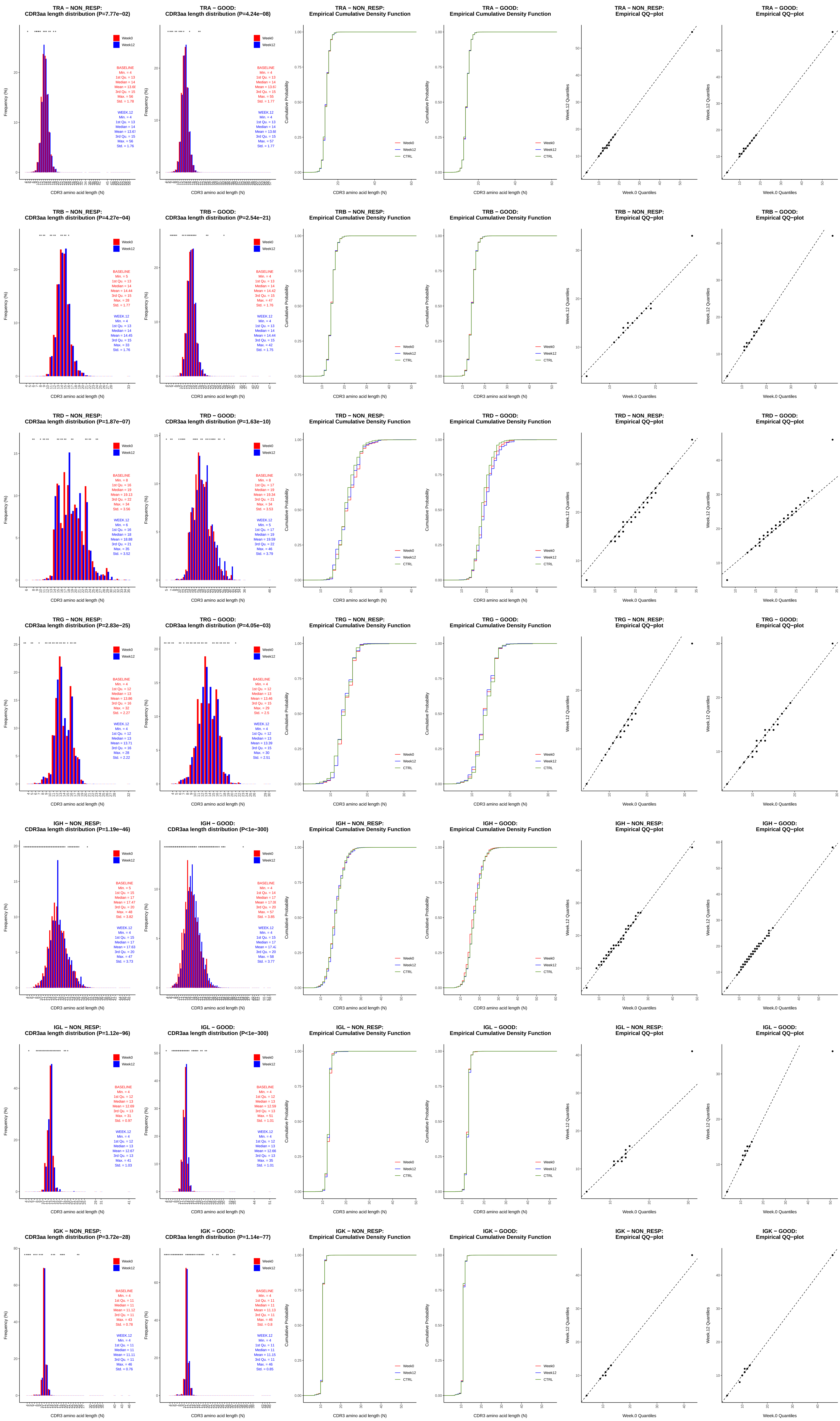

Supplement: Supplementary file 32 — Additional file 32: Figure S7. Graphical representation of the association between the length of the CDR3 amino acid sequences and rheumatoid arthritis. The results of the case-control, case-case (i.e., association analysis with clinical phenotypes in rheumatoid arthritis) and longitudinal analysis (i.e., baseline vs. week 12 and baseline vs. week 12 stratified by clinical response) are provided at the chain level. Shifts in the clone length distribution and the statistical significance of the difference in the abundance of clones with a particular length of the CDR3 amino acid sequence between the two indicated conditions are shown on the left side. This plot also shows the summary statistics detected for each condition. In the middle, the empirical cumulative density distribution of the abundance of CDR3 amino acid sequences is shown separately for each condition. On the right side, the empirical quantile-quantile plot computed for the two indicated conditions is shown. Abbreviations: P, p-value of the Wilcoxon test;*, p-value<0.05 in the Fisher test; **, p-value<0.005 in the Fisher test. [file 13059_2024_3210_MOESM32_ESM.pdf]
