# Supplementary material for: Seven-chain adaptive immune receptor repertoire analysis in rheumatoid arthritis reveals novel features associated with disease and clinically relevant phenotypes
Source: Genome Biol. 2024 Mar 11;25:68. doi: 10.1186/s13059-024-03210-0 (PMC10926600; doi:10.1186/s13059-024-03210-0)
Supplement: Supplementary file 36 — Additional file 36: Figure S8. Clonality profile of the samples included in the present study. Density distribution resulting from the analysis of the clone frequency at the sample and chain levels. [file 13059_2024_3210_MOESM36_ESM.pdf]

Fig S8. Clonality profile of the samples included in the present study. Density distribution resulting from the analysis of the clone frequency at the sample and chain levels.

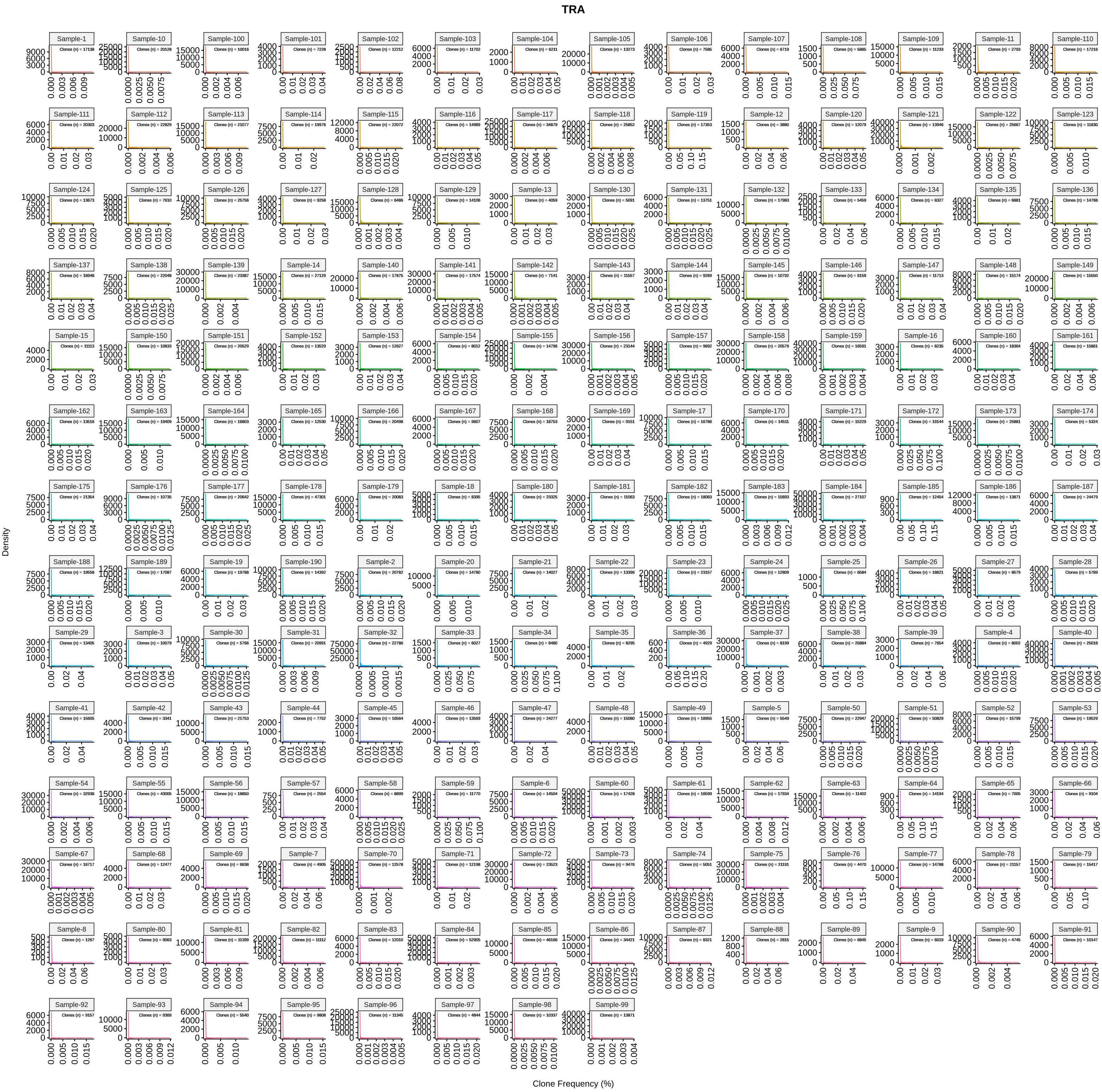
TRA
Sample-1
Sample-10
Sample-100
Sample-101
Sample-102
Sample-103
Sample-104
Sample-105
Sample-106
Sample-107
Sample-108
Sample-109
Sample-11
Sample-110
Sample-111
Sample-112
Sample-113
Sample-114
Sample-115
Sample-116
Sample-117
Sample-118
Sample-119
Sample-12
Sample-120
Sample-121
Sample-122
Sample-123
Sample-124
Sample-125
Sample-126
Sample-127
Sample-128
Sample-129
Sample-13
Sample-130
Sample-131
Sample-132
Sample-133
Sample-134
Sample-135
Sample-136
Sample-137
Sample-138
Sample-139
Sample-14
Sample-140
Sample-141
Sample-142
Sample-143
Sample-144
Sample-145
Sample-146
Sample-147
Sample-148
Sample-149
Sample-15
Sample-150
Sample-151
Sample-152
Sample-153
Sample-154
Sample-155
Sample-156
Sample-157
Sample-158
Sample-159
Sample-16
Sample-160
Sample-161
Sample-162
Sample-163
Sample-164
Sample-165
Sample-166
Sample-167
Sample-168
Sample-169
Sample-17
Sample-170
Sample-171
Sample-172
Sample-173
Sample-174
Sample-175
Sample-176
Sample-177
Sample-178
Sample-179
Sample-18
Sample-180
Sample-181
Sample-182
Sample-183
Sample-184
Sample-185
Sample-186
Sample-187
Sample-188
Sample-189
Sample-19
Sample-190
Sample-2
Sample-20
Sample-21
Sample-22
Sample-23
Sample-24
Sample-25
Sample-26
Sample-27
Sample-28
Sample-29
Sample-3
Sample-30
Sample-31
Sample-32
Sample-33
Sample-34
Sample-35
Sample-36
Sample-37
Sample-38
Sample-39
Sample-4
Sample-40
Sample-41
Sample-42
Sample-43
Sample-44
Sample-45
Sample-46
Sample-47
Sample-48
Sample-49
Sample-5
Sample-50
Sample-51
Sample-52
Sample-53
Sample-54
Sample-55
Sample-56
Sample-57
Sample-58
Sample-59
Sample-6
Sample-60
Sample-61
Sample-62
Sample-63
Sample-64
Sample-65
Sample-66
Sample-67
Sample-68
Sample-69
Sample-7
Sample-70
Sample-71
Sample-72
Sample-73
Sample-74
Sample-75
Sample-76
Sample-77
Sample-78
Sample-79
Sample-8
Sample-80
Sample-81
Sample-82
Sample-83
Sample-84
Sample-85
Sample-86
Sample-87
Sample-88
Sample-89
Sample-9
Sample-90
Sample-91
Sample-92
Sample-93
Sample-94
Sample-95
Sample-96
Sample-97
Sample-98
Sample-99
Density
Clone Frequency (%)

TRB

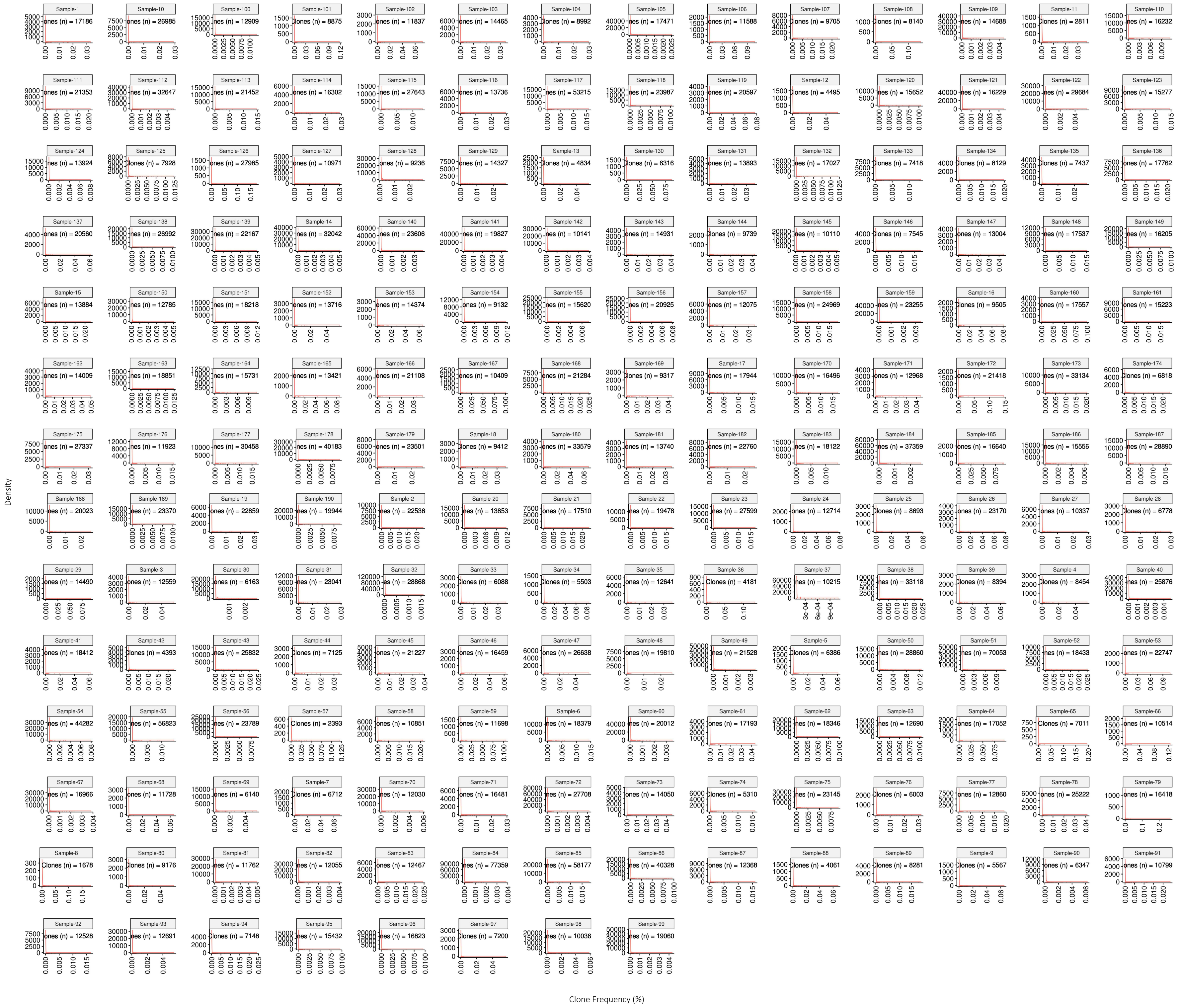
Density
Clone Frequency (%)
Sample-1 ones (n) = 17186
Sample-10 ones (n) = 26985
Sample-100 ones (n) = 12909
Sample-101 Clones (n) = 8875
Sample-102 ones (n) = 11837
Sample-103 ones (n) = 14465
Sample-104 Clones (n) = 8992
Sample-105 nes (n) = 17471
Sample-106 ones (n) = 11588
Sample-107 ones (n) = 9705
Sample-108 Clones (n) = 8140
Sample-109 ones (n) = 14688
Sample-11 Clones (n) = 2811
Sample-110 nes (n) = 16232
Sample-111 ones (n) = 21353
Sample-112 ones (n) = 32647
Sample-113 ones (n) = 21452
Sample-114 ones (n) = 16302
Sample-115 ones (n) = 27643
Sample-116 ones (n) = 13736
Sample-117 nes (n) = 53215
Sample-118 nes (n) = 23987
Sample-119 ones (n) = 20597
Sample-12 Clones (n) = 4495
Sample-120 ones (n) = 15652
Sample-121 ones (n) = 16229
Sample-122 ones (n) = 29684
Sample-123 ones (n) = 15277
Sample-124 nes (n) = 13924
Sample-125 Clones (n) = 7928
Sample-126 ones (n) = 27985
Sample-127 ones (n) = 10971
Sample-128 ones (n) = 9236
Sample-129 ones (n) = 14327
Sample-13 Clones (n) = 4834
Sample-130 Clones (n) = 6316
Sample-131 ones (n) = 13893
Sample-132 ones (n) = 17027
Sample-133 Clones (n) = 7418
Sample-134 Clones (n) = 8129
Sample-135 Clones (n) = 7437
Sample-136 ones (n) = 17762
Sample-137 ones (n) = 20560
Sample-138 nes (n) = 26992
Sample-139 ones (n) = 22167
Sample-14 nes (n) = 32042
Sample-140 nes (n) = 23606
Sample-141 ones (n) = 19827
Sample-142 ones (n) = 10141
Sample-143 ones (n) = 14931
Sample-144 Clones (n) = 9739
Sample-145 ones (n) = 10110
Sample-146 Clones (n) = 7545
Sample-147 ones (n) = 13004
Sample-148 ones (n) = 17537
Sample-149 nes (n) = 16205
Sample-15 ones (n) = 13884
Sample-150 ones (n) = 12785
Sample-151 ones (n) = 18218
Sample-152 ones (n) = 13716
Sample-153 ones (n) = 14374
Sample-154 ones (n) = 9132
Sample-155 nes (n) = 15620
Sample-156 nes (n) = 20925
Sample-157 ones (n) = 12075
Sample-158 nes (n) = 24969
Sample-159 nes (n) = 23255
Sample-16 Clones (n) = 9505
Sample-160 ones (n) = 17557
Sample-161 ones (n) = 15223
Sample-162 ones (n) = 14009
Sample-163 nes (n) = 18851
Sample-164 nes (n) = 15731
Sample-165 ones (n) = 13421
Sample-166 ones (n) = 21108
Sample-167 ones (n) = 10409
Sample-168 ones (n) = 21284
Sample-169 Clones (n) = 9317
Sample-17 ones (n) = 17944
Sample-170 ones (n) = 16496
Sample-171 ones (n) = 12968
Sample-172 ones (n) = 21418
Sample-173 ones (n) = 33134
Sample-174 Clones (n) = 6818
Sample-175 ones (n) = 27337
Sample-176 nes (n) = 11923
Sample-177 ones (n) = 30458
Sample-178 nes (n) = 40183
Sample-179 ones (n) = 23501
Sample-18 Clones (n) = 9412
Sample-180 ones (n) = 33579
Sample-181 ones (n) = 13740
Sample-182 ones (n) = 22760
Sample-183 ones (n) = 18122
Sample-184 ones (n) = 37359
Sample-185 ones (n) = 16640
Sample-186 nes (n) = 15556
Sample-187 nes (n) = 28890
Sample-188 nes (n) = 20023
Sample-189 nes (n) = 23370
Sample-19 ones (n) = 22859
Sample-190 nes (n) = 19944
Sample-2 nes (n) = 22536
Sample-20 nes (n) = 13853
Sample-21 ones (n) = 17510
Sample-22 nes (n) = 19478
Sample-23 nes (n) = 27599
Sample-24 ones (n) = 12714
Sample-25 Clones (n) = 8693
Sample-26 ones (n) = 23170
Sample-27 ones (n) = 10337
Sample-28 Clones (n) = 6778
Sample-29 Clones (n) = 14490
Sample-3 ones (n) = 12559
Sample-30 Clones (n) = 6163
Sample-31 nes (n) = 23041
Sample-32 es (n) = 28868
Sample-33 Clones (n) = 6088
Sample-34 Clones (n) = 5503
Sample-35 ones (n) = 12641
Sample-36 Clones (n) = 4181
Sample-37 nes (n) = 10215
Sample-38 ones (n) = 33118
Sample-39 Clones (n) = 8394
Sample-4 Clones (n) = 8454
Sample-40 nes (n) = 25876
Sample-41 ones (n) = 18412
Sample-42 Clones (n) = 4393
Sample-43 nes (n) = 25832
Sample-44 Clones (n) = 7125
Sample-45 ones (n) = 21227
Sample-46 ones (n) = 16459
Sample-47 ones (n) = 26638
Sample-48 ones (n) = 19810
Sample-49 nes (n) = 21528
Sample-5 Clones (n) = 6386
Sample-50 nes (n) = 28860
Sample-51 nes (n) = 70053
Sample-52 nes (n) = 18433
Sample-53 ones (n) = 22747
Sample-54 nes (n) = 44282
Sample-55 nes (n) = 56823
Sample-56 nes (n) = 23789
Sample-57 Clones (n) = 2393
Sample-58 ones (n) = 10851
Sample-59 ones (n) = 11698
Sample-6 nes (n) = 18379
Sample-60 nes (n) = 20012
Sample-61 ones (n) = 17193
Sample-62 nes (n) = 18346
Sample-63 nes (n) = 12690
Sample-64 ones (n) = 17052
Sample-65 Clones (n) = 7011
Sample-66 ones (n) = 10514
Sample-67 nes (n) = 16966
Sample-68 ones (n) = 11728
Sample-69 ones (n) = 6140
Sample-7 Clones (n) = 6712
Sample-70 ones (n) = 12030
Sample-71 ones (n) = 16481
Sample-72 nes (n) = 27708
Sample-73 ones (n) = 14050
Sample-74 Clones (n) = 5310
Sample-75 nes (n) = 23145
Sample-76 Clones (n) = 6003
Sample-77 ones (n) = 12860
Sample-78 ones (n) = 25222
Sample-79 ones (n) = 16418
Sample-8 Clones (n) = 1678
Sample-80 Clones (n) = 9176
Sample-81 ones (n) = 11762
Sample-82 ones (n) = 12055
Sample-83 ones (n) = 12467
Sample-84 nes (n) = 77359
Sample-85 nes (n) = 58177
Sample-86 nes (n) = 40328
Sample-87 Clones (n) = 12368
Sample-88 Clones (n) = 4061
Sample-89 Clones (n) = 8281
Sample-9 Clones (n) = 5567
Sample-90 Clones (n) = 6347
Sample-91 ones (n) = 10799
Sample-92 ones (n) = 12528
Sample-93 nes (n) = 12691
Sample-94 ones (n) = 7148
Sample-95 nes (n) = 15432
Sample-96 nes (n) = 16823
Sample-97 Clones (n) = 7200
Sample-98 nes (n) = 10036
Sample-99 nes (n) = 19060

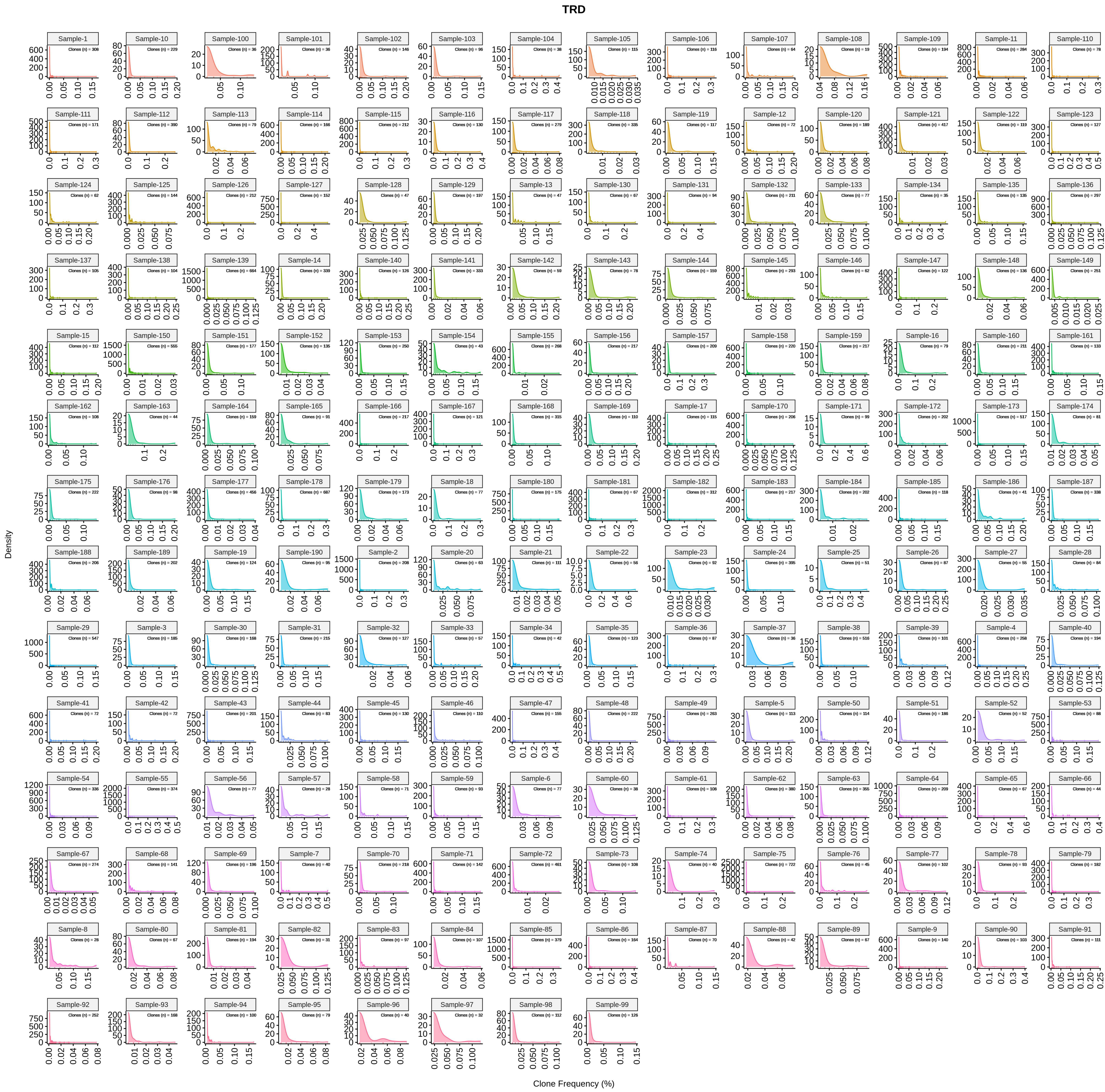
TRD
Density
Clone Frequency (%)

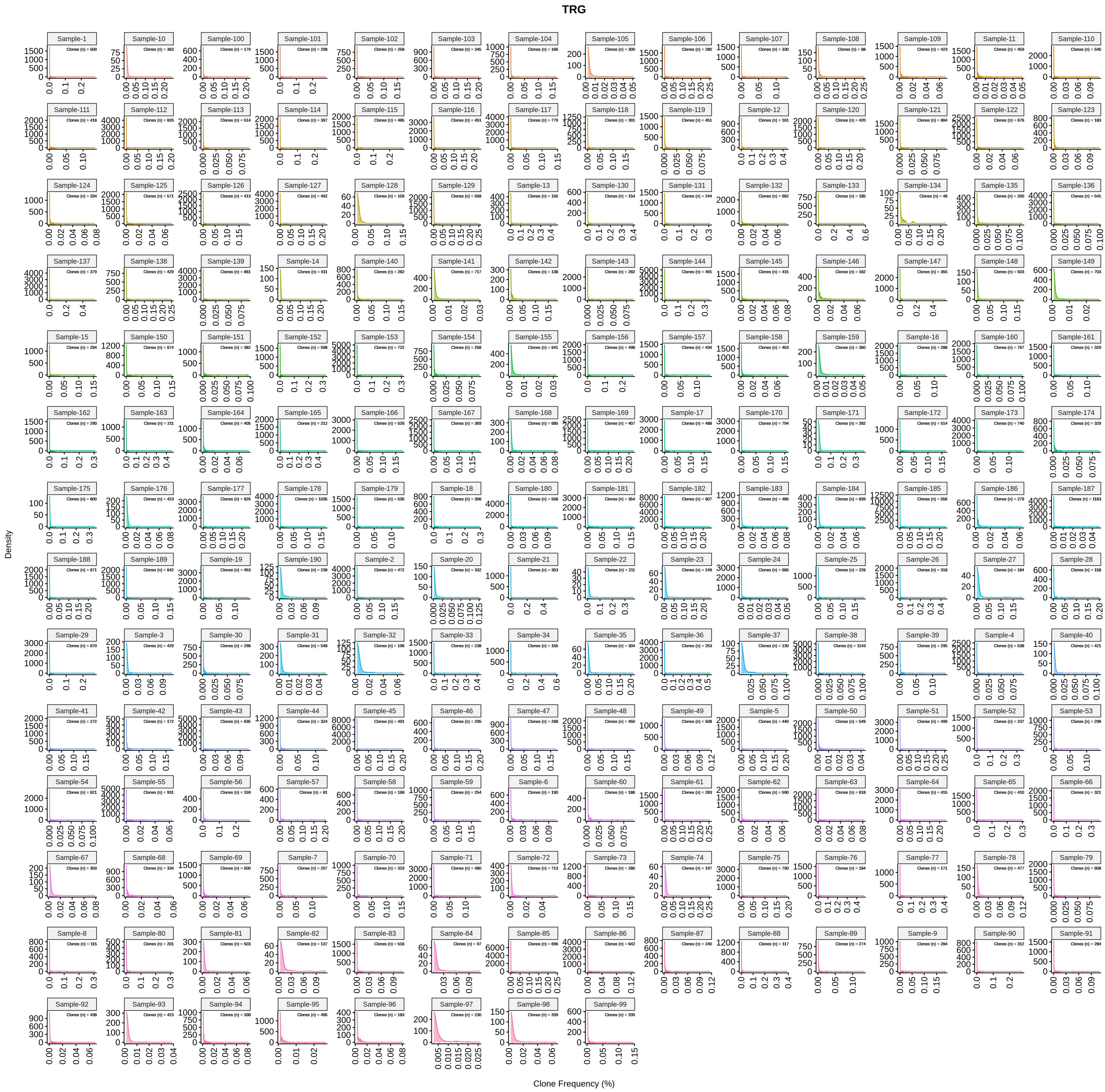
TRG
Density
Clone Frequency (%)

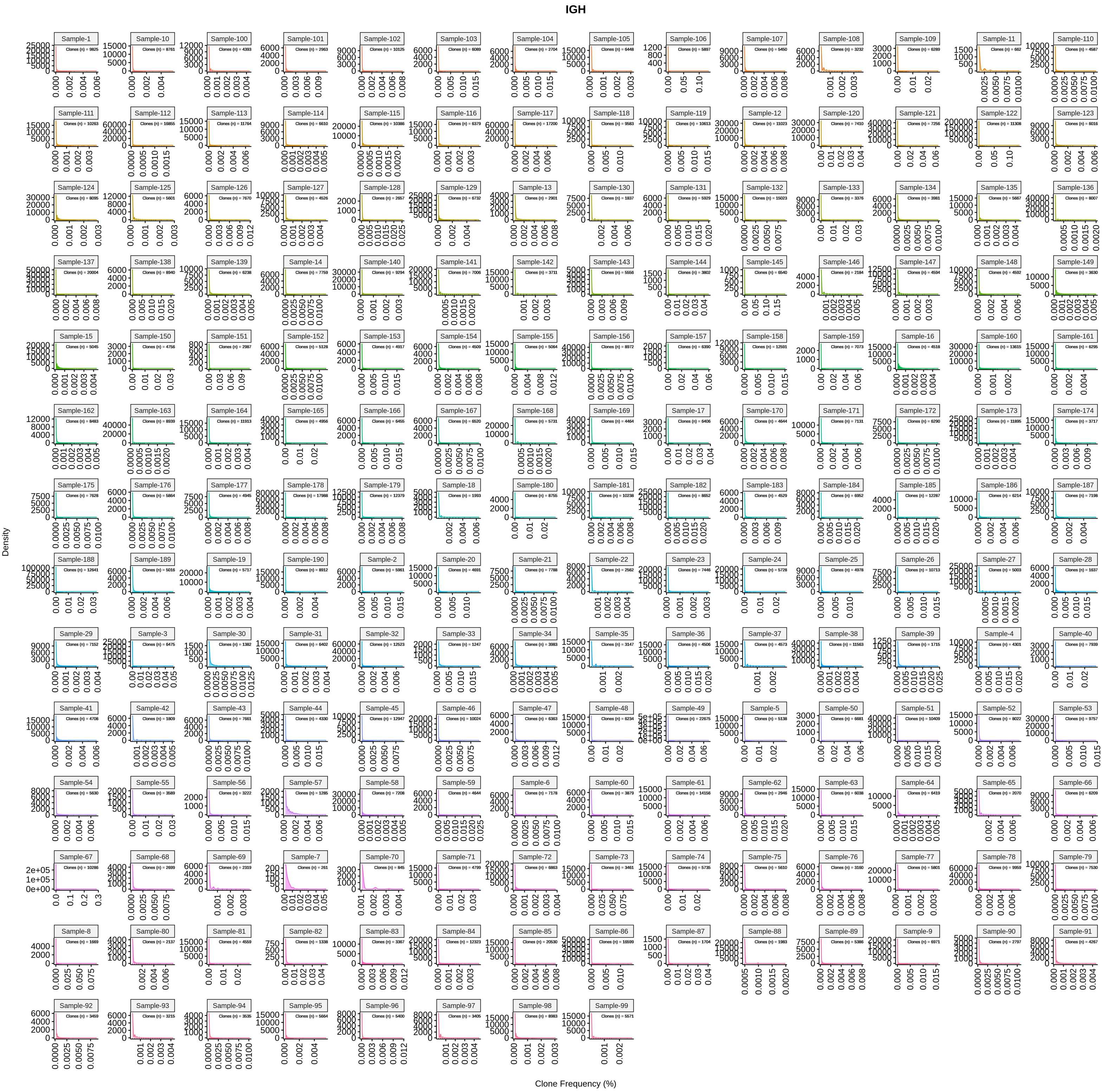
IGH
Density
Clone Frequency (%)

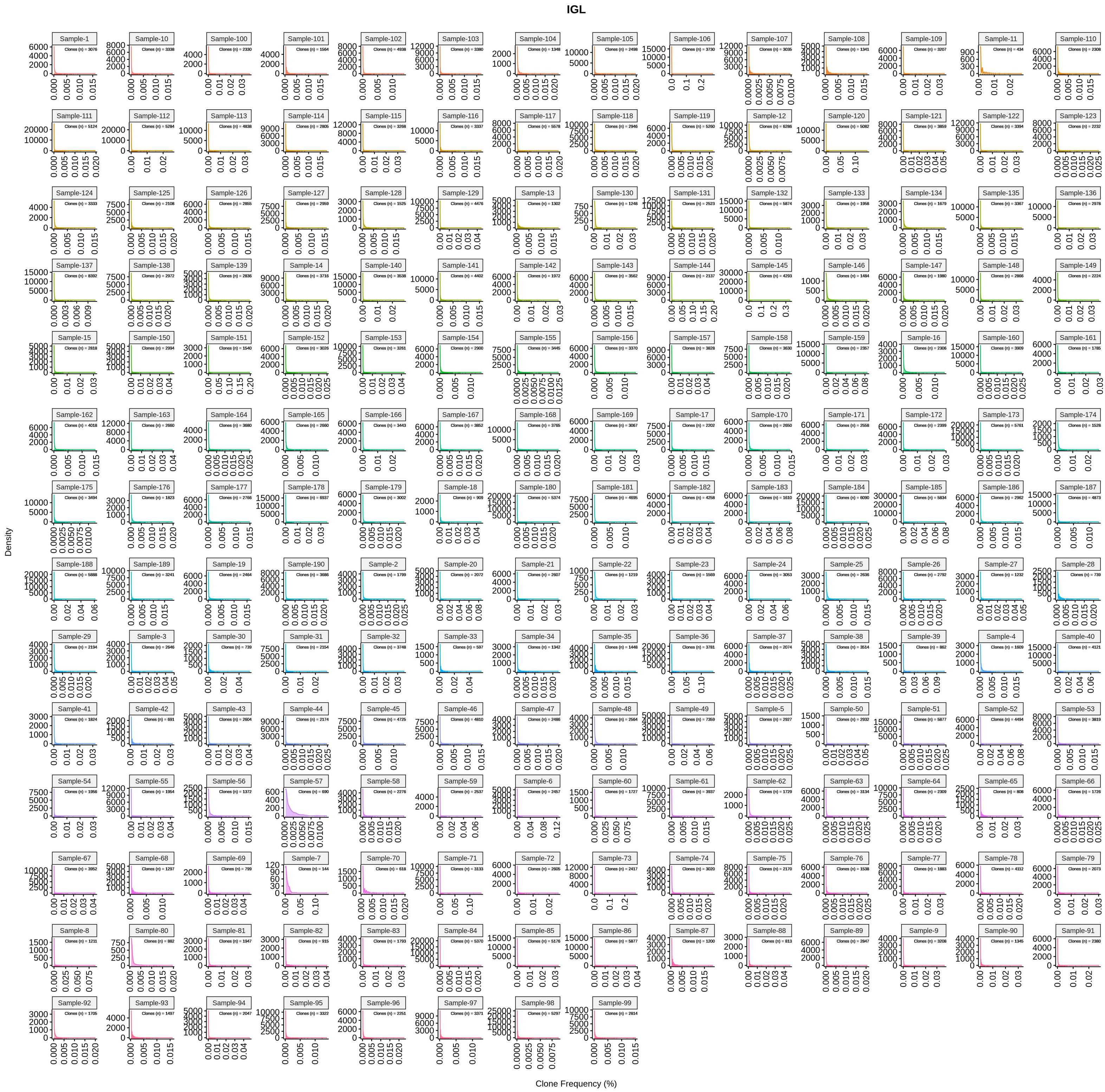
IGL
Density
Clone Frequency (%)

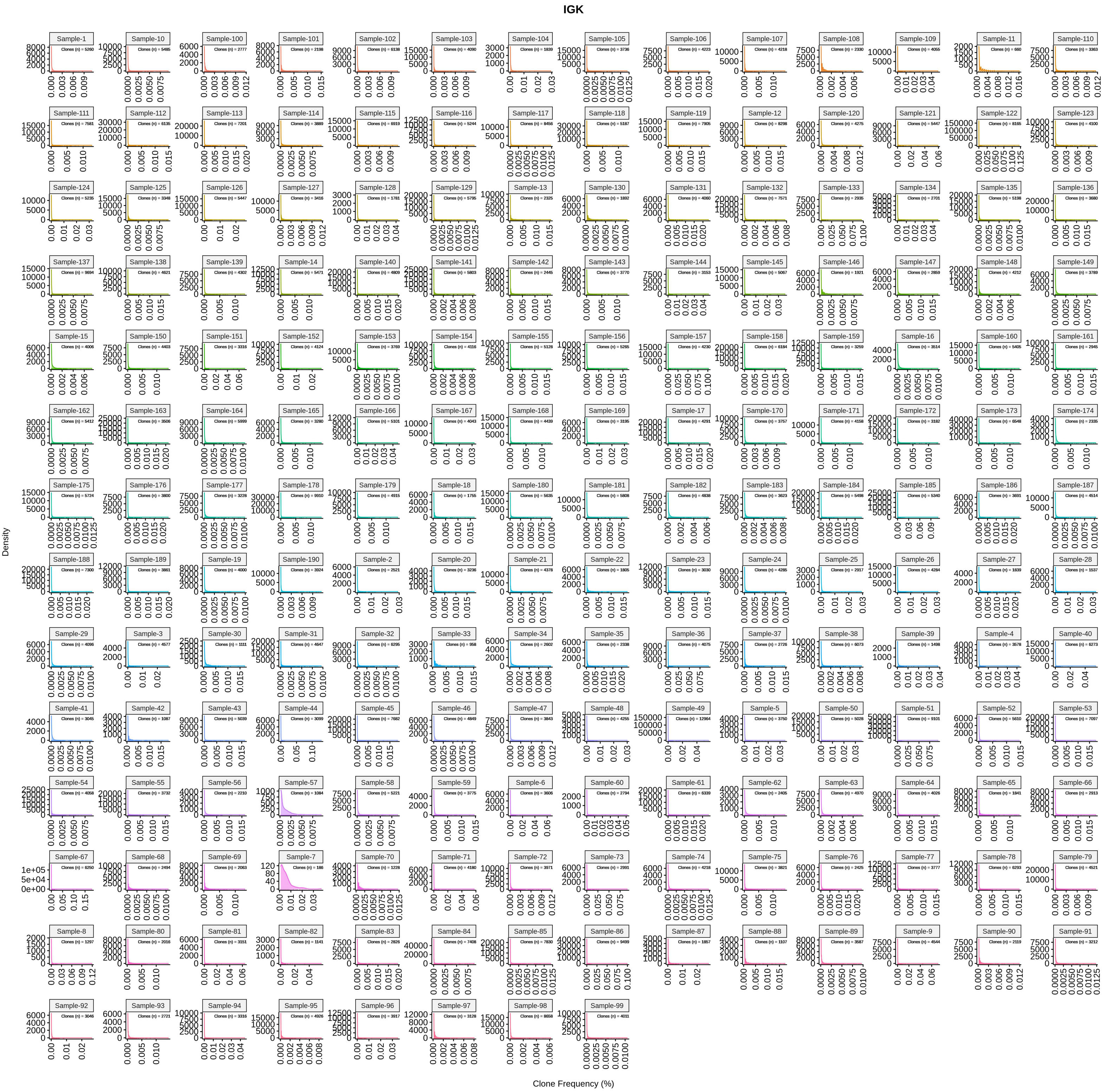
IGK
Density
Clone Frequency (%)
Sample-1
Sample-10
Sample-100
Sample-101
Sample-102
Sample-103
Sample-104
Sample-105
Sample-106
Sample-107
Sample-108
Sample-109
Sample-11
Sample-110
Sample-111
Sample-112
Sample-113
Sample-114
Sample-115
Sample-116
Sample-117
Sample-118
Sample-119
Sample-12
Sample-120
Sample-121
Sample-122
Sample-123
Sample-124
Sample-125
Sample-126
Sample-127
Sample-128
Sample-129
Sample-13
Sample-130
Sample-131
Sample-132
Sample-133
Sample-134
Sample-135
Sample-136
Sample-137
Sample-138
Sample-139
Sample-14
Sample-140
Sample-141
Sample-142
Sample-143
Sample-144
Sample-145
Sample-146
Sample-147
Sample-148
Sample-149
Sample-15
Sample-150
Sample-151
Sample-152
Sample-153
Sample-154
Sample-155
Sample-156
Sample-157
Sample-158
Sample-159
Sample-16
Sample-160
Sample-161
Sample-162
Sample-163
Sample-164
Sample-165
Sample-166
Sample-167
Sample-168
Sample-169
Sample-17
Sample-170
Sample-171
Sample-172
Sample-173
Sample-174
Sample-175
Sample-176
Sample-177
Sample-178
Sample-179
Sample-18
Sample-180
Sample-181
Sample-182
Sample-183
Sample-184
Sample-185
Sample-186
Sample-187
Sample-188
Sample-189
Sample-19
Sample-190
Sample-2
Sample-20
Sample-21
Sample-22
Sample-23
Sample-24
Sample-25
Sample-26
Sample-27
Sample-28
Sample-29
Sample-3
Sample-30
Sample-31
Sample-32
Sample-33
Sample-34
Sample-35
Sample-36
Sample-37
Sample-38
Sample-39
Sample-4
Sample-40
Sample-41
Sample-42
Sample-43
Sample-44
Sample-45
Sample-46
Sample-47
Sample-48
Sample-49
Sample-5
Sample-50
Sample-51
Sample-52
Sample-53
Sample-54
Sample-55
Sample-56
Sample-57
Sample-58
Sample-59
Sample-6
Sample-60
Sample-61
Sample-62
Sample-63
Sample-64
Sample-65
Sample-66
Sample-67
Sample-68
Sample-69
Sample-7
Sample-70
Sample-71
Sample-72
Sample-73
Sample-74
Sample-75
Sample-76
Sample-77
Sample-78
Sample-79
Sample-8
Sample-80
Sample-81
Sample-82
Sample-83
Sample-84
Sample-85
Sample-86
Sample-87
Sample-88
Sample-89
Sample-9
Sample-90
Sample-91
Sample-92
Sample-93
Sample-94
Sample-95
Sample-96
Sample-97
Sample-98
Sample-99
