# Supplementary material for: Seven-chain adaptive immune receptor repertoire analysis in rheumatoid arthritis reveals novel features associated with disease and clinically relevant phenotypes
Source: Genome Biol. 2024 Mar 11;25:68. doi: 10.1186/s13059-024-03210-0 (PMC10926600; doi:10.1186/s13059-024-03210-0)
Supplement: Supplementary file 37 — Additional file 37: Figure S9. Clone publicity profile of the study population. Graphical representation of the percentage of clones that are shared by more than two individuals (i.e., publicity degree) at the chain level. The clone publicity profile is separately shown for healthy individuals and rheumatoid arthritis patients. For each condition, the highest number of individuals sharing a clone is annotated on the right side. Abbreviations: CTRL, healthy individuals; N, sample size; RA, rheumatoid arthritis. [file 13059_2024_3210_MOESM37_ESM.pdf]

Fig S9. Clone publicity profile of the study population. Graphical representation of the percentage of clones that are shared by more than two individuals (i.e., publicity degree) at the chain level. The clone publicity profile is separately shown for healthy individuals and rheumatoid arthritis patients. For each condition, the highest number of individuals sharing a clone is annotated on the right side. Abbreviations: CTRL, healthy individuals; N, sample size; RA, rheumatoid arthritis.

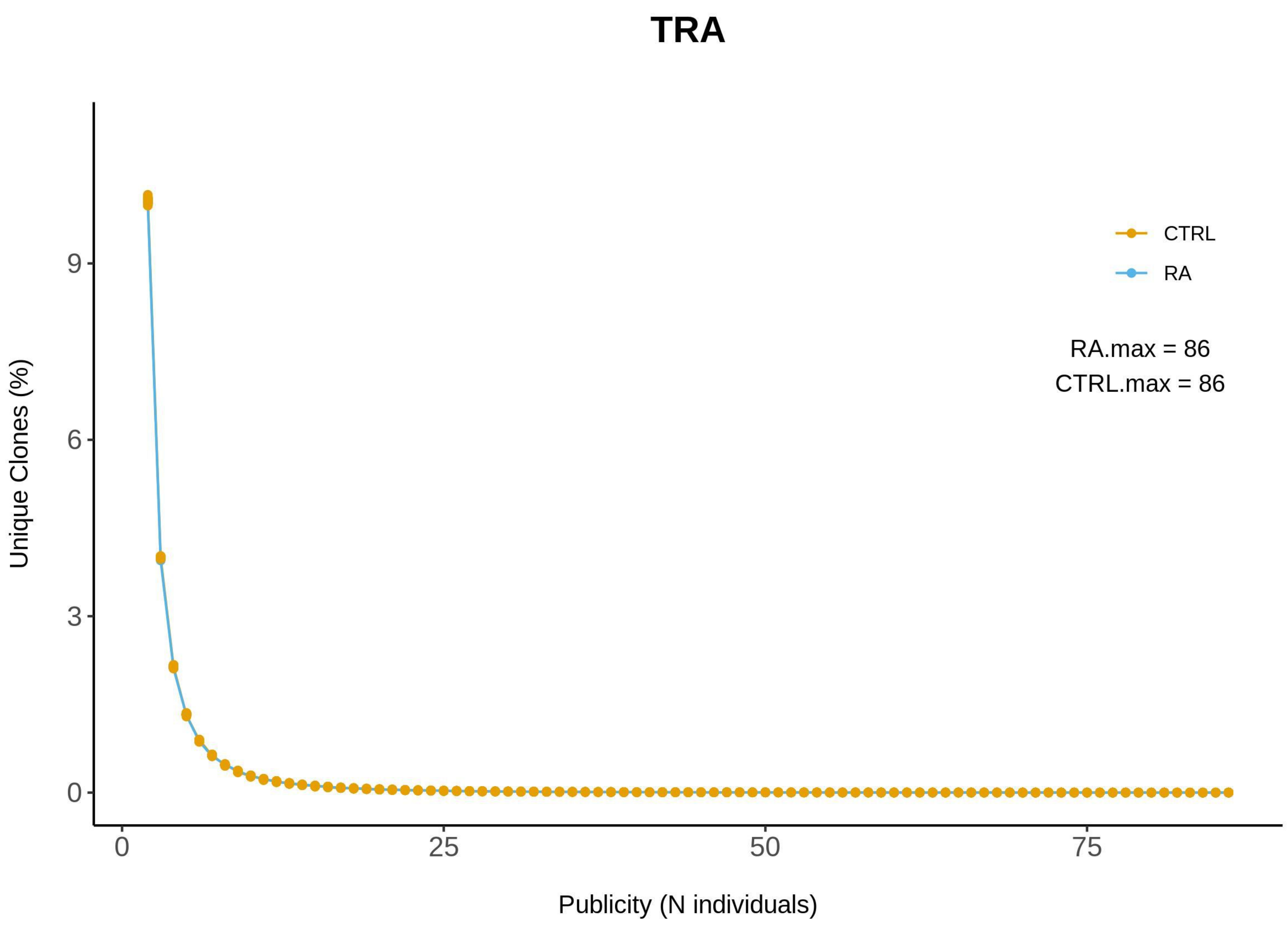
TRA
Unique Clones (%)
9
6
3
0
0
25
50
75
Publicity (N individuals)
CTRL
RA
RA.max = 86
CTRL.max = 86

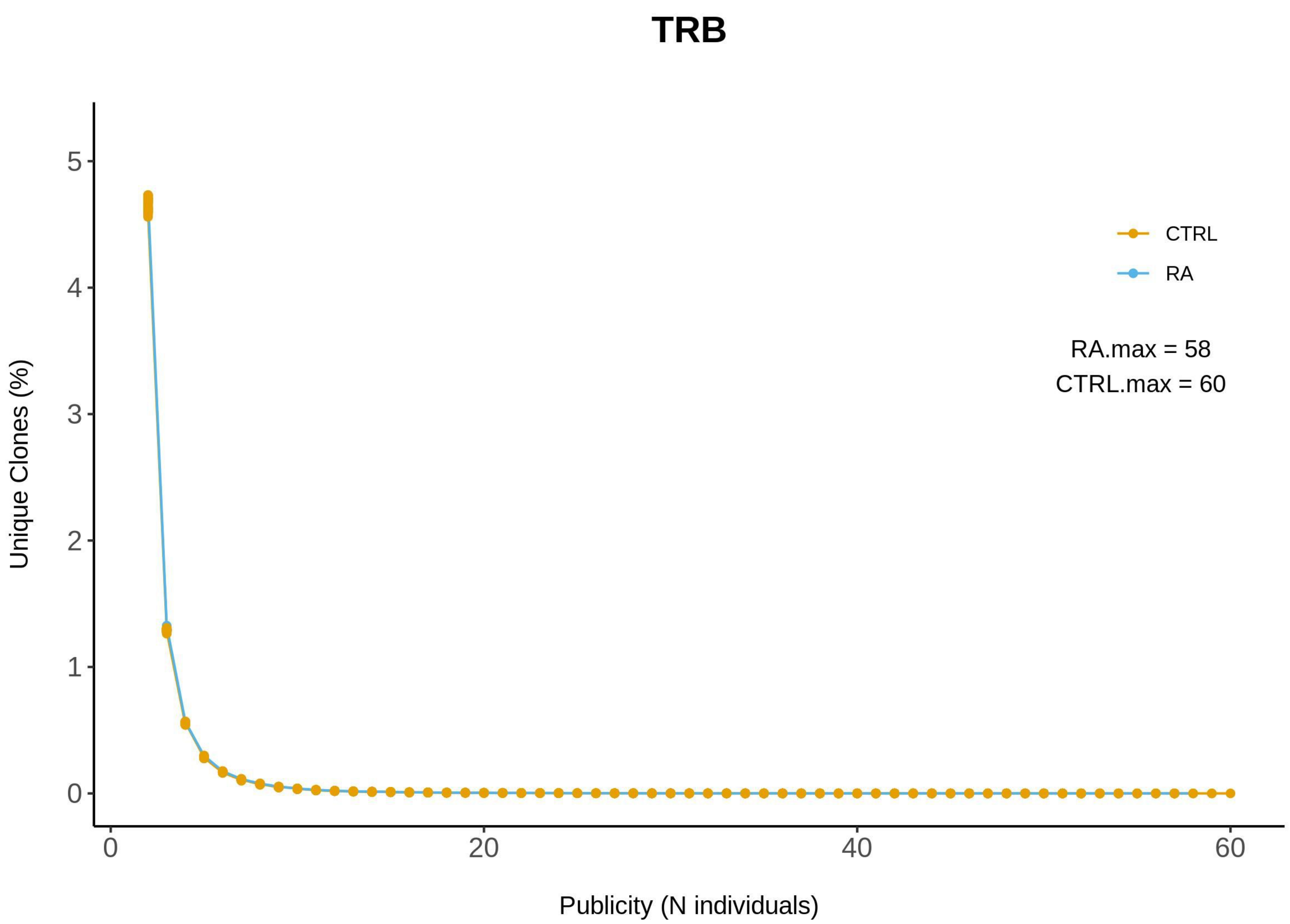

TRB
Unique Clones (%)
5
4
3
2
1
0
0
20
40
60
Publicity (N individuals)
CTRL
RA
RA.max = 58
CTRL.max = 60

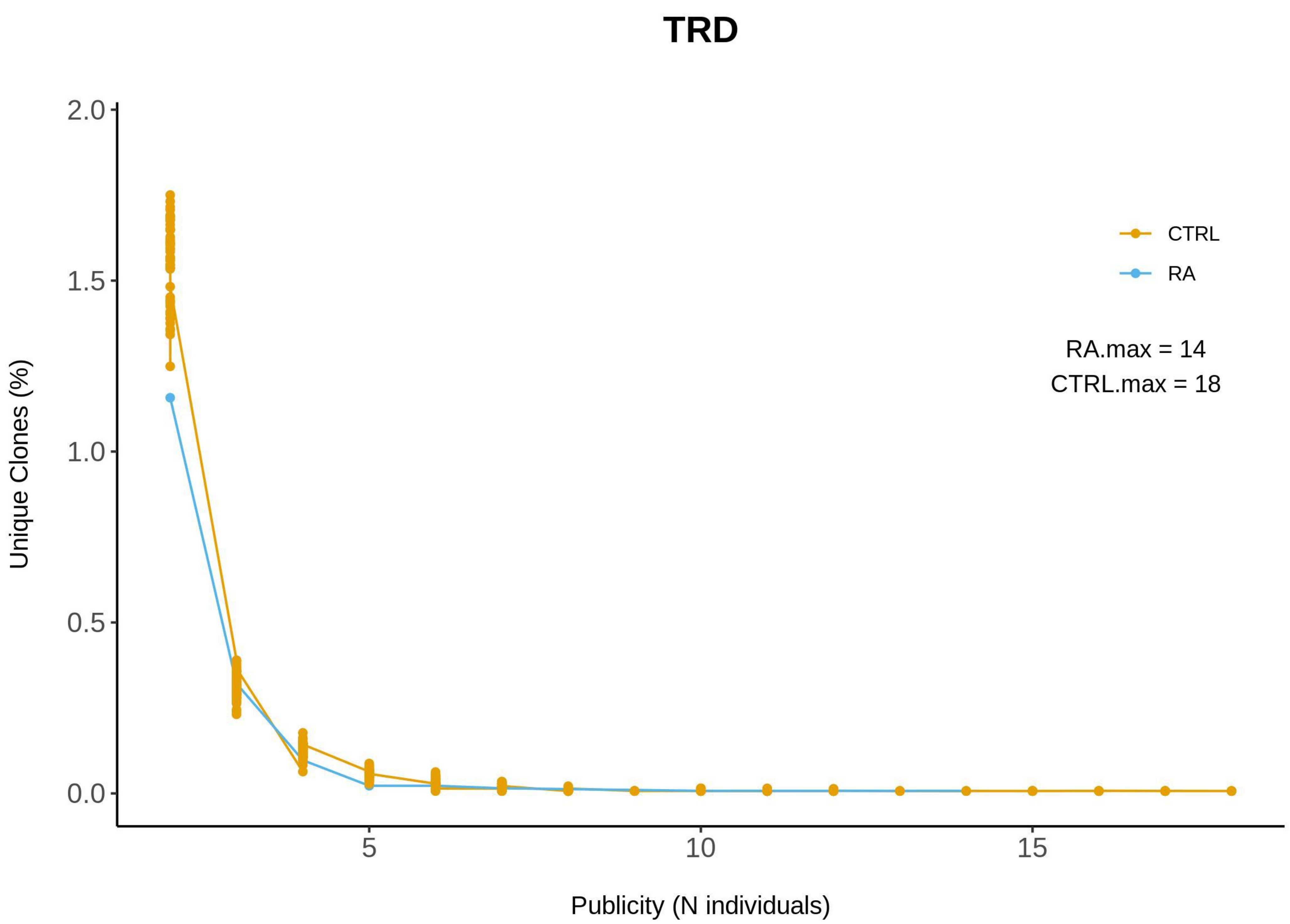

TRD
Unique Clones (%)
2.0
1.5
1.0
0.5
0.0
5
10
15
Publicity (N individuals)
CTRL
RA
RA.max = 14
CTRL.max = 18

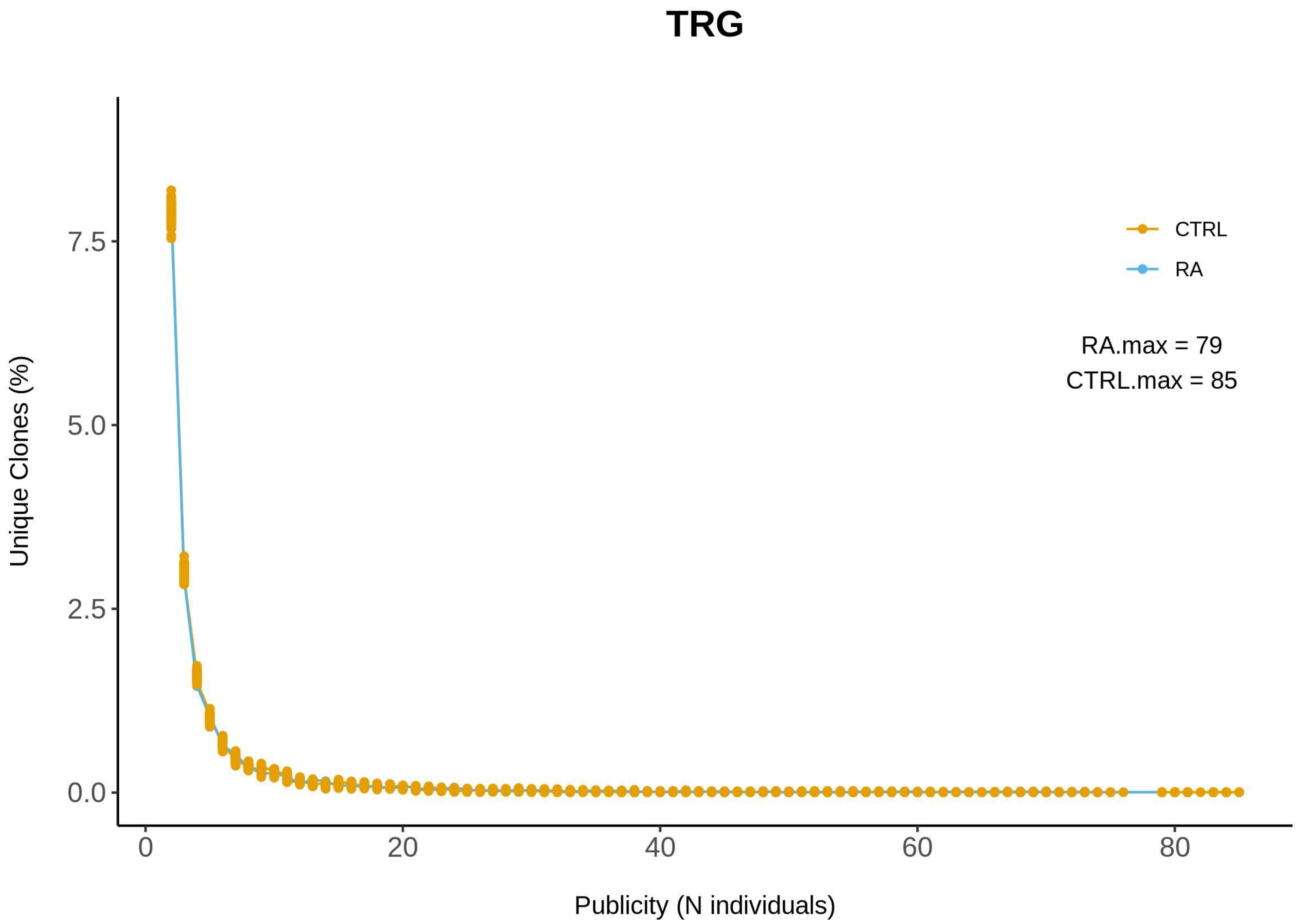
TRG
Unique Clones (%)
7.5
5.0
2.5
0.0
0
20
40
60
80
Publicity (N individuals)
CTRL
RA
RA.max = 79
CTRL.max = 85

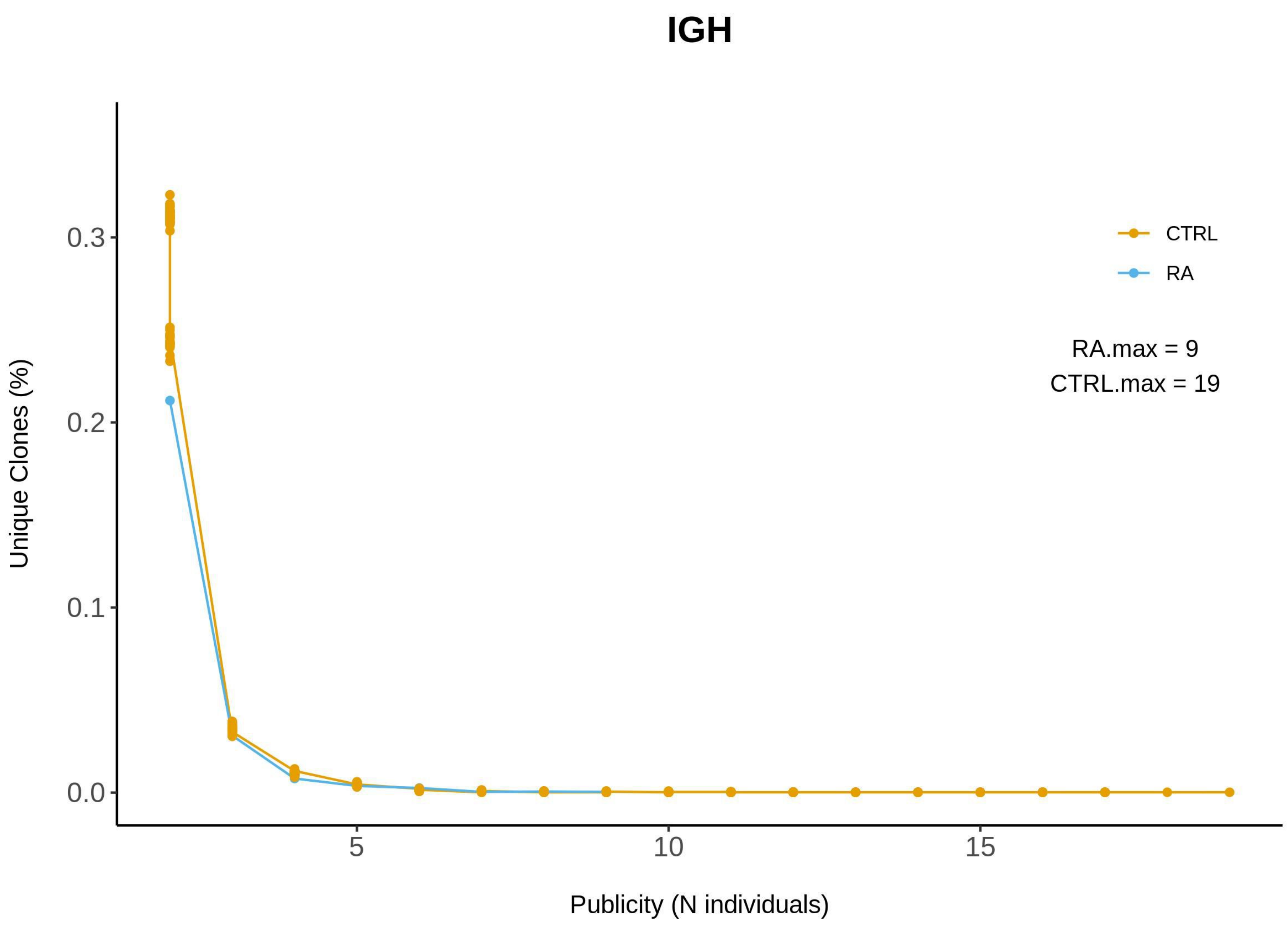

IGH
Unique Clones (%)
0.3
0.2
0.1
0.0
5
10
15
Publicity (N individuals)
CTRL
RA
RA.max = 9
CTRL.max = 19

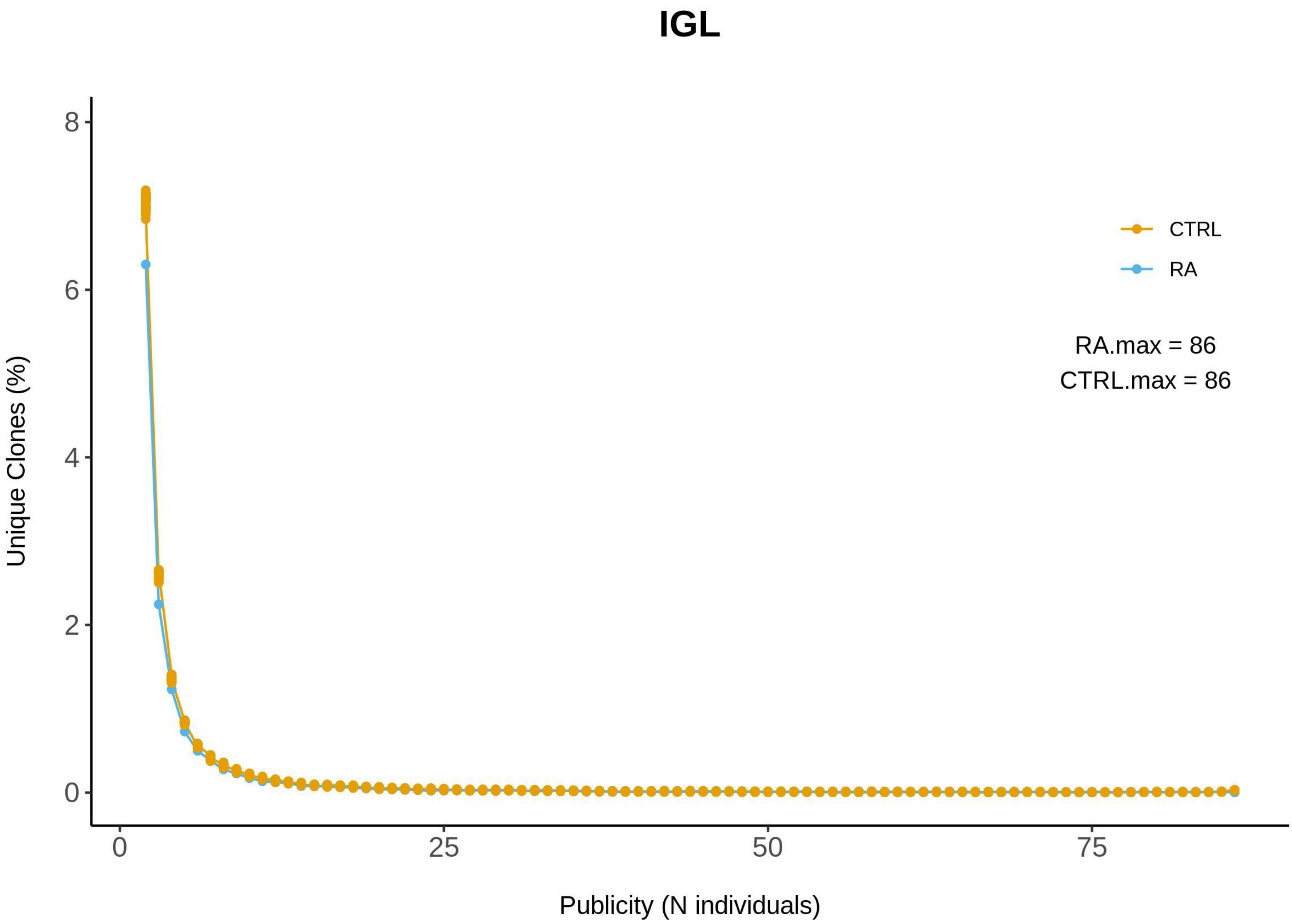
IGL
Unique Clones (%)
Publicity (N individuals)
CTRL
RA
RA.max = 86
CTRL.max = 86
0
2
4
6
8
0
25
50
75

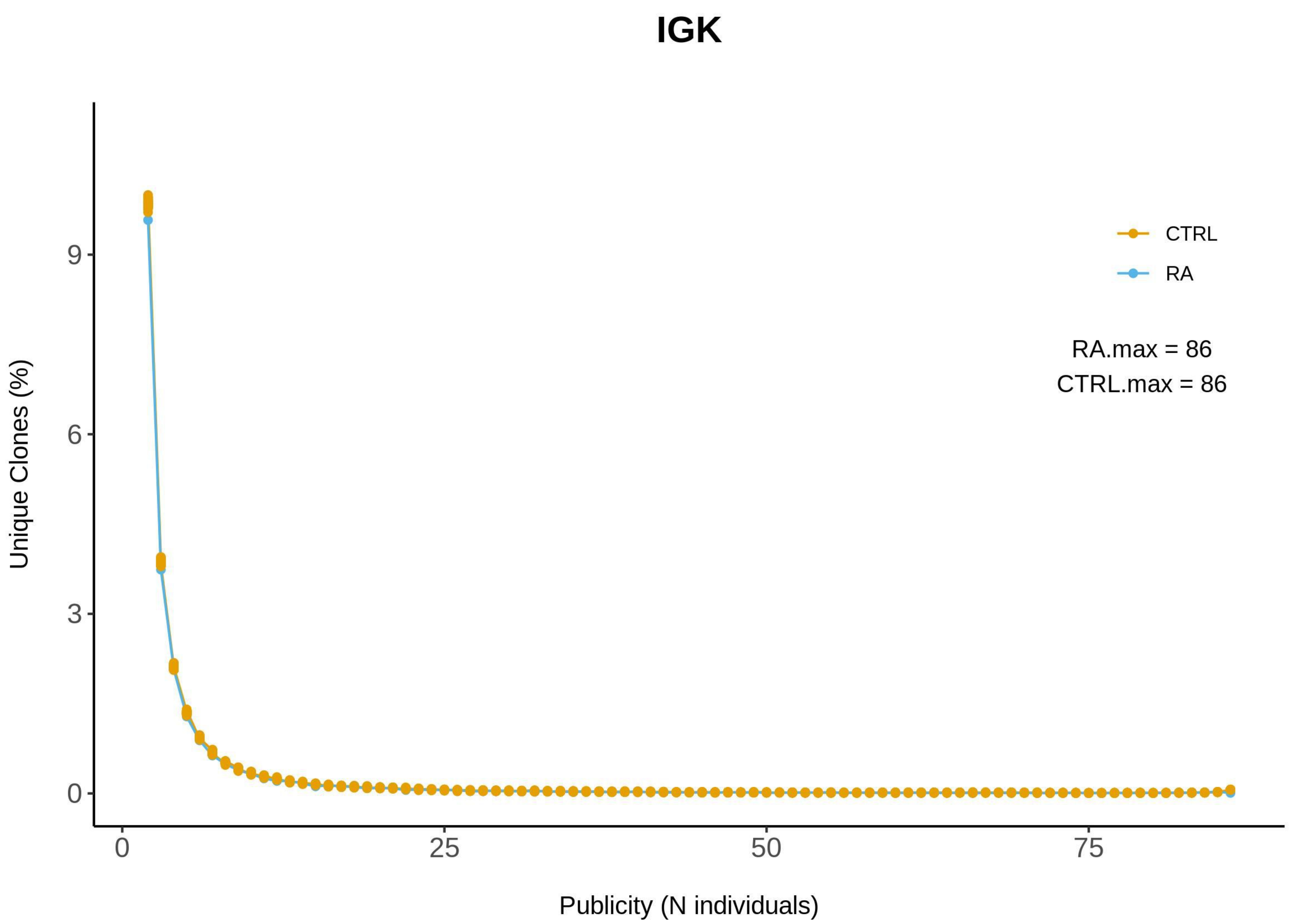
IGK
CTRL
RA
RA.max = 86
CTRL.max = 86
Unique Clones (%)
9
6
3
0
0
25
50
75
Publicity (N individuals)
