# Supplementary material for: Seven-chain adaptive immune receptor repertoire analysis in rheumatoid arthritis reveals novel features associated with disease and clinically relevant phenotypes
Source: Genome Biol. 2024 Mar 11;25:68. doi: 10.1186/s13059-024-03210-0 (PMC10926600; doi:10.1186/s13059-024-03210-0)
Supplement: Supplementary file 40 — Additional file 40. Review history. [file 13059_2024_3210_MOESM40_ESM.docx]

Review History

**First round of review**

**Reviewer 1**

**Were you able to assess all statistics in the manuscript, including the appropriateness of statistical tests used?**

Yes, and I have assessed the statistics in my report.

**Comments to author:**

The authors present an extensive immune receptor repertoire analysis, which is unique in its size and breadth of information on the combination of BCR and TCR repertoires from individual patients. The study provides an important addition to our current understanding of the AIRR in RA and its changes upon treatment. At the current stage, the data as presented raise several questions that will need to be addressed.

In the method section, the authors describe the patient selection and mention that patients should have had at least 2 years of follow-up since diagnosis. All recruited patients had erosive disease defined as erosions in more than one joint group including hands and/or feet. These inclusion criteria suggest that patients will have received a range of different treatments before entering the study. Such treatments could explain differences in the baseline diversity measures observed. The authors should provide the information on prior treatment and discuss how this could have impacted impact the conclusions on AIRR changes upon TNFi treatment.

The authors should further address the stability of AIRR repertoires over time in RA patients. How stable is the TCR and BCR repertoire from patients or healthy donors over the course of 12 weeks without a specific intervention? As a control intervention group or a replication dataset is lacking, this information is important to determine whether the changes in diversity of the repertoire that the authors observe are due to TNFi or a coincidental finding?

In Figure 3, the expression of the different immune chains is expressed in %reads of all reads from the AIRR of one patient. To support the statement of a lower abundance of IGL and a skewing in the IGK/IGL ratio, the authors s hould depict the % of reads for IGL or IGK from the pool of IGL/IGK reads. Like the graphs in figure 1d, it is important to see whether the skewing of the IGK/IGL ratio is present in many patients or in a specific group of patients. In addition, the skewed IGK/IGL ratio could have multiple reasons. For example, IGL-expressing B cells could be more differentiated to the plasmablast/plasma cell compartment producing more antibody sequences compared to other stages of B-cell development. The correlation with the number of plasmablasts/cells could also be an explanation for the differences observed between blood and synovium. The authors should consider these additional explanations and elaborate on this in the manuscript.

The definition of TCR and BCR clones are currently based on identical CDR3aa sequences. Did the authors include V-J gene annotation for this definition? And did the authors consider the use of a certain hamming distance to allow for a certain difference in CDR3aa sequence due to sequencing errors?

Although TNFi treatment seems to have a remarkable impact on the diversity of the AIRR of RA patients, it seems to have little impact on the gene usage profile of patients. Is the increased diversity indeed composed of similar V- and J-genes as those seen in the baseline AIRR? If so, how do the authors explain these two results?

All 60 RA-associated IGL and IGK clones were screened with the PIRD database and the authors state that none of the clones recognize any of the antigens discovered so far. Could the authors elaborate on the number of clones that can be compared to clones described in the database? Do none of the clones recognize any discovered antigen or should it rather be stated that "it cannot be excluded that the BCR of these clones, if produced as a IGH-IGK/IGL combination as a monoclonal antibody, recognize the discovered antigens. However, comparison with the database did not yield any defined recognition of an antigen."

Based on the data presented, the AIRR predicts RA with a 95.2% accuracy if compared to healthy donors. Could this high accuracy be explained by the selected patient group with at least two years of follow-up after diagnosis and erosive disease? Would this prediction be as accurate if the AIRR of newly diagnosed patients would be considered as a predictive value? How does this prediction compare/add to the assessment of a combination of ACPA, RF and inflammatory markers instead of separate comparisons, as the authors discuss?

**Reviewer 2**

**Were you able to assess all statistics in the manuscript, including the appropriateness of statistical tests used?**

Yes, and I have assessed the statistics in my report.

**Comments to author:**

This paper uses "seven chain" adaptive immune receptor sequencing: namely, sequencing of the TRA, TRB, TRD, TRG, IGH, IGL, and IGK immune receptor chains. Each of these chains are then simplified to their CDR3 amino acid sequences, the unique instances of which are called "clones" in the text. The paper then proceeds to perform comparison using the table formed by these various clones and subject phenotype.

Having this massive data set with all of these different immune receptors is indeed novel, and the authors perform analyses that suggest interesting interactions between disease status and repertoire, as well as interaction between treatment response and repertoire.

Overall response

The main "seven chain" novelty of this paper is more technical rather than biological, and forces some analysis decisions that don't feel biologically motivated. Indeed, by trying to pull together this diversity of data types into a single framework, the analysis feels vague and high-level and the conclusions feel suggestive rather than clear. Also, with the exception of a classification tool, the novel joint seven-chain nature of the data isn't really leveraged.

For example, the paper needs to use a definition of some data type which is shared between all seven chains, and so use the definition of a "clone", which as described is an amino acid CDR3 sequence. This is a feature that can be used for all seven chains. Because of effects of the other CDRs, this is already losing some biology: we cannot assume that two immune receptors with the same CDR3 sequence bind the same antigens.

However, when taking this definition to multiple chains creates real problems for interpretation. For example, immunoglobulins diversify via somatic hypermutation, leading to the definition of "clonal family" of sequences that share the same VDJ recombination. Thus the frequency of a given CDR3-clone is a conflation of generation probability, clonal expansion, and degree of somatic hypermutation. This is different than for the TCR case, which expand without mutation. Furthermore, the diversity of these loci varies wildly. Just by virtue of the simpler VJ recombination in the light chain loci, for example, these will have orders of magnitude less diversity than loci with a D gene.

The present manuscript treats these various features on the same footing, but they are not equivalent. I'm not saying that one couldn't come up with a means of analyzing all of this data in a coherent framework, but the following two issues seem significant.

Overall points

(1) Multiple testing

In order for this work to be scientifically sound, we need a close consideration of multiple testing. There are many many analyses presented here, but approaches to handle multiple hypothesis testing don't seem to be used. I don't think the p-values in, say, Figure 1b, are corrected for multiple testing. This can be acceptable for the purposes of a plot if it's made clear.

However, some conclusions are reported that do not appear to be corrected for multiple testing. For example, the manuscript states "At the clinical level (Fig. S2, Table S4), the most consistent signal across diversity measures was found to be a significant increase of clone diversity of the IGK chain in ACPA-positive patients in comparison to ACPA-negative patients". In this, the authors seem to just be taking the most significant p-value across all of the chains, the diversity measures, and the clinical criteria. Because this p-value is 0.00912, this would never survive multiple testing correction and thus should not be reported. There are sections such as "The low diversity that characterizes the AIRR of RA patients is..." in which different statistical tests are reported for different conclusions. This isn't acceptable.

If this has been addressed, please correct me, though all I could find was a FDR analysis for two sub-analyses.

(2) Contracted and expanded clones

If I understand correctly the methodology developed in Puelma Touzel et al (2020) is used to identify "significantly" contracted and expanded clones. This methodology was designed for full TCR sequences, but is used here for CDR3-AA-clones, including in the immunoglobulin locus. I have checked with the authors of this method and they are confirmed that neither using CDR3-AA sequences nor BCR sequences correspond to the assumptions made in the development of their method. Using CDR3-AA-clones might be OK, but to decide if this is the case one would need replicate measurements to calibrate the sensitivity of the method. However, the method definitely does not extend in a simple way to the BCR locus, because of the process of affinity maturation. They are working on an extension to BCRs but this is going to take time and careful consideration.

I believe that these methodological issues drive the extraordinary number of expansions and contractions seen in the IGH, IGK, and IGL loci, and I don't feel clear that these are meaningful.

Specific points

It seems like there is additional relevant work, such as

https://www.sciencedirect.com/science/article/abs/pii/S0896841120300457

https://www.jrheum.org/content/45/7/905

The authors claim that the sequencing procedure is unbiased. That is a strong claim-- even getting a single chain assay to be unbiased is quite difficult. Reference 30, which is cited, does not claim that the sequencing process is unbiased. Is there further evidence of unbiasedness? If not, how is this addressed?

The biggest effect of RA is a reduction of diversity in the IG loci. The current paper can only say that this is a reduction of diversity on the level of clones, whereas more detailed analyses (e.g. Elliott 2020 https://www.sciencedirect.com/science/article/pii/S1521661619306448) find that this is due to expansion of clonal families, and can understand the responsive clones and what is driving their expansion. This includes functional characterization and response to citrullinated antigens.

The "clinical level" conclusions don't appear to be significant when accounting for multiple hypothesis testing. The TNFi results in Figure 1d appear to be robust but corrected p-values should be reported. For Figure 1e, there is a sample size issue here: there are substantially fewer nonresponders, which looks to me to be driving the difference.

I don't understand the right two columns in the Figure 1b/c heatmaps. Are the bubbles meaningful? If so, please describe. Please describe the colors in these heatmaps a little more. I see they are "effect size": does this mean the ratio of the statistic in the two conditions? What was the statistical test done? If a randomization test, I am not sure what is being randomized.

P4L37: "all subsequent AIRR analyses were adjusted for the sequencing depth as well as for sex and age." Is this really all analyses? I did read about how these variables are included as covariates, but does that impact the diversity analyses, e.g.?

What is the scale of the "significance" in Figure 2c? Log p value?

p6L13-14: this p-value is for the properties being shared? How are these being computed?

Figure 2a-- the colors are so light, so that I can't tell if I can see IGH. Perhaps see https://clauswilke.com/dataviz/color-basics.html#color-as-a-tool-to-distinguish

Figure 2e-- what is up with the contracted responders? Why are there concentric pie charts?

P7L7: The IGK has much higher expression in the synovium than IGL. How does that fit into this analysis?

P8L17: Can you provide more details on this "random repertoire"? It is sampled from the (observed?) clones, with uniform weight on each? Does this really represent a reasonable null?

Figure 3c-e: could we have some labels on these figures, like V gene, etc? If I understand correctly, all of the different V genes on the different chains are all put together in the same PC plot. Thus, we could have the same proportional use of the germline genes, but different frequency of chains in the data, and get a striking effect. Could that be happening here? In any case, if my interpretation is correct, then one should reframe the analysis or change it to be only about germline gene usage. This is also true of the association with clinical phenotype in the next section.

CDR3 length analysis and supplementary figure 7: Many of these distributions look nearly identical yet get extraordinary p-values (1e-136!). If we reshuffle cases between RA and control, do we get significant p-values? If so, perhaps it's time to reconsider how the statistical analysis is done. The TRD distribution does look different, but biggest difference is a huge spike at 18 amino acids in the control group. Is that worth examining more closely?

P10L54: this analysis wouldn't survive multiple testing correction.

P11-- Please describe the hold-out strategy in more detail. The accuracy reported is on the held-out set?

The supplementary material must be downloaded from the authors' web site, which I don't think is technically allowable for peer review.

Zenodo or some such service allows for anonymous download.

**Reviewer 3**

**Were you able to assess all statistics in the manuscript, including the appropriateness of statistical tests used?**

Yes, and I have assessed the statistics in my report.

**Comments to author:**

In the manuscript "Seven chain adaptive immune receptor repertoire analysis in rheumatoid arthritis: association to disease and clinically relevant phenotypes", the authors perform a comprehensive analysis of the BCR and TCR repertoires in RA patients before and after TNFi treatment, and compared with a baseline of healthy individuals. They find that in RA patients the diversity of the BCR repertoire is reduced and other features are skewed. TNFi treatment partially normalizes the repertoires in individuals, which is correlated with clinical response to the treatment.

The work is clearly presented, but I have some very fundamental methodological concerns that in my opinion prevent this manuscript from being accepted in its current form.

Specific points:

1. Especially regarding figure 3C,D,E and also several other figures- the differences between the patients and the healthy controls seem almost too good to be true… Such clear differences in V J usage have never been previously properly reported, and when something like that was published it was never validated. This raises a strong concern for batch effects. Did the authors sequence the patients and healthy repertoires together or separated? Were the libraries prepared simultaneously for all groups? There is no info on any quality control steps that were taken.

2. There is no systematic investigation towards the source of these huge differences. Is it T cells? B cells? Is it the ratio between CD4 and CD8 that was not discussed?

3. What is the hypothesis behind these differences?

4. What is the mechanism behind the clonal selectivity of the TNFi treatment? This point is strange to me, as this is a general cytokine.

5. Methods sections indicate that the analysis was not careful enough. For example, the use of MiXCR for BCR is not adequate for such analysis. How was the alignment performed? Which reference set? Etc.

6. The definition of meta-clones is not clear. What does "high similarity" mean? These details must be described carefully in a way that the results can be reproduced.

7. Page 7 first paragraph, says that there is a shift from IGL to IGK expression in the blood of RA patients, and the authors try to explain this by migration of the IGL harboring B cells from the blood to the synovium. There is indeed an increase in IGL expression in the synovium samples (figure 3B), but an even stronger increase in IGK, so the hypothesis cannot be correct. It also seems unlikely that the increase in IGK is caused by the general increase in the blood, since the increase in the blood is very weak and not statistically significant.

**Authors Response**

**Point-by-point responses to the reviewers’ comments:**

Reviewer #1

The authors present an extensive immune receptor repertoire analysis, which is unique in its size and breadth of information on the combination of BCR and TCR repertoires from individual patients. The study provides an important addition to our current understanding of the AIRR in RA and its changes upon treatment. At the current stage, the data as presented raises several questions that will need to be addressed.

*Response: We thank the reviewer for appreciating the uniqueness of the present manuscript, as well as for highlighting the novel insights that we provide into the AIRR and how it is modulated by therapy in RA. We are now addressing all the questions raised in his/her revision.*

1. In the method section, the authors describe the patient selection and mention that patients should have had at least 2 years of follow-up since diagnosis. All recruited patients had erosive disease defined as erosions in more than one joint group including hands and/or feet. These inclusion criteria suggest that patients will have received a range of different treatments before entering the study. Such treatments could explain differences in the baseline diversity measures observed. The authors should provide the information on prior treatment and discuss how this could have impacted impact the conclusions on AIRR changes upon TNFi treatment.

*Response: Following the reviewer’s comment, we have evaluated the range of treatments of the RA patients before entering the study. Since the European League Against Rheumatism (EULAR) recommends immunosuppressant therapy before anti-TNF agents (Smolen et al., 2017), which are commonly administrated when first-line immunosuppressants fail (Taylor et al., 2022; Pappas et al., 2021; Silvagni et al., 2018), as expected, RA patients had been previously treated with disease-modifying antirheumatic drugs (DMARDs). From these, most of the patients had received methotrexate (53.25%) or leflunomide (29.87%). Following the reviewer’s indication, we have now added this therapeutic summary to Table S1 (pages 4, 13 and 41). We agree with the reviewer that previous immunosuppressive drugs could have influenced the baseline diversity of the AIRR. To evaluate this, we have compared the immune repertoire diversity (i.e., D50) at baseline between RA patients that had been treated and untreated with methotrexate. In this analysis, we have found that the most common immunosuppressive drug does not alter the diversity of RA patients at baseline (Table 1).*

*Table 1. Summary statistics of the diversity association analyses conducted between methotrexate-treated and untreated RA patients at baseline and chain-wide scale.*

*Following the reviewer’s suggestion, we have commented in the discussion (pg. 24) that first-line drugs for RA treatment are unlikely to impact on the AIRR diversity.*

2. The authors should further address the stability of AIRR repertoires over time in RA patients. How stable is the TCR and BCR repertoire from patients or healthy donors over the course of 12 weeks without a specific intervention? As a control intervention group or a replication dataset is lacking, this information is important to determine whether the changes in diversity of the repertoire that the authors observe are due to TNFi or a coincidental finding?

*Response: We agree with the reviewer that immunosequecing additional blood samples from healthy controls and untreated patients after 12 weeks of follow-up could provide additional information for the study. One limitation to this is that all controls included here are anonymous donors that attended to the Spanish National DNA Bank and, therefore, they cannot be recontacted. This precludes the obtention of additional samples from this group of healthy individuals. In this study, we prioritized the collection and immunosequencing of longitudinal samples from patients under TNFi therapy to better understand the impact of this pharmacological intervention in the patient’s AIRR and in relation to clinical response. In line with this, previous longitudinal AIRR-seq analyses at the single-chain level have been exclusively focused on treated samples to determine the effect that other biological therapies like rituximab have on the BCR of RA patients (Pollastro et al., 2019). Therefore, our study design is robust to identify treatment-induced changes in the repertoire diversity, which we acknowledge and discuss that should be validated when new chain-wide immunosequencing data is generated (pg. 24).*

3. In Figure 3, the expression of the different immune chains is expressed in %reads of all reads from the AIRR of one patient. To support the statement of a lower abundance of IGL and a skewing in the IGK/IGL ratio, the authors should depict the % of reads for IGL or IGK from the pool of IGL/IGK reads. Like the graphs in figure 1d, it is important to see whether the skewing of the IGK/IGL ratio is present in many patients or in a specific group of patients. In addition, the skewed IGK/IGL ratio could have multiple reasons. For example, IGL-expressing B cells could be more differentiated to the plasmablast/plasma cell compartment producing more antibody sequences compared to other stages of B-cell development. The correlation with the number of plasmablasts/cells could also be an explanation for the differences observed between blood and synovium. The authors should consider these additional explanations and elaborate on this in the manuscript.

*Response: The use of this method to amplify the seven chains of the immune repertoire under the same conditions in one PCR reaction allows to directly compare the expression level of these chains (Niu et al., 2020; Han J et al., 2020). In order to generate Figure 3, we have implemented the methodology used in Niu et al., 2020 (Figure 1a). Following the reviewer’s indication, we now depict the % of IGK reads from the total pool of IGK and IGL reads in the new version of Figure 3A (pages 7 and 32). We agree that this graphical representation more clearly demonstrates the existence of a skewed IGK/IGL ratio in RA patients. More specifically, this skewing was found to be very common among RA patients: 84.8% of RA patients have a higher % of IGK reads from the total pool of IGK and IGL reads compared to healthy controls.*

*We thank the reviewer for raising an additional hypothesis that could also explain the skewed IGK/IGL ratio detected in RA patients. Although the plasmablast concentration in blood and synovium was not measured at the individual level in the dataset Lewis et al., 2019, we think that the alternative biological explanation raised by the reviewer is relevant for the interpretation of our work and, therefore, we have now incorporated it in the new version of the manuscript (pg. 25).*

4. The definition of TCR and BCR clones are currently based on identical CDR3aa sequences. Did the authors include V-J gene annotation for this definition? And did the authors consider the use of a certain hamming distance to allow for a certain difference in CDR3aa sequence due to sequencing errors?

*Response: As indicated by the reviewer, in the manuscript section “Definition of AIRR variables” we describe that clones were defined by the CDR3 amino acid sequences and, therefore, V-J gene annotation was not incorporated into the clone definition. Although the same CDR3 amino acid sequence can be associated to different combinations of V-J segments, we have found that, in practice, this occurs only in a few cases of CDR3 amino acid sequences (TCR=0.8%; BCR=4.5%). Despite this, in order to confirm that our analyses are not compromised by clone definition, we have recalculated diversity using three different clonal definitions: CDR3 amino acid sequence, CDR3 amino acid sequence + VJ segments and CDR1-->FR4 amino acid sequence (FR1 is only partially covered due to sequencing limitations). These analyses show a very strong correlation between diversity indexes estimated using different clonal definitions (TRAr2=0.991-0.999; TRBr2= 0.9995-0.999; TRDr2=0.992-0.999; TRGr2=0.991-0.999; IGHr2=0.935-0.999; IGKr2=0.8833-0.997 and IGLr2=0.896-0.997). These results clearly demonstrate that clonal definition has a negligible impact in the analyses here performed.*

*In regards to the application of additional sequencing filters like hamming distance, we followed the protocol of reference for UMI data processing described in Turchaninova et al., 2016. In this analytical protocol, the authors describe that, after UMI collapse, PCR-error is largely corrected for the presence of high-quality consensus sequences. Importantly, any additional filtering step like the MiXCR’s clustering procedure (i.e., analogous to the hamming distance) would have a negative impact and reduce clone discovery.*

5. Although TNFi treatment seems to have a remarkable impact on the diversity of the AIRR of RA patients, it seems to have little impact on the gene usage profile of patients. Is the increased diversity indeed composed of similar V- and J-genes as those seen in the baseline AIRR? If so, how do the authors explain these two results?

*Response: The question raised by the reviewer is of major interest to better understand the nature of the clones that are contracted. In our analyses we have found that contraction is higher than expansion for six out of the seven chains (Fig 2b). Our results therefore indicate that TNF inhibition promotes an overall clonal contraction of the immune repertoire and, consequently, this leads to the increased diversity detected in RA patients after 12 weeks of treatment. Following the reviewer’s indication, we have investigated if contracted clones are characterized by the use of V, J and VJ gene segments that, at baseline, are also more used by the immune repertoire of RA patients compared to controls. In this additional screening, we have found that 85.7% of the most widely used V segments in contracted clones from RA patients (i.e., detected in n>60 patients) also show a significantly higher usage in RA patients compared to controls (P<0.05). This new information based on the reviewer’s comment is relevant and has been now included as part of Table S7 and also mentioned in the Results section of the revised manuscript (pages 8 and 41). In conclusion, we have found that gene segments that are most widely present in contracted clones by TNFi therapy tend to be also gene segments that are associated to the disease when comparing to controls. These findings are totally consistent with our original results and provide additional insights into how differentially used segments at baseline are related to the repertoire modulation associated to TNF inhibition when restoring BCR diversity.*

6. All 60 RA-associated IGL and IGK clones were screened with the PIRD database and the authors state that none of the clones recognize any of the antigens discovered so far. Could the authors elaborate on the number of clones that can be compared to clones described in the database? Do none of the clones recognize any discovered antigen or should it rather be stated that "it cannot be excluded that the BCR of these clones, if produced as a IGH-IGK/IGL combination as a monoclonal antibody, recognize the discovered antigens. However, comparison with the database did not yield any defined recognition of an antigen."

*Response: Following the reviewer’s indication, we have now included the number of clones per chain that were available for analysis in the PIRD database and, therefore, that can be compared to the set of 60 RA-associated IGL and IGK clones (pg. 8).*

*Although our comparison with the PIRD database did not yield any defined recognition of an antigen, we totally agree with the reviewer that we cannot discard the recognition of discovered antigens by the BCR of these 60 RA-associated IGL and IGK clones if produced as IGH-IGK/IGL monoclonal antibody. We have now modified this statement accordingly (pg. 8).*

7. Based on the data presented, the AIRR predicts RA with a 95.2% accuracy if compared to healthy donors. Could this high accuracy be explained by the selected patient group with at least two years of follow-up after diagnosis and erosive disease? Would this prediction be as accurate if the AIRR of newly diagnosed patients would be considered as a predictive value? How does this prediction compare/add to the assessment of a combination of ACPA, RF and inflammatory markers instead of separate comparisons, as the authors discuss?

*Response: We appreciate the reviewer’s comments aimed at evaluating the translational potential of our molecular predictor. Given that anti-TNF drugs are specially indicated to patients with erosions and usually administrated after first-line DMARDs therapy (additional information provided now in Table S1), samples from newly diagnosed patients with these two clinical features are very scarce at the outpatient’s clinics. Although additional studies are needed to determine if the phenotypic characteristics of our cohort impact on the predictor’s accuracy (see paragraph below), the present work demonstrates that our molecular predictor is generalizable and not exclusively indicated for RA patients with rare clinical phenotypes like non-erosive disease.*

*The second question raised by the reviewer is essential to determine to which extent our molecular classifier could be used as a diagnostic tool for RA patients in their initial stages. Leveraging our analytical pipeline to build a molecular predictor of pre-RA rather than newly diagnosed RA can be of even greater interest in the clinical setting (Kissel et al., 2020). For this objective, new samples from individuals at risk for RA (i.e., having high titers of ACPA or moderate to low ACPA with positivity to Rheumatoid Factor) need to be collected and further immunosequenced to build the multi-chain AIRR predictor of pre-RA patients. However, this analysis is out of the scope of the present work.*

*Finally, we have compared the predictive performance between our molecular classifier and a new classifier constructed by combining the serologic information available in our individuals (i.e., ACPA and RF). In this analysis (See Table 2 in ResponsesToReviewers document updated as Personal Cover), we have found that the accuracy of the serologic predictor (94.05%) is lower, but comparable, than the accuracy of the multi-chain AIRR predictor (95.20%). Despite comparable accuracies, the serologic predictor failed on the identification of 12% of RA patients while our molecular predictor was able to properly identify 100% of RA patients failing only on the identification of 8% of controls. This is one of the most important findings from the present study. In conclusion, we provide a molecular classifier with an unprecedented diagnostic prediction that could be used in combination with the serologic markers suggested by the reviewer to correctly predict all seronegative patients, an objective that has been extremely elusive until now. New studies should be undergone to evaluate its performance in the earliest stages of the disease.*

*Note: when reviewing the inclusion criteria that had to be fulfilled by the patients enrolled in the present work, we have detected a typographic error related to the year of the ACR/EULAR classification criteria used here. Accordingly, “ACR/EULAR 2020” has been now replaced by “ACR/EULAR 2010” (pg. 13)*

Reviewer#2

This paper uses "seven chain" adaptive immune receptor sequencing: namely, sequencing of the TRA, TRB, TRD, TRG, IGH, IGL, and IGK immune receptor chains. Each of these chains are then simplified to their CDR3 amino acid sequences, the unique instances of which are called "clones" in the text. The paper then proceeds to perform comparison using the table formed by these various clones and subject phenotype. Having this massive data set with all of these different immune receptors is indeed novel, and the authors perform analyses that suggest interesting interactions between disease status and repertoire, as well as interaction between treatment response and repertoire.

*Response: We thank the reviewer for appreciating the novelty of the large AIRR-seq dataset generated in the present study. We also thank the reviewer for appreciating the clinical interest of our discoveries. We herein include a point-by-point response to each of his/her comments.*

1. The main "seven chain" novelty of this paper is more technical rather than biological, and forces some analysis decisions that don't feel biologically motivated. Indeed, by trying to pull together this diversity of data types into a single framework, the analysis feels vague and high-level and the conclusions feel suggestive rather than clear. Also, with the exception of a classification tool, the novel joint seven-chain nature of the data isn't really leveraged.

For example, the paper needs to use a definition of some data type which is shared between all seven chains, and so use the definition of a "clone", which as described is an amino acid CDR3 sequence. This is a feature that can be used for all seven chains. Because of effects of the other CDRs, this is already losing some biology: we cannot assume that two immune receptors with the same CDR3 sequence bind the same antigens.

*Response: We agree with the reviewer that an important aspect of the novelty of our manuscript is the use of a cutting-edge technology that is capable of performing an unbiased and simultaneous analysis of the seven immune receptor chains in a single assay (Han & Lotze, 2020). With this tool we have been able to undercover different new properties of the adaptive immune response associated to RA, as well as demonstrating its potential as a diagnostic tool. By combining the information from different chains from the TCR and BCR repertoires we have developed a classifier with unprecedented disease diagnosis accuracy. The RA predictor is therefore a clear example of the power of multi-chain integration.*

*Our study also provides new features on the biology of RA. By sequencing both small immunoglobulin chains, IGL and IGK, we have found evidence of an increased migration rate of IGL-harboring B cells to the RA synovial tissue (Fig. 3). To date, there is very little knowledge of the functional differences between IGK and IGL that could explain this variability. It has been described that IGK and IGL differ in their glycosylation levels and CDR3 physicochemical properties (Townsend CL, ‘16). Our study suggests that these types of differences could be very important in autoimmune pathology and could be the basis of new immunological properties. Together with the analysis of the immunoglobulin light chain variability, a unique feature of our study is that we have analyzed the disease association to TRG and TRG, the two chains that determine antigen specificity in γδ T cells. Although γδ T cells have not been studied in depth, there is increasing evidence showing the role of these low-frequency subtype of T cells in autoimmune disease pathology (Bank, 2020; Paul & Lal, 2015). In RA, for example, accumulation of γδ T clones has been detected in the inflamed synovium (Holoshitz J, ‘99). In our work, the role of γδ T cells in RA pathogenesis is further supported by a highly significant enrichment of long TRD in disease compared to controls (Fig. S7). The length of TRD clones was also found to be significantly different between ACPA-positive and ACPA-negative patients, and between high and low disease activity patients (Fig. S7), supporting the expansion of self-reactive clones from this T cell type (Wardemann H, ‘03).*

*We agree with the reviewer that CDR1 and CDR2 are also important variability regions, especially for B cells. Accordingly, we have used this data in the computation of the somatic hypermutation (SHM) index. We acknowledge that the use of the two CDRs in our analyses was not clearly exposed in the initial version of our manuscript. We have now recalculated diversity using alternative clone definitions to investigate if our analyses could be compromised when considering other CDR regions. For this objective, we have used three different clonal definitions: CDR3 amino acid sequence, CDR3 amino acid sequence + VJ segments and CDR1-->FR4 amino acid sequence (FR1 is only partially covered due to sequencing limitations). These analyses show a very strong correlation between diversity indexes estimated using different clonal definitions (TRAr2=0.991-0.999; TRBr2= 0.9995-0.999; TRDr2=0.992-0.999; TRGr2=0.991-0.999; IGHr2=0.935-0.999; IGKr2=0.8833-0.997 and IGLr2=0.896-0.997). These results clearly demonstrate that considering CDR1 and CDR2 has negligible effect on the immune repertoire diversity. In the new version of the manuscript, and in line with the potential utility of CDR1 and CDR2 raised by the reviewer, we have now emphasized this aspect in the revised version of the manuscript (pages 16 and 17).*

2. However, when taking this definition to multiple chains creates real problems for interpretation. For example, immunoglobulins diversify via somatic hypermutation, leading to the definition of "clonal family" of sequences that share the same VDJ recombination. Thus the frequency of a given CDR3-clone is a conflation of generation probability, clonal expansion, and degree of somatic hypermutation. This is different than for the TCR case, which expand without mutation. Furthermore, the diversity of these loci varies wildly. Just by virtue of the simpler VJ recombination in the light chain loci, for example, these will have orders of magnitude less diversity than loci with a D gene. The present manuscript treats these various features on the same footing, but they are not equivalent. I'm not saying that one couldn't come up with a means of analyzing all of this data in a coherent framework, but the following two issues seem significant.

*Response: The reviewer raises an immunological feature of BCR repertoire that is relevant AIRR-seq analyses. We acknowledged this specific diversity mechanism in the Introduction of the original manuscript (pg. 3). It is also a known feature of the immune repertoire that, compared to TRA and IGL/IGK chains, the clonal diversity is larger for TRB and IGH due to the addition of a D gene segment during DNA recombination process that occurs during αβ-T and B lymphocyte development, respectively. Based on all these aspects, we did not analyze the differences between chains that have constitutively different diversities (pages 4 and 5). Instead, we analyzed these diversities in relation to features of interest like disease or therapeutic response. We do agree with the reviewer that this is a key aspect that must not be forgotten so as not to lead to erroneous interpretations on the results of our study.*

3. In order for this work to be scientifically sound, we need a close consideration of multiple testing. There are many analyses presented here, but approaches to handle multiple hypothesis testing don't seem to be used. I don't think the p-values in, say, Figure 1b, are corrected for multiple testing. This can be acceptable for the purposes of a plot if it's made clear.

However, some conclusions are reported that do not appear to be corrected for multiple testing. For example, the manuscript states "At the clinical level (Fig. S2, Table S4), the most consistent signal across diversity measures was found to be a significant increase of clone diversity of the IGK chain in ACPA-positive patients in comparison to ACPA-negative patients". In this, the authors seem to just be taking the most significant p-value across all of the chains, the diversity measures, and the clinical criteria. Because this p-value is 0.00912, this would never survive multiple testing correction and thus should not be reported. There are sections such as "The low diversity that characterizes the AIRR of RA patients is..." in which different statistical tests are reported for different conclusions. This isn't acceptable.

If this has been addressed, please correct me, though all I could find was a FDR analysis for two sub-analyses.

*Response: We agree with the reviewer that multiple testing correction must be applied to reduce the probability of identifying false positive results when multiple statistical tests are simultaneously performed. For this objective, we have applied this adjustment in all analyses involving multiple comparisons: single- and meta-clone association analyses, differential gene usage analysis as well as GLIPH analysis. The unique exception to this was the longitudinal analysis at the single-clone level that we performed using the novel NoisET method. For this, followed the procedure as implemented in the longitudinal study of reference performed by the authors of the method (Minervina et al., 2021) In this approach the posterior probability of expansion or contraction is computed for each clone. This measure (<0.05) is subsequently used to identify significantly expanded or contracted clones. We have now discussed this important methodological aspect in the revised version of the manuscript (pg. 27).*

*In the particular diversity analysis raised by the reviewer, we have evaluated only a small number of measures (n=5) with the unique purpose of checking the consistency across complementary diversity measures, as previously recommended (Kaplinsky & Arnaout, 2016). These measures are not independent among them and, therefore, multiple testing correction would not be appropriate. The diversity analysis presented here has been conducted based on a recent TCR study that explored the repertoire diversity of RA patients through three complementary metrics (Jiang et al., 2020). Fig S2, Table S4 and the statement mentioned by the reviewer refer to the results of the diversity analysis conducted at the clinical phenotype level.*

*In the present study, we have not just taken the most significant p-value across all chains. Instead, in the Results section we highlight the diversity associations that show the highest consistency across chains. Nonetheless, the reviewer is right that the associations detected at the phenotype level are relatively weak compared to those identified in the case-control analysis. Accordingly, we have now softened the statement that we initially made to present our phenome-wide diversity results (pages 4 and 5).*

*Finally, the low diversity of the AIRR of RA patients is one of the most strongly supported statements of the manuscript (Fig 1b shows the strong statistical significance of the BCR compartment). The use of different statistical tests used here to support our claim is not only acceptable, but also the most rigorous strategy to deal with several diversity measures that follow a completely different distribution (e.g., D50 and Shannon entropy follow a negative binomial and normal distributions, respectively). In order to avoid any confusion, we have now rewritten and focused the diversity results on D50, one of the most commonly used diversity metrics in AIRR-seq studies (pages 4 and 5).*

4. If I understand correctly the methodology developed in Puelma Touzel et al (2020) is used to identify "significantly" contracted and expanded clones. This methodology was designed for full TCR sequences, but is used here for CDR3-AA-clones, including in the immunoglobulin locus. I have checked with the authors of this method and they are confirmed that neither using CDR3-AA sequences nor BCR sequences correspond to the assumptions made in the development of their method. Using CDR3-AA-clones might be OK, but to decide if this is the case one would need replicate measurements to calibrate the sensitivity of the method. However, the method definitely does not extend in a simple way to the BCR locus, because of the process of affinity maturation. They are working on an extension to BCRs but this is going to take time and careful consideration. I believe that these methodological issues drive the extraordinary number of expansions and contractions seen in the IGH, IGK, and IGL loci, and I don't feel clear that these are meaningful.

*Response: The reviewer is right that NoisET method was designed to identify contracted and expanded TCR clones. However, it is not correct that we have implemented this Bayesian methodology to analyze CDR3 amino acid sequences. As we described in the Methods section (pg. 20), we used the CDR3 nucleotide sequence of each clone to infer the noise model in NoisET that allows to identify contracted and expanded clones in an unbiased manner. Importantly, following the method’s guidelines, we had also incorporated AIRR-seq information from sample replicates in order to account for the variability in read counts that is due to experimental and technical noise (pg. 20).*

*Unlike TCR, BCR is submitted to affinity maturation and, as raised by the reviewer, it could impact on the accuracy of the identification of expanded and contracted clones. Prof. Walczak (developer of NoisET and principal investigator of Laboratoire de Physique de l’École Normale Supérieure, University of Paris, France) (Puelma Touzel et al., 2020), guided us on the implementation of NoisET to analyze BCR clones. In particular, Prof. Walczak raised the possibility that applying NoisET to BCR and TCR sequences can be equally valid if BCR clones are not highly hypermutated. Since we have found that only a very small fraction of the BCR clones is hypermutated (e.g., <3.75% across chains; Figure 6c), it is unlikely that it had a major impact on the BCR longitudinal analysis. Moreover, it has been demonstrated that, by modelling technical noise, this probabilistic approach overcomes the power of the methods previously used to detect clonal frequency differences like Fisher’s exact test (Emerson et al., 2017; Puelma Touzel et al., 2020). We are therefore convinced that NoisET is the most optimal approach available so far to identify expanded and contracted clones at the TCR, but also at the BCR level. It is nonetheless true that we cannot discard the existence of a small fraction of BCR clones that do not meet the NoisET assumptions and, consequently, the expansion/contraction estimates could be inaccurate. We have consequently now stated this possibility in the Discussion section of the manuscript, so that the results can be interpreted considering this technical aspect raised by the reviewer (pg. 27).*

*5. It seems like there is additional relevant work, such as https://www.sciencedirect.com/science/article/abs/pii/S0896841120300457 and https://www.jrheum.org/content/45/7/905.*

*Response: The additional work raised by the reviewer is relevant to better understanding the association of TCR diversity with RA and, therefore, we agree that it deserves to be mentioned in the manuscript. We apologize for not having included these two scientific publications in the original manuscript. Accordingly, we have now incorporated both articles in the revised version of the manuscript (pages 16, 26 and 27).*

6. The authors claim that the sequencing procedure is unbiased. That is a strong claim-- even getting a single chain assay to be unbiased is quite difficult. Reference 30, which is cited, does not claim that the sequencing process is unbiased. Is there further evidence of unbiasedness? If not, how is this addressed?

*Response: The essentially bias-free accuracy of the iR-RepSeq+ 7-Chain technology is achieved by addressing every source of possible errors in AIRR-seq experiments in accordance to the state-of-the-art practice guidelines described by the AIRR community (Trück et al., 2021). In the present study, we have generated quantitative and essentially bias-free repertoire information by incorporating into the high-throughput sequencing workflow the following technical measures: i) V-C primer-specific PCR amplification was not included in our PCR-based library preparation to eliminate a biased template choice, ii) The library generation process was designed to be fully automatic, thereby removing any potential source of human error and sample cross-contamination, iii) UMI barcoding system was used to remove any enzyme and/or sequencing error, iv) a set of complex parallel biological control samples was used with every plate to reveal any possible batch effect, v) comparison of replicate amplifications was performed for every sample-replicate pair to ensure reproducibility, and vi) cellular spike-in controls at known concentrations and control individual chain mixtures were additionally used by iRepertoire, Inc (iRepertoire, US) for benchmarking of the method. Although the quantitative repertoire information generated from our samples is as unbiased as it can be technically achieved, we agree with the reviewer that the claim is too strong. Accordingly, we have now softened the claim in the revised version of the manuscript (pages 3, 4 and 14).*

7. The biggest effect of RA is a reduction of diversity in the IG loci. The current paper can only say that this is a reduction of diversity on the level of clones, whereas more detailed analyses (e.g. Elliott 2020 https://www.sciencedirect.com/science/article/pii/ S1521661619306448) find that this is due to expansion of clonal families, and can understand the responsive clones and what is driving their expansion. This includes functional characterization and response to citrullinated antigens.

*Response: The reviewer is right that the strongest effect of the disease in the repertoire diversity is restricted to the B cell compartment. It is important to underscore that this effect was detected in our study by contrasting RA patients and controls. In Elliot et al, the diversity analysis was conducted only in samples of RA patients, with no contrast to healthy individuals. In particular, the authors showed a reduction on the diversity of the IGH-IGL repertoire in synovial tissue compared to peripheral blood. In our study not only we have demonstrated that BCR diversity is reduced in the peripheral blood of RA compared to controls, but also that therapeutic inhibition of TNF is able to restore this low diversity towards that of healthy (homeostatic) level. Furthermore, the strongest therapeutic effect was detected in the IGK, a BCR chain that was not analyzed by Elliott and colleagues. Therefore, our study design is clearly superior in terms of repertoire chain diversity analysis as well as the analysis of the longitudinal changes in relation to therapy.*

*Elliott and colleagues show the existence of clonal BCR expansions that are shared between peripheral blood and synovial tissue. Importantly, this finding demonstrating B cell trafficking between both compartments supports our hypothesis that IGL-harboring B cells migrate to the target tissue of RA. We have now provided more details on our hypothesis resulting from the differential chain usage analysis (pg. 25). In addition, we agree with the reviewer that the functional approach conducted by Elliott and colleagues to characterize the expanded clones is of major interest. Accordingly, we have used this relevant work as a reference to suggest how RA-associated IGL and IGK clones could be characterized at the functional level (pg. 27).*

8. The "clinical level" conclusions don't appear to be significant when accounting for multiple hypothesis testing. The TNFi results in Figure 1d appear to be robust but corrected p-values should be reported. For Figure 1e, there is a sample size issue here: there are substantially fewer non-responders, which looks to me to be driving the difference.

*Response: We agree with the reviewer that differences in immune repertoire diversity between clinical phenotypes are moderate in comparison to those detected in the case-control analysis. Also, we thank the reviewer for appreciating the robustness of the results obtained in the longitudinal diversity analysis that are represented in Fig. 1d. In addition to the p-value correction aspect (discussed in response to comment #3), the sample size issue raised by the reviewer is also of relevance.*

*As reported in many previous studies, response to TNF inhibition occurs in 60-75% of patients (Kievit W et al., 2008). Therefore, prospective cohorts will always show an unbalanced representation of each response class. As we show in Fig. 1e, it is unlikely that the lower sample size of non-responders has impacted on the diversity differences detected in IGH. In IGK and IGL, however, we cannot discard the sample size as one of the factors underlying the non-significant differences detected between responders and non-responders. As we have now included in the revised version of the manuscript (pages 5 and 24), the collection, follow-up and immunosequencing of other cohorts of RA patients treated with TNF inhibition will provide independent corroboration of the present findings. In particular, immunosequencing analysis of larger patient cohorts could provide more statistically significant associations, albeit with smaller effect sizes. If this was the case, the immune repertoire would not be powerful enough for robust prediction of TNF inhibition response by itself and, instead, it should be used in combination with other relevant molecular or clinical factors of the patient.*

9. I don't understand the right two columns in the Figure 1b/c heatmaps. Are the bubbles meaningful? If so, please describe. Please describe the colors in these heatmaps a little more. I see they are "effect size": does this mean the ratio of the statistic in the *two conditions? What was the statistical test done? If a randomization test, I am not sure what is being randomized.*

*Response: In line with the graphical strategy used by Bashford-Rogers et al., we have used bubbles to graphically illustrate the association results at the chain level and, in turn, facilitate the interpretation of our findings. We apologize for not having presented this type of representation clearly enough. To avoid any confusion, we have now clarified this aspect in the legend of Figure 1 (pg. 31).*

*As raised by the reviewer, the heatmaps shown in Figure 2 b/c are colored according to the effect size of the association between each diversity measure and the indicated trait (i.e., RA and Week 12 of TNF inhibition in Figure 2b and 2c, respectively). This effect size corresponds to the estimate retrieved from the statistical model used for testing each association. For example, a negative binomial regression model was used to test the association between D50 and RA. The exact effect sizes together with the reference group for referring the effect direction (Table S3 and S5) and the statistical models used to conduct the diversity analysis (Table S28) are provided in Supplementary Material. We have now clarified this aspect in the legend of Figure 2 (pages 31 and 32).*

10. P4L37: "all subsequent AIRR analyses were adjusted for the sequencing depth as well as for sex and age." Is this really all analyses? I did read about how these variables are included as covariates, but does that impact the diversity analyses, e.g.?

*Response: We confirm that all analyses included in the present manuscript were adjusted for sex, age and sequencing depth. Our thorough analysis of the impact that technical and epidemiological variables in AIRR clearly differentiates our work from previous analyses of the AIRR, and it is therefore one of the key strengths of our study (Emerson et al., 2017; Puelma Touzel et al., 2020). In order to show the impact that each of these variables has on the AIRR measures here analyzed, we generated Figure S1. For example, in relation to the diversity analysis raised by the reviewer, Figure S1 clearly shows how sequencing depth is strongly correlated with D50 estimation in TRB chain (r²=0.58). Therefore, it was considered essential to adjust for this technical variable to minimize its possible confounding effect. Age and sex showed also significant associations to some AIRR measures (e.g., gender association with isotype percentage, P=0.028, Figure S1). Despite these associations were of small magnitude, we decided to adjust also for sex and age as a conservative analytical strategy.*

11. What is the scale of the "significance" in Figure 2c? Log p value?

*Response: We confirm that the statistical significance that we show in Figure 2c is represented in the negative logarithmic scale. We apologize for not having described it clearly in the “Figure legends” section. We have now added this information in the legend of Figure 2 (pages 31 and 32).*

12. p6L13-14: this p-value is for the properties being shared? How are these being computed?

*Response: The p-value indicated in p6L13-14 refers to the low molecular weight, a molecular property that is shared by contracted and expanded clones. In order to determine whether a given set of CDR3 amino acid sequences (e.g., contracted/expanded clones) are characterized by a particular biochemical property, we have used a resampling strategy. Using this analytical approach, the statistical significance of the biochemical property was computed by contrasting the observed metric in a given set of clones against the null distribution obtained after sampling >1,000 times a random set of clones from the same size and receptor chain. After obtaining the statistical significance of enrichment and depletion in a particular biochemical feature, both statistics were log-transformed and subsequently aggregated into a single biochemical enrichment score (BES). The details of this analytical strategy are provided in page 22.*

13. Figure 2a-- the colors are so light, so that I can't tell if I can see IGH. Perhaps see https://clauswilke.com/dataviz/color-basics.html#color-as-a-tool-to-distinguish

*Response: In accordance with the reviewer’s observation, we have generated a new version of Figure 2a and Figure 2b so that BCR chains are now totally distinguishable (Figure 2).*

14. Figure 2e-- what is up with the contracted responders? Why are there concentric pie charts?

*Response: In Figure 2e, we used IGL to exemplify the behavior observed in all seven chains that contracted and expanded clones have a higher sequence similarity than expected by chance in both responders and non-responders to anti-TNF therapy. The graphical representation for the six remaining chains can be accessed through the website of the Rheumatology Research Group (www.urr.cat/AIRR-RA; FOR REVIEWERS ONLY → username: ReviewerAIRR.RA / password: tcrbcr.2021). The details of the analytical approach used to determine if a given set of clones have a higher sequence similarity than expected by chance are described in “Comparison against a random repertoire” section and the statistics resulting from this analysis are provided in Table S9.*

*The graphical characteristics of nodes defining all the similarity networks of expanded and contracted clones were set by the Kamanda-Kawai layout algorithm implemented in the igraph R package. Therefore, concentric pie charts were displayed on the plane in accordance to the physical model of springs that is used by the Kamanda-Kawai layout algorithm. In order to clarify how clone similarity networks were generated, we have now added this methodological information in “Amino acid sequence similarity” section (pg. 21).*

15. P7L7: The IGK has much higher expression in the synovium than IGL. How does that fit into this analysis?

*Response: As the reviewer indicates, unlike IGL, blood expression of IGK chain was not found to be different between RA patients and healthy individuals. Therefore, our results do not support that the increased expression of IGK chains detected in the synovium is due to increased migration of B cell clones from blood to the target tissue of RA. Instead, this result supports a local expansion of IGK-harboring B cells in the synovial tissue. This is consistent with the development of functional germinal center-like structures with antibody-producing plasmablasts that is characteristic of the inflamed synovial tissue in RA (Humby et al., 2019). This interpretation of our findings has been now added in the discussion of the revised manuscript (pg. 25). As raised by Reviewer #1, an analysis of correlation between the synovial expression of IGK and plasmablast cell number could help to validate our hypothesis. However, the cell counts of plasmablasts were not available from synovial tissues or blood samples analyzed in this previous dataset (Lewis et al., 2019).*

16. P8L17: Can you provide more details on this "random repertoire"? It is sampled from the (observed?) clones, with uniform weight on each? Does this really represent a reasonable null?

*Response: In the Methods section of the original manuscript (pg. 22), we describe that a random repertoire was generated by sampling >1,000 times a random set of clones from the same chain and size as a group of clones of interest (e.g., contracted IGH clones). However, we agree with the reviewer that this information could be insufficient to understand the approach. We also think that the first time that “random repertoire” is introduced -P8L17 highlighted by the reviewer- might be imprecise. Following the reviewer’s indications, we have now extended its description the first time that it is cited in the manuscript (pg. 8) and, additionally, we have added more technical details in the Methods section (pg. 22).*

*The reviewer raises also an important methodological aspect that we took into account when designing the approach to generate the null distribution. In the present work, the random set of clones that we used to build a random repertoire was sampled from the observed clones, and the probability of being sampled was equally assigned to each one. To our knowledge, this work represents the first AIRR-seq study that incorporates a statistical framework (See Figure 1 in ResponsesToReviewers document updated as Personal Cover) to characterize a group of clones at the biochemical level. In line with the reviewer’s comment, future methods specifically devised for this purpose could improve the performance of our approach. Nevertheless, we are convinced that the analytical strategy designed and implemented here lead to a robust null. To clarify the technical aspects underlying the clone sampling methodology, we have now added this information in the Methods section of the revised manuscript (pg. 22).*

*Figure 1. Graphical representation of the statistical analysis that we implemented to characterize a group of clones of interest.*

17. Figure 3c-e: could we have some labels on these figures, like V gene, etc? If I understand correctly, all of the different V genes on the different chains are all put together in the same PC plot. Thus, we could have the same proportional use of the germline genes, but different frequency of chains in the data, and get a striking effect. Could that be happening here? In any case, if my interpretation is correct, then one should reframe the analysis or change it to be only about germline gene usage. This is also true of the association with clinical phenotype in the next section.

*Response: The information required was initially provided in Figure 3 legend. We have nonetheless generated a new version of Figure 3 in order to incorporate the reviewer’s suggestion in the revised manuscript.*

*The interpretation of the reviewer that all gene segments from different chains are put together in the same plot is partially accurate. After performing principal component analysis, here we represent the first two principal components (i.e., PC1 and PC2) and, therefore, only those gene segments having the strongest effect on phenotypic variation are contributing to the observed profile. Furthermore, the gene segment information used to generate gene usage profiles is independent of chain frequency. More specifically, the usage of each V, J and VJ segment pair was computed as its frequency in clones within each chain at the individual level. In the phenotypic analysis that showed the strongest gene usage differences (i.e., case-control), we have checked that the top contributors to phenotypic variability are gene segments from TRA, TRB and IGK (Table S11), three chains with highly similar expression levels (Figure 3). Hence, we can confirm that the scenario suggested by the reviewer is not happening in the analysis presented here.*

18. CDR3 length analysis and supplementary figure 7: Many of these distributions look nearly identical yet get extraordinary p-values (1e-136!). If we reshuffle cases between RA and control, do we get significant p-values? If so, perhaps it's time to reconsider how the statistical analysis is done. The TRD distribution does look different, but biggest difference is a huge spike at 18 amino acids in the control group. Is that worth examining more closely?

*Response: The reviewer is right that, with the present graphic, it is difficult to visualize the differences between patients and controls in the CDR3 amino acid length distribution that led to the strong statistical significance. It is important to take into account that in this analysis what we tested was the null hypothesis that the two distributions are identical and that there is no significant shift between them. Accordingly, the Wilcoxon test conducted here compared the distribution originated by the length of 2,066,414 clones in RA against the length of 2,324,291 clones detected in controls. Consequently, these large sample sizes allow the detection of very subtle distribution shifts. Based on the reviewer observation, we have now compared the clone length distributions unweighting the analysis by clone abundance (N.cases=1,327,101 clones; N.controls=1,505,606 clones). As we show in Figure 2 (See Figure 1 in ResponsesToReviewers document updated as Personal Cover), the statistical significance still remains very strong (P=6.13e-51), but not as strong as the original analysis (P=1.25e-136).*

*We have further reshuffled RA and controls as indicated by the reviewer. Given the high computational cost of this additional analysis, reshuffling has been performed 150 times. Importantly, a statistical significance lower than the observed (P=1.25e-136) was only detected in 7 out of the 150 analyses performed here, being >1e-50 in >70% of the comparisons. These results clearly indicate that our statistical approach is appropriate to detect shifts between two clone length distributions.*

*Finally, we thank the reviewer for raising that the biggest difference between RA and controls at TRD chain is driven by a clone length of 18 amino acids. This result should definitively be studied in additional functional analyses, but we consider it is out of the scope of the current work. We agree with the reviewer that the length difference of specifically 18 amino acids is of high interest and should be underscored in the manuscript. Accordingly, we have now added this observation in the Results section of the revised version of the manuscript (pg. 10).*

19. P10L54: this analysis wouldn't survive multiple testing correction.

*Response: The reviewer is right that the association of IgE-IgG switching with RA is significant at the nominal level, but it is not significant after multiple testing correction. We are nonetheless convinced that this secondary finding deserves to be mentioned because it is totally in line with the work of Bashford-Rogers et al., the most comparable BCR analysis performed so far in autoimmunity. In this study, an aberrant IgE-IgG class switching was also detected in autoimmune diseases like SLE and CD. IgG but not IgE isotypes are essential to activate the complement pathway and, therefore, the association here detected -albeit nominal- is consistent with the pathologic association of this innate immune response mechanism in RA (Trouw et al., 2017). Following the reviewer’s suggestion, in the revised version of the manuscript we have emphasized the nominal nature of the association, and the need for replication of the finding in independent case-control cohorts (pages 11 and 26).*

20. P11-- Please describe the hold-out strategy in more detail. The accuracy reported is on the held-out set?

*Response: Following the reviewer’s indication, we have now extended the description of the leave one out cross validation strategy that we used to estimate the accuracy of our multi-chain AIRR predictor (pg. 23).*

*The accuracy of our machine learning classification algorithm is based on the number of correct predictions observed after applying the leave one out cross validation strategy. We have also clarified this aspect in the revised version of the manuscript (pg. 23).*

21. The supplementary material must be downloaded from the authors' web site, which I don't think is technically allowable for peer review. Zenodo or some such service allows for anonymous download.

*Response: In order to facilitate the review of the supplementary material without accessing the author’s web site, we have now uploaded two independent folders (i.e., Supplementary Figures and Supplementary Tables) together with the revised version of the manuscript.*

Reviewer #3

In the manuscript "Seven chain adaptive immune receptor repertoire analysis in rheumatoid arthritis: association to disease and clinically relevant phenotypes", the authors perform a comprehensive analysis of the BCR and TCR repertoires in RA patients before and after TNFi treatment, and compared with a baseline of healthy individuals. They find that in RA patients the diversity of the BCR repertoire is reduced and other features are skewed. TNFi treatment partially normalizes the repertoires in individuals, which is correlated with clinical response to the treatment. The work is clearly presented, but I have some very fundamental methodological concerns that in my opinion prevent this manuscript from being accepted in its current form.

*Response: We would like to thank the reviewer for the positive evaluation of the presentation of our manuscript. We herein include a point-by-point response to each of the reviewer’s specific points. We are convinced that our revised manuscript has significantly improved the quality of the original version.*

1. Especially regarding figure 3 C, D, E and also several other figures- the differences between the patients and the healthy controls seem almost too good to be true… Such clear differences in V J usage have never been previously properly reported, and when something like that was published it was never validated. This raises a strong concern for batch effects. Did the authors sequence the patients and healthy repertoires together or separated? Were the libraries prepared simultaneously for all groups? There is no info on any quality control steps that were taken.

*Response: The present study is the first to perform gene usage analysis in RA at two different levels: i) comparing the usage of each individual segment in the seven immune receptor chains to identify chain-specific associations, and ii) integrating multi-chain usage information into a principal component analysis to discover the gene usage profile that underlies each clinical phenotype. As the reviewer indicates, we have found very high gene usage differences between cases and controls, particularly when comparing the usage of the VJ segment pairs (Figure 3E). However, in a case-control analysis of the TRB chain immunosequenced in individuals from the Chinese population (Liu et al., 2019), very significant differences between RA patients and controls were also detected. Furthermore, many of the TRB-V usage associations in this Asian population study have been validated in the present AIRR-seq study (e.g., V20-1, V6-1, V7-8 with FDR<0.05, Table S11). Taken together, our results provide strong evidence that gene usage in RA is very distinct from that of healthy individuals.*

*We agree with the reviewer that strong differences may raise questions of potential technical issues biasing the results. However, our study was designed to minimize any possible source of bias. The library preparation for immunorepertoire sequencing was conducted by coauthors of this study and members of iRepertoire, Inc. (Huntsville, US). This company has pioneered the development of immune repertoire sequencing technologies and products. As such, we are highly aware of all the potential biases that can affect this type of analysis and, consequently, we took special care to minimize the introduction of any batch effect. As the reviewer suggests, separate analysis of cases and controls can be a major factor of confounding. To avoid this, we evenly distributed control and patient samples across the sequencing plates. Furthermore, within each sequencing plate, samples were randomized to reduce the possibility of location biases. Each sequencing plate included 80 samples that were simultaneously amplified and sequenced on the same run using the Illumina NovaSeq platform. This is an important technical aspect and quality control measure of the study. In order to clarify this, we have generated a new supplementary table as part of the original Table S2 (pages 4, 15 and 41) showing the sample distribution across sequencing plates (i.e., 15 control and 24 patient samples of varying clinical response per sequencing plate) as well as the RNA integrity and concentration of each sample. Together with Fig S1, this data clearly shows that the potential confounding effects of plate and/or sample integrity are efficaciously corrected when adjusting for sequencing depth.*

2. There is no systematic investigation towards the source of these huge differences. Is it T cells? B cells? Is it the ratio between CD4 and CD8 that was not discussed?

*Response: The reviewer is right that we have not explicitly shown whether the 34 V segments, 12 J segments and 127 V-J pairs that were found to be significantly different between RA and control subjects (FDR<0.05, Table S11) are from T or B cells. Although the use of PBMC did not allow us to discriminate between T cell types like CD4 or CD8, we can determine whether the huge differences of gene usage profile that distinguish RA patients from controls are mainly driven by gene segments from T or B cells. For this objective, we have now added in the “Results” section of the revised version of the manuscript that gene usage differences between cases and controls are mainly driven by gene segments from T cells (pg. 7).*

3. What is the hypothesis behind these differences?

*Response: The strongest differences mentioned by the reviewer are mainly driven by IGKV2-28, TRAV20, IGLV3-21 and TRBV20-1 (FDR<3.5e-06) at the V segment level, as well as by IGKV2-28/IGKJ1, IGKV2-28/IGKJ4 and TRBV20-1/IGKJ2-5 (FDR<2e-06) at the VJ level (Table S11). In both cases, the top signal was found to involve V2-28 segment from IGK. Not surprisingly, this AIRR feature was the most important predictor variable in our disease association model. IGKV2-28 segment has been previously found associated to the generation of autoantibodies in systemic lupus erythematosus (al Kindi et al., 2015). Taken together, these results support the importance of this gene segment in autoimmune diseases with a key B cell component.*

*Given that V(D)J rearrangement determines the CDR3 sequence and therefore the recognition of antigenic peptides (Fischer et al., 2020; Rossjohn et al., 2015), our hypothesis is that IGK antibodies harboring V2-28 segment are autoreactive and accumulate in RA patients because of an altered DNA recombination process that, consequently, increases the autoreactivity burden of the patient. We have now added our hypothesis in the revised version of the manuscript (pg. 26).*

4. What is the mechanism behind the clonal selectivity of the TNFi treatment? This point is strange to me, as this is a general cytokine.

*Response: The bigger increase in BCR diversity observed in responders was found to be mainly driven by the reduction of clones with a specific biochemical profile. These novel findings suggest that responders and non-responders to TNFi therapy represent two classes of patients that are characterized by the expansion of different B cell populations that react against different autoantigens. Consistently, there is evidence that activated B cells can be polarized into different subtypes that produce proinflammatory cytokines other than TNF (Shen et al. 2015). This is also in line with our recent discovery that anti-TNF response is associated to synovium levels of peripheral helper T cells, a pathogenic subtype that helps B cell activation through TNF-independent mechanisms (Julià et al., 2020). According to our mechanism, a downstream effect of the autoantibodies produced by distinct B cell populations in responders and non-responders is the activation of different macrophage subtypes. Importantly, macrophage heterogeneity has been also associated with TNF inhibition response (Haringman et al., 2005; Kuo et al., 2019; Onuora, 2018). Therefore, these previous evidences indicate that BCR diversity is likely to increase, after TNF inhibition, specifically in patients who have a strong burden of activated TNF-dependent mechanisms. It is in this group of patients where TNF blocking would be more effective at disrupting the pro-inflammatory cascade. According to our findings, this is the group of patients that showed a significant clinical improvement. In order to clarify the suggested mechanism for TNF inhibition selectivity, we have now rephrased the 3rd paragraph of the Discussion section in the revised manuscript (pg. 25).*

5. Methods sections indicate that the analysis was not careful enough. For example, the use of MiXCR for BCR is not adequate for such analysis. How was the alignment performed? Which reference set? Etc.

*Response: We do not agree with the reviewer’s comment that the analysis was not careful enough. We have applied MiXCR to both AIRR-seq (pg. 14) and RNA-seq (pg. 21). However, in our previous manuscript version most of the analytical details were only provided for the AIRR-seq data processing (pages 14 and 15). We think that this could have generated confusion and we have now clarified this in the present version of the manuscript.*

*The reviewer is right that the details of the alignment were not included in the original manuscript and we apologize for that. Accordingly, we have now included this technical information (pg. 14).*

6. The definition of meta-clones is not clear. What does "high similarity" mean? These details must be described carefully in a way that the results can be reproduced.

*Response: We agree with the reviewer that the meta-clone definition might not have been described clearly enough. We have now rewritten this section to improve clarity and facilitate the reproducibility of our analyses (pages 9 and 15). High similarity, in particular, refers to communities of clones differing by at most one amino acid in the CDR3 sequence. These communities are identified by optimizing a modularity score with the cluster.fast.greedy function (igraph R package) within the clone sequence similarity network that was built using the mutation.network function (tcR R package).*

7. Page 7 first paragraph, says that there is a shift from IGL to IGK expression in the blood of RA patients, and the authors try to explain this by migration of the IGL harboring B cells from the blood to the synovium. There is indeed an increase in IGL expression in the synovium samples (figure 3B), but an even stronger increase in IGK, so the hypothesis cannot be correct. It also seems unlikely that the increase in IGK is caused by the general increase in the blood, since the increase in the blood is very weak and not statistically significant.

*Response: Here, we would like to clarify that we do not interpret our result as a shift of expression from IGL to IGK. In blood, significant expression differences between cases and controls were uniquely detected for IGL and, consequently, we hypothesized that it could be due to an increased migration of IGL-harboring B cells from blood to synovial tissue. This hypothesis was supported by the analysis of paired blood and synovial samples.*

*In the case of the IGK increase detected in the synovium, this was not reflected as a downregulation in the circulating levels. There could be many possible reasons for this observation. Instead of new B cell clones infiltrating the disease target tissue, one possible explanation for the higher expression of IGK detected in synovial tissue compared to blood could be the local expansion of IGK-harboring B cells. In RA, functional germinal center-like structures (e.g., tertiary lymphoid structures) have been shown to be present in the inflamed synovial tissue (Humby et al., 2019). These B cell activation centers have been shown to expand ACPA-producing plasmablasts. For this reason, the increased expression of IGK detected in the synovial tissue does not invalidate our migration hypothesis for IGL-harboring B cells. Although the IGK expression differences raised by the reviewer are a secondary finding detected when testing our IGL migration hypothesis for validation, these differences are significant and deserve to be mentioned in the manuscript. Accordingly, we have now commented on this finding in the Discussion section of the revised manuscript (pg. 25).*

*REFERENCES*

*Al Kindi, M. A., Chataway, T. K., Gilada, G. A., Jackson, M. W., Goldblatt, F. M., Walker, J. G., Colella, A. D., & Gordon, T. P. (2015). Serum SmD autoantibody proteomes are clonally restricted and share variable-region peptides. Journal of Autoimmunity, 57, 77–81.*

*Bank, I. (n.d.). cells The Role of Gamma Delta T Cells in Autoimmune Rheumatic Diseases.*

*Bashford-Rogers, R. J. M., Bergamaschi, L., McKinney, E. F., Pombal, D. C., Mescia, F., Lee, J. C., Thomas, D. C., Flint, S. M., Kellam, P., Jayne, D. R. W., Lyons, P. A., & Smith, K. G. C. (2019). Analysis of the B cell receptor repertoire in six immune-mediated diseases. Nature 2019 574:7776, 574(7776), 122–126.*

*Calis, J. J. A., & Rosenberg, B. R. (2014). Characterizing immune repertoires by high throughput sequencing: Strategies and applications. In Trends in Immunology (Vol. 35, Issue 12, pp. 581–590). Elsevier Ltd.*

*Elliott, S. E., Kongpachith, S., Lingampalli, N., Adamska, J. Z., Cannon, B. J., Blum, L. K., Bloom, M. S., Henkel, M., McGeachy, M. J., Moreland, L. W., & Robinson, W. H. (2020). B cells in rheumatoid arthritis synovial tissues encode focused antibody repertoires that include antibodies that stimulate macrophage TNF-α production. Clinical Immunology, 212.*

*Emerson, R. O., DeWitt, W. S., Vignali, M., Gravley, J., Hu, J. K., Osborne, E. J., Desmarais, C., Klinger, M., Carlson, C. S., Hansen, J. A., Rieder, M., & Robins, H. S. (2017). Immunosequencing identifies signatures of cytomegalovirus exposure history and HLA-mediated effects on the T cell repertoire. Nature Genetics 2017 49:5, 49(5), 659–665.*

*Fischer, D. S., Wu, Y., Schubert, B., & Theis, F. J. (2020). Predicting antigen specificity of single T cells based on TCR CDR 3 regions. Molecular Systems Biology, 16(8).*

*Han, J., & Lotze, M. T. (2020). The Adaptome as Biomarker for Assessing Cancer Immunity and Immunotherapy. Methods in Molecular Biology, 2055, 369–397.*

*Haringman, J. J., Gerlag, D. M., Zwinderman, A. H., Smeets, T. J. M., Kraan, M. C., Baeten, D., McInnes, I. B., Bresnihan, B., & Tak, P. P. (2005). Synovial tissue macrophages: a sensitive biomarker for response to treatment in patients with rheumatoid arthritis. Annals of the Rheumatic Diseases, 64(6), 834–838.*

*Holoshitz, J. (1999). Activation of γδ T cells by mycobacterial antigens in rheumatoid arthritis. Microbes and Infection. 1(3):197-202*

*Humby, F., Bombardieri, M., Manzo, A., Kelly, S., Blades, M.C., Kirkham, B., Spencer, J., Pitzalis, C. (2009). Ectopic lymphoid structures support ongoing production of class-switched autoantibodies in rheumatoid synovium. PLoS Medicine*

*Jiang, X., Wang, S., Zhou, C., Wu, J., Jiao, Y., Lin, L., Lu, X., Yang, B., Zhang, W., Xiao, X., Li, Y., Wu, X., Wang, X., Chen, H., Zhao, L., Fei, Y., Yang, H., Zhang, W., Zhang, F., … Zhang, X. (2020). Comprehensive TCR repertoire analysis of CD4+ T-cell subsets in rheumatoid arthritis. Journal of Autoimmunity, 109.*

*Julià, A., Ávila, G., Celis, R., Sanmartí, R., Ramírez, J., Marsal, S., Cañete, JD. (2020). Lower peripheral helper T cell levels in the synovium are associated with a better response to anti-TNF therapy in rheumatoid arthritis. Arthritis Research & Therapy. 22(1):196*

*Kaplinsky, J., & Arnaout, R. (2016). Robust estimates of overall immune-repertoire diversity from high-throughput measurements on samples. Nature Communications 2016 7:1, 7(1), 1–10.*

*Kievit, W., Adang, EM., Fransen, J., Kuper, HH., Van der Laar, MAFJ., Jansen, TL., De Gendt, CMA., De Rooij, D-JRAM., Brus, HLM., Van Oijen, PCM., Van Riel, PCLM. (2008). The effectiveness and medication costs of three anti-tumour necrosis factor alpha agents in the treatment of rheumatoid arthritis from prospective clinical practice data. Annals of the Rheumatic Diseases, 67(9):1229-34.*

*Kissel, T., Reijm, S., Slot, L. M., Cavallari, M., Wortel, C. M., Vergroesen, R. D., Stoeken-Rijsbergen, G., Kwekkeboom, J. C., Kampstra, A. S. B., Levarht, E. W. N., Drijfhout, J. W., Bang, H., Bonger, K. M., Janssen, G. M. C., van Veelen, P. A., Huizinga, T. W. J., Scherer, H. U., Reth, M., & Toes, R. E. M. (2020). Antibodies and B cells recognising citrullinated proteins display a broad cross-reactivity towards other post-translational modifications. Annals of the Rheumatic Diseases, 79(4), 472–480.*

*Kuo, D., Ding, J., Cohn, I. S., Zhang, F., Wei, K., Rao, D. A., Rozo, C., Sokhi, U. K., Shanaj, S., Oliver, D. J., Echeverria, A. P., DiCarlo, E. F., Brenner, M. B., Bykerk, V. P., Goodman, S. M., Raychaudhuri, S., Rätsch, G., Ivashkiv, L. B., & Donlin, L. T. (2019). HBEGF+ macrophages in rheumatoid arthritis induce fibroblast invasiveness. Science Translational Medicine, 11(491).*

*Lewis, M. J., Barnes, M. R., Blighe, K., Goldmann, K., Rana, S., Hackney, J. A., Ramamoorthi, N., John, C. R., Watson, D. S., Kummerfeld, S. K., Hands, R., Riahi, S., Rocher-Ros, V., Rivellese, F., Humby, F., Kelly, S., Bombardieri, M., Ng, N., DiCicco, M., Pitzalis, C. (2019). Molecular Portraits of Early Rheumatoid Arthritis Identify Clinical and Treatment Response Phenotypes. Cell Reports, 28(9), 2455.*

*Liu, X., Zhang, W., Zhao, M., Fu, L., Liu, L., Wu, J., Luo, S., Wang, L., Wang, Z., Lin, L., Liu, Y., Wang, S., Yang, Y., Luo, L., Jiang, J., Wang, X., Tan, Y., Li, T., Zhu, B., … Lu, Q. (2019). T cell receptor β repertoires as novel diagnostic markers for systemic lupus erythematosus and rheumatoid arthritis. Annals of the Rheumatic Diseases, 78(8), 1070–1078.*

*Minervina, A. A., Komech, E. A., Titov, A., Koraichi, M. B., Rosati, E., Mamedov, I. Z., Franke, A., Efimov, G. A., Chudakov, D. M., Mora, T., Walczak, A. M., Lebedev, Y. B., & Pogorelyy, M. v. (2021). Longitudinal high-throughput tcr repertoire profiling reveals the dynamics of t-cell memory formation after mild covid-19 infection. ELife, 10, 1–17.*

*Niu, X., Li, S., Li, P., Pan, W., Wang, Q., Feng, Y., Mo, X., Yan, Q., Ye, X., Luo, J., Qu, L., Weber, D., Byrne-Steele, M. L., Wang, Z., Yu, F., Li, F., Myers, R. M., Lotze, M. T., Zhong, N., … Chen, L. (2020). Longitudinal Analysis of T and B Cell Receptor Repertoire Transcripts Reveal Dynamic Immune Response in COVID-19 Patients. Frontiers in Immunology, 11.*

*Onuora, S. (2018). Anti-TNF kills the macrophage response. Nature Reviews Rheumatology 2018 14:2, 14(2), 64–64.*

*Pappas, DA., St John, G., Etzel, CJ., et al (2021). Comparative effectiveness of first-line tumour necrosis factor inhibitor versus non-tumour necrosis factor inhibitor biologics and targeted synthetic agents in patients with rheumatoid arthritis: results from a large US registry study. Ann Rheum Dis 2021; 80: 96–102*

*Paul, S., & Lal, G. (2015). Role of gamma-delta (gd) T cells in autoimmunity. J. Leukoc. Biol, 97, 259–271.*

*Pollastro, S., Klarenbeek, P. L., Doorenspleet, M. E., van Schaik, B. D. C., Esveldt, R. E. E., Thurlings, R. M., Boumans, M. J. H., Gerlag, D. M., Tak, P. P., Vos, K., Baas, F., van Kampen, A. H. C., & de Vries, N. (2019). Non-response to rituximab therapy in rheumatoid arthritis is associated with incomplete disruption of the B cell receptor repertoire. Annals of the Rheumatic Diseases, 78(10), 1339–1345.*

*Puelma Touzel, M., Walczak, A. M., & Moraid, T. (2020). Inferring the immune response from repertoire sequencing. PLoS Computational Biology*

*Rossjohn, J., Gras, S., Miles, J. J., Turner, S. J., Godfrey, D. I., & McCluskey, J. (2015). T cell antigen receptor recognition of antigen-presenting molecules. Annual Review of Immunology, 33, 169–200.*

*Shen, P. & Fillatreau, S. (2015). Antibody-independent functions of B cells: a focus on cytokines. Nat Rev Immunol. 15(7):441–51.*

*Silvagni, E., Bortoluzzi, A., Carrara, G., et al. (2018). Comparative effectiveness of first-line biological monotherapy use in rheumatoid arthritis: a retrospective analysis of the RECord-linkage On Rheumatic Diseases study on health care administrative databases. BMJ Open 2018; 8: e021447.*

*Smolen, JS., Landewé, R., Bijlsma, J. et al. (2017). EULAR recommendations for the management of rheumatoid arthritis with synthetic and biological disease-modifying antirheumatic drugs: 2016 update. Ann Rheum Dis. 76(6):960-977.*

*Taylor, PC., Cerinic, MM., Alten, R., Avouac, J., Westhovens, R. (2022). Managing inadequate response to initial anti-TNF therapy in rheumatoid arthritis: optimising treatment outcomes. Therapeutic Advances in Musculoskeletal Disease. 2022, 14*

*Townsend, CL., Laffy, JM., Bryan-Wu, YC., O’Hare, JS., Martin, V., Kipling, D., Fraternali, F., Dunn-Walters, DK. (2016). Significant differences in physicochemical properties of human immunoglobulin kappa and lambda CDR3 regions. Frontiers in Immunology. 7-388*

*Trouw, L. A., Pickering, M. C., & Blom, A. M. (2017). The complement system as a potential therapeutic target in rheumatic disease. Nature Reviews. Rheumatology, 13(9), 538–547.*

*Trück, J., Eugster, A., Barennes, P., Tipton, C. M., Luning Prak, E. T., Bagnara, D., Soto, C., Sherkow, J. S., Payne, A. S., Lefranc, M. P., Farmer, A., Bostick, M., & Mariotti-Ferrandiz, E. (2021). Biological controls for standardization and interpretation of adaptive immune receptor repertoire profiling. ELife, 10.*

*Turchaninova, M. A., Davydov, A., Britanova, O. v., Shugay, M., Bikos, V., Egorov, E. S., Kirgizova, V. I., Merzlyak, E. M., Staroverov, D. B., Bolotin, D. A., Mamedov, I. Z., Izraelson, M., Logacheva, M. D., Kladova, O., Plevova, K., Pospisilova, S., & Chudakov, D. M. (2016). High-quality full-length immunoglobulin profiling with unique molecular barcoding. Nature Protocols 2016 11:9, 11(9), 1599–1616.*

*Wardemann, H., Yurasov, S., Schaefer, A., Young, JM., Meffre, E & Nussenzweig, MC. (2003). Predominent autoantibody production by early human B cell precursors. Science. 301(5638):1374-7.*

**Second round of review**

**Editorial board member**

As requested, I have tried to put myself in the place of reviewer 1 and answer if their concerns have been addressed satisfactorily.
The major outstanding point from Ref 1 is their request for longitudinal sampling from healthy individuals. My opinion is that this is a dataset that would certainly be nice to have, but is not essential to identify the treatment-induced changes, as argued by the authors, and probably falls out of scope of this study. As long as it is well discussed I would be happy with this.
I think most of ref1s comments are addressed well (3,5,6 especially). Q7 about the predictive model I found a little hard to understand, as I don't have experinec in this type of analysis, but I am prepared to give the authors the benefit of the doubt.
My only flagging concern relates to Q4 about the universal definition of clones based on CDR3 sequences, which is also a major feature of Ref2s response. There are certainly different definitions in the literature, but what concerns me is the author's response about somatic hypermutation in response to Ref2Q4. I am not convinced at all that they have adequately understood BCR specific features
Their statement below is telling - even 3.75% is often considered to be highly mutated at the BCR
"Since we have found that only a very small fraction of the BCR clones is hypermutated (e.g. <3.75% across chains; Figure 6c), it is unlikely that it had a major impact on the BCR longitudinal analysis."
Their statement "We are therefore convinced that NoisET is the most optimal approach available so far to identify expanded and contracted clones at the TCR, but also at the BCR level." is not at all supported by any data (I may have missed something). They make a similar statement on page 27. They need to tone this statement down considerably (unless they want to benchmark it properly), compare their BCR clonotype assignment and SHM calculation with another BCR-specific method (as this can be influenced by the clonotype assignment step).
My instinct would be to follow Ref 2 on this and to ask for another revision. I think the authors do still need to clarify/caveat/expand their discussion a bit more honestly about the limitations of their method.
As an aside, does GB have a policy about color-blind friendly images? Most of the color schemes in this paper are red-green and I think many would be impossible to read for color-blind individuals (8% of men and 0.5% of women).

**Reviewer 2**

The comments from the other reviewers are more important and I look forward to reading the response to those comments.

My comments were mostly technical, and I'll just provide a few further comments along those lines.

3. Multiple testing correction

The authors imply in their response that tests need to be independent in order to be corrected.

I offer this quote from https://onlinelibrary.wiley.com/doi/epdf/10.1002/sim.6082 :

"One of the most widespread of these misunderstandings is that the method would be based on an assumption of independence between p-values"

The authors also say that they are not looking at the most significant p-values but rather "we highlight the diversity associations that show the highest consistency across chains."

In any case, I just suggest that the authors clarify what they are doing: if the p-values are uncorrected, say so, and describe the consistency measure that was used.

9. The authors have said that they have clarified the bubble graphic, but all that is said is

"The bubbles shown in the right two columns are used to graphically illustrate the association results at the chain level"

Please describe what the bubbles are and how their size is determined by the data.

14. The authors explain the concentric pie charts with "each node is divided into as many fractions as individuals have the clone."

I'm afraid I still don't understand what the rings of the concentric pie charts represent, and what sets their radial width.

Finally, the fonts are much to small, even for individual plots, much less for the multi-panel graphics shown here.

**Reviewer 3**

The authors made efforts to address the reviewers' comments.

**Authors Response**

**Point-by-point responses to the reviewers’ comments:**

Editorial board member

As requested, I have tried to put myself in the place of reviewer 1 and answer if their concerns have been addressed satisfactorily.
The major outstanding point from Ref 1 is their request for longitudinal sampling from healthy individuals. My opinion is that this is a dataset that would certainly be nice to have, but is not essential to identify the treatment-induced changes, as argued by the authors, and probably falls out of scope of this study. As long as it is well discussed I would be happy with this.

*Response: We thank the editorial board member for thoroughly evaluating how we have addressed each of the comments originally raised by Reviewer #1. In line with our Response #2 to Reviewer #1, we totally agree that having the suggested dataset with longitudinal immunosequencing data from healthy donors could provide additional information for the present study. We are nonetheless convinced that our study design is highly robust to identify changes in the immune repertoire that are induced by TNF blocking, the objective of the present work. Following the indications of the editorial board member, we have now expanded the discussion of the revised manuscript to emphasize that contrasting our results with the immune repertoire changes expected in healthy individuals could further contribute to corroborate our findings (pg. 27).*

I think most of ref1s comments are addressed well (3,5,6 especially). Q7 about the predictive model I found a little hard to understand, as I don't have experinec in this type of analysis, but I am prepared to give the authors the benefit of the doubt.

*Response: We thank the editorial board member for considering that we have properly addressed almost all comments raised by Reviewer #1. We also appreciate his/her acceptance of our response to Q7 justifying the generalizability of our molecular predictor as well as its potential to be used in combination with serologic information to accurately predict all seronegative patients.*
My only flagging concern relates to Q4 about the universal definition of clones based on CDR3 sequences, which is also a major feature of Ref2s response. There are certainly different definitions in the literature, but what concerns me is the author's response about somatic hypermutation in response to Ref2Q4. I am not convinced at all that they have adequately understood BCR specific features
Their statement below is telling - even 3.75% is often considered to be highly mutated at the BCR
"Since we have found that only a very small fraction of the BCR clones is hypermutated (e.g. <3.75% across chains; Figure 6c), it is unlikely that it had a major impact on the BCR longitudinal analysis."

*Response: The two comments raised by the editorial board member address distinct questions from Reviewer #2. The initial concern revolves around the complexity of defining clones based on CDR3 sequences. In this regard, we totally agree that there exists no universally applicable definition for clones solely based on CDR3 sequences. This is precisely why, in addition to our established clone definition (excluding V-J gene annotation), we re-analyzed the immune repertoire of the study’s individuals using alternative clonal definitions that encompass CDR3 amino acid sequences along with V-J segments. Importantly, as elucidated previously in response to Q4 from Reviewer #2, these additional analyses unequivocally demonstrated that the choice of clonal definition exerts a virtually insignificant effect on our findings.*

*The second comment raised by the editorial board member pertains to the assertion in our previous response that NoisET method could extend its applicability to the B cell compartment with minimal influence from BCR-specific attributes like somatic hypermutation. Nevertheless, we acknowledge potential concerns associated with our initial statement intended to communicate that only a small proportion of BCR clones undertook somatic hypermutations. We understand now that, even in this scenario longitudinal, BCR analysis could be affected. We have also become aware of further important aspects that might render the use of NoisET in BCR analysis more challenging and, therefore, they deserve to be discussed in the manuscript. Accordingly, we have now modified the discussion of the revised manuscript accordingly (pg. 30).*
Their statement "We are therefore convinced that NoisET is the most optimal approach available so far to identify expanded and contracted clones at the TCR, but also at the BCR level." is not at all supported by any data (I may have missed something). They make a similar statement on page 27. They need to tone this statement down considerably (unless they want to benchmark it properly), compare their BCR clonotype assignment and SHM calculation with another BCR-specific method (as this can be influenced by the clonotype assignment step). My instinct would be to follow Ref 2 on this and to ask for another revision. I think the authors do still need to clarify/caveat/expand their discussion a bit more honestly about the limitations of their method.

*Response: We agree with the editorial board member that empirical data is needed to support our statement, as our claim might have been too assertive without sufficient analytical evidence behind it. We apologize for this assertion, which was formulated to convey the idea that, given the absence of alternative methods for longitudinal analysis, we considered that NoisET method could be valid for BCR analysis. However, while we are confident that NoisET is likely to outperform conventional methods such as Fisher’s exact test, we acknowledge that the foundational assumptions underlying NoisET algorithm are based on TCR properties and, therefore, precise performance is not inherently guaranteed. In line with this, we appreciate the editorial board member for affording us the opportunity to address the concerns regarding the applicability of NoisET to BCR analysis. Following his/her indications, we have not only softened our statement but also emphasized that the applicability of NoisET is particularly challenging and caution should be taken when employed in BCR analysis (pg. 30).*

*Following the reviewer indications, we have further computed the percentage of SHM using IgBlast, an alternative method that is commonly used for BCR analysis. Subsequently, we compared this additionally computed percentage of SHM with the estimation resulting from MiGEC/MiXCR analysis that is described in our original manuscript. Importantly, SHM rates estimated by IgBlast and MiGEC/MiXCR methods showed a strong and significant correlation across the six immunoglobulin isotypes here analyzed (P<0.01, Figure 1a). Although we acknowledge that the percentage of SHM was found to be generally higher when determined by IgBlast (Figure 2b), the capacity of IgBlast to distinguish SHM from PCR errors is substantially lower compared to MiXCR/MiGEC method and, consequently, this can lead to a significant rise of SHM that is driven by a high rate of sequencing errors. Unlike IgBlast, MiGEC/MiXCR method is designed to process UMI data minimizing the impact of sequencing errors like PCR duplicates and, therefore, this method offers us a strong degree of confidence to estimate SHM accurately. According to the relevance of the comparative analysis raised by the reviewer, we have now expanded and improved the description of SHM estimation in the revised version of the manuscript (pg. 19).*

*Figure 1. Comparative evaluation of somatic hypermutation estimation using IgBlast and MIGEC/MIXCR methods. a) Correlation between IgBlast- and MIGEC/MIXCR-estimated percentages of SHM at the isotype level. b) Percentage of SHM estimated using IgBlast and MIGEC/MIXCR methods for each isotype. p: p-value resulting from Wilcoxon test.*

*The editorial board member also underscores the importance of transparently discussing the limitations of the method used in our study. In line with this, we have now thoroughly revised the discussion of the manuscript to provide an expanded and more comprehensive assessment of the potential areas of improvement and limitations that are inherent to NoisET approach (pg. 30). We are confident that the updated discussion ensures that readers have a clear understanding of the boundaries and challenges associated with the method used for studying the dynamics of the immune repertoire.*
As an aside, does GB have a policy about color-blind friendly images? Most of the color schemes in this paper are red-green and I think many would be impossible to read for color-blind individuals (8% of men and 0.5% of women).

*Response: The reviewer's comment is correct. We would like to express our commitment to modify the figure colors if it is eventually required by the editorial board of Genome Biology journal.*

**Reviewer 2**

The comments from the other reviewers are more important and I look forward to reading the response to those comments.

My comments were mostly technical, and I'll just provide a few further comments along those lines.

*Response: We thank the reviewer for considering that we have satisfactorily addressed all technical comments raised in his/her review. We herein include a point-by-point response to each of the additional comments raised in this second review.*

3. Multiple testing correction

The authors imply in their response that tests need to be independent in order to be corrected.

I offer this quote from https://onlinelibrary.wiley.com/doi/epdf/10.1002/sim.6082 :

"One of the most widespread of these misunderstandings is that the method would be based on an assumption of independence between p-values"

The authors also say that they are not looking at the most significant p-values but rather "we highlight the diversity associations that show the highest consistency across chains."

In any case, I just suggest that the authors clarify what they are doing: if the p-values are uncorrected, say so, and describe the consistency measure that was used.

*Response: We thank the reviewer for his/her constructive comment as well as for indicating how the multiple testing aspect could be addressed in the manuscript. Firstly, instead of using any consistency measure, we would like to clarify that we have employed the term “consistency” to describe those associations that are aligned in both direction and significance across chains or diversity measures. Secondly, in response to the reviewer’s suggestion, we have now emphasized in the results section to which the reviewer is referring that p-values are uncorrected (pg. 6).*

9. The authors have said that they have clarified the bubble graphic, but all that is said is

"The bubbles shown in the right two columns are used to graphically illustrate the association results at the chain level"

Please describe what the bubbles are and how their size is determined by the data.

*Response: We apologize for not having properly clarified the meaning of the bubbles represented in Fig 1b and Fig 1c. For the sake of facilitating the interpretation of our diversity findings, we have represented clones as bubbles and their size has been set proportional to their frequency. Low and high diversity repertoires are therefore illustrated with a few and many large bubbles, respectively. Nonetheless, we would like to make clear that this graphical representation was not fed with our data. Instead, as previously done (Bashford-Rogers et al. Nature ‘19), we have harnessed this illustrative strategy to facilitate the interpretation of the accompanying heatmaps. We are convinced that the adjacent heatmaps can be better understood when presented together with bubble plots, but we are totally open to removing this section from Fig 1b and Fig 1c if the reviewer confirms that it can be misleading.*

14. The authors explain the concentric pie charts with "each node is divided into as many fractions as individuals have the clone."

I'm afraid I still don't understand what the rings of the concentric pie charts represent, and what sets their radial width.

*Response: We also apologize for not having clarified the immune repertoire information that is represented by concentric pie charts. Using the iR-RepSeq+ 7-Chain technology (iRepertoire, US), we have quantified TCR and BCR clones (i.e., CDR3 sequences) that are circulating in a particular individual at a given moment. These quantitative results showed that CDR3 sequences are not evenly detected in all individuals. To graphically represent this information, we have divided each clone of interest (i.e., node) into as many fractions as individuals with the indicated clone in circulation. For instance, the significantly contracted CDR3 sequence CSSYTSSSTWF from IGL chain was detected in 15 out of 77 RA patients and, consequently, the node representing this clone in Fig 2e has been illustrated using a pie chart that is divided into 15 fractions.*

*Furthermore, we would like to clarify that the radial width of a given clone (i.e., node) is proportional to its degree centrality value within the network, being this quantitative measure informative of the number of clones that differ by at most one amino acid with the indicated clone. Accordingly, large clones represent CDR3 amino acid sequences that are highly similar to the sequence of many other clones from the set of interest (e.g., contracted clones). Likewise, small clones are barely linked to other clones indicating that their CDR3 amino acid sequence differs from the sequence of the set of interest. This is the message that we wanted to convey with the network representation used in Fig 2e. To avoid any misinterpretation, we have now clarified the meaning of the radial width in the revised manuscript (Figure 2 legend; pg. 36).*

*As raised by the reviewer, we think that pie charts displayed specifically for clones that are contracted by TNF inhibition in responders look concentric and, therefore, different from the others. However, we confirm that we used exactly the same Kamanda-Kawai layout (igraph R package) that we used to represent expanded clones from non-responders and both expanded and contracted clones from responders. Hence, the important features of the graphical representation to convey our longitudinal findings are clone size, divisions, and edges, rather than the layout.*

Finally, the fonts are much to small, even for individual plots, much less for the multi-panel graphics shown here.

*Response: We agree with the reviewer that, particularly for multi-panel graphics, the font size could be considered small. However, recently published studies in top-tier journals of reference have also used font sizes smaller than usual to fit all findings derived from the analysis of high throughput data within a single figure (e.g., Figs 1-6 from Garrido-Trigo A et al. Nature Communications ‘23). We guarantee that all figures incorporated into our manuscript are of high resolution, allowing for seamless zooming of up to 400% when needed.*

Reviewer #3

The authors made efforts to address the reviewers' comments.

*Response: We thank the reviewer for appreciating the effort that we have made to properly address all comments raised in his/her review.*

**Third round of review**

**Editorial board member**

I have just now reviewed the manuscript and response to reviewers. I feel pretty satisfied with their responses, and more measured discussion of the limitations of their approach. Inclusion of discussion and new analyses around SHM is important and address my major concern and that of reviewer 1's Q4. I think reviewer 2's minor comments all look good as well, although agree that the figure image quality (presentation, font size etc) is improved a bit.

**Authors Response**

**Point-by-point responses to the reviewers’ comments:**

Editorial board member

I have just now reviewed the manuscript and response to reviewers. I feel pretty satisfied with their responses, and more measured discussion of the limitations of their approach. Inclusion of discussion and new analyses around SHM is important and address my major concern and that of reviewer 1's Q4. I think reviewer 2's minor comments all look good as well, although agree that the figure image quality (presentation, font size etc) is improved a bit.
